# Stable closure of acute and chronic wounds and pressure ulcers and control of draining fistulas from osteomyelitis in persons with spinal cord injuries: non-interventional study of MPPT passive immunotherapy delivered via telemedicine in community care

**DOI:** 10.3389/fmed.2023.1279100

**Published:** 2024-01-05

**Authors:** Jeanette Sams-Dodd, Maurizio Belci, Surendra Bandi, Damian Smith, Frank Sams-Dodd

**Affiliations:** ^1^Willingsford Ltd., Southampton, Hampshire, United Kingdom; ^2^The National Spinal Injuries Centre, Stoke Mandeville Hospital, Aylesbury, Buckinghamshire, United Kingdom; ^3^Duke of Cornwall Spinal Treatment Centre, Salisbury District Hospital, Salisbury, Wiltshire, United Kingdom

**Keywords:** spinal cord injury, infection, osteomyelitis, wound, pressure ulcer, immunosuppression, antimicrobial resistance, sustainability

## Abstract

**Background:**

Micropore particle technology (MPPT) is a topical wound treatment. It is a passive immunotherapy, acting via the skin and wound microbiome without the use of antimicrobial action. In a general patient population, it removed wound infections 60% and initiated tissue regeneration 50% quicker than antibiotics and antiseptics. As MPPT supports the immune system, the aim was to confirm that MPPT is also effective in immunocompromised individuals. People with spinal cord injury (SCI) are immunodeficient due to their injury and not an underlying disease and recruit 50% fewer immune cells to an injury. The study, therefore, determined the efficacy, safety, health economics, and sustainability of MPPT in acute and chronic wounds and pressure ulcers in this patient population.

**Methods:**

Pressure ulcers in SCI persons are an orphan indication, patient variability is high, and ICH E10 excludes comparators due to ethical concerns. The study design was, therefore, a single-arm, non-interventional, observational, post-market surveillance study of MPPT for treating wounds and pressure ulcers and removing soft tissue infection in connection with draining fistulas in SCI persons. The study was based on telemedicine in community care.

**Results:**

The study included 44 wounds. All acute and chronic grade 1–4 wounds and pressure ulcers reached stable closure. In wounds acting as fistulas draining from an underlying, primary focus of infection, e.g., osteomyelitis, MPPT removed the soft tissue infection in approx. 2.5 months and supported regeneration, considerably reducing fistula sizes. Compared to standard care, per-wound cost savings were 51 to 94% depending on wound grade and age, and substantial nursing resources were freed up. The telemedicine approach was well received by participants and supported independence and self-care. The use of antimicrobials, plastics, and synthetic polymers was essentially eliminated. MPPT did not require bed rest.

**Conclusion:**

The study confirmed that MPPT is safe and effective in treating acute and chronic wounds in immunocompetent and immunocompromised individuals, including wounds with antimicrobial-resistant infections. MPPT also removes soft tissue infections caused by an underlying primary focus of infection, such as osteomyelitis. Non-healing wounds currently represent an unmet clinical need. The findings suggest that a therapy acting via the microbiome without antimicrobial actions is effective.

## 1 Introduction

The US FDA stated in 2022 that wounds failing to naturally undergo an orderly healing process constitute an unmet medical need due to the lack of effective treatments (1). The result, in the UK, is an annual growth of 11% in the number of wounds requiring treatment by the National Health Service (NHS) (2). Over 10% of the population at any one time has a wound that requires attention by a healthcare professional. In 2022, the NHS will treat 6.3 million wounds in community care, with a total cost of £34.4 billion (Supplementary material S1). According to Kings Fund (3), the NHS funding in 2022 will be £173.8 billion, which means that 20.1% of the 2022 NHS budget will be allocated to wounds in community care. The need for innovative treatments is consequently urgent. However, the FDA identified complex and poorly understood wound healing processes, difficulties in conducting clinical trials, e.g., difficulties with patient enrolment, heterogeneous study designs with varying standard of care protocols and study populations, and difficulty achieving the most commonly utilized primary efficacy endpoint of complete wound healing, and, once approved, complicated reimbursement processes, as reasons for limited development of new treatments in the field (1).

Most treatment approaches focus on removing microorganisms in wounds, but research over the past 20 years has found that all external body surfaces incorporate microbiomes, i.e., organized, diverse, synergistic communities of bacteria, archaea, viruses, protozoa, fungi, and mites, which extend into deeper dermal layers and aid in the body’s protection and health (Supplementary material S2), e.g., they are involved in keeping the skin healthy and in activating the immune response in the event of developing infection (4–6). Microbiomes are closely controlled by the immune system, and infection is when one or a few species of microorganisms take control of the wound from the immune system. The infective organisms are usually commensals that live naturally in or on the human skin. Bacterial presence is required for wound regeneration and healing (7), and the aim of treatment is not to eradicate them but to return control to the immune system for it to re-establish balance in the microbiome (Supplementary material S2). Both antibiotics and antiseptics are standard care for infected wounds, and both contribute to and are limited by antimicrobial resistance (8–16). On a wound, they will, therefore, selectively kill the non-resistant strains, and this will favor the resistant strains, which typically are more virulent (17). The result is that the infection and the pathology are exacerbated (10, 18, 19). This is consistent with antimicrobials not having been shown effective in treating wound infections or supporting healing (20–22).

Micropore particle technology (MPPT) (Supplementary material S3) is a novel technology that interacts with the skin and wound microbiomes to assist the immune system in regaining control of the wound environment, thereby enabling the immune cells to remove infection and advance healing (23). MPPT uses physical forces, i.e., capillary evaporation or the pumping of microscopic amounts of moisture away from the wound and skin surface, (1) to remove the toxins, enzymes, and signaling molecules that bacteria and fungi release to kill, inactivate, or inhibit immune cells and other microorganisms; and (2) to disrupt the structure of the biofilm, a gelatinous shield produced by bacteria and fungi to protect the microbiome against immune cells and other adversaries (23). This disarmament of both offensive and defensive weaponry returns control to the immune system. This was confirmed in a preclinical wound healing model (24), where MPPT, compared to the topical antibiotic gentamicin, led to a 107% (2.1-fold) increase in the overall number of immune cells in the wound, including a 24.8-fold increase in the number of macrophages and a 7.2-fold increase in lymphocytes (23, 24). The level of immune cells in the gentamicin group was similar to untreated controls. Wound colonization in terms of the number of bacteria, however, was similar in the MPPT and untreated control groups, demonstrating that MPPT is not antimicrobial. As expected, gentamicin reduced the bacterial count.

The clinical benefits of these effects have been confirmed. A 266-patient RCT (25) found that MPPT removes wound infections 60% and initiates tissue regeneration 50% quicker than antibiotics (gentamicin) and antiseptics (iodine) in surgical wounds, abscesses, diabetic foot ulcers, venous leg ulcers, and burns. In dehisced surgical wounds (26), the use of MPPT for 3–5 days resulted in a healing wound suitable for discharge into community care, whereas standard care, consisting of debridement followed by negative pressure wound therapy (NPWT), required 3 weeks or more to reach a similar stage, i.e., a reduction of 81% by using MPPT. MPPT has also improved wounds caused by pyoderma gangrenosum (27) and effectively treated necrotizing fasciitis (28). No adverse events have been seen (23, 25, 26, 29, 30).

MPPT, therefore, builds upon a new understanding of wound healing and represents a novel treatment approach. Clinical studies have confirmed the ability of MPPT to treat wound infections and to support tissue regeneration and wound closure in a wide patient population, but since the effects of MPPT depend upon the immune system, it was important both scientifically and medically to confirm that it retains its efficacy in confirmed immunocompromised individuals. These individuals can only recruit reduced numbers of immune cells to a wound, and the aim was to investigate whether the support provided by MPPT is sufficient to achieve the desired outcome of wound closure.

Persons with spinal cord injury (SCI) are immunodeficient (Supplementary material S4) due to the loss of communication between the immune system and the nervous system. SCI results in a 50% reduction in the number of macrophages recruited to a wound (31, 32) and leads to strongly impaired wound healing (33–37) as well as changes in their microbiomes (38). Furthermore, data suggest that pressure ulcers and their consequences account for 25% of the total costs of healthcare for SCI (39) and that pressure ulcers account directly for the death of 10 to 12% of SCI persons (40–42). Once a wound or pressure ulcer penetrates the anatomical and immunological barriers of the skin reaching muscle (grade 4), 32% of a general patient population will develop osteomyelitis within a median of 4 months (43, 44), and this proportion will be even higher in SCI. Once present, the osteomyelitis will continuously release debris into the surrounding tissue, leading to collections and the formation of a draining fistula that typically gives rise to an uncontrolled festering wound. Osteomyelitis requires surgical removal (45), but Russell et al. (44) reported surgical failure rates of 71% for patients with osteomyelitis caused by pelvic pressure ulcers and median survival times of only 2 years from the first surgery if the wound does not heal (64% of patients) and only 7 years even if the wound does heal (36% of patients). Demonstrating efficacy in treating wounds and pressure ulcers and in controlling soft tissue infection caused by an underlying primary focus of infection, e.g., osteomyelitis, in SCI persons would, therefore, confirm the efficacy of MPPT in people with an impaired immune response. In addition to the importance for people with SCI to have access to effective treatment of their wounds, these data are equally relevant for wound healing in patients who are immunocompromised for other reasons, e.g., long-term illness, cancer, or trauma. The scientific importance of these data is to understand whether the clinical efficacy of an approach acting via the microbiome to support the immune system is retained in immunocompromised patients.

The FDA highlighted difficulties regarding clinical trial design. A traditional RCT was considered but deemed unethical because ICH guidance (46) excludes the use of comparators when (1) these are known to be subeffective (22, 47); (2) participants are at risk of irreversible morbidity and death; and (3) the study treatment has previously demonstrated efficacy (25, 26) (Supplementary material S5). Moreover, pressure ulcers in SCI persons are an orphan indication, which means the recruitment rate would be very low and patient variability high, which means that homogeneous groups allowing cross-group comparisons are difficult to achieve (48). Based on these considerations, a single-arm non-interventional observational study with wide inclusion criteria was chosen as it is likely to provide real-world evidence on the outcome of using MPPT (49). A limitation of single-arm studies is the lack of internal comparators, and to overcome this, the US FDA recommends the use of external controls representing comparable conditions (50). As the study focused on routine clinical community care, Guest et al. (51) for acute wounds and Bennett et al. (52) for chronic wounds and draining fistulas could be used as external comparators as they report on the outcome of routine clinical care in a community setting (50)(Supplementary material S6).

The primary aim of the study was, therefore, to confirm the efficacy of MPPT in an immunocompromised population to demonstrate that MPPT could support the remaining immune function to achieve an effective clinical outcome. The study used MPPT to treat wounds and pressure ulcers in SCI persons to determine its ability to achieve wound closure or, if osteomyelitis had developed, to control the associated soft tissue infection. This would extend previous findings to a group of highly immunocompromised individuals and show whether an approach based on interactions with the microbiome, without the use of antimicrobials, could be effective in treating wound infections and supporting regeneration across wound types and immune status. The findings would be relevant to wound healing in general as MPPT acts on the wound infection, which usually is the primary reason for non-healing, and supports tissue regeneration.

The study also sought to determine whether MPPT treatment could successfully be delivered via telemedicine, with patients, family members, or carers being responsible for the hands-on treatment procedures (53). Community nurses currently spend over 50% of their time on wound care (54), and the implementation of an effective treatment delivered via telemedicine could free up substantial resources.

The manuscript will first present the clinical outcome of treating acute wounds, chronic wounds, and draining fistulas, followed by evaluations of health economics and environmental sustainability.

## 2 Materials and methods

### 2.1 Study design

The study was a UK non-interventional, observational, post-market surveillance study of the CE-marked medical device, MPPT, for treating wounds and pressure ulcers and controlling soft tissue infection in connection with draining fistulas in persons with spinal cord injury. Inclusion criteria were a wound or a pressure ulcer of any age located below the site of a traumatic or non-traumatic injury of the spinal cord. It could have been exposed to other treatments. Participants who consistently, despite guidance, did not follow Instructions for Use is regulatory document were omitted from the study analysis. By including all wounds fulfilling these criteria during the inclusion period, a representative sample of the patient population was obtained. Wounds were followed to closure or until the cause of non-healing had been identified. Where possible, draining fistulas were followed for longer to establish the benefits and safety of long-term use. Post-market surveillance is a regulatory requirement for medical device companies, and in the UK, post-market surveillance studies are not subject to ethical approvals as they fall within normal clinical practice and are not classified as research (55). MPPT was used in accordance with its approved use; participants were not assigned to treatment; the decision to use MPPT was separate from the decision to include the participant in the study; no diagnostic or monitoring procedures were applied other than those which are ordinarily applied in the course of normal clinical practice; and only epidemiological methods were used in the analysis.

The inclusion period was from 2017 to 2020, and the study period was from 2017 to 2021. Individuals would learn of MPPT through the National Spinal Injuries Centre, Stoke Mandeville Hospital, Aylesbury, UK; The Duke of Cornwall Spinal Treatment Centre, Salisbury, UK; Defence Medical Rehabilitation Centre (DMRC), Stanford Hall, UK; NHS Wiltshire Community Care, UK; SIA (Spinal Injuries Association), UK; as well as by word-of-mouth and closed SCI community Facebook groups of which no-one from Willingsford was a member. Individuals would contact Willingsford and be requested to provide pictures of their wounds and information on prior treatment history to determine the suitability of MPPT for their wounds and be provided guidance on what to expect. No wounds or ulcers suitable for MPPT treatment were dismissed. Based on this, the individual would decide whether to use MPPT. In most cases, the individual would self-fund the MPPT. All participants provided written permission for the anonymized use of their medical history related to the wound and their pictures for medical, scientific, and informational purposes.

### 2.2 Wound treatment

MPPT (tradenames Amicapsil® and Amicapsil-SCI®) is a CE-marked medical device. It is in powder form and approved as a treatment for wounds, i.e., with a therapeutic outcome. Once daily (see Supplementary material S3 for detailed description), the wound was thoroughly washed, preferably showered, using plenty of clean tap water. No surfactants, e.g., wound rinsing solutions or soaps, were used because they are antimicrobial. Standing water was gently removed by dabbing, and MPPT was applied in an unbroken 1–2 mm layer to the entire wound surface and wound edges, including 5–10 mm beyond onto healthy skin. If no dressing would be worn, the thickness was determined by how much would stick to the surface, but the layer would always be unbroken. Red, irritated, inflamed, nodulous, or cracked areas of skin surrounding any type of wound or in proximity to the wound had MPPT gently and briefly massaged into the affected skin and 5–10 mm beyond onto healthy skin.

MPPT acts via micro-evaporation of moisture and requires air circulation across the wound surface. Depending on individual circumstances, the wound was either left uncovered or covered with a single, woven, 100% pure cotton gauze swab.

Participants were encouraged to minimize bed rest. When air was prevented from accessing the wound, e.g. when the participant sat or rested on the wound, air to the wound surface was supplied using a small portable air-pump. In bone-debris-draining fistulas, this could be combined with absorbent wound dressings, providing the participant full freedom.

Wounds in immunocompromised persons generally require daily application until closure. The use of antibiotics, e.g., for treating UTIs, can decelerate healing and might even cause a flare-up of wounds and/or bone infections caused by resistant species, thereby extending the MPPT treatment period.

### 2.3 Telemedicine

Participants were living in their own homes, and the hands-on treatment was performed by themselves, family members, carers, and, for one participant, when at the DMRC, by nurses. Participants took photos daily of key steps during the treatment regime and these, along with any comments or questions, were emailed to an external wound expert for daily evaluation and advice on how to proceed at the next treatment session.

### 2.4 Outcome measures

Clinically relevant outcome measures were chosen (Supplementary material S6).

Adverse event information attributable to MPPT, e.g., wound irritation, allergic reactions, and bleeding, was based on participant reporting during the daily correspondence and on analysis of the daily pictures received by the wound experts with subsequent confirmation by the participant. MPPT is classified as inherently safe (ISO 13485:2016 and ISO 14971:2019).

Efficacy measurements for acute and chronic wounds were closure rate, and days to closure; and for draining fistulas, were the control of soft tissue infection relative to start of treatment.

For health economic calculations, the number of bottles of MPPT based on delivery notes and days of receiving advice from wound experts based on e-mail communication was recorded. For draining fistulas, it was assumed that the bottles delivered were used evenly across days until the next delivery. For cost calculation, a unit price of £119.84 per bottle of 750 mg MPPT and £65 per day for evaluating pictures and providing comments were used. The cost per evaluation was set to equal the cost of a specialist nurse visit in community care, meaning that either telemedicine or nurse visits can be chosen as an approach without impacting calculated savings. Using these numbers, the cost per wound was calculated. If a participant had more than one wound, the costs were not divided across wounds but applied in full for each wound to ensure that the costs would be valid independently of whether the person had one or more wounds. Moreover, if treatment was only needed for a few days and the entire bottle was not used, the full cost of the bottle was recorded.

The use of MPPT on a draining fistula was considered an improvement if, in general, the level of necrotic tissue and slough, the number of disseminated abscesses, cellulitis, and odor were reduced, and if granulation and epithelialization occurred. Overall, such improvement can be summed up as a considerable reduction in the generalized soft tissue infection and improved control of the draining fistula. The analysis of improvement took the state and progression of the primary focus of infection, e.g., osteomyelitis, into consideration (For details, please see Supplementary materials S6, S9).

The EPUAP/NPUAP Pressure Injury Staging (56) was used for the classification of wounds and ulcers as it reflects the level of penetration through the anatomical and immunological (Supplementary material S2) barriers of the skin.

## 3 Results

### 3.1 Patients

A total of 28 persons with spinal cord injuries were included in the study. Two participants were forced to withdraw after a few days due to external circumstances despite their wounds responding well to MPPT, and one participant was excluded due to severe non-compliance. The mean age of the remaining 25 participants was 54.4 ± 14.2 years, ranging from 35 to 84 years. Participants were of different racial origins, representing different types of skin structure. One participant withdrew prematurely due to personal circumstances, but data are included. The gender ratio was approx. 2:3 (female:male), and the distribution of para vs. tetraplegic was 1:1.

**Figure 1:**
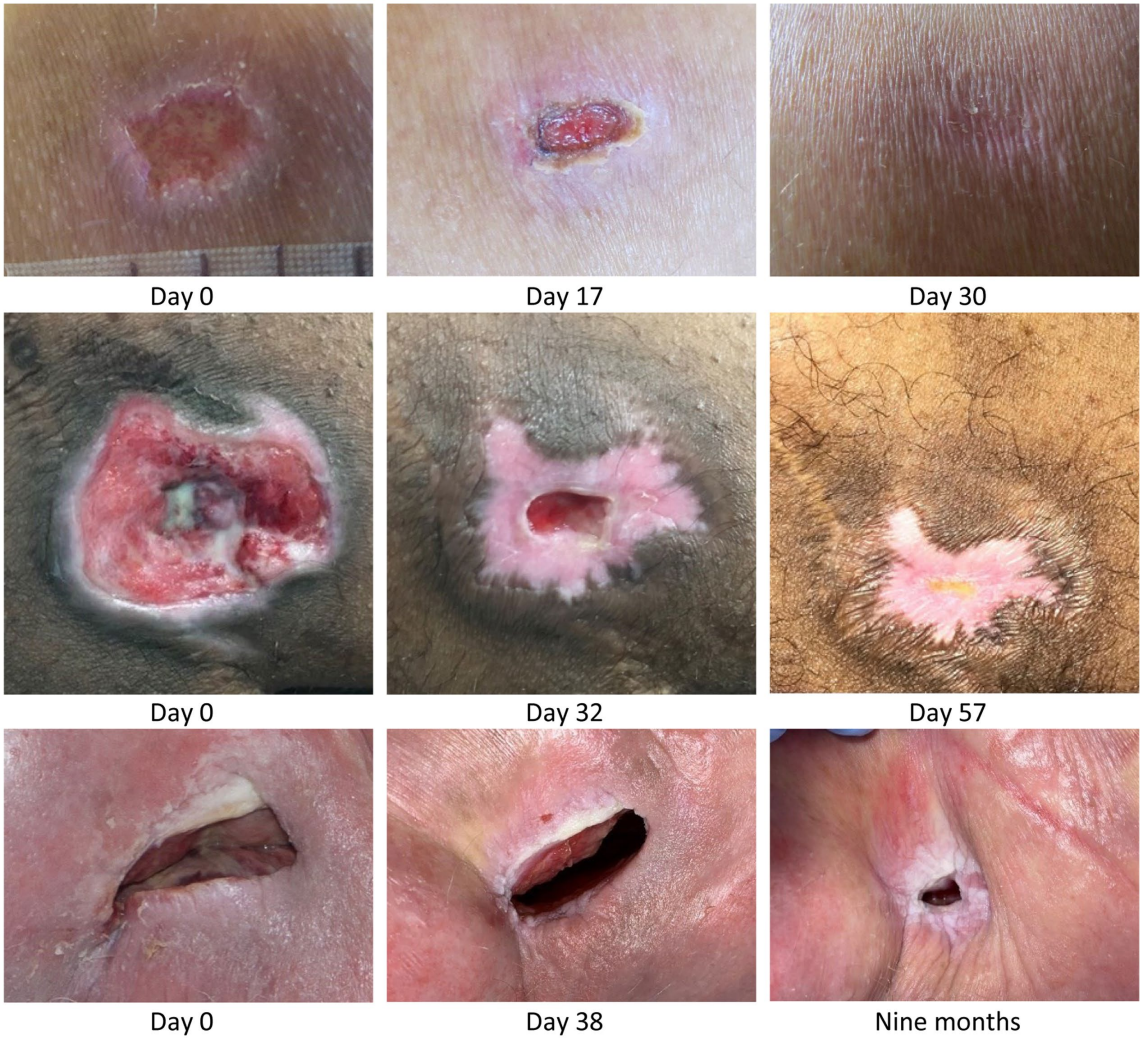
*Top row*: Acute grade 3 pressure ulcer over *tuber ischiadicum* in 47-year-old paraplegic male patient, L1/L2 & T9/T10 complete. An infected 2-week-old non-healing, deteriorating pressure ulcer was changed to MPPT. The wound was treated daily but sub-optimally between Day 5 and Day 16. Within a total of 4 weeks, the wound was fully closed with minimal scarring. *Center row*: Chronic 9 weeks-old, ischial tuberosity pressure ulcer in 34-year-old tetraplegic male patient, C4/5 incomplete. The patient had had flap-surgery in the area 7 years earlier. MPPT closed the wound in 2 months followed by 8 months of cycles with minor expulsions of infection followed by closure, until the wound 10 months after start reached fully stable closure. *Bottom row*: Many years old ischial tuberosity ulcer acting as draining fistula from confirmed osteomyelitis in a 84-year-old paraplegic female patient, T5 complete. At start, the wound consisted of a skin opening 6 cm × 2 cm leading into an expanding 10 cm deep cavity that led into 2 additional, wide, 3-cm deep tunnels in both directions along palpable bone. After 6 months with MPPT, the extent of the fistula had reduced to a skin opening 1.5 cm × 0.5 cm leading into 3 narrow 2–3 mm wide, 3–4 cm deep tunnels. All pictures are shown at scale to allow direct comparison of wound opening size. See Supplementary material S7: *Monitoring infective organisms* for a description of how the release of bacterial toxins was used to monitor wound status.

The study included 44 wounds (38 pressure ulcers, 3 trauma, 1 burn, 1 abscess, and 1 radiation) (Table 1; Figure 1), which fell into three groups: (1) 21 acute wounds less than 6 weeks old; (2) 10 chronic wounds 6 weeks and older without an underlying, primary focus of infection; and (3) 13 wounds acting as draining fistulas from an underlying, primary focus of infection, i.e., osteomyelitis or an anal fistula. Some acute wounds and all chronic wounds and draining fistulas had received or were receiving antimicrobials without achieving closure or control of the soft tissue infection. There were an equal number and level of severity of acute wounds on the lower legs and feet compared to acute wounds in the pelvic area, but for the chronic wounds and draining fistulas (wounds with osteomyelitis), the distribution was skewed with only 2 on the ankle and 21 wounds in the pelvic region. No correlation between healing and age, gender, or level of injury was seen.

**Table 1:** Outcome of study for primary efficacy and cost endpoints.

	N	Age median (range)	Outcome	Days to closure median (range)	Costs MPPT median (range)	Total Costs median (range)
Acute						
Grade 1–2	10	<7 days	100% closure	7 days	£120 (£120 to £120)	£217 (£120 to £770)
Grade 3–4	11	7 days (<7 to 21 days)	100% closure	47.5 days (25 to 63 days)	£240 (£120 to £599)	£1,420 (£184 to £4,455)
Chronic						
Grade 3	6	19.5 months (3 to 144 months)	100% closure	75 days (23 to 243 days)	£539 (£240 to £1,438)	£3,584 (£1,735 to £8,003)
Grade 4	4	4 months (2 to 132 months)	100% closure	183 days (72 to 313 days)	£1,618 (£360 to £3,955)	£7,966 (£3,220 to £9,870)
Underlying primary focus of infection						
Draining fistula	13	18 months (2 to 62 months)	100% clear improvement	Month 1	£1,678/month (£306–£12,720)	£3,638/month (£2,256–£14,670)
				Month 12	£495/month (£99–£4,670)	£560/month (£164–£4,735)

All acute and chronic wounds are closed. The cost of one MPPT 750 mg bottle was £119.84 and one evaluation £65/day; the cost per evaluation was set to equal the price of a specialist nurse visit in community care, meaning that either telemedicine or nurse visits can be chosen as an approach without impacting calculated savings. The number of evaluations for chronic wounds was calculated as daily for the first month and then every third day until closure. For Draining Fistulas, the costs include daily evaluations in the first month, every second day in the second month, every third day in the third month, and thereafter once monthly. Family and carers were responsible for hands-on dressing changes and rapidly learned to manage the wound independently.

Supplementary material S6 contains detailed results in all subsequent sections.

### 3.2 Safety

No adverse events or side effects were observed or reported. MPPT was used daily on the same area for more than 6 months by 11 participants and for more than 12 months by 5 patients; the latter included direct daily application onto bone with chronic osteomyelitis, and one participant was on anticoagulant therapy. No adverse effects on skin, muscle, tendon, or bone were observed, and MPPT was not, including in hyperallergic participants, associated with any allergy, irritation, or bleeding. MPPT retains a certain moist level on the wound surface and prevents desiccation.

Skin structure is affected by racial origin (57), and MPPT was found safe and effective across different skin structures.

### 3.3 Acute and chronic wounds

**Figure 2:**
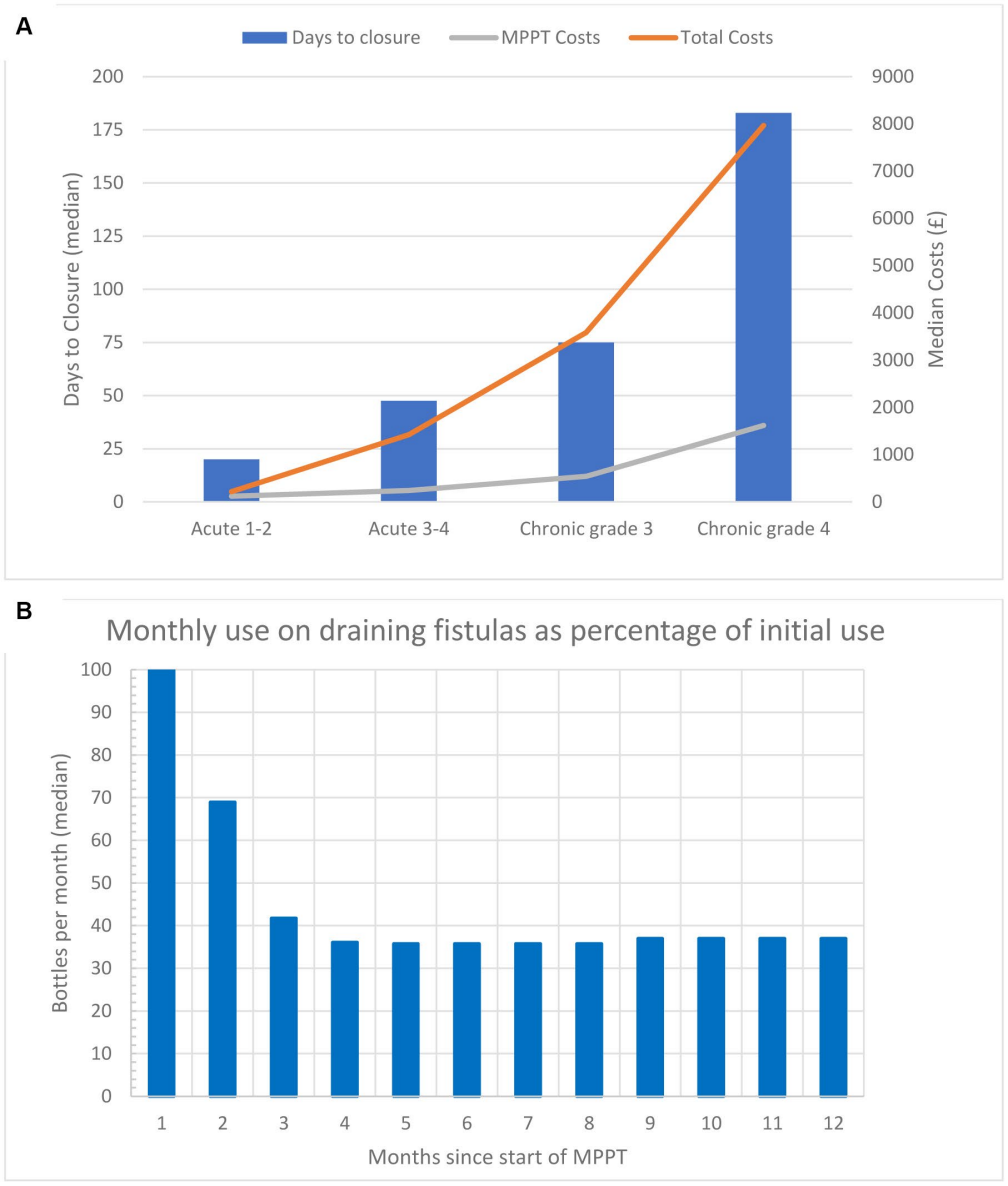
**(A)** Median time to closure and median cost per wound (costs of MPPT and total costs) to reach closure for acute (less than 6 weeks old) and chronic wounds (6 weeks or older) in the study. Both parameters show an exponential increase as the severity of the wounds increases. **(B)** Monthly amount (median) of MPPT, expressed as a percentage relative to the first month of treatment, used for treating 12 wounds acting as draining fistulas caused by an underlying primary focus of infection, e.g., osteomyelitis or anal fistula. The data are also given in Table 2.

All acute and chronic wounds are closed with MPPT treatment. Tables 1, 2, and Figure 2A show how days to closure and costs increase with increasing wound grade and wound age at the start of MPPT treatment. It is worth noting that the median age at the start of the chronic grade 3 and 4 wounds (see Supplementary material S8 for presentation of individual wounds) were 19.5 and 4 months, respectively. This is consistent with grade 4 wounds having a considerably higher risk of developing osteomyelitis (43, 44) as the wound has penetrated all anatomical barriers and escaped the specialized immune system of the skin (Supplementary material S2), which is located in the layers above the muscle and bone where it limits the spread of the infection.

**Table 2:** Mean ± standard deviation and median with 25 and 75% quantiles.

(A)	Acute grade 1–2		Acute grade 3–4		Chronic grade 3		Chronic grade 4	
	Mean ± SD	Median (Q1; Q3)	Mean ± SD	Median (Q1; Q3)	Mean ± SD	Median (Q1; Q3)	Mean ± SD	Median (Q1; Q3)
Days to closure	21 ± 0	21 (21;21)	49 ± 13	48 (38;58)	98 ± 85	75 (35;133)	188 ± 102	183 (133;238)
MPPT Bottles used	1.0 ± 0	1 (1;1)	2 ± 1	2 (1;3)	5 ± 4	5 (3;6)	16 ± 13	14 (8;22)
Wound evaluations	2.4 ± 2.8	1.5 (1;2.8)	28 ± 20	20 (15;38)	52 ± 29	45 (32;64)	83 ± 34	81 (64;99)
MPPT costs (£)	120 ± 0	120 (120;120)	252 ± 152	240 (120;300)	620 ± 445	539 (300;689)	1,887 ± 1,564	1,618 (899;2,607)
Evaluation costs (£)	156 ± 184	98 (65;179)	1,788 ± 1,297	1,300 (943;2,470)	3,380 ± 1,891	2,925 (2,053;4,187)	5,368 ± 2,200	5,265 (4,176;6,457)
Total costs (£)	276 ± 184	217 (185;299)	2,039 ± 1,402	1,420 (1,062;2,889)	3,999 ± 1,890	3,584 (2,412; 4,696)	7,255 ± 2,999	7,966 (5,884;9,338)

(B)	Percentage MPPT used monthly relative to first month from start of treatment											
	1	2	3	4	5	6	7	8	9	10	11	12
Mean ± SD	100 ± 0	68 ± 28	59 ± 38	57 ± 50	54 ± 42	47 ± 31	45 ± 28	41 ± 21	39 ± 17	42 ± 20	42 ± 17	40 ± 13
Median (Q1;Q3)	100 (100;100)	69 (41;88)	42 (33;75)	36 (30;63)	36 (27;71)	36 (29;56)	36 (29;54)	36 (27;49)	37 (30;49)	37 (30;55)	37 (30;57)	37 (30;47)

(A) Outcome parameters for acute and chronic wounds, corresponding to Figure 2A. For acute grade 1–2 wounds, most participants did not report an exact closing date because the wound was uncomplicated and generally had a severe wound they were more concerned about. When asked, they had, therefore, forgotten the exact closure date. The only reported closure date was, therefore, 21 days for 1 wound, which is the only one included. However, based on general correspondence, it could be observed that the period for resolving a grade 1 ulcer was 1–5 days and for grade 2 was around 1–2 weeks. (B) Monthly use of MPPT for treating the draining fistulas for the first year, corresponding to Figure 2B. For health economic calculations, mean values were used to align with the data reported by Guest et al. (51) and Bennet et al. (52).

The efficacy of MPPT was not explicable simply by the change in wound dressing procedures, e.g., the use of tap water and allowing air to the wound surface, because in a couple of cases, the MPPT had in error been damaged (by exposure to heat) and this clearly resulted in the loss of efficacy until new MPPT was supplied.

### 3.4 Fistulas draining from an underlying, primary focus of infection

In all wounds acting as draining fistulas, MPPT reduced the soft tissue infection, supported tissue regeneration, and maintained control of the draining fistula (Supplementary material S9). The treatment is unavoidably symptomatic since it cannot treat the underlying primary focus of infection, but controlling the soft tissue infection as well as the toxins (see Supplementary material S7) and other harmful debris draining from the primary infection considerably reduces the risk of toxemia and sepsis and improves wellbeing. Moreover, participants were not required to remain on bed rest, and they were able to assume responsibility for the daily dressing changes, two aspects that strongly supported independence and self-care.

In five cases, extensive cellulitis was present in a wide area around the wound opening. It was removed by MPPT, and the skin was restored to its natural structure.

During the study, several patients required antibiotic treatment for non-wound related conditions, such as UTIs, GI infections, toothache, or flare-ups of their osteomyelitis. This generally caused wound healing to slow down or, in a few instances, to stall while the antibiotics were taken. Generally, following a course of antibiotics, the infective debris seemed to return stronger, and to maintain the draining canal free of infection, slightly more MPPT was, in some cases, needed temporarily. In case a more serious condition than a wound develops, it has been shown that the body will redirect its resources to this condition and that healing will consequently slow down (58).

Figure 2B and Table 2 shows the amount of MPPT used during the first 12 months of treatment as a percent reduction relative to the first month. The wounds were generally festering and out of control at the start, but over 2.5 months, they gradually came under control, and after Month 4, the required amount of MPPT had reduced by 63% compared to the start. Table 1 compares the 1st and 12th months of monthly cost of MPPT and management. They fell by 63 and 85%, respectively, as the need for MPPT and assistance by a wound expert fell.

Distinguishing features of possible diagnostic value of wounds with osteomyelitis or an anal fistula were the presence of air bubbles on the wound surface, the persistent presence of gorges in the wound bed, and the resistance to stable closure when treated with MPPT, examples in Supplementary material S9.

### 3.5 Health economics

Guest et al. (51) determined clinical outcome, mean cost, and resource use for the first 12 months after presentation to the NHS of acute pressure ulcers treated with standard care. Their treatment groups correspond to the acute wounds in the present study, allowing direct comparison (50). However, the patient population in Guest et al. only included a low percentage of persons with SCI, meaning they generally had an intact immune system and would likely respond better to treatment. Table 3 compares the percentage of wounds that closed, mean time to closure for the ones that achieved closure, and mean cost of treatment for each wound grade of acute wounds treated without and with antimicrobials as standard care to acute wounds treated with MPPT. In all groups, the use of MPPT resulted in higher healing rates, shorter time to closure, and lower costs, with per-wound cost savings ranging from 59 to 94% in the first year and 100% in subsequent years as the wounds treated with MPPT had closed (Supplementary material S6).

**Table 3:** Comparison of study outcome of acute wounds to Guest et al. (51).

Acute wounds	Guest et al. (51) Acute wounds – Standard of care								Study acute wounds MPPT		
	No antimicrobials				Antimicrobials						
Grade	% of cohort	Closure	Time months	Cost	% of cohort	Closure	Time months	Cost	Closure	Time months	Cost
1	82%	100%	1.2	£801	18%	100%	4.0	£4,806	100%	<1	£328
2	53%	57%	4.9	£3,801	47%	0%	-	£13,084	100%	<1	£224
3	27%	23%	6.6	£5,219	73%	15%	8.2	£9,679	100%	1.8	£1,884
4	24%	0%	-	£8,226	76%	20%	7.3	£17,610	100%	1.3	£2,645

Percentage of cohort is the distribution of wounds receiving non-antimicrobial vs. antimicrobial treatment in the study by Guest et al. (51); Closure is the percentage of the group reaching closure; Time is the average number of months to closure for the ones that closed; Cost is mean costs first year (51) which in the case of MPPT also means cost to closure. Unstageable are not included as they represent a mix of grades. Adjusted to the beginning of 2022 prices (Supplementary material S1).

The most frequent type of acute wound in both Guest et al. (51) and the MPPT study is grade 3 pressure ulcers. For these, Table 4 shows the costs associated with using standard care relative to MPPT after 12 months of treatment. These were £9,679 and £5,219 for antimicrobial and non-antimicrobial standard care approaches in the first year, respectively, and 82.5% (77–85%) of these wounds remained unhealed. In contrast, with MPPT, all wounds closed the first year, and average cost was only £1,884. This facilitated potential savings per wound between 63.9 and 80.5% in the first year alone (Supplementary material S6).

**Table 4:** Mean costs of treating acute grade 3 wounds and pressure ulcers in SCI persons with MPPT compared to standard care by the NHS the first year.

Wound types	Portion of cohort	Closure rate	Time to closure (months)	Cost First year	Excess cost first year compared to MPPT	Potential first year savings with MPPT
MPPT Grade 3 with infection	100%	100%	1.6	£1,884	£0 Unhealed: 0%	-
Standard Care Grade 3 with antimicrobials*	73%	15%	8.2	£9,679	£7,795 Unhealed: 85%	80.5%
Standard Care Grade 3 no antimicrobials*	27%	23%	6.6	£5,219	£3,335 Unhealed: 77%	63.9%

*Guest et al. (51). Costs have been adjusted to the beginning of 2022 prices (Supplementary material S1).

**Figure 3:**
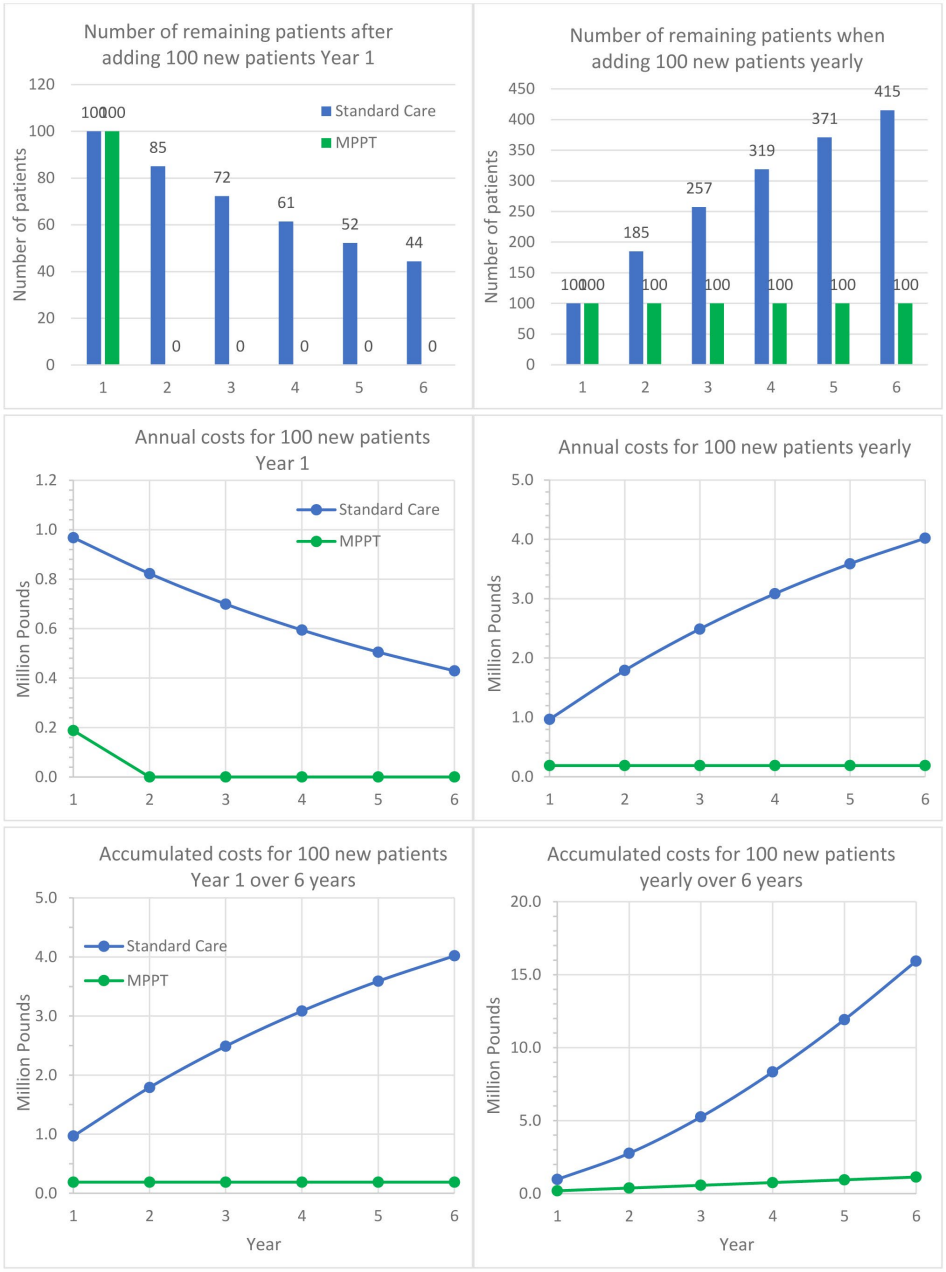
Comparison of MPPT to standard care for treating acute, infected grade 3 wounds and pressure ulcers over a 6-year period. The left column follows a cohort of 100 patients developing an ulcer in Year 1 and are being followed for 5 additional years. The graph shows the remaining unhealed patients annually, annual costs, and accumulated costs. The right column assumes 100 new patients appear every year and shows the development in unhealed patients, annual costs, and accumulated costs.

Table 4 brings to attention the low closure rate with standard care and the very long duration of treatment required for the 17% of the acute grade 3 pressure ulcers that reached closure within the first year. The implication of such numbers is that 83% of wounds that did not heal will continue to need treatment in the following years, as well. Another consequence is that over time, a grade 3 wound tends to deteriorate into a grade 4, which has a high probability of developing osteomyelitis (Supplementary material S4); this can happen in as little as 7 weeks and is associated with a high risk of death and irreversible morbidity. In contrast, the table shows that MPPT achieved a 100% closure rate with an average time to closure of 1.6 months. The table also compares the total cost of MPPT until closure to the cost of standard care for the first year only. The costs generated with standard care will, of course, continue to accumulate in the following years, while the MPPT costs on average stop after 1.6 months. The failure of 83% of acute grade 3 wounds treated with standard care to close within 1 year from first presentation to the NHS will necessarily affect costs in the following years, as shown in Figure 3, which illustrates the implications of the differences in closure rates and costs of treatment.

**Figure 4:**
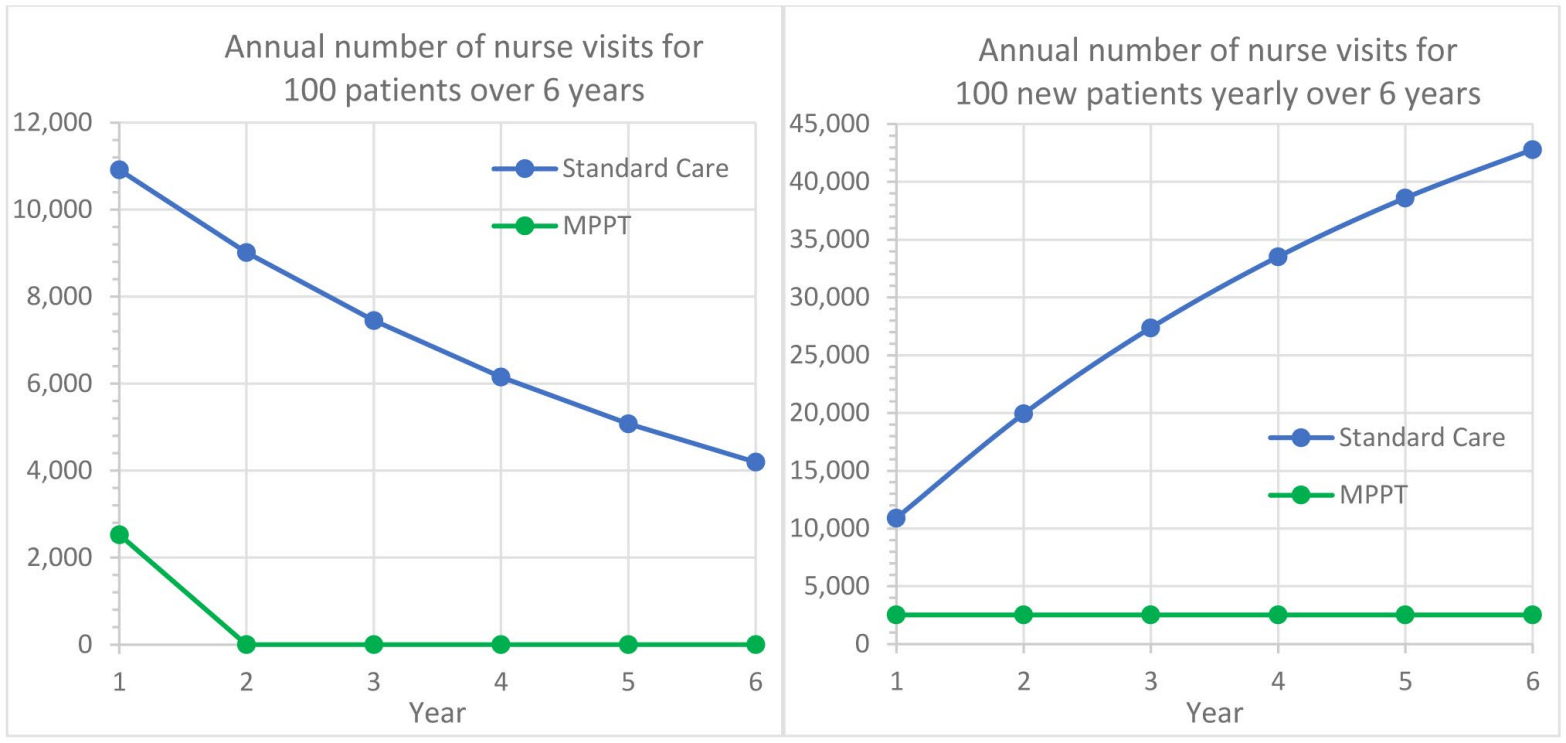
Comparison of MPPT to standard care in terms of annual number of nurse visits or wound evaluations used for the treatment of acute grade 3 pressure ulcers from Year 1 to Year 6. The left graph follows a cohort of 100 new grade 3 pressure ulcers developed in Year 1 and their annual need for dressing changes followed for an additional period of 5 years. The right graph assumes that 100 new grade 3 pressure ulcers develop annually and illustrates the total annual need for nurse visits.

Guest et al. (51) also found that an acute grade 3 pressure ulcer on average required 109.14 nurse visits the first year, whereas MPPT required an average of 25.3 remote wound expert evaluations (virtual visits) to reach closure, i.e., a reduction of 76.8%. With 82.5% of standard care patients carrying over into the following year, Figure 4 shows, in a similar manner to Figure 3, the implications over time. For example, after the first year and an additional 5 years of treatment of initially 100 patients with standard care, 42,805 dressing changes will have been performed, and 40 patients will remain unhealed. In comparison, MPPT will require 94% fewer changes (2,530), and all wounds will have healed (Supplementary material S6).

All chronic wounds were non-healing and were, before MPPT, receiving treatment with standard care. After changing to MPPT, all wounds reached closure, i.e., the improvement rate was 100% (Table 1; Figure 2A). The median and mean costs of MPPT treatment to reach closure were £3,584 and £3,999, respectively, for chronic grade 3; and £7,966 and £7,255, respectively, for chronic grade 4 wounds. The median and average number of days to closure were 75 and 98 days, respectively, for grade 3; and 183 and 188 days, respectively, for grade 4. The mean daily costs of treatment were, therefore, £40.81 and £38.64 for chronic grade 3 and 4 pressure ulcers, respectively. Bennett et al. (52) determined the mean daily cost of treatment with standard care for grade 3 and 4 pressure ulcers with critical colonization, similar to the MPPT-chronic wound group, to be £82.76 in 2022 prices (Supplementary material S1). Comparing the costs of treatment in the present study to Bennett et al. (52), we found average daily savings of 51 and 53%, respectively (Supplementary material S6). The overall economic benefit will necessarily be substantially greater as the wounds reach closure with MPPT, whereby the treatment cost is stopped.

Wounds acting as draining fistulas were, at enrolment, being treated with standard care. It took approximately 2.5 months to stabilize the wounds (Figure 2B), during which time the monthly use of MPPT decreased by 63%. The total costs of MPPT treatment during its first year of use, which includes the initial period of bringing the wound under control, was £24,054, and the cost of its 12th month of use was £1,230, which represents the monthly cost for continued treatment after stabilization of the fistula. In comparison, Bennett et al. (52) calculated the annual costs of wounds with osteomyelitis, i.e., draining fistulas, at £95,400 and the monthly costs at £7,950. Therefore, during the first year of use, the cost savings with MPPT were 74.8% relative to standard care, and during the 12th month of treatment with MPPT, which represents monthly maintenance costs, MPPT provided savings of 84.5%. Furthermore, the use of MPPT will delay follow-on physical conditions and be associated with improvements in the quality of life as patients are not required to remain on bed rest, and treatment can be delivered by telemedicine, providing independence and freedom to exercise. Feedback from patients indicated that they were feeling better, which is consistent with the reduced level of soft tissue infection and toxemia (Supplementary material S6).

### 3.6 Telemedicine

The telemedicine approach was well received and facilitated self-care and independence. The level of support required rapidly reduced as participants became familiar with the process.

### 3.7 Equality

MPPT is suitable for telemedicine, and this allows the provision of equal access to treatment with guidance by wound experts independently of location, including in remote areas. This can either be directly to patients or their family or carers, who are assisting, but it can also provide support to healthcare professionals, who are non-wound experts, meaning that equal quality of treatment can be provided independently of location.

### 3.8 Environmental sustainability

Each dressing change is associated with resource use, e.g., nurse visits, dressing materials, antimicrobials, surfactants, and transport (Table 5). In addition to a strong reduction in the requirement for nurse visits, as shown above, MPPT is not an antimicrobial, and the use of antimicrobials and surfactants in the procedures surrounding its use is specifically advised against. The use of antimicrobials and surfactants was therefore avoided altogether with MPPT. MPPT only contains natural, non-toxic, directly bio-recyclable ingredients, and occlusion of the wound is contraindicated. The only polymer associated with its use is a tape adhesive. Consequently, the use of plastics and other synthetic polymers and silicones was reduced by >99%. CO_2_ emission from transport only involved the delivery of the materials to the patient and was reduced by >99% per wound on average compared to standard care (Supplementary materials S1, S6).

**Table 5:** Comparison of the sustainability of standard care (51) vs. MPPT for treating an acute grade 3 pressure ulcer during the first 12 months.

Resources	Standard care		MPPT		Reduction with MPPT
	Units	Amount	Units	Amount	
Nurse visits	109.1		25.3		77%
Antimicrobials (antibiotics and antiseptics)					
Dressings	211.3	1.1 kg	25.3	0	100%
Prescriptions antibiotics	2.4	36.0 g	0	0	100%
Topical treatments	8.44	0.01 kg	0	0	100%
Plastics, and other synthetic polymers and silicones					
Dressings	211.3	21.1 kg	25.3	0.03	>99%
Bandages	16.3	0.2 kg	0	0	100%
CO_2_-emissions					
Transport miles	1309.7	266.1 kg	12	3.1 kg	>99%

Standard care dressings are assumed to contain an average of 5 g of antimicrobials and 60 g of plastics, silicones, and other synthetic polymers per dressing. Prescribed antibiotics are assumed to include a 10-day course of 3 × 500 mg daily. Bandages primarily contain compression bandaging composed of cotton, viscose, polyethylene terephthalate [PET], cotton-Lycra, and PET-Lycra; they are assumed to contain 10 g plastics on average. Topical treatments typically include antiseptics (e.g., iodine and silver), hydrogels, drugs (e.g., phenytoin), and antibiotics (e.g., silver sulfadiazine); they are assumed to contain 1 g antimicrobials each on average. MPPT and its use do not involve any antibiotics, antiseptics, drugs, plastics, or other synthetic polymers and silicones, i.e., their use is contraindicated. For transport, it is assumed that standard care will involve transport by a standard car (127 g CO_2_ per km). MPPT will involve the delivery of the treatment by van (160 g CO_2_ per km). For a grade 3 pressure ulcer, the patient will normally only receive one delivery of MPPT. An average distance of 6 miles is assumed between the starting point and the patient.

## 4 Discussion

The study reached its primary endpoints by MPPT treatment, resulting in stable closure of all acute and chronic wounds and pressure ulcers independently of grade and prior treatment, and in the control of soft tissue infection in wounds acting as a fistula draining from an underlying, primary focus of infection, e.g., osteomyelitis. MPPT was consistently able to remove antimicrobial-resistant infections. No adverse events were observed, including following daily application for over 2 years directly onto muscle and exposed bone. As a result of reaching closure or controlling the soft tissue infection, substantial per-wound cost savings between 51 and 94%, depending on wound grade and age, as well as the reduction in demand for nurse capacity, e.g., 76.8% for acute grade 3 ulcers the first year alone, were achieved. The use of MPPT meant eradicating all use of antimicrobials; almost completely abandoning the use of plastics and other synthetic polymers and silicones, and chemicals; and substantially reducing CO_2_ emissions due to highly reduced requirements for transport. The economic and environmental sustainability profiles of MPPT were, therefore, very beneficial compared to standard care (Supplementary material S6).

Pressure ulcers are a common wound type that responds poorly to existing treatment approaches (22). The majority of wounds in the study were pressure ulcers, and MPPT was consistently able to heal these to closure in an immunocompromised patient population. The findings are in agreement with previous studies using MPPT (23, 25, 26, 29, 30).

Smith (59, 60) from the British patient organization SIA (Spinal Injuries Association) conducted an online survey to establish the experiences SCI persons had had with their use of MPPT. The survey had 41 respondents. All wounds (*n* = 33), primarily pelvic pressure ulcers, had reached full closure. The median duration of MPPT use and time to closure were 3 and 4 weeks for acute wounds (<6 weeks old) and 8 and 10 weeks for chronic wounds, respectively. On draining fistulas (*n* = 9), MPPT was used by the respondents to reduce wound size, remove soft tissue infection, avoid sepsis, reduce autonomic dysreflexia, improve overall health and wellbeing, and avoid bed rest while waiting for surgery. Comments on MPPT were 84% highly positive, 11% positive, and 0% negative; 5% were uncertain whether the achieved closure was due to MPPT or the change in treatment regime. No adverse events were reported. The user feedback, therefore, closely resembled the findings of this study and provided independent confirmation of the findings of the present study.

The present study was designed to be performed in community care with participants, their families, or carers being responsible for the daily hands-on treatment. Furthermore, participants had to actively request access to MPPT, be willing to take responsibility for their own care, and provide daily pictures. There will always be a subgroup who live alone and are unable to treat the wound themselves due to its location. This subgroup will require daily nursing support. First, the involvement of an experienced nurse will mean that the usual learning curve for new users can be avoided, which means that the start of treatment with MPPT will be more efficient. Second, the nurse will replace the use of remote support and, as the pricing of this corresponds to the visit costs of a senior nurse, the implications on costs will be limited. Furthermore, participants seeking inclusion in the study would likely be individuals who had poor prior experiences with the use of standard care, which, on one hand, could lead to better compliance but, on the other hand, could also indicate that they were under-average healers. While these factors could result in a selection bias, it is difficult to determine whether it would affect the outcome and to what extent. The study by Smith (59) represents a broader population and it led to the same outcome, including quicker healing, which suggests that the outcome determined in this study is reasonably representative of a general SCI population.

The study was subject to several limitations. First, due to the poor response of SCI persons to current wound products, the study had to be single-arm as required by ICH E10 guidance (46). Second, pressure ulcers in SCI persons are an orphan indication, which impact the recruitment rate and number of wounds that realistically can be included. The outcome in terms of wound healing was consistent across the study and confirmed independently by Smith (59, 60). However, due to the single-arm design, in-study comparators could not be included, and it was consequently necessary to compare to published data on pressure ulcers (51, 52). However, these data did not only include SCI persons but rather a broad range of conditions that had resulted in the development of pressure ulcers. The cost comparisons will, therefore, have limitations, but given that pressure ulcers account for 25% of the total healthcare costs of SCI (39) and that the outcome of treatment changes from non-closure for many wounds and possibly developing osteomyelitis to completely resolving the condition by closing the wound, the level of costs savings will necessarily be very substantial. Furthermore, cost savings were only calculated for the first year and did not include follow-on consequences, e.g., hospitalizations with sepsis, and each surgery for osteomyelitis readily cost £75,000 to £150,000 depending upon complications, and repeated surgery is often necessary. Other cost categories, such as social care implications and lost productivity, were similarly not included. Calculated cost savings are, therefore, indicative of the benefits that can be achieved and very likely underestimated.

Wounds and pressure ulcers clearly pose a substantial risk to SCI persons, with the level of risk depending on grade and age of the wound. In grade 1–3 wounds, not having penetrated the basal membrane of *tela subcutanea*, the main risk is further deterioration of the wound. MPPT is able to close these wounds, thereby removing the risk. In grade 4 wounds, the risk of developing osteomyelitis via contiguous spread increases rapidly as the main anatomical and immunological barriers have been breached, allowing infection to spread relatively unhindered in the tissue. MPPT is able to close these wounds as long as osteomyelitis is not present, thereby removing the risk. Once the infection has spread to the bone, the primary source of infection is no longer the wound but the osteomyelitis. This converts the wound into a draining fistula, secondary to the osteomyelitis. MPPT can control this consequential infection in the soft tissue and reduce the risk of sepsis originating in the soft tissue, but the risk of sepsis originating in the bone remains. Its resolution requires surgery for the primary causative condition, i.e., osteomyelitis (Supplementary material S4). If the surgery is not performed successfully, the osteomyelitis will, at varying speeds, continue to spread over time and affect an increasing part of the bone, thereby constituting an increased risk of sepsis. As the infection progresses, the infected area of the bone enlarges, and part of the debris is generated increasingly further from the draining canal, i.e., the wound. Instead of increasing the distance to travel, the debris may carve new fistulas that resemble wounds. Each will represent an increased risk of sepsis originating in the soft tissue and will need MPPT to control this. There is consequently a high level of urgency in treating any new wound to prevent the development of osteomyelitis as this is associated with poor prognosis (44, 45).

MPPT acts by removing microbial toxins and disrupting biofilm shields, i.e., it interferes with the weaponry of microbes and leaves them vulnerable, and this, in turn, leads to a larger proportion of immune cells surviving and being functional. These two parallel actions enable the immune system to remove the infection, control tissue regeneration, e.g., hypertrophic scarring is not seen with MPPT, and achieve stable wound closure. In an immunocompetent person with a non-healing wound, short-term use of MPPT is usually sufficient to enable the body to regain control and once achieved, it can itself control the wound environment and progress the wound to closure. In contrast, in an immunocompromised person, the reduced presence or impaired efficacy of immune cells means that the immune system will have difficulty achieving and retaining full control and need ongoing support. Consequently, the duration of use of MPPT is determined by the immune status of the person, the virulence and chronicity of the infection, and the use of antimicrobials and immunosuppressant medication. Duration of application, therefore, ranges from daily application for 1–5 days to assist in the healing of an acute wound to daily application until closure in an immunocompromised person.

Patients are frequently placed on bed rest to support healing, but this is associated with negative health effects (61–64) (Supplementary material S6). Bed rest is not required with MPPT, allowing the individuals to retain an active life.

Ease of use and the fact that rapid progress could be seen when using MPPT were strongly motivating and permitted the management of MPPT treatment via telemedicine. As highlighted in the study by Smith (59) this approach was well-received by MPPT users. This combination of self-care and no requirement for bed rest supported independence as participants were not bed-bound and could plan their own day and assume responsibility for their treatment and progress. This contributed to social sustainability as the participants were able to hold a job or livelihood, follow an education, and engage socially outside the home. The telemedicine approach also allows access to an equal level of expert support independently of location, e.g., remote areas, and it frees up nursing resources and contributes to net-zero due to the reduced need for transport CO_2_ emissions. The telemedicine approach is also suitable for other wound types and can furthermore support community nurses, who may not have been specifically trained in the treatment of wounds but are required to evaluate and treat wounds. Using digital approaches, the process of taking and sending pictures can be streamlined to further simplify the process (53, 65, 66).

The US FDA emphasized that the development of new and effective wound treatments and their adoption into clinical practice is complicated by difficulties in conducting clinical trials (1). The present study was performed in an orphan indication, with the wounds mainly being treated in community care. The US FDA recommends wound closure as the primary endpoint, which was used in the present trial. Due to ethical considerations, the trial was single-arm, and it was chosen to use a non-interventional approach to increase the probability that the outcome would be representative of clinical implementation. Independent confirmation of study findings by real-world data is rarely achieved; however, the survey by Smith (59) provided such data on the use of MPPT by this specific patient population and with the same primary endpoint, i.e., wound closure. As shown in Table 6, the closure rates were the same in both studies, but the time to wound closure was quicker based on real-world data compared to the clinical study, possibly because the wound experts were more cautious about stopping treatment early than users. In relation to draining fistulas, respondents used MPPT to reduce wound size, remove soft tissue infection, avoid sepsis, reduce autonomic dysreflexia, improve overall health and wellbeing, and avoid bed rest while waiting for surgery. These are the same benefits as observed in this study. The comments on MPPT were highly positive, emphasizing that the speed of healing, no need for bed rest, and the telemedicine approach were well received. Finally, no adverse events were reported in the survey, which is the same as seen in the present study. From a clinical trial viewpoint, the consistency between the two studies highlights the benefits of non-interventional studies as a clinical trial design to provide clinically relevant data (49, 67, 68). In relation to MPPT, they affirm the findings of this study with MPPT in relation to efficacy, safety, health economic and social benefits.

**Table 6:** Comparison of outcome of the survey in the SCI community on user experiences with MPPT (59) to the current study.

	Smith (59, 60)		MPPT study	
	Closure rate	Time to closure (median)	Closure rate	Time to closure (median)
Acute	100%	4 weeks	100%	6 weeks
Chronic	100%	10 weeks	100%	14.4 weeks

The number of acute and chronic wounds and pressure ulcers included were 33 and 32 in the studies, respectively. For the MPPT study, Grade 1 pressure ulcers were not included as they do not involve skin breakage, and the number of days to closure was therefore not entered.

An increasingly important consideration when choosing between treatment approaches is their sustainability profile, i.e., the wider impact of using the treatment, which must be evaluated in relation to its health benefits. Antimicrobials are the most commonly used approach to treating infected wounds despite their proven limited clinical efficacy (20–22). However, both antibiotics and, what is less well-known, antiseptics cause AMR (8, 9, 12, 13, 15). Furthermore, new studies show that they damage the gut microbiome, resulting in long-term health problems such as obesity, cancer, and mental health issues (69–72). About half of the antibiotics and antiseptics that are used escape sewage treatment plants and end up in nature, damaging natural soil and aquatic microbiomes by reinforcing the resistant strains to become dominant (73). This severely impacts the dynamics of the natural eco-systems and causes, among others, deforestation and desertification (74–76). Moreover, it is the microbes in the soil and oceans that are responsible for removing more than 50% of the greenhouse gasses from the atmosphere, and damaging these systems inevitably impacts the ability of the Earth to control levels of atmospheric greenhouse gasses and thereby control the climate (77). In addition to AMR, they can also cause environmental toxicity, e.g., nano-silver is toxic to nitrogen-fixating organisms, which are essential for plant life and key in the fight against climate change (16, 78). Finally, it is worth mentioning that many types of chemicals contribute to AMR, including surfactants (79), which are used extensively in standard care wound cleansers and soaps. As they are chemically very stable, they remain in nature unchanged for a very long time. However, there are grounds for optimism because some findings suggest that these systems will return to a more natural state if the release of antimicrobials and similar chemicals is stopped (13).

MPPT includes only natural ingredients, which are readily biologically recyclable. It contains no antimicrobials, surfactants, or other chemicals. Moreover, the use of any of these substances, e.g., in wound cleansing solutions, as well as dressings with plastics, silicones, and similar, is contraindicated in wounds treated with MPPT. Only tap water and pure cotton can be used and MPPT will therefore essentially eliminate the use of antimicrobials, surfactants, plastics, other synthetic polymers and silicones, and chemicals in wound care.

In relation to SCI, future research will focus on the use of MPPT to remove soft tissue infection before surgery to allow surgery to be performed in non-infected tissue and on long-term use of MPPT in persons with inoperable osteomyelitis to determine quality of life benefits and a potential impact on the osteomyelitis itself, as preliminary data indicate bone improvement following long-term use.

An increasing body of data demonstrates that commensal bacteria are essential for skin health and wound healing and that antimicrobials delay wound healing (7). MPPT is the first approach to use interactions with the wound microbiome to treat wound infections and support tissue regeneration. People with SCI have difficulty fighting wound infections due to their immunosuppressed state and respond very poorly to standard wound treatment approaches. This study, together with two independent studies (29, 59, 60), has now confirmed, in this population, that an approach acting via the wound microbiome to support the immune system is effective in healing to closure their wounds and pressure ulcers and in controlling soft tissue infection caused by an underlying primary focus of infection. Together with prior studies of MPPT, these findings, therefore, confirm the safety and effectiveness of this approach across wound types and immune status and, importantly, confirm that it is effective on antimicrobial-resistant infections. Hopefully, this means that the classification of wounds as a field of unmet medical need (1) may belong to the past.

## Data availability statement

The raw data supporting the conclusions of this article will be made available by the authors, without undue reservation.

## Ethics statement

Ethical approval was not required for the study involving humans in accordance with the local legislation and institutional requirements. Written informed consent to participate in this study was not required from the participants or the participants’ legal guardians/next of kin in accordance with the national legislation and the institutional requirements.

## Author contributions

JS-D: Writing – original draft. MB: Writing – review & editing. SB: Writing – review & editing. DS: Writing – review & editing. FS-D: Writing – original draft.
