# Supplementary material for: Stable closure of acute and chronic wounds and pressure ulcers and control of draining fistulas from osteomyelitis in persons with spinal cord injuries: non-interventional study of MPPT passive immunotherapy delivered via telemedicine in community care
Source: Front Med (Lausanne). 2024 Jan 5;10:1279100. doi: 10.3389/fmed.2023.1279100 (PMC10797031; doi:10.3389/fmed.2023.1279100)
Supplement: Supplementary file 1 [file Data_Sheet_1.docx]

# S1: The costs of wounds

The NHS “THIN” database contains over 11 million patient records from 550 general practices in the UK and has been shown to be representative of the UK (1). In a series of studies, Guest and colleagues used this database to determine the burden of wounds in primary care on the UK healthcare system, NHS. The purpose of this document is to use a comprehensive set of published data to estimate the number of wound and their burden on primary care in the UK National Health Service (NHS) in 2022 and to project the development in numbers and costs until 2035. Year 2022 is used as reference point in the analysis as inflation was relatively low and stable between 2012 and 2022, whereas it increased significantly in 2022 and demonstrated significant fluctuations. Therefore, by using 2022 as the reference point, the calculations are more reliable.

## Estimation method and nursing costs

Guest et al. (1) determined the costs of wound care for 2012/2013 to be £5.3 billion for UK primary care. The calculations used a method based on “excess costs”, where the cost of care for 1000 patients with wounds was determined and from this the cost of care for 1000 patients with matched co-morbidities but without a wound was subtracted. This led to an average cost per wound of £2,394 (2013 prices). However, in a later series of studies, Guest et al. (2–5) determined the cost of care for the first 12 month after presentation to the NHS for surgical wounds, pressure ulcers, diabetic foot ulcers and venous leg ulcers and found an average cost of £7,786 (2015 prices). For example, Guest et al. (1,6) found the average cost of a surgical wound to be £3,881 and of a pressure ulcer to be £3,463 per wound, respectively, i.e. the surgical wound was more expensive than a pressure ulcer, but the detailed studies (2–5) found just the opposite, i.e. £7,344 and £8,377, respectively.

|  | **Guest et al.** (1,6) | | | **Guest et al.** (2–5) | | |
| --- | --- | --- | --- | --- | --- | --- |
|  | Nursing costs | Cost of wound | % Nursing | Nursing costs | Cost of wound | % Nursing |
| **Surgical** | £307 | £3,880 | 8% | £4,440 | £7,344 | 60% |
| **PU** | £628 | £3,464 | 18% | £7,210 | £8,377 | 83% |
| **DFU** | £600 | £,3281 | 14% | £4,045 | £7,856 | 52% |
| **VLU** | 3701 | £3,388 | 21% | £6,163 | £7,568 | 82% |

Table 1. Comparison of the costs of nursing included in the cost of wound calculation for Guest et. al (1,6) and Guest et al. (2–5). Surgical: surgical wounds; PU: pressure ulcers; DFU: diabetic foot ulcers; VLU: venous leg ulcers.

Table 1 compares the contribution of nursing costs, i.e. community nurses, district nurses and general practice nurses, to overall costs for each of the two methods for determining costs of wounds. For each method, the latter column (% Nursing) shows the percentage by which nursing costs contribute to overall costs. The differences between Guest et al. (1,6) and Guest et al. (2–5) are substantial. The comparison indicates that the original method of basing the estimates on excess costs led to the omission of substantial nursing costs despite these usually being the main contributor to the cost of wound care, e.g. Urwin et al. (7). Due to this, it was decided to use the number of wounds from Guest et al. (1) and costing data from Guest et al. (2–5). This required first normalising all data to 2012 and subsequently projecting the development to 2035. At the end, validity checks were performed and are shown at the bottom of this document.

## Distribution of wound types and direct costs of care

|  | **Wound types** | **Number of wounds** | | **Direct Costs of Care (£)** | | **Mean cost per wound** | |
| --- | --- | --- | --- | --- | --- | --- | --- |
|  | Surgical | 253,307 |  | £983,000,000 |  | 3,881 |  |
|  | Pressure ulcer | 153,317 |  | £531,000,000 |  | 3,463 |  |
|  | Diabetic foot | 168,871 |  | £554,000,000 |  | 3,281 |  |
|  | Leg ulcers (all types) | 731,035 |  | £ 1,938,000,000 |  | 2,651 |  |
|  | Abscess (Other) | 159,983 |  | 290,000,000 |  | 1,813 |  |
|  | Burn (Other) | 86,658 |  | 90,000,000 |  | 1,039 |  |
|  | Open wound (Other) | 239,957 |  | 410,000,000 |  | 1,709 |  |
|  | Trauma (Other) | 157,761 |  | 159,000,000 |  | 1,008 |  |
|  | Unspecified (Other) | 271,083 |  | 364,000,000 |  | 1,343 |  |
|  | **All wounds** | **2,221,972** |  | **£5,319,000,000** |  | **£2,394** |  |

Table 2. Guest et al. (1,6) determined the number, distribution, and average cost of main wound types requiring treatment by the NHS in community care and the direct costs of caring for the wounds. This was based on the analysis of 1000 randomly selected records of patients with wounds and 1000 records of patients without wounds and with matched comorbidities. Costs were determined as excess cost in the group with a wound. 2013/2014 prices used.

Guest et al. (1,6) determined (Table 2) that 2.2 million wounds required treatment by the NHS in 2012/2013 with costs of care of £5.3 billion in 2013/2014 prices, resulting in an average cost per wound of £2,394.

## Costs of wounds the first 12 months following presentation to the NHS

|  | **Patient distribution** | | | **% healed within first 12 months** | | | **Time to closure (months)*** | | | **Mean cost per wound (12 months)** | | |
| --- | --- | --- | --- | --- | --- | --- | --- | --- | --- | --- | --- | --- |
|  | **No** | **AM** | **ABX** | **No** | **AM** | **ABX** | **No** | **AM** | **ABX** | **No** | **AM** | **ABX** |
| **SW** | 16% | 18% | 66% | 92% | 83% | 85% | 1.9 | 5.8 | 6.5 | £2,001 | £6,966 | £8,742 |
| **PU** | 32% | 15% | 53% | 40% | 19% | 14% | 3.7 | 5.3 | 7.7 | £4,662 | £7,368 | £10,906 |
| **DFU** | 25% | 41% | 34% | 70% | 32% | 16% | 2.3 | 6.2 | 6.1 | £2,604 | £7,967 | £11,585 |
| **VLU** | 53% | 6% | 40% | 75% | 45% | 25% | 2.2 | 3.9 | 4.9 | £3,328 | £10,777 | £12,893 |

Table 3. Data from Guest et al. (2–5) used for calculating burden of wound care in 2015/2016 prices. **No**: No treatment with antibiotics or antimicrobial dressings; **AM**: Treatment with antimicrobial dressings but no systemic antibiotics; **ABX**: Treatment with systemic antibiotics with or without an antimicrobial dressing. **PU**: pressure ulcer; **DFU**: diabetic foot ulcer; **VLU**: venous leg ulcer; **SW**: surgical wound. * time to closure for the wounds that healed within the first year. The costs of the unhealed wounds continue into the following year, but these wounds are not included in the calculations.

Guest et al. (2–5) determined the overall costs of four wound types for the first 12 months after first presentation to the NHS (Table 3). This in-depth analysis included many more patient records per wound type compared to the 2015-study, full nursing costs as well as all other directly wound related activities, e.g. scans, surgery, hospitalisation etc. However, any conditions arising later, as a consequence of the wound, were not included. As major surgical interventions are usually associated with wounds that have deteriorated over time, these would be unlikely to have been required the first year, and the approach taken is therefore likely to underestimate the costs. Costs of social care are not included either, and would also be increasing in subsequent years matching the deterioration of the wound.

In Table 3, the wounds are divided into those that did not receive an antimicrobial, those that only received a topical antimicrobial on the wound and those that received systemic antibiotics with and without topical antimicrobials. Patient records do not define what is considered an infected wound, and, in the analysis, it is therefore assumed that a wound receiving an antimicrobial is infected. As can be seen, healing rates, time to closure and costs vary substantially between groups. The average cost per wound is £7,786 across the four wound types. This estimate includes both healed and unhealed wounds in the first 12-month period after presentation.

The findings show a poorer outcome for wounds receiving antimicrobials. This may be due to the presence of infection, since both NICE (8) and the FDA (9,10) have independently concluded, that there are no studies demonstrating the efficacy of antimicrobials for treating wound infections. However, it is also known, that antiseptics are cytotoxic and that antimicrobials will favour resistant bacterial strains, which typically are more virulent [S2]. An additional factor could therefore be, that the use of antimicrobials directly impairs wound healing. As will be shown further down, an annual increase in the number of wounds of 11.1% has been observed and this may, at least in part, be attributed to the global increase in antimicrobial resistance. The low healing rates could therefore be due to the presence of infection and the use of treatments impeding healing.

## Inflation

| 2012 | 2013 | 2014 | 2015 | 2016 | 2017 |
| --- | --- | --- | --- | --- | --- |
| 5.228 | 5.300 | 5.373 | 5.448 | 5.523 | 5.600 |

Table 4. Estimation of annual average inflation rate (1.385%) based on Guest et al. (2020), page 8.

Costs in Guest et al. (1,6) were in 2013/2014 prices, meaning that the 2012/2013 estimate of costs actually was in 2013/2014 prices; Guest et al. (2–5) used 2015/2016 prices. To standardise all costs to 2012/2013 prices to allow direct comparison, it was possible to use data from Guest et al. (11). This publication states that the costs of wound care in 2012/2013, i.e. £5.3 bn in 2013/2014 prices, would have corresponded to £5.6 in 2017/2018 prices. Using this information, the annual inflation rate was determined at 1.385%, as shown in Table 4.

## Costs of wounds 2012

Using the cost estimates in Guest et al. (2–5) and the estimates of number of wounds in 2012 in Guest et al. (1), the costs of wounds in 2012 was determined in the following manner:

1. Guest et al. (2–5) calculated the overall costs for the first year after presentation to the NHS for four wound types. These amounts include both wounds that healed during the 12-month period and wounds that remained unhealed and therefore represent a balanced figure across one year. The costs were strictly limited to the first year of existence of the wound.

| **2011** | | |  |  | **2012** | |  |  |  |  |  |  |  |  |  |  | **2013** | |  |  |
| --- | --- | --- | --- | --- | --- | --- | --- | --- | --- | --- | --- | --- | --- | --- | --- | --- | --- | --- | --- | --- |
| **8** | **9** | **10** | **11** | **12** | **1** | **2** | **3** | **4** | **5** | **6** | **7** | **8** | **9** | **10** | **11** | **12** | **1** | **2** | **3** | **4** |
|  |  |  |  |  | 23 |  |  |  |  |  |  |  |  |  |  |  |  |  |  |  |
|  |  |  |  |  |  | 23 |  |  |  |  |  |  |  |  |  |  |  |  |  |  |
|  |  |  |  |  |  |  | 23 |  |  |  |  |  |  |  |  |  |  |  |  |  |
|  |  |  |  |  |  |  |  | 23 |  |  |  |  |  |  |  |  |  |  |  |  |
|  |  |  |  |  |  |  |  |  | 23 |  |  |  |  |  |  |  |  |  |  |  |
|  |  |  |  |  |  |  |  |  |  | 23 |  |  |  |  |  |  |  |  |  |  |
|  |  |  |  |  |  |  |  |  |  |  | 23 |  |  |  |  |  |  |  |  |  |
|  |  |  |  |  |  |  |  |  |  |  |  | 23 |  |  |  |  |  |  |  |  |
|  |  |  |  |  | 23 |  |  |  |  |  |  |  | 23 |  |  |  |  |  |  |  |
|  |  |  |  |  | 23 |  |  |  |  |  |  |  |  | 23 |  |  |  |  |  |  |
|  |  |  |  |  | 23 |  |  |  |  |  |  |  |  |  | 23 |  |  |  |  |  |
|  |  |  |  |  | 23 |  |  |  |  |  |  |  |  |  |  | 23 |  |  |  |  |
|  |  |  |  |  | 77 | 77 | 77 | 77 | 77 | 77 | 77 | 77 | 77 | 77 | 77 | 77 |  |  |  |  |

Figure 1. Method used to correct wound numbers, using pressure ulcers as example. Guest et al. (1) counted all pressure ulcers in 2012 and Guest et al. (4) estimated the number of healing pressure ulcers within the first 12 months (23.1%). For healing pressure ulcers, some would have started in 2011 and healed in 2012 and some started in 2012 and healed in 2013. However, if both are included in the calculations and the “average cost per wound” figure represents the first 12 months, the amount of healing wounds will be included twice in the calculation. To calculate the correction factor, it was therefore assumed that 100 new PUs developed each month and these were divided into healed and unhealed, and their distribution was plotted as shown above. Using this approach, it was found that 2012 included 1293 PUs, but the true number is 1200. Therefore, a correction factor of 1200/1293=0.93 was calculated. This was next multiplied by the number of PUs determined by Guest et al. (1), i.e. 153,317, to reach the final number of 142,585, which was used to calculate the cost of pressure ulcers. Figure colours: Yellow: wounds expected to heal; Brown: wounds remaining unhealed during the year; Red: Healing wounds from the previous year that should not be included in the calculations.

| **Treat**  **ment** | **%** | **Cost per**  **Wound** | **Proportionate**  **cost** | **Average cost**  **per wound** | **New Wounds in 2012** | **Total Costs** |
| --- | --- | --- | --- | --- | --- | --- |
|  | | |  |  |  |  |
| **Surgical** | |  |  | £7,344 | 197,579 (0.78) | £1,450,976,135 |
| **No** | 16 | £2,001 | £320 |  |  |  |
| **AM** | 18 | £6,966 | £1,254 |  |  |  |
| **ABX** | 66 | £8,742 | £5,770 |  |  |  |
|  | | |  |  |  |  |
| **PU** | |  |  | £8,377 | 142,585 (0.93) | £1,194,464,322 |
| **No** | 32 | £4,662 | £1,492 |  |  |  |
| **AM** | 15 | £7,368 | £1,105 |  |  |  |
| **ABX** | 53 | £10,906 | £5,780 |  |  |  |
|  | | |  |  |  |  |
| **DFU** | |  |  | £7,856 | 150,295 (0.89) | £1,180,722,109 |
| **No** | 25 | £2,604 | £651 |  |  |  |
| **AM** | 41 | £7,967 | £3,266 |  |  |  |
| **ABX** | 34 | £11,585 | £3,939 |  |  |  |
|  | | |  |  |  |  |
| **VLU** | |  |  | £7,568 | 621,380 (0.85) | £4,702,390,679 |
| **No** | 53 | £3,328 | £1,764 |  |  |  |
| **AM** | 6 | £10,777 | £647 |  |  |  |
| **ABX** | 40 | £12,893 | £5,157 |  |  |  |
|  | | |  |  |  |  |
| **Other*** | |  |  | £7,344 | 787,280 (0.86) | £5,781,596,254 |
| **No** | 16 | £2,001 | £320 |  |  |  |
| **AM** | 18 | £,6966 | £1,254 |  |  |  |
| **ABX** | 66 | £,8742 | £5,770 |  |  |  |
|  |  |  |  |  |  |  |
| Total number and cost of wounds in 2012 in 2015 prices | | | | | 1,899,119 | £14,310,149,499 |
| 2015 prices calculated to 2012 value using 1.385 % annual inflation factor (Table 4): | | | | | | |
| **Average cost, total number and cost of new wounds in 2012 in 2012 prices** | | | | **£7,231** | **1,899,119** | **£13,731,660,403** |

Table 5. Average cost for each wound type determined by multiplying patient distribution data with mean cost for each wound type (Table 2); this was subsequently multiplied by prevalence data for each wound type from Guest et al. (1) (Table 2), and corrected for double counting (factor in parenthesis) as explained above and in Fig 1. The costs of wounds were in 2015/2016 prices and were converted to 2012 prices using the inflation rate calculated in Table 4. The final row is therefore number of wounds and their total costs in 2012. *For wound types not covered in Guest et al. (2–5) papers, the data on surgical wounds were used as this was the cheapest and the wounds do not involve an underlying condition; however, given the high healing rate of surgical wounds and the varied composition of this group (see Table 2), this will likely result in an overestimation of healing rates and underestimation of costs. The correction factor was the average of the factors for the other wound types.

1. Guest et al. (1) provided a break-down of the number of wounds of each wound type in 2012. These numbers include both healed and unhealed wounds, i.e. wounds that did and did not close during the year. The healed wounds will include those already present from the previous year and those having developed and healed during the year; and the unhealed wounds will include those that were present at the start of the year and remained unhealed during the entire year as well as those that developed during the year and would have healed the following year but were recorded as unhealed because they developed in the latter part of the year. This is shown in Fig. 1. When using an “average cost per wound” figure that corresponds to the first year of treatment, only new wounds from that year and wounds starting that year and continuing into the next year should be included to avoid overestimations, i.e. the healing wounds carried over from 2011 should be excluded (shown in red). Figure 1 illustrates this principle for pressure ulcers and is assuming that the development of new wounds is evenly distributed across the year. For the calculation illustrated in Figure 1, it was assumed that 100 new PUs develop every month; 76.9% of these will not heal within one year and can therefore be included in full; and 23.1% will heal in 4.7 months (calculated based on Table 2). Using this approach, a correcting factor was determined. For PUs, it was 0.93, whereas it for surgical wounds was 0.78 (surgical wounds have a higher healing rate). This wound type specific correcting factor was used to calculate the number of wounds in 2012 as shown in Table 5 for each wound type.
2. Guest et al. (2–5) (Table 3) provided data on surgical wounds, diabetic foot ulcers, venous leg ulcers and pressure ulcers and their average cost for the first year is calculated in Table 5. These estimates include both wounds that healed and remained unhealed the first year. For the remaining “other” wound types, the costs were calculated based on the costs of surgical wounds as these represent the lowest figure, thereby avoiding any potential exaggeration of costs. As correction factor for the number of wounds, an average of the known 4 types was used.
3. Using these data, the total costs of wounds in 2012 were estimated in Table 5 at £14,310,149,499. However, these costs were in 2015/2016 prices. Therefore, the final step was to use the inflation percentage (Table 4) to determine the costs in 2012/2013 prices such that all numbers represented the same year, i.e. 2012. The calculation therefore showed the total costs of wounds in **2012** to be **£13,731,660,403** with an average cost per wound of **£7,231**.

## Burden of wounds 2012 to 2035

| **UK** | **2012** | **2015** | **2020** | **2022** | **2025** | **2030** | **2035** |
| --- | --- | --- | --- | --- | --- | --- | --- |
| **Total number of Wounds**  **(million)** | 2.2 | 3.0 | 5.1 | 6.3 | 8.6 | 14.5 | 24.5 |
| **Cost of wounds**  **(billion £)** | £13.7 | £18.1 | £28.6 | £34.4 | £45.3 | £71.7 | £113.5 |
| **Number of dressing changes**  **(million)** | 233 | 319 | 538 | 662 | 906 | 1,527 | 2,573 |
| **Medical waste**  **(tonnes)** | 18,665 | 25,526 | 43,013 | 52,997 | 72,480 | 122,133 | 205,800 |
| **Antimicrobials**  **(tonnes)** | 23.2 | 31.8 | 53.5 | 66.0 | 90.2 | 152.0 | 256.2 |

Table 6. Using the distribution of wounds in 2012 (Table 2), the costs of the first 12 months of treatment for new wounds (Table 3 and 5), an inflation of 1.385% (Table 4), an annual increase of 11.1% in the number of wounds and 8.4% in the costs of wounds (11), the development in the burden of wounds on the NHS was estimated until 2035. For dressing changes, every second day for 7 months per wound was assumed; waste was calculated as 80g per dressing change and antimicrobials as 15 grams/patient with an infected wound.

Guest et al. (11) used the NHS THIN-database and the approach described in Guest et al. (1,6) to determine the number and annual costs of wounds for 2017/2018. Next, they compared these data to their findings for 2012/2013 in Guest et al. (1,6). As both studies used the same data collection method, percentage changes will be valid. They found a 70% increase in the number of wounds, corresponding to an annual growth of 11.1%, and a 48% increase in costs, corresponding to an annual growth of 8.4%. These growth rates in number of wounds are in line with the findings in Guest et al. (12), thus providing two individual estimations of the increase in number of wounds. Using these numbers and the annual inflation of 1.385% (Table 4), the annual increase in number of wounds and total costs of wounds were estimated until 2035 (Table 6). In addition, a study (13) estimated the annual number of wound dressing changes performed in UK community care to be every second day for 7 months per wound. In-house estimates had found that 80 grams of waste is produced per wound dressing change. This included gloves, pans, aprons, absorbent coverings, simple standard dressings etc, but did not include more waste heavy NPWT or more advanced dressings. To calculate the annual use of antimicrobials, it was assumed that the number of infected wounds was 69.7% (based on Guest et al. (2–5) and that each patient annually received the equivalent of 15 grams of antimicrobials; corresponding to 10 days of antibiotics with 3 tablets of 500 mg daily. As wounds in general do not respond to antibiotics, antibiotic courses are likely to be repetitive; and, as many patients with non-healing wounds are treated with antimicrobials for many months, this estimate will reflect the absolute minimum amount. Using these data, the burden of wounds was calculated until 2035. An 11.1% annual increase in the number of wounds will not continue indefinitely, but it may for a considerable number of years due to the continuous increase in antimicrobial resistance. Furthermore, as AMR complicates treatment outcomes further, it will lead to an increase in costs of treatment per wound. The cost estimates are therefore likely to present a realistic picture of what is to be expected in the foreseeable future.

For **2022**, it was found that **6.3 million wounds** will require professional treatment in community care, leading to a total cost of wounds to the NHS of **£34.4 billion**. The **662 million dressing changes** will result in at least **52,997 tonnes of waste** and **66 tonnes of antimicrobials** entering the environment as most waste is disposed of as household waste.

Figure 2. Percentage of NHS budget allocated to wounds as a percentage to the yearly budget. Years 2020 and 2021 were different due to Covid-19. Source Kings Fund (14).

A recent analysis reported that the NHS in 2022 will receive £173.8 billion in funding (Kings Fund 2022). This means that 20.1% of this NHS budget in 2022 will be allocated to wounds. Figure 2 shows the percentage of the NHS budget used annually on wounds since 2012 and the proportion projected to 2035. With an annual growth of 11.1%, wounds clearly represent a looming catastrophe to the healthcare systems in the UK and other Western countries. Furthermore, as wounds are debilitating and will make people more dependent on help to continue living in their own homes, the increase in number of wounds, including the large number of non-healing wounds, will have a substantial knock-on effect on social care costs. Finally, the data also identify the resources that could be freed up across the NHS by introducing an effective wound treatment.

## Validating the cost estimate for 2012

Two sets of validation were performed to ensure that the costs of wounds calculated above are realistic. One was based on the estimation by Guest et al. of the number of new pressure ulcers in the UK in 2018 and the second was based on a study of the costs of pressure ulcers in Australia in 2020. Both calculations indicated that the costs estimated above are realistic.

|  | **2011** | **2012** | **2018** |
| --- | --- | --- | --- |
| New PUs | 96,332 | 106,928* | 200,000 |
| Unhealed PUs from previous year |  | 57,011** |  |
| Total number of PUs |  | 163,939 |  |
| Cost/PU (2012 prices) |  | £7,820*** |  |
| Total costs of PUs |  | £1,298,892,050 |  |
| Guest et al. (2015) PUs |  | £523,746,116**** |  |
| Factor difference |  | 2.45 |  |
| Total wounds Guest et al. (2015) |  | £5.228 billion***** |  |
| Total Estimated 2012 |  | £12.80 billion |  |

Table 7. Estimate of costs of wounds in 2012 based on Guest et al. (1,4) using pressure ulcers as example. *: The number of new PUs was estimated by Guest et al. (4) for 2018. This was calculated back to 2012 and 2011 assuming an annual increase of 11% (11). **: Year 2011 times healing rate twice (0.7693) to reflect reduction in numbers; non-healing PUs before 2011 are not included. ***: Cost per PU in 2012, assuming an inflation rate of 1.385% (Table 4) and a cost of £8,377 in 2015 (Table 5). ****: Total costs of PUs in 2012 based on Guest et al. (1) study in 2012 prices. *****: Total cost of wounds in 2012 prices.

Guest et al. (4) estimated that 200,000 new pressure ulcers would develop in 2018. Assuming an annual increase in wounds of 11% (11) and inflation of 1.385% (Table 4), Table 7 calculates the costs of pressure ulcers in 2012 at £1.3 billion, which is approx. 2.5 times more than estimated in Guest et al. (1). If this factor is applied to all costs in Guest et al. (1), the cost of wounds in 2012 becomes £12.8 billion, which is comparable to the estimated £13.7 billions presented above.

An Australian study (15) estimated the prevalence of pressure ulcers in hospital settings at 12.9% for 2020 and annual costs at AU$ 9.11 billion. Using an exchange rate of AU$1=£0.56958, a population of 25.69 million and 67.22 million for Australia and the UK, respectively for 2020, the estimated equivalent costs of pressure ulcers would be £13.58 billion in the UK in 2020. This is only for hospital in-patients with proportionally high costs of hospitalisation days, but the size of the amount indicates that a total cost of £28.3 billion for all wounds in community care (Table 6) is not unrealistically high considering that pressure ulcers in 2012 (Table 2) only represented 6.9% of all wounds and that by far the majority of pressure ulcers are treated in community care.

## References

1. Guest JF, Ayoub N, McIlwraith T, Uchegbu I, Gerrish A, Weidlich D, et al. Health economic burden that wounds impose on the National Health Service in the UK. BMJ Open. 2015 Dec 7;5(12):e009283.

2. Guest JF, Fuller GW, Vowden P. Venous leg ulcer management in clinical practice in the UK: costs and outcomes. Int Wound J. 2018 Feb;15(1):29–37.

3. Guest JF, Fuller GW, Vowden P. Diabetic foot ulcer management in clinical practice in the UK: costs and outcomes. International Wound Journal. 2018;15(1):43–52.

4. Guest JF, Fuller GW, Vowden P, Vowden KR. Cohort study evaluating pressure ulcer management in clinical practice in the UK following initial presentation in the community: costs and outcomes. BMJ Open. 2018 Jul 25;8(7):e021769.

5. Guest JF, Fuller GW, Vowden P. Costs and outcomes in evaluating management of unhealed surgical wounds in the community in clinical practice in the UK: a cohort study. BMJ Open. 2018 Dec 14;8(12):e022591.

6. Guest JF, Ayoub N, McIlwraith T, Uchegbu I, Gerrish A, Weidlich D, et al. Health economic burden that different wound types impose on the UK’s National Health Service. Int Wound J. 2017 Apr;14(2):322–30.

7. Urwin S, Dumville JC, Sutton M, Cullum N. Health service costs of treating venous leg ulcers in the UK: evidence from a cross-sectional survey based in the north west of England. BMJ Open. 2022 Jan 6;12(1):e056790.

8. NICE. Chronic wounds: advanced wound dressings and antimicrobial dressings [Internet]. NICE; 2016 [cited 2022 Dec 22]. Available from: https://www.nice.org.uk/advice/esmpb2/chapter/Key-points-from-the-evidence

9. FDA. FDA executive summary. Classification of wound dressings combined with drugs. Prepared for the Meeting of the General and Plastic Surgery Devices Advisory Panel September 20–21, 2016 [Internet]. FDA; 2016. Available from: https://www.fda.gov/media/100005/download

10. Verma KD, Lewis F, Mejia M, Chalasani M, Marcus KA. Food and Drug Administration perspective: Advancing product development for non-healing chronic wounds. Wound Repair Regen. 2022 May;30(3):299–302.

11. Guest JF, Fuller GW, Vowden P. Cohort study evaluating the burden of wounds to the UK’s National Health Service in 2017/2018: update from 2012/2013. BMJ Open. 2020 Dec 22;10(12):e045253.

12. Guest JF, Vowden K, Vowden P. The health economic burden that acute and chronic wounds impose on an average clinical commissioning group/health board in the UK. J Wound Care. 2017 Jun 2;26(6):292–303.

13. Wounds-UK. New research finds UK nurses carry out 180 wound dressing changes a year – on each chronic wound patient - Wounds UK [Internet]. 2018 [cited 2022 Dec 22]. Available from: https://www.wounds-uk.com/news/details/new-research-finds-uk-nurses-carry-out-180-wound-dressing-changes-a-year-on-each-chronic-wound-patient-

14. Kings Fund. NHS in a nutshell [Internet]. 2022. Available from: https://www.kingsfund.org.uk/projects/nhs-in-a-nutshell/nhs-budget

15. Nghiem S, Campbell J, Walker RM, Byrnes J, Chaboyer W. Pressure injuries in Australian public hospitals: A cost of illness study. International Journal of Nursing Studies. 2022 Jun 1;130:104191.
