# Supplementary material for: Stable closure of acute and chronic wounds and pressure ulcers and control of draining fistulas from osteomyelitis in persons with spinal cord injuries: non-interventional study of MPPT passive immunotherapy delivered via telemedicine in community care
Source: Front Med (Lausanne). 2024 Jan 5;10:1279100. doi: 10.3389/fmed.2023.1279100 (PMC10797031; doi:10.3389/fmed.2023.1279100)
Supplement: Supplementary file 2 [file Data_Sheet_2.docx]

# S2: Microbiome, SIS, AMR and virulence

External body areas are in constant, direct contact with the environment and are impossible to keep sterile. To prevent the invasion of pathogens, the body has therefore evolved a synergistic strategy, where it actively populates these surfaces, including our skin, with commensal microorganisms such as bacteria, archaea, viruses, protozoa, fungi, and mites, totalling over 1000 different species, which constitute our skin microbiome (Grice et al. 2009; Wang et al. 2021). It has also developed its own specialised skin immune system, SIS, with specialised immune cells within all three compartments of the skin (Abdallah et al. 2017). The microbiome and SIS act in a mutualistic relationship (Sanford and Gallo, 2013; Belkaid and Segre 2014).

The skin microbiome has a high degree of diversity and populates all three compartments of the skin. This physical occupation in itself makes it difficult for true pathogens or opportunistic commensal microbes to gain a foothold and spread. The composition of species populating the epidermis is unique to each individual and easily adaptable to changes within climate, hygiene, hormone status, food, age, sex, ethnicity, neurological system, and genetic predisposition, whereas the core microbiome of the dermis is relatively universal and stable (Bay et al. 2020). The *tela subcutanea* is the adipose compartment of the skin. Its microbiome has not yet been extensively investigated. Its basal membrane constitutes the last anatomical barrier between the skin and the sterile interior body containing e.g. muscles and bone.

The microbiome and the immune system are in constant communication and the immune system helps maintain a good balance in the microbial composition of the microbiome, ensuring a high degree of diversity and avoiding the dominance of one or a few species (Abdallah et al. 2017). The commensal bacteria, if sensing possible pathogens, will activate the immune response (Bhaskaran et al. 2018; Pandiyan et al. 2019).

Skin and wound infection develop as an imbalance in the microbiome (dysbiosis) of such severity that the immune system has lost control of the area with the result that one or a few microbial species have become dominant and taken over control from the immune system (Fig. 1).

Historically, to treat an infected (dysbiotic) wound, an antiseptic or an antibiotic was applied. When it was effective, it would kill the microbes present in the wound at that moment in time and act as a reset button, providing the immune system with a second opportunity to control the subsequent microbial repopulation process and ensure diversity and balance were observed. If the infection was caused by a true pathogen gaining control, the antiseptic would eliminate this pathogen, ensuring a repopulation by commensals.

Many microbes, including the commensals, are intrinsically resistant to antimicrobials and many already possess “intrinsic” or have “acquired” a high degree of clinical tolerance and resistance to both antibiotics (Ciofu & Tolker-Nielsen 2019; Antimicrobial Resistance Collaborators 2022) and antiseptics (Wassenaar et al. 2015; Wand et al. 2016; Ignak et al. 2017; Hassan et al. 2019; McCarlie et al. 2020; McCarlie 2021; van Dijk et al. 2022; Wicaksono et al. 2022). When an antibiotic is administered or an antimicrobial is applied to the wound, some of the microbes will survive while the rest are eliminated. The antimicrobial therefore does not act as a reset button, but actively favours the resistant microbial species to spread in the now non-occupied territory and take over control. As antimicrobial resistance is usually linked to increased microbial virulence (Bengoechea and Sa Pessoa 2019), the wound infection has become even more challenging and the required efforts by the immune system to fight the infection, i.e. regain control of the area, have actually increased (Fig. 2.) (Wang et al. 2021)

| ***Infection is an imbalance in the microbiome (dysbiosis)*** | | |  |
| --- | --- | --- | --- |
| *Red with border: Intrinsically resistant strain* | |  | |
| 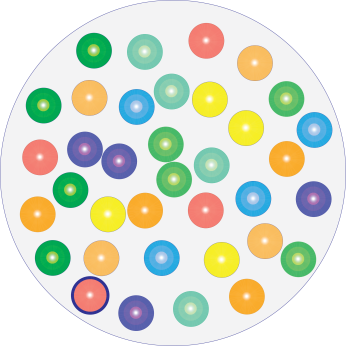  **B**  **A** | 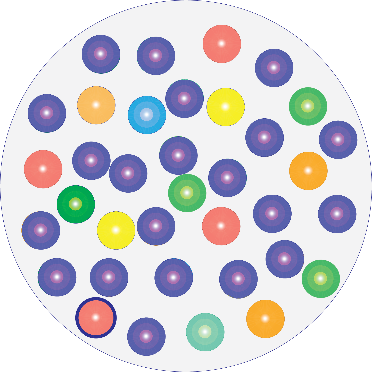 |  | |
| **Healing wound** | **Infected wound** |  | |
| Diverse and balanced microbiome. | One species is dominating and has taken over control. |  | |
| One resistant bacterium is among the commensals, but this does not cause a problem as no antimicrobials are present or being administered. | The microbiome is dysbiotic. As no antimicrobial is used, the dominating species that has taken over control has not been selected for resistance and therethrough increased virulence. Also, if it were to spread to other organs or into the bloodstream, effective antibiotic treatment will be available. |  | |

Figure 1.

| ***Dysbiosis in a wound containing a resistant bacterium and treated with an antimicrobial*** | | | |
| --- | --- | --- | --- |
| *Red with border:*  *Inrinsically resistant strain* |  | *Red with border and spikes:*  *Resistant strain with enhanced virulence* | |
| 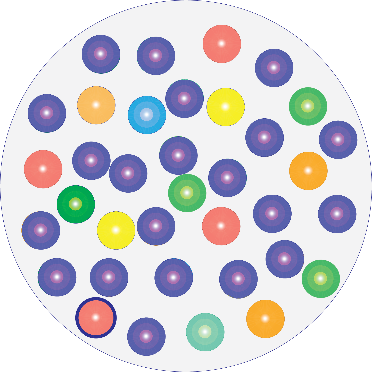  **B**  **A** | 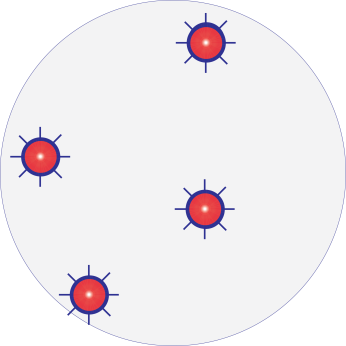 | | 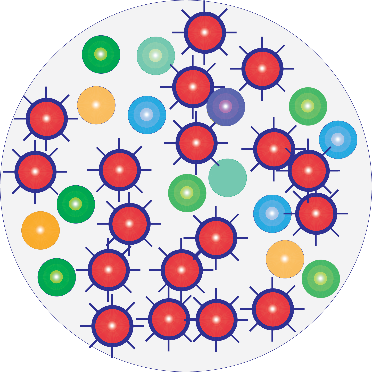  **C** |
| **Infected wound**  **with resistant bacteria** | **Immediately after treatment with antimicrobial** | | **A few hours following antimicrobial treatment** |
| Dysbiosis with one species dominating. | Resistant strain(s) survives and becomes more virulent. | | Infection has worsened and become more dangerous. |
| Unbalanced microbiome composition with one strain dominating. | Antimicrobial application favours the resistant, virulent strain. This can now reproduce and populate the space freed up and made available by the antimicrobial. | | The microbial composition has shifted. The resistant and more virulent strain has gained territory and is in control, causing infection.  Other microbial strains are starting to repopulate the area, but unable to restore balance. The diversity of the microbiome has been reduced, as some commensals are no longer present. |
| *Use of antimicrobials in wounds can exacerbate the infection.*  *(Jo et al. 2021; Wicaksono et al. 2022).* | | | |
| *Antiseptics affect all single-cell organisms, including the immune cells that have been mobilised to restore health (Bonacorsi et al. 2014) as well as the body’s keratinocytes, osteoblasts and fibroblasts, required for tissue regeneration (Yabes et al. 2017).* | | | |

Figure 2.

Consequently, treatment of wounds with antimicrobials will typically change the control of the wound space to a different microbial species. This can lead the clinician to believe that the antimicrobial has had a positive effect as the short-term clinical symptoms, e.g. level of exudate, inflammation and smell will have changed. The wound will, however, not proceed to healing and closure, because an infection (dysbiosis) remains - only with a different, and resistant, species in control, causing a different clinical symptomatology.

As resistance is often linked to increased virulence, this dominating, resistant species will typically be more invasive and aggressive than the former one. Whilst the clinical picture of this resistant infection can seem improved on the surface, it will typically have an increased ability to penetrate anatomical barriers, and to impede the immune response. Such invigorated spreading, potentially of both a contiguous and haematogenous nature, can easily take place in the deeper structures, out of sight. Consequently, the use of antimicrobials in wounds will typically favour and support more dangerous infections, associated with higher rates of morbidity and mortality (Bengoechea and Sa Pessoa, 2018).

As shown, systemic antibiotic use has been shown to shift the composition of the microbiome and to select for emergence and expansion of antimicrobial resistant bacteria on the skin (Jo et al. 2021). Likewise, antiseptics select for species that possess antimicrobial-resistant capabilities (McCarlie et al. 2020). As the microbial population is synergistic and distribute the specialised tasks among them within the biofilm (Liu et al. 2019), these capabilities are shared with the rest of the community, strengthening and fortifying the defence of the whole of the biofilm/community despite the fact that the species selected for may usually not be abundant (Wicaksono et al. 2022).

The treatment of a wound infection needs to restore the balance between the microbes in the skin microbiome. Ideally, it must not be antimicrobial so as not to contribute to antimicrobial resistance.

## References

- Abdallah F, Mijouin L, Pichon C. Skin Immune Landscape: Inside and Outside the Organism. *Mediators Inflamm*. 2017;2017:5095293. doi:10.1155/2017/5095293
- Antimicrobial Resistance Collaborators. Global burden of bacterial antimicrobial resistance in 2019: a systematic analysis. *Lancet*. 2022;399(10325):629-655. doi:10.1016/S0140-6736(21)02724-0
- Bay L, Barnes CJ, Fritz BG, et al. Universal Dermal Microbiome in Human Skin. *mBio*. 2020;11(1):e02945-19. Published 2020 Feb 11. doi:10.1128/mBio.02945-19
- Belkaid Y, Segre JA. Dialogue between skin microbiota and immunity. *Science*. 2014;346(6212):954-959. doi:10.1126/science.1260144
- Bengoechea JA, Sa Pessoa J. Klebsiella pneumoniae infection biology: living to counteract host defences. FEMS Microbiol Rev. 2019;43(2):123‐144. doi:10.1093/femsre/fuy043
- Bhaskaran N, Quigley C, Paw C, Butala S, Schneider E, Pandiyan P. Role of Short Chain Fatty Acids in Controlling T_regs_ and Immunopathology During Mucosal Infection. *Front Microbiol*. 2018;9:1995. Published 2018 Aug 24. doi:10.3389/fmicb.2018.01995
- Bonacorsi C, Raddi MS, Carlos IZ. Cytotoxicity of chlorhexidine digluconate to murine macrophages and its effect on hydrogen peroxide and nitric oxide induction. Braz J Med Biol Res. 2004;37(2):207‐212. doi:10.1590/s0100-879x2004000200007.
- Byrd AL, Belkaid Y, Segre JA. The human skin microbiome. *Nat Rev Microbiol*. 2018;16(3):143-155. doi:10.1038/nrmicro.2017.157
- Ciofu O, Tolker-Nielsen T. Tolerance and Resistance of *Pseudomonas aeruginosa* Biofilms to Antimicrobial Agents-How *P. aeruginosa* Can Escape Antibiotics. *Front Microbiol*. 2019;10:913. Published 2019 May 3. doi:10.3389/fmicb.2019.00913
- Grice EA, Kong HH, Conlan S, et al. Topographical and temporal diversity of the human skin microbiome. Science. 2009;324(5931):1190-1192. doi:10.1126/science.1171700
- Grice, E., Segre, J. The skin microbiome. *Nat Rev Microbiol* **9,** 244–253 (2011). <https://doi.org/10.1038/nrmicro2537>
- Ignak S, Nakipoglu Y, Gurler B. Frequency of antiseptic resistance genes in clinical staphycocci and enterococci isolates in Turkey. *Antimicrob Resist Infect Control*. 2017;6:88. Published 2017 Aug 30. doi:10.1186/s13756-017-0244-6
- Hassan KA, Naidu V, Edgerton JR, et al. Short-chain diamines are the physiological substrates of PACE family efflux pumps. *Proc Natl Acad Sci U S A*. 2019;116(36):18015-18020. doi:10.1073/pnas.1901591116
- Jo JH, Harkins CP, Schwardt NH, et al. Alterations of human skin microbiome and expansion of antimicrobial resistance after systemic antibiotics. *Sci Transl Med*. 2021;13(625):eabd8077. doi:10.1126/scitranslmed.abd8077
- Liu H, Chen X, Hu X, et al. Alterations in the gut microbiome and metabolism with coronary artery disease severity. *Microbiome*. 2019;7(1):68. Published 2019 Apr 26. doi:10.1186/s40168-019-0683-9
- Mc Carlie S, Boucher CE, Bragg RR. Molecular basis of bacterial disinfectant resistance. *Drug Resist Updat*. 2020;48:100672. doi:10.1016/j.drup.2019.100672
- McCarlie S. A new front. The Biologist. 2021 September 8. <https://thebiologist.rsb.org.uk/biologist-features/too-much-of-a-good-thing>
- Pandiyan P, Bhaskaran N, Zou M, Schneider E, Jayaraman S, Huehn J. Microbiome Dependent Regulation of T_regs_ and Th17 Cells in Mucosa. *Front Immunol*. 2019;10:426. Published 2019 Mar 8. doi:10.3389/fimmu.2019.00426
- Sanford JA, Gallo RL. Functions of the skin microbiota in health and disease. *Semin Immunol*. 2013;25(5):370-377. doi:10.1016/j.smim.2013.09.005
- van Dijk, H.F.G., Verbrugh, H.A. & Ad hoc advisory committee on disinfectants of the Health Council of the Netherlands. Resisting disinfectants. *Commun Med* **2,** 6 (2022). <https://doi.org/10.1038/s43856-021-00070-8>
- Wand ME, Bock LJ, Bonney LC, Sutton JM. Mechanisms of Increased Resistance to Chlorhexidine and Cross-Resistance to Colistin following Exposure of Klebsiella pneumoniae Clinical Isolates to Chlorhexidine. Antimicrob Agents Chemother. 2016;61(1):e01162-16. Published 2016 Dec 27. doi:10.1128/AAC.01162-16
- Wang, G., Sweren, E., Liu, H., Wier, E., Alphonse, M.P., Chen, R., Islam, N., Li, A., Xue, Y., Chen, J., Park, S., Chen, Y., Lee, S., Wang, Y., Wang, S., Archer, N.K., Andrews, W., Kane, M.A., Dare, E., Reddy, S.K., Hu, Z., Grice, E.A., Miller, L.S., Garza, L.A., 2021. Bacteria induce skin regeneration via IL-1β signaling. Cell Host & Microbe 29, 777-791.e6. https://doi.org/10.1016/j.chom.2021.03.003
- Wassenaar TM, Gunzer F. The prediction of virulence based on presence of virulence genes in E. coli may not always be accurate. *Gut Pathog*. 2015;7:15. Published 2015 Jun 19. doi:10.1186/s13099-015-0062-4
- Wicaksono WA, Erschen S, Krause R, Müller H, Cernava T, Berg G. Enhanced survival of multi-species biofilms under stress is promoted by low-abundant but antimicrobial-resistant keystone species. *J Hazard Mater*. 2022;422:126836. doi:10.1016/j.jhazmat.2021.126836
- Yabes JM, White BK, Murray CK, et al. In Vitro activity of Manuka Honey and polyhexamethylene biguanide on filamentous fungi and toxicity to human cell lines. *Med Mycol*. 2017;55(3):334-343. doi:10.1093/mmy/myw070
