# Supplementary material for: Stable closure of acute and chronic wounds and pressure ulcers and control of draining fistulas from osteomyelitis in persons with spinal cord injuries: non-interventional study of MPPT passive immunotherapy delivered via telemedicine in community care
Source: Front Med (Lausanne). 2024 Jan 5;10:1279100. doi: 10.3389/fmed.2023.1279100 (PMC10797031; doi:10.3389/fmed.2023.1279100)
Supplement: Supplementary file 3 [file Data_Sheet_3.docx]

# S3: MPPT, MoA, evidence, use and telemedicine

MPPT (micropore particle technology) is a first-in-class anti-infective passive immunotherapy that acts via the microbiome (Sams-Dodd and Sams-Dodd 2018). MPPT is approved in the UK and EU as Amicapsil for all wound types and as Amicapsil-SCI for persons, who are immunocompromised, e.g. persons with spinal cord injury. SertaSil is the veterinary version. MPPT has been approved in the EU since 2016 and is classified as a medical device based on its mode-of-action. It is approved as a treatment for wounds, i.e. with a therapeutic outcome, and is available over-the-counter. The World Anti-Doping Agency (WADA) in Canada has concluded that its use in wound care is permitted in sports, i.e. there are no doping issues.

## Mode-of-action

MPPT is a white powder that uses coupled capillary-evaporation, i.e. micro-pumping, to remove excess moisture from the skin and wound surfaces. This, in parallel, removes the toxins and enzymes that microbes secrete to inactivate immune cells and competing microbes as well as to break down tissue. Bacteria and fungi also secrete biofilm to create a protecting shield. In the state of infection, the infecting species typically reinforce this biofilm and renders it exceptionally hard for other species as well as immune cells to penetrate. The pumping process of MPPT creates holes in this layer, thereby allowing immune cells to enter. MPPT therefore disrupts the microbes’ virulence factors, such as enzymes, toxins and biofilm, in other words, its “offence and defence systems”. This enables the body’s immune cells to resume control of the wound and remove the infection – hence an immunotherapy. Due to the mode-of-action of MPPT, it is important that the wound is cleaned only with tap-water and that air can move freely across the wound surface to support evaporation and to suppress anaerobic organisms.

## Antimicrobial resistance

MPPT involves no antimicrobial components; it is effective against antimicrobial resistant strains; and it will not contribute to the creation of new resistance. In contrast, both antibiotics and antiseptics (which are currently the most used products in the management of infected wounds) contribute to AMR and, in a wound containing resistant species, antimicrobials will favour the resistant strains. As resistance tends to be linked to increased virulence (Bengoechea and Sa Pessoa 2019), antimicrobials are likely to exacerbate the infection.

## Environment

MPPT only contains natural non-toxic ingredients that are readily returned to the biological cycle. Its use only requires tap-water and natural cotton, all packaging is recyclable, and patients or their carers can perform the dressing changes, thus eliminating the need for transport. Unlike antimicrobials, it has no negative effects on biodiversity, greenhouse gases or the aquatic environment.

## Safety

MPPT contains no toxic ingredients and has not been associated with any side-effects such as wound irritation, allergy or bleeding, including after daily application onto muscle and bone for over 2 years.

## Efficacy

A preclinical study (Bilyayeva et al. 2014) performed in a rat wound healing model found that MPPT resulted in a wound bed free of infection 60% quicker than a topical antibiotic (gentamicin) group and untreated controls. The study confirmed that MPPT is not antimicrobial, and that it promotes increased migration of neutrophils, macrophages and lymphocytes to the wound and accelerates the progression from the inflammatory to the proliferative wound healing phase.

A comparative clinical study with 266 hospital in-patients (Bilyayeva et al. 2017), covering a range of wound types, found that the use of MPPT resulted in a wound free from infection following daily application for 3 days (mean), whereas a topical antibiotic (gentamicin) required 7 days and an antiseptic (iodine) 8 days, i.e. a 60% acceleration by MPPT. Overall, MPPT reduced the number of hospitalisation days by 31% compared to gentamicin and by 39% compared to iodine. The study included different subgroups, i.e. acute wounds (infected surgical wounds, abscesses, carbuncles), diabetic foot ulcers, venous leg ulcers, and burns. It showed that the time to a non-infected wound was independent of wound type, whereas the reduction in number of hospitalisation days appeared to depend upon the underlying disease process. The reduction in hospitalisation days by MPPT compared to gentamicin was 41% for acute wounds; 31% for diabetic foot ulcers; and 19% for venous leg ulcers; and compared to iodine 44% for acute wounds; 51% for diabetic foot ulcers; and 36% for venous leg ulcers. The effects of the topical antibiotic and the antiseptic were in line with generally published data, and the outcome of the study, i.e. the effects of MPPT and gentamicin, were identical to the preclinical study, which supports the robustness of the findings.

Ryan (2017) conducted a clinical case-series at Bristol University Hospital, which included nine acute dehisced surgical wounds and one non-healing category 4 pressure ulcer (non-SCI). The grade 4 pressure ulcer had unsuccessfully been treated with a variety of approaches for 4 weeks before MPPT. In all wounds, the use of MPPT led to an infection-free, healing wound in 3-5 days and all wounds that could be followed were confirmed to have reached closure. Standard-of-care at the hospital consisted of 1 week with UrgoClean followed by 2 or more weeks with NPWT to reach a healing wound, allowing discharge. This same level of healing was achieved in 3-5 days with MPPT, i.e. a 81% shortening in time.

Lovgren et al. (2018) showed efficacy of MPPT on chronic, non-responsive pyoderma gangrenosum ulcers.

O’Sullivan et al. (2020) reported efficacy and cost-effectiveness of MPPT in treating a chronic wound in a person with spinal cord injury compared to standard care.

In-house data include cases of trauma wounds, dehisced surgical wounds, burns, diabetic foot ulcers, venous leg ulcers, and dermatological lesions such as hidradenitis suppurativa. MPPT has consistently been able to treat wound infection and support healing. In many cases, laypersons were responsible for the hands-on dressing changes.

## How to Use

Once daily, the wound is thoroughly washed, preferably showered, using plenty of clean tap-water (not saline, see Angerås et al. 1992). The wound is gently dapped relatively dry. In shallow wounds, MPPT is applied in an unbroken 1-2 mm layer to the entire wound surface and wound edges including 5-10 mm beyond onto healthy skin. If no dressing is to be worn, the thickness will be determined by how much MPPT will stick to the surface, but the layer must always be unbroken. In deep cavity wounds with long relatively narrow sinus or fistula formation either under the skin or tracking deeper into the muscle, as well as in wounds with cavities widening once past the opening in the skin, MPPT is applied onto all surfaces that can be reached, including all sides lining a tunnel, bottom area of a sinus and all angles of a cavity until all surface-linings are covered. Cavities should not be filled up. Undermining has MPPT applied to both “floor” and “ceiling” area. Red, irritated, inflamed, nodulous, or cracked areas of skin surrounding any type of wound or in proximity to the wound should have MPPT gently massaged into the affected skin and 5-10 mm beyond onto healthy skin. If any area is too dry for MPPT to stick, it can very gently be touched with a moist gauze before application.

MPPT acts via the evaporation of moisture and requires air-circulation across the wound surface. Depending on individual circumstances, the wound should either be left uncovered or only covered with a woven, 100% cotton, gauze swab. When free access to air is impeded, e.g. when the person is sitting on the wound or it must be covered by footwear or clothing, air can be supplied to the wound surface using a simple portable air-pump that, through soft pharma-grade silicone tubing, delivers a gentle flow of air across the wound surface. This can be combined with absorbent wound dressings and can provide the person full freedom of movement. Bed rest is not required. Extended bed rest is known to have a detrimental impact on health with a high risk of leading to conditions that are difficult to treat and increase dependence on caregivers (Dittmer and Teasell 1993; Teasell and Dittmer 1993).

Acute wounds in immunocompetent individuals only require once daily application of MPPT for 3-5 days to reach a healing stage that enables the wound to close without further treatment (Ryan 2017). Wounds in immunocompromised persons, however, usually require daily application until the wound surface has fully epithelialized and occasionally even stabilised.

Systemic antibiotics used concomitantly with MPPT for treating a different, non-wound-related infection will, in immunocompetent individuals, normally only cause the healing to stall for approximately 24 hours in connection with starting and stopping the course. However, in immunocompromised individuals the use of antibiotics, e.g., for treating UTIs, can completely stall healing and might even cause a flare-up of wound infection caused by resistant species, thereby extending the required MPPT treatment period.

## References

1. Angerås MH, Brandberg A, Falk A, Seeman T. Comparison between sterile saline and tap water for the cleaning of acute traumatic soft tissue wounds. *Eur J Surg*. 1992;158(6-7):347-350.
2. Bengoechea JA, Sa Pessoa J. Klebsiella pneumoniae infection biology: living to counteract host defences. FEMS Microbiol Rev. 2019;43(2):123‐144. doi:10.1093/femsre/fuy043
3. Bilyayeva O, Neshta VV, Golub A, Sams-Dodd F. Effects of SertaSil on wound healing in the rat. J Wound Care. 2014, 23(8):410, 412-4, 415-6.
4. Bilyayeva OO, Neshta VV, Golub AA, Sams-Dodd F. Comparative Clinical Study of the Wound Healing Effects of a Novel Micropore Particle Technology: Effects on Wounds, Venous Leg Ulcers, and Diabetic Foot Ulcers. Wounds. 2017; 29(8):1-9.
5. Dittmer DK, Teasell R. Complications of immobilization and bed rest. Part 1: Musculoskeletal and cardiovascular complications. *Can Fam Physician*. 1993;39:1428-1437.
6. Lovgren M-L, Wernham A, James M, Martin-Clavijo A. Pyoderma gangrenosum ulcers treated with novel micropore particle technology. Br.J.Dermatol. 2018; 179 (Suppl. 1):BI22, p. 152.
7. O'Sullivan O, Hayton L, Findlay-Cooper K, Phillip R. Novel micropore particle technology for spinal cord injury chronic wound healing: a new paradigm? [published online ahead of print, 2020 Aug 4].
8. Ryan E. The use of a micropore particle technology in the treatment of acute wounds. J Wound Care. 2017; 26(7): 404-413.
9. Sams-Dodd J, Sams-Dodd F. Time to Abandon Antimicrobial Approaches in Wound Healing: A Paradigm Shift. Wounds. 2018; 30(11):345-352.
10. Sams-Dodd J, Sams-Dodd F. Micropore Particle Technology Promotes Wound Healing, Whereas Polyhexamethylene Biguanide Causes Tissue Degeneration: A Case Report. Wounds. 2020;32(3):E6-E10.
11. Teasell R, Dittmer DK. Complications of immobilization and bed rest. Part 2: Other complications. *Can Fam Physician*. 1993;39:1440-1446.
