# Supplementary material for: Stable closure of acute and chronic wounds and pressure ulcers and control of draining fistulas from osteomyelitis in persons with spinal cord injuries: non-interventional study of MPPT passive immunotherapy delivered via telemedicine in community care
Source: Front Med (Lausanne). 2024 Jan 5;10:1279100. doi: 10.3389/fmed.2023.1279100 (PMC10797031; doi:10.3389/fmed.2023.1279100)
Supplement: Supplementary file 4 [file Data_Sheet_4.docx]

# S4: SCI, immune dysfunction, osteomyelitis and aim of MPPT

## Immune dysfunction

A spinal cord injury affects the communication between the immune system and the nervous system. The result is chronic immune dysfunction, i.e. an impaired or suppressed immune response (Riegger et al. 2009; Schwab et al. 2014; Carpenter et al. 2020; Bietar et al. 2021). This has also been described as “spinal cord injury-induced immune deficiency syndrome” (SCI-IDS) (Riegger et al. 2007, 2009) or as “immune paralysis” (Brommer et al. 2016). The effects of spinal cord injury on wound healing have been replicated in animal models and it was found that the immune response to wound infections was reduced by approximately 50% (Marbourg et al. 2017; Kumar et al. 2018) and that spinal cord injury significantly increases susceptibility to infections (Brommer et al. 2016).

## Microbiomes within changed immune function

The immune system controls the microbiome (See S2: Microbiomes, SIS, AMR and virulence), and a spinal cord injury will consequently affect the composition and function of the microbiome (Kigerl et al. 2016; Valido et al. 2022).

In SCI, the permeability of the gut increases and microbes, that normally would be restricted to the gut, can now cross the intestinal wall with greater ease and pass into the blood stream. Here, the microbes will be distributed throughout the body and can cause infections (Valido et al. 2022). This means, that people with SCI have increased numbers of microbes circulating their body at any one time, and these can potentially settle anywhere in the capillary networks of the body or anywhere with a temporary or chronic weak point. Where they settle, they will, as any other organism, endeavour to thrive and multiply and can give rise to an infection that can be difficult to fight, given the immunosuppression of people with a SCI. These circulating bacteria can give rise to an *internal* focus of infection, starting from within and working its way out towards the skin. This is most likely the reason why ulcers in people with SCI can be both old and heavily infected before they even proceed to breaching the skin. As an example, a bacteria can settle in a cluster of soft tissue cells that have been damaged by potentially only brief exposure to excess pressure which has rendered them non-viable. Such limited internal necrotic incidents would normally be dealt with and absorbed by the body’s defence mechanisms. However, the immune system of persons with SCI is unable to combat this infection due to the compromised immune response. This also explains why such ulcers have a tendency to be shaped as tunnels containing a “plug” of necrotic tissue, typically on top of a bone tuberosity, *before* they even break through the skin.

With the changes in immune function, the skin microbiome also changes in SCI. Among others, *Pseudomonas aeruginosa; Staphylococcus aureus, Klebsiella pneumoniae* and *Serratia marcescens* (formerly classified as part of the Enterobacter family) (Ciofu & Tolker-Nielsen 2019; Fawcett et al. 1986; Ogura et al. 2022) become permanent residents of the skin. These are usually resistant to antimicrobials. P*. aeruginosa, S. marcescens and K. pneumoniae* are known to cause chronic infections, among others in the lungs, the urinary tract and soft tissue, i.e. the areas of special concern in persons with SCI. They are all known to cause bacteraemia, meningitis and sepsis (Ciofu & Tolker-Nielsen 2019). *S. aureus* is the sole most common causative agent of osteomyelitis (Lew and Waldvogel, 2004; Muthukrishnan et al 2019), now accompanied by the increasingly virulent *Staphylococcus epidermidis* (Kavanagh et al. 2018), also a common inhabitant of the skin of SCI people. Antibiotic treatment cannot normally eradicate these infections due to their *intrinsic* antibiotic tolerance *and* the development of *mutational* antibiotic resistance (Ciofu & Tolker-Nielsen 2019; Zembower 2015). These bacteria may even work in synergy to exacerbate the attack on the immune defences (DeLeon et al. 2014) and on the human tissue (Alves et al. 2018).

## Osteomyelitis

Osteomyelitis can develop via multiple mechanisms. Osteomyelitis associated with a wound or ulcer in SCI people typically has two major avenues of origin, haematogenous and contiguous. The haematogenous spread is *endogenous* and is caused by an infective agent being carried via the bloodstream, e.g. as mentioned above, and caught in a capillary bed including the periosteal or cortical bone capillaries, or in a dense troubled area such as an accumulation of necrotic tissue of any type or size. In this scenario, the infective agent is operating in the area inferior to the *tela subcutanea* of the hypodermis, typically muscle or bone. As the infection expands, the increased amount of debris it creates will gradually infect and break down the soft tissue between the bone and skin and eventually break through the skin from within, creating an ulcer. The contiguous spread is *exogenous* and occurs when infection in adjacent tissue penetrates the protecting periosteum and invades the bone, e.g. through the osteocyte lacuno-canalicular network (Masters et al. 2019a,b). This is the typical avenue for an osteomyelitis caused by a wound infection that spreads from a skin lesion through the compartments of the skin and penetrates the *tela subcutanea*. This is the last anatomical barrier of all three skin compartments and, having reached muscle, the infection spreads easily to bone.

Infection in a wound or pressure ulcer that is operating below *tela subcutanea*, i.e. in muscle, (grade 4) can spread relatively freely in the tissue where the specialised skin immune response is absent (See S2: Microbiome, SIS, AMR and virulence). This increases the risk of sepsis from the soft tissue and the risk of developing osteomyelitis of a contiguous origin. The risk of developing osteomyelitis from a pressure ulcer is high, e.g. Rennert et al. (2009) reported that 32% of all grade 4 ulcers (i.e. in muscle) lead to osteomyelitis and Russel et al. (2020) reported that the median age of the ulcer giving rise to the osteomyelitis is only 4 months, but can be down to 7 weeks. The bone may seem well covered with soft tissue even by a trained eye and still be infected, as the inability to probe to bone does not rule out osteomyelitis (Fritz and McDonald, 2008). The presence of osteomyelitis also considerably increases the risk of sepsis.

Osteomyelitis is difficult to diagnose and, whilst MRI, CT and X-ray are helpful tools, their value in this condition has limitations (Sgarzani et al. 2019; Masters et al. 2022). Osteomyelitis can therefore only be relatively reliably diagnosed by biopsy (Brunel et al. 2015; Panteli and Giannoudis 2016) and even this only has a sensitivity of 87% (Fritz and McDonald, 2008).

Osteomyelitis (over 6 weeks old) can only be resolved with bone surgery, where the infected part of the bone is physically removed (Panteli and Giannoudis 2016; <https://bestpractice.bmj.com/topics/en-gb/3000178>). Russel et al. (2020) reported a 71% failure rate of osteomyelitis surgery in patients with pelvic osteomyelitis associated with a pressure ulcer. They also found that, in 64% of the patients operated for osteomyelitis, the wound did not heal and the patients died within a median of 2 years of first attempt of surgery. The remaining 36% healed, but patients still died within a median of 7 years of first surgery. Comparable findings were made by Van Beest et al. (2022). These findings are consistent with the view that osteomyelitis cannot be cured, but should be seen as a recurrent or intermittent disease for which “a ‘cure’ cannot be safely declared.” (Panteli and Giannoudis 2016; Masters et al. 2022). Rabadi et al. (2013), Kriz et al. (2021) and Thietje et al. (2022) reported that 10% - 12% of people with SCI will die of direct complications from pressure ulcers.

## Draining fistulas caused by underlying primary focus of infection

What presents as a wound can be a draining fistula for an underlying primary infectious condition such as osteomyelitis or an anal fistula. These continuously release debris, that in itself is both infectious and toxic to the immune cells as well as to the tissue, and that disseminates from the bone into the adjacent soft tissue causing abscesses, infective collections, and wider tissue break-down. The body must minimise the damage that this harmful debris causes in all directions within the soft tissue, and will, therefore, endeavour to push it to the surface of the body and through the skin for disposal. Depending on whether the osteomyelitis is haematogenous or contiguous in origin, this undertaking either opens a new wound from within, or utilises the route through an already open wound, respectively. An internal primary focus of infection, e.g. osteomyelitis or an anal fistula, will consequently inevitably always be associated with an exuding wound, serving as a debris-draining fistula. The level of exudate can vary to a very great extent and depends on a variety of factors. As the debris is highly infectious and toxic to the soft tissue, and as the infection never ceases to release these substances, such wounds often present with severe tissue breakdown and further widely disseminated soft tissue infection. Chronic osteomyelitis, typically more than 6 weeks old, will continue to spread in the bone until it is removed surgically (Panteli and Giannoudis 2017). The variable speed with which the spread in the bone progresses is influenced by many factors (Masters, Trombetta et al. 2019). Chronic osteomyelitis should be regarded as a recurrent or intermittent disease with low probability of a cure, even if surgery is performed (Panteli and Giannoudis 2017; Masters et al. 2022).

## Aim of MPPT for managing draining fistulas

In these draining fistulas - that look like severe, deep wounds - the aim of MPPT treatment is to control and reduce the soft tissue infection in preparation for surgery to be performed in minimally infected tissue, as well as to preserve as much soft tissue as possible to enable primary closure without tissue grafting techniques and secure minimal loss of soft tissue required for cushioning to minimise the risk of future ulcers in the same area. In cases where surgery cannot be performed, e.g. due to comorbidities, or prolonged waiting times for surgery, the osteomyelitis will expand over time (can be years) along a wider area of the bone and will consequently be releasing increasing amounts of debris from a widened infective focus. As this happens, material that needs to drain to the surface, may be generated relatively far from the initial focus with its accompanying fistula-wound, and, instead of travelling along the bone and through the existing fistula, new fistulas may form through which this more distant debris can travel. In such cases of palliative use of MPPT, the aim of the MPPT treatment is to control and minimise the soft tissue infection, breakdown and loss, and to reduce the risk of sepsis posed by soft tissue infection, including cellulitis.

## Conclusion

In conclusion, people with SCI are immunocompromised and at high risk with regards to infections and any entry point of potential infection should be prevented or promptly dealt with. Soft tissue infection alone can spread into the blood stream and cause sepsis. Once an infective agent reaches muscle, the microbes can spread with little constraint and may reach the bone, resulting in osteomyelitis which can develop very quickly and is associated with a high degree of morbidity and mortality.

## References

- Alves PM, Al-Badi E, Withycombe C, Jones PM, Purdy KJ, Maddocks SE. Interaction between Staphylococcus aureus and Pseudomonas aeruginosa is beneficial for colonisation and pathogenicity in a mixed biofilm. *Pathog Dis*. 2018;76(1):10.1093/femspd/fty003. doi:10.1093/femspd/fty003
- Bietar B, Lehmann C, Stadnyk AW. Effects of CNS Injury-Induced Immunosuppression on Pulmonary Immunity. *Life (Basel)*. 2021;11(6):576. Published 2021 Jun 18. doi:10.3390/life11060576
- Brommer B, Engel O, Kopp MA, et al. Spinal cord injury-induced immune deficiency syndrome enhances infection susceptibility dependent on lesion level. *Brain*. 2016;139(Pt 3):692-707. doi:10.1093/brain/awv375
- Brunel et al. 2015, Diagnosing pelvic osteomyeltisi beneath pressure ulcers in spinal cord injured patients: a prospective study.
- Carpenter RS, Marbourg JM, Brennan FH, et al. Spinal cord injury causes chronic bone marrow failure. *Nat Commun*. 2020;11(1):3702. Published 2020 Jul 24. doi:10.1038/s41467-020-17564-z
- Ciofu O, Tolker-Nielsen T. Tolerance and Resistance of *Pseudomonas aeruginosa* Biofilms to Antimicrobial Agents-How *P. aeruginosa* Can Escape Antibiotics. *Front Microbiol*. 2019;10:913. Published 2019 May 3. doi:10.3389/fmicb.2019.00913
- DeLeon S, Clinton A, Fowler H, Everett J, Horswill AR, Rumbaugh KP. Synergistic interactions of Pseudomonas aeruginosa and Staphylococcus aureus in an in vitro wound model. *Infect Immun*. 2014;82(11):4718-4728. doi:10.1128/IAI.02198-14
- Fawcett C, Chawla JC, Quoraishi A, Stickler DJ. A study of the skin flora of spinal cord injured patients. *J Hosp Infect*. 1986;8(2):149-158. doi:10.1016/0195-6701(86)90041-1
- Kavanagh N, Ryan EJ, Widaa A, et al. Staphylococcal Osteomyelitis: Disease Progression, Treatment Challenges, and Future Directions. *Clin Microbiol Rev*. 2018;31(2):e00084-17. Published 2018 Feb 14. doi:10.1128/CMR.00084-17.
- Kigerl KA, Hall JC, Wang L, Mo X, Yu Z, Popovich PG. Gut dysbiosis impairs recovery after spinal cord injury. *J Exp Med*. 2016;213(12):2603-2620. doi:10.1084/jem.20151345Kriz, J., Sediva, K., Maly, M., 2021. Causes of death after spinal cord injury in the Czech Republic. Spinal Cord 59, 814–820. https://doi.org/10.1038/s41393-020-00593-2
- Kumar S, Yarmush ML, Dash BC, Hsia HC, Berthiaume F. Impact of Complete Spinal Cord Injury on Healing of Skin Ulcers in Mouse Models. *J Neurotrauma*. 2018; 35(6):815-824
- Lew DP, Waldvogel FA. Osteomyelitis. *Lancet*. 2004;364(9431):369-379. doi:10.1016/S0140-6736(04)16727-5
- Marbourg JM, Bratasz A, Mo X, Popovich PG. Spinal Cord Injury Suppresses Cutaneous Inflammation: Implications for Peripheral Wound Healing. J Neurotrauma. 2017; 34(6):1149-1155.
- Masters EA, Trombetta RP, de Mesy Bentley KL, et al. Evolving concepts in bone infection: redefining "biofilm", "acute vs. chronic osteomyelitis", "the immune proteome" and "local antibiotic therapy". *Bone Res*. 2019;7:20. Published 2019 Jul 15. doi:10.1038/s41413-019-0061-z
- Masters EA, Salminen AT, Begolo S, et al. An in vitro platform for elucidating the molecular genetics of S. aureus invasion of the osteocyte acuna-canalicular network during chronic osteomyelitis. *Nanomedicine*. 2019;21:102039. Doi:10.1016/j.nano.2019.102039
- Masters EA, Ricciardi BF, Bentley KLM, Moriarty TF, Schwarz EM, Muthukrishnan G. Skeletal infections: microbial pathogenesis, immunity and clinical management. *Nat Rev Microbiol*. 2022;20(7):385-400. doi:10.1038/s41579-022-00686-0
- Muthukrishnan G, Masters EA, Daiss JL, Schwarz EM. Mechanisms of Immune Evasion and Bone Tissue Colonization That Make Staphylococcus aureus the Primary Pathogen in Osteomyelitis. *Curr Osteoporos Rep*. 2019;17(6):395-404. doi:10.1007/s11914-019-00548-4
- Ogura K, Furuya H, Takahashi N, et al. Interspecies Regulation Between *Staphylococcus caprae* and *Staphylococcus aureus* Colonized on Healed Skin After Injury. *Front Microbiol*. 2022;13:818398. Published 2022 Mar 1. doi:10.3389/fmicb.2022.818398
- Panteli M, Giannoudis PV. Chronic osteomyelitis: what the surgeon needs to know. *EFORT Open Rev*. 2017;1(5):128-135. Published 2017 Mar 13. doi:10.1302/2058-5241.1.000017
- Rabadi, M.H., Mayanna, S.K., Vincent, A.S., 2013. Predictors of mortality in veterans with traumatic spinal cord injury. Spinal Cord 51, 784–788. <https://doi.org/10.1038/sc.2013.77>
- Rennert R, Golinko M, Yan A, Flattau A, Tomic-Canic M, Brem H. Developing and evaluating outcomes of an evidence-based protocol for the treatment of osteomyelitis in Stage IV pressure ulcers: a literature and wound electronic medical record database review. Ostomy Wound Manage. 2009;55(3):42-53.
- Richards JS, Waites K, Chen YY et al. The Epidemiology of Secondary Conditions Following Spinal Cord Injury. Top Spinal Cord Inj Rehabil 2004;10(1):15–29
- Riegger T, Conrad S, Schluesener HJ, et al. Immune depression syndrome following human spinal cord injury (SCI): a pilot study. Neuroscience. 2009;158(3):1194‐1199. doi:10.1016/j.neuroscience.2008.08.021
- Russell CD, Tsang SJ, Simpson AHRW, Sutherland RK. Outcomes, Microbiology and Antimicrobial Usage in Pressure Ulcer-Related Pelvic Osteomyelitis: Messages for Clinical Practice. J Bone Jt Infect. 2020;5(2):67-75. Published 2020 Mar 26. doi:10.7150/jbji.41779
- Schwab JM, Zhang Y, Kopp MA, Brommer B, Popovich PG. The paradox of chronic neuroinflammation, systemic immune suppression, autoimmunity after traumatic chronic spinal cord injury. Exp Neurol. 2014; 258:121-129.
- Sgarzani R, Sara T, Trapani FF, et al. Osteomyelitis of the pelvic bones in patients with spinal cord injury: Is magnetic resonance useful for preoperative diagnosis?. Integr Mol Med. 2019; 6 DOI: 10.15761/IMM.1000373.Thietje, R., Kowald, B., Böthig, R., Schulz, A.P., Northmann, M., Rau, Y., Hirschfeld, S., 2022. Long-Term Survival and Causes of Death in Patients below the Age of 60 with Traumatic Spinal Cord Injury in Germany. Journal of Clinical Medicine 11, 26. https://doi.org/10.3390/jcm11010026
- Valido E, Bertolo A, Fränkl GP, et al. Systematic review of the changes in the microbiome following spinal cord injury: animal and human evidence. *Spinal Cord*. 2022;60(4):288-300. doi:10.1038/s41393-021-00737-y
- Van Beest D, Koh SJ, Tzen YT, et al. Healthcare utilization and outcomes of spinal cord injured veterans with stage 3-4 pressure injuries [published online ahead of print, 2022 Apr 4] [published correction appears in J Spinal Cord Med. 2022 Apr 25;:1]. *J Spinal Cord Med*. 2022;1-9. doi:10.1080/10790268.2022.2052500
- Zembower N, Zhu A, Malczynski M. Brain and Spinal Cord Injury Patients With Klebsiella pneumoniae Carbapenemase (KPC)-Producing K. pneumoniae: Prevalence and Potential for Prolonged Colonization, Open Forum Infectious Diseases, Volume 2, Issue suppl_1, December 2015, 1795, <https://doi.org/10.1093/ofid/ofv133.1345>
