# Supplementary material for: Stable closure of acute and chronic wounds and pressure ulcers and control of draining fistulas from osteomyelitis in persons with spinal cord injuries: non-interventional study of MPPT passive immunotherapy delivered via telemedicine in community care
Source: Front Med (Lausanne). 2024 Jan 5;10:1279100. doi: 10.3389/fmed.2023.1279100 (PMC10797031; doi:10.3389/fmed.2023.1279100)
Supplement: Supplementary file 5 [file Data_Sheet_5.docx]

# S5: Choice of clinical study design for evaluating a wound treatment in SCI-persons

## Choice of study design

The aim was to evaluate MPPT for the treatment of wounds and pressure ulcers in persons with spinal cord injury (SCI). The traditional design for a clinical trial would be to compare the effects of MPPT to a placebo group and a comparator representing the generally accepted treatment approach in the field, the “gold standard”, with participants being randomised to treatment arms.

The ICH (The International Council for Harmonisation of Technical Requirements for Pharmaceuticals for Human Use) in 2000 published a guidance on the choice and use of comparators and placebo controls in clinical trials. The guidance states:

“*In cases* ***where an available treatment is known to prevent serious harm, such as death or irreversible morbidity in the study population, it is generally inappropriate to use a placebo control****.*” Section 2.1.3. EMA 2001 / ICH E10 (2000).

“***Where there is therapy known to be effective in preventing death or irreversible morbidity, it is no more ethically acceptable to randomize deliberately to subeffective control therapy than it is to randomize to placebo****.*” Section 2.3.3. EMA 2001 / ICH E10 (2000).

Therefore, according to guidance, it is not acceptable to include a placebo control or a comparator, if their inclusion will expose the participants to avoidable risk of death or irreversible morbidity, i.e. life-long implications. A comparator must be a generally accepted, standardised treatment regimen demonstrated to be effective.

The first question is therefore whether wounds and pressure ulcers in persons with spinal cord injury give rise to mortality and irreversible morbidity such that the use of comparators needs to be questioned. The second question is whether an accepted gold standard exists which has demonstrated effects in clinical trials and can act as a meaningful representative of the current state of the field. Thirdly, the question must be asked whether, based on existing data, MPPT can be expected to be superior to these options to the extent that comparators from an ethical viewpoint should be excluded because it is already known that they will expose participants to the risk of mortality and irreversible morbidity and because the degree of efficacy of MPPT would be sufficient to allow reliance on external controls for data analysis. In oncology, it is increasingly becoming the norm to perform single-arm trials due to the seriousness of the condition and the lack of effective treatments, and a comparable situation may exist for the current evaluation. The use of external controls is discussed in S6: Study design, controls, outcome (safety, efficacy, cost), cost comparisons and sustainability.

## Impact of pressure ulcers and wounds in SCI-persons

At the time of designing the study, it was known that people with spinal cord injury are immunocompromised due to the loss of communication between the immune system and the nervous system (Riegger et al. 2009; Schwab et al. 2014):

- SCI-persons show enhanced infection susceptibility and have impaired ability to fight infection (Riegger et al. 2009; Schwab et al. 2014; Brommer et al. 2016).
- Marbourg et al. (2017) found the number of macrophages decreased by 50% in a mouse model of SCI in response to a challenge and concluded this would be consistent with impaired wound healing.

It was also known that pressure ulcers are a very serious complication of SCI that has a considerable impact on quality of life and physical health:

- Pressure ulcers have the potential to “interfere with physical, psychological and social well-being and to impact overall quality of life” (Consortium for Spinal Cord Medicine 2000, p. 9).
- Pressure ulcers may “disrupt rehabilitation, prevent individuals with SCI from attending work or school, and interfere with community reintegration” (Houghton et al. 2013, p. 6).
- The occurrence of a pressure ulcers can lead to hospitalization often with an extended length of stay (Fuhrer et al. 1993; Krause 1998; Consortium for Spinal Cord Medicine 2000).

It was furthermore known that pressure ulcers and wounds could cause soft tissue infections leading to the development of osteomyelitis and sepsis:

- Panteli and Giannoudis (2016) reported that osteomyelitis, once more than 6 weeks old, is a condition that is managed, not cured, as it will most likely recur after surgery with recurrence rates of 20-30%. Therefore, it needs to be prevented. Immunosuppression negatively affects the physiology of the disease, and the risk of recurrence is increased in immunosuppressed individuals. Generally, the incidence of chronic osteomyelitis following contiguous spread, typically from a wound causing the osteomyelitis, is increasing.
- Rabadi et al. (2013) found that 11% of veterans with spinal cord injury died of infected pressure ulcers.
- Rennert et al. (2009) reported that up to 32% of full thickness pressure ulcers were associated with osteomyelitis. They also concluded that as soon as a grade 4 ulcer was present, the patient was at high risk of developing osteomyelitis. Septicaemia was an underlying or contributing cause in 39.7% of all pressure ulcer-associated deaths.
- Jordan and Lewis (2017) in a retrospective review discussed the high failure and recurrence rates associated with surgery for osteomyelitis in SCI-persons, highlighting the devastating and life-long impact this condition has on a person.
- Bodavula et al. (2015) reported that approximately a third of grade 4 pressure ulcers resulted in osteomyelitis.

Clinical data clearly demonstrate that wounds and pressure ulcers in SCI-persons are associated with high rates of mortality, irreversible morbidity and long-term to permanent implications on quality of life. Approximately a third of grade 4 pressure ulcers result in the development of osteomyelitis, which is considered an incurable condition, particularly in immunocompromised persons. Osteomyelitis can develop at any timepoint after infection reaches the bone and treatment of the grade 4 pressure ulcer is therefore of urgency. The use of treatments known not to be effective will therefore expose the participant to a very considerable risk. Based on these observations, the inclusion of a placebo control would be unethical. Similarly, a differential study design would also be unethical, as the risk of developing osteomyelitis whilst on a placebo would be substantial.

## Choice of comparator

ICH guidance states “*it is no more ethically acceptable to randomize deliberately to subeffective control therapy than it is to randomize to placebo*”. It must consequently be a requirement that any comparator has demonstrated effectiveness.

At the time of designing the study, among others, the following was known:

- NICE (2014) in their official guidance for the prevention and management of pressure ulcer advised that systemic antibiotics, dressings with antimicrobials and NPWT (negative pressure wound therapy) should not be used for treating pressure ulcers. The guidance did not provide a recommended treatment for infected pressure ulcers. (These NICE guidelines are regularly reviewed in case of changes in the field, but no new updates have been issued since).
- FDA (2016) concluded, following an extensive review of clinical guidance policies established by American medical associations and clinical publications, that dressings containing antibiotics or antiseptics are ineffective in treating wound infections and in supporting wound healing.
- NICE (2016) concluded, following a review of clinical publications on the treatments for chronic wounds, that there are no data to support the use of antimicrobials for treating chronic wounds.
- Westby et al. (2017) in a Cochrane review performed a meta-analysis of data from 39 studies evaluating 21 dressings and topical agents for use on pressure ulcers. They found that the evidence is sparse and of low or very low certainty (due mainly to risk of bias and imprecision). Consequently, they were unable to determine which dressings or topical agents are the most likely to have a positive impact on pressure ulcers, and they concluded that it was unclear whether the treatments examined are more effective than gauze wetted with saline.

These findings conclusively demonstrate that no effective gold standard with a standardised treatment regimen is available. On the contrary, official guidance directly advises against the use of the most commonly used treatments, i.e. antimicrobials, in pressure ulcers.

## MPPT and wound healing

At the time of designing the study, a number of studies had already compared MPPT (S3: MPPT, MoA, evidence, use and telemedicine) to routinely used standard care approaches for infected wounds and had consistently found highly superior efficacy:

- A preclinical study (Bilyayeva et al. 2014) performed in a rat wound healing model had found that MPPT resulted in an infection-free and healing wound 60% quicker than a topical antibiotic (gentamicin) group and untreated controls. The study confirmed that MPPT is not antimicrobial, that it promotes increased migration of neutrophils, macrophages and lymphocytes to the wound and a more rapid progression from the inflammatory to the proliferative wound healing phase.
- A randomised comparative clinical study with 266 patients (Bilyayeva et al. 2017), covering a range of wound types, had found that the use of MPPT resulted in an infection-free and healing wound following daily application for 3 days, whereas a topical antibiotic (gentamicin) required 7 days and an antiseptic (iodine) 8 days, i.e. a 60% reduction in time by MPPT. Overall MPPT reduced the number of hospitalisation days by 31% compared to gentamicin and by 39% compared to iodine. The study included different subgroups, i.e. acute wounds (abscesses, carbuncles, infected surgical wounds), diabetic foot ulcers, venous leg ulcers and burns. It showed that the time to a non-infected, healing wound was independent of wound type, whereas the reduction in number of hospitalisation days appeared to depend upon the underlying disease process. The reduction in hospitalisation days by MPPT compared to gentamicin was 41% for acute wounds; 31% for diabetic foot ulcers; and 19% for venous leg ulcers; and compared to iodine 44% for acute wounds; 51% for diabetic foot ulcers; and 36% for venous leg ulcers. The effects of the topical antibiotic and the antiseptic were in line with generally published data.
- Ryan (2017) conducted a clinical case-series at Bristol University Hospital, which included nine acute dehisced surgical wounds and one non-healing category 4 pressure ulcer. The grade 4 pressure ulcer had unsuccessfully been treated with a variety of approaches for 4 weeks before MPPT. In all wounds, the use of MPPT led to an infection-free healing wound in 3-5 days and all wounds that could be followed were confirmed to reach closure. Local standard-of-care was 1 week with UrgoClean followed by 2 or more weeks with NPWT to reach the same state of healing as was achieved in 3-5 days with MPPT, i.e. a reduction of 81%.
- A number of cases had shown that MPPT was able to treat a wide variety of wounds (in-house data).

The level of improvement with MPPT compared to antibiotics, antiseptics and NPWT, which are the most used standard care approaches, were in the order of 60-80% reduction in the duration of time required to reach a non-infected, healing wound. In relation to the present study, this reduction is important because a healing non-infected wound will not cause osteomyelitis. Furthermore, all wounds had responded to MPPT-treatment, including wounds that had failed to respond to different types of standard care. This treatment effect of MPPT held against the lack of efficacy of standard care, pointed towards the fact that it would be unacceptable, according to ICH guidance, to include a placebo control and/or a comparator and that it would be required to rely on external controls for evaluating efficacy.

## Choice of study design

ICH (2000) state:

“*In cases where an available treatment is known to prevent serious harm, such as death or irreversible morbidity in the study population, it is generally inappropriate to use a placebo control.*“

“*Where there is therapy known to be effective in preventing death or irreversible morbidity, it is no more ethically acceptable to randomize deliberately to subeffective control therapy than it is to randomize to placebo.*”

The analysis conclusively demonstrated that wounds and pressure ulcers are associated with death and irreversible morbidity in SCI-persons, which means that the conditions of the ICH (2000) guidance were fulfilled in relation to this clinical evaluation. The review had found that standard care had not been shown to be more effective than placebo and that the probability of MPPT having a significant treatment effect on the wounds and pressure ulcers was high. It would therefore be unethical to include a placebo and a comparator group as this would expose participants to an avoidable risk of death and irreversible morbidity.

It had increasingly become the norm, when designing clinical evaluations for diseases and conditions that expose participants to mortality and irreversible morbidity, to use a single-arm design and to rely on external controls for comparison. Oxnard et al. (2016) provided an example from oncology and a similar approach would be suitable for the present study. It would therefore, in patients’ interest, be more acceptable to use a single-arm design, in which all participants were given MPPT.

Wounds and ulcers in SCI is an orphan indication, and this means low availability of patients to recruit from. Furthermore, patient variability is high, making it difficult to obtain homogenous groups suitable for statistical analysis or alternatively requiring large group sizes. Based on these considerations, the study was designed as a non-interventional, observational, post-market surveillance study. This design should lead to the collection of real-world data. The study would include all wounds and pressure ulcers that presented themselves during the inclusion period, irrespectively of age, location, and prior treatment. The study would therefore include both infected and non-infected wounds and acute and chronic wounds. This would also allow the use of a differential analysis approach as all chronic wounds as well as a proportion of acute wounds would have been exposed to prior treatment with standard care, and could therefore act as their own controls.

## Post-study analysis

Following study performance and data analysis the choice of design was reevaluated.

The single-arm study design has become increasingly common practice as described in the white paper by the Clinical Research Expert Group reviewing innovation in clinical trial design on behalf of the European Federation of Pharmaceutical Industries and Associations (EFPIA):

“*Single arm studies have been often used by sponsors to support the registration of medicinal products in some specific circumstances, i.e. in areas of* ***unmet medical need****, when there is* ***no other approved alternative****, or* ***no consensus on alternate salvage therapy****, or in situations where a* ***placebo*** ***control is not acceptable****. In their 10-year report [45] of experience of Conditional Marketing Authorisations (CMA) published in September 2017, the EMA noted that ‘Most studies (34/58) were randomised multiple arm studies, but* ***just over a third of studies consisted of a single arm****.*” EFPIA (2020).

Furthermore, the medical literature on the treatment of wounds and pressure ulcers in SCI-persons, and on pressure ulcers in the wider population as well, has reconfirmed, that no effective treatments are available, meaning that pressure ulcers constitute an unmet medical need; that there is a lack of consensus on treatment approaches; and that the existing approaches used to manage pressure ulcers are subeffective and fail to prevent high rates of mortality and morbidity:

- Guest et al. (2018) found that 66% of all acute pressure ulcers in community care are infected and that only 15% of these close within the first 12 months.

This shows current care to be subeffective. A wide range of products were used with no consensus on treatment approaches, except that these generally involved the use of antimicrobials.

- EPUAP/NPIAP (2019) guidelines on pressure ulcer treatment do not provide consensus on a recommended approach for treating pressure ulcers, but only suggestions of what can be tried.
- Russel et al. (2020) performed a retrospective study of 35 patients of a mean age of 57.4 years with pressure ulcer-related pelvic osteomyelitis. The responsible pressure ulcers started as grade 3 or 4 and osteomyelitis had developed within a median of 4 months, ranging between 7 weeks and 12 months. Treatment failure, defined as requiring repeat surgery or intravenous antimicrobials, occurred in 71% of patients. 64% of patients had unhealed ulcers, including after repeated surgery, and died within a median time of 2 years, ranging between 7 months and 3.4 years, from first surgery. 36% of all the ulcers healed, but in this group the median survival time was only 7 years, ranging between 2.7 and 11.3 years, from first surgery.

These data show a very high risk of death and irreversible morbidity from pressure ulcers associated with osteomyelitis, despite surgical intervention followed by use of standard care. They also show that osteomyelitis develops very quickly, usually within 4 months, but can be less than 2 months. These findings confirm that the inclusion of a placebo group or a comparator known to be subeffective would have exposed the participant to the risk of death and irreversible morbidity, because any treatment regime would need to be allowed at least 2 months to demonstrate efficacy, and the development of osteomyelitis from a wound starting as a grade 3 or 4 would clearly have constituted such a risk within this time period.

- Kala et al. (2019) contacted infectious disease physicians with a questionnaire focusing on how they managed grade 4 pressure ulcers with possible underlying osteomyelitis. They concluded: “The physicians reported widely divergent diagnostic and treatment approaches. Most of the reported practice is not supported by the available evidence, which is quite limited and of low quality.” This shows that no consensus on therapy exists, a fact that reflects sub-effectiveness of current standard care of ulcers associated with osteomyelitis as well as of the treatment of the osteomyelitis itself.

The implications of these findings are that if a patient develops osteomyelitis, the physician does not have access to established proven procedures. Consequently, if a participant had developed osteomyelitis as a consequence of receiving placebo or a subeffective comparator treatment, this would have placed the participant at an increased risk of death or irreversible morbidity.

- Kriz et al. (2021) and Thietje et al. (2022) reported that 10% to 12% of spinal cord injured die due to their pressure ulcers, but the true number is likely higher as deaths caused by osteomyelitis, where the ulcer was responsible for causing the osteomyelitis in the first place, are usually not recorded as a consequence of the ulcer.

During the study period, two case-reports on MPPT versus a standard care approach were published (O’Sullivan et al. 2020; Sams-Dodd and Sams-Dodd 2020). Both demonstrated a superior clinical effect of MPPT compared to the use of antimicrobials and therefore confirmed the observations above. One case found that the use of the antimicrobial directly on exposed infected bone caused damage to the bone and direct worsening of the patient. These findings support existing NICE guidance to not use antimicrobials for treating pressure ulcers.

The conclusion is that the treatment of pressure ulcers in SCI-persons, and in non-SCI persons as well, represents an *unmet medical need* (see also Verma et al. 2022). The clinical evidence shows that current approaches to managing pressure ulcers are *subeffective*; and that *no consensus* exists on how to treat pressure ulcers. Furthermore, clinical data show that osteomyelitis is a frequent, direct, and rapidly developing consequence of pressure ulcers and that it is associated with a substantial risk of irreversible morbidity and death. It can, and often does, develop in less than two months after first detection of the ulcer. Therefore, when the treatment being evaluated in clinic is known to be substantially more effective than existing approaches, it is *unethical* to include a standard care or placebo group as a control arm, particularly as the comparator will always be given for a minimum of 2 months to establish whether it is effective, and this duration of time is sufficient for osteomyelitis to develop. Each of these 4 factors individually - and together - renders the performance of a randomised, controlled, comparative trial unacceptable once efficacy of a treatment has been demonstrated. This was the case for MPPT, confirming that the choice of a single-arm design was correct.

Choosing a single-arm design does not exclude the use of narrow inclusion criteria and highly controlled study conditions. However, pressure ulcers in SCI-persons is an orphan indication and patient variability is normally high as the age, location, wound characteristics, and prior treatments will vary very substantially from one person to another (Malmivaara et al. 2022). Achieving homogenous groups, e.g. to reduce variability in outcome measures, would be possible, but would also result in a very protracted study duration. The present study already took 5 years to complete and it is questionable whether attaining more homogenous groups would justify a longer study duration, when SCI-persons die from their wounds, when it is already known that existing wound treatments are ineffective, and when the treatment benefits identified are very clear, when compared to existing published data.

The selected study design was a non-interventional, observational, post-market surveillance design, where participants are treated as *per* normal clinical care, i.e. no additional measures or evaluations are included. In theory, this should result in an outcome that reflects what can be expected when implemented in daily clinical use. The British Spinal Injuries Association (SIA), which is a patient organisation, conducted in July 2022 a survey among current and prior users of MPPT in the SCI-community to document their experiences with MPPT (Smith 2023). The survey included 41 respondents and 49 wounds, and the response very closely resembled the findings of this study, i.e. a 100% closure rate for acute and chronic wounds and pressure ulcers and the control of soft tissue infection associated with a draining fistula. The survey therefore confirmed both the findings of the study as well as the value of a non-interventional, observational study design for providing data that will reflect real-world-use of the treatment. The value of this design will extend to economic, social and environmental benefits as the findings were compared to retrospective analysis of patient records, i.e. data that similarly reflect real-world use.

In hindsight, the choice of study design was therefore appropriate to address the study question.

## References

1. Bilyayeva O, Neshta VV, Golub A, Sams-Dodd F. (2014) Effects of SertaSil on wound healing in the rat. J Wound Care. 23(8):410, 412-4, 415-6.
2. Bilyayeva OO, Neshta VV, Golub AA, Sams-Dodd F. Comparative Clinical Study of the Wound Healing Effects of a Novel Micropore Particle Technology: Effects on Wounds, Venous Leg Ulcers, and Diabetic Foot Ulcers. Wounds. 2017; 29(8):1-9.
3. Brommer B, Engel O, Kopp MA, et al. Spinal cord injury-induced immune deficiency syndrome enhances infection susceptibility dependent on lesion level. *Brain*. 2016;139(Pt 3):692-707. doi:10.1093/brain/awv375
4. Bodavula P, Liang SY, Wu J, VanTassell P, Marschall J. Pressure Ulcer-Related Pelvic Osteomyelitis: A Neglected Disease?. *Open Forum Infect Dis*. 2015;2(3):ofv112. Published 2015 Aug 6. doi:10.1093/ofid/ofv112
5. Consortium for Spinal Cord Medicine. Pressure injury prevention and treatment following spinal cord injury: A clinical practice guideline for health care professionals (pp. 1-77). 2000.
6. EFPIA Clinical Trial Design Taskforce (2020) Innovation in Clinical Trial Design: A review of The Clinical Trial Design Landscape. https://www.efpia.eu/media/547507/efpia-position-paper-innovation-in-clinical-trial-design-white-paper.pdf.
7. FDA. FDA executive summary. Classification of wound dressings combined with drugs. Prepared for the Meeting of the General and Plastic Surgery Devices Advisory Panel September 20–21, 2016. FDA website. 2016;38–39. <https://www.fda.gov/media/100005/download>
8. Fuhrer MJ, Garber SL, Rintala DH, Clearman R, Hart KA. Pressure injuries in community-resident persons with spinal cord injury: prevalence and risk factors. Arch Phys Med Rehabil 1993;74(11):1172-1177.
9. Guest JF, Fuller GW, Vowden P, Vowden KR. Cohort study evaluating pressure ulcer management in clinical practice in the UK following initial presentation in the community: costs and outcomes. BMJ Open. 2018 Jul 25;8(7):e021769. doi: 10.1136/bmjopen-2018-021769.
10. Houghton PE, Campbell KE and CPG Panel. Canadian best practice guidelines for the prevention and management of pressure injuries in people with spinal cord injury: A resource handbook for clinicians. Accessed at http://www.onf.org. 2013.
11. ICH E10 (2000) Choice of control group and related issues in clinical trials. ICH harmonised tripartite guideline.
12. Jordan SW, De la Garza M, Lewis VL Jr. Two-stage treatment of ischial pressure ulcers in spinal cord injury patients: Technique and outcomes over 8 years. *J Plast Reconstr Aesthet Surg*. 2017;70(7):959-966. doi:10.1016/j.bjps.2017.01.004
13. Kaka AS, Beekmann SE, Gravely A, Filice GA, Polgreen PM, Johnson JR. Diagnosis and Management of Osteomyelitis Associated With Stage 4 Pressure Ulcers: Report of a Query to the Emerging Infections Network of the Infectious Diseases Society of America. Open Forum Infect Dis. 2019;6(11):ofz406. Published 2019 Nov 1. doi:10.1093/ofid/ofz406
14. Krause JS. Skin sores after spinal cord injury: relationship to life adjustment. Spinal Cord 1998;36(1):51-56.
15. Kriz, J., Sediva, K., Maly, M., 2021. Causes of death after spinal cord injury in the Czech Republic. Spinal Cord 59, 814–820. <https://doi.org/10.1038/s41393-020-00593-2>
16. Kumar S, Yarmush ML, Dash BC, Hsia HC, Berthiaume F. Impact of Complete Spinal Cord Injury on Healing of Skin Ulcers in Mouse Models. J Neurotrauma. 2018 Mar 15;35(6):815–24.
17. Manring MM, Hawk A, Calhoun JH, Andersen RC. Treatment of war wounds: a historical review. *Clin Orthop Relat Res*. 2009;467(8):2168-2191. doi:10.1007/s11999-009-0738-5
18. Malmivaara A, Zampolini M, Stam H, Gutenbrunner C. Pros and Cons of Randomized Controlled Trials and Benchmarking Controlled Trials in Rehabilitation: An Academic Debate within the European Academy of Rehabilitation Medicine. Journal of Rehabilitation Medicine. 2022 Oct 10;54:jrm00319–jrm00319.
19. Marbourg JM, Bratasz A, Mo X, Popovich PG. Spinal Cord Injury Suppresses Cutaneous Inflammation: Implications for Peripheral Wound Healing. J Neurotrauma. 2017; 34(6):1149-1155.
20. NICE (2014) Pressure ulcers: prevention and management. 2014. NICE, UK.
21. NICE. Chronic wounds: advanced wound dressings and antimicrobial dressings. 2016; <http://nice.org.uk/guidance/esmpb2>.
22. NPIAP. Clinical Practice Guidelines. 2019.
23. Panteli M, Giannoudis PV. Chronic osteomyelitis: what the surgeon needs to know. *EFORT Open Rev*. 2017;1(5):128-135. Published 2017 Mar 13. doi:10.1302/2058-5241.1.000017
24. O'Sullivan O, Hayton L, Findlay-Cooper K, Phillip R. Novel micropore particle technology for spinal cord injury chronic wound healing: a new paradigm? [published online ahead of print, 2020 Aug 4].
25. Oxnard GR, Wilcox KH, Gonen M, Polotsky M, Hirsch BR, Schwartz LH. Response Rate as a Regulatory End Point in Single-Arm Studies of Advanced Solid Tumors. *JAMA Oncol*. 2016;2(6):772-779. doi:10.1001/jamaoncol.2015.6315
26. Rabadi MH, Mayanna SK, Vincent AS. Predictors of mortality in veterans with traumatic spinal cord injury. Spinal Cord. 2013 Oct;51(10):784–8.
27. Rennert R, Golinko M, Yan A, Flattau A, Tomic-Canic M, Brem H. Developing and evaluating outcomes of an evidence-based protocol for the treatment of osteomyelitis in Stage IV pressure ulcers: a literature and wound electronic medical record database review. Ostomy Wound Manage. 2009;55(3):42-53.
28. Riegger T, Conrad S, Schluesener HJ, et al. Immune depression syndrome following human spinal cord injury (SCI): a pilot study. Neuroscience. 2009;158(3):1194‐1199. doi:10.1016/j.neuroscience.2008.08.021
29. Russell CD, Tsang SJ, Simpson AHRW, Sutherland RK. Outcomes, Microbiology and Antimicrobial Usage in Pressure Ulcer-Related Pelvic Osteomyelitis: Messages for Clinical Practice. J Bone Jt Infect. 2020;5(2):67-75. Published 2020 Mar 26. doi:10.7150/jbji.41779
30. Ryan E. The use of a micropore particle technology in the treatment of acute wounds. J Wound Care. 2017; 26(7): 404-413.
31. Sams-Dodd J, Sams-Dodd F. Micropore Particle Technology Promotes Wound Healing, Whereas Polyhexamethylene Biguanide Causes Tissue Degeneration: A Case Report. Wounds. 2020;32(3):E6-E10.
32. Schwab JM, Zhang Y, Kopp MA, Brommer B, Popovich PG. The paradox of chronic neuroinflammation, systemic immune suppression, autoimmunity after traumatic chronic spinal cord injury. Exp Neurol. 2014; 258:121-129.
33. Thietje, R., Kowald, B., Böthig, R., Schulz, A.P., Northmann, M., Rau, Y., Hirschfeld, S., 2022. Long-Term Survival and Causes of Death in Patients below the Age of 60 with Traumatic Spinal Cord Injury in Germany. Journal of Clinical Medicine 11, 26. https://doi.org/10.3390/jcm11010026
34. Verma KD, Lewis F, Mejia M, Chalasani M, Marcus KA. Food and Drug Administration perspective: Advancing product development for non-healing chronic wounds. Wound Repair Regen. 2022 May;30(3):299–302.
35. Westby MJ, Dumville JC, Soares MO, Stubbs N, Norman G. Dressings and topical agents for treating pressure ulcers. Cochrane Database Syst Rev. 2017;6(6):CD011947. Published 2017 Jun 22. doi:10.1002/14651858.CD011947.pub2
