# Supplementary material for: Stable closure of acute and chronic wounds and pressure ulcers and control of draining fistulas from osteomyelitis in persons with spinal cord injuries: non-interventional study of MPPT passive immunotherapy delivered via telemedicine in community care
Source: Front Med (Lausanne). 2024 Jan 5;10:1279100. doi: 10.3389/fmed.2023.1279100 (PMC10797031; doi:10.3389/fmed.2023.1279100)
Supplement: Supplementary file 6 [file Data_Sheet_6.docx]

# S6: Study design, controls, outcome (safety, efficacy, cost), cost comparisons and sustainability

## Study design and outcome measures

The aim of the study was to determine the ability of MPPT to treat wounds and wound infections in persons with spinal cord injury (SCI). An analysis of ICH (2000) guidance, which covers the criteria for including placebo and comparator control groups in clinical trials, found that the inclusion of a placebo control and a comparator would be unethical due to increased risk of death and irreversible morbidity to participants and because it was known that MPPT is effective in treating wound infections (*S5 - Clinical trial design for pressure ulcer treatment in SCI-persons*). Furthermore, spinal cord injury is an orphan indication, which means that the patient population of spinal cord injured with wounds and pressure ulcers suitable for inclusion is limited, and there is a high degree of patient variability, making it difficult to achieve homogenous groups (Malmivaara et al. 2022).

Based on these considerations and to focus on real-world-data, it was decided to use a non-interventional, observational, post-market surveillance design, where participants are treated in accordance with routine clinical care. Study inclusion criteria were wide, and by including everyone appearing during the inclusion period, i.e. no selection beyond compliance, it would be reasonable to assume that the study would reflect the true patient population. Participants could have more than one wound as MPPT is a topical product that only affects healing of the wound to which it is applied, i.e. nothing is released from the particles or absorbed by the body.

Treatment was delivered in community care by telemedicine. Participants, family or carers were responsible for daily hands-on dressing changes. They were asked to send pictures daily of the inside of the used dressing, the wound before and after wash, the wound with MPPT applied, and the wound with the fresh secondary dressing, if one was used. The pictures together with any comments were sent by e-mail to a wound expert, who would analyse them and respond via e-mail how to proceed at the next dressing change and would answer any questions.

Clinically relevant endpoints were chosen as outcome measures, i.e. no surrogate markers or measures which would not be part of normal care. A confounding factor was, that some wounds would be secondary conditions to a primary focus of infection, e.g. osteomyelitis or an anal fistula, meaning that they were in fact draining fistulas, between that condition and the skin, disguised as severe wounds. As such fistulas cannot close without removing the primary focus of infection, i.e. they are not “genuine” wounds, the clinical picture of such draining fistulas can be very similar to a “genuine” wound, and the resultant difficulty in diagnosing them meant, that it was not necessarily known at start of treatment whether the wound was a wound or a draining fistula, requiring surgery for the underlying primary condition before it could achieve closure. All wounds that did not heal were therefore followed until the cause for non-closure could be reasonably established based on presentation, clinical history and available evidence such as imaging and biopsies. This meant that some participants would need to be followed for prolonged periods of time. As all wounds presenting during the inclusion period were included to avoid bias, the inclusion of a new wound could extend the study period by 1 to 2 years. It was therefore necessary to consider the length of the recruitment period to avoid the study continuing for years and thereby considerably postponing the communication of the findings.

| **Increase** | | **Decrease** | |
| --- | --- | --- | --- |
|  | Autolytic debridement |  | Odour |
|  | Granulation |  | Cellulitis |
|  | Epithelialisation |  | Inflammation |
|  | Abscess evacuation |  | Nodulation |

Table 1. Features used as markers of improvement in wounds acting as draining fistulas. The use of MPPT on a draining fistula was considered to have improved the state of the wound, if the level of necrotic tissue and slough in general, cellulitis, number of disseminated abscesses, inflammation and smell was unambiguously reduced and if granulation and epithelialisation clearly occurred. Overall, such improvement can be summed up as considerable reduction in the generalised soft tissue infection and improved control of the draining fistula – always with reference to and in accordance with the state of development and progress of the primary focus of infection, e.g. the osteomyelitis.

Based on these considerations, the outcome measures and primary endpoints were:

- Safety:
  Observations of adverse events, wound irritation, allergy, bleeding, and visual changes in bone structure.
- Efficacy in acute and chronic wounds and pressure ulcers:
  Number of days to full, stable closure.
- Efficacy in wounds acting as draining fistulas:
  Control of soft tissue infection and the formation of a controlled draining fistula, while taking age/duration and changes in the underlying primary infection into consideration. Markers used to show improvement are shown in Table 1.
- Costs:
  Number of bottles of MPPT with a set unit cost of £119.84.
  Number of days performing picture analysis and providing guidance. The treatment was delivered by telemedicine with the participants, family, or carers responsible for the daily hands-on dressing changes. The unit cost of a wound evaluation was chosen as equal to the high-end costs of a specialty nurse visiting a patient in community care, i.e. £65 per evaluation. The cost calculations for MPPT would thereby be the same, independent of whether treatment is provided by telemedicine or by a visiting nurse.

## External controls

Single-arm studies necessarily lack the inclusion of comparators and the evaluation of the level of improvement relative to existing approaches therefore needs to build on either differential treatment or comparison to published data (FDA 2013; Davi et al. 2020; Rahman et al. 2021; Yap et al. 2022). Each approach has advantages and disadvantages:

- Differential response to treatment if the patient has previously been treated with other approaches, i.e. Treatment A followed by Treatment B.

The advantage is, that this eliminates individual variability, but it usually also means that the condition is chronic or has become chronic during the use of Treatment A, and therefore often has become more complex for Treatment B to treat, particularly in immunocompromised patients, in whom an infection will easily spread and where time is a highly critical factor. As the single-arm study design meant that MPPT would always be the last of the treatments, i.e. Treatment B, MPPT would always be at a disadvantage.

- Comparison to published data.

This requires that the conditions of the study are reasonably similar to the published data and that the degree of improvement in outcome is substantially different to the published data to identify true changes, e.g. a change of only a few percentage points does not necessarily reflect a true difference.

|  | **Primary analysis method** | |
| --- | --- | --- |
| **Wound category** | **Efficacy** | **Costs** |
| Acute wounds | Guest et al. (2018) | Guest et al. (2018) |
| Chronic wounds | Differential | Bennett et al. (2004) |
| Draining fistula | Differential | Bennett et al. (2004) |

Table 2. Updated approach for analysis of study outcome.

Table 2 outlines the planned approach for evaluating the outcome. At study start, the intention was to use the data from Bennett et al. (2004) for comparison of all three wound categories. Bennett et al. (2004) had determined the daily treatment costs of grade 1-4 pressure ulcers with different stages and levels of complications, including with osteomyelitis. The study is based on pressure ulcers in long-term care facilities, but the costing does not include the care-facility costs, i.e. the numbers are excess costs caused by the pressure ulcer, which makes them comparable to a community care situation with participants living at home. During the study period, however, Guest et al. (2018) published detailed data on the outcome of standard care for treating different grades of acute pressure ulcers in UK community care, thereby providing data allowing a direct and detailed comparison to the treatment of acute pressure ulcers with MPPT. Guest et al. (2018) includes 9% wheelchair users of whom only a proportion would be persons with SCI, and the healing rates in their study are likely to be slightly faster than if it had included only persons with SCI. This has not been corrected for. With respect to chronic wounds and draining fistulas in the study, they will all have been treated with standard care prior to inclusion, which will allow differential analysis, and Bennett et al. (2004) provide suitable data for their cost-analysis.

All costs were, where indicated, adjusted to 2022 prices using an inflation factor of 1.1385%. (Please, see *S1: Costs of Wounds* on the calculation of the inflation factor and its use.) Year 2022 is used as reference point in the analysis as inflation was relatively low and stable between 2012 and 2022, whereas it increased significantly in 2022 and demonstrated significant fluctuations. Therefore, by using 2022 as the reference point, the calculations are more reliable.

## Study outcome

### Study details

The study was conducted between 2017 and 2021, including participants from 2017 to 2020, and included a total of 44 wounds (Table 3), which fell into 3 groups: 1) 21 acute wounds less than 6 weeks old; 2) 10 chronic wounds 6 weeks or older without an underlying infective condition; and 3) 13 draining fistulas, i.e. wounds with an underlying infective condition, i.e. osteomyelitis or an anal fistula. Essentially all were pressure ulcers. All chronic wounds and draining fistulas had previously been treated with a wide range of products, including different types of antimicrobials, without achieving closure or a controlled state, respectively. At the time of inclusion in the study, they were still being treated with standard care. For details, please see *S8: Analysis of chronic grade 3 and 4 wounds* and *S9: Analysis of wounds draining from underlying primary focus of infection*.

A total of 28 persons with spinal cord injury were recruited into the study. Two recruits were forced to withdraw after a few days due to external circumstances despite their wounds responding well to MPPT and one recruit was excluded due to severe non-compliance. The mean age of the remaining 25 participants was 54.4 ± 14.2 years, ranging from 35 to 84 years. One participant withdrew prematurely due to personal circumstances, but data are included. The gender ratio was approx. 2:3 (female:male) and the distribution of para vs. tetraplegic was 1:1. There were an equal number and level of severity of acute wounds on the lower legs and feet compared to acute wounds in the pelvic area. Of the chronic wounds and the draining fistulas, i.e. those with osteomyelitis, the distribution was skewed with only 2 on the ankle and 21 wounds in the pelvic region. No correlation between healing and age, gender or level of injury were seen.

### Safety of MPPT

In no participant were signs of wound irritation, allergy or bleeding observed in response to MPPT. The study included several hypersensitive individuals but no allergic reactions to MPPT were seen even after prolonged use. MPPT was used daily on the same area for more than 6 months by 11 participants and of these for more than 12 months by 5 patients; the latter included direct daily application onto bone with chronic osteomyelitis and with one participant on anticoagulant therapy. No adverse effects on skin, muscle, tendon or bone were observed and MPPT was not, including in hyperallergic participants, associated with any allergies, irritation, or bleeding. MPPT was also used on inflamed, irritated and macerated skin around the wound, e.g. caused by tape allergy or exudate, and MPPT was consistently able to reduce these conditions.

Skin structure is affected by racial origin (Naik and Farrukh 2022) and MPPT was found safe and effective across different skin structures.

### Efficacy and cost

| **Category** | **Number** | **Closure** | **Wounds showing improvement compared to Start of MPPT** |
| --- | --- | --- | --- |
| Acute wounds | 21 | 100% | 100% |
| Chronic wounds | 10 | 100% | 100% |
| Draining fistula | 13 | NA | 100% |

Table 3. Clinical efficacy with relation to healing and improvement rates. NA: non applicable.

The clinical results of the study are summarised in Table 3:

- All acute and chronic wounds, independently of grade, reached stable closure and all draining fistulas improved with the use of MPPT.
- All chronic wounds and draining fistulas were receiving standard care at inclusion in the study and all improved following the change to MPPT. Therefore, the differential response demonstrated a clear treatment effect of MPPT.

There was therefore, for all treatment groups, a clear response to the use of MPPT, which is in line with prior studies (Bilyayeva et al. 2017; Ryan 2017; Sams-Dodd and Sams-Dodd 2018; O’Sullivan et al. 2020; Sams-Dodd and Sams-Dodd 2020). The efficacy of MPPT was not simply due to the change in wound dressing procedures, e.g. the use of tap-water and allowing air to the wound surface, because in a couple of cases the MPPT had in error been damaged (by exposure to heat) and this clearly resulted in loss of efficacy until new MPPT had been supplied.

|  | **N** | **Age**  **median (range)** | **Outcome** | **Days to closure**  **median (range)** | **Costs MPPT**  **median (range)** | **Total Costs**  **median (range)** |
| --- | --- | --- | --- | --- | --- | --- |
| **Acute** | | | | | | |
| **Grade 1-2** | 10 | <7 days | 100%  closure | 7 days | £120  (£120 to £120) | £217  (£120 to £770) |
| **Grade 3-4** | 11 | 7 days  (<7 to 21 days) | 100%  closure | 47.5 days  (25 to 63 days) | £240  (£120 to £599) | £1420  (£184 to £4,455) |
| **Chronic** | | | | | | |
| **Grade 3** | 6 | 19.5 months  (3 to 144 months) | 100%  closure | 75 days  (23 to 243 days) | £539  (£240 to £1,438) | £3,584  (£1,735 to £8,003) |
| **Grade 4** | 4 | 4 months  (2 to 132 months) | 100%  closure | 183 days  (72 to 313 days) | £1,618  (£360 to £3,955) | £7,966  (£3,220 to £9,870) |
| **Underlying primary focus of infection** | | | | | | |
| **Draining fistula** | 13 | 18 months  (2 to 62 months) | Clear improvement | **Month 1** | £1,678/month  (£306 - £12,720) | £3,638/month  (£2,256 - £14,670) |
|  |  |  |  | **Month 12** | £495/month  (£99 - £4,670) | £560/month  (£164 – £4,735) |

Table 4. Outcome of study for primary efficacy and cost endpoints. All acute and chronic wounds closed. For cost calculations, the cost of one MPPT bottle was £119.84 and one evaluation £65/day. Number of evaluations for chronic wounds were calculated as daily for the first month and then every third day until closure. For Draining Fistulas, the costs include daily evaluations the first month, every second day the second month, every third day the third month and thereafter once monthly. Family and carers were responsible for hands-on dressing changes and rapidly learned to manage the wound independently.

| **Table 5A** | **Acute grade 1-2** | | **Acute grade 3-4** | | **Chronic grade 3** | | **Chronic grade 4** | |
| --- | --- | --- | --- | --- | --- | --- | --- | --- |
|  | **Mean ± SD** | **Median**  **(Q1; Q3)** | **Mean ± SD** | **Median**  **(Q1; Q3)** | **Mean ± SD** | **Median**  **(Q1; Q3)** | **Mean ± SD** | **Median**  **(Q1; Q3)** |
| **Days to closure** | 21 ± 0 | 21  (21; 21) | 49 ± 13 | 48  (38; 58) | 98 ± 85 | 75  (35; 133) | 188 ± 102 | 183  (133; 238) |
| **MPPT Bottles used** | 1.0 ± 0 | 1  (1; 1) | 2 ± 1 | 2  (1; 3) | 5 ± 4 | 5  (3; 6) | 16 ± 13 | 14  (8; 22) |
| **Wound evaluations** | 2.4 ± 2.8 | 1.5  (1; 2.8) | 28 ± 20 | 20  (15; 38) | 52 ± 29 | 45  (32; 64) | 83 ± 34 | 81  (64; 99) |
| **MPPT costs (£)** | 120 ± 0 | 120  (120; 120) | 252 ± 152 | 240  (120; 300) | 620 ± 445 | 539  (300; 689) | 1,887 ± 1,564 | 1,618  (899; 2,607) |
| **Evaluation costs (£)** | 156 ± 184 | 98  (65; 179) | 1,788 ± 1,297 | 1,300  (943; 2,470) | 3,380 ± 1,891 | 2,925  (2,053; 4,187) | 5,368 ± 2,200 | 5,265  (4,176; 6,457) |
| **Total costs (£)** | 276 ± 184 | 217  (185; 299) | 2,039 ± 1,402 | 1,420  (1,062; 2,889) | 3,999 ± 1,890 | 3,584  (2,412; 4,696) | 7,255 ± 2,999 | 7,966  (5,884; 9,338) |

| **Table 5B** | **Percentage MPPT used monthly relative to first month from start of treatment.** | | | | | | | | | | | |
| --- | --- | --- | --- | --- | --- | --- | --- | --- | --- | --- | --- | --- |
|  | **1** | **2** | **3** | **4** | **5** | **6** | **7** | **8** | **9** | **10** | **11** | **12** |
| **Mean ± SD** | 100 ± 0 | 68 ± 28 | 59 ± 38 | 57 ± 50 | 54 ± 42 | 47 ± 31 | 45 ± 28 | 41 ± 21 | 39 ± 17 | 42 ± 20 | 42 ± 17 | 40 ± 13 |
| **Median**  **(Q1;Q3)** | 100  (100; 100) | 69  (41; 88) | 42  (33; 75) | 36  (30; 63) | 36  (27; 71) | 36  (29; 56) | 36  (29; 54) | 36  (27; 49) | 37  (30; 49) | 37  (30; 55) | 37  (30; 57) | 37  (30; 47) |

Table 5. Mean ± standard deviation and median with 25% and 75% quantiles. Table A: Outcome parameters for acute and chronic wounds, corresponding to Fig. 1. For acute grade 1-2 wounds, most participants did not report an exact closing date, because the wound was uncomplicated and because they generally had a severe wound, they were more concerned about. When asked, they had therefore forgotten the exact closure date. The only reported closure date was therefore 21 days for 1 wound, which therefore is the only one included. However, based on general correspondence, it could be observed that the period for resolving a grade 1 ulcer was 1-5 days and for grade 2 around 1-2 weeks. Table B: Monthly use of MPPT for treating the draining fistulas for the first year, corresponding to Fig. 2. For health economic calculations, mean values were used to align with the data reported by Guest et al. (Guest et al., 2018) and Bennet et al. (Bennett et al., 2004).

Figure 1. Median time to closure and median cost per wound (costs of MPPT and total costs) to reach closure for acute (less than 6 weeks old) and chronic wounds (6 weeks or older) in the study. Both parameters show an exponential increase as the severity of the wounds increases.

#### Acute and chronic wounds

All acute and chronic wounds closed following treatment with MPPT. Table 4 and 5 and Fig. 1 show how days to closure and costs increased with increasing wound grade and wound age at start of MPPT treatment. It is worth noting, that the median age at start of the chronic grade 3 and 4 wounds were 19.5 and 4 months, respectively. This is consistent with grade 4 wounds having a considerably higher risk of causing osteomyelitis (Rennert et al. 2009; Russell et al. 2020) as the wound has penetrated all anatomical barriers and escaped the specialised immune response of the skin, which could have limited the spread of the infection (for details, please see *S4: SCI, immune dysfunction, osteomyelitis and aim of MPPT*). The graph in Fig. 1 clearly highlights the need for initiating treatment as early as possible. See *S8: Analysis of chronic grade 3 and 4 wounds* for a presentation of the individual chronic wounds.

#### Wounds acting as draining fistulas

In all wounds acting as draining fistulas (see *S9: Analysis of wounds draining from underlying primary focus of infection*), MPPT was able to control and reduce soft tissue infection and promote tissue regeneration leading to reduced wound volume. The wounds would typically present in two different categories: One (see wounds 37, 38, 40) would appear as a very small opening in the skin categorically refusing to close. This would be an indication of osteomyelitis causing the wound from within and the MPPT treatment ensured a well-controlled development of this draining fistula into a draining canal free of infection and associated with a low risk of septicaemia. The other category would present as wide open wounds, with necrosis, sinuses or gorge formation in the wound bed, and usually with high levels of exudate. Whether the wound had caused the osteomyelitis or was originally caused by the osteomyelitis was, at this stage, not possible to determine and did not impact the treatment approach. In these wounds, MPPT treatment brought the infection under control, stopped the progression soft tissue necrosis, instigated tissue regeneration, and created a well-controlled draining canal with minimal dissemination of infection into the surrounding soft tissue thereby lowering the risk of septicaemia substantially.

In five cases, extensive cellulitis was present in a wide area around the wound opening. It was removed by MPPT and the skin restored to its natural structure. The typical clinical signs of skin infection were a broad band of dark-red/purple skin of an unmistakably stiff texture resembling cardboard, and in three cases the skin infection was further exacerbated by large areas of pronounced nodulous, thickened skin.

During the study, several patients required antibiotic treatment for non-wound related conditions such as UTIs, GI infections, toothache, or for flare-ups of their osteomyelitis. This generally caused wound healing to slow down or, in a few instances, to stall while the antibiotics were taken. Generally, following a course of antibiotics, the infective debris seemed to return stronger, and, in order to maintain the draining canal free of infection, slightly more MPPT was in some cases needed.

Distinguishing features of possible diagnostic value of wounds with osteomyelitis or an anal fistula were the presence of air bubbles/foam on the wound surface, the persistent presence of gorges in the wound bed, and resistance to stable closure when treated with MPPT.

Figure 2. Monthly amount of MPPT, expressed as percentage relative to the first month of treatment, used for treating 12 wounds acting as draining fistulas caused by an underlying primary focus of infection, e.g. osteomyelitis or anal fistula. One case (#40) was excluded because the associated fistula had not yet broken down the soft tissue into a festering wound, as it had been surgically debrided and unsuccessfully attempted closed by primary closure. This obscured the presence of the fistula somewhat and the case was therefore not comparable to others. Draining fistulas show a great variety in presentation, but a common factor is usually a widely disseminated infiltration of the soft tissue in need of control. In cases, where treatment was stopped early, the last level of monthly use was extended until end of the year, i.e. no further decreases in use were included in the calculations, meaning that further reductions would have been possible. Termination of treatment was not due to lack of effect, but external factors.

Figure 2 and Table 5B show the amount of MPPT used during the first 12 months of treatment as percentage of the first month of treatment. The wounds were generally festering and out of control at start and Fig. 2 shows that it generally took 2.5 months to control the wound after which point, the treatment was primarily maintenance. As shown in Table 4, the monthly costs of management fell by 85% from the 1’st to the 12’th month as the monthly amount of MPPT needed fell by 63% and the need for assistance with the management of the wound by an expert fell, as well.

The treatment is unavoidably symptomatic, since MPPT cannot treat an underlying primary focus of infection outside the realm of the soft tissue, but controlling the soft tissue infection and the toxins drained from the focus of infection considerably reduces toxaemia and the risk of sepsis (see *S4: SCI, immune dysfunction, osteomyelitis and aim of MPPT*). Also, participants were not required to remain on bed rest and they were able to assume responsibility for the daily dressing changes, two aspects which strongly supported independence and self-care.

## Risk


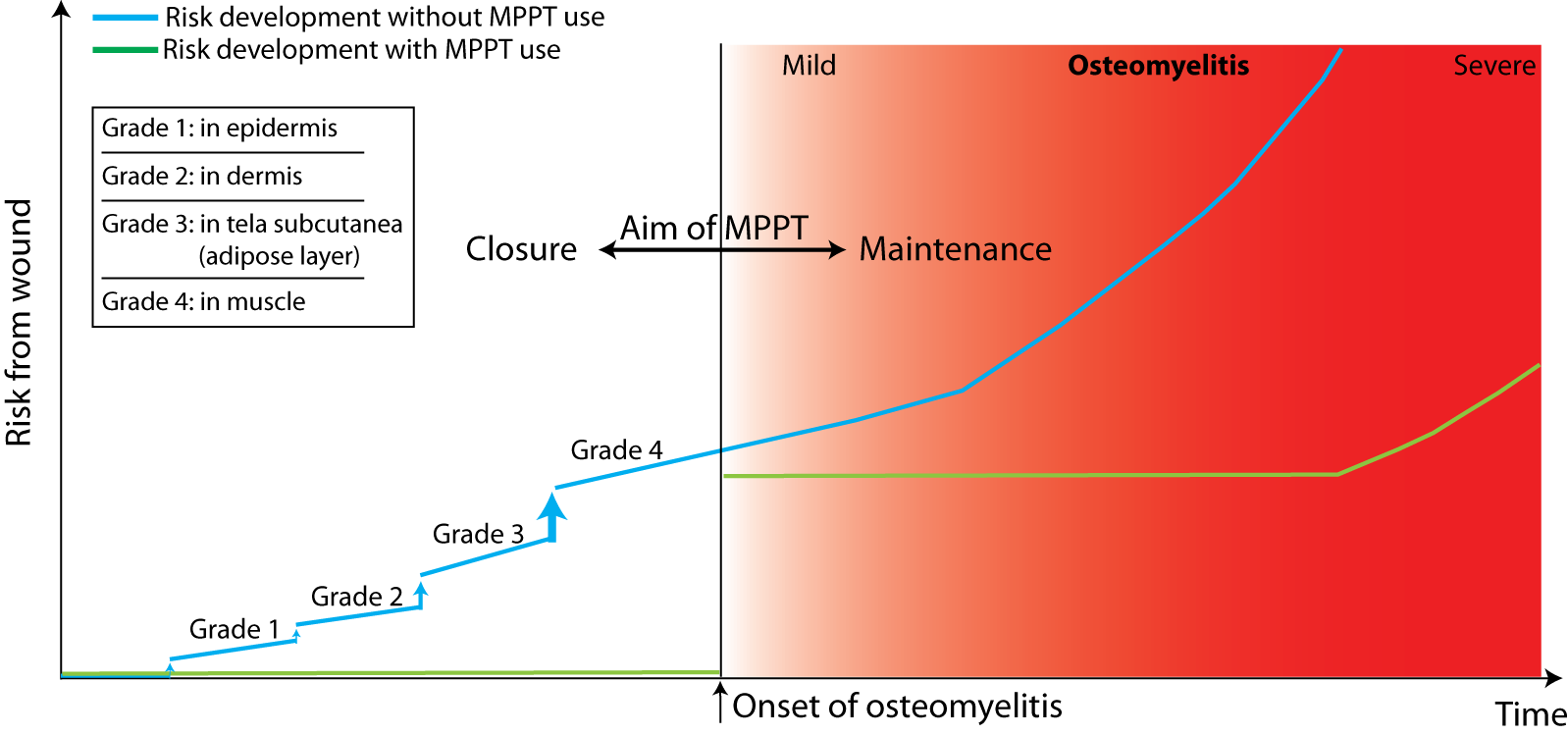


Figure 3. Risk to patient from pressure ulcers and wounds, and benefits of MPPT.

The impact of wounds and pressure ulcers on patient health and the effects of MPPT can be viewed from a risk perspective, where risk factors include spread of infection, toxaemia, septicaemia, development of osteomyelitis, and sepsis. Using risk as the outcome measure, the findings of the study are summarised in Fig. 3. In grade 1-3 wounds, not having penetrated the basal membrane of *tela subcutanea*, the main risk is further deterioration of the wound with associated sepsis originating in the soft tissue (blue line); MPPT is able to close these wounds, thereby removing the risk (green line). In grade 4 wounds, the risk of developing osteomyelitis via contiguous spread increases rapidly as the main anatomical and immunological barriers have been breached, allowing infection to spread relatively unhindered in the tissue. MPPT is able to close these wounds, thereby removing the risk provided that treatment is initiated before osteomyelitis has developed. Once the primary source of infection is not the wound but osteomyelitis or e.g. an anal fistula, MPPT can control the soft tissue infection and can reduce the risk of sepsis originating in the soft tissue, which is constantly bathed in infectious debris from the bone infection, but the risk of sepsis originating in the bone remains. Resolution requires surgery for the primary causative condition. Furthermore, the osteomyelitis will continue to spread and affect an increasing part of the bone and thereby constitute an increasing risk of sepsis. As the infected area of the bone enlarges, part of the debris is generated increasingly further from the draining canal, i.e. the wound. Instead of increasing the distance to travel, the debris may carve new fistulas that resemble wounds on the skin. Each will represent an increased risk of sepsis originating in the soft tissue and will need MPPT to control this.

## Cost analysis

### Cost-analysis: Acute wounds and pressure ulcers

| **Acute wounds** | **Guest et al. (2018)** | | **Study MPPT** | |
| --- | --- | --- | --- | --- |
| **Grade** | **% of cohort** | **Closure** | **% of cohort** | **Closure** |
| 1 | 11% | 100% | 24% | 100% |
| 2 | 7% | 30% | 24% | 100% |
| 3 | 60% | 17% | 48% | 100% |
| 4 | 10% | 15% | 5% | 100% |
| Unstageable | 12% | 9% | 0% |  |

Table 6. Distribution of acute wound grade and healing rates within the first 12 months for Guest et al. (2018, table 9) and the present study.

Guest et al. (2018) analysed in detail the records in the THIN-database of 209 patients with acute pressure ulcers and determined the outcome and mean costs for the first 12 months after presentation to the NHS. Their treatment groups correspond to the acute wounds in the present study, allowing direct comparison. Table 6 compares the results of the present study to Guest et al. (2018) in terms of distribution of wound stages and closure rates. In Guest et al. (2018) the closure rates fell as the wounds increased in severity. In contrast, with MPPT all acute wounds closed, independently of grade and prior treatment. All costs are shown as mean and adjusted to 2022 prices to allow direct comparison.

| **Acute**  **wounds** | **Guest et al. (2018) – Acute wounds – Standard of Care** | | | | | | | | **Study MPPT**  **Acute wounds** | | |
| --- | --- | --- | --- | --- | --- | --- | --- | --- | --- | --- | --- |
|  | **No antimicrobials** | | | | **Antimicrobials** | | | |  |  |  |
| **Grade** | **% of cohort** | **Closure** | **Time**  **months** | **Cost** | **% of cohort** | **Closure** | **Time**  **months** | **Cost** | **Closure** | **Time**  **months** | **Cost** |
| 1 | 82% | 100% | 1.2 | £801 | 18% | 100% | 4.0 | £4,806 | 100% | <1 | £328 |
| 2 | 53% | 57% | 4.9 | £3,801 | 47% | 0% | - | £13,084 | 100% | <1 | £224 |
| 3 | 27% | 23% | 6.6 | £5,219 | 73% | 15% | 8.2 | £9,679 | 100% | 1.8 | £1,884 |
| 4 | 24% | 0% | - | £8,226 | 76% | 20% | 7.3 | £17,610 | 100% | 1.3 | £2,645 |

Table 7. Comparison of study outcome of acute wounds to Guest et al. (2018, Table 9). % of cohort is the distribution of wounds receiving non-antimicrobial vs. antimicrobial treatment in the Guest et al. (2018) study; Closure is percentage of group reaching closure; Time is average number of months to closure for those that closed; Cost is mean costs first year (Guest et al. 2018) which in the case of MPPT also means Cost to closure (Study). Unstageable are not included as they represent a mix of grades. Adjusted to beginning of 2022 prices (*S1: Costs of wounds*).

For each wound grade, Table 7 compares closure rate, mean time to closure for those that achieved closure, and mean cost of treatment of acute wounds treated without and with antimicrobials as standard care in Guest et al. (2018) to acute wounds treated with MPPT. In all groups receiving MPPT, the healing rates are higher, the time to closure shorter and the costs lower, compared to standard care. Furthermore, the differences are substantial, as required when comparing to published data (see above), and they are consistent across groups.

| **Wound types** | **Portion of Cohort** | **Closure rate** | **Time to closure (months)** | **Cost**  **First year** | **Excess cost first year compared to MPPT** | **Potential first year savings with MPPT** |
| --- | --- | --- | --- | --- | --- | --- |
| **MPPT**  **Grade 3 with infection** | 100% | 100% | 1.6 | £1,884 | £0  Unhealed: 0% | - |
| **Standard Care**  **Grade 3 with infection*** | 73% | 15% | 8.2 | £9,679 | £7,795  Unhealed: 85% | 80.5% |
| **Standard Care**  **Grade 3 no infection*** | 27% | 23% | 6.6 | £5,219 | £3,335  Unhealed: 77% | 63.9% |

Table 8. Mean costs of treating acute grade 3 wounds and pressure ulcers in SCI-persons with MPPT compared to standard care by the NHS the first year. *: Guest et al. (2018). Costs have been adjusted to 2022 prices.

Acute grade 3 wounds were the most common type in both Guest et al. (2018) and the present study and will be analysed in more detail. Table 8 compares MPPT to standard care for the first year of treatment using mean costs. The table brings to attention the low closure rate with standard care and the very long duration of treatment required for the 17% (Table 6) of the acute grade 3 pressure ulcers that reached closure within the first year. The implication of such numbers is that the 83% of wounds that did not heal will continue to need treatment the following years, as well. Another consequence is that over time a grade 3 wound tends to deteriorate into a grade 4, which has a high probability of developing of osteomyelitis (see *S4: SCI, immune dysfunction, osteomyelitis and aim of MPPT*), which can happen in as little as 7 weeks and is associated with high risk of death and irreversible morbidity. In contrast, the table shows that MPPT achieved a 100% closure rate with an average time to closure of 1.6 months. The table also compares the total cost of MPPT until closure to the cost of standard care for the first year only. The costs generated with standard care will, of course, continue to accumulate the following years whilst the MPPT costs on average stop after 1.6 months. The higher efficacy of MPPT clearly results in considerably less treatment costs with MPPT versus standard care as shown by the excess cost for the first year alone of standard care relative to MPPT; avoiding these excess costs leads to the substantial potential average savings per wound the first year shown in the last column.


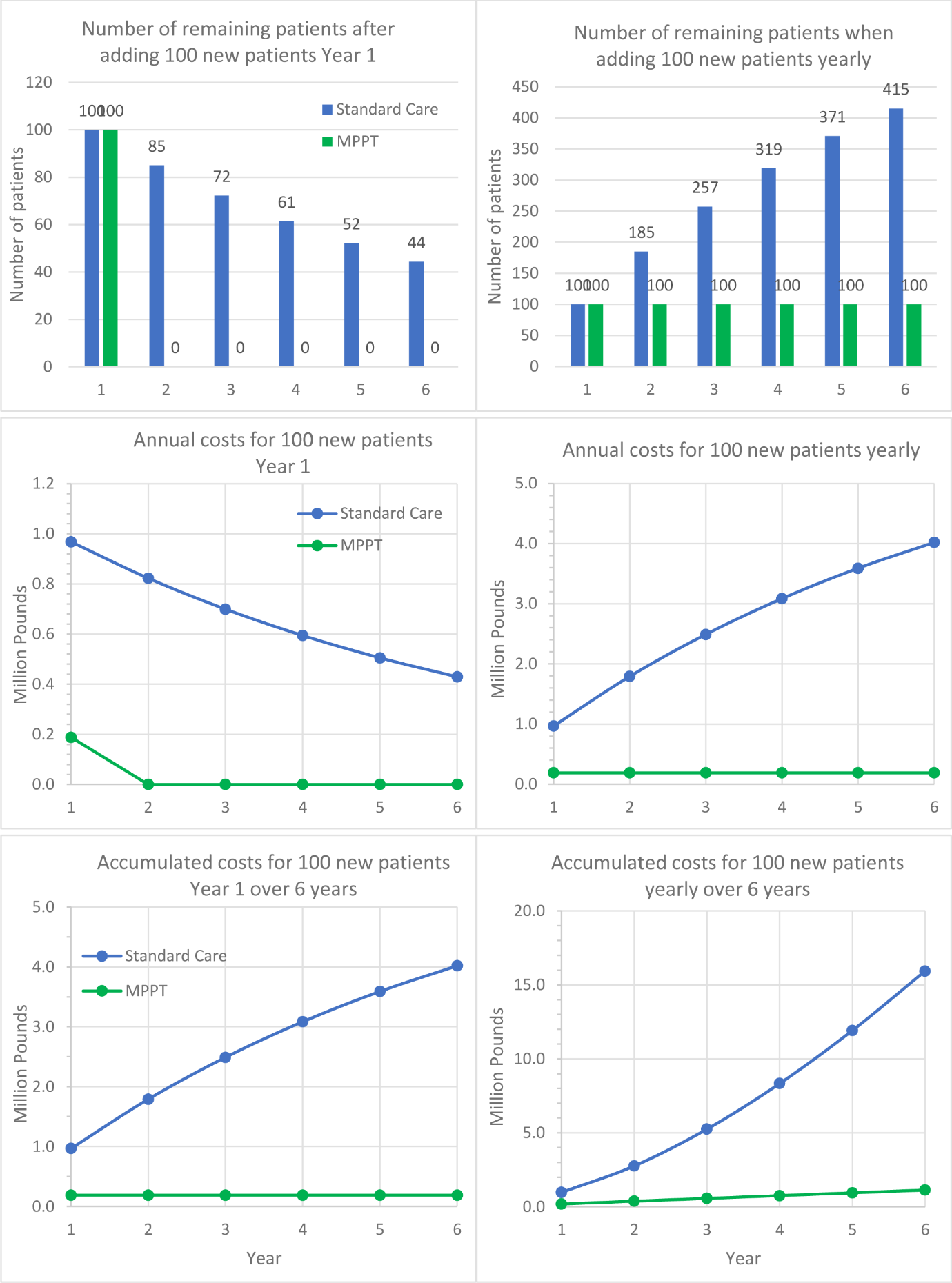
Figure 4. Comparison of MPPT to standard care for treating acute, infected grade 3 wounds and pressure ulcers over a 6-year period. Left column follows a cohort of 100 patients included Year 1 and followed over 5 years. It shows remaining unhealed patients annually, annual cost, and accumulated costs. Right column assumes 100 new patients appear every year and shows the development in unhealed patients, and annual and accumulated costs.

The failure of 83% of acute grade 3 wounds treated with standard care to close within one year from first presentation to the NHS necessarily entails that the costs of the unhealed wounds will continue into the following years. Figure 4 illustrates the implications of the differences in closure rates and costs of treatment. The left column represents a cohort of 100 patients with an acute infected grade 3 pressure ulcer, starting Year 1. The graphs show number of remaining patients, i.e. unhealed, each year during this period (top) and annual (middle) and accumulated (lower) costs over the following 5 years. The data are based on the assumption that the healing rate remains at 15% after the first year, which is unlikely as chronic wounds are more difficult to heal and the healing rate therefore is likely to decrease further. At the end of Year 6, the annual costs are over 0.4 million and the accumulated costs £4 million with 44 patients still remaining to heal. In contrast, at the end of Year 6 with MPPT, the total costs are £188,400, i.e. 100 patients times £1,884, with all patients healed within the first year. The right column in Fig. 4 assumes that 100 new patients appear every year and follow the same healing pattern as shown in the left column. At Year 6, the annual costs are £4 million and still growing and the accumulated costs have now reached £15.9 million and are growing exponentially. In contrast, MPPT closes all wounds within the same year, which means no accumulation will occur. In addition to the economic implications, the importance of time to closure is of paramount importance to the patients, as well. Quicker closure reduces the risk of further deterioration and follow-on conditions, and with standard care bed rest is normally recommended, which means that a person for months to years will be totally isolated and any jobs, educations, social networks etc. will be destroyed, necessarily resulting in severe mental health issues as well as deterioration of their general health due to lack of physical activity.

### Nursing resource-analysis: Acute wounds and pressure ulcers

Guest et al. (2018) determined the mean number of times a nurse visited a patient to perform wound dressing changes during the first 12 months of care for each category of pressure ulcers. For acute grade 3 pressure ulcers, the mean number of visits was 109.14 per wound, corresponding to approx. twice weekly dressing changes. Nursing represents the most significant resource category contributing to the burden of wounds and number of visits is an important comparator because it is not affected by inflation and because availability of nursing staff is a limiting factor in many areas.

| **Acute grade 3** | **Closure rate** | **Time to closure (months)** | **Mean number of evaluations/visits first year** | **Excess visits first year compared to MPPT** | **Potential reduction in visits with MPPT** |
| --- | --- | --- | --- | --- | --- |
| **MPPT** | 100% | 1.6 | 25.3 | 0 Unhealed: 0% | - |
| **Standard care** | 17.4%* | 8.2** | 109.14 | 83.84 Unhealed: 82.6% | 76.8% |

Table 9. Mean number of nurse visits or remote wound evaluations for treating acute grade 3 pressure ulcers with MPPT compared to standard care by the NHS the first year. *: Overall closure rate with standard care (Guest et al. 2018). **: Time to closure of the wounds that closed. (82.6% did not close.)

Table 9 compares the mean number of nurse visits using standard care to the number of remote wound evaluations with MPPT during the first year of care after developing a new grade 3 pressure ulcer. For standard care, it is 109.14 visits per pressure ulcer and, for MPPT, it is 25.3 remote wound evaluations per ulcer. The excess number of events, either visits or wound evaluations, needed with standard care was 83.84 per ulcer. Compared to standard care, MPPT therefore provided a reduction of 76.8% the first year alone in the number of days a healthcare professional had to engage with the patient regarding the care of the wound.


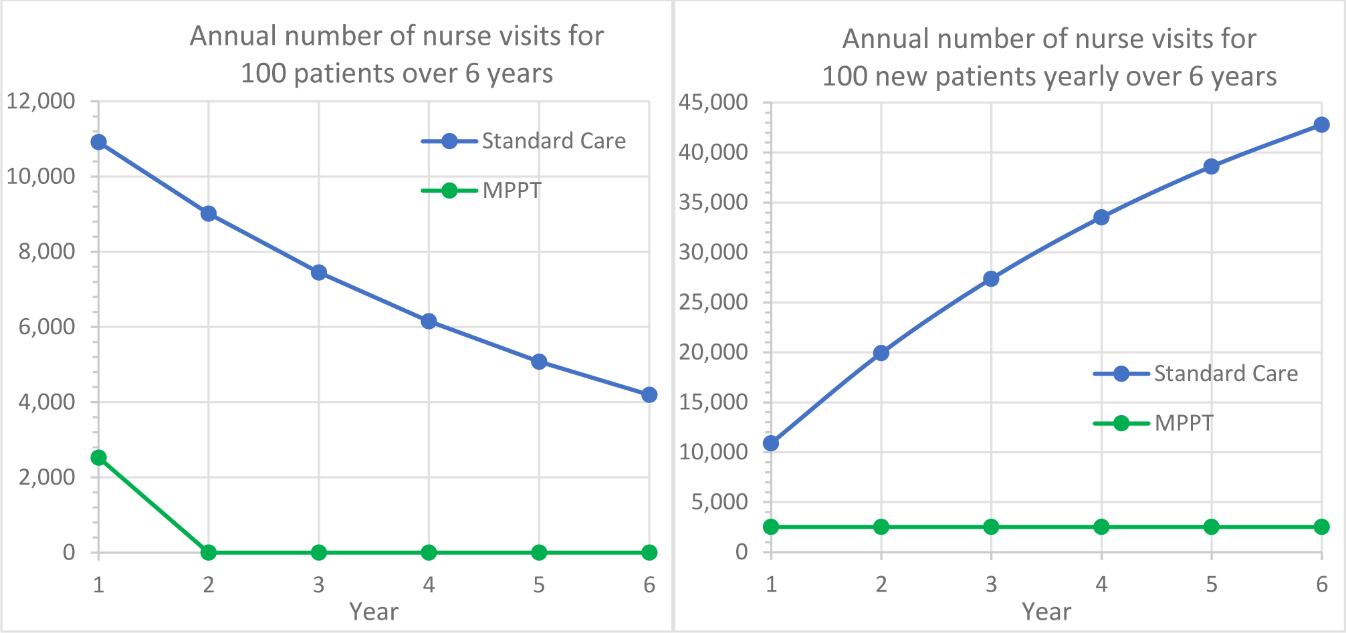


Figure 5. Comparison of MPPT to standard care in terms of annual number of nurse visits or wound evaluations used for the treatment of acute grade 3 pressure ulcers from Year 1 to Year 6. Left graph follows a cohort of 100 new grade 3 pressure ulcers developed Year 1 and their annual need for dressing changes until Year 6. Right graph assumes 100 new grade 3 pressure ulcers dev

elop annually and illustrates the total annual need for nurse visits.

An important difference between MPPT and standard care is that all wounds reach closure with MPPT, whereas with standard care only 17.4% of all grade 3 pressure ulcers close within the first 12 months. As a consequence, with standard care a considerable number of patients are carried-over from one year to the next, leading to an accumulation of wounds requiring care. Fig. 5-left represents a cohort of 100 patients with an acute grade 3 pressure ulcer, starting Year 1. The graph shows the annual numbers of dressing changes or remote wound evaluations required until Year 6. With MPPT, all pressure ulcers close within Year 1 and there is no carry-over to the following year. For standard care, it is assumed that the healing rate remains at 17.5% every year, although this is likely to be lower as chronic wounds are more difficult to heal, which means 82.6% of the patients continue to need visits into the following year. This translates into 9000 dressing changes in Year 2 and almost 4000 visits in Year 6 as close to 40% of the initial 100 patients remain unhealed. As new pressure ulcers will continuously develop, Fig. 5-right shows the development if 100 new grade 3 pressure ulcers emerge every year. With MPPT, all wounds close within a year, which means that none are carried over and that nurse visits or wound evaluations are only required for pressure ulcers developed within that year. In contrast, the low healing rate of 17.5% with standard care will cause a continued accumulative increase in the number of wounds with a corresponding, unsustainable demand for nurse visits, as shown in Fig 5-right.

|  | **Visits per  ulcer per year** | **Visits for 100 patients  Y0 to Y5** | **Annual visits per FTE** | **FTEs required** |
| --- | --- | --- | --- | --- |
| **Standard care (20 min)** | 109.14 | 42,803 | 3,960 | 10.81 |
| **MPPT - 20 min visit** | 47.50 | 4,750 | 3,960 | 1.20 |
| **MPPT - 40 min visit** | 47.50 | 4,750 | 1,980 | 2.40 |
| **MPPT - 60 min visit** | 47.50 | 4,750 | 1,320 | 3.60 |

Table 10. Comparison of nursing staff requirements with standard care vs. MPPT for treating 100 acute grade 3 pressure ulcers from Year 0 to Year 5. FTE: full-time employee with 220 workdays per year and 6 hours daily dedicated to wound dressing changes.

The telemedicine approach is based on the patient, family or carers being able to perform the daily dressing changes. However, this will not always be possible. Table 10 compares the number of nurse FTEs (full-time employees) required from Year 0 to Year 5 to care for 100 acute grade 3 pressure ulcers developed in Year 0 using standard care versus MPPT, i.e. the scenario in Fig. 5 left.

To allow direct comparison of the number of FTEs required, an FTE is throughout assumed to consist of 220 workdays each year with 6 hours daily allocated to visits to perform wound dressing changes. Using the numbers on standard care from Table 9, it is assumed that annually 109.14 dressing changes are required per pressure ulcer and that 17.5% of the wounds reach closure within each year. Each visit is expected to have an average duration of 20 min. Again, using the numbers from Table 9, it is assumed that with MPPT 47.5 dressing changes, corresponding to the days to closure, are needed for each wound to reach wound closure and that the closure rate is 100% within the first year. For MPPT the required number of nurse FTEs are determined for three different scenarios of the patient’s dependency on the nurse, i.e. a duration of the daily visit of either 20 minutes; 40 minutes; or 60 minutes. For 100 new acute grade 3 pressure ulcers developed in Y0, which is the same scenario as in Figure 5-left, these 100 ulcers will from Y0 to Y5 with standard care require 42,803 dressing changes, and with MPPT 4,750 dressing changes. The rightmost column shows that for standard care this will be equivalent to 10.81 FTEs, whereas for MPPT the FTE requirements will vary between 1.2 (20 min per dressing change) and 3.60 (60 min per dressing change). Therefore, the higher efficacy of MPPT means, that even if 1 hour were spent with each patient, it is still considerably less demanding on nursing resources than standard care.

In practice, the implementation of MPPT would be based on a combination of telemedicine and nurse-visits of different duration depending upon the requirements of the individual patient. However, the calculations show that substantial benefits would be gained – even if 1 hour were allocated per dressing change to all patients. Reducing the current time constraints on a visit by a nurse would benefit both patients and healthcare professionals.

Table 10, third column from left, shows the number of nurse-visits the first year and the following 5 years required for managing 100 acute pressure ulcers. Since each nurse-visit involves the use of consumables, and transport to and from the patient, it also reflects non-staff resource consumption. Changing to MPPT will therefore result in an 89% reduction in resource consumption and waste generation and a 100% reduction in the use of antimicrobials, as MPPT does not contain these and their used is expressly excluded in the dressing procedures.

| **Resources** | **Standard care** | | **MPPT** | | **Reduction**  **with MPPT** |
| --- | --- | --- | --- | --- | --- |
|  | **Units** | **Amount** | **Units** | **Amount** |  |
| **Nurse visits** | 109.1 |  | 25.3 |  | 77% |
|  | | |  | |  |
| **Antimicrobials (antibiotics and antiseptics)** | | |  |  |  |
| Dressings | 211.3 | 1.1 kg | 25.3 | 0 | 100% |
| Prescriptions antibiotics | 2.4 | 36.0 g | 0 | 0 | 100% |
| Topical treatments | 8.44 | 0.01 kg | 0 | 0 | 100% |
|  | | |  | |  |
| **Plastics, and other synthetic polymers and silicones** | | |  | |  |
| Dressings | 211.3 | 21.1 kg | 25.3 | 0.03 | >99% |
| Bandages | 16.3 | 0.2 kg | 0 | 0 | 100% |
|  | | |  | |  |
| **CO_2_-emissions** |  | |  | |  |
| Transport miles | 1309.7 | 266.1 kg | 12 | 3.1 kg | >99% |

Table 11. Comparison of the sustainability of standard care (Guest et al. 2018, table 5) vs. MPPT for treating an acute grade 3 pressure ulcer during the first 12 months. Standard care dressings are assumed to contain an average of 5 g of antimicrobials and 60 g of plastics, silicones, and other synthetic polymers per dressing. Prescribed antibiotics are assumed to include a 10-day course of 3x500 mg daily. Bandages primarily contain compression bandaging and these are composed of cotton, viscose, polyethylene terephthalate [PET], cotton-Lycra and PET-Lycra; they are assumed to contain 10 g plastics on average. Topical treatments typically include antiseptics (e.g. iodine and silver), hydrogels, drugs (e.g. phenytoin), and antibiotics (e.g. silver sulfadiazine); they are assumed to contain 1 g antimicrobials each on average. MPPT and its use do not involve any antibiotics, antiseptics, drugs, plastics, or other synthetic polymers and silicones, i.e. their use is contraindicated. For transport, it is assumed that standard care will involve transport by a standard car (127 g CO_2_ per km). MPPT will involve delivery of the treatment by van (160g g CO_2_ per km). For a grade 3 pressure ulcer, the patient will normally only receive one delivery of MPPT. An average distance of 6 miles is assumed between the starting point and the patient.

Table 11 compares, for acute grade 3 pressure ulcers, the use of antimicrobials; plastics and other synthetic polymers and silicones; and CO_2_-emissions for standard care vs. MPPT during the first 12 months of treatment. As only 17.4% of pressure ulcers treated with standard care will close the first year, the resource use will continue the subsequent years, but this has not been included in these calculations. The key findings are a reduction in these compounds by 100%, because MPPT does not involve the use of antimicrobials and plastic and synthetic polymer containing dressings, i.e. they are specifically contraindicated, because they interfere with the effects of MPPT. With MPPT, the only non-natural component used is the non-plastic tape used to fasten the single 100% cotton gauze swab. These resource reductions will be seen across all wound types because MPPT does not involve the use of these compounds. With regards to CO_2_ emissions, MPPT shows a reduction of over 99%. Because community nurses are not required for the daily MPPT dressing changes, the transport associated with MPPT only involves the initial delivery of the MPPT by a delivery van whereas standard care requires transport to and from every dressing change.

### Cost-analysis: Chronic wounds and pressure ulcers

Published data covering healing rates are not available for chronic wounds. However, all the wounds had, prior to MPPT, been receiving standard care, and this allowed for comparison via the differential approach. As all the wounds had failed to close with standard care, and all the wounds closed with MPPT, the improvement rate was 100%.

|  | **Chronic grade 3 per day** | **Chronic grade 4 per day** |
| --- | --- | --- |
| **Bennett et al. (2004)** | £82.76 | £82.76 |
| **Study (MPPT)** | £40.81 | £38.64 |
| **Savings with MPPT** | 50.7% | 53.3% |

Table 12. Comparing daily cost of treatment of chronic grade 3 and 4 pressure ulcers with MPPT to standard care (Bennett et al. 2004). Mean costs are used. Costs have been converted to 2022 prices, using the inflation factor of 1.385%.

The mean total costs of treatment with MPPT until full closure were £3,999 for chronic grade 3 and £7,255 for chronic grade 4 wounds; they had all been treated with one or more different antimicrobials as standard care prior to MPPT and had failed to close. The average number of days to closure were 98 days and 188 days, respectively. Bennett et al. (2004) had calculated the daily cost of treatment of grade 3 and 4 pressure ulcers with critical colonisation to be £82.76 in 2022 prices. Table 12 compares the costs of the present study to Bennett et al. (2004) and finds average daily savings of 51% and 53%, respectively. These figures are per day and the overall economic benefit is consequently bigger as the wounds reached closure with MPPT and the treatment stopped.

These data are also in agreement with Guest et al. (2018), who for acute wounds, which are easier to heal, found treatment costs the first year of £9,679 and £17,610 for grade 3 and 4 pressure ulcers, respectively. Comparing the costs of MPPT in the present study to Guest et al. (2018) finds cost saving of 59% for both chronic grade 3 and 4 wounds and healing rates of 100% compared to 17% for standard care the first year. This confirms that the considerable cost savings with MPPT in acute wounds are also seen in the chronic wounds.

### Cost-analysis: Wounds acting as draining fistulas

At enrolment, the wounds acting as draining fistulas were being treated with standard care and were generally festering and associated with deep and widespread soft tissue infection. As shown in Fig. 2, it took approximately 2.5 months to stabilise the wounds during which time the daily use of MPPT decreased by 63%.

|  | **Bennett et al. (2004)** | | **Study** | |
| --- | --- | --- | --- | --- |
|  | **Osteomyelitis** | | **Draining fistula (osteomyelitis)** | |
|  | **One year** | **1 month** | **First year** | **Month 12** |
| **Cost** | £95,400 | £7,950 | £24,054 | £1,230 |
| **Savings with MPPT** | 74.8% | 84.5% |  |  |

Table 13. Comparison of average costs of treating draining fistulas using MPPT to standard care for ulcers with underlying osteomyelitis (Bennett et al. 2004, Table 2). Bennett et al. (2004) provided cost of treatment on a per day basis, and, based on these figures, the cost of treatment for one year and for one month have been determined. For MPPT, the cost of first year and 12’th month are provided. The first year will include the initial 2.5 months whilst the wound comes under control, and the 12’th month represents the costs going forward once the wound is under control and the treatment aim is maintenance. Costs are in 2022 prices, using an inflation factor of 1.385%. For MPPT, it is assumed that wound experts guided the patients daily the first month, every second day the second month, every third day the third month and thereafter once monthly.

For cost-comparisons (Table 13), the total costs of MPPT during its first year of use, which includes the initial period of bringing the wound under control, and during its 12^th^ month of use, which represents the monthly cost for continued treatment, are compared to the data from Bennett et al. (2004). Bennett et al. provide data for wounds with osteomyelitis, which correspond to the wounds acting as draining fistulas in the present study. During the first year of use, the cost savings with MPPT were 74.8% relative to standard care for wounds with osteomyelitis. The 12’th month of treatment with MPPT represents the costs that can be expected after the wound has been brought under control. At this point in time, MPPT provided savings of 84.5% relative to standard care for wounds with osteomyelitis. The use of MPPT to manage draining fistulas will be associated with improvements in quality-of-life as patients will not be required to maintain bed rest and treatment can be delivered by telemedicine providing freedom and independence. Feedback from patients also indicate that they are feeling better, which is consistent with the reduced level of soft tissue infection and toxaemia.

## Telemedicine

The telemedicine approach was well received and facilitated self-care and independence. The level of support required rapidly reduced as participants became familiar with the process. Current state-of-the art wound-care involves a nurse seeing the wound every other day at the GP or in the patient’s home. This involves travelling to an appointment which often is incompatible with the patient’s schedule or waiting at home for the nurse to arrive at a non-specified time between 9AM and 5PM. This interferes with keeping a job, a business or attend education, and family and friends stop relying upon that person’s contribution, leading to social isolation. In standard wound care, this arrangement runs between 3 months and several years. Over time, the person is forced to cancel his/her own life commitments to make him/herself available to care, and soon all life decisions revolve around the wound. Therefore, most patients are motivated for self-treatment to regain their independence and alter their daily focus of attention. Having self-treated with MPPT once, the patient retains the learned procedures and requires minimal assistance with future ulcers. The MPPT/telemedicine approach has also been used successfully for diabetic foot and venous leg ulcers and surgical wounds.

The participants were able to fully benefit from the independence provided via the telemedicine approach. Some participants had been on bed rest for months or years due to their wounds and changing to MPPT enabled them to resume an active lifestyle.

## Equality

MPPT is effective in immunocompetent and immunocompromised individuals, and in different wound types. To the individual, slow healing means loss of opportunities. The considerably quicker healing achieved with MPPT relative to standard care can reduce the disadvantage experienced by patients with wounds.

MPPT is suitable for telemedicine. This can provide equal access to treatment with guidance by wound experts independently of locations, including remote areas. If the patient is unable to perform own treatment, the telemedicine approach can provide expert advice to a non-wound-expert meaning that equal quality of the treatment can be provided by healthcare professionals independently of prior training as well as by non-professional, dedicated carers. This can facilitate equal quality of the treatment. In most cases, the patient, a family member or a carer are able to perform the treatment, and the non-dependency on hands-on support from healthcare professionals, provides an independence that reduces loss of work, school and social opportunities.

## Sustainability

The FDA concluded in 2022 that wounds constitute an area of unmet medical need (Verma et al. 2022). Due to the lack of effective treatments, the burden of wounds on the healthcare systems continues to increase by 11% annually (Guest et al. 2020) and calculations indicate that in 2022 wounds in community care alone demand 20% of the entire NHS budget (*S1: Costs of wounds*). Furthermore, the treatment of wounds with current standard care results in substantial medical waste, which in 2022 includes at least 53,000 tonnes of plastics and chemical waste, 1.8 million tons CO_2_ emissions due to the involved transport; and over 66 tonnes of antimicrobials released into the environment contributing to AMR, reductions in biodiversity, and climate change (*S1: Costs of wounds*). When evaluating the sustainability of MPPT it must be taken into consideration that MPPT has demonstrated better clinical efficacy and safety than standard care.

### Economic sustainability

- Healthcare costs: Figure 4 compares the consequences in patient numbers and costs of using standard care (Guest et al. 2018) vs. MPPT for treating acute grade 3 pressure ulcers and Figure 5 provides the equivalent calculation for nursing time needed for wound dressing changes. No healthcare system will be able to cope with this level of growth in the number of patients and consequent costs. The outcome will necessarily be a reduction in the funding available per wound and is likely to render some wounds untreated or push their treatment initiation into the future, thereby causing chronicity and further increase the severity and costs of their treatment as well as the treatment of consequential follow-on conditions, e.g. osteomyelitis, heart disease and diabetes. The existing arrangement is therefore unsustainable. In comparison, MPPT was able to heal all acute wounds, which meant that there is no carry-over effect across years. MPPT therefore has the potential to render the system sustainable.
- Healthcare resources: Figure 5 and Tables 9 and 10 compare the number of nurse-visits required with standard care and with MPPT for treating acute grade 3 pressure ulcers, which are the most common type. Using Table 10, the number of nurse visits was reduced by 89% when changing from standard care to MPPT. A visit involves both staff time as well as dressing materials and transport. The implementation of MPPT will therefore free-up substantial healthcare staff resources, reduce waste from consumables, reduce transport requirements and associated CO_2_ emissions.
- Social care costs: A non-healing wound impacts the physical and mental health of the person and may result in the person changing from being an active independent individual to a person dependent upon social care, possibly unable to remain in own home. Social care budgets are already stretched and a continuously growing influx of individuals with non-healing wounds requiring care is unsustainable. MPPT will be able to heal a high percentage of the wounds and thereby allow more people to remain active, independent and in own home for longer. This will help to reduce the costs of social care, thereby improving sustainability.
- Individual’s costs of living: A non-healing or slow-healing wound may interfere with a person’s ability to hold a job, run a business, complete an education etc. Quicker healing will reduce the impact of the wound on the person’s life and livelihood. Furthermore, by combining MPPT with telemedicine, it is possible to support the person’s independence also during the healing period by allowing them to decide when and where to perform the dressing change. This will support economic sustainability both at the societal and personal level.

### Social sustainability

- AMR: Antimicrobial resistance (AMR) has been dubbed the silent pandemic (Antimicrobial Resistance Collaborators 2022). The annual number of deaths due to AMR is rising exponentially and the number of last resort antibiotic treatments is declining and has in some instances proven exhausted. The role of antibiotics in creating antimicrobial resistance is well documented, but it is less well known that antiseptics, e.g. chlorhexidine, PHMB and silver, also cause resistance, including cross resistance to antibiotics (Wassenaar et al. 2015; Wand et al. 2016; Ignak et al. 2017; Hassan et al. 2019; McCarlie et al. 2020; McCarlie 2021; van Dijk et al. 2022; Wicaksono et al. 2022; Yonathan et al. 2022); see also *S2: Microbiome, SIS, AMR and virulence*. Accumulation of toxic antiseptics with negative impacts on health have been shown in the environment as well as in bodily organs (Ferdous et al. 2020). The use of antimicrobials therefore needs to be limited to infections where efficacy has been demonstrated. The efficacy of antibiotics and antiseptics to remove infections in wounds has never been demonstrated and this lack of efficacy has been identified by both the US FDA (FDA 2016; Verma et al. 2022) and NICE (NICE 2014, 2016). The scale of this unwarranted use of antimicrobials and its contribution to the continuous creation of AMR, makes the use of antimicrobials in wounds irresponsible and unsustainable. Furthermore, the use of antibiotics and antiseptics will exacerbate the infection (please see *S2: Microbiome, SIS, AMR and virulence* for details). Such deterioration of the wound necessarily leads to an increased treatment period, which will unavoidably increase the consumption of resources as well as the generation of waste. This renders the continued use of antibiotics and antiseptics in wound care further unsustainable and highlights the urgent need to replace them with alternatives. MPPT is effective in removing infections in wounds, including antimicrobial resistant strains. It contains no antimicrobials, has no antimicrobial action, and advises against the use of antimicrobials in its treatment procedures. Its use does therefore not contribute to the creation of AMR but offers a sustainable solution.
- Personal health and social integration: For the individual person, having a chronic wound will in many cases be associated with pain, smell, anxiety, limited mobility and reduced physical well-being. All these factors contribute to social isolation, depression and difficulties living independently. Furthermore, the person will for months to years one or more times every week be forced to either visit a local clinic or to remain at home waiting for a nurse to visit at a non-specified time between 9AM and 5PM in order to have the dressing changed. Such a requirement is incompatible with holding a job, running a business, or taking an education etc. and consequently has a severe economic impact on the person’s livelihood, and also affects their ability to establish and maintain a social network. Additionally, some patients will be placed on bed rest for months to years to support healing, which only exacerbates the above conditions and causes a deterioration in generalised health as well as follow-on future health complications (Teasell and Dittmer 1993; Dittmer and Teasell 1993; Norton and Sibbald, 2004). All of this leads to poor quality of life, social isolation, decreased physical and mental health and reduced ability to remain in own home and increased dependence on care (family and social care), i.e. a down-ward spiral, which is unsustainable at an individual as well as a societal level. MPPT has demonstrated a faster healing rate, a higher number of wounds closed, a large proportion of patients themselves being able to care for their own wounds; and MPPT does not require bed rest for healing to occur. These factors contribute to a reduction in social isolation and depression and an increase in quality of life and the ability to live independently and retain a livelihood. If a wound is associated with osteomyelitis, arrangements can be made to allow the person to remain active. For most individuals, MPPT will therefore considerably improve their situation and have a lesser impact on their lives less compared to current care.

### Environmental sustainability

- Antibiotics and antiseptics: Both antibiotics and antiseptics contribute to antimicrobial resistance (see above) and very high proportions of the used antibiotics and antiseptics escape sewage treatment plants and landfills (Polianciuc et al. 2020; Larsson and Flach 2022). They end up in soil, aquatic environments and are carried by precipitation to all regions of the Earth (Di Cesare et al. 2017; Els et al. 2019). Antibiotics and antiseptics are generally very stable chemically and will affect the environments they reach. Microbes in the soil, aquatic environment and air form the basis for all plant and animal life, as they are key to the control of carbon and nitrogen cycles, nitrogen fixation to enable plant growth, and soil and aquatic sediment structures, see e.g. Cavicchioli et al. (2019) and Cycoń et al. (2019). The presence of antibiotics and antiseptics - see Yonathan et al. (2022) for a review of the impact of nano-silver as an example - will affect the microbial composition of the environment by favouring certain organisms and this results in reduced biodiversity at the microbial level (Cycoń et al. 2019), which in turn destabilises the natural rate and flow of the nitrogen and carbon cycles and renders some resources superfluous whilst exhausting others (Fennel 2017). The exhaustion of the upper soil and sea floor causes compaction, erosion and deforestation, leaving behind deserts of non-fertile land and ocean beds without the adequate types and numbers of organisms to catch the carbon and hold on to it, or to convert the nitrogen at the right pace (McGee 2020). Currently, the soil microbiome is responsible for around 50% of the CO_2_ absorption and storage as well as for nitrogen fixation; the microorganisms in the oceans are responsible for more than 50% of the absorption of CO_2_ from the atmosphere. It is consequently crucial to maintain healthy microbiomes (Bond-Lamberty et al. 2018). The continued use of antibiotics and antiseptics, particularly where they provide no benefits, is therefore irresponsible and unsustainable. MPPT provides better clinical outcomes and it involves no antibiotics or antiseptics.
- Surfactants: Surfactants are compounds that lower the surface tension and include detergents, wetting agents, and emulsifiers (Badmus et al. 2021). They have antimicrobial properties (Falk 2019) and are extensively used in healthcare, e.g. in wound rinsing solutions. They escape from sewage plants and landfills and end up in the environment. By affecting surface tension, they also affect the exchange of gasses between air and water. Their impact can be substantial, e.g. between1994 and 2007, oceans absorbed 34 gigatonnes of carbon dioxide, or 31 percent of what humans put into the atmosphere during that time (Gruber et al. 2019). Surfactants have been seen to suppress the absorption of CO_2_ into the oceans by 32% (Pereira et al. 2018). Such blockade will lead to increases in atmospheric CO_2_, even if CO_2_ emission itself into the atmosphere does not increase and will accelerate climate change. Surfactants also contribute to heavy rain (Sugo et al. 2019), which can cause flooding, including flash flooding, and significantly increase erosion (Piacentini et al. 2018). The release of surfactants into the environment is therefore not environmentally sustainable. MPPT and its use do not involve surfactants and directly advises against its use in connection with MPPT treatment.
- Plastics, and other synthetic polymers and silicones, and chemicals: Calculations indicate that, in 2022, at least 53,000 tonnes of waste from wound dressing changes are created in the UK (*S1: Cost of wounds*). Most of this contains plastics, synthetic polymers and silicones and other chemicals that are not readily recyclable and therefore lead to pollution. The dressings have not been shown to be effective in wound care. Most of this medical waste is avoidable by changing to MPPT, which can increase sustainability in wound care, since the components of MPPT are biologically recyclable and can return directly into the biological cycle; all packaging materials are readily recyclable or biodegradable; and its use is not associated with the use of antimicrobials, antiseptics/disinfectants, surfactants, plastics or other synthetic polymers or silicones, or other chemicals or petroleum-based products.
- CO_2_-emission and net-zero: Quicker wound healing means fewer wound dressing changes, and this will result in a reduced need for transport and its associated CO_2_-emission. Additionally, MPPT is suitable for telemedicine, which can provide further reductions in transport need. The shift to MPPT will therefore improve sustainability.

## Summary

The study found that MPPT consistently removed soft tissue infection. In acute and chronic wounds and ulcers, this led to healing and closure and, in draining fistulas, to improved control of the soft tissue infection in the wound associated with the fistula. No adverse events were seen even after daily application onto muscle and bone for over 2 years. The first year, cost savings for acute wounds ranged from 60% for grade 1 to 85% for grade 4 wounds, and for chronic grade 3 and 4 wounds, it was more than 50%. For the subsequent years, savings for acute and chronic wounds were 100% because the wounds had already closed. The fact that MPPT closed the wounds much quicker, limited the impact on the participant’s life and on society and health care systems. In contrast, standard care required many months and the majority of wounds did not reach closure within the first year, leading to a complete disruption to the person’s life. Slow healing also means increased risk of deterioration and the possibility of developing osteomyelitis, which is a permanently life changing event.

The development of a pressure ulcer is a common event in connection with the initial spinal cord injury due to the requirement of full bed rest until the spine has stabilised. These pressure ulcers often affect people for years as well as the initial essential rehabilitation efforts, resulting in the permanent loss of abilities that could have been preserved, if a normal rehabilitation programme could have been followed; this leaves the patient permanently more dependent on care. Access to an effective treatment at this stage could therefore substantially help making the most of the rehabilitation efforts.

For persons with a draining fistula, cost savings of 74.8% to 84.4% were gained and, because MPPT does not require bed rest and treatment can be organised via telemedicine, the participants’ quality of life improved substantially. It is well known that bed rest has detrimental physical and mental health consequences (Teasell and Dittmer 1993; Dittmer and Teasell 1993; Norton and Sibbald, 2004) and the ability of the participants themselves to select the time for their dressing changes meant that they could control their own schedule, e.g. enabling them to hold a job or take an education. It is also important, in a future study, to evaluate the benefits of MPPT for managing draining fistulas with regards to impending surgery as the extent of soft issue infection and the size of the fistula itself are reduced, which means that less tissue needs to be removed, thereby preserving vital tissue to act as cushioning to prevent future pressure ulcers.

The British patient organisation SIA (Spinal Injuries Association) conducted in 2022 a survey on the experience of current and prior MPPT-users in the SCI-community (Smith 2023). 41 individuals completed the survey reporting on 49 wounds, and their response wholly matched the findings of this study, i.e. a 100% closure rate for acute and chronic wounds and pressure ulcers and the control of draining fistulas. This supports the value of non-interventional, observational studies and confirms that the findings of this study reflect real-world use. Given that the external controls (Bennett et al. 2004; Guest et al. 2018) were retrospective studies of routine clinical care, i.e. in a real world setting, the estimated benefits resulting from the implementation of MPPT in clinical practice are also likely to be accurate.

## References:

Antimicrobial Resistance Collaborators. Global burden of bacterial antimicrobial resistance in 2019: a systematic analysis. *Lancet*. 2022;399(10325):629-655. doi:10.1016/S0140-6736(21)02724-0

Badmus, S.O., Amusa, H.K., Oyehan, T.A. *et al.* Environmental risks and toxicity of surfactants: overview of analysis, assessment, and remediation techniques. *Environ Sci Pollut Res* **28,** 62085–62104 (2021). <https://doi.org/10.1007/s11356-021-16483-w>

Bennett G, Dealey C, Posnett J. The cost of pressure ulcers in the UK. *Age Ageing*. 2004;33(3):230-235. doi:10.1093/ageing/afh086.

Bilyayeva OO, Neshta VV, Golub AA, Sams-Dodd F. Comparative Clinical Study of the Wound Healing Effects of a Novel Micropore Particle Technology: Effects on Wounds, Venous Leg Ulcers, and Diabetic Foot Ulcers. Wounds. 2017; 29(8):1-9.

Bond-Lamberty B, Bailey VL, Chen M, Gough CM, Vargas R. Globally rising soil heterotrophic respiration over recent decades. *Nature*. 2018;560(7716):80-83. doi:10.1038/s41586-018-0358-x

Cavicchioli R, Ripple WJ, Timmis KN, et al. Scientists' warning to humanity: microorganisms and climate change. Nat Rev Microbiol. 2019;17(9):569-586. doi:10.1038/s41579-019-0222-5

Chan BC, Nanwa N, Mittmann N, Bryant D, Coyte PC, Houghton PE. The average cost of pressure ulcer management in a community dwelling spinal cord injury population. Int Wound J. 2013;10(4):431-440. doi:10.1111/j.1742-481X.2012.01002.x

Cycoń M, Mrozik A, Piotrowska-Seget Z. Antibiotics in the Soil Environment-Degradation and Their Impact on Microbial Activity and Diversity. Front Microbiol. 2019;10:338. Published 2019 Mar 8. doi:10.3389/fmicb.2019.00338.

Davi R, Mahendraratnam N, Chatterjee A, Dawson CJ, Sherman R. Informing single-arm clinical trials with external controls. Nat Rev Drug Discov. 2020;19(12):821-822. doi:10.1038/d41573-020-00146-5

Di Cesare A, Eckert EM, Rogora M, Corno G. Rainfall increases the abundance of antibiotic resistance genes within a riverine microbial community. *Environ Pollut*. 2017;226:473-478. doi:10.1016/j.envpol.2017.04.036

Dittmer DK, Teasell R. Complications of immobilization and bed rest. Part 1: Musculoskeletal and cardiovascular complications. *Can Fam Physician*. 1993;39:1428-1437.

Falk NA. Surfactants as Antimicrobials: A Brief Overview of Microbial Interfacial Chemistry and Surfactant Antimicrobial Activity. J Surfactants Deterg. 2019 Sep;22(5):1119-1127. doi: 10.1002/jsde.12293. Epub 2019 Jun 4. PMID: 32336911; PMCID: PMC7166552.

FDA. Role of Single Group Studies in Agency for Healthcare Research and Quality Comparative Effectiveness Reviews (2013), AHRQ Publication No. 13-EHC007-EF.

FDA. FDA executive summary. Classification of wound dressings combined with drugs. Prepared for the Meeting of the General and Plastic Surgery Devices Advisory Panel September 20–21, 2016. FDA website. 2016;38–39. <https://www.fda.gov/media/100005/download>

Fennel K. Biogeochemistry: Ocean hotspots of nitrogen loss. *Nature*. 2017;551(7680):305-306. doi:10.1038/551305a

Ferdous Z, Nemmar A. Health Impact of Silver Nanoparticles: A Review of the Biodistribution and Toxicity Following Various Routes of Exposure. Int J Mol Sci. 2020 Mar 30;21(7):2375. doi: 10.3390/ijms21072375. PMID: 32235542; PMCID: PMC7177798.

Guest JF, Ayoub N, McIlwraith T, Uchegbu I, Gerrish A, Weidlich D, Vowden K, Vowden P. Health economic burden that wounds impose on the National Health Service in the UK. BMJ Open. 2015 Dec 7;5(12):e009283. doi: 10.1136/bmjopen-2015-009283.

Guest JF, Ayoub N, McIlwraith T, Uchegbu I, Gerrish A, Weidlich D, Vowden K, Vowden P. Health economic burden that different wound types impose on the UK's National Health Service. Int Wound J. 2017 Apr;14(2):322-330. doi: 10.1111/iwj.12603.

Guest JF, Fuller GW, Vowden P, Vowden KR. Cohort study evaluating pressure ulcer management in clinical practice in the UK following initial presentation in the community: costs and outcomes. BMJ Open. 2018 Jul 25;8(7):e021769. doi: 10.1136/bmjopen-2018-021769.

Guest JF, Fuller GW, Vowden P. Cohort study evaluating the burden of wounds to the UK's National Health Service in 2017/2018: update from 2012/2013. *BMJ Open*. 2020;10(12):e045253. Published 2020 Dec 22. doi:10.1136/bmjopen-2020-045253

ICH E10 (2000) Choice of control group and related issues in clinical trials. ICH harmonised tripartite guideline.

Ignak S, Nakipoglu Y, Gurler B. Frequency of antiseptic resistance genes in clinical staphycocci and enterococci isolates in Turkey. *Antimicrob Resist Infect Control*. 2017;6:88. Published 2017 Aug 30. doi:10.1186/s13756-017-0244-6

Hassan KA, Naidu V, Edgerton JR, et al. Short-chain diamines are the physiological substrates of PACE family efflux pumps. *Proc Natl Acad Sci U S A*. 2019;116(36):18015-18020. doi:10.1073/pnas.1901591116

Larsson, D.G.J., Flach, CF. Antibiotic resistance in the environment. Nat Rev Microbiol 20, 257–269 (2022). <https://doi.org/10.1038/s41579-021-00649-x>

Malmivaara A, Zampolini M, Stam H, Gutenbrunner C. Pros and Cons of Randomized Controlled Trials and Benchmarking Controlled Trials in Rehabilitation: An Academic Debate within the European Academy of Rehabilitation Medicine. Journal of Rehabilitation Medicine. 2022 Oct 10;54:jrm00319–jrm00319.

McCarlie S, Boucher CE, Bragg RR. Molecular basis of bacterial disinfectant resistance. *Drug Resist Updat*. 2020;48:100672. doi:10.1016/j.drup.2019.100672

McCarlie S. A new front. The Biologist. 2021 September 8. <https://thebiologist.rsb.org.uk/biologist-features/too-much-of-a-good-thing>

McGee CF. The effects of silver nanoparticles on the microbial nitrogen cycle: a review of the known risks. *Environ Sci Pollut Res Int*. 2020;27(25):31061-31073. doi:10.1007/s11356-020-09548-9

Naik PP, Farrukh SN. Influence of Ethnicities and Skin Color Variations in Different Populations: A Review. *Skin Pharmacol Physiol*. 2022;35(2):65-76. doi:10.1159/000518826

NICE (2014) Pressure ulcers: prevention and management. 2014. NICE, UK.

NICE. Chronic wounds: advanced wound dressings and antimicrobial dressings. 2016; <http://nice.org.uk/guidance/esmpb2>.

Norton L, Sibbald RG. Is bed rest an effective treatment modality for pressure ulcers? *Ostomy Wound Manage*. 2004;50(10):40-53.

O'Sullivan O, Hayton L, Findlay-Cooper K, Phillip R. Novel micropore particle technology for spinal cord injury chronic wound healing: a new paradigm? [published online ahead of print, 2020 Aug 4]. *BMJ Mil Health*. 2020;bmjmilitary-2020-001509. doi:10.1136/bmjmilitary-2020-001509

Pereira, R., Ashton, I., Sabbaghzadeh, B. *et al.* Reduced air–sea CO_2_ exchange in the Atlantic Ocean due to biological surfactants. *Nature Geosci* **11,** 492–496 (2018). https://doi.org/10.1038/s41561-018-0136-2

Piacentini T, Galli A, Marsala V, Miccadei E. Analysis of soil erosion induced by heavy rainfall: A case study from the NE Abruzzo Hills Area in Central Italy. Water. 2018 Sep 22;10(10):1314.

Polianciuc SI, Gurzău AE, Kiss B, Ştefan MG, Loghin F. Antibiotics in the environment: causes and consequences. Med Pharm Rep. 2020;93(3):231-240. doi:10.15386/mpr-1742

Rahman R, Ventz S, McDunn J, et al. Leveraging external data in the design and analysis of clinical trials in neuro-oncology. Lancet Oncol. 2021;22(10):e456-e465. doi:10.1016/S1470-2045(21)00488-5

Rennert R, Golinko M, Yan A, Flattau A, Tomic-Canic M, Brem H. Developing and evaluating outcomes of an evidence-based protocol for the treatment of osteomyelitis in Stage IV pressure ulcers: a literature and wound electronic medical record database review. Ostomy Wound Manage. 2009;55(3):42-53.

Ryan E. The use of a micropore particle technology in the treatment of acute wounds. J Wound Care. 2017; 26(7): 404-413.

Smith, D., 2023a. Addressing the challenges of treating pressure ulcers following spinal cord injury with Amicapsil-SCI micropore particle technology. Presented at the 62nd International Spinal Cord Society Annual Scientific Meeting (ISCoS 2023), Edinburgh, p. P049.

Smith, D., 2023b. Survey of user-experiences in the spinal cord injured-community with MPPT for treating wounds and pressure ulcers and for controlling soft tissue infection caused by osteomyelitis. submitted.

Sugo T, Okochi H, Uchiyama R, Yamanokoshi E, Ogata H, Katsumi N, Nakano T. The role of humic-like substances as atmospheric surfactants in the formation of summer-heavy rainfall in downtown Tokyo. City and Environment Interactions. 2019 Nov 1;3:100022.

Teasell R, Dittmer DK. Complications of immobilization and bed rest. Part 2: Other complications. *Can Fam Physician*. 1993;39:1440-1446.

van Dijk, H.F.G., Verbrugh, H.A. & Ad hoc advisory committee on disinfectants of the Health Council of the Netherlands. Resisting disinfectants. *Commun Med* **2,** 6 (2022). <https://doi.org/10.1038/s43856-021-00070-8>

Verma KD, Lewis F, Mejia M, Chalasani M, Marcus KA. Food and Drug Administration perspective: Advancing product development for non-healing chronic wounds. *Wound Repair Regen*. 2022;30(3):299-302. doi:10.1111/wrr.13008

Wand ME, Bock LJ, Bonney LC, Sutton JM. Mechanisms of Increased Resistance to Chlorhexidine and Cross-Resistance to Colistin following Exposure of Klebsiella pneumoniae Clinical Isolates to Chlorhexidine. Antimicrob Agents Chemother. 2016;61(1):e01162-16. Published 2016 Dec 27. doi:10.1128/AAC.01162-16

Wassenaar TM, Gunzer F. The prediction of virulence based on presence of virulence genes in E. coli may not always be accurate. *Gut Pathog*. 2015;7:15. Published 2015 Jun 19. doi:10.1186/s13099-015-0062-4

Wicaksono WA, Erschen S, Krause R, Müller H, Cernava T, Berg G. Enhanced survival of multi-species biofilms under stress is promoted by low-abundant but antimicrobial-resistant keystone species. *J Hazard Mater*. 2022;422:126836. doi:10.1016/j.jhazmat.2021.126836

Wounds-UK (2018) https://www.wounds-uk.com/news/details/new-research-finds-uk-nurses-carry-out-180-wound-dressing-changes-a-year-on-each-chronic-wound-patient-

Yap TA, Jacobs I, Baumfeld Andre E, Lee LJ, Beaupre D, Azoulay L. Application of Real-World Data to External Control Groups in Oncology Clinical Trial Drug Development. *Front Oncol*. 2022;11:695936. Published 2022 Jan 6. doi:10.3389/fonc.2021.695936

Yonathan K, Mann R, Mahbub KR, Gunawan C. The impact of silver nanoparticles on microbial communities and antibiotic resistance determinants in the environment. *Environ Pollut*. 2022;293:118506. doi:10.1016/j.envpol.2021.118506
