# Supplementary material for: Stable closure of acute and chronic wounds and pressure ulcers and control of draining fistulas from osteomyelitis in persons with spinal cord injuries: non-interventional study of MPPT passive immunotherapy delivered via telemedicine in community care
Source: Front Med (Lausanne). 2024 Jan 5;10:1279100. doi: 10.3389/fmed.2023.1279100 (PMC10797031; doi:10.3389/fmed.2023.1279100)
Supplement: Supplementary file 7 [file Data_Sheet_7.docx]

# S7: Monitoring infective organisms

*Pseudomonas aeruginosa* and *Serratia marcescens* are facultative anaerobic, multidrug-resistant (intrinsic and required), and biofilm forming (Nordmann and Poirel, 2002). *P. aeruginosa* and *S. marcescens* secrete pigmented toxins that affect the immune system as well as different microbial species. *P. aeruginosa* produces green pigment, whereas *S. marcescens* has a characteristic bright red pigment (Liu and Nizet, 2009). Both bacteria secrete pore-forming toxins, which are potent virulence factors capable of perforating membranes of host cells and the toxins of both *P. aeruginosa* and *S. marcescens* have the capacity to disrupt cell-cell junctions, hence breaching major tissue barriers (Reboud et al., 2017).

*S. marcescens* has been associated with extensive tissue abscess formation in immunocompromised individuals, including many small abscesses scattered in the tissue (Fawcett et al., 1986). It is nosocomial and results in considerable morbidity and mortality in immunocompromised patients. It usually forms part of the skin microbiome of SCI people [S2] and has a tendency to cause invasive infection when the person’s defences are additionally strained, e.g. by a wound. It is associated with a range of infections, such as pneumonia, wound and skin infections, urinary tract infection, meningitis, and bacteraemia and sepsis. *S. marcescens* can contaminate disinfectants, such as Chlorhexidine solutions, and cause outbreaks (de Frutos et al., 2017; Francés-Cuesta et al., 2021; Knowles et al., 2000; Vigeant et al., 1998).

*P. aeruginosa* is also common in healthcare settings and usually part of the natural skin microbiome in people with SCI (Fawcett et al., 1986). It can evade the host’s immune defence (Alhede et al., 2009) and its large repertoire of virulence factors (Morin et al., 2021) enables it to cause serious infections essentially in all organs of the body, among others lung infection, soft tissue infection in wounds, urinary tract infection, and bacteraemia and sepsis (Morin et al., 2021). *P. aeruginosa* and *S. aureus* act synergistically to enhance colonisation, virulence and persistence and are the two most prevalent types of polymicrobial infection in chronic wounds (DeLeon et al., 2014). *P. aeruginosa* promotes an invasive phenotype in *S. aureus*, which is the most common causative agent of osteomyelitis (Alves et al., 2018).

Due to their characteristic pigments (Liu and Nizet, 2009), these species could be followed using photographic evidence and they were used to monitor the infection status of the wound. However, they were only used as markers of infection because many other species will also be present and may be the primary causative agent(s) of the infection. The pigment of *S. marcescens* often resembled fresh blood but by catching some on a white gauze and leaving it exposed to air for at least 3-4 hours, it was possible to distinguish as blood would change colour to dark-brown, whereas the pigment would not change colour, i.e.it would remain bright red.

MPPT uses capillary-evaporation to remove bacterial and fungal toxins [S3] and, if the wound is covered by a dressing, these often coloured toxins or breakdown products will be deposited on the dressing (Fig. 1). The dressing therefore provides information on wound status and offers a simple method to detect whether certain infective species are present.

## References:

Alhede, M., Bjarnsholt, T., Jensen, P.Ø., Phipps, R.K., Moser, C., Christophersen, L., Christensen, L.D., van Gennip, M., Parsek, M., Høiby, N., Rasmussen, T.B., Givskov, M., 2009. Pseudomonas aeruginosa recognizes and responds aggressively to the presence of polymorphonuclear leukocytes. Microbiology (Reading) 155, 3500–3508. https://doi.org/10.1099/mic.0.031443-0

Alves, P.M., Al-Badi, E., Withycombe, C., Jones, P.M., Purdy, K.J., Maddocks, S.E., 2018. Interaction between Staphylococcus aureus and Pseudomonas aeruginosa is beneficial for colonisation and pathogenicity in a mixed biofilm. Pathog Dis 76. https://doi.org/10.1093/femspd/fty003

de Frutos, M., López-Urrutia, L., Domínguez-Gil, M., Arias, M., Muñoz-Bellido, J.L., Eiros, J.M., Ramos, C., 2017. Serratia marcescens outbreak due to contaminated 2% aqueous chlorhexidine. Enferm Infecc Microbiol Clin 35, 624–629. https://doi.org/10.1016/j.eimc.2016.06.016

DeLeon, S., Clinton, A., Fowler, H., Everett, J., Horswill, A.R., Rumbaugh, K.P., 2014. Synergistic interactions of Pseudomonas aeruginosa and Staphylococcus aureus in an in vitro wound model. Infect Immun 82, 4718–4728. https://doi.org/10.1128/IAI.02198-14

Fawcett, C., Chawla, J.C., Quoraishi, A., Stickler, D.J., 1986. A study of the skin flora of spinal cord injured patients. J Hosp Infect 8, 149–158. https://doi.org/10.1016/0195-6701(86)90041-1

Francés-Cuesta, C., Sánchez-Hellín, V., Gomila, B., González-Candelas, F., 2021. Is there a widespread clone of Serratia marcescens producing outbreaks worldwide? Journal of Hospital Infection 108, 7–14. https://doi.org/10.1016/j.jhin.2020.10.029

Knowles, S., Herra, C., Devitt, E., O’Brien, A., Mulvihill, E., McCann, S.R., Browne, P., Kennedy, M.J., Keane, C.T., 2000. An outbreak of multiply resistant Serratia marcescens: the importance of persistent carriage. Bone Marrow Transplant 25, 873–877. https://doi.org/10.1038/sj.bmt.1702218

Liu, G.Y., Nizet, V., 2009. Color me bad: microbial pigments as virulence factors. Trends Microbiol 17, 406–413. https://doi.org/10.1016/j.tim.2009.06.006

Morin, C.D., Déziel, E., Gauthier, J., Levesque, R.C., Lau, G.W., 2021. An Organ System-Based Synopsis of Pseudomonas aeruginosa Virulence. Virulence 12, 1469–1507. https://doi.org/10.1080/21505594.2021.1926408

Nordmann, P., Poirel, L., 2002. Emerging carbapenemases in Gram-negative aerobes. Clin Microbiol Infect 8, 321–331. https://doi.org/10.1046/j.1469-0691.2002.00401.x

Reboud, E., Bouillot, S., Patot, S., Béganton, B., Attrée, I., Huber, P., 2017. Pseudomonas aeruginosa ExlA and Serratia marcescens ShlA trigger cadherin cleavage by promoting calcium influx and ADAM10 activation. PLoS Pathog 13, e1006579. https://doi.org/10.1371/journal.ppat.1006579

Vigeant, P., Loo, V.G., Bertrand, C., Dixon, C., Hollis, R., Pfaller, M.A., McLean, A.P.H., Briedis, D.J., Perl, T.M., Robson, H.G., 1998. An Outbreak of Serratia marcescens Infections Related to Contaminated Chlorhexidine. Infection Control & Hospital Epidemiology 19, 791–794. https://doi.org/10.1086/647728

| 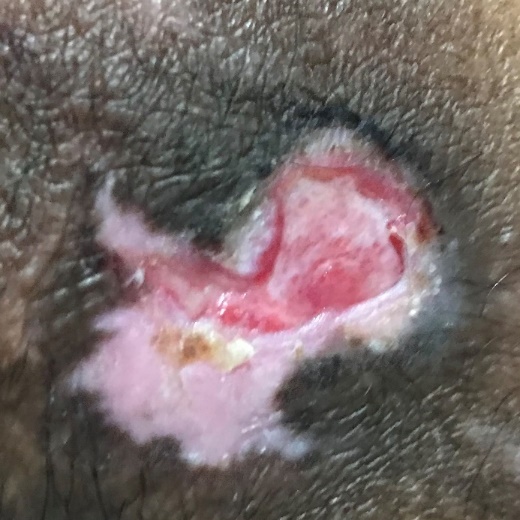  **1A** | 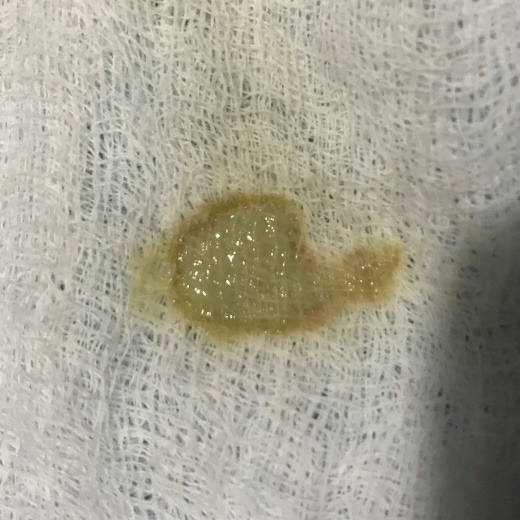  **1C**  **1B** | 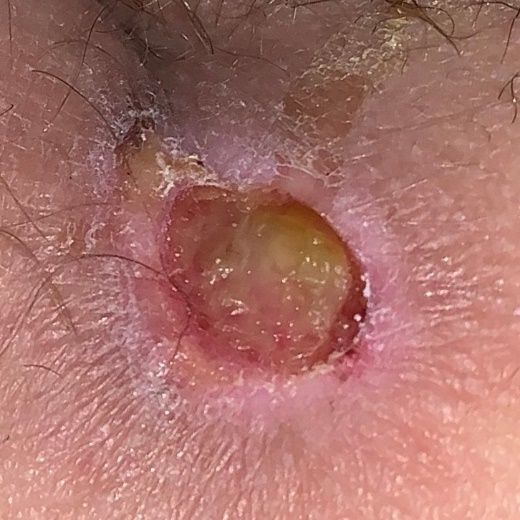 |
| --- | --- | --- |
| 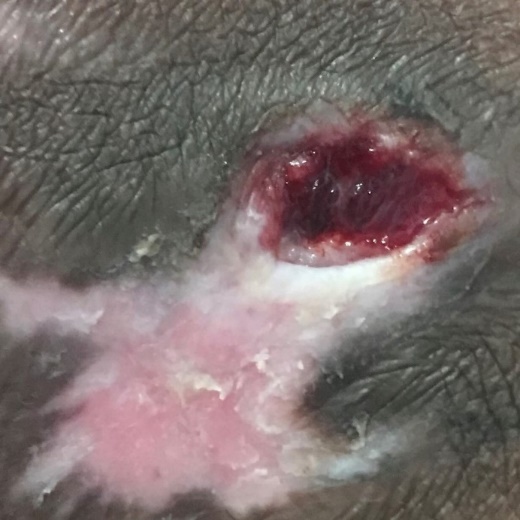  **2A** | 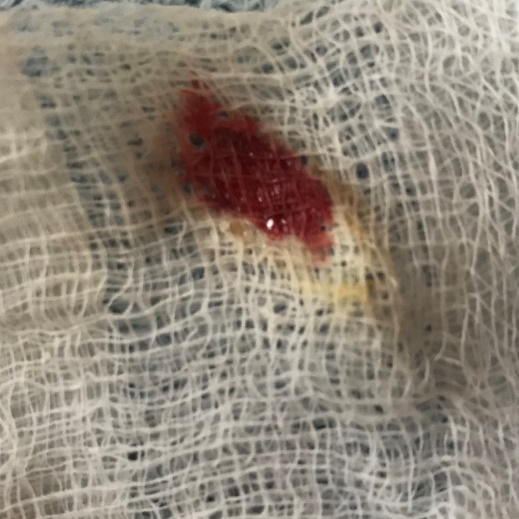  **2B** | 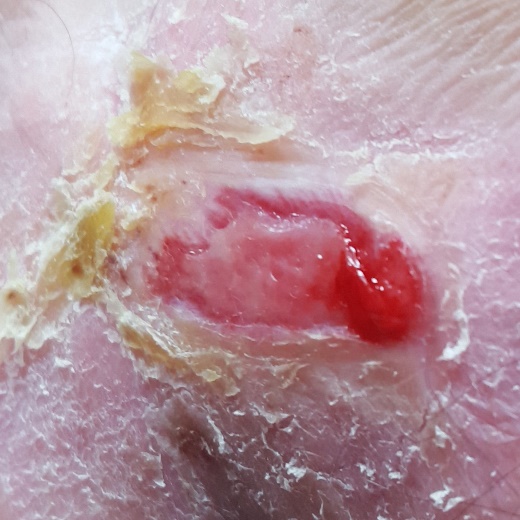  **2C** |
| 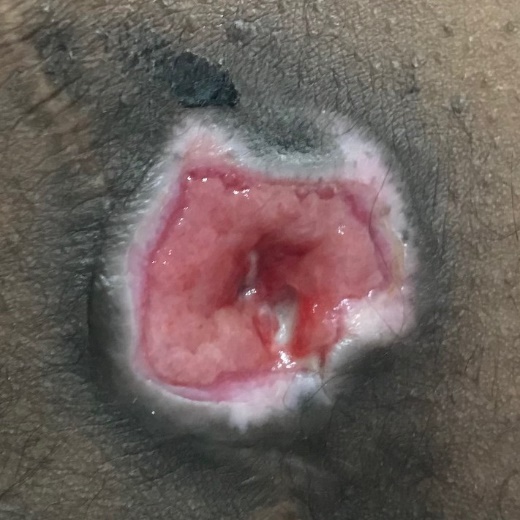  **3A** | 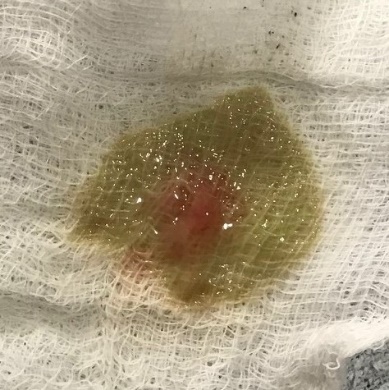  **3B** | 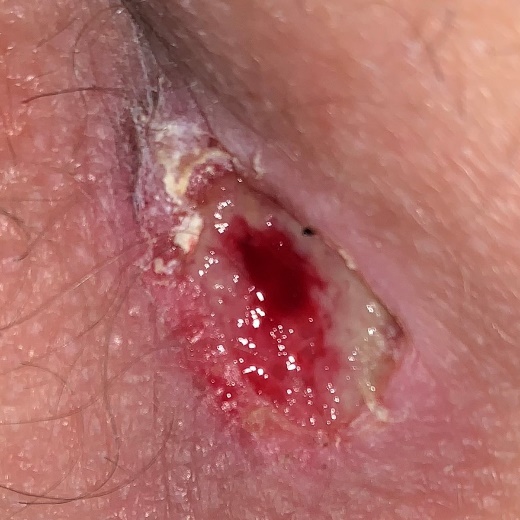  **3C** |
| **Figure 1.** The pictures in column A are of the same wound on different days. Column B shows the corresponding simple 100% cotton gauze dressings removed after having covered the wound for 24 hours. The pictures illustrate that the level of exudate is very low and that most of the expelled material caught in the gauze is pigmented toxins produced by the bacterial strain dominating the wound microbiome at that point in time. The Green colour dominating in row 1 is characteristic of *Pseudomonas aeruginosa* and the red colour of row 2 is typical for *Serratia marcescens*. Row 3 shows that two species can dominate different fields of the wound, often without mixing. The wounds in column C have been directly exposed to air without a secondary covering dressing for the past 24 hours. These have undergone a similar expulsion of toxins from the infected wound tissue onto the wound surface where the toxins remain and can be washed off with tap water. | | |
