# Supplementary material for: Stable closure of acute and chronic wounds and pressure ulcers and control of draining fistulas from osteomyelitis in persons with spinal cord injuries: non-interventional study of MPPT passive immunotherapy delivered via telemedicine in community care
Source: Front Med (Lausanne). 2024 Jan 5;10:1279100. doi: 10.3389/fmed.2023.1279100 (PMC10797031; doi:10.3389/fmed.2023.1279100)
Supplement: Supplementary file 8 [file Data_Sheet_8.pdf]

S8 – Analysis of chronic grade 3 and 4 wounds and ulcers

Number	Grade	Wound Age	Location	Cause	Patient	Outcome
22	4	6 weeks	Ischial tuberosity	Pressure	T10 complete	Closure
23	4	9 weeks	Ischial tuberosity	Pressure	C4/5 incomplete	Closure
24	4	11 years	Sacrum	Pressure	T9/10 & L1/2 complete & head injury	Closure
25	4	6 months	Ischial tuberosity	Pressure	C5/6 complete	Closure
26	3	2 years	Sacrum	Trauma	C5 complete	Closure
27	3	11 weeks	Ankle	Unknown	C5/6 incomplete	Closure
28	3	12 years	Ischial tuberosity	Pressure	L1 incomplete	Closure
29	3	3 years	Sacrum	Pressure	C5 complete	Closure
30	3	3 months	Ischial tuberosity	Pressure	T8/9 complete	Closure
31	3	15 months	Sacrum	Pressure	C5 complete	Closure

“**Day 0**” always equals the first day of MPPT application.

Wound number 22			Patient	SCI	
**Grade 4**	**6 weeks old**	**Ischial tuberosity**	**41-year-old male**	**15 years**	**T10 complete**

Two months prior to the appearance of the present pressure ulcer, the patient had had a toe amputated due to osteomyelitis caused by a wound. Leading up to amputation he had had several courses of several weeks' duration of three different antibiotics, oral and IV. This is likely to have reduced the diversity of the patient's general microbiome and selected for an antibiotic resistant and accordingly virulent composition of the species that make up the patient's microbiota.

This sore was detected 6 weeks earlier. It appeared to be a "true" pressure ulcer originating as necrotising soft tissue at the ischial tuberosity gradually extending to the skin surface (pic 1). Necrotic tissue is the ideal breeding ground for microorganisms and acted as a highly viscous, fixed plug between skin and bone. The wound was treated daily with a combination of cadexomer iodine and gel (Iodoflex) and covered with an occlusive secondary dressings. The indwelling necrotic tissue plug remained firmly fixed between the bone and the skin, and its comprised infection spread into the adjacent soft tissue expanding the wound (pic2). The necrotic tissue plug underwent further fixation by an expanding weave of fibrin; visible on top of it (pic 3). The excessive amounts of fibrin also infiltrated the adjacent tissue accompanying the spreading infection, thereby expanding the wound further (pic3). This is when Amicapsil treatment was started (pic 3).

The wound rapidly contracted, started epithelializing along the edges, and actively began removing the necrotic plug by supporting the processes of autolytic debridement (pic 4). The wound also divided into two main fields with regards to microbial imbalance, with the area characterised by fibrin seemingly dominated by a species producing green pigmented toxin, e.g. *Pseudomonas aeruginosa*, and the area characterised by the plug, by a bright-red pigment producing species, such as *Serratia marcescens* (pic 5 & 6). The plug finally gave way definitively (pic 6) and the tunnel void was immediately filled with granulation tissue (pic 7 & 8). Epithelialisation, from the edges and as islands, progressed simultaneously with the microbial imbalances being fought whilst the wound continued divided into the two fields (pic 8 & 9) until closure. A scab developed over the yet to epithelialize area of the wound (pic 10) and gradually fell off at the rate at which each area beneath it healed and matured enough to let go of this protection. (pic 11). Even at full wound closure, i.e. wound 100% epithelialized, the original division into two fields remained, showing the former necrosis-filled tunnelled area as purple and the former fibrin dominated area as red (pic 12). The maturation phase continued steadily and eventlessly for months after closure (pic 13).

As a general remark, the sore closed well and without setbacks and yet it seemed unusually pertinacious which could potentially be due to the many courses of systemic antibiotic shortly prior to the occurrence of the present sore.

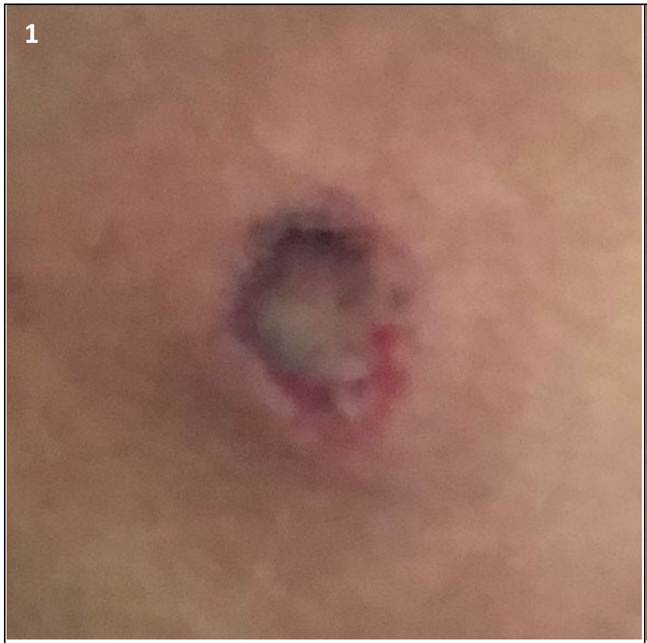

Day minus-37

5.5 weeks before start MPPT

Ulcer detected. Already necrotic with evident microbial imbalance.

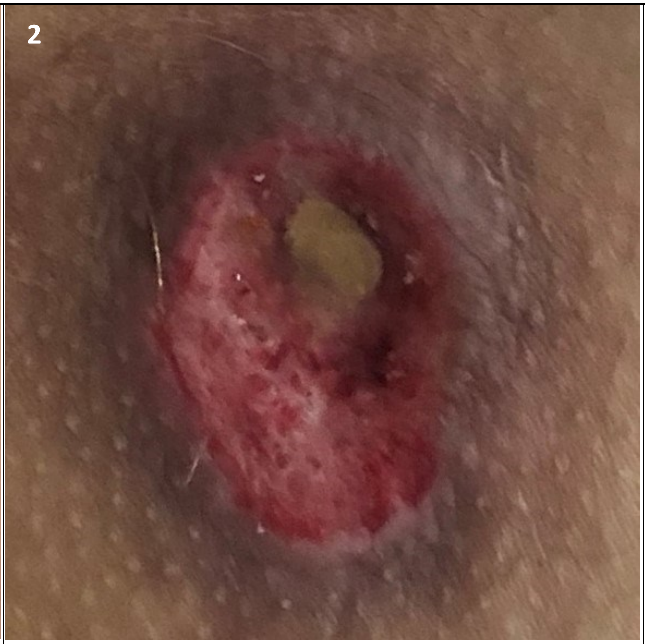

Day minus-15

2 weeks before start MPPT

A plug of dried out necrotic tissue has settled in the track between the osseous tuberosity and the skin surface. Necrosis harbours excessive levels of microbes, causing infection to spread and break down the surrounding soft tissue.

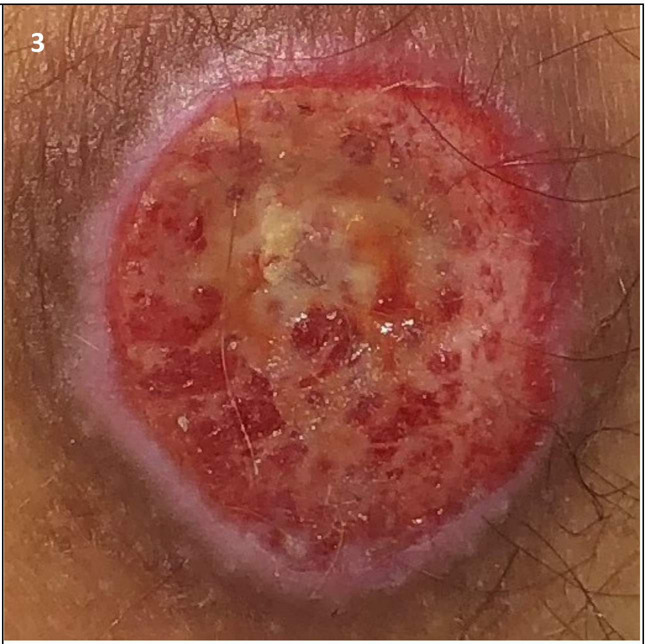

Day 0

Just before first MPPT

Approximately 40 x 40 mm.

Excessive fibrin interweave fixates the plug further. Also, fibrin travels in parallel with the spreading infection into the adjacent tissue.

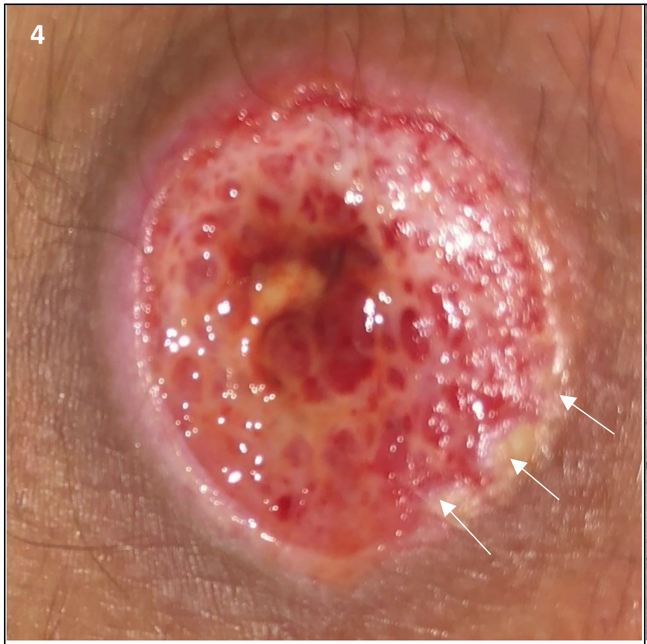

Day 7 1 week

String of granulation buds along the wound edge with actively epithelializing wound edges.

The plug is being removed by means of autolytic debridement.

Arrows: Patches of new epithelium.

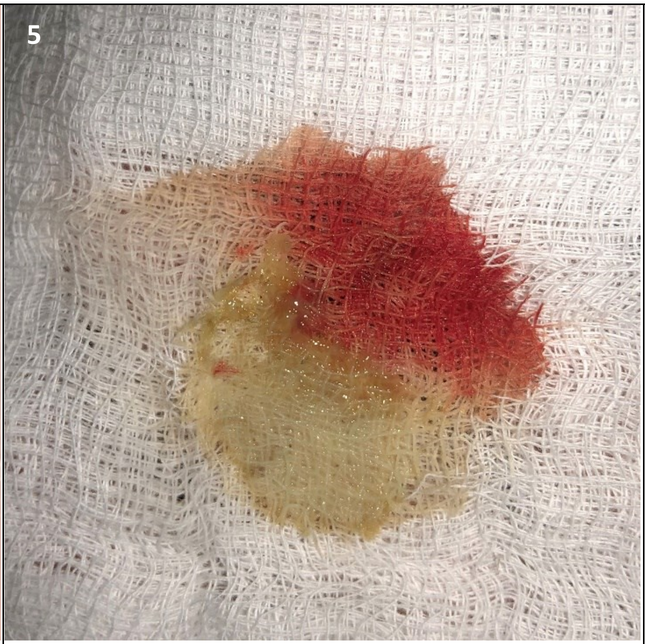

Day 14 2 weeks

Dressing removed from wound reflecting the wound separation into two separate, distinct fields characterised by green and red pigmented toxins, respectively. (The red is pigment, not blood.)

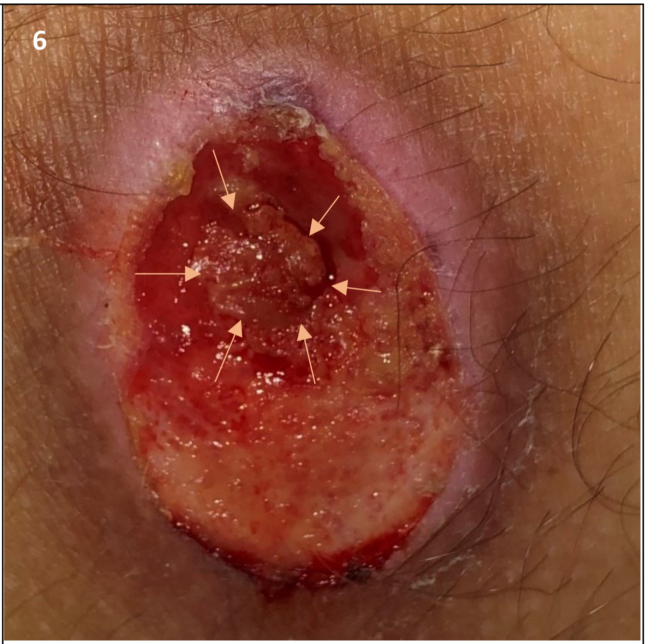

Day 22 3 weeks

The separation of the wound in two main fields, is reflected in the wound surface and remains throughout healing.

The plug is clearly coming loose. It will be pushed out over the coming days.

Arrows: Groove demarcation of plug.

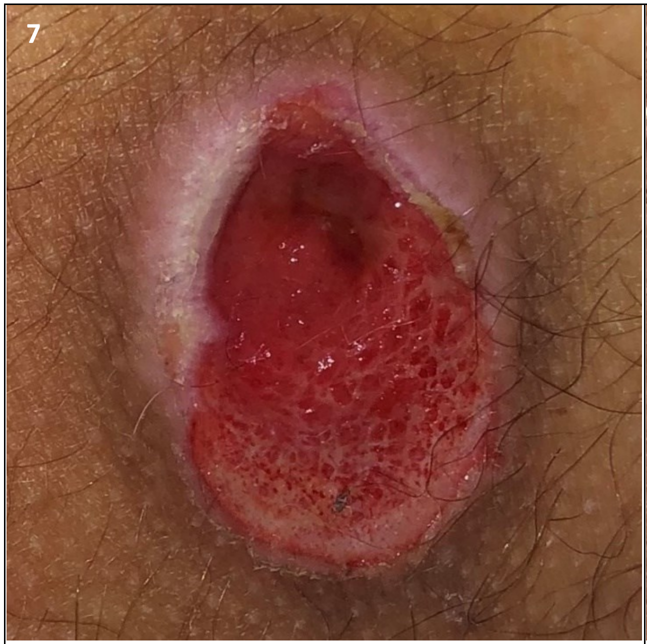

Day 29 4 weeks

The plug has been definitively removed and substituted by granulation tissue of an inordinately red colour.

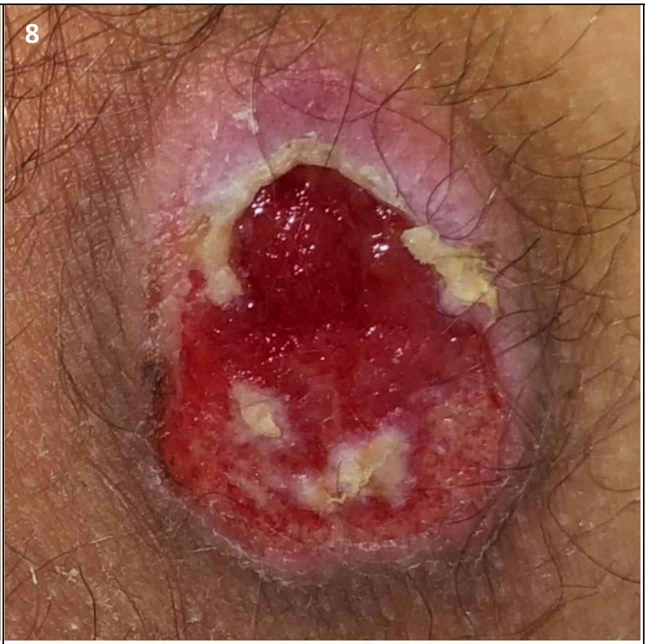

Day 53 1.5 months

Epithelium moving in from the edges as well as forming as islands in the wound bed.

The division into two fields remains throughout the healing process.

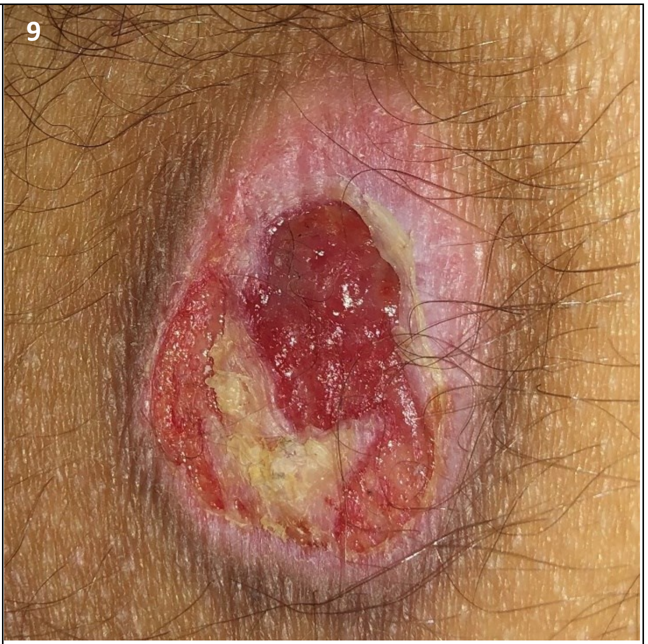

Day 62 2 months

Infection removal occurs simultaneously with tissue regeneration and epithelialisation.

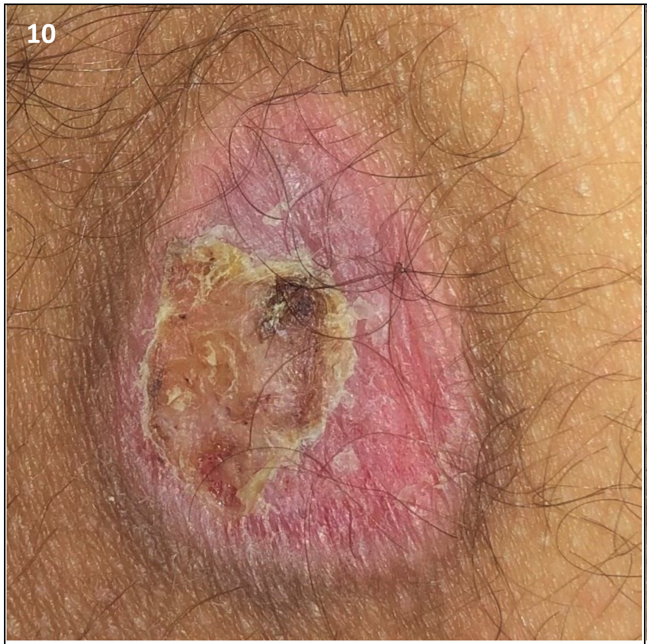

Day 93 3 months

Entire wound area protected by either new epithelium or a healthy scab.

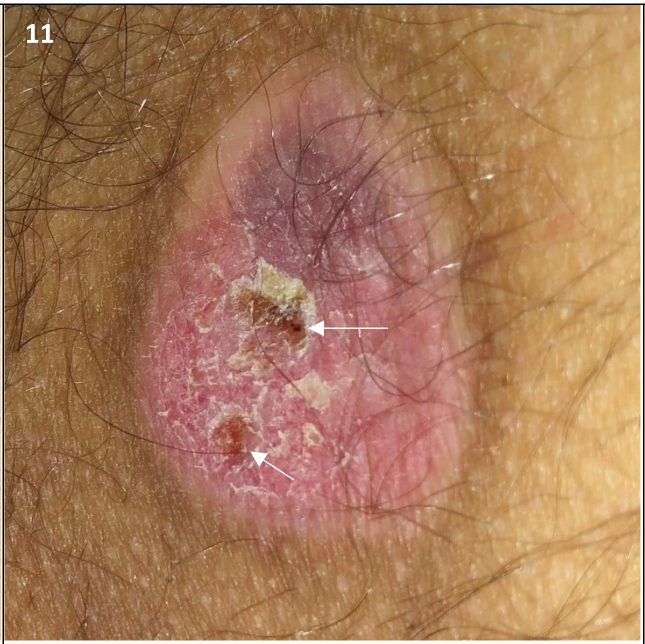

Day 124 4 months

The scab falls off as the epithelium underneath matures and releases it.

Arrows: Scab still attached

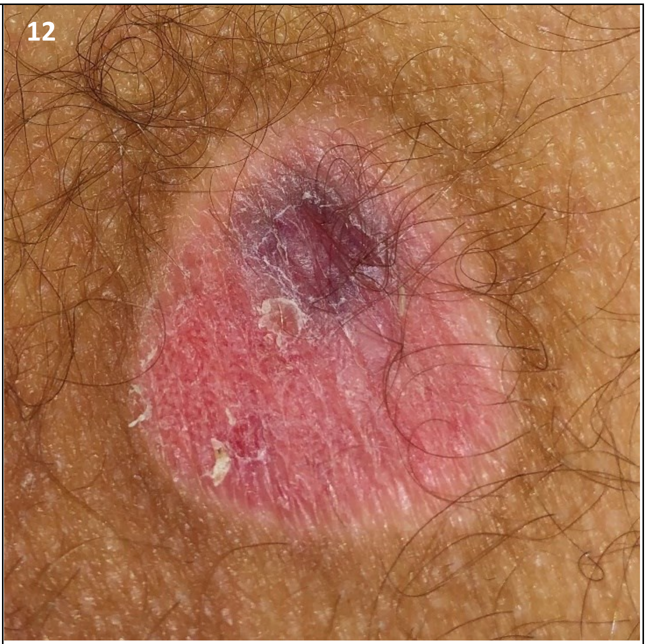

Day 146 4.5 months

Full closure

100% epithelised

The separation into two fields remains visible.

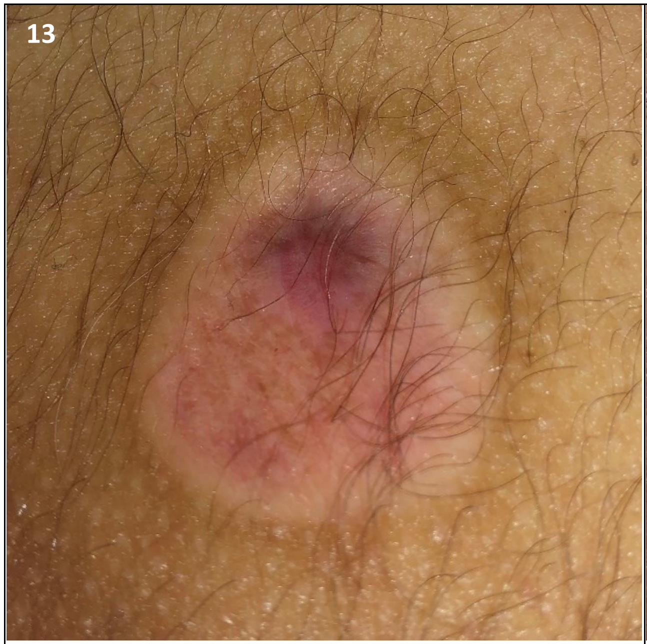

Day 223 7.5 months

77 days after full closure

Regeneration progressing well with the wound maturing and gaining strength with no issues.

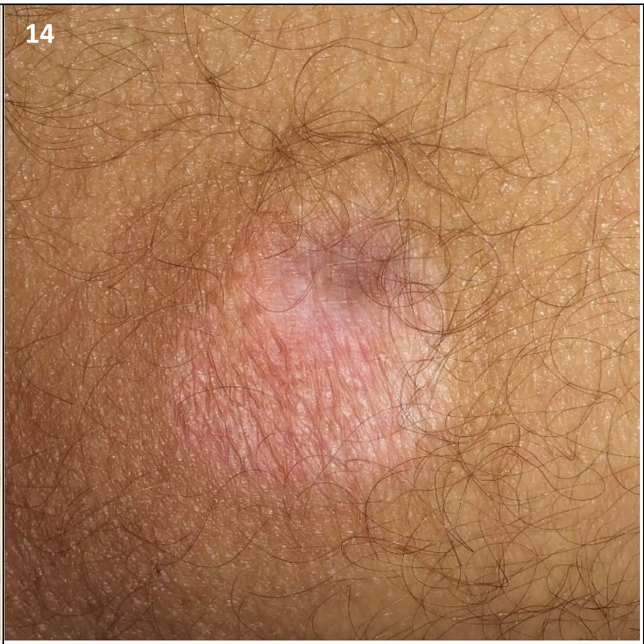

Day 431 14 months

10 months after full closure

The scar tissue is gradually fading as the tissue matures into what increasing looks like the original natural tissue type.

Wound number 23			Patient	SCI	
**Grade 4**	**9 weeks old**	**Ischial tuberosity**	**34-year-old male**	**8 years**	**C4/5 incomplete**

While in hospital with the original spinal injury, the patient developed a stage 4 pressure ulcer which, 5 months later, was surgically closed.

7 years later, an ulcer appeared in the scar line in the same place in connection with weight loss and the patient being confined to bed for an unrelated condition. This consequently raised well founded doubt whether the wound was caused by pressure, making it much more likely to be due to one or more old abscesses, from the original wound and extensive surgery, bursting as the patient was going through an overall immune- challenging period. The infective material inside the abscess would subsequently, while infecting the surroundings it passed, have made its way to the surface of the body before finally breaking through the skin creating a small, but potentially deep, wound.

After 2 months' weekly dressing with Manuka Honey sheets (Medihoney) under an absorbent foam dressing (Allevyn), the ulcer had grown to the size of 45mm x 30mm with no undermining. The central area was severely infected and extensive viscous slough prevented the inspection for potential tunnelling and for evaluating the full depth (pic 1). Treatment was changed to MPPT (pic 2). The wound rapidly cleaned out all slough via autolytic debridement including the deep central part which had been impossible to evaluate (pic 3). The deep central part of the wound was expelling red toxins (typical for *Serratia marcescens*) and the rest of the wound green toxins (typical for *Pseudomonas aeruginosa*) (pic 4). The wound granulated and epithelialized quickly and efficiently with no signs of trouble (pic 5 & 6) until the patient went into hospital for a few days for a routine check. Here the wound was cleaned daily with the antimicrobial, Prontosan (PHMB and surfactant). This very efficiently changed the microbial composition and shut down the healing process (pic 7). The wound microbiome had been permanently changed. Antimicrobials reduce the microbial diversity needed by the immune system to achieve the microbial balance required for healing. They favour the resistant bacterial strains which, in addition, are known to be more virulent (increased ability to infect, expand and spread) than their non-resistant counterparts (Bengoechea & Pessoa 2019). Within a week, the central part of the wound, which from start of MPPT and throughout the healing process had been dominated by the red-toxin-producing microbe, displayed a demonstration of empowered bacterial motility and invasion (pic 8) (Bengoechea & Pessoa 2019). This caused a delay but was seemingly mitigated as the wound practically closed 2 months from start of MPPT treatment (pic 9).

Nonetheless, during the following 8 months, the wound would go through several closing cycles (pic 10) seemingly caused by the high degree of virulence that the antimicrobials had created in the main infective bacterial strain.

Several instances were seen of evasive behaviour, even beyond the area of the original wound bed, where bacteria would suddenly "shoot" out dark streaks under the skin (pic 11), from the focus of the wound to expand and infect in the vicinity. These attempts to evade the immune system (Bengoechea & Pessoa 2019) were controlled and the ensued tissue damage was healed (pic 12 – 15).

Other instances were observed of red-pigmented bacterial toxins, intended to cause tissue damage and inhibit the immune cells, being collected and accumulated by the immune system. These clusters were then moved towards the surface with the increasing closeness to the epithelium seen as an increase in purple darkness (pic 16). The epithelium would eventually open, and the collection of toxins would be expelled over one or very few days (pic 17 & 18). Red expulsions were usually followed by a rapid build-up of the deep lost soft tissue and a relatively longer period of clear green pigment expulsion from a seemingly shallow wound bed (pic 19). The damaged area would then epithelialize.

Such instances would occur a few times with the wound seemingly closed for an increasing number of days between each incident, indicating that the infection was gradually losing momentum. These instances were presumably the clinical picture of the process of limiting and stopping the evasive and virulent behaviour of the primary infective agent.

Eventually, 10 months after start of MPPT, the wound definitively closed (pic 20) and remained closed and remodelling well. (pic 21).

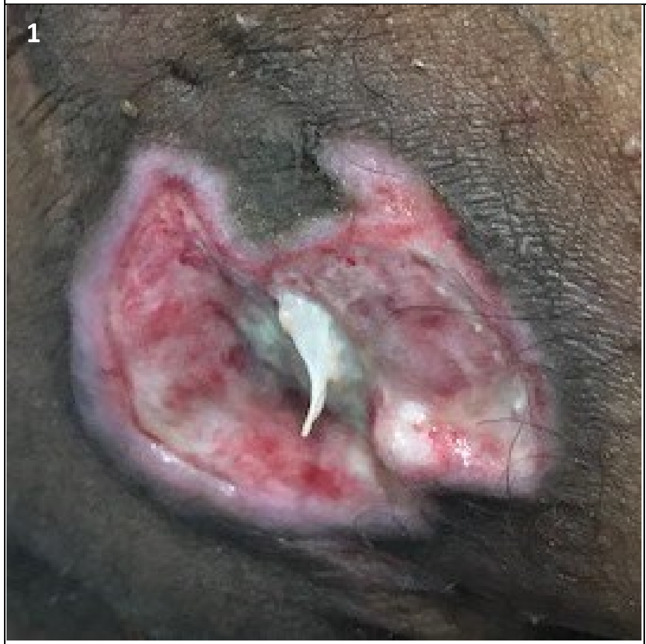

Day minus-7

1 week before start MPPT

Viscous slough in heavily infected central area

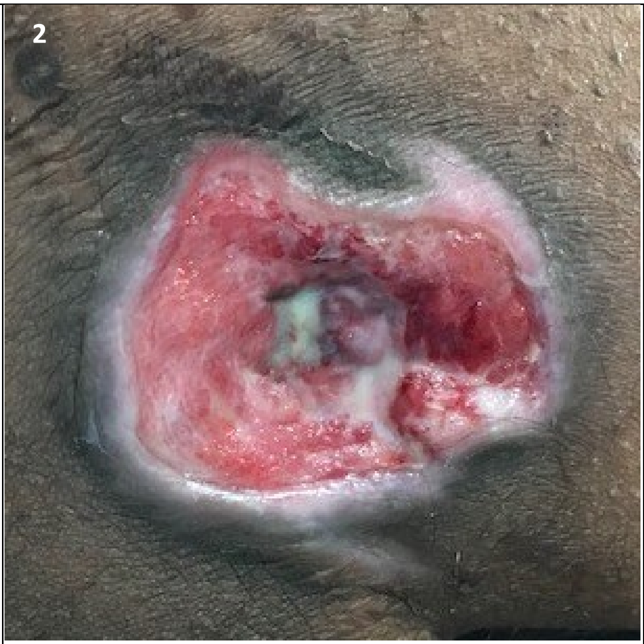

Day 0

Just before first MPPT

45 x 30 mm

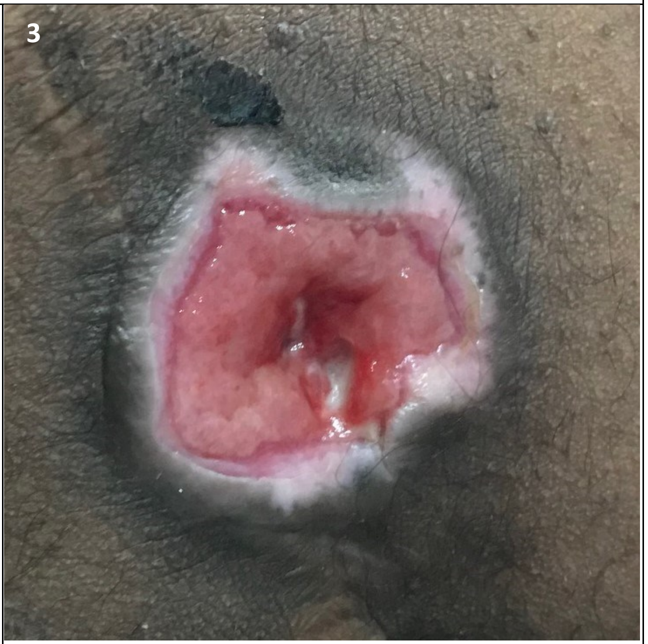

Day 11

Healthy wound epithelializing edges with a string of granulation pearls along the entire inside wound edge, i.e. the border of the wound bed.

Central area is being cleaned of slough and infection and is regenerating.

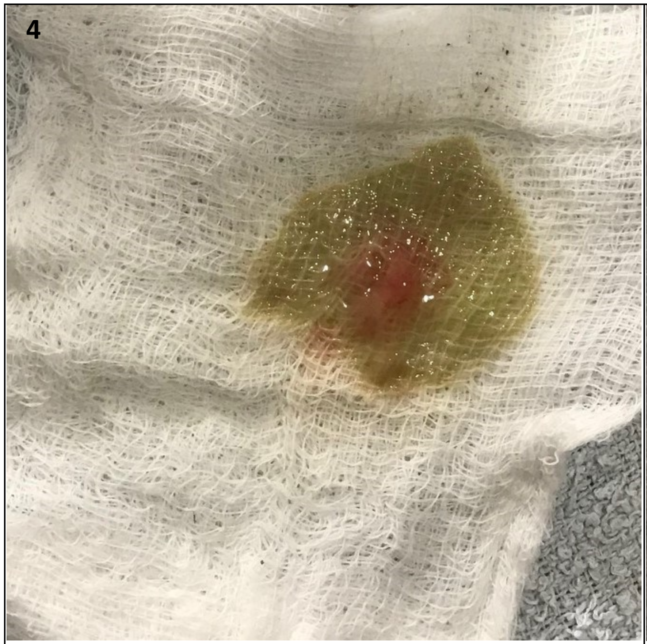	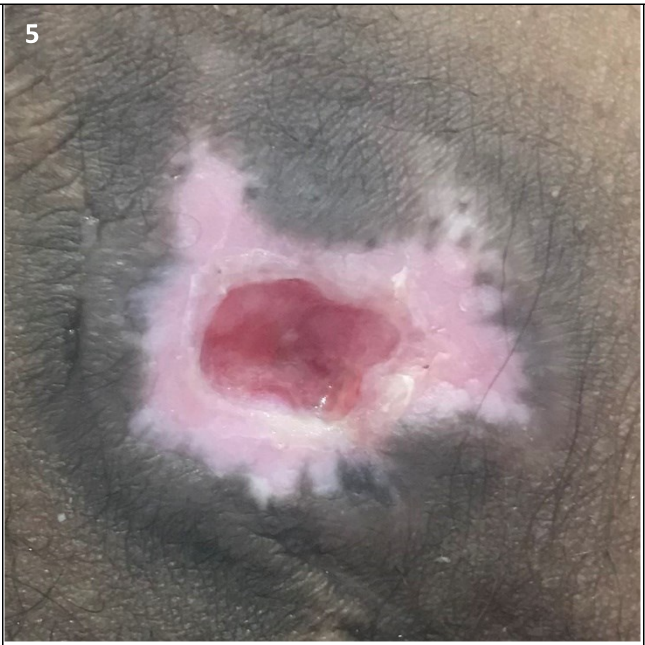	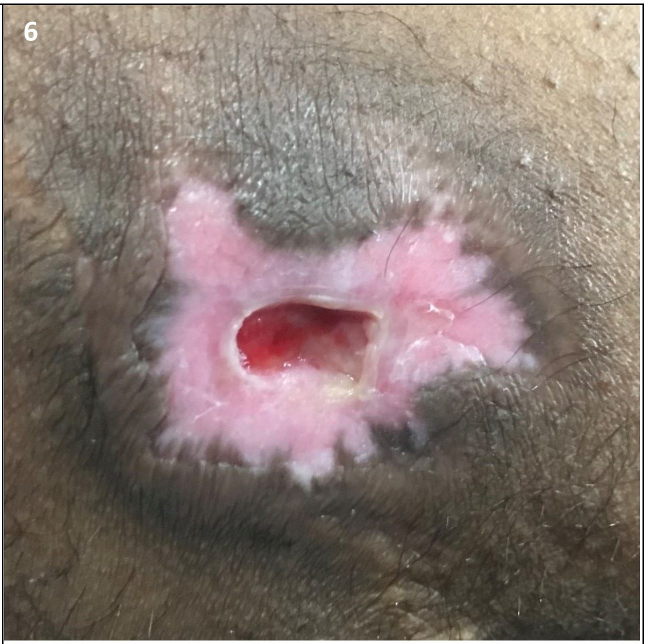
**Day 11**<br>**Removed dressing**<br>Example of corresponding secondary dressing worn 24 hours. Green and red pigment neatly deposited in the gauze. Position suggests the location of the respective bacterial species in the wound.	**Day 25**<br>Infection is controlled. The wound is epithelializing and granulating including the central deepest part of the wound which is now clean.	**Day 32**<br>Wound closing fast.

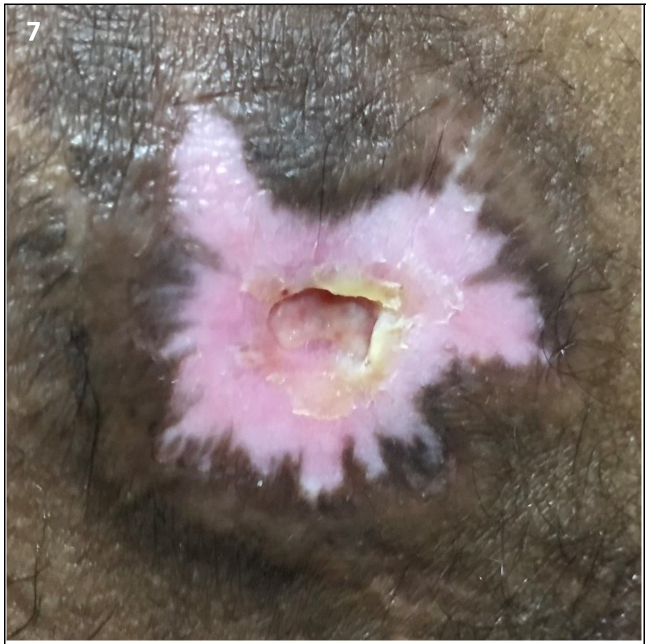

Day 38

Picture is taken after washing.

Wound bed and edges are damaged.

Healing is temporarily halted.

The microbial composition of the microbiome has been changed. This was caused by applying antiseptics (PHMB & surfactants) to healthily healing tissue. This brought about a change in the behaviour of the infection, rendering it more evasive and more aggressive.

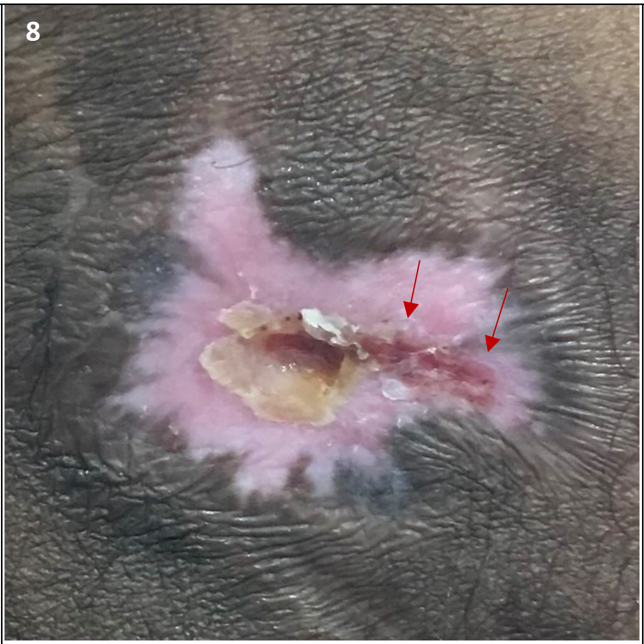

Day 44

Increased bacterial virulence is seen as increased motility and invasiveness.

Red arrows: Infection “shoots” out from the main focus in the wound.

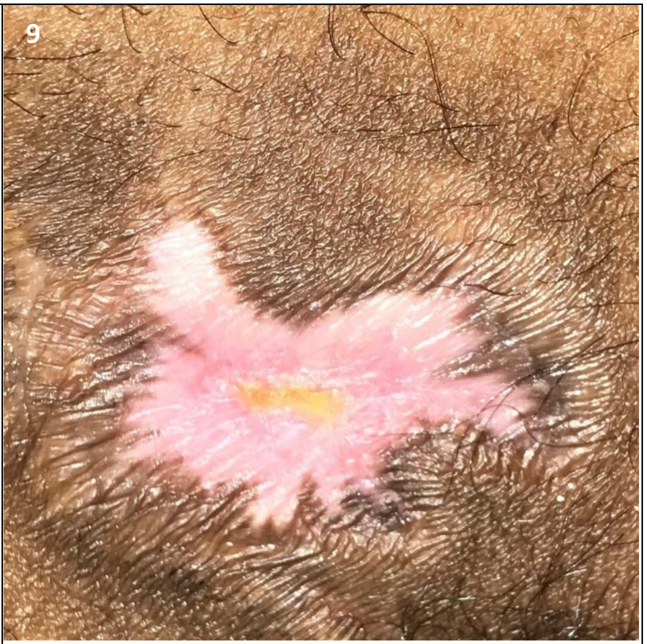

Day 57

Seemingly closed

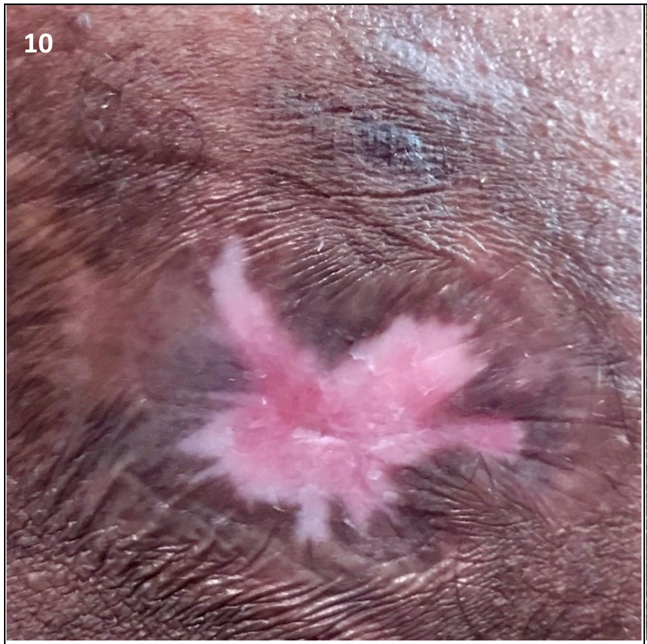

Day 102

In many instances the wound seemed closed. But bacterial evasiveness caused repeated upset.

On very close inspection, 0.1 x 0.1 mm opening can be detected in the centre.

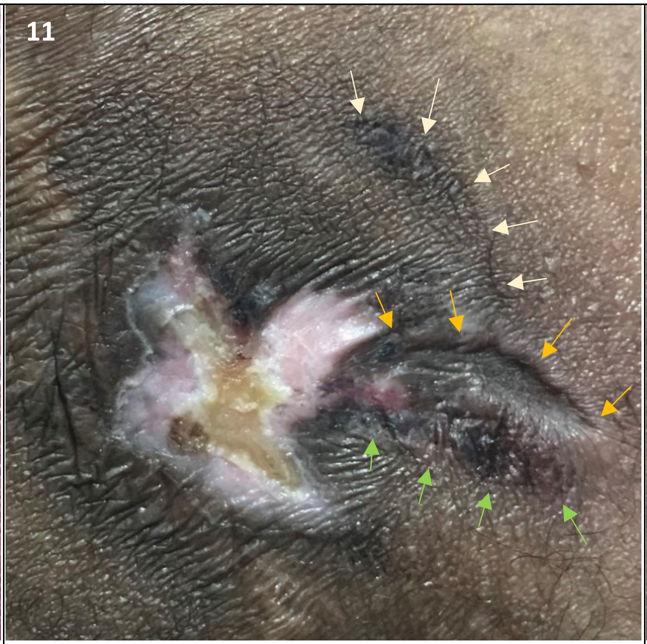

Day 139

Evasive bacterial behaviour peripherally to the original wound bed – to avoid the immune response.

Seen as two parallel dark broad streaks under the intact skin and a streak moving above the wound recognisable as a black spot.

Orange arrows: Upper parallel streak.
The skin of the upper streak is not broken but sucked into a hollow trench. This is the initiation of a peripheral "shoot". It is seen only for max 24 hours and often dismissed as an "optical illusion".

Green arrows: Lower parallel streak.
The lower streak is hours ahead in its development, having levelled with the skin surface and turned the skin very dark.

Pale arrows: Dark mark.
The dark mark is presumably a third streak travelling deeper in the soft tissue with the dark mark reflecting it getting closer to the surface.

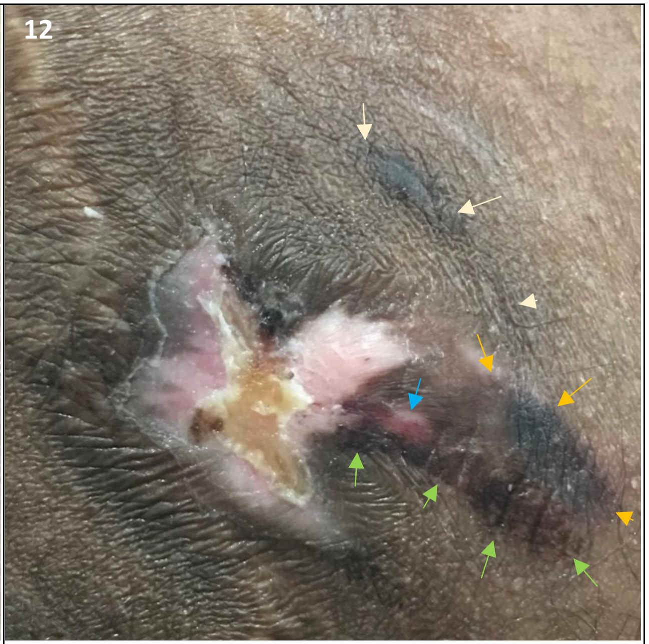

Day 140

The discolouration is presumably caused by a high concentration of red-pigmented toxins moving in the soft tissue and possibly by non-viable soft tissue damaged during the bacterial offensive. Such streaks will often be dismissed as "irregularities of the skin" but will, if ignored, cause severe tissue break-down.

Orange arrows: Upper parallel streak

Green arrows: Lower parallel streak

Pale arrows: Dark mark

Blue arrow: Skin breakage.

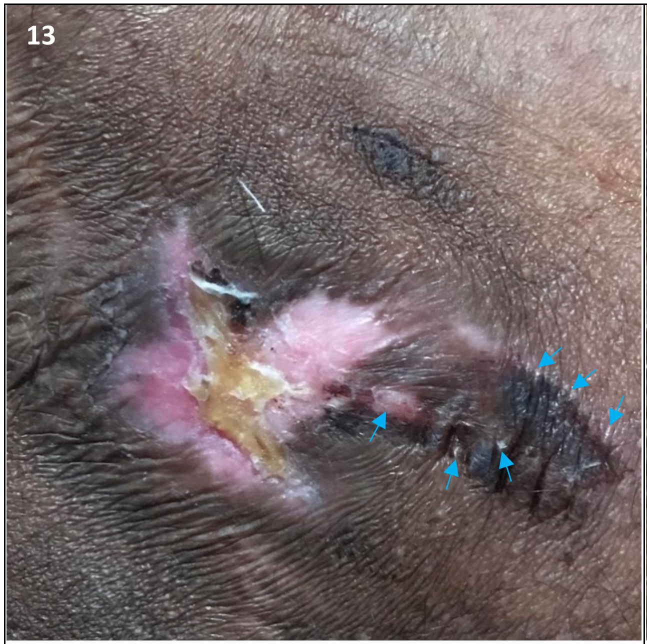

Day 143

The skin on top of the two parallel streaks is damaged from within and breaks ever so slightly in some places but the infection is mainly fought underneath and within the skin.

Notice how the focus of the infection, i.e. the original wound bed, is also engaged in the ongoing battle.

Blue arrows: Skin breakage

Orange arrows: Upper parallel streak

Green arrows: Lower parallel streak

Pale arrows: Dark mark

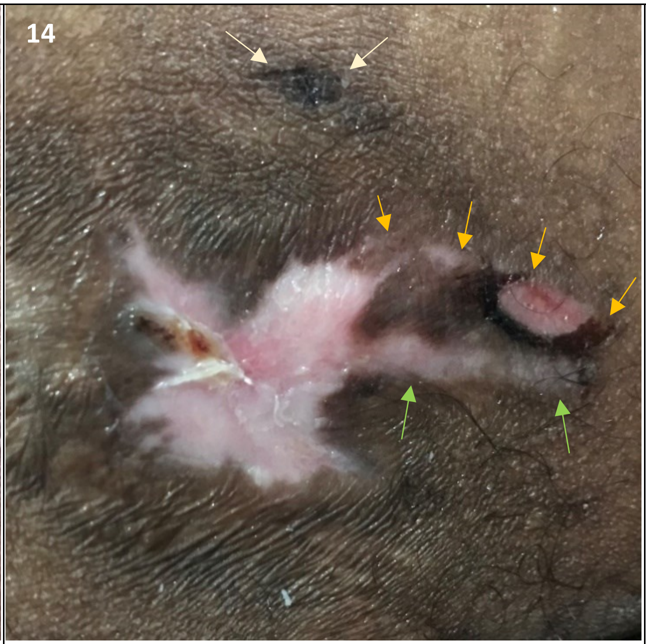

Day 148

The damaged soft tissue and skin of the lower streak is now healed. This leaves behind a pink scar with skin pigment still to move in.

The upper streak is well contained and healing.

The dark spot above the wound is reducing.

Orange arrows: Upper parallel streak

Green arrows: Lower parallel streak

Pale arrows: Dark mark

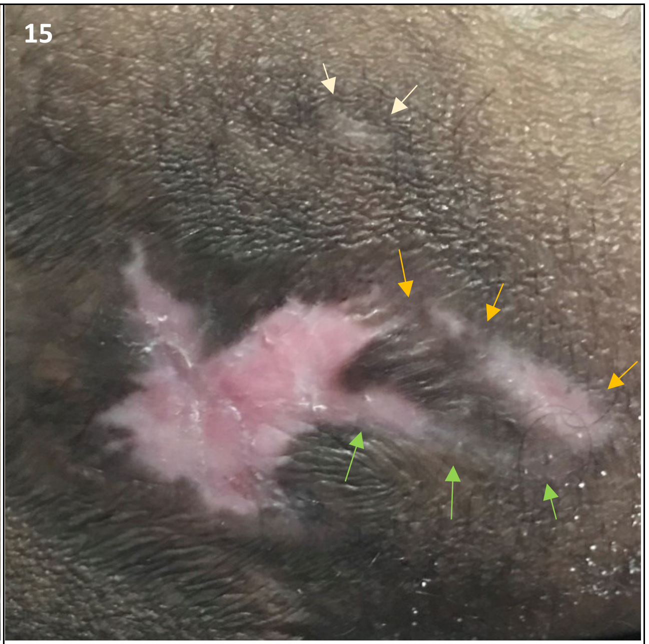

Day 154

Both streaks and the spot above the wound are now healed and converted into pink scar tissue in the process of restoring pigmentation.

The whole area seems clear and closed.

Orange arrows: Upper parallel streak

Green arrows: Lower parallel streak

Pale arrows: Dark mark

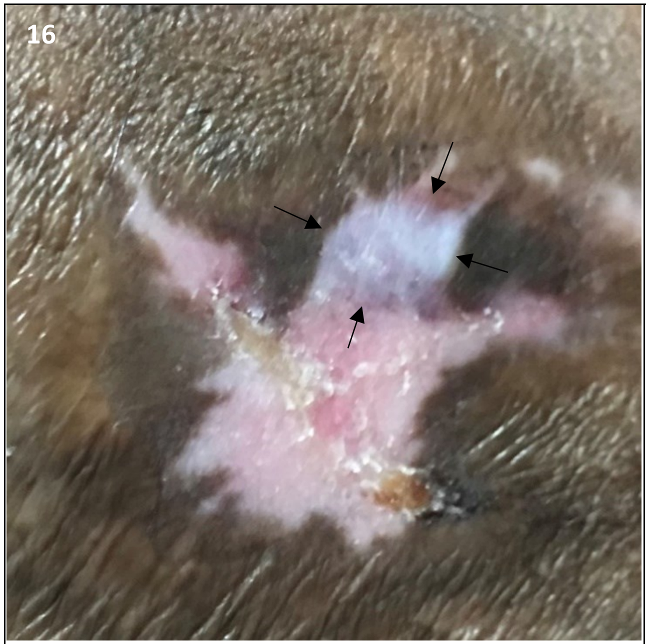

Day 159 – closeup

Red pigmented toxins being collected and accumulated seen as discoloured concentrations under the new epithelium. Gradually, they are be pushed towards the skin surface for expulsion.

Black arrows: Collection of toxins under skin.

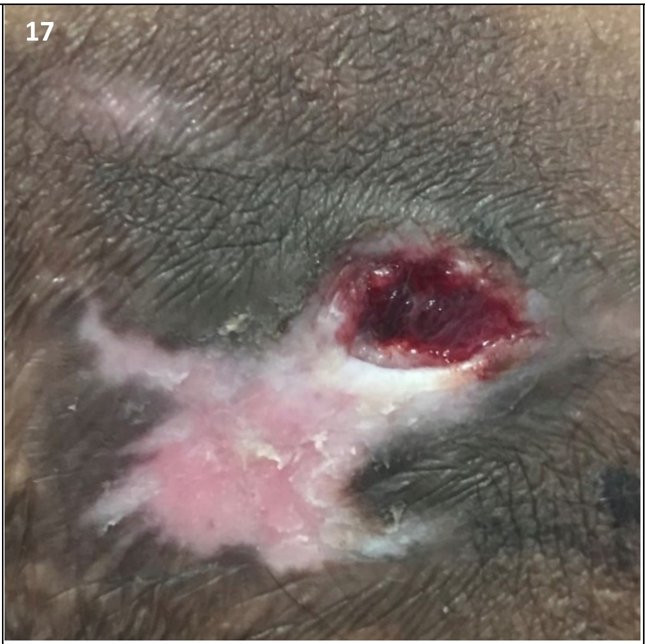

Day 179 - closeup

The dark purple collection has travelled to the skin surface and is currently in the process of expelling the red toxins. It will be out in a couple of hours.

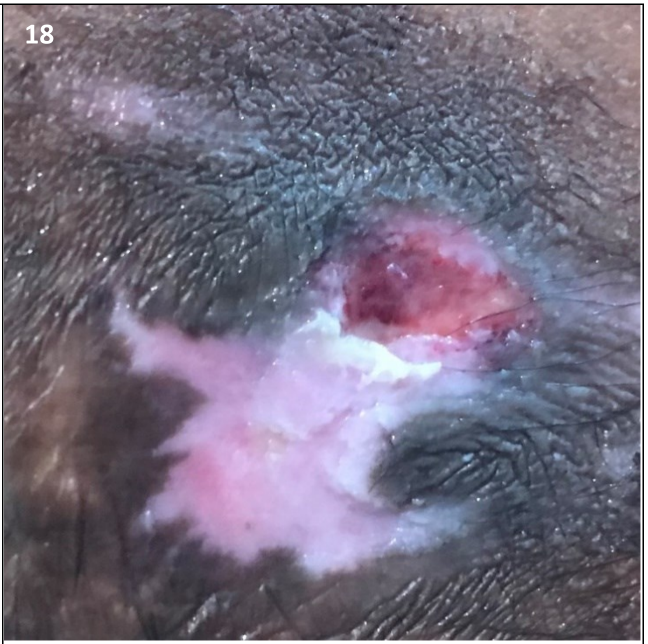

Day 180 - closeup

Following expulsion. The collection of the red toxins is almost empty.

With every incident, the time, spent on getting the dark collections under control and expelled, reduces - indicating that the infection is gradually being defeated.

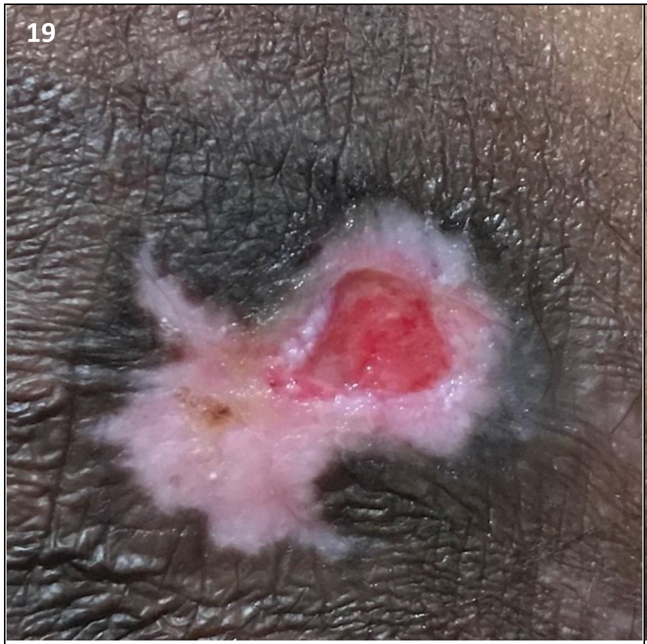

Day 196 - closeup

A shallow wound bed associated with expulsion of green pigmentation over more days would typically follow the shorter but more forceful, red-pigmented expulsions.

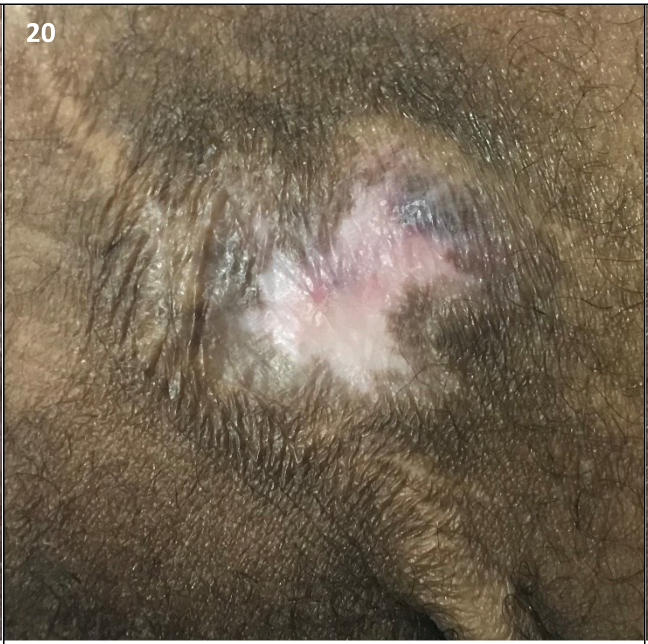

Day 307 10 months

Definitively closed

MPPT was discontinued 3 weeks prior. The wound is lastingly closed. Regeneration and maturation of the soft tissue and the new skin are levelling the scar with the body’s surface and increasing its strength.

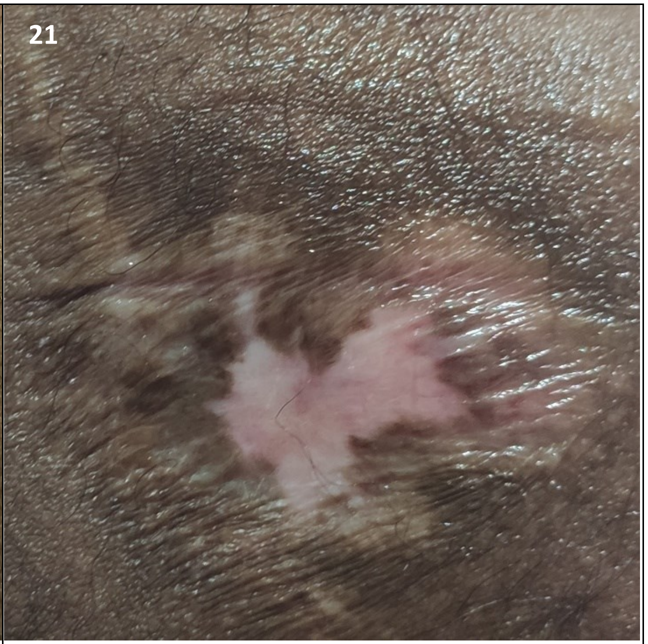

Day 359 12 months

Wound remains well closed. Steady remodelling taking place with the scar steadily reducing in size and increasing in tensile strength.

Wound number 24			Patient	SCI	
**Grade 4**	**11 years old**	**Sacrum**	**47-year-old male**	**24 years**	**T9/T10 & L1/L2 complete & severe head injury**

This ulcer, which had been present in some form and treated for the past 11 years, was located central-right of the midline. The patient had furthermore and more recently acquired a trochanter pressure ulcer on his left side. This had left him in hospital with sepsis for 4 weeks receiving IV Benzylpenicillin and Flucloxacillin and oral Metronidazole and topical Metronidazole cream on both wounds. The hospital-stay focused on stabilising the generalised infection (sepsis) and neither of the wounds nor the osteomyelitis, which were causing the septic state, were addressed surgically whilst in hospital. Upon discharge, the patient continued the antibiotic Flucloxacillin uninterrupted for the following 16 months and was also prescribed Omeprazole for wound healing. The wounds were treated daily with antibiotic Metronidazole cream and dressed with silicone foam dressing (Mepilex border). Since they continued to deteriorate, it was decided to treat the sacrum sore with MPPT whilst keeping the left trochanter sore on conventional packing and dressing. During this time, the left trochanter sore continued to deteriorate substantially and its many sinuses grew wider and longer, with one, of particular relevance to the sacrum sore, making its way towards the sacrum and gradually infecting and destabilising the deep soft tissue to the left of the midline. In addition, the uncontrollable amount of exudate coming from the trochanter sore, despite daily changes of packing and highly absorbent dressings, would cause the dressings to fall off regularly and leak the infectious debris into the sacrum sore. This would destabilise the microbiome, and thereby re-infect the otherwise healing sacrum sore and/or the adjacent areas. The patient was also prone to bladder accidents, and it was observed, that his urine impacted the sacrum sore with an atypical forcefulness to promptly exploit any imperfection in the old scar tissue to dig in and re-infect.

At the start of MPPT treatment of the sacrum wound, the wound itself as well as the old scar tissue in the area had a shiny, dry, dull and inactive appearance (pic 1). During the first few weeks, the wound would regularly expel concentrated red and some bright yellow pigment (pic 2). After 3 weeks (pic 4), the wound was considerably smaller and covered with a healthy, thin protective scab that would lift and fall off in small pieces along the edges as the latter epithelialized underneath. This healing pattern is typical in chronic wounds in persons with SCI and it is therefore likely that this process would have continued, leading directly to durable closure.

However, due to the repeated cross-contamination from the heavily infected trochanter sore and the relatively frequent urine leaks, the infection would repeatedly re-establish itself in still fragile, immature scar tissue. This would cause the infection to move around within the scarred area that had been weakened over the past 11 years and was likely to be harbouring a sub-optimal microbiome (pic 5). After 5 months, the original wound location was fully closed (pic 6). The infection had had the opportunity to relocate to both the left and right of this original area. By 7 months, the entire sacrum area was cleared of infection and closed (pic 7).

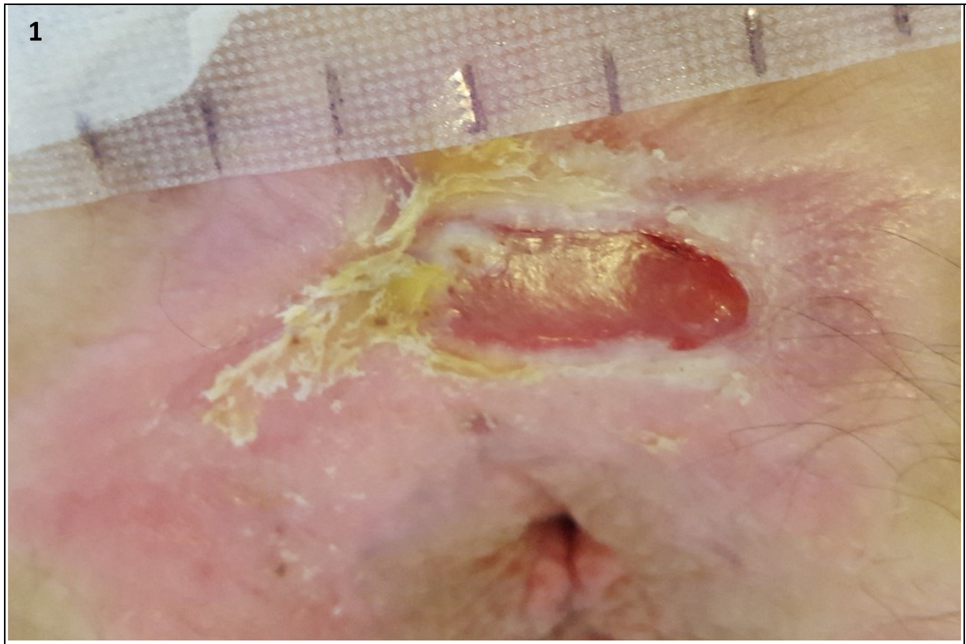	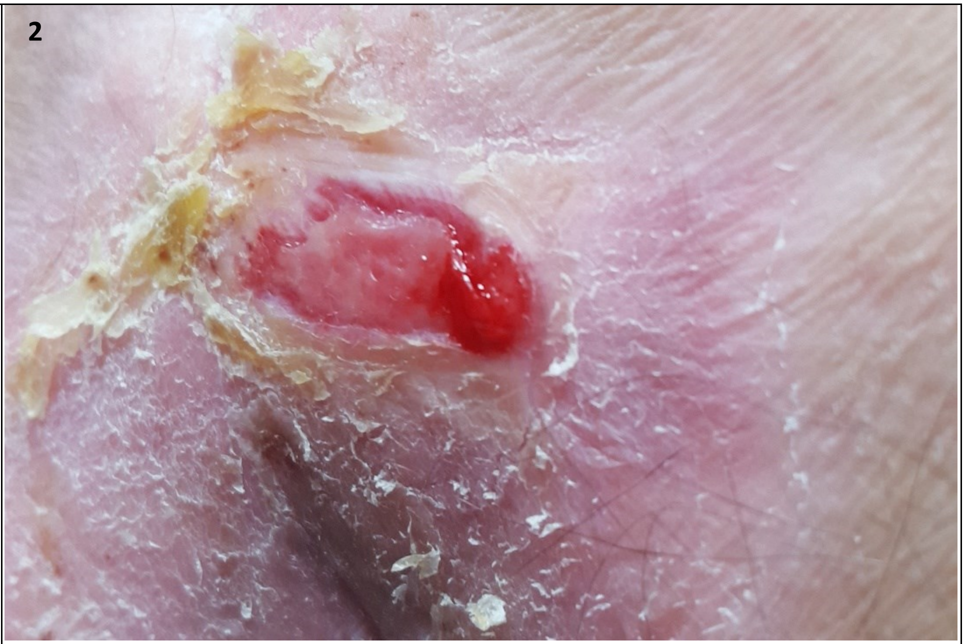
**Day 0**<br>**Just before first MPPT**<br>The upper right corner has a deep elongated aperture into muscle tissue.	**Day 2**<br>A collection of bacterial red-pigment toxins is in the process of being expelled from the tissue through the wound.

3 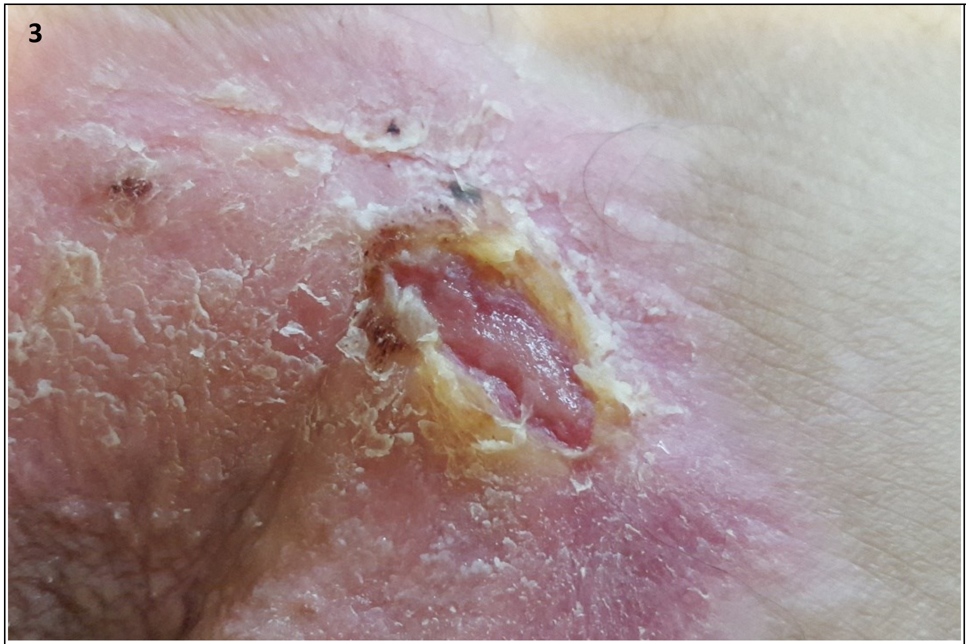 **Day 12**	4 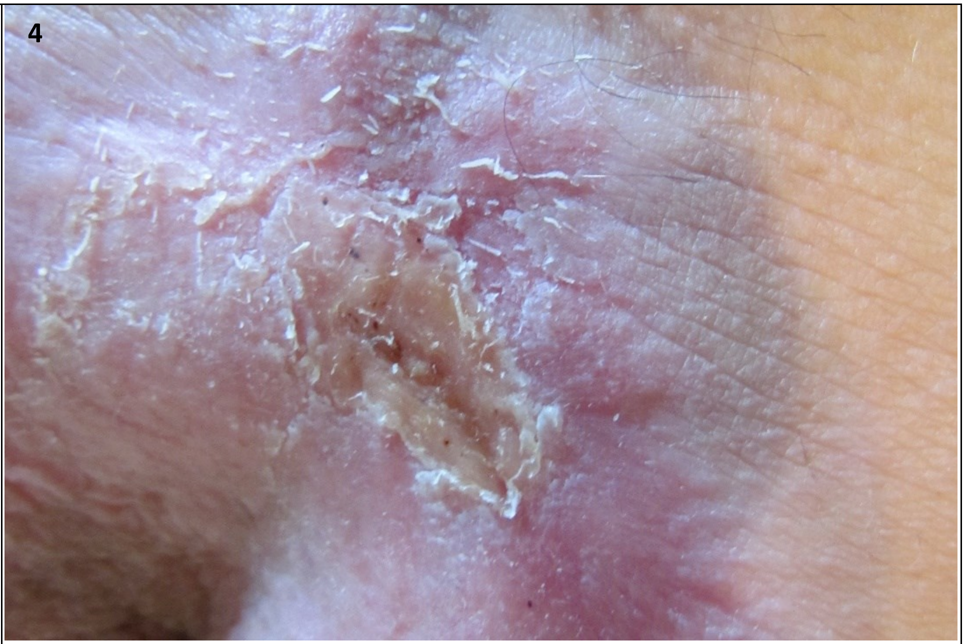 **Day 20** Wound is covered with healthy scab.

5

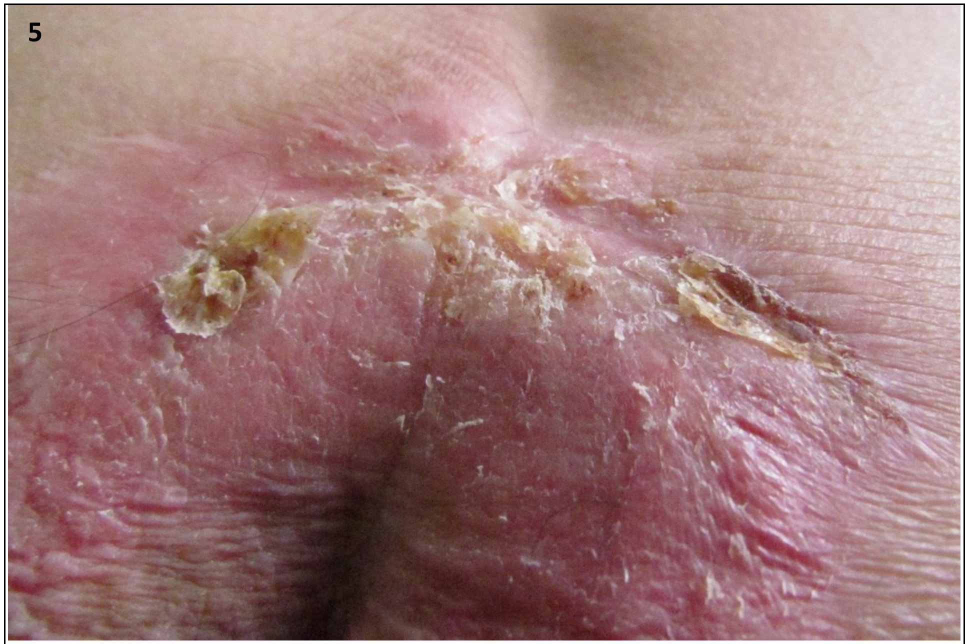

3.5 months Day 105

Exudate from highly infected wound in the vicinity is infecting the scar tissue on all sides of the original ulcer. These areas are already covered with protective scab and healing.

6

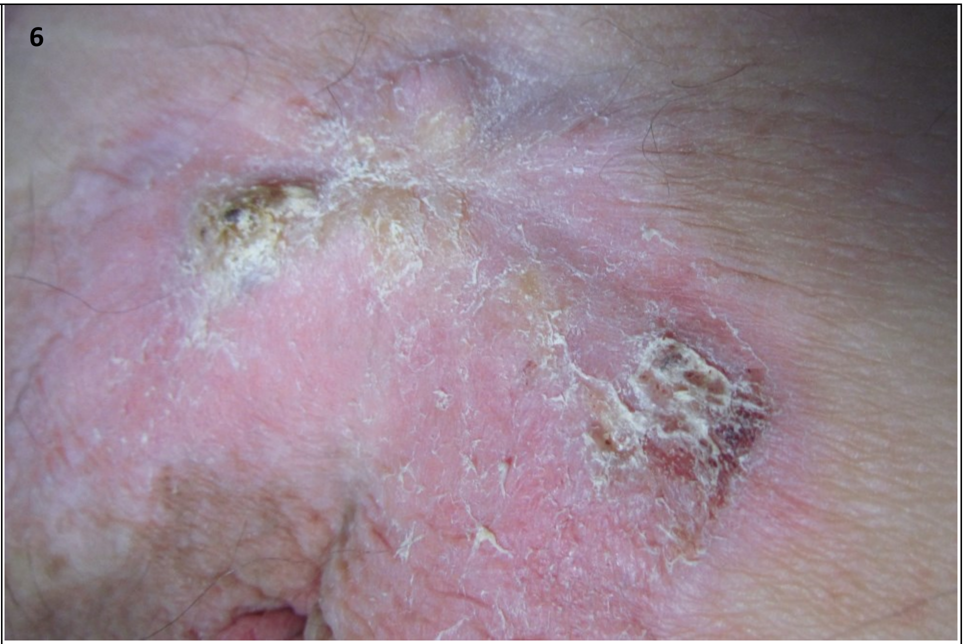

5 months Day 151

Original wound location is fully closed.

7

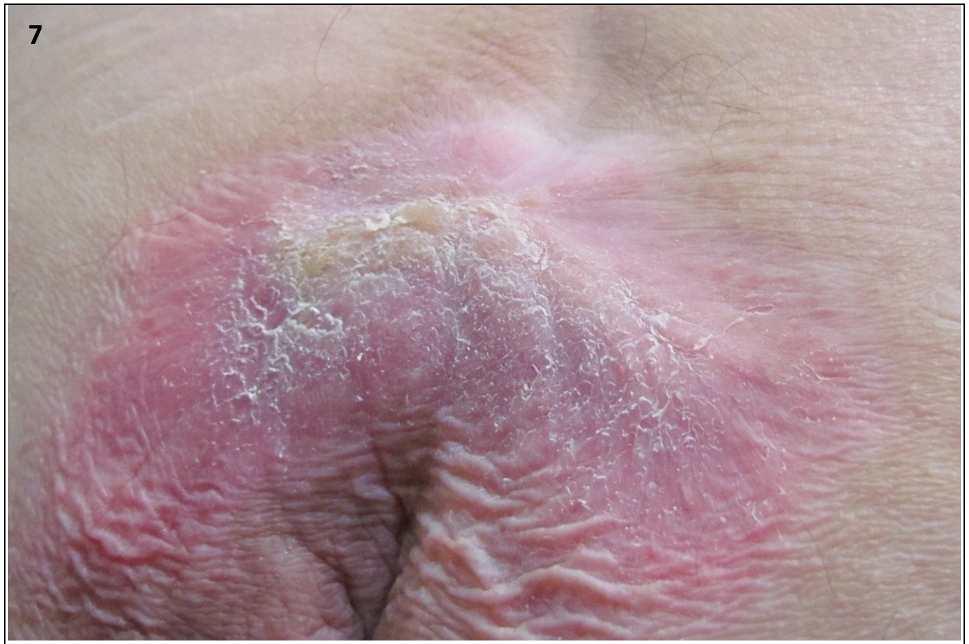

7 months   Day 213

Closed

Wound number 25			Patient	SCI	
**Grade 4**	**6 months old**	**Ischial tuberosity**	**50-year-old male**	**44 years**	**C5/6 complete**

This 6-months old ischial tuberosity wound had, 3 months prior to starting MPPT treatment, been shown to reach bone without affecting bone. Since then, it had been dressed daily first with hydrofiber/hydrogel (Aquacel) but the depth did not reduce and the width of the sinus enlarged. Directly before the MPPT treatment, the wound and tunnel were packed daily with povidone iodine (Braunol) and occluded. This led to further deterioration, and the treatment was changed to MPPT.

MPPT rapidly reduced the exudate level and size of the wound. The tunnel closed from the bottom upwards and once it reached the skin level it epithelialized.

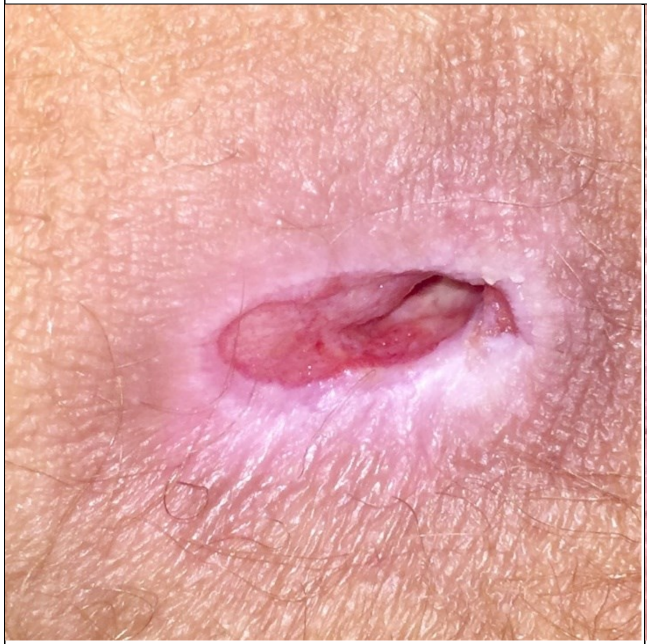

Day minus-12

2 weeks before MPPT

Sinus entrance clearly visible

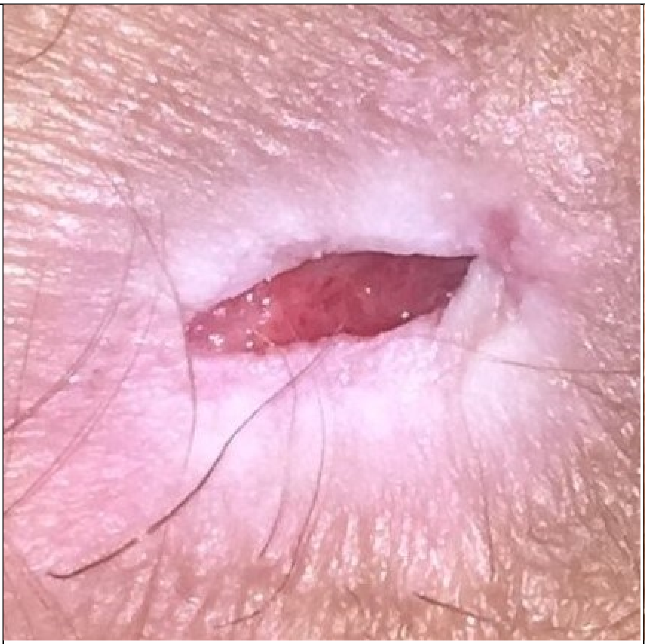

Day 0

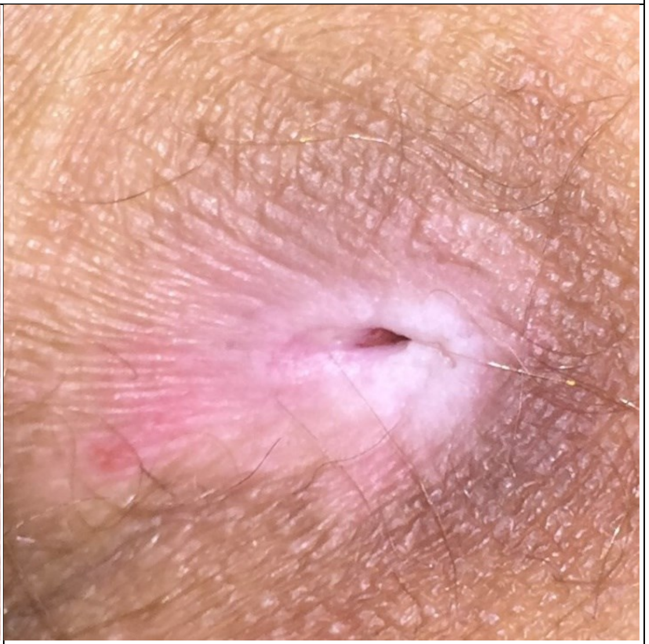

Day 11

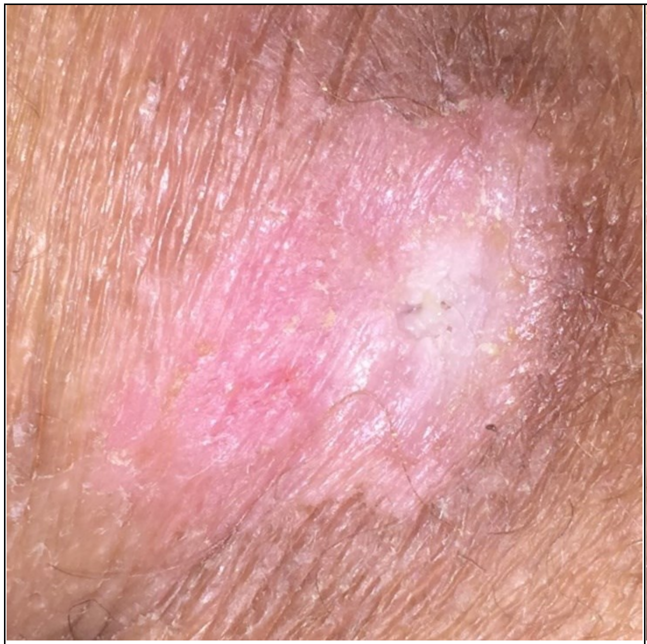	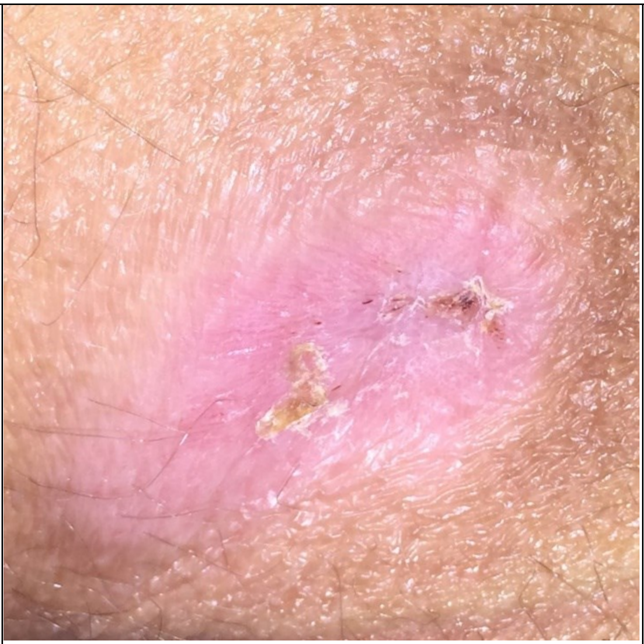	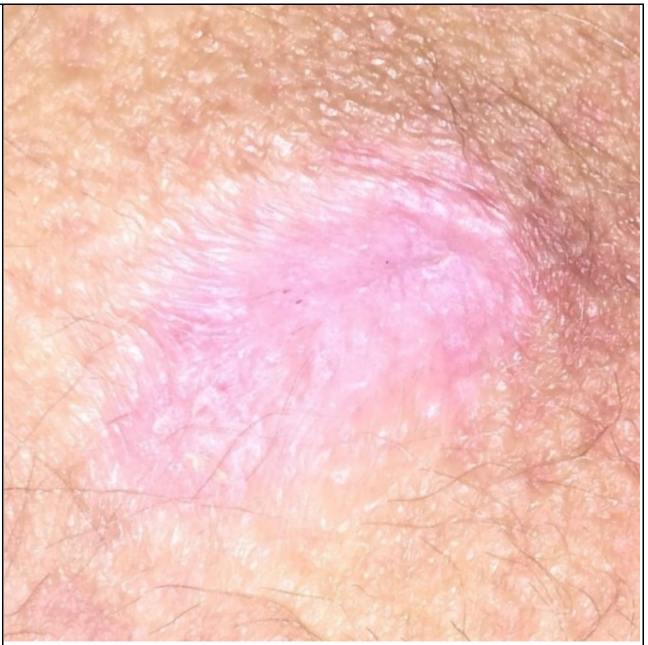
**Day 34 1 month**	**Day 73 2.5 months**	**Day 79**<br>**1 week following closure**

Wound number 26			Patient	SCI	
**Grade 3**	**2 years old**	**Sacrum**	**38-year-old female**	**20 years**	**C5 complete**

The ulcer caused by a bad transfer 2 years earlier and requiring prolonged periods of bedrest, was 1.5 x 1.4 cm, with no tunnelling or undermining. It had been treated with Silver sulfadiazine, silver impregnated dressings and hydrocolloid dressings and had, at some point, shown some progress, but then deteriorated and turned chronic. The patient had been on full bedrest for the past 6 weeks prior to MPPT without bringing about any improvement. Judging from what was apparent to the naked eye, the ulcer seemed covered with a layer of tenacious biofilm (pic 1).

MPPT was applied once daily for three days (Day 0, 1 and 2) and once more later in the healing process due to a bowel accident followed by very thorough showering with only tap water. The patient was keeping a high degree of bedrest. From Day 14 to 16 the patient was in hospital resting on the ulcer to undergo a planned surgical non-wound-related intervention. The ulcer was epithelialized on Day 17 (pic 4) and the patient celebrated her new-found freedom by flying on holidays on Day 20. The site continued to mature and gain tensile strength over the following months.

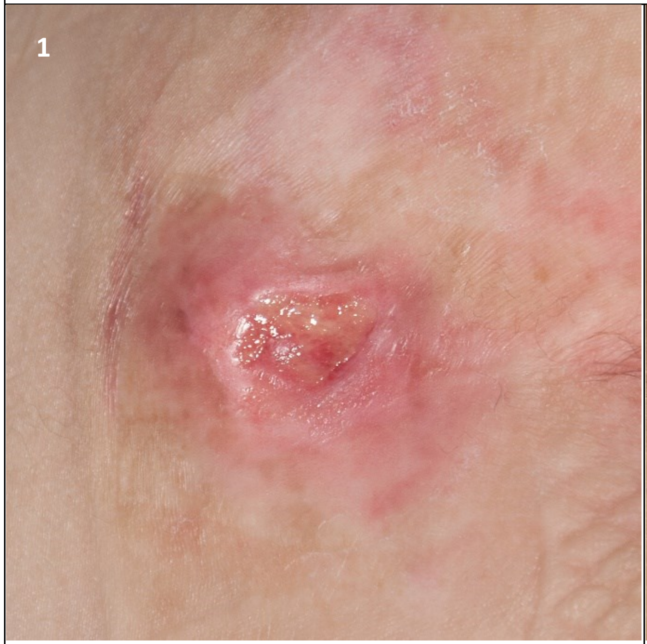

Day 0 –Just before first MPPT

1.5 x 1.4 cm

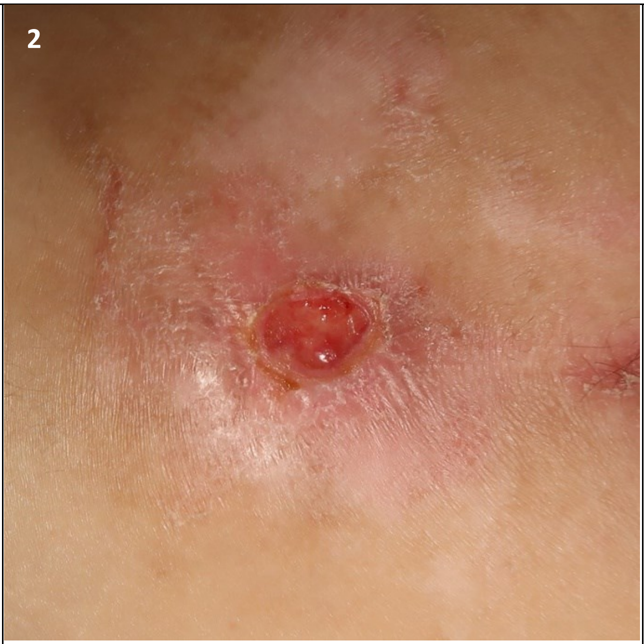

Day 5

Tensile contraction of skin visible

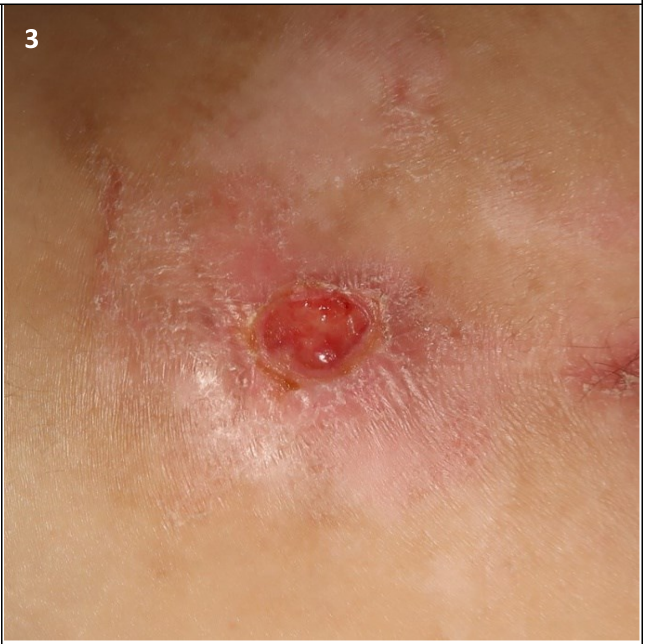

Day 9

Flakes visible on the intact skin

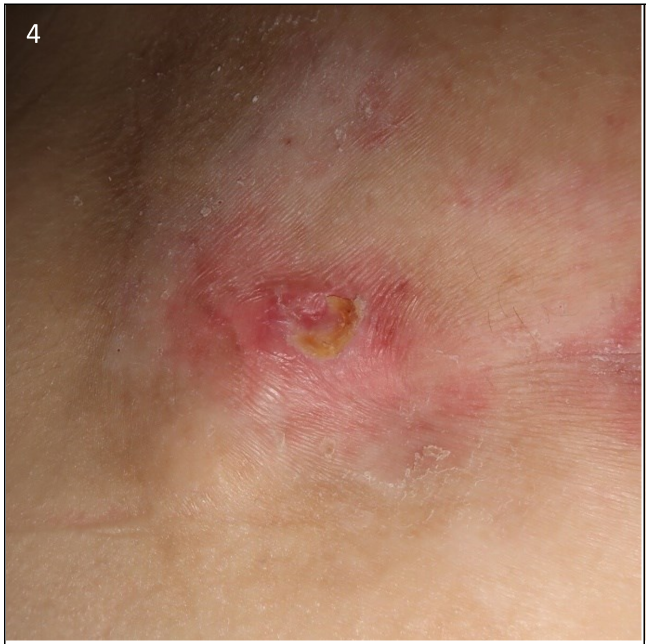

Day 17

Epithelialized

12 hours after returning from hospital after resting on it for 2 days and rubbing its surface during long drive home.

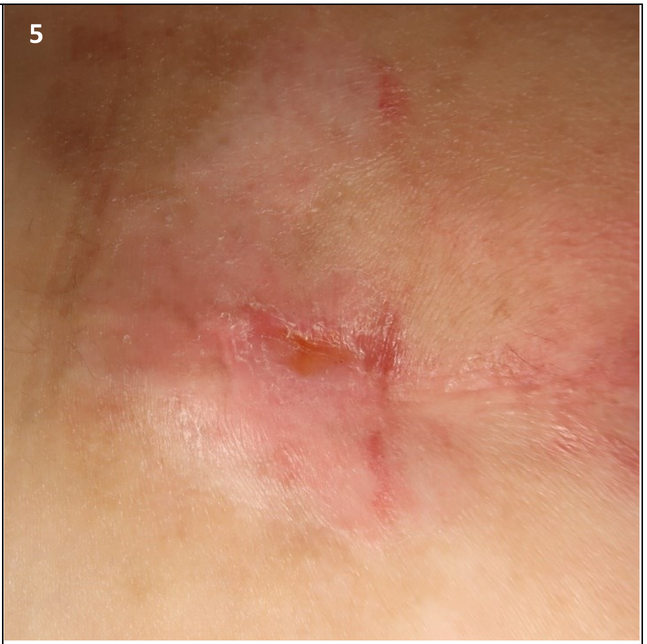

Day 23

Picture taken whilst on holiday following driving and flying

Wound number 27			Patient	SCI	
**Grade 3**	**11 weeks old**	**Ankle**	**50-year-old female**	**10 years**	**C5/6 incomplete**

10 years before the appearance of this wound, the patient had broken her ankle requiring metalwork on both sides right above the malleolus and six months later suffered the accident causing tetraplegia. 6 years later, the metalwork was removed and, whilst it healed satisfactorily, half a year later, the leg suffered generalised cellulitis (foot to knee) and has since been causing trouble swelling. Deep vein thrombosis was ruled out, but Doppler test showed poor pulse.

11 weeks earlier, the appearance of a dark purple spot on the scar rapidly progressed to an open wound (pic 1). It was treated with povidone iodine (Inadine) dressings. As the wound worsened, first different types of honey dressings were tried but the wound continued to deteriorate, and next poly-absorbent polyacrylate fibres with petroleum jelly, an oestrogen-mimic, (Urgoclean) was used for 5 weeks, but the wound continued to deteriorate. The leg and foot were, hot, swollen, very painful and with frequent and strong spasms that also impeded sleep. The condition was diagnosed as “Spastic Phenomenon”. At this time MPPT was commenced (pic 2). At the same time, the fear of an osteomyelitis based on the history of the old metalwork and based on the unexplainable appearance of the wound, prompted a 21-days course of two systemic antibiotics (Phenoxymethylpenicillin and Flucloxacillin) as a cautionary measure and an X-ray which, 20 days later, revealed excess fluid around the ankle but no osteomyelitis, for which reason the antibiotics were discontinued (pic 4). The patient had been suffering from chronic severe headache since shortly after the first appearance of the wound 3 months earlier.

The first 24 hours with MPPT, the wound was hot, stingy and with lots of additional spasm. These symptoms gradually reduced over the following days and so did the headache. The systemic antibiotics complicated the autolytic debridement processes and the slough stiffened and remained intertwined within and on top of the developing granulation tissue in the wound bed (Pic 4). The moment the systemic antibiotics were discontinued, the stiffened slough started to ease and autolytic debridement efficiently cleared the slough allowing granulation and healing to considerably pick up speed (pic 5 & 6). 48 hours after the discontinuation of the systemic antibiotics, for the first time in months, the patient enjoyed a restful night, falling asleep and sleeping through, with no spams to wake her and the leg was no longer hot – it was still warm and would swell when in prolonged vertical position. Gradually, a firm dry protective scab developed covering the entire wound bed (pics 6 & 7 & 8). This was checked daily to confirm that no liquid, e.g. pus, would appear from under it. The scab was left undisturbed for proliferation to proceed under its natural protection. It would break off gradually in tiny pieces along all edges over many days, leaving new epithelium underneath. Eventually, the large main piece it fell off by itself leaving a fully epithelialized, i.e. closed, surface behind (pic 13). The natural maturation and remodelling processes to build up tensile strength continued in the closed wound (pic 14 & 15). The healing suffered a +4 week interruption in the healing process when a tape adhesive caused a severe allergic reaction in the wound and its surroundings (pic 9 & 10 & 11).

<table>
<tr>
<td>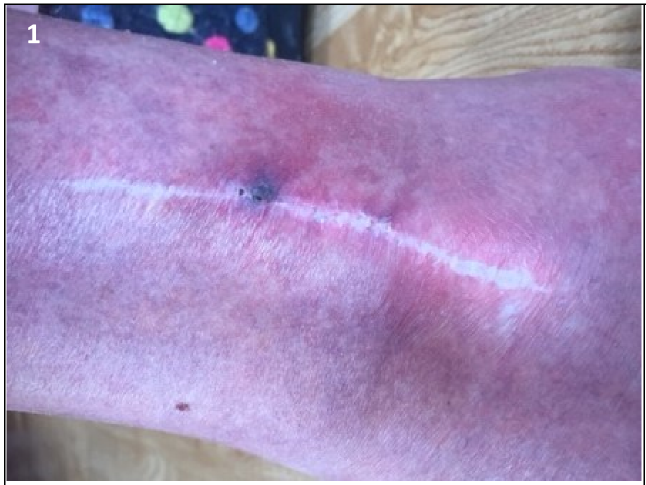</td>
<td>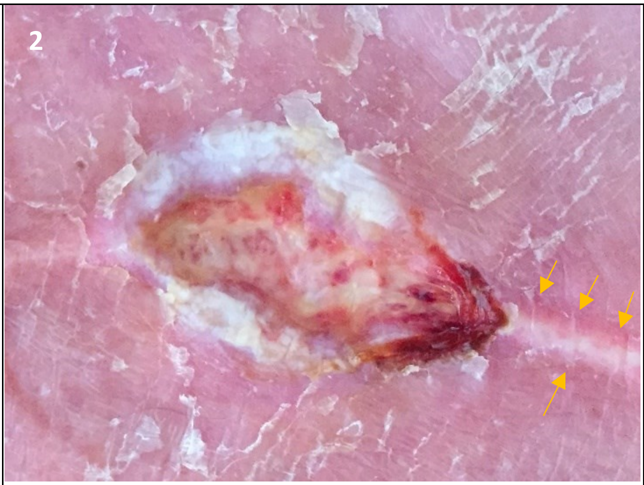</td>
<td>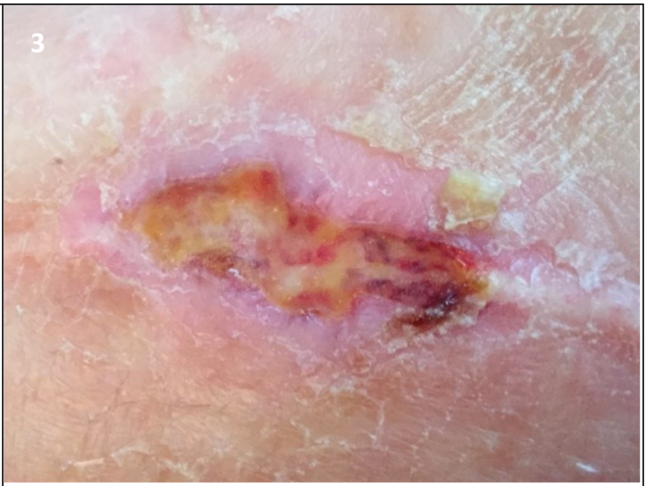</td>
</tr>
<tr>
<td>11 weeks before starting MPPT
A few days after first detection</td>
<td>Day 0
Just before first MPPT</td>
<td>Day 1</td>
</tr>
<tr>
<td>Wound opening from within as a black spot in the old surgical scar. The black spot holds two tiny but distinct holes. It is associated with diffuse inflammation and swelling.</td>
<td>The scar is dehiscing particularly in the distal direction (right side in all pictures). Notice the bright red colour along the white scar (orange arrows).

Skin generally inflamed. Wound edges macerated.</td>
<td>Wound edges have contracted, the maceration has cleared and the disseminated skin inflammation is contained.

The scar dehiscence is halted, the scar is mending as the red line along the scar is disappearing.

Leg and foot had been stingy and hot overnight with lots of additional spasms.</td>
</tr>
</table>

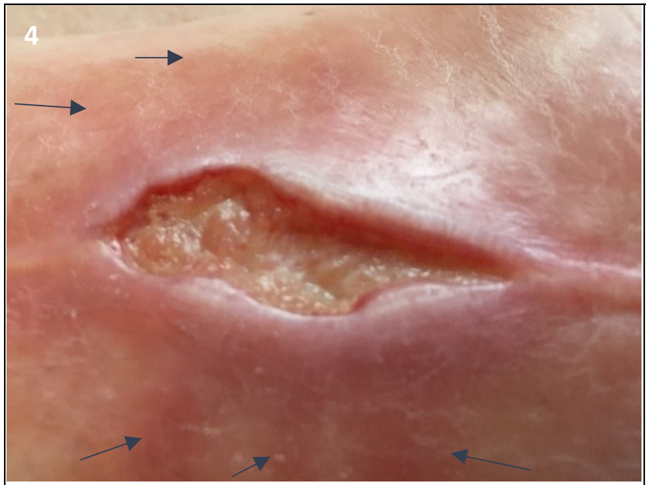

Day 17

Still one day left of working alongside 2 systemic antibiotics

Whereas the wound was improving noticeably, the two systemic antibiotics considerably reduced the speed of autolytic debridement, toughening the wound bed and stiffening the slough within and on top of new granulation tissue.

Notice the red streaks radiating from the wound (*blue arrows*) indicating that the infection is not entirely contained in the skin but still seeking to spread. The skin has an unnaturally stiff appearance.

Epithelialisation along the edges was taking place but at an unusually low speed and with a swollen appearance.

The antibiotics had for the duration of the course been causing nausea and loose bowels.

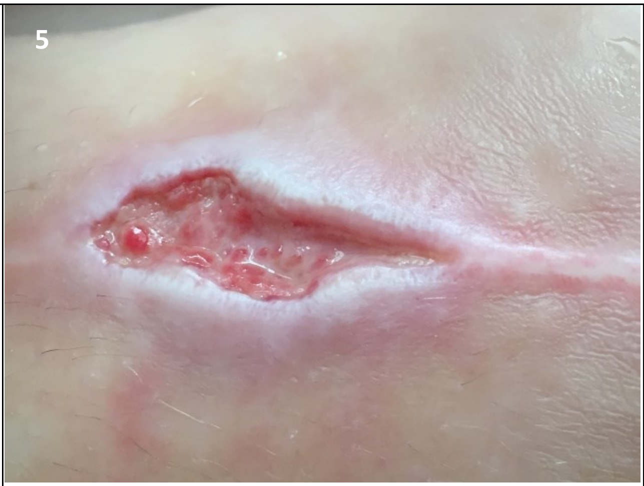

Day 20

The stiff and embedded slough is giving way and the granulation is peeping through in entire wound bed.

The systemic antibiotic were discontinued 24 hours prior to this picture. This rapidly accelerated healing.

The radiating red streaks are narrowing and gradually disappearing over the following days.

For the first time since the wound appeared, the patient fell asleep at bedtime and slept through. The leg was warm but no longer hot, there were no spasm at all, neither during the night nor the following day. Leg still swelling when vertical.

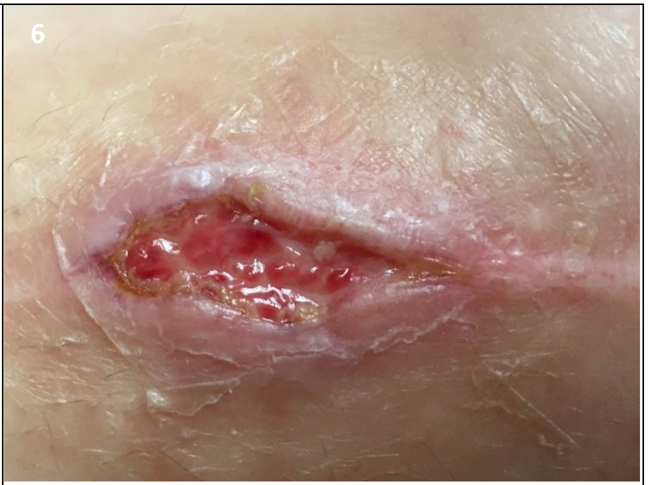

Day 25

Proliferation in all aspects

Wound bed granulating, slough has disappeared, exudate is minimal, the edges are epithelialising and the yet-to-mature skin cells are moving in from all sides protected by brown, still soft scab.

Red streaks in skin are disappearing indicating that the infection is contained and no longer spreading.

The skin has a natural and bendy appearance.

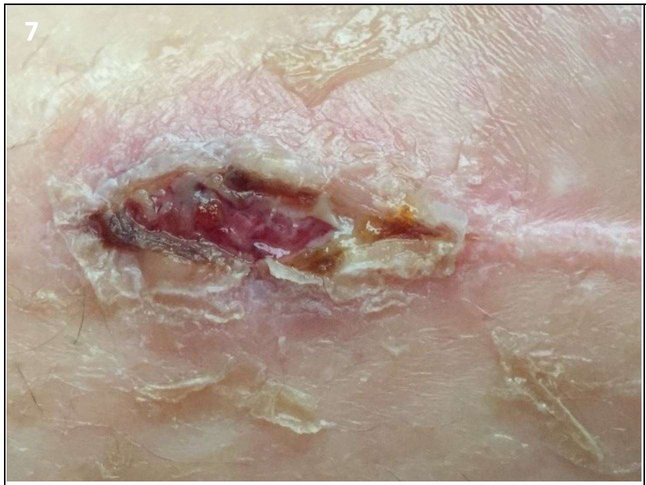

Day 36 1 month

Same epithelialisation processes continue. The edges are gradually being covered and protected by broader and thicker scab formation.

Red streaks in skin (see pic 4) have disappeared

The red line along both side of the scar (see pic 2 arrows) has cleared.

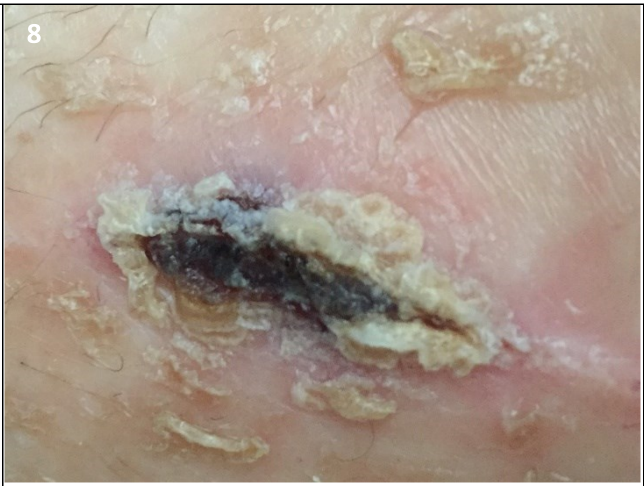

Day 50 1.5 month

Fully scabbed

A firm, hard scab is covering the entire wound. Along all its sides, the scab consists of layers of flakes.

This scab is nature's "secondary dressing" and offers effective protection and the optimal conditions for the regeneration processes to proceed underneath. It remains in place and falls off when epithelialisation in complete and the new skin cells require further air to fully mature.

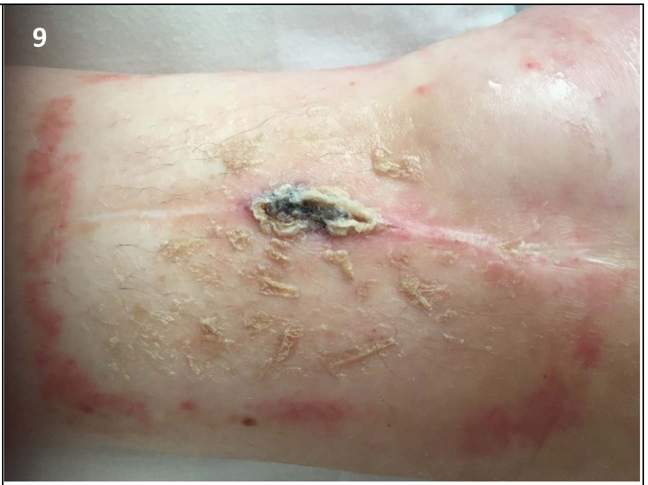

Day 66 2 months

Allergy from tape adhesive

The adhesive of a new sticking plaster tape caused an allergic reaction in the surrounding skin. The allergy in the pictures is already well advanced. Allergy immediately brings all healing processes to a halt and brings the area back to the pre-healing inflammatory processes.

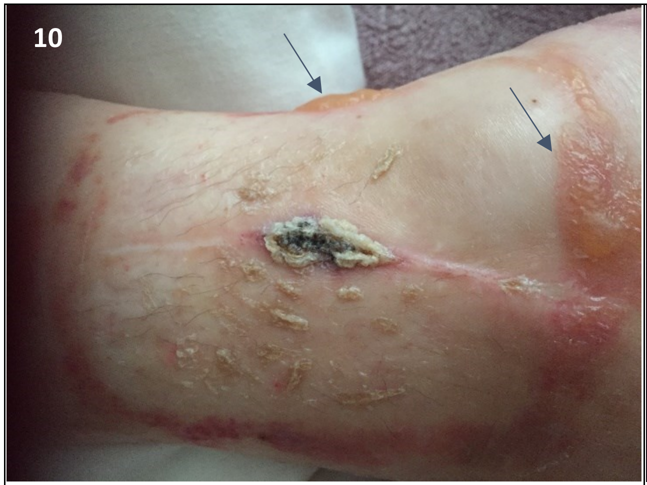

Day 72 2.5 months

Allergy from tape adhesive

The severe tape allergy was not reacted upon in time and blisters developed in several places ((arrows show examples). The tape was now changed and, of course, positioned differently.

The scab remained in place while it, together with the newly created blister wounds, were treated with MPPT, and recovered.

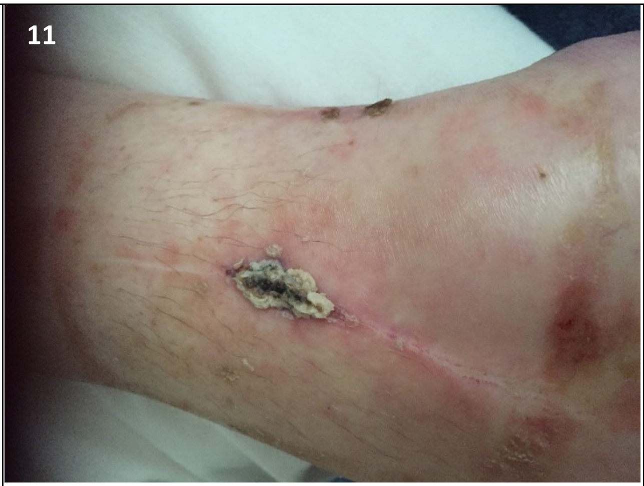

Day 91 3 months

Allergy relatively mitigated

Allergy and its sequelae have greatly improved. The scab had stayed on the wound and healing along the sides seems to be restarting.

Every day the scab was pressed down gently to check that no liquid would appear along its side(s).

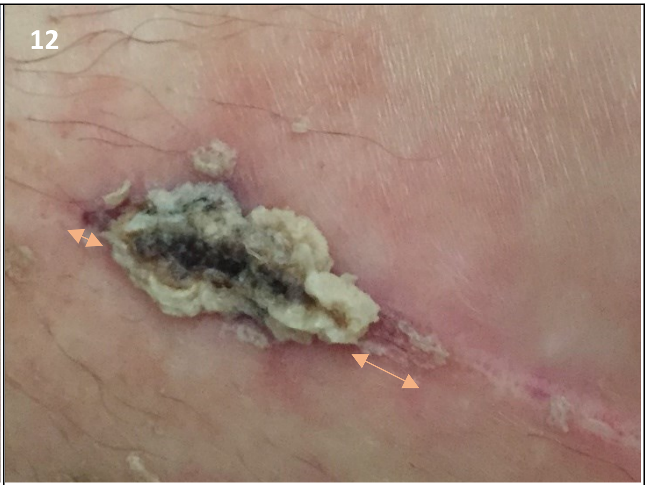

Day 91 3 months - close-up

Allergy relatively mitigated - healing restarting

Allergy relatively mitigated but the immediate surroundings of the wound are still inflamed as is the old scar line, which should be white.

The scab is reducing in size as it lifts and falls of in tiny pieces along all edges leaving new epithelium to mature (compare to pic 8 just before allergy). Evident examples are seen above the double ended arrows.

<table>
<tr>
<td>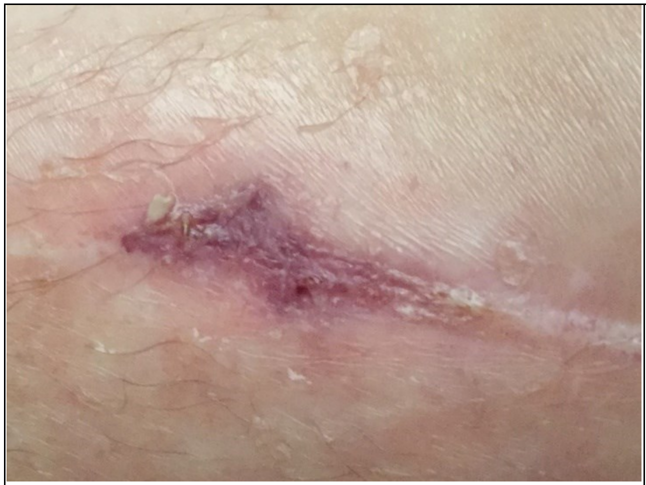</td>
<td>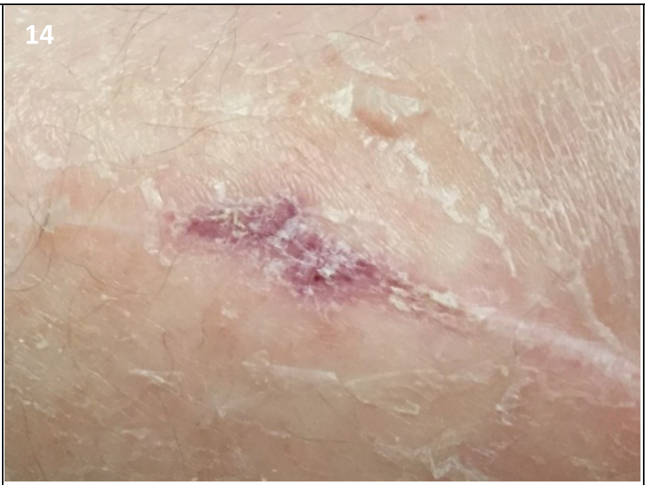
</td>
<td>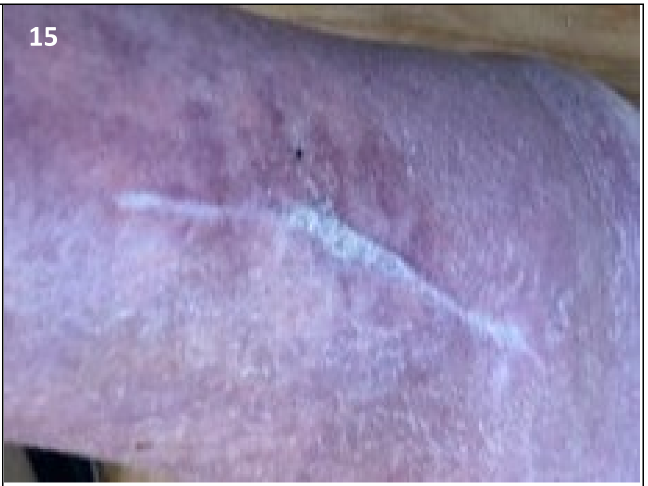
</td>
</tr>
<tr>
<td>Day 101 3.5 months
Closed
The scab fell off by itself leaving a fully epithelialized wound. The area is still maturing - this requires air.
The leg is still prone to swelling when in vertical position.
Pain, spasms, and headache have not returned.</td>
<td>Day 112 3.5 months
All tissues are maturing and gaining tensile strength.</td>
<td>Day 277
6 months after closure
The wound was located in an old surgical scar which is now looking similarly to how it looked before the wound broke it (see pic 1).
The leg remained prone to swelling.</td>
</tr>
</table>

Wound number 28			Patient	SCI	
**Grade 3**	**12 years old**	**Ischial tuberosity**	**45-year-old male**	**27 years**	**L1 incomplete**

For the past 12 years, this grade 3 pressure ulcer had been constantly reopening and closing only to reopen again. It had not passed beyond the deepest anatomical barrier of tela subcutanea (fascia superficialis) into muscle. It had been cleaned daily with a shower followed by wound wash containing 0.5% of the antiseptic Cetrimide (alkyltrimethylammonium bromide) and covered with a plain dressing. When MPPT treatment was started (pic 1) it had been open and non-healing for the past 5 months. It was 1 cm in diameter and 3 mm deep with light exudate.

Within 4 days, the wound had reduced 50% in size and the entire wound bed was granulating healthily (pic 2). Within the following 5 days, the wound was covered with a natural scab, acting as nature's own protective dressing (pic 4). The scab would keep changing in appearance signifying active healing processes taking place underneath. Once, the scab was physically removed (pic 5). This exposed a healthy and healing soft tissue structure. Unfortunately, it had been disturbed too early in its maturation processes, but the wound rapidly rebuilt some of the scab (pic 6). Furthermore, the area beyond the wound bed was also still using the wound opening and its scab to discharge any debris that would be created from the processes of clearing all spots containing infection, potentially from 12 years back, from the whole area (pic 7).

The patient would continue an active lifestyle working full time, and the wound would continue its regenerating processes under the scab which would continuously change in appearance. At times, the scab would show signs of infective waste material, e.g. inhibited red-pigmented toxins, emanating from the infection removal processes that were taking place in the infiltrated soft tissue beyond the opening (see pic 7). This was caught and embedded in the scab as dark elongated spots, where after it was gradually disposed of with the shredding of the upper layers of the scab whilst new scab was forming at the level of the wound bed as part of the ongoing regeneration processes. 4 months after start, the wound was closed and remained closed with no further issues (pic 8). Ongoing regeneration and maturation processes continued to level the scar and reduce its size (pic 9).

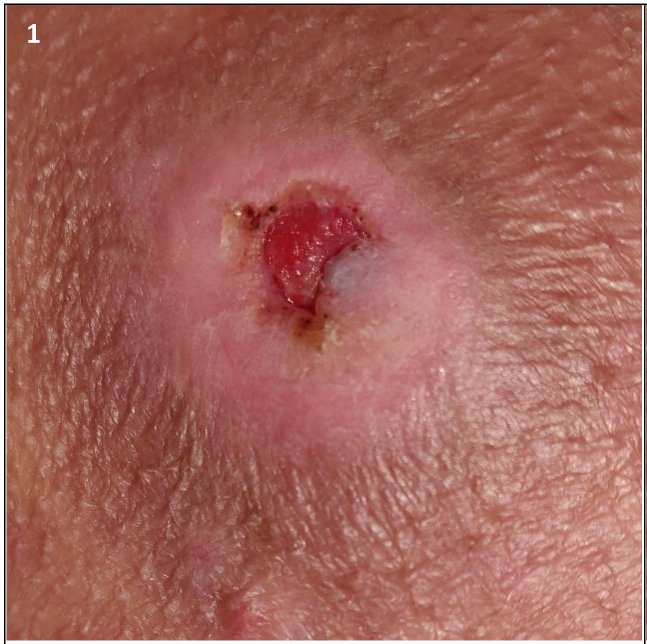	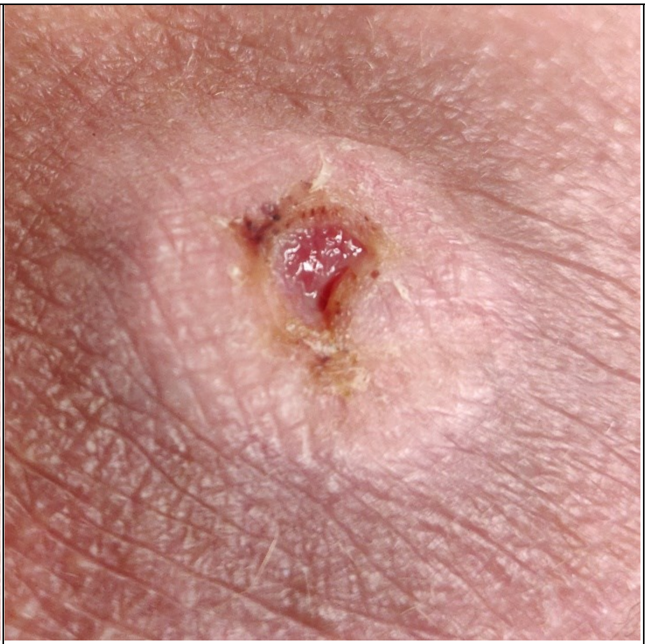	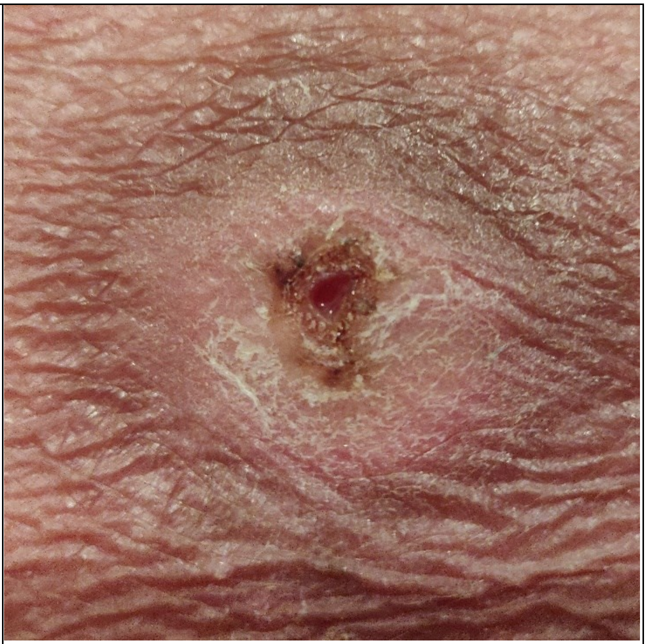
**Day 0**<br>**Just before first MPPT**<br>10 mm diameter. 3 mm depth.	**Day 4**<br>50% reduction in wound area.<br>Granulating wound bed and epithelializing edges.	**Day 6**<br>Wound bed is very small, with scab moving to cover and protect from all sides.

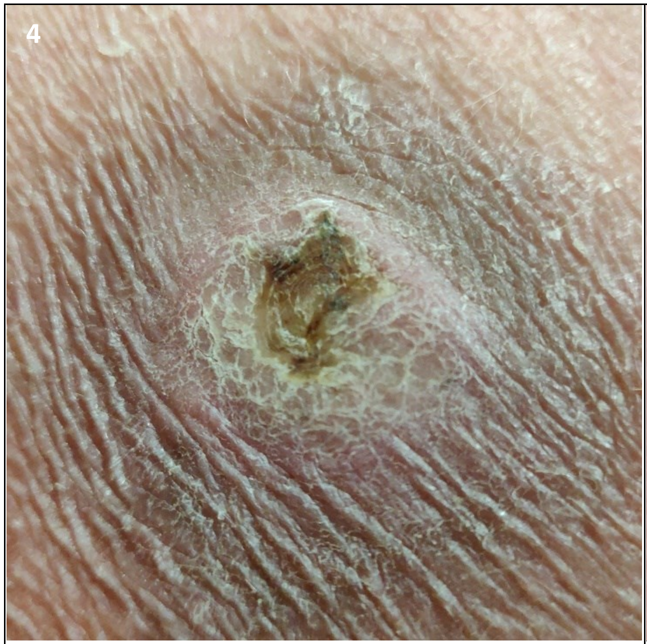	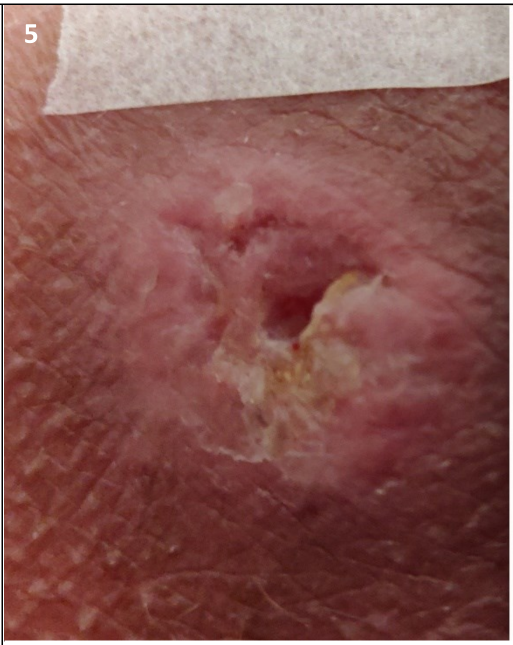	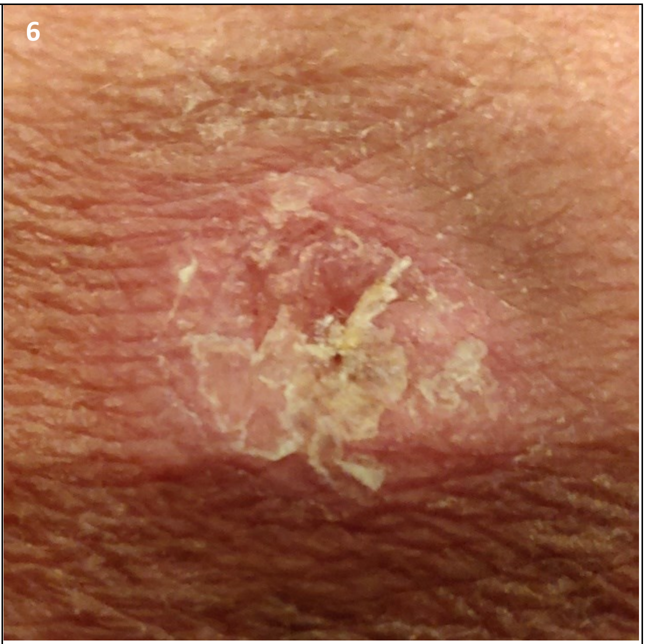
**Day 9**	**Day 18 morning**	**Day 18 evening**
The granulating wound bed is now fully protected by a natural scab.	Scab has been physically removed.<br>Underneath is healthy tissue. This new soft tissue was still too immature to be without the protection of the scab. Also, the surrounding area is still using the opening and the scab for disposing of debris.	12 hours later, the wound had already reconstructed some of the lost protective scab.

<table>
<tr>
<td>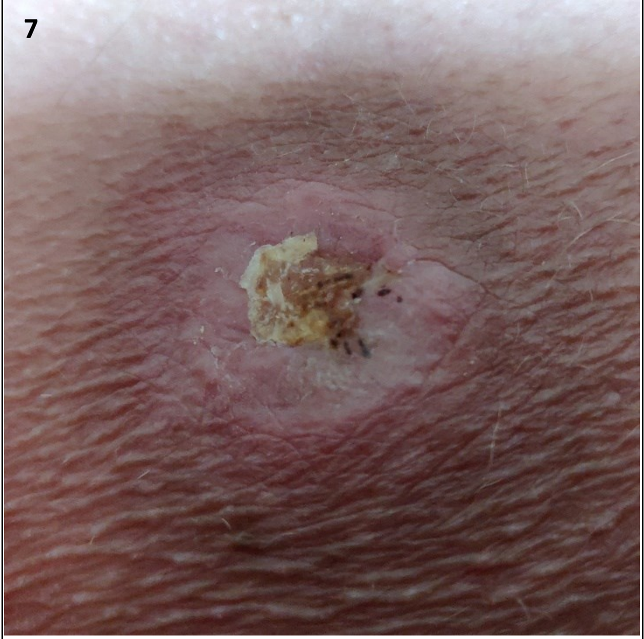
Day 32

Red-pigmented bacterial toxins are being caught, inhibited and expelled through the wound bed. Here, they are seen embedded in the scab as dark elongated shapes. They are shredded as the old scab breaks off whilst new scab substitutes from the bottom.</td>
<td>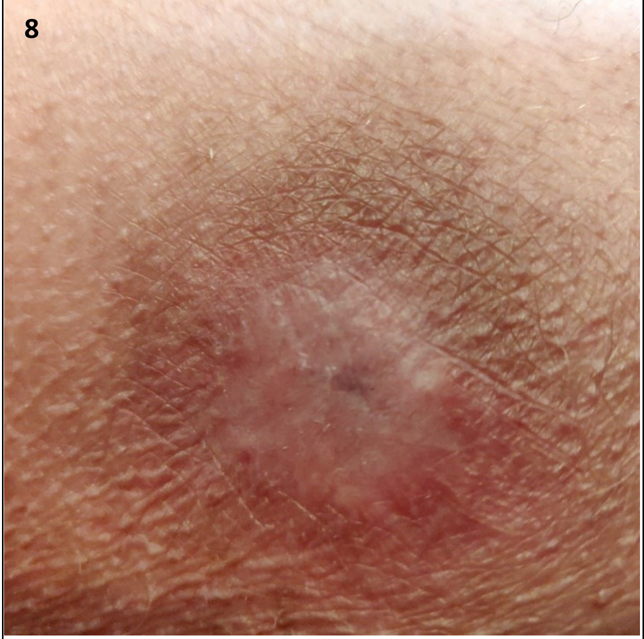
4 months Day 125
Closed</td>
<td>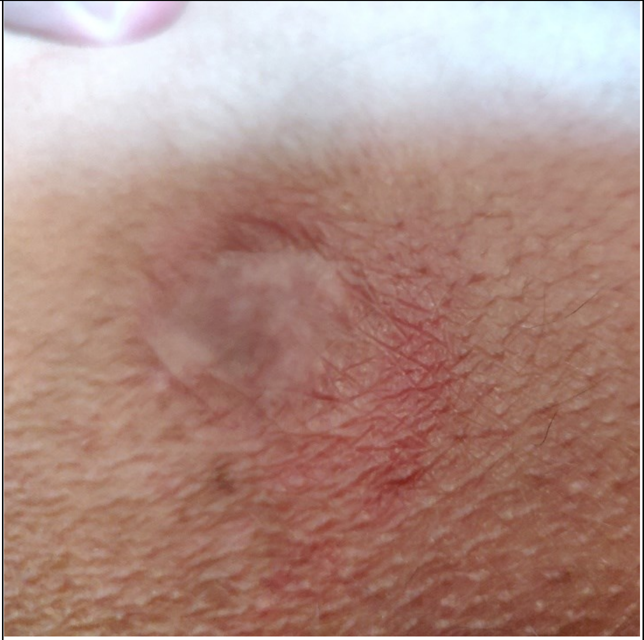
13 months Day 397
Long-term follow-up
Remains firmly closed whilst regeneration and maturation of the soft tissue levels the scar with the body’s surface and reduces its size.</td>
</tr>
</table>

Wound number 29			Patient	SCI	
**Grade 3**	**3 years old**	**Sacrum**	**40-year-old male**	**8 years**	**C5 complete**

This sacral pressure ulcer developed in connection with initial hospitalisation at the time of the accident causing the spinal injury 8 years earlier. It immediately developed into a grade 4. Closing it presented great difficulties and it had been causing trouble ever since. It had been open for the past 3 years undergoing changes in size but at no point passing from a grade 3 into muscle. During the 3 months prior to MPPT treatment, the wound had, every 3 days, been cleaned and left to soak for 10 minutes with 0.1% PHMB and betaine surfactant (Prontosan) after which it had been dressed with a hydrogel contact layer dressing (Kerralite Cool) cut to fit the shape of the wound; the surrounding skin had been treated with a film of polymers in hexamethyldisiloxane (Secura No-Sting Barrier Film); this had all been covered with a highly absorbent polyurethane foam dressing (Tegaderm Foam).

Not long before the initiation of MPPT treatment, the wound had started to increase in size and depth. For two weeks leading up to MPPT treatment, the PHMB cleansing agent had been substituted by water to reduce the load of antimicrobials accumulated in the tissue. The wound opening was rather small (18mm x 4mm) but with a broad glassy, polished, stiff and seemingly passivated border with rolled down wound edges and a stalled irresponsive wound bed (pic 1). Gradually the wound changed into a bendable non-shiny texture actively and constantly changing with granulating wound edges (pic 2) and expulsions of small amounts of bacterial toxins and other debris. The lifestyle of the patient limited the access of air to the wound and the ongoing healing was consequently periodically put on hold particularly in the beginning of treatment. This was eventually remedied by a solution that did not change the lifestyle exceedingly, and after 8 eight months the wound was fully closed (pic 3).

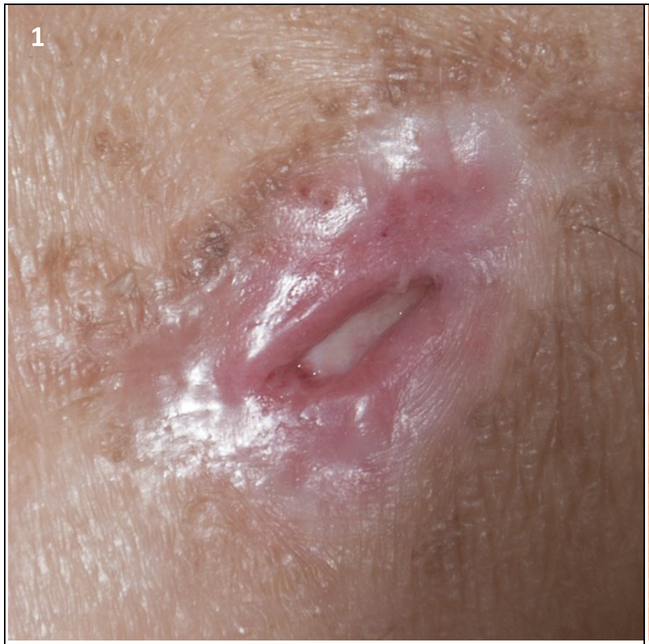	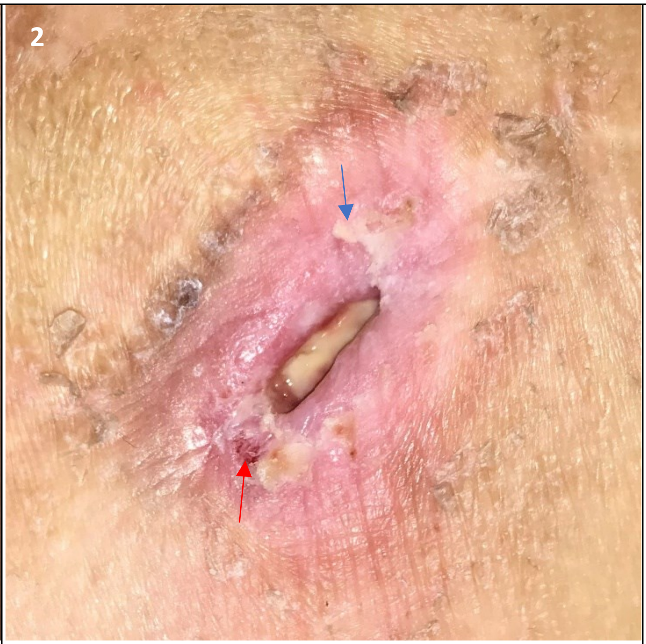	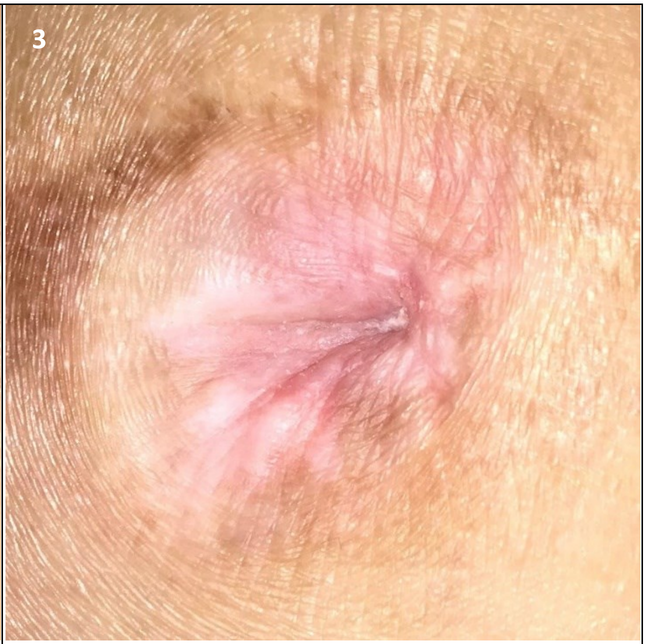
**Day 0**<br>**Just before first MPPT**<br>18mm x 4 mm x 4 mm<br>Glassy, shiny, stiff, stalled, lifeless appearance of broad area of skin immediately surrounding the wound opening.<br>Rolled down wound edges. Non-responsive wound-bed.	**Day 4**<br>19mm x 5 mm x 4 mm<br>Active, changing wound edges.<br>Skin bendable, with uneven appearance of disseminated flakes and irregularities.<br>*Blue arrow:* New epithelium being generated.<br>*Red arrow:* Red bacterial toxins expelled, caught and controlled in scab that will fall off when ready.	**Day 243 8 months**<br>**Closed**<br>Last bit of scab that had been protecting the final regeneration and epithelialisation processes has fallen off. Maturation will continue.

Wound number 30			Patient	SCI	
**Grade 3**	**3 months old**	**Ischial tuberosity**	**61-year-old female**	**44 years**	**T8/9 complete**

The patient acquired her first ischial tuberosity ulcer in this location during her hospitalisation in connection with the original spinal cord injury 44 years earlier. This was successfully operated on 40 years ago and only the occasional small ulcers of short duration had appeared in the area until a change of wheelchair caused one 6 years ago. This ulcer refused all attempts to heal and instead developed an abscess that ruptured causing septicaemia and hospitalisation with thorough surgical debridement. Still the wound refused to heal, and 6 months later the diagnosis of osteomyelitis prompted bone debridement and flap operation. Healing was very slow and the new skin was unusually thin and delicate in a way that, after the wound had epithelialized 5 months later, the patient was instructed to remain on bedrest for most of the day, with a maximum sitting time of 1.5 hours. The practically 100% bedrest of the prior 1.5 years had already lost the patient her job and caused severe mental health problems including anxiety and depression. The flap surgery had removed a great deal of tissue that would normally serve as cushioning between bone and skin when sitting. The area was therefore prone to rapid weakening.

5 years later, a punctured cushion causes a stubborn ulcer in the same area. This is treated with bedrest and Manuka Honey (Activon) but refuses to heal. The mental health problems, including insomnia, return. After three months, the ulcer starts treatment with MPPT. After 2 weeks, the patient starts sleeping approximately 6 consecutive hours every night and regains hope and optimism. 35 days after treatment start the wound is fully closed and the patient self-drives to attend a several-day further education course on location. The wound responds well. The area continues to gain strength. The patient regains confidence and reverts to being an active extrovert person.

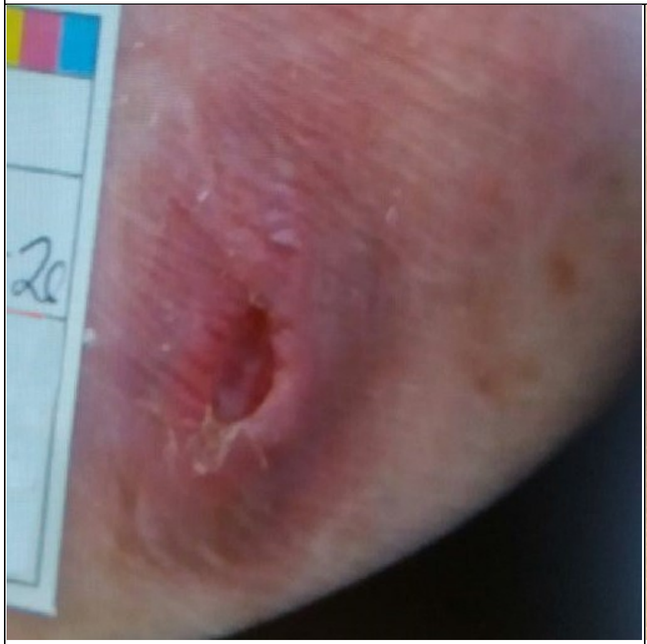

Day -21

3 weeks before first MPPT

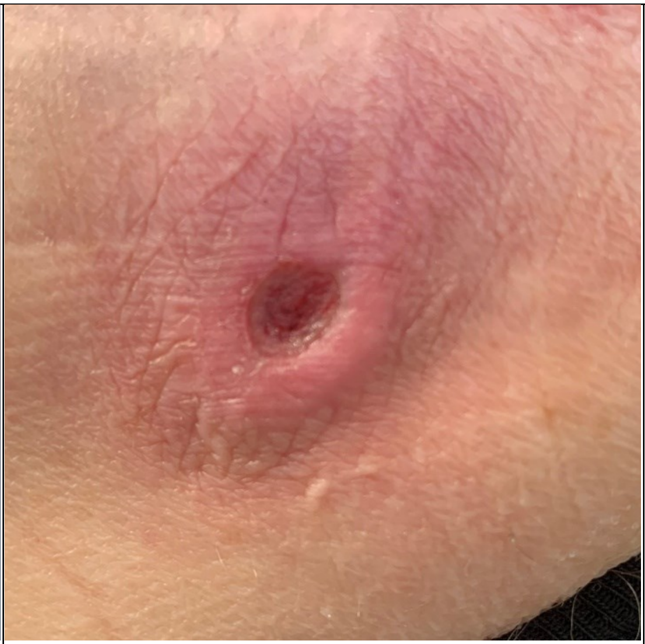

Day 4

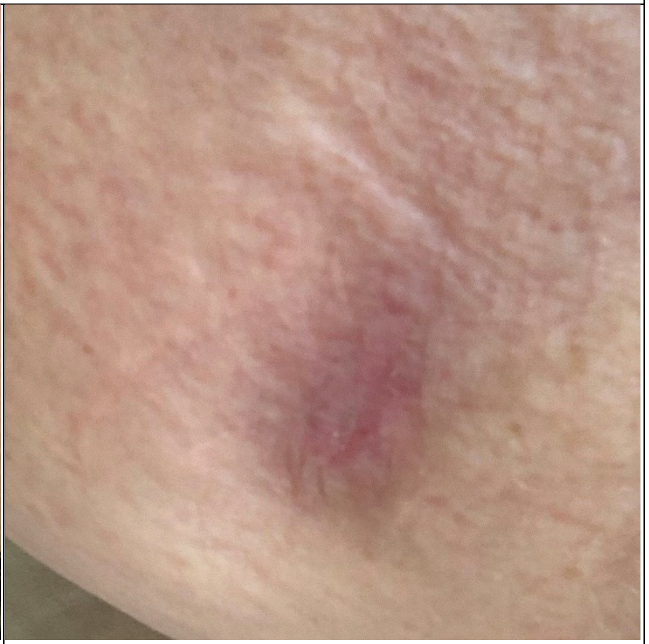

Day 44

1 week after closure

<table>
<tr><td colspan="3">Wound number 31
Grade 3 **15 months old** **Sacrum – Gluteal fold**</td><td>Patient
66-year-old male</td><td colspan="2">SCI
50 years **C5 complete**</td></tr>
<tr><td colspan="6">Originally, the pressure ulcer was 7 cm deep and had halved in size by the use of NPWT (VAC) for 6 months where after it was switched to a series of conventional dressings. Over the following 6 months, the healing slowly progressed but closure could not be reached. The following 3 months the healing stagnated and the continuous discharge caused maceration and required regular debridement.

MPPT was used on the area irregularly, i.e. not daily. The area ceased discharging within a few days after the first application. The two tiny openings enlarged in both width and depth while clearing out the non-viable tissue. Within a month from the start, the area was fully closed. Follow-ups over the subsequent year confirmed that the area remained closed and that the scar tissue matured and reduced.</td></tr>
<tr><td colspan="2">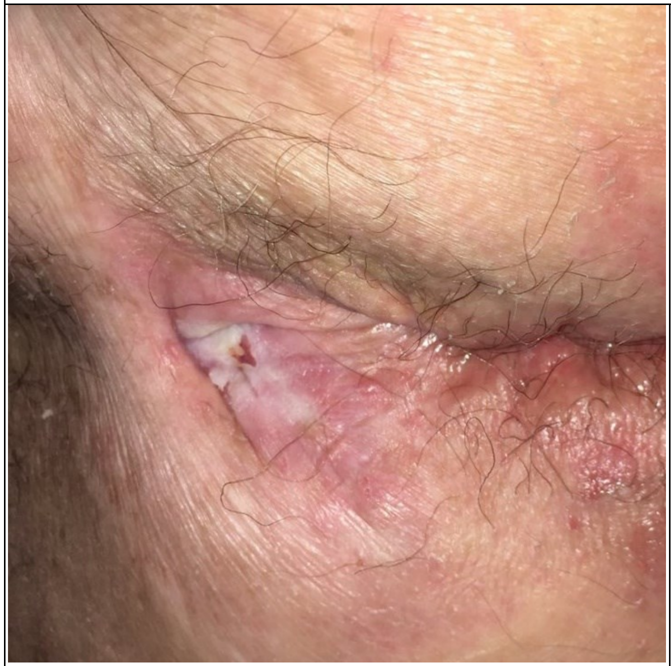
Just before MPPT
Exuding</td><td colspan="2">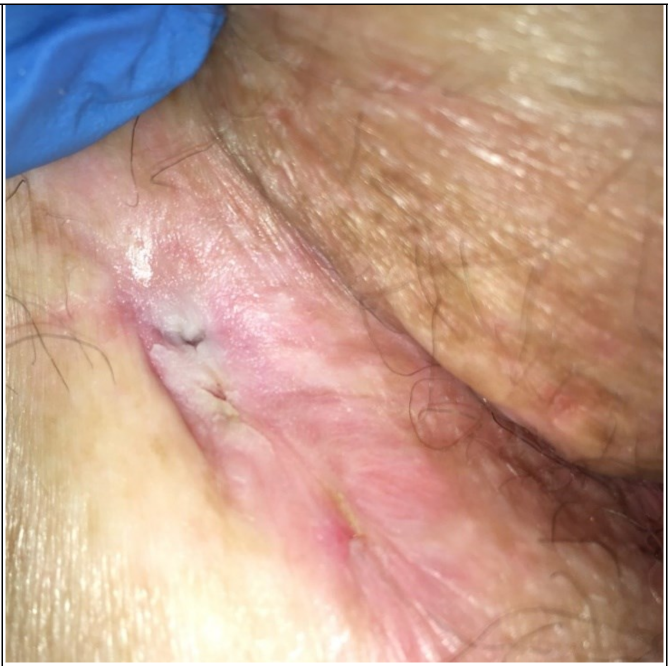
1 week
Non-viable tissue and infection being removed</td><td colspan="2">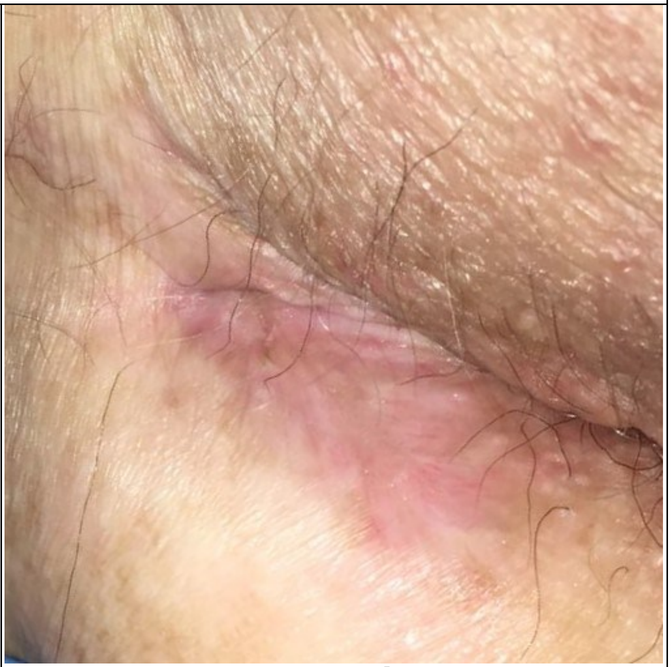
1 month
Closed</td></tr>
</table>
