# Supplementary material for: Stable closure of acute and chronic wounds and pressure ulcers and control of draining fistulas from osteomyelitis in persons with spinal cord injuries: non-interventional study of MPPT passive immunotherapy delivered via telemedicine in community care
Source: Front Med (Lausanne). 2024 Jan 5;10:1279100. doi: 10.3389/fmed.2023.1279100 (PMC10797031; doi:10.3389/fmed.2023.1279100)
Supplement: Supplementary file 9 [file Data_Sheet_9.pdf]

S9: Analysis of wounds acting as fistulas draining from an underlying primary focus of infection

Number	Grade	Wound Age	Location	Cause	Patient
32	4 - draining fistula	3 years & 6 months	Sacrum	Pressure	T4 complete
33	4 - draining fistula	5 years & 2 months	Ischium & Pubis	Pressure	C5 complete
34	4 - draining fistula	8 months	Ischial tuberosity	Abscess	T6 incomplete
35	4 - draining fistula	3 months	Sacrum, ilium, ischium & femur	Radiation	L2 incomplete
36	4 - draining fistula	Many years	Ischial tuberosity	Pressure	T5 complete
37	4 - draining fistula	3 ¾ months	Ankle	Unknown	C3, C6, C8 incomplete & T1 complete
38	4 - draining fistula	5 years	Gluteal fold	Unknown	C5/6 complete
39	4 - draining fistula	9½ weeks	Ischial tuberosity	Pressure	Paraplegic
40	4 - draining fistula	15 months	Ischial tuberosity - Lower back	Pressure	C7 / T1
41	4 - draining fistula	2 years	Coccyx	Trauma (Laceration)	T5 complete & C6 Syrinx
42	4 - draining fistula	3 years	Sacrum	Pressure	C2/3 incomplete & T10/11 complete
43	4 - draining fistula	6 months	Ischial tuberosity	Pressure	C6
44	4 - draining fistula	1 year & 6 months	Sacrum	Pressure	Tetraplegic

In all cases, “**Day 0**” equals the first day of MPPT application.

Wound number 32		Patient		SCI	
**Grade 4**	**42 months**	**Sacrum**	**65-year-old male**	**3.5 years**	**T4 complete**

Part one – picture 1 – 12.

Whilst in hospital Intensive care unit when the spinal injury happened, the patient developed a stage 4 pressure ulcer. Despite many attempts to heal it, the ulcer caused wider tissue infiltration and breakdown and gradually sinus tracts developed in 4 different directions with corresponding skin inflammation (pic 1 - 4). The first 1 ¼ year following injury were spent in hospital, of which 9 months exclusively due to the ulcer. During this 15-month period, a wide array of approaches were tried, including NPWT with and without saline instillation, as well as Inadine, Kaltostat, Durafiber, Durafiber Ag, Allevyn Life, Allevyn Adhesive, Allevyn Gentle, Tegaderm foam, Sorbaderm, Debrisoft, Aquacel Foam, Promogran, Iodoflex. Concomitantly, the diet was monitored and optimised. Eventually, the patient was discharged into community care with an open wound. In community care, over the following 2 ¼ years, the following products were tried: Prontosan, Aquacel Ag, VAC pump, Inadine, Kaltostat, Durafiber, Durafiber Ag, Allevyn Gentle Border, Tegaderm Foam, Sorbaderm, Debrisoft, Aquacel Foam, Promogran, Iodoflex, PICO, Suprasorb, Medihoney gel, packing ribbon, Manuka honey dressings, Flamazine, Cutimed Sorbact, Flaminal Forte, Biatain Silicone, Kliniderm, Allevyn. However, closure could not be achieved. Surgical double flap operation was considered. The treatment goal of MPPT was to stop the diffuse spread of the infection and reduce the existing infection as much as possible to allow a more restricted surgical intervention and increase its chances of success. After two months of MPPT, the wound was deemed ready for surgery (pic 5 - 12).

When starting MPPT, the 3.5-year-old wound was malodorous and exuding uncontrollably. An area of approximately 18 x 18 cm was heavily inflamed and covered in tiny fissures all over (pic 5). The tissue stretching various cm around the opening was purple, warm and stiff like cardboard, indicative of cellulitis (pic 5 & 9); Two tunnels of 4 and 5 cm exited in frontal direction under undermined skin. Three more tunnels would a few days later turn out to be present, albeit as yet inaccessible, due to firmly rolled down wound edges along the sides and distal end. The anatomical location of the tunnels corresponded to their location 2 ½ years (28 months) prior (pic 4). The visible part of the wound bed was necrotic and sloughy (pic 9).

As the infection was stretching at least 20 x 20 cm hidden under the strongly inflamed skin, the fact that the wound opening was so relatively small, was a complicating factor as this limited the access to the infection, to this opening and the fissures in the skin (pic 5). The depth of the infection and the extent of the network of hidden tunnelling was unknown but presumed extensive due to the wound's 3.5 years' history.

On Day 6, the cellulitis had resolved - thereby fending off the most acute risk of septicaemia - and so had the skin inflammation with the skin steadily returning to its original colour and pliability (pic 6 & 10). There was no malodour and the level of exudate had reduced to a level easily controlled when dressed with a single gauze-swab. The opening (pic 10) had reduced distally by one third, the cranial undermining had halved and the inside had granulated up from the bottom reducing the depth despite being the place where all the necrotic and infective material draining from the fistulas would be deposited before being lifted out of the wound with the daily thorough wash. After two months, the wound opening had granulated up to sub skin level forming a dip of the size of one third of the original wound opening. The dip was covered with protecting epithelium (pic 7 & 8 & 12).

The wound as such was healed (pic 7 & 8 & 12). The frontal fistulas had fused to one and was, together with the three other fistulas, draining infectious debris from the bone to the body surface into the former wound area, i.e. the dip. This happened through non-infected openings directly into non-infected epithelium (pic 8).. Epithelium is the optimal protection of the dip against the drained harmful substance.

Whilst awaiting surgery for osteomyelitis, the situation could be controlled and maintained with MPPT. This would reduce the risk of septicaemia as well as the amount of soft tissue that would need to be removed during surgery.

Part two – picture 13 – 29.

This treatment regime was, however, not installed. The wound only received some increasingly sporadic MPPT applications and, consequently, over the following year, the continuous flow of infective and cytotoxic debris from the bone-infection reinfected the fistulas including the soft tissue surrounding their paths. This was perceptible as inflammation at the level of the skin, increasing malodour, and exudate. Finally, this generated a renewed deep, festering wound in the exit area of the four known fistulas, i.e. the old wound area (pic 13).

The cranial end showed the old known fistula, the caudal end showed a distinct, long gorge splitting the epithelium potentially associated with the old, known caudal fistula (pic 4 & 8 & 13). Both sides along the opening were highly inflamed and nodulous, and, particularly the right side, seemed to contain a very broad belt of infiltration along the entire side spreading laterally to both sides apparently following the trajectory of the underlying fistulas. (pic 4 & 8 & 13).

MPPT treatment was reinstated, and the diffuse spread infiltrating large areas of soft tissue on both sides was stopped. Large amounts of debris were pushed out into the wound area via the fistulas and microbial toxins, with corresponding debris, were concentrated in clusters in the infiltrated skin surrounding the wound opening and also expelled (pic 14). (For an example of the controlled process of expulsion of toxins through the skin, see also pic 25 A-L).

This was part of the deep and extensive autolytic debridement that took place over the following weeks (pic 14 – 18). The infection in the skin was contained and controlled, and the processes of fighting it were initiated, including inside broad protruding nodules in the epithelium along both sides, as well as in the epithelium situated assumingly along the paths of the fistulas as well as in the soft tissue surrounding these. This fight against the infection, harboured in the nodulous structures, progressed steadily for many months (pic 14– 25). Eventually, the major part of the nodules subsided into smooth non-infected skin levelling with the body surface, and the grooves in the scar tissue became wider and less deep (pic 26), indicating that the infection removing and tissue regeneration processes were advancing successfully, including in the many-year-old pre-MPPT scar tissue (pic 5 & 27).

As the wound was cleaned out and new tissue could start granulating (pic 15 & 16), the presence of deep gorges running through the wound bed became even more evident (pic 13 – 29). Gorges are usually found in wound beds situated on top of osteomyelitis. The gorges are spaces through which the debris, generated by the infection in the bone, is regularly or continuously expelled. The tissue can appear closed when not actively passing debris. Gorges can often be identified in granulating tissue (pic 15 – 18). In infected non-granulating tissue, gorges are also present but rather as wide pockets of old slough and therefore harder to identify. Gorges can also be found in epithelialized areas in very old wounds coping with very old osteomyelitis underneath. In the present wound, such epithelium developed inside the dip to protect the tissue at the exits of the fistulas against the infective and corrosive properties of the debris (pic 19 - 29). This approach by the immune system mimics the one taken two years earlier when creating an epithelialized dip the first time (pic 8) when the osteomyelitis had not spread quite as far inside the bone. Such epithelium develops late in the stages of removing the infection from the soft tissue.

The cleaning of the wound and clearing of the infection also made it gradually easier to identify the four known fistulas (pic 20 – 24 & 26 - 29). These gradually ceased moving locations and aligned with the centre line of the wound, the body, and the underlying bone (pic 26 – 29). This could indicate that the path of the fistulas between the bone and the skin surface had now become more direct.

During the entire process of infection removal, tissue regeneration (healing) and maintenance, the wound intermittently expelled small quantities of debris, usually distinctly coloured either red, potentially red-pigmented toxins from *S. marcescens* or red by-product from the haemolytic activity of *S. aureus*; or yellow

potentially from *S. aureus*, or sometimes tinged green potentially from *P. aeruginosa*. Such expulsions were much more frequent and of larger quantities during the infection combatting period and would then abate to sometimes being imperceptible.

The nodulation would occasionally expel red, presumably toxins, directly through the skin, i.e. not through the fistulas leading into the pit. After each expulsion, the affected area would rapidly recover to its original controlled state, except that the relevant nodule would have reduced a little bit in volume and the skin would look a little bit improved (example in pic 25 A-L).

Over time, however, despite the absence of soft tissue infection, the expulsions seemed to increase very subtly, presumably in accordance with the rate of spread of the osteomyelitis inside the bone.

All pictures: Up: cranial. Down: distal. Left: left. Right: right.

--------------- Part one ---------------

All pictures: Up: cranial. Down: distal. Left: left. Right: right.

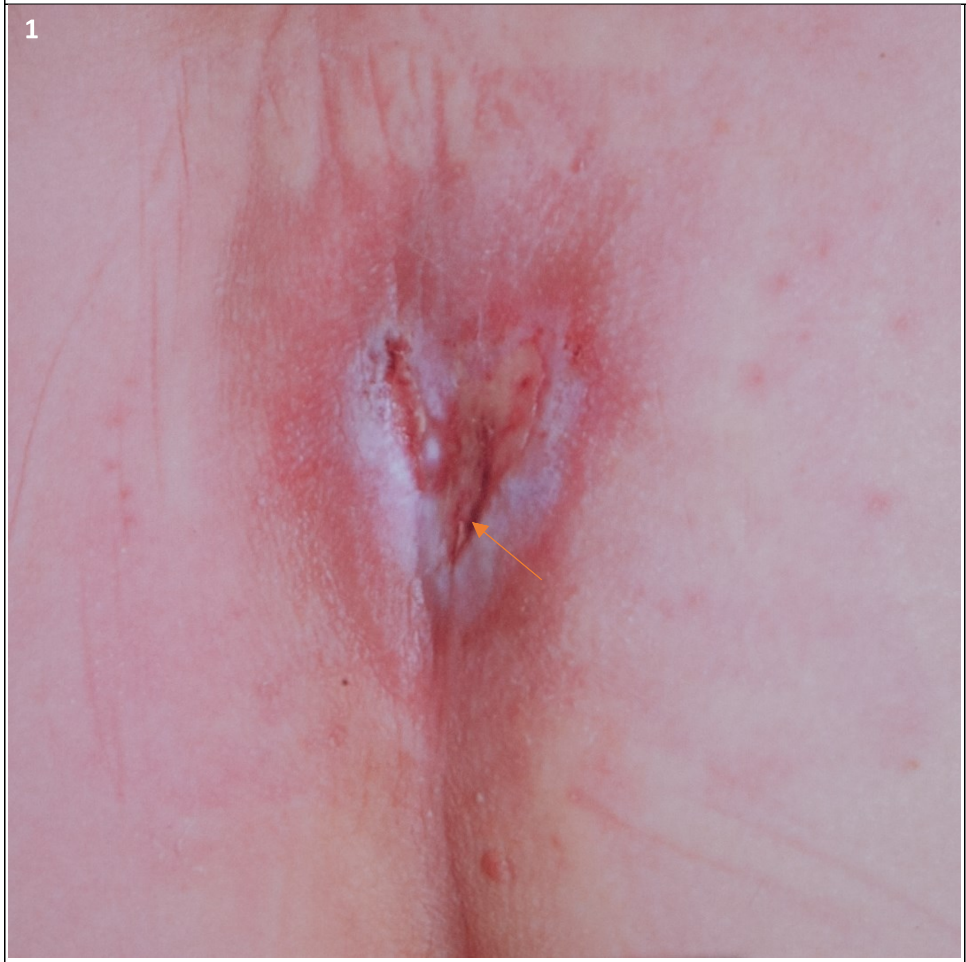

Day minus-1226 2.5 month old ulcer

3 years 4 months before start of MPPT

The ulcer developed within the first few days of the accident causing the spinal injury. It did not seem deep but the arrow points to what could be a deep reaching albeit narrow rupture, similar to a puncture wound. Narrow ruptures reaching far into muscle, have anaerobic conditions combined with necrotic tissue (even if only small amounts) favouring infection. As they reach deep, they represent a high risk of causing osteomyelitis if the infection is not immediately brought under control.

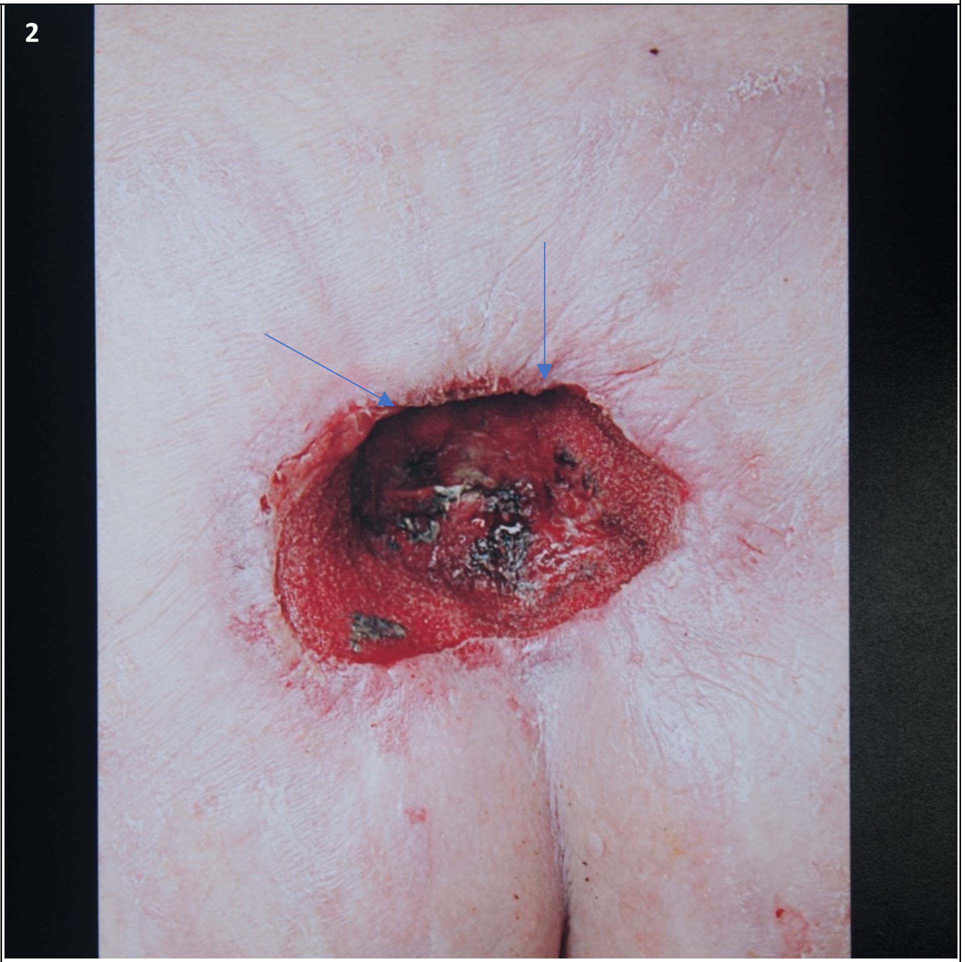

Day minus-1026 8 months old ulcer

2 years 10 months before start of MPPT

After NPWT with and without saline instillation while in hospital.

The *blue arrows* point to tunnels from soft tissue infection leading into the wound bed.

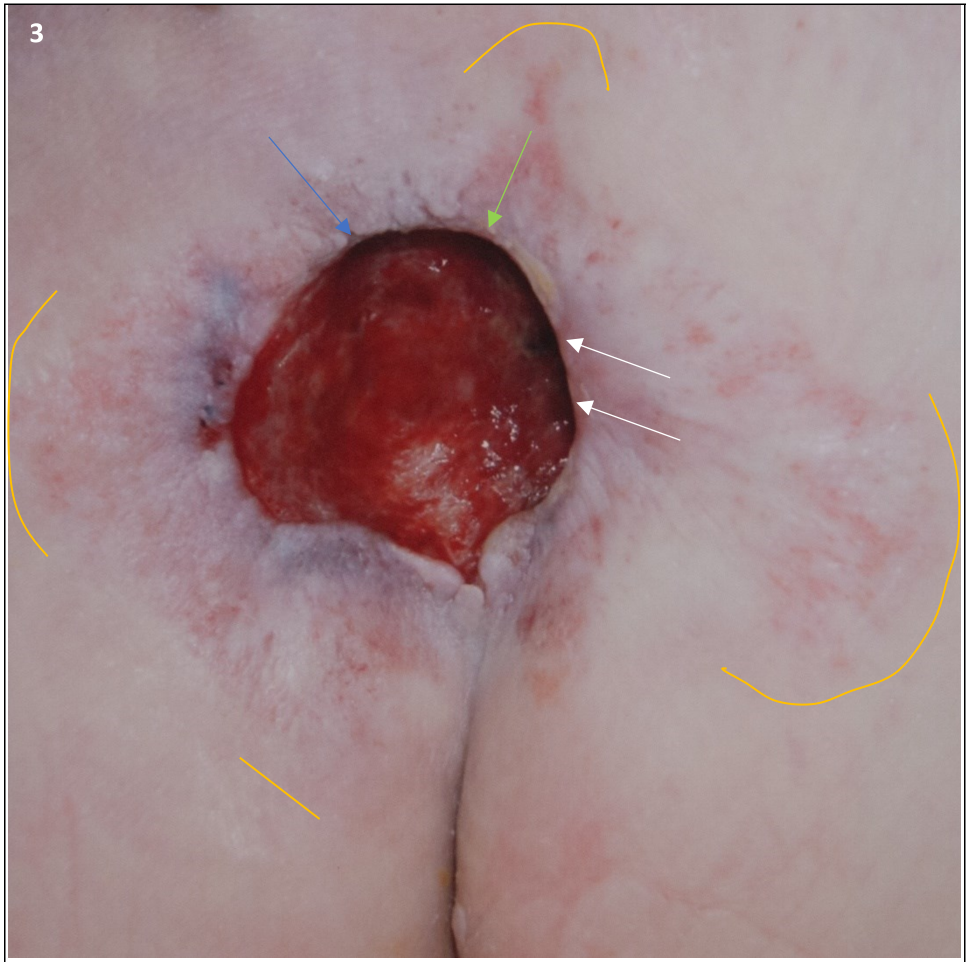

Day minus-963 10 months old ulcer

2 years 7 ½ months before start MPPT

Arrows: Tunnelling leading into the wound bed along the edges.

Yellow lines indicate how the infection is infiltrating in and underneath the skin in the precise area that, 2 ¾ years later, still remains extensively infected – though deeper and with more extensive tunnelling, by then.

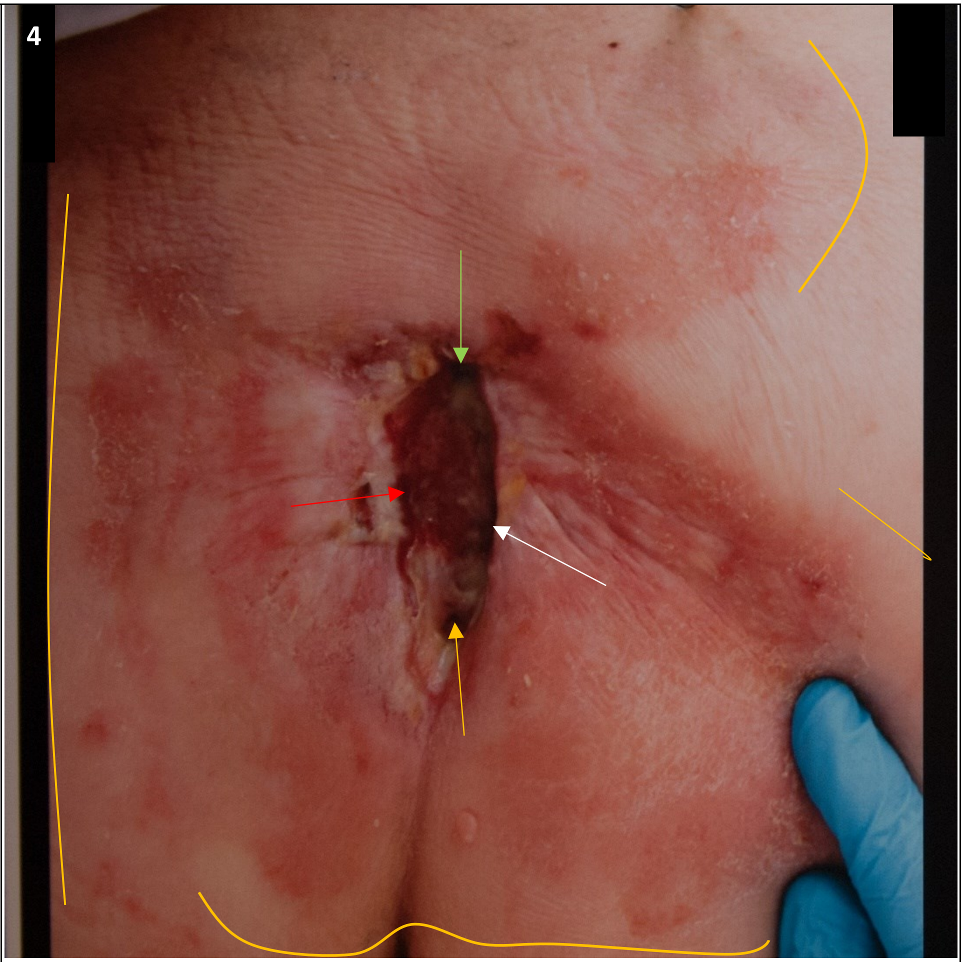

Day minus-841 14 months old ulcer

2 years 3 ½ months before start of MPPT

Deep spreading tissue infiltration is reflected in the skin as inflammation.

Yellow lines indicate how the infection is spreading in the precise area that remains extensively infected 2 ¼ years later – though deeper and with more extensive tunnelling, by then.

Green arrow: frontal fistula

White arrow: right side fistula

Yellow arrow: caudal fistula – long gorge splitting the epithelium

Red arrow: Left side fistula

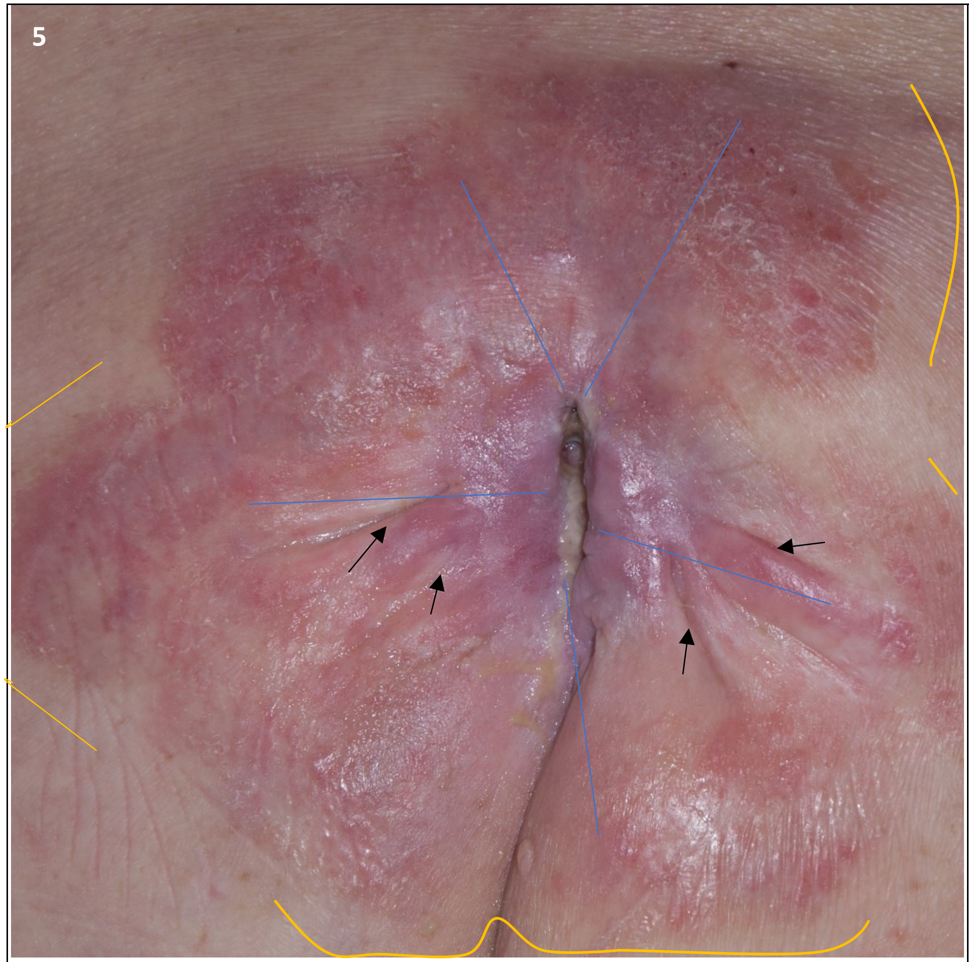

Day 0 3 ½ years old ulcer

Just before first MPPT

Malodorous; very high exudate level; cellulitis with broad purple edges;

20 x 20 cm heavily inflamed area with red, warm and swollen skin containing mini fissures.

Yellow lines: examples of the border of the inflammation

Blue lines show the location and direction of the tunnelling and the depth at which they are wide enough to be probed.

Black arrows: deep grooves in old, elevated scar tissue.

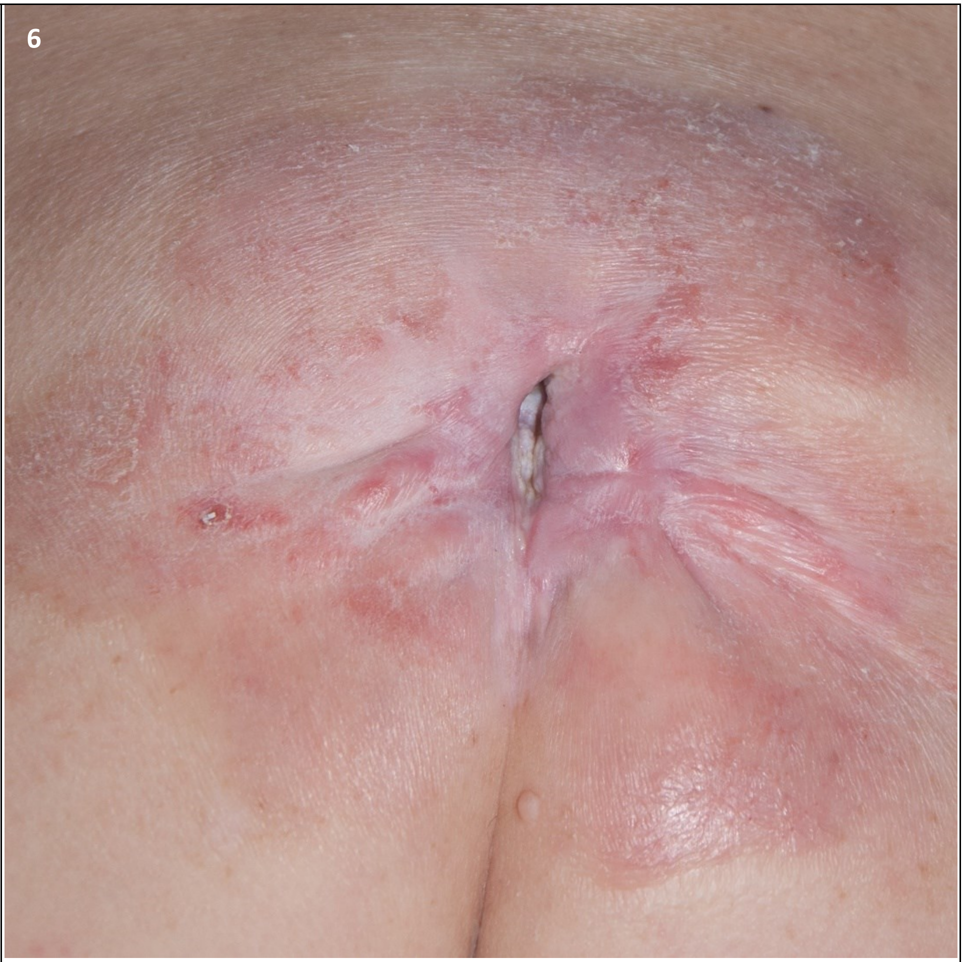

Day 6

Odour has disappeared.

Exudate level is manageable with a single gauze swab as secondary dressing.

Cellulitis has resolved.

A clean, deep gorge is running craniocaudally in the length of the wound bed

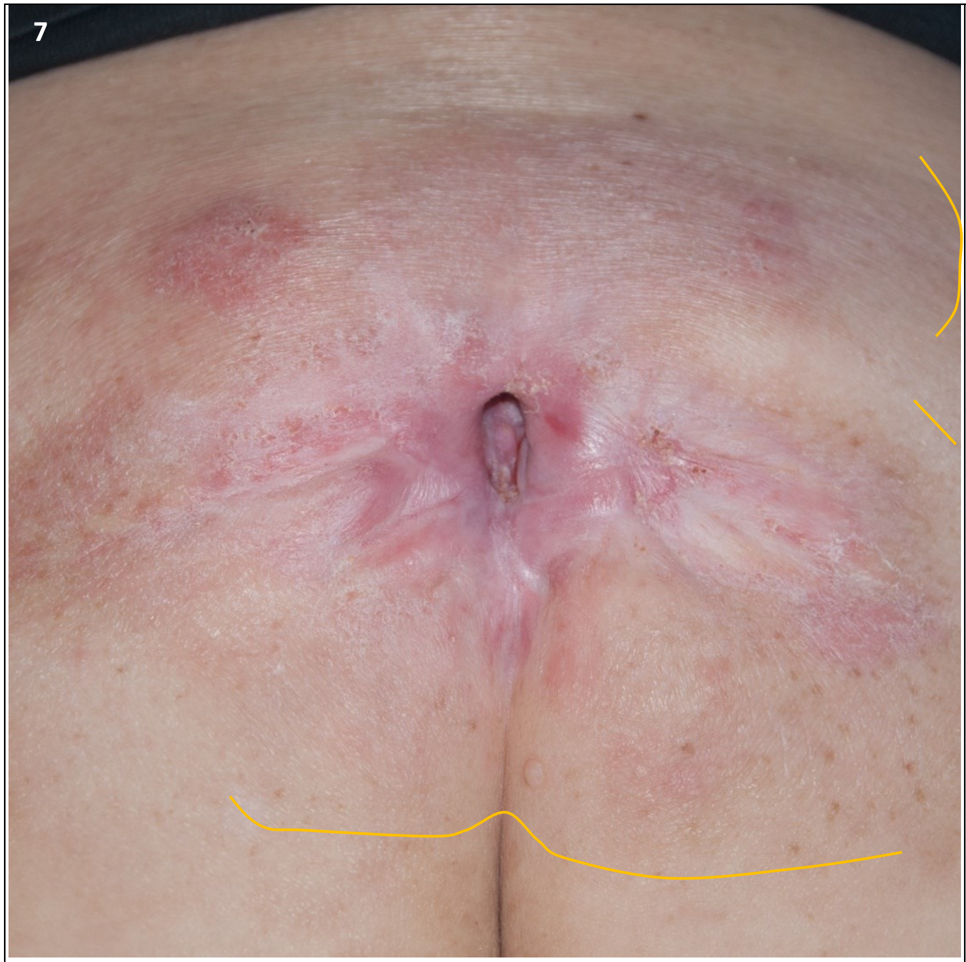

Day 59

Yellow lines are in the same anatomical position as those in pic 3 taken on Day 0

The old scar tissue is remodelling and levelling with the skin, and has thereby made the deep grooves practically disappear.

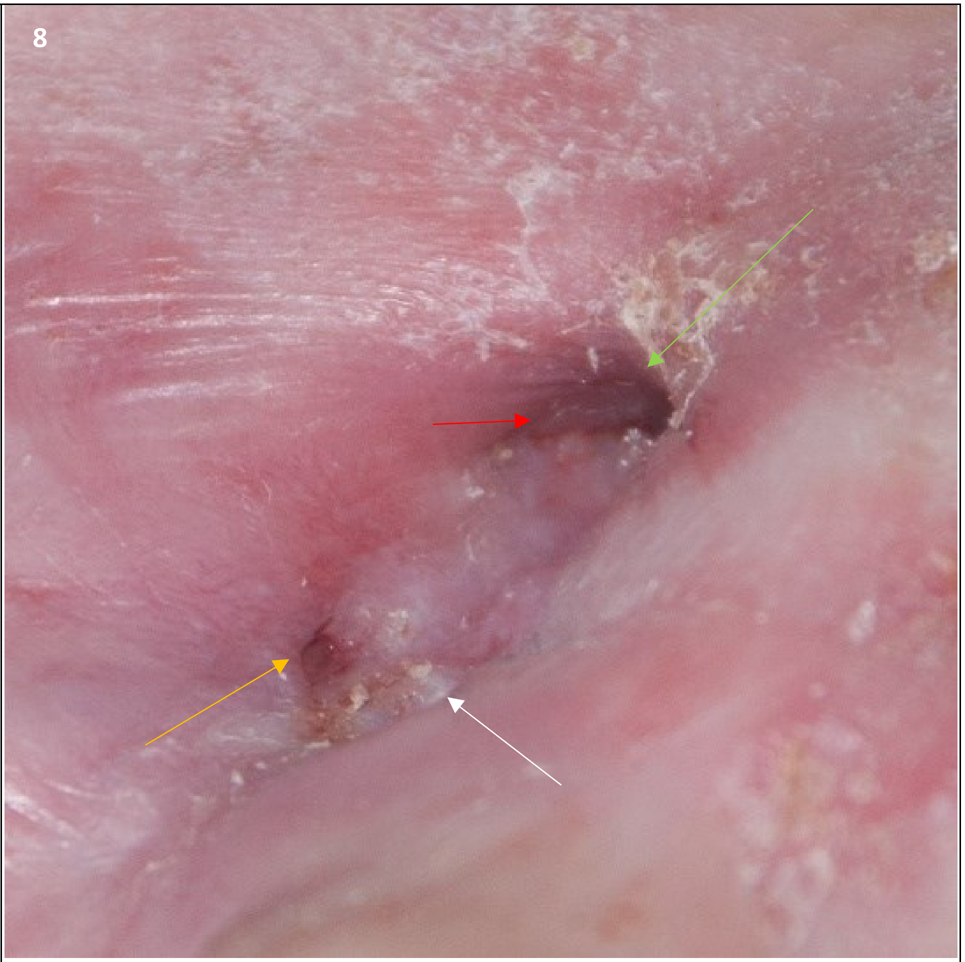

Day 59 Closeup of dip

Wound fully epithelialized except for the three original fistulas leading down to the osteomyelitis.

Green arrow: frontal fistula

White arrow: right side fistula

Yellow arrow: caudal fistula

Red arrow: Left side fistula

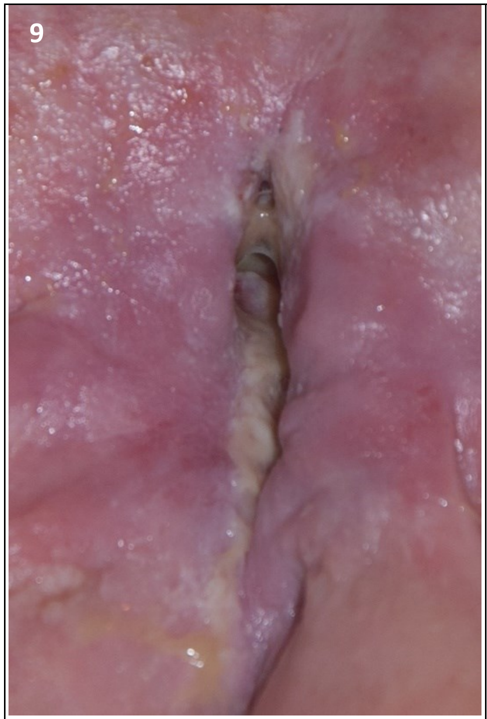

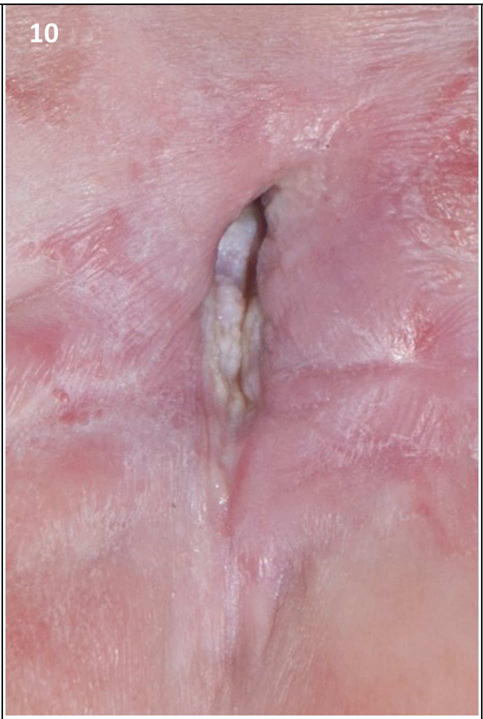

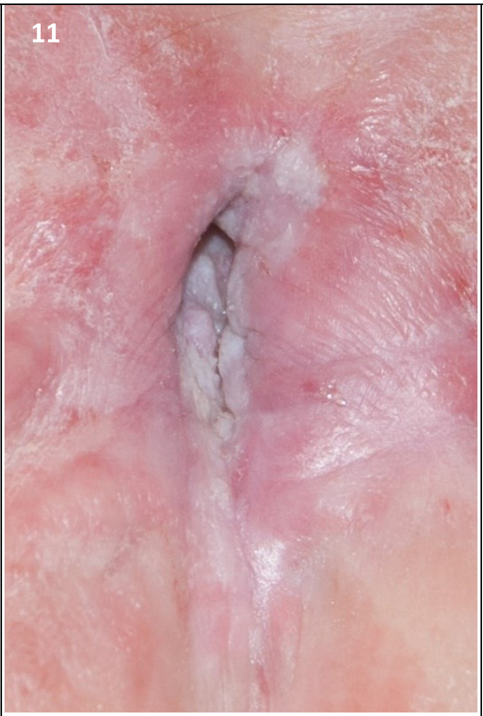

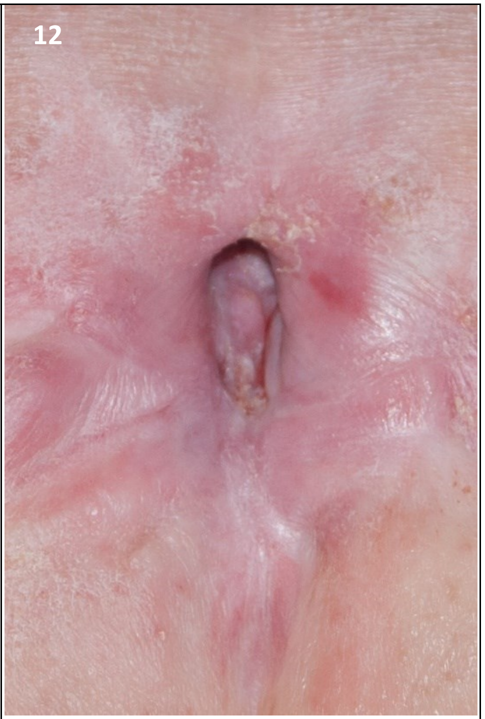

Day 0	Day 6	Day 20	Day 59
Heavily exuding. Malodorous.<br><br>Cellulitis – skin stiff, purple and hot.<br><br>1 cm broad undermining along cranial and entire right edge.<br><br>Necrotic wound bed with gorge.<br><br>2 tunnels accessible in cranial corner.<br><br>Firmly rolled down wound edges.	Cellulitis cleared – skin pliable and pink.<br><br>Wound edges have been unrolled and wound bed is pink and regenerating.<br><br>Gorge is clean.	All undermining halved.<br><br>Gorge clean and granulating profoundly.	The wound and surrounding skin and soft tissue are cleared of infection.<br><br>Wound bed has transformed into a pit covered with epithelium. 4 fistulas are draining as required into this pit without displaying signs of infection. The fistulas are too narrow to be probed. The amount drained is minimal.<br><br>No undermining.

--------------- Part two ---------------

After approximately a year (11 months) with only very sporadic use of MPPT

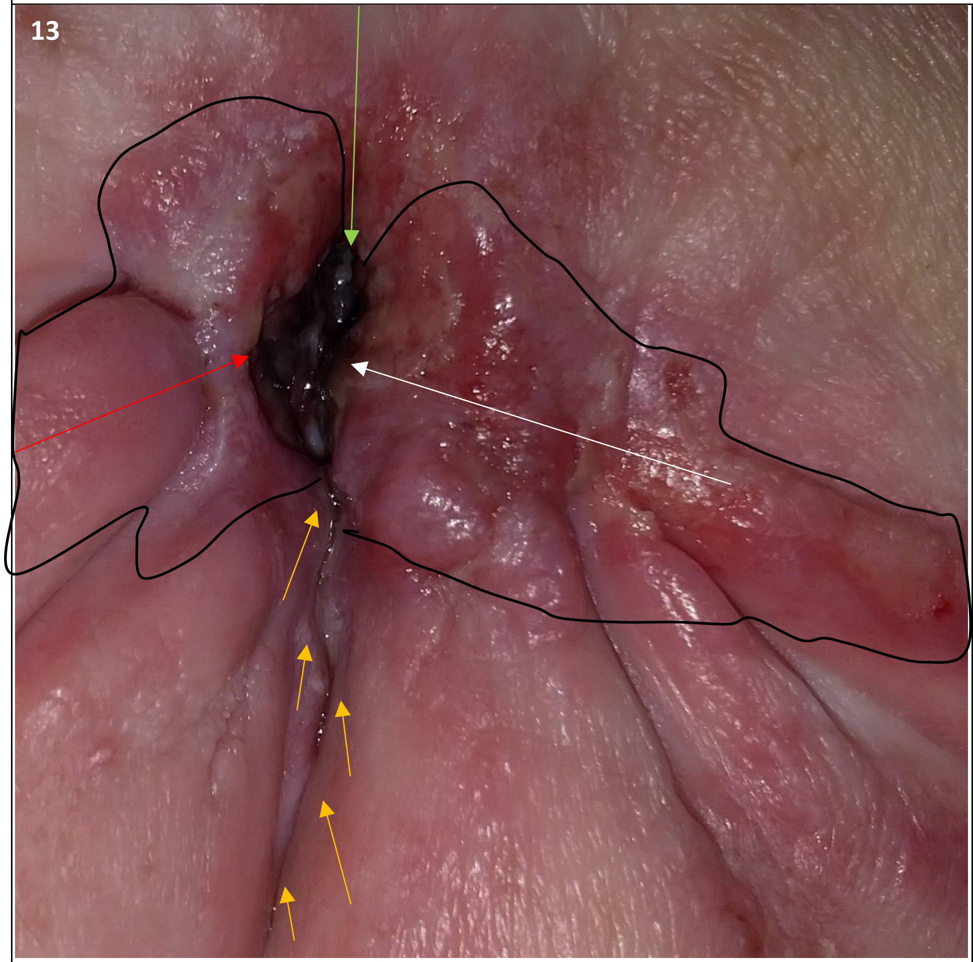

Day minus-2

After nearly a year, MPPT treatment is reinstated 2 days after this picture.

black: Broad skin infiltration and infection with distinct nodulation.

Green arrow: frontal fistula

White arrow: right side fistula

Yellow arrow: caudal fistula – long gorge splitting the epithelium

Red arrow: Left side fistula.

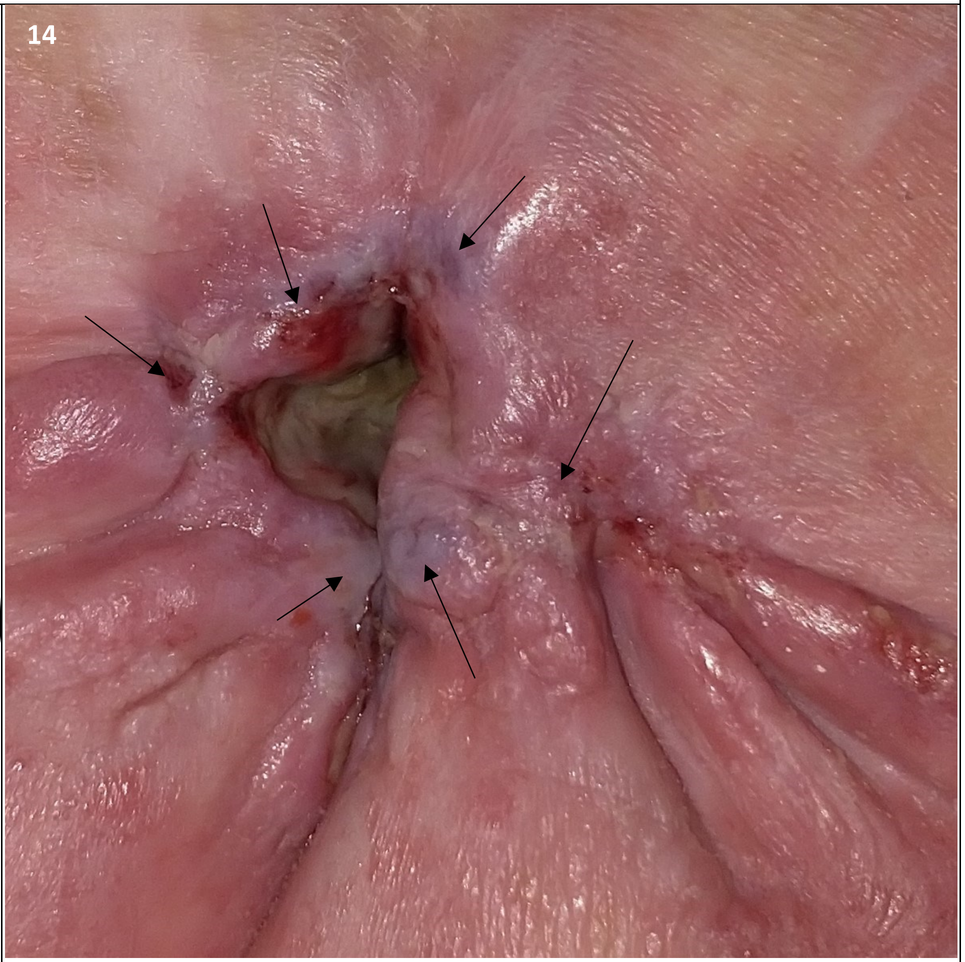

Day 2

Clear limitations of the diffuse infiltration in the skin which was spreading outwards with the wound as central focus. Sloughy wound bed.

Black arrows: concentrations of toxins and harmful by-products on different stages of the process of being collected, concentrated, transported toward the surface, and expelled, preventing them from causing further harm to the tissue.

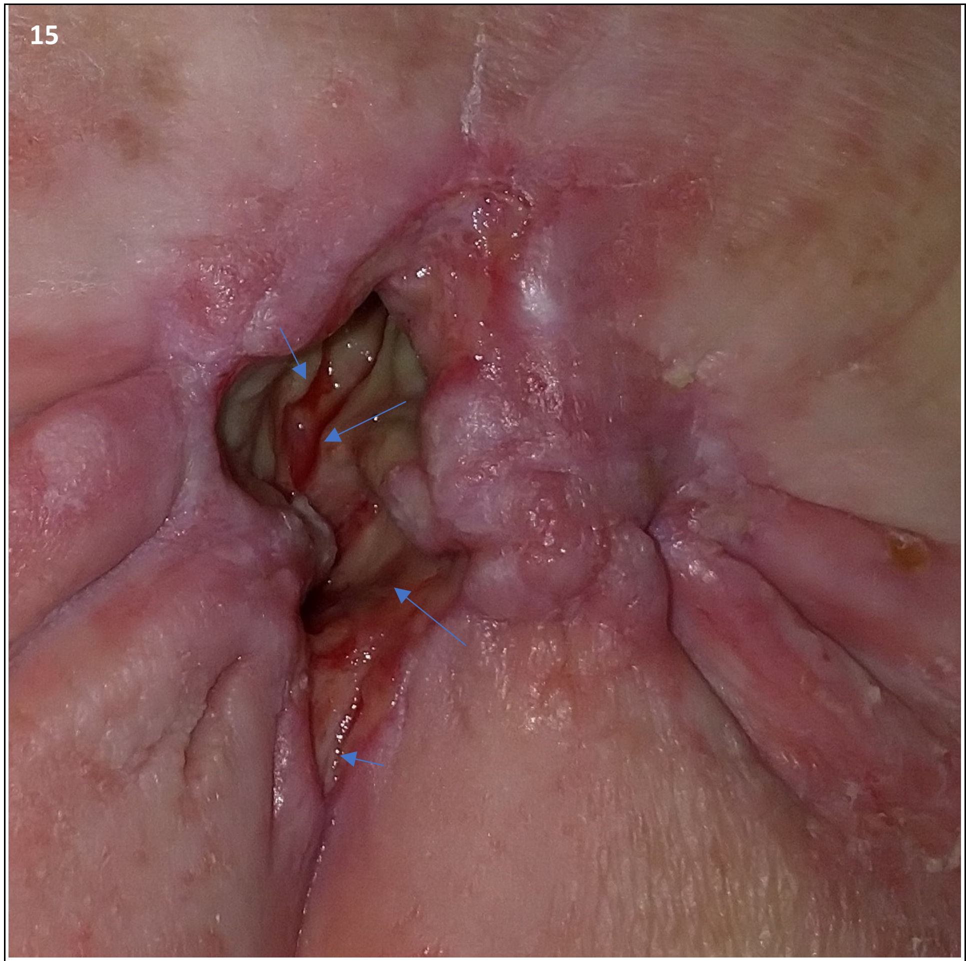

Day 8

The non-viable tissue is being removed via autolytic debridement and the infectious material in nodulous dermis is contained. The border of the skin infiltration is distinct, indicating that the spread has stopped (is no longer diffuse).

Blue arrows: Examples of gorges in the wound bed.

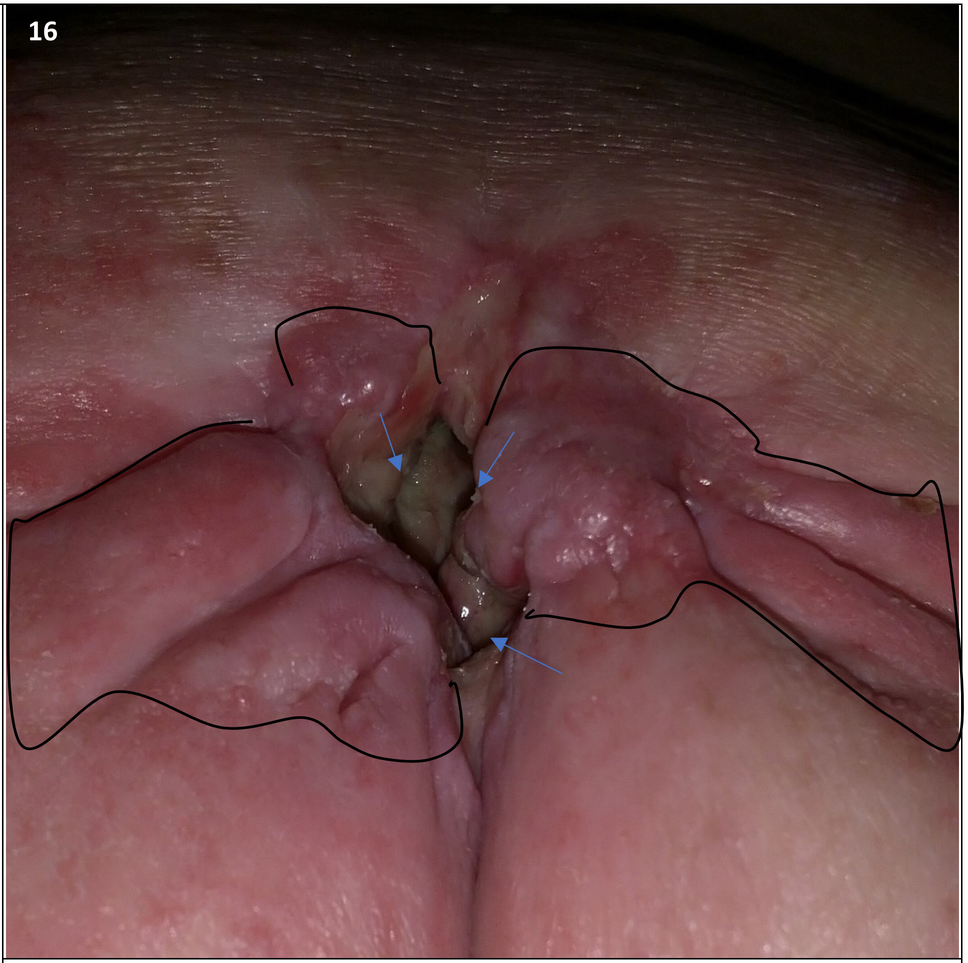

Day 9 view from side (not from usual above)

Autolytic debridement exposes the plenty of deep gorges in the wound bed as well as large nodulous congregations leading into strongly swollen and puffed up dark red to purple inflamed skin with deep groove formations.

Blue arrows: Examples of very deep gorges in the wound bed that are quite wide because they are being cleaned of the old slough.

Black encircled: Nodulous skin and puffed-up, inflamed skin with grooves.

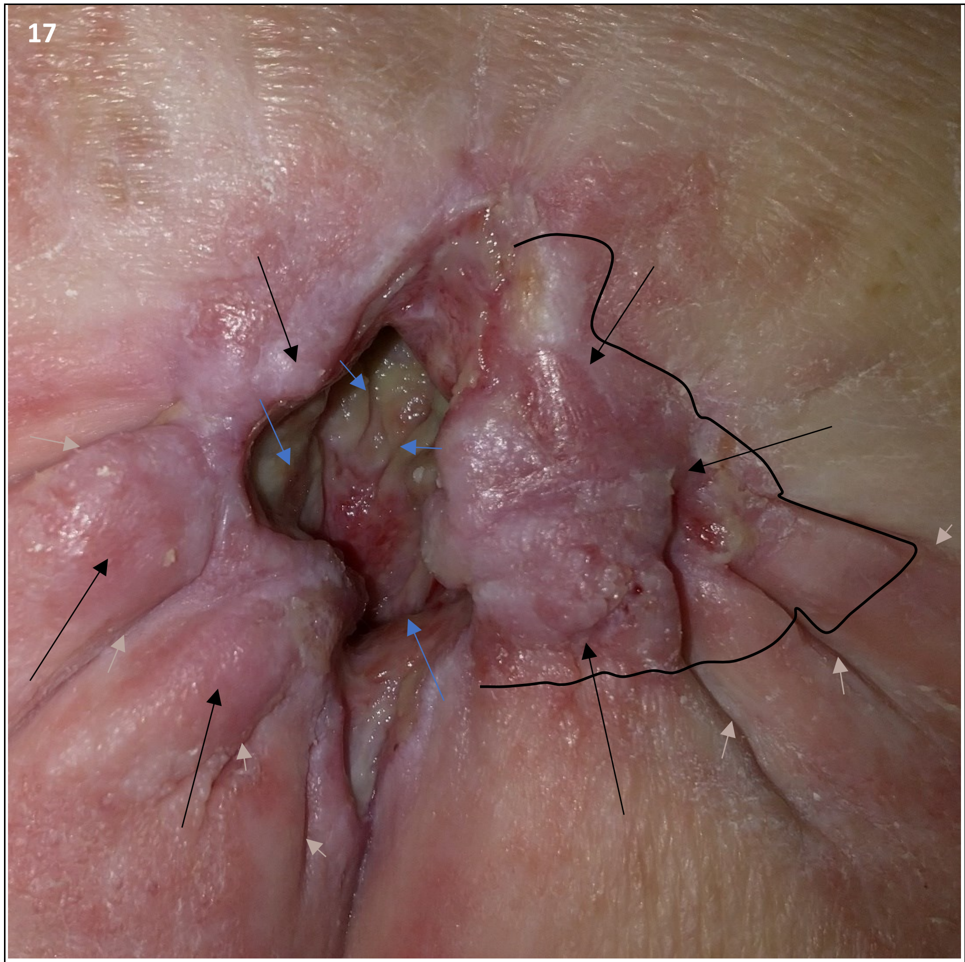

Day 13

Infection spread has been halted and the skin is no longer inflamed beyond the strong nodulation. Nodulation is evident and shows the border of the infection in the skin.

Autolytic debridement is advanced with granulation evident in wound bed as well as in wound sides.

Blue arrows: Examples of gorges in the wound bed.

Black arrows and encircled: Nodulous puffed up skin. Concentrations separated by deep grooves (*light brown arrows*).

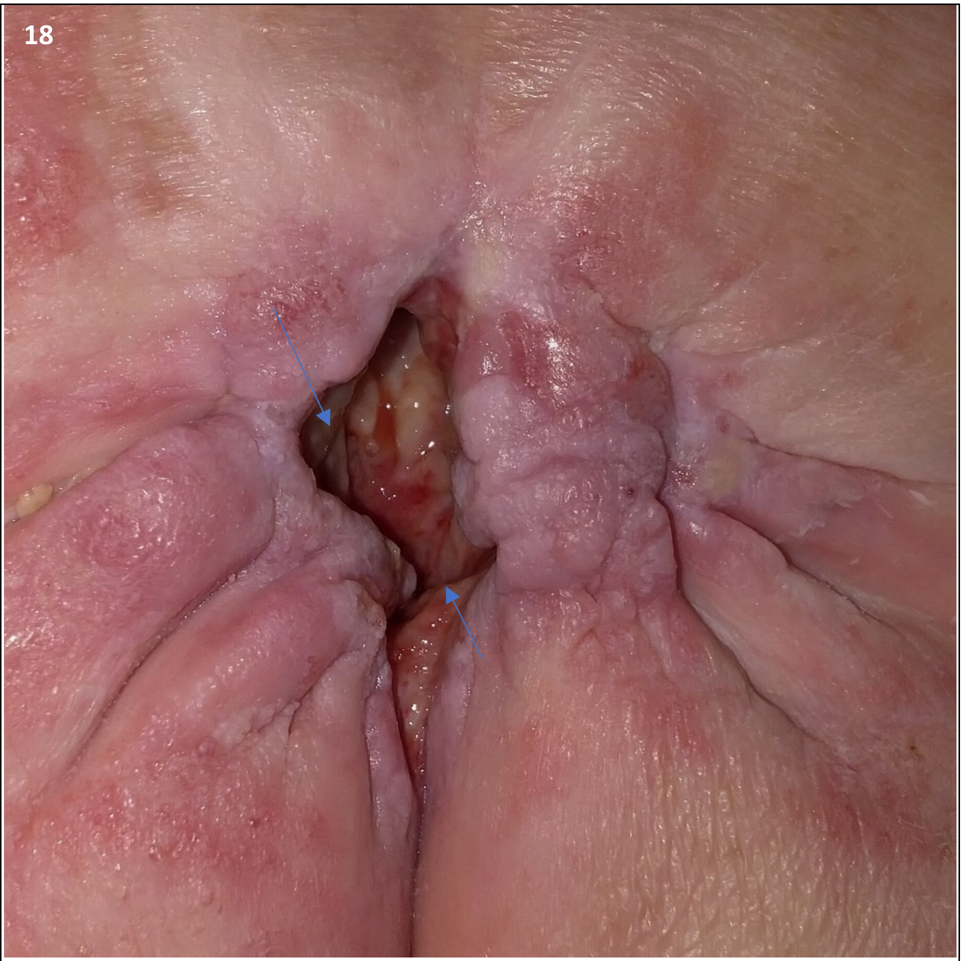

Day 26

Wound bed is now clean, granulating and reducing in size. Gorges remain present

Skin continues to regenerate, and nodulation is gradually reducing.

Blue arrows: Examples of deep gorges in the wound bed.

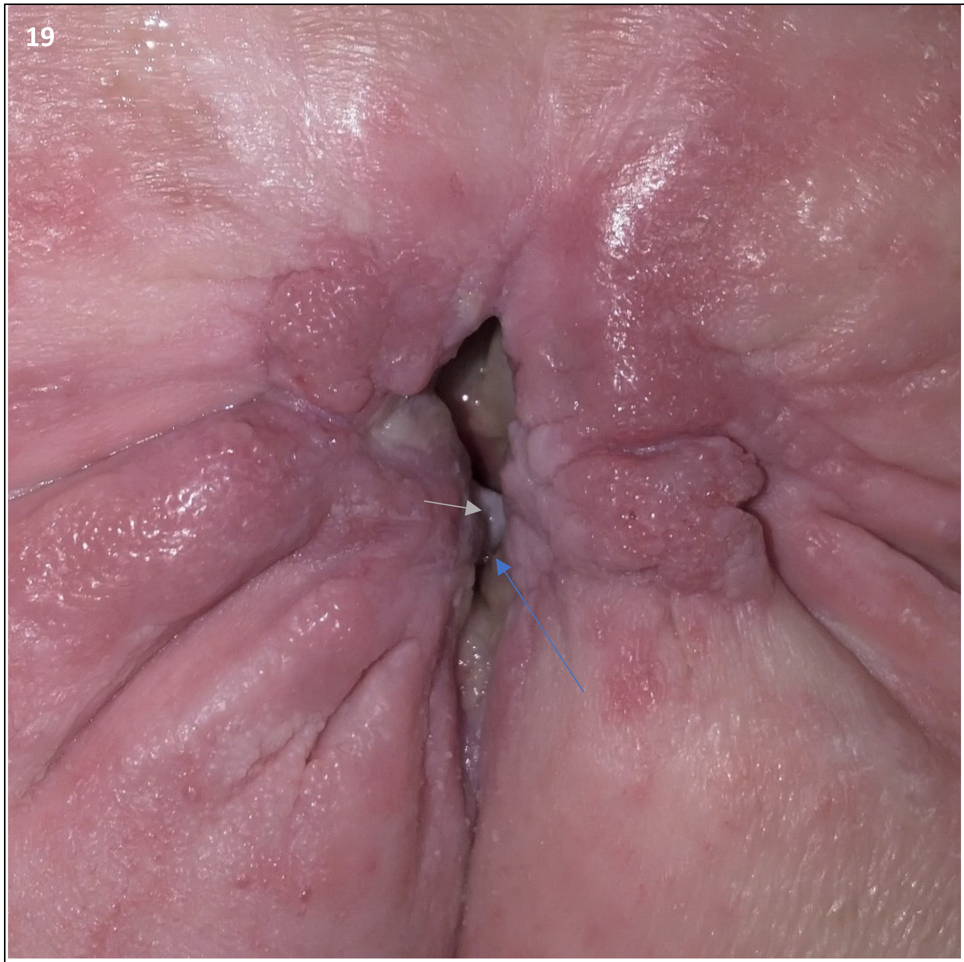

Day 90   3 months

Wound has granulated up from the bottom and epithelialised along the edges, including the left side which is now restored with new, smooth epithelium.

Blue arrow: Example of deep gorge in the wound bed.

Grey arrow: Epithelium appearing in wound bed / pit.

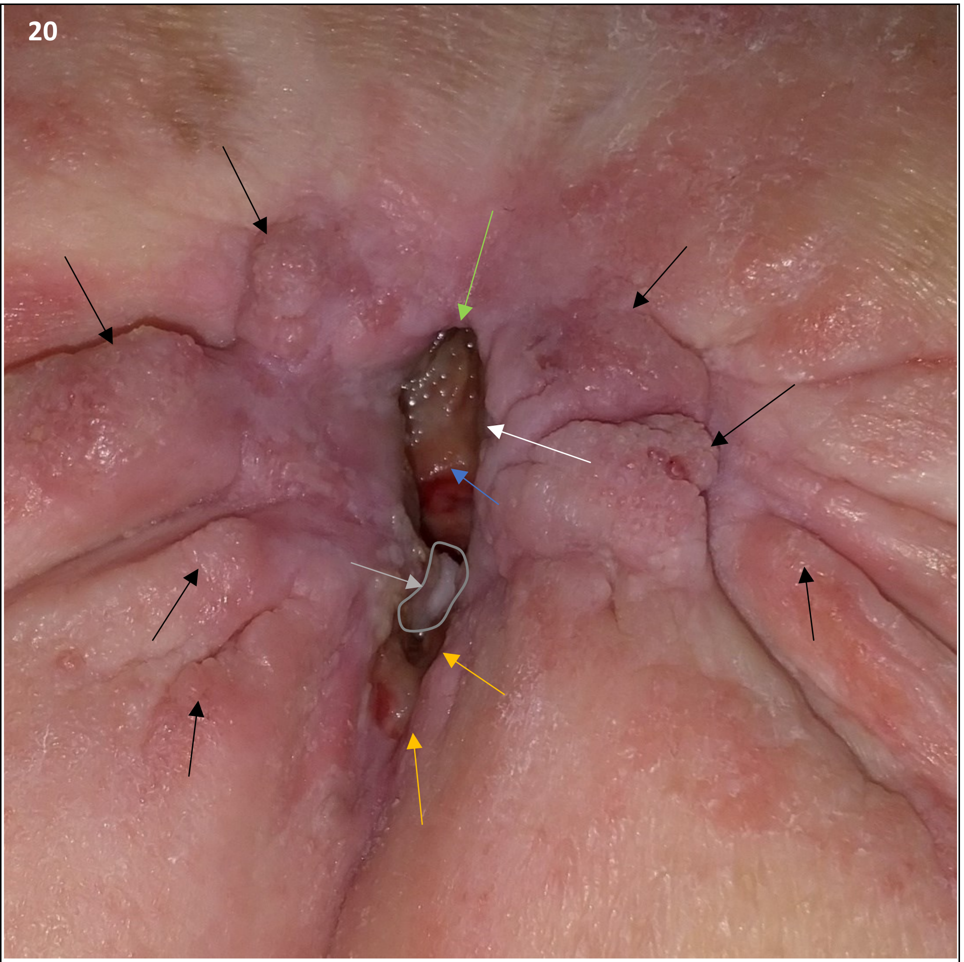

Day 107   3 ½ months

Wound has granulated up further. The reduced infection of the tissue allows the fistula exits to show themselves. Epithelium is moving into the pit between the frontal and distal fistulas to protect the soft tissue in the wound bed.

Green arrow: frontal fistula

White arrow: right side fistula

Yellow arrow: caudal fistula – long gorge splitting the epithelium

Grey arrow and encircled: Epithelium in wound bed / pit.

Blue arrow: Example of gorge in the granulating tissue

Black arrows: Examples of nodulous skin concentrations separated by grooves.

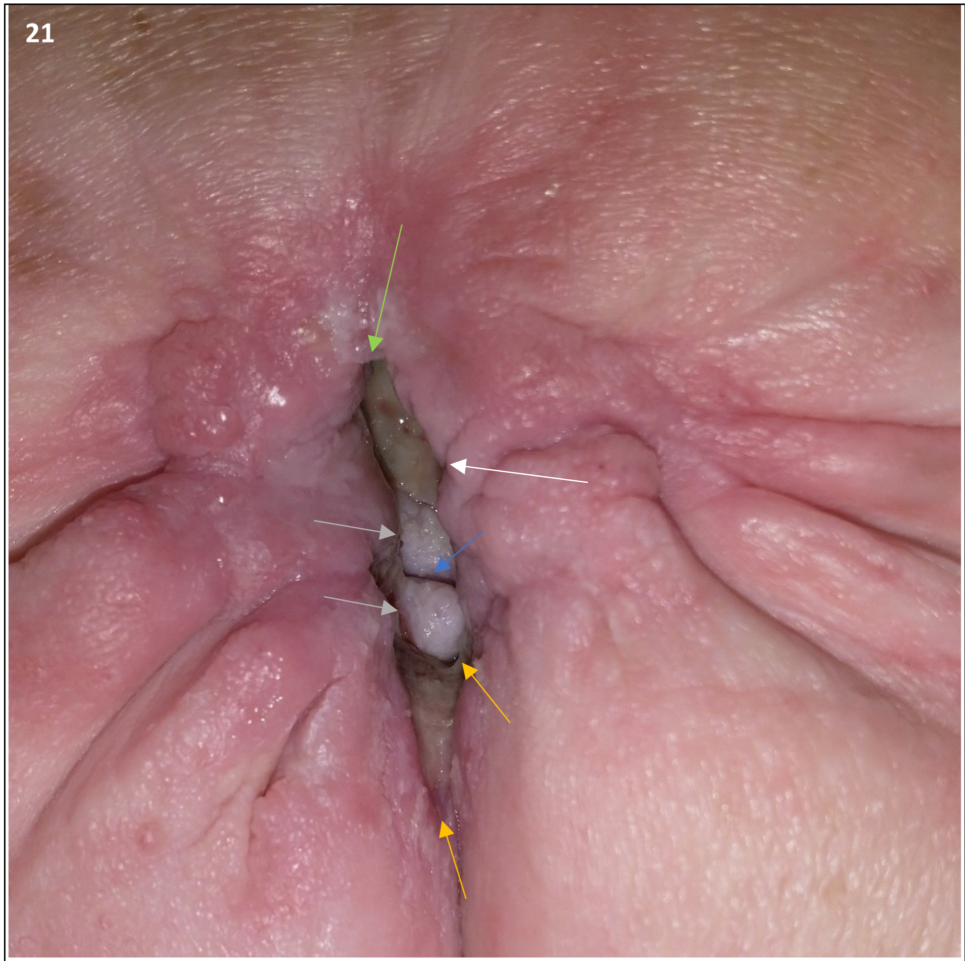

Day 154 5 months

Protective epithelium in the middle of the wound bed grows but with a dividing gorge.

Green arrow: frontal fistula

White arrow: right side fistula

Yellow arrows: caudal fistula

Grey arrows: Epithelium in wound bed.

Blue arrow: Example of gorge in the epithelium

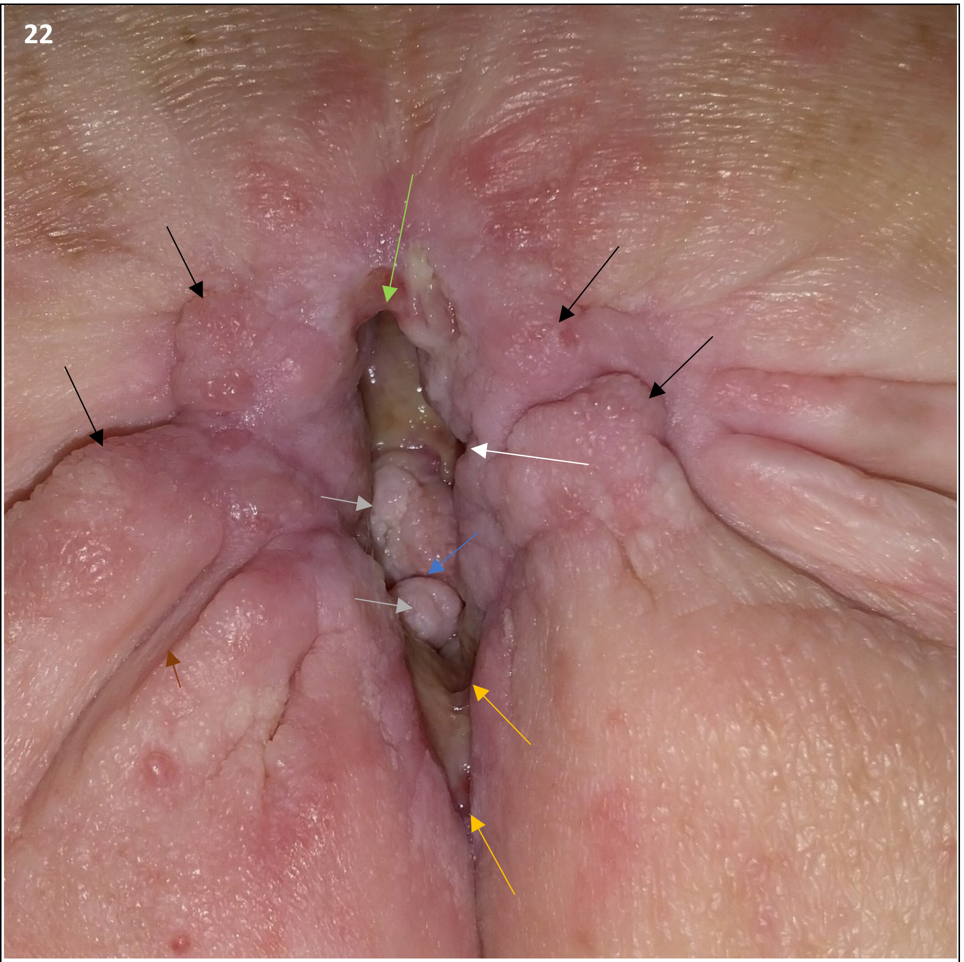

Day 185 6 months

The general inflammation is gone. The wound continues to protect the sides and central part of the wound by further epithelialisation. The nodulation is still prominent but steadily reducing. This is recognisable through the decreased width and height of the nodulation and the increased width of the separating grooves.

Length of wound bed: approx. 5 cm

Green arrow: cranial fistula – Can be probed at 3 cm.

White arrow: right side fistula – Can be probed at 2 cm

Yellow arrow: caudal fistula – Not sufficiently cleared to be probed yet; Is probed one month later at 9.5 cm.

	Grey arrows: Epithelium in wound bed<br><br>*Blue arrow:* Example of gorge in the epithelium<br><br>*Black arrows:* Examples of nodulous skin concentrations separated by increasingly wider grooves (e.g. *brown arrow*).

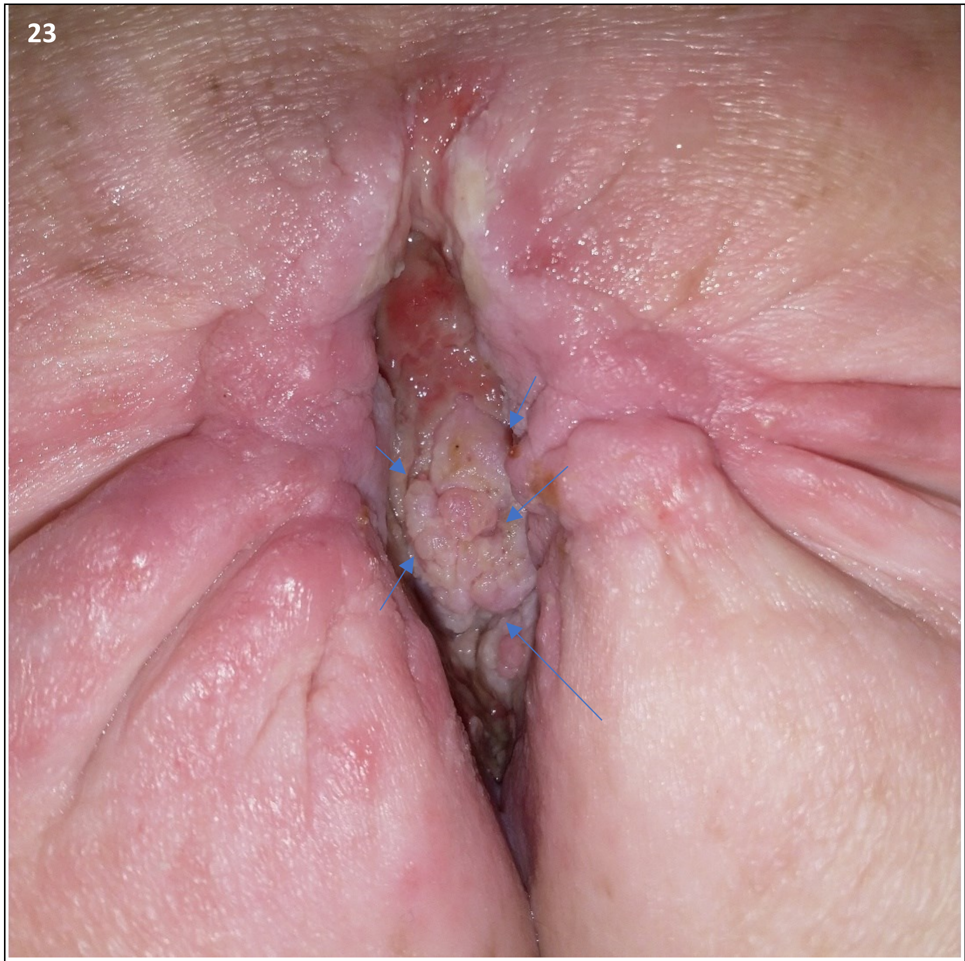

Day 294 9 ½ months

Epithelium is more prominent, but gorges are evident. The gorges largely coincide with the known fistula exits.

Nodulation is still reducing.

Frontal part is granulating well. The tissue along the two distal fistulas, i.e. deep down and out of sight, is undergoing heavy cleaning, daily emptying large quantities of very old debris into the dip.

Blue arrows: Examples of gorges in the epithelialized dip.

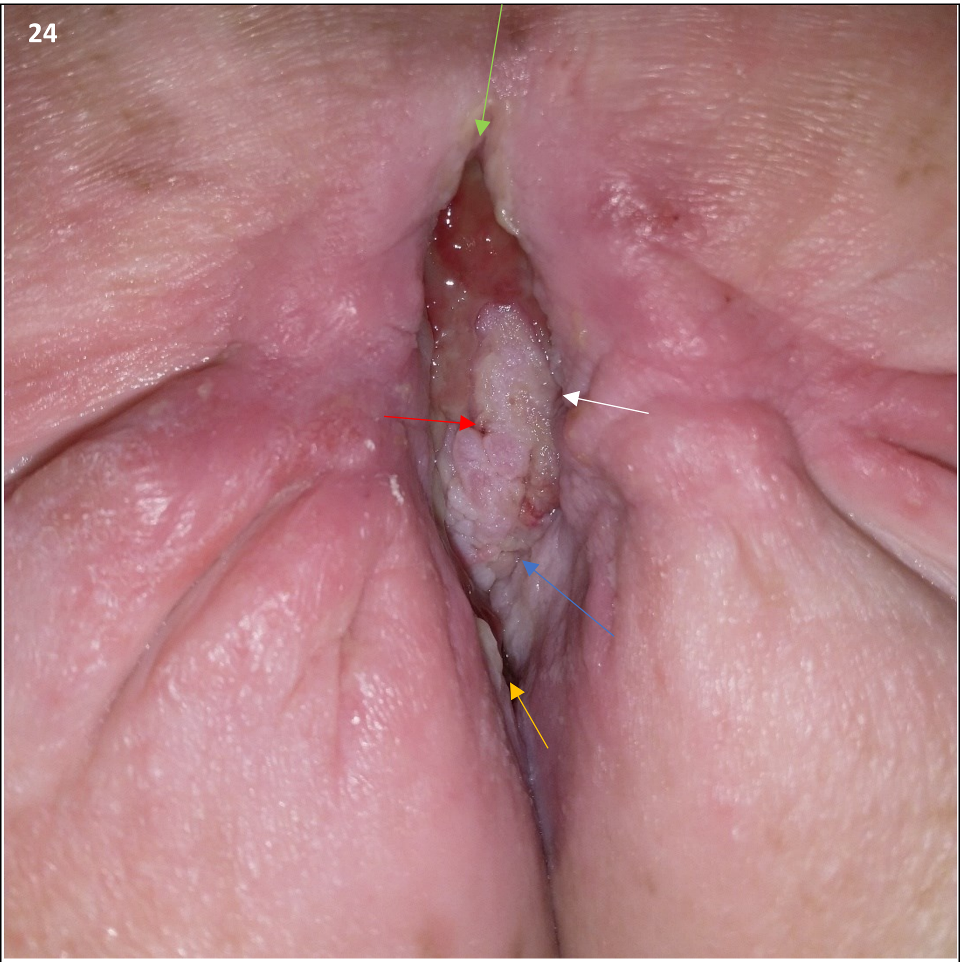

Day 328 10 ¾ months

Epithelium is now protecting the internal sides of the dip. The bottom of the dip is also being protected by epithelialisation with enough flexibility to open - to let the infective debris from the bone infection, pass as needed - and then reclose. This protects the bone and soft tissue.

The dip area is reducing in size.

The nodulation continues to reduce and its appearance is smoother despite the clusters still elevated above skin height. Their dark red appearance in a few areas reveals that they still contain infection and should not be mistaken for “uneven scar tissue”.

Green arrow: cranial fistula.

	Yellow arrow: caudal fistula.<br><br>*Red arrow*: Left side fistula<br><br>*Blue arrow:* Example of gorge in the epithelialized dip.

Picture 25 A-L

The picture series start on Day 340 (11 months) in the morning and extends over a period of 16 days with the hours between pictures displayed

The following sequence of closeups show an example of toxins - produced by the infection inside the nodulation - being concentrated; transported to the surface of the dermis; expelled directly through the epidermis; and the skin reclosing immediately after finishing. All steps follow within hours of each other.

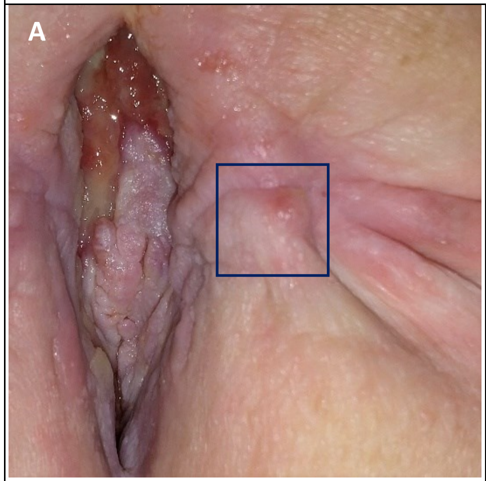

02.01 morning Hours 0
The location of the nodulation in all the following closeups.

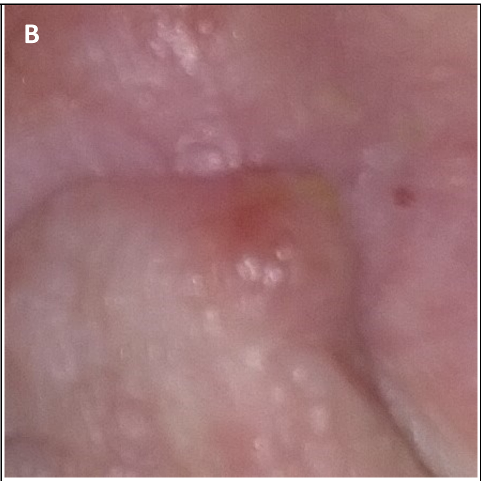

02.01 morning 0
Toxins concentrating and pushing towards the surface.

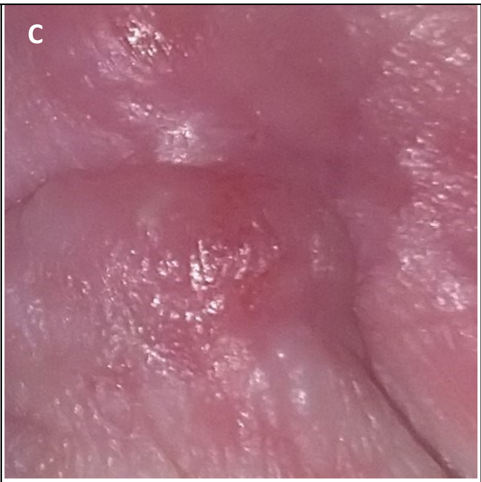

02.01 evening +12
Concentrating and pushing outwards.

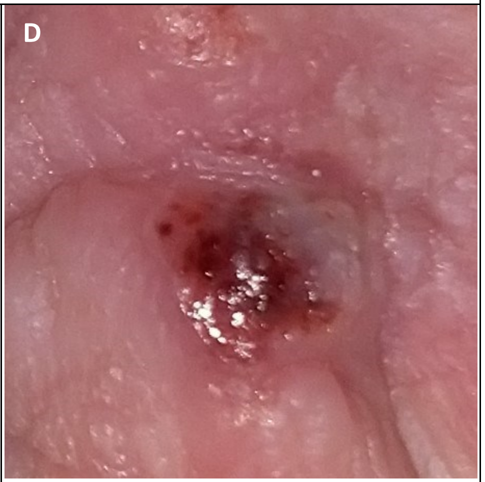

04.01 morning +36
Concentration has reached dermis.

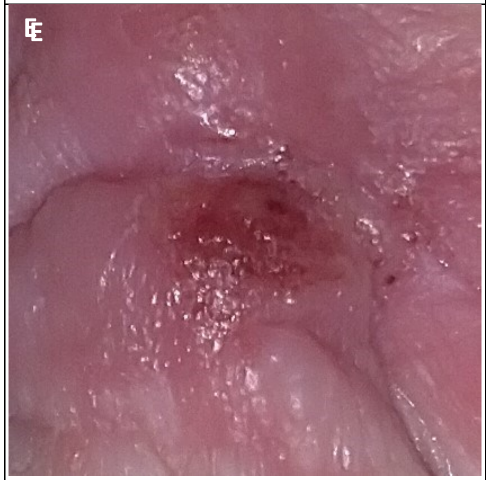

04.01 evening +12
Concentration reached dermis and is starting to break through.

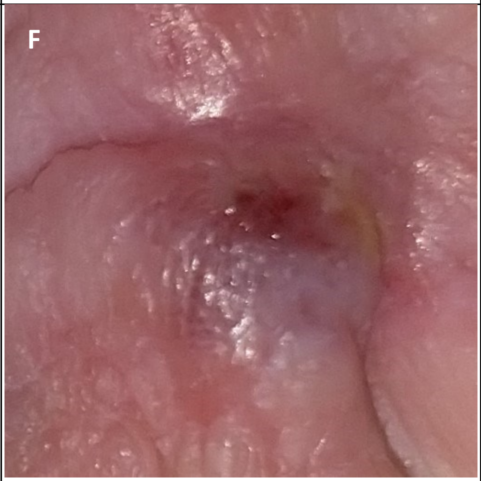

07.01 morning +60
Concentration breaking through epidermis.

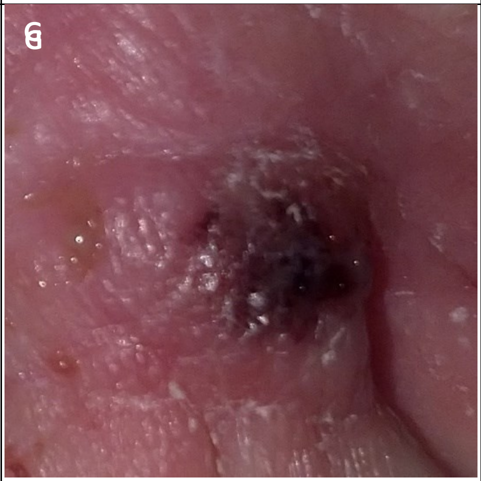

07.01 evening +12
Concentration expelling toxins through epidermis.

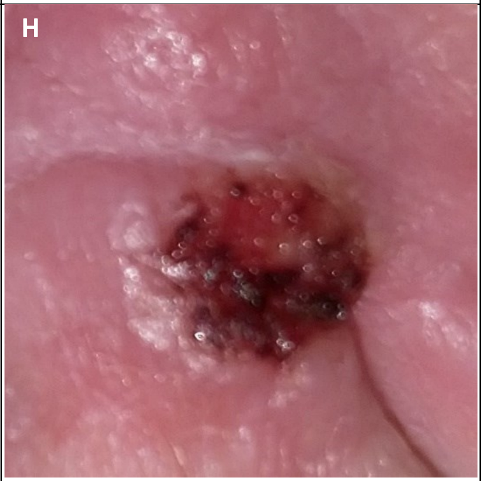

08.01 evening +24
Finishing expelling toxins through epidermis.

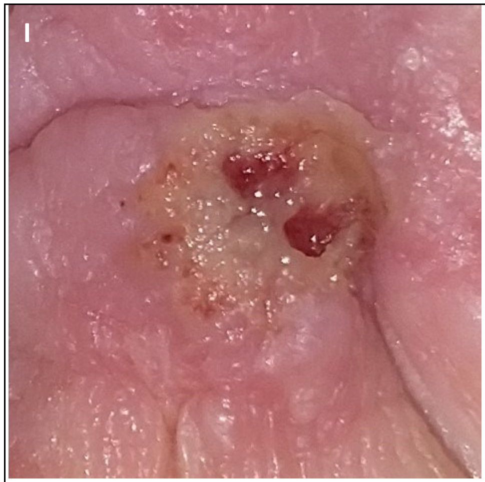	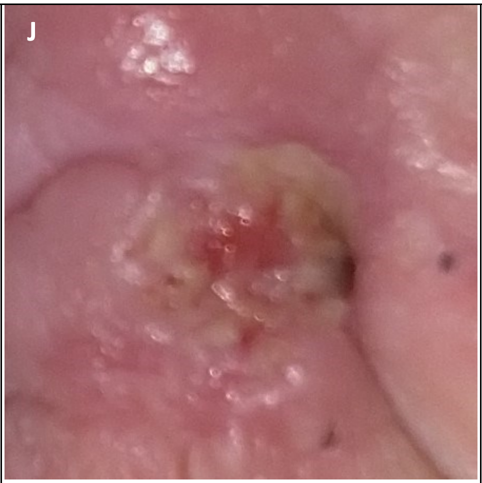	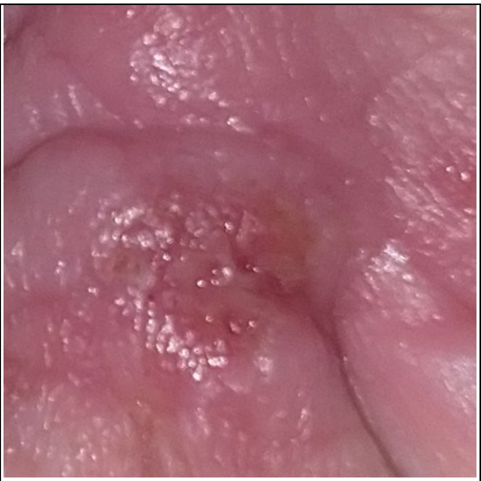	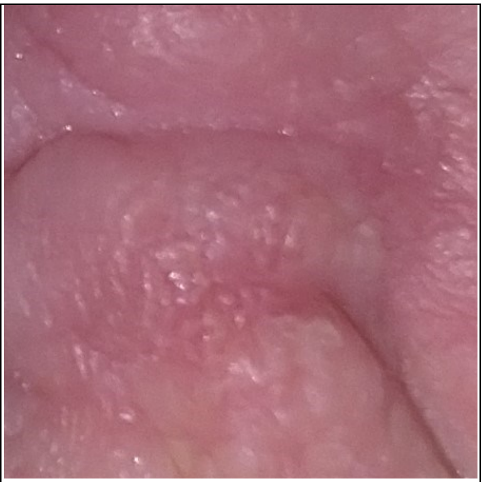
09.01 morning +12<br>Nodule epithelium regenerating.<br>Granulation visible in the two openings	10.01 morning +24<br>Nodule granulating and epithelialising	10.01 evening +12<br>Epithelialising	17.01 evening +168 / 1W<br>Fully back to pre-expulsion state

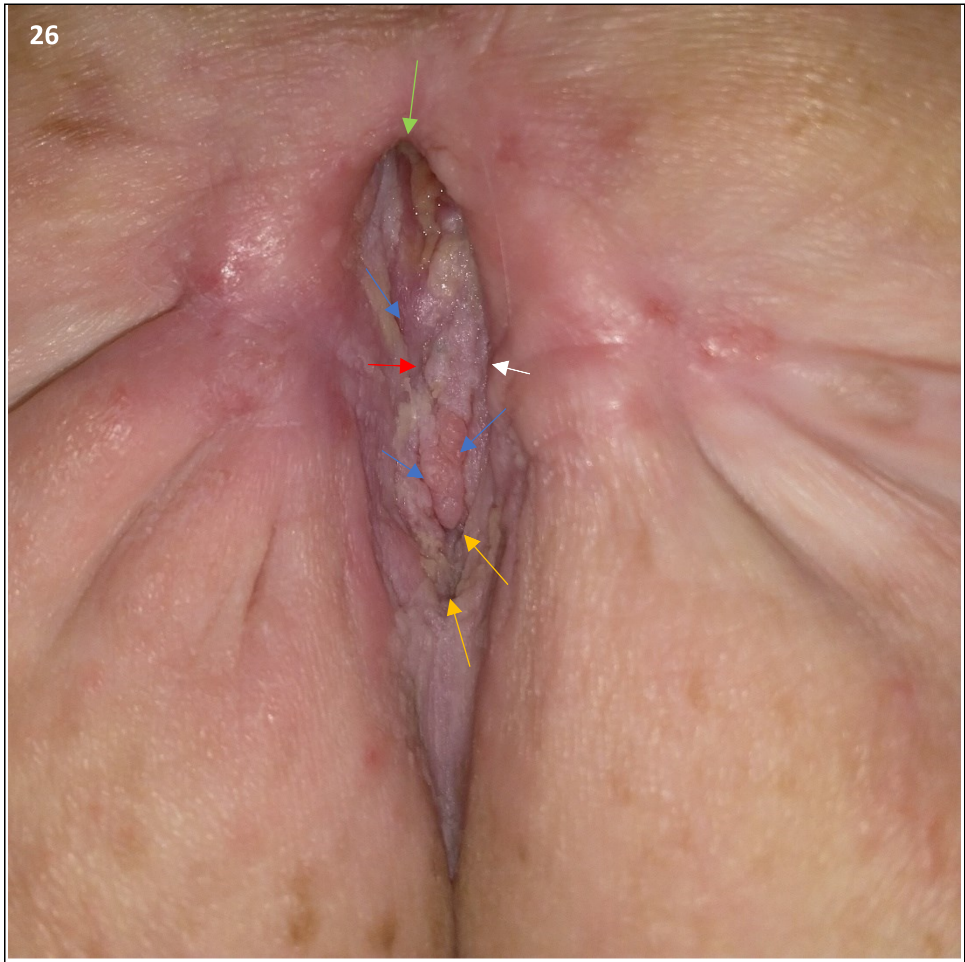

Day 449 1 year 2 ¾ months

The dip is practically completely covered by protective epithelium and has reduced to a length of approximately 3cm. The gorges open and reclose when infective debris, generated by the infection in the bone, is expelled.

The nodulation is practically gone. The skin is smooth and no longer inflamed and the remaining much hollower grooves are remains of the prior existing scar tissue and can continue to regenerate for many months or years.

All four fistulas are visible with clean non-inflamed exit holes and aligned with the midline of the wound dip, the body midline and the underlying bone. The cranial fistula exit into granulation tissue; the other fistulas into epithelium. All four fistulas are too narrow to be probed.

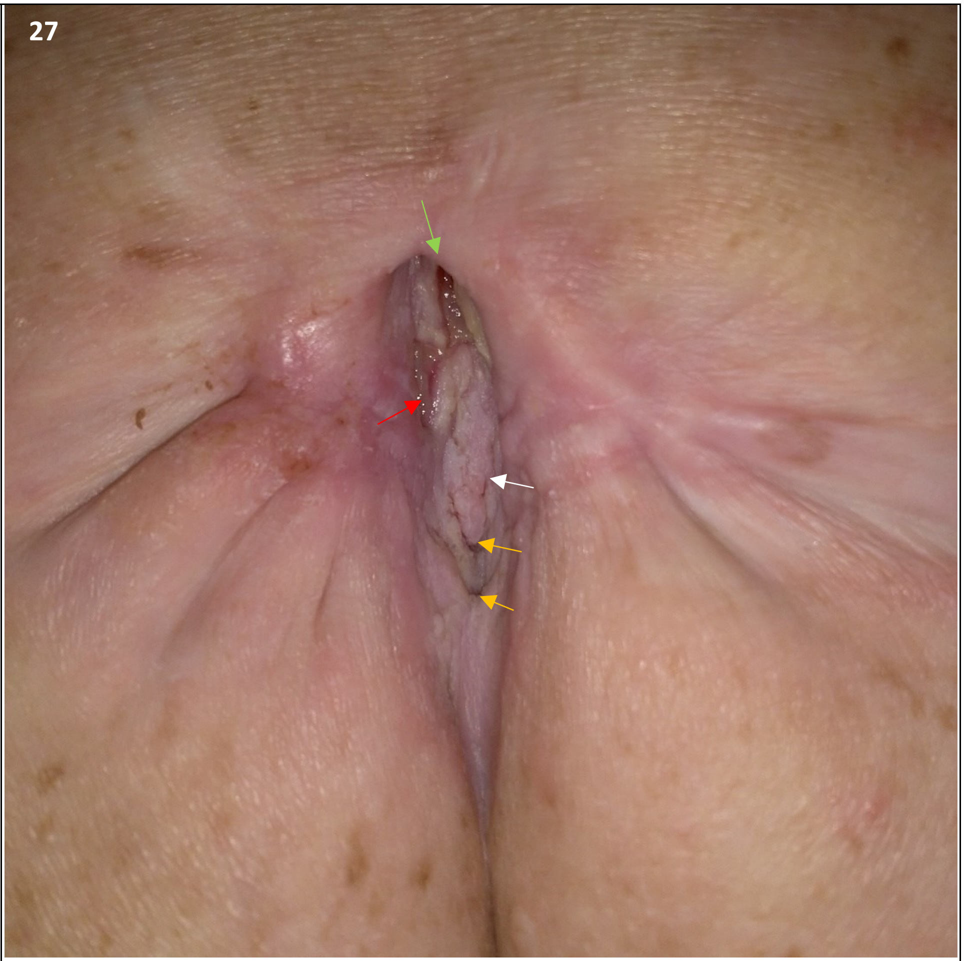

Day 489 1 year 4 months

The mostly epithelialized dip receives and controls the daily expulsion of debris from the 5-year-old osteomyelitis through the fistulas and the gorges in the epithelial and granulation tissues. The area is maintained free of infection. This reduces the risk of septicaemia to the risk posed directly by the osteomyelitis.

Surrounding skin is maintained healthy without tissue infiltration, inflammation or nodulation.

Green arrow: cranial fistula.

White arrow: right side fistula.

Yellow arrows: caudal fistula.

Red arrow: Left side fistula

Green arrow: cranial fistula.<br><br>*White arrow:* right side fistula.<br><br>*Yellow arrows:* caudal fistula.<br><br>*Red arrow*: Left side fistula.<br><br>*Blue arrows:* Examples of gorges in the epithelialized dip.	

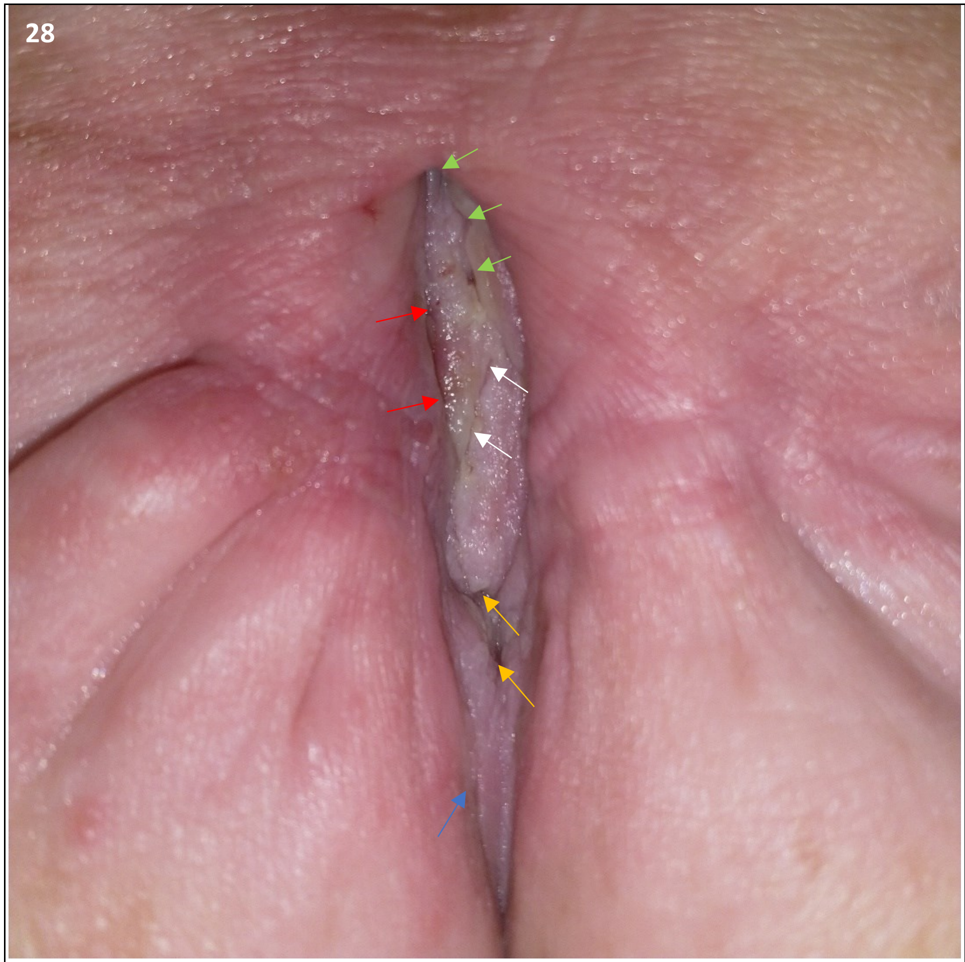

Day 535 1 year 5 ½ months

The fistula exits are gradually turning into short, clean, infection-free gorges showing the flexibility to open and close as required. The gorges are relatively centred on the cranio-caudal axis, in line with the underlying bone.

Surrounding skin is maintained healthy without any issues.

Green arrow: cranial fistula-gorge.

Red arrow: Left side fistula-gorge.

White arrow: right side fistula-gorge.

Yellow arrow: caudal fistula-gorge.

Blue arrows: Example of gorge.

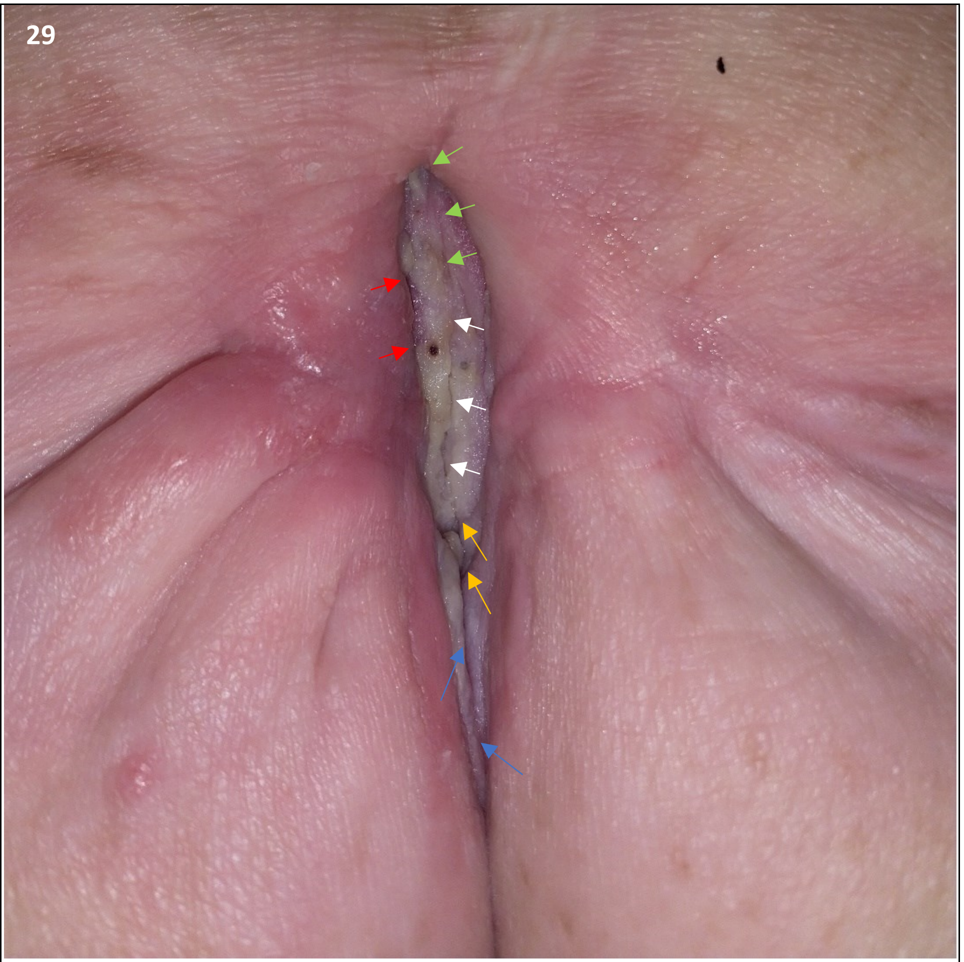

Day 604 1 year 8 months

Maintenance continues whilst waiting for osteomyelitis surgery in non-infected soft tissue.

The fistula-gorges are centring along the midline directly above the bone.

Surrounding skin is maintained healthy without any issues.

Green arrow: cranial fistula-gorge.

White arrow: right side fistula-gorge.

Yellow arrow: caudal fistula-gorge.

Red arrow: Left side fistula-gorge.

Blue arrows: Example of gorge.

Wound number 33			Patient	SCI	
**Grade 4**	**5 years 2 months**	**Ischium Pubis**	**66-year-old male**	**50 years**	**C5 complete**

The right ischial tuberosity had suffered from pressure ulcers in the past, that had always closed, eventually. This grade 4 pressure ulcer, however, dates back over 5 years to a hospital stay for a colonoscopy, when a perforated colon caused abdominal sepsis and Type II Respiratory Failure. Having survived, the patient has since been suffering from bronchiectasis chronically colonised with *Pseudomonas aeruginosa* and *Staphylococcus aureus* and uses non-invasive-positive-pressure-ventilation at night and has access to it when required during the day. These pulmonary conditions require him to be upright in his chair for as many hours as possible daily and impedes full bedrest. The patient, in addition, suffers from a list of other comorbidities, of which a few examples are chronic hypernatremia, severe anxiety, and autonomic dysreflexia (AD).

The wound was never brought to stable closure and over a period of 4.5 years, it very slowly but steadily deteriorated. Whereas it never exceeded approximately 1 cm diameter (pic 1), the level of exudate would very slowly increase; until 4½ years after its creation, 8 months prior to MPPT, the whole area got out of control. At that time, the AD also started to increase dramatically in frequency as well as severity. Usually, the patient had had 2 or 3 episodes of AD weekly which could be dealt with by timely administration of 5mg Nifedipine. The frequency now increased and at the time MPPT was started, the patient suffered 45-minute episodes of AD 3 times daily with life-threatening blood pressure levels, strong spasms, shivering, and breathing difficulties - uncontrollable with the maximum recommended dose of Nifedipine. Presumably, the cause of the AD was the ulcer, as it would always occur at dressing changes as well as if anyone touched the wound or surrounding skin or there was a fold in the clothing in the vicinity of the ulcer.

4 months after the breakdown, i.e. 4 months before MPPT, the wound was surgically debrided, but to no avail. NPWT (vacuum pump) was not an option as the amount of slough was too high. Following the breakdown and both before and after the surgical debridement, the wound was treated with an array of different wound products, including: Prontosan solution, 8 months, i.e. from breakdown to start of MPPT; Prontosan gel, 2-3 months in total; Aquacel Ag, 3 months in total; Intrasite gel, 2 – 3 months in total; Purilon gel, 1 month in total; Promogran, 10 days; Promogran Prisma Ag. The deterioration of the wound and adjacent skin with widespread, severe cellulitis seemed unstoppable. Despite 3 times daily dressing changes of the superabsorbent packing plus two layers of superabsorbent pads plus an extra cover of superabsorbent foam, the dressing still leaked and fell off (pic 2 & 3 & 4).

At the time MPPT treatment was started 4 months after surgical debridement and 8 months after severe breakdown, the 7 cm long and 3 cm broad wound carved 5 cm deep and the bottom could not be identified due to abundant viscous slough filling out the winding gorges, (pic 3 & 4). There was a wide band of stiff, very touch-sensitive, purple-red cellulitis of a stiff, unbendable consistency similar to cardboard stretching approximately 10 cm down the leg, 15 cm into the scrotum and at least 5 cm up the back. The wound edges were elevated due to swelling and the swelling stretched into the adjacent skin. At the same time MPPT was started, an MRI, revealed that the 7 x 3 cm ulcer was directly on top of severe chronic osteomyelitis stretching from the right inferior pubic ramus and well beyond the ischial tuberosity. The sore reached far beyond the bony structures into the abdominopelvic cavity, involving also the area very close to the posterior wall of the rectum and extending a tract towards the natal cleft. Also, abscesses were present of which one of 1 cm diameter was found just below the coccyx. Both sides of the pelvis were suffering extensive and chronic myositis.

This slow but steady deterioration of an ulcer refusing to fully close and very gradually increasing the level of exudate, is not an atypical history of infection taking hold in the bone of immunosuppressed individuals, e.g. SCI. The sudden, irreversible and uncontrollable breakdown, experienced in this case 8 months

earlier, is not atypical either, but presumably happens when the amount of infectious, toxic debris generated in the bone, as well as in the adjacent contiguously infected structures, surpasses a certain threshold at which the local soft-tissue immune response is able to cope. By the time the soft tissue completely breaks down, the osteomyelitis will, presumably, have been present for a period of time already.

Due to the patient's many comorbidities and the extent and severity of the osteomyelitis and associated infections, surgical removal of the osteomyelitis was ruled out leaving only palliative care as an option. The patient was therefore prescribed antibiotics to slow down the unavoidable spread of the osteomyelitis and associated risk of sepsis as much as possible. The osteomyelitis was, however, already severe and, as it could not be reversed, it would continue to be associated with large amounts of infectious, corrosive exudate that needed to drain through the soft tissue from the bone to the surface, i.e. the wound. Soft tissue infection presents a very genuine risk of sepsis and cannot be treated with antibiotics. The aim of the MPPT treatment was therefore to remove the very widely disseminated pelvic soft tissue infection, including the cellulitis and myositis as well as to reduce the wound to serve as a controlled draining fistula and to overall maintain the soft tissue in the pelvis with the minimal infection possible for the longest time possible. This would reduce the risk of sepsis considerably. It was also likely that it would reduce the volume of the wound.

A reduction in AD blood pressure was noticeable within 5 days (pic 5) and, over the following weeks, the blood pressure became controllable with gradually lower doses of Nifedipine. This was presumably due to the removal of non-viable tissue and slough which serves as breeding ground for microbes (pic 6) as well as reduction in the extremely high volume of bacterial toxins disseminated in the entire area. Pic 7 shows an example of the large amounts of red-pigmented toxins that were repeatedly collected in the soft tissue; transported through the interstitial space; and expelled through the wound. The speed at which it happens demonstrates that it must be regarded as a controlled and targeted process (pic 7 & 8). Presumably, these toxins were also collected and expelled after penetration of abscesses disseminated in the soft tissue. Pic 6 shows an example of red-pigmented toxins being expelled through the skin, presumably from the evacuation of a small soft tissue abscess.

The cellulitis was brought under control and would steadily reduce over the following few months (pic 4 & 5 & 8 & 9 & 13 & 18). The touch-induced spasms would reduce at a similar pace as they were practically gone after 1 months.

By the time the osteomyelitis treatment with antibiotics was started, 6 weeks after start of MPPT, the cellulitis was gone; the slough had been cleaned out of all gorges and crevices; the wound bed was granulating; and the volume of the wound had reduced by an estimated 80% (pic 9).

This progress will, obviously, stagnate with the start of antibiotics. However, the overall palliative aim was to reduce the overall risk of sepsis which can readily originate in the bone as well as in the soft tissue. Administering systemic antibiotics, despite it causing harm to the soft tissue, was an attempt to reduce the speed of spread of the primary infection in the bone. MPPT was used to keep the soft tissue as healthy and free of infection as possible, despite MPPT working better in soft tissue without concomitant antibiotics.

MPPT can perform in parallel with systemic antibiotics being administered for a different condition, e.g. osteomyelitis or UTI. It is not ideal, because MPPT is designed to balance the natural microbiome, and antibiotics strongly alter this. The composition of the individual's microbiome and the MoA of the chosen antibiotic will influence the level of impact of the antibiotic. Mostly, the greatest set-back in the soft tissue (observed via the wound) is seen when the person starts and stops taking antibiotics as this coincides with the greatest sudden impact on the composition - and consequently balance - of the microbiome. Therefore, the fewer changes, the better stability and regeneration (granulation) in the soft tissue. Fewer changes also lower the risk of antimicrobial resistance, e.g. in the bone or in the urinary tract. MPPT will remove infection by antimicrobial resistant strains in the soft tissue.

The osteomyelitis treatment with antibiotics was started 6 weeks after the MRI was performed and the MPPT treatment was started (pic 9). It was 4 weeks Doxycycline (tetracycline) which was well tolerated by the patient, followed by 2 weeks of Co-trimoxazole (Trimethoprim/sulfamethoxazole) which was also well

tolerated. This was then changed back to Doxycycline which caused a severe increase in the AD blood pressure and after 3 days urgently changed back to 15 days of Co-trimoxazole which impacted the AD to a lesser degree. At this time, the patient was also started on systemic antifungal medicine against thrush in a different part of the body. A culture of a wound swab meant to identify the infective agent in the bone, showed that the presumed infective agent was resistant to Co-trimoxazole and the patient was switched back on Doxycycline with the result that the AD soared and the patient overall got extremely unwell and, after 3 days, was forced to stop taking it. After 5 days without antibiotics, Clindamycin was prescribed. The patient felt very unwell, with strong spasms, chest and neck pain and need for constant breathing assistance - but not as miserable as with the Doxycycline, However, after taking the Clindamycin for a few days, the patient had developed a severe urinary tract infection and colitis with diarrhoea and displayed blood infection markers (CRP) of 100+ mg/L (normal range below 10-20). After taking the Clindamycin for 5 days, he was changed to Trimethoprim, which was better tolerated in relation to AD but caused severe respiratory difficulties. This was stopped after 7 days and no more antibiotics were prescribed. In total the 4 different antibiotics and the antifungal were taken over a period of 81 days (pic 11).

Soft tissue infections cannot be treated with antibiotics. Instead, such infections can best be treated by establishing a well-balanced commensal microbiome. MPPT helps the immune system establish this. Every time an antibiotic is introduced or changed, the balance is overturned, and a new balance needs to be reached. Every change also introduces the risk of creating resistant strains which are typically linked to increased bacterial virulence, including in the bacterial species residing in and making up the microbiome of the soft tissue, and consequently worsening the state of the soft tissue. MPPT was able to keep the soft tissue stable despite these 10 abruptly induced changes in microbial balance performed during this approximately two and a half months period. The generated granulation tissue only reduced minimally during this time and the wound opening only enlarged minimally (pic 10 & 11).

From Day 124 onwards (pic 11), MPPT was continued as sole treatment. The easing of the strain was noticeable quickly (pic 12) and the wound continued to improve over the following 3.5 months (pic 13). During this period, the CRP also steadily reduced: 49 on Day 134 (pic 12);   48 on Day 141;   45 on Day 148;   40 on Day 155;   36 on Day 169;   32 on Day 189;   29 on Day 217;   24 on Day 227 (pic 13).

On Day 239 (pic 13), 3.5 months after stopping all antibiotics, based on a 3.5-month-old microbiology report, the patient was started on a combination of Rifadin (Rifampicin) and Fucidin (Fusidic acid). The AD blood pressure soared from its usual 100/70-80 to 170+/100 at least 3 times daily. The skin got very sensitive to even sitting up quietly and the liver function parameter, serum alkaline phosphatase, steadily increased. The CRP first rose to 36 but gradually decreased to 16 over the 1.5 months period (45 days). The patient was outright suffering from the bad tolerance of the antibiotics and, after 1.5 months, agreed with his consultants that antibiotics were not required and that antibiotics were only to be taken for episodes of noticeable deterioration in the osteomyelitis. (Osteomyelitis unavoidably expands continuously, but the clinical picture is usually characterised by “episodes” or “flare-ups” linked to stages of deterioration in the overlying wound and in patient wellbeing.) The wound tolerated these two antibiotics well (14).

From Day 284 MPPT was now again continued as sole treatment (pic 14). The liver function parameter rapidly returned to normal range and the CRP remained low: 14 on Day 293 (pic 15);   16 on Day 358;   12 on Day 400 (pic 17), at which point regular CRP tests were discontinued as routine. The patient continued MPPT as sole treatment.

On Day 389, nearly 13 months after MPPT treatment was started, an MRI was performed (pic 17). The MRI report interpreted that the osteomyelitis seemed to have undergone a change to the better. The patient had now been doing very well overall and been without antibiotics for 7.5 months (pic 14 - 18). The MRI report was delivered on Day 510, 1 year 5 months after start of MPPT (pic 18). Palliative MPPT care is continuing.

All pictures: Up: distal. Down: cranial. Left: lateral. Right: medial

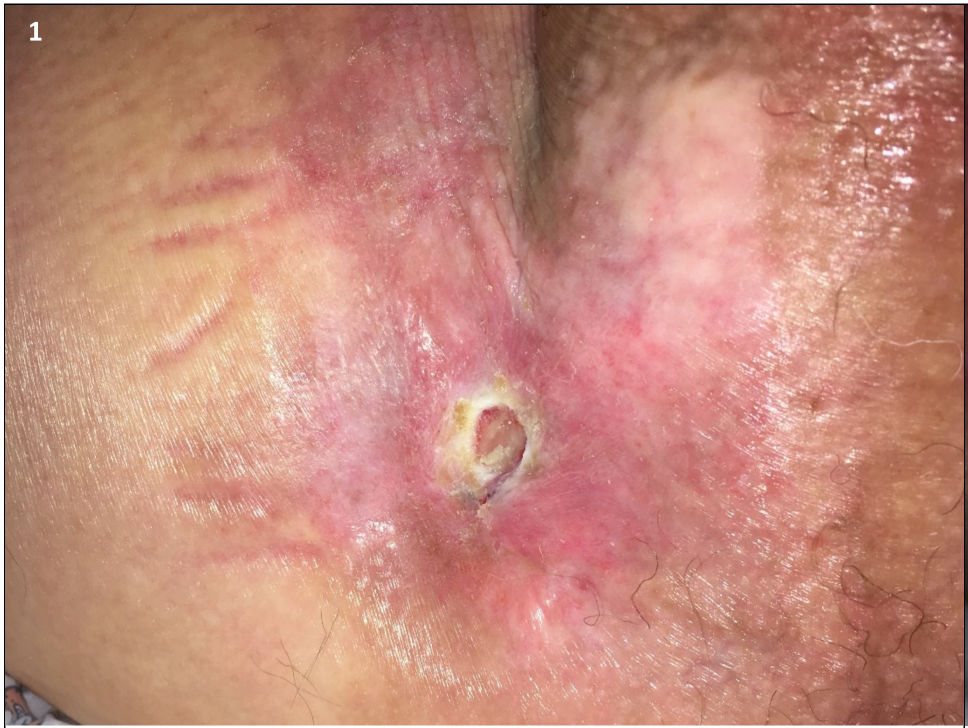	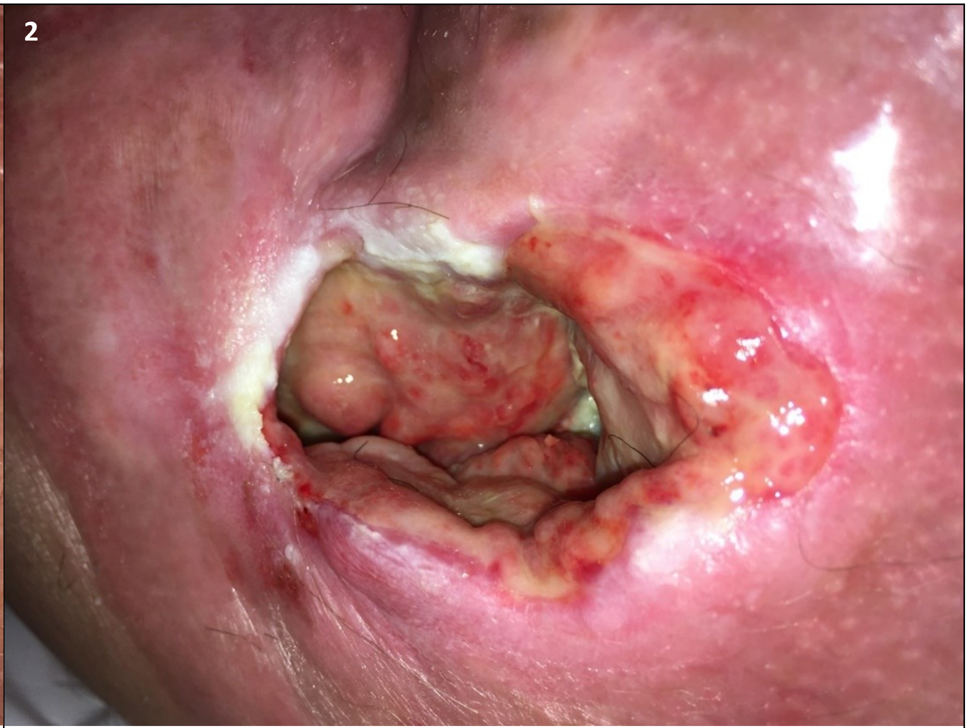
**Day minus-1015**<br>**2 years old ulcer, nearly 3 years before MPPT**	**Day minus-34**<br>**1 months before MPPT**<br>Strong tissue infultration and deeply embedded slough. Bottom is not reachable due to plug of tough slough. The speckled appearance shows infiltration (not granulation).<br>Cellulitis

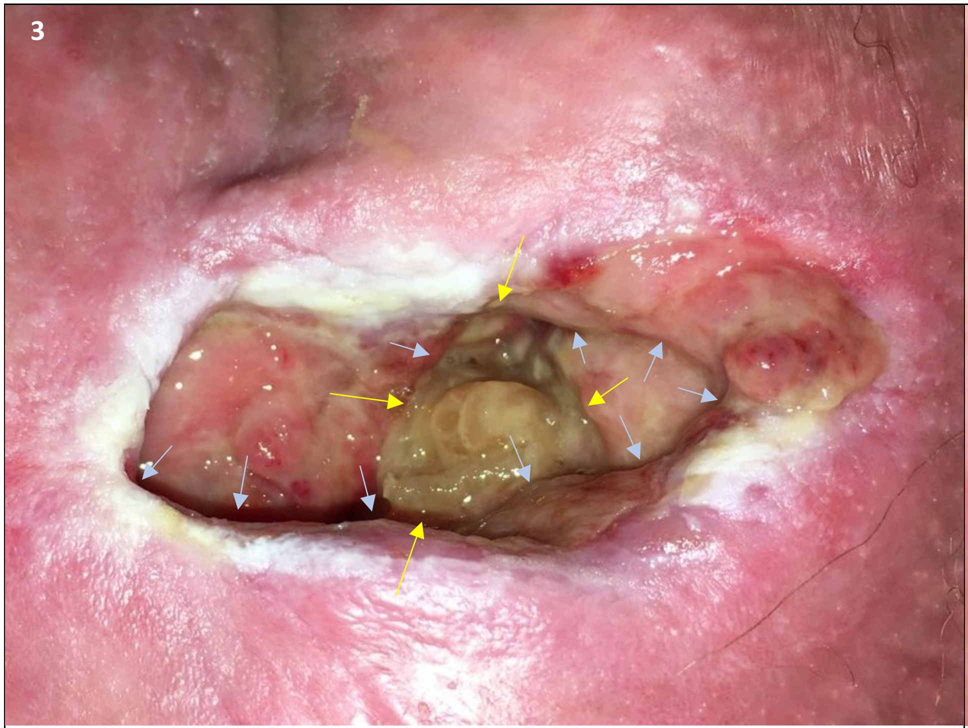

Day minus-9

1 week before start MPPT

Strong tissue infultration and deeply embedded slough. Abundant, tough non-removalble slough at the bottom. (The photo is taken after wound cleaning.)

Cellulitis

Light blue arrows: Wide gorge running across the entire bottom. Its trajectory corresponds to the underlying bone and it is draining the debris created by the infection in the bone.

*Yellow arrows: "*Hole" full of slough situated in the gorge. It leads into the abdominopelvic cavity.

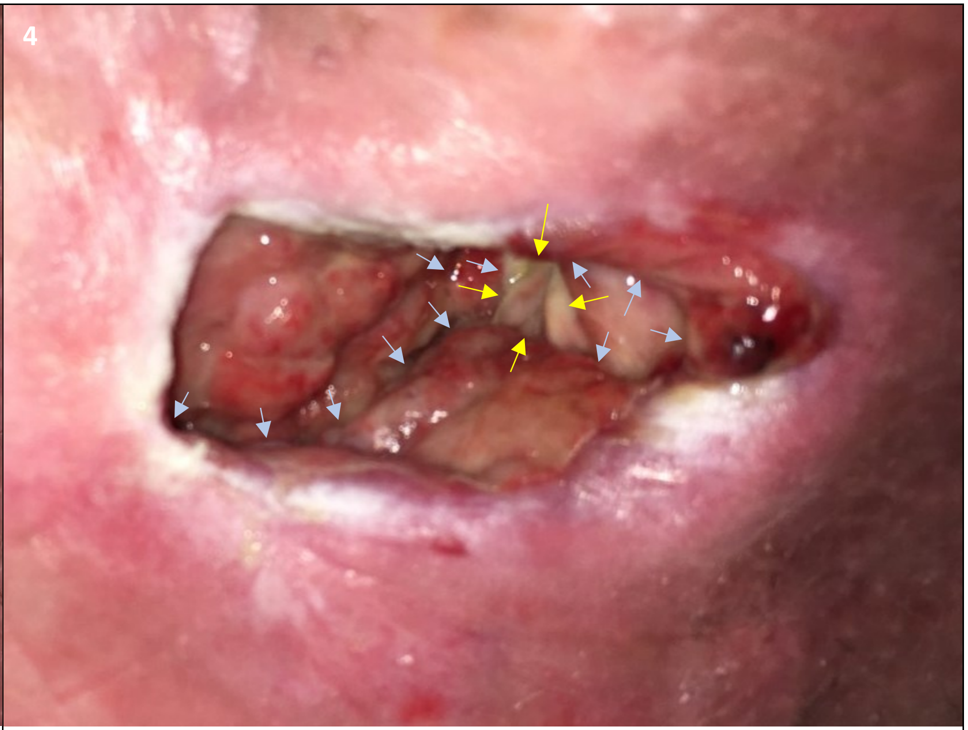

Day 0

Just before first MPPT

7 x 3 cm wound opening

Strong tissue infiltration and deeply embedded slough. A wound bottom cannot be identified or reached due to tough slough plugging the gorge and what seems to be a "hole" or severe widening of the gorge as well as the cavity behind it. This leads into the abdominopelvic cavity. The slough filling up the gorge is not visible from the angle the picture is taken.

Stiff, unbendable, purple-red cellulitis. Highly swollen and elevated wound edges. The cellulitis is affecting a 15 cm broad band of skin surrounding the wound opening.

Generalised swelling of all tissue.

Light blue arrows: Wide gorge running across the entire wound bed draining the debris created by the infection in the bone.

Yellow arrows: Hole situated in the gorge leading into abdominopelvic cavity.

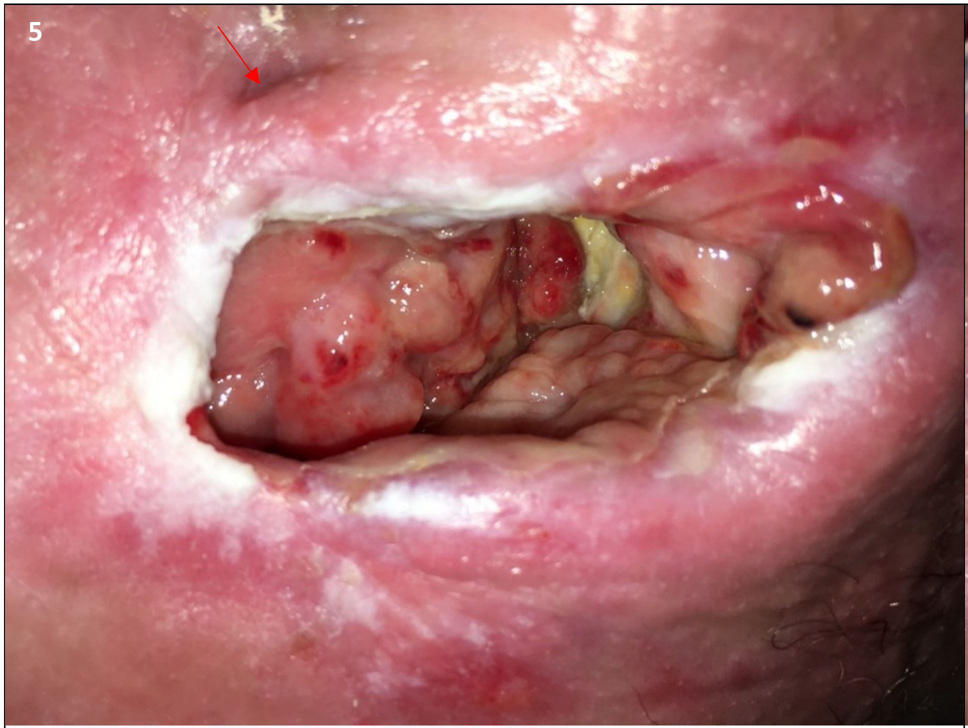

Day 5

Autolytic debridement is clearing out the slough in the gorge, allowing it to drain freely, and is in the process of clearing the "hole" as well.

Cellulitis remains. Edges are still elevated and highly swollen.

Red arrow: this shadow demonstrates the elevation of the wound edges.

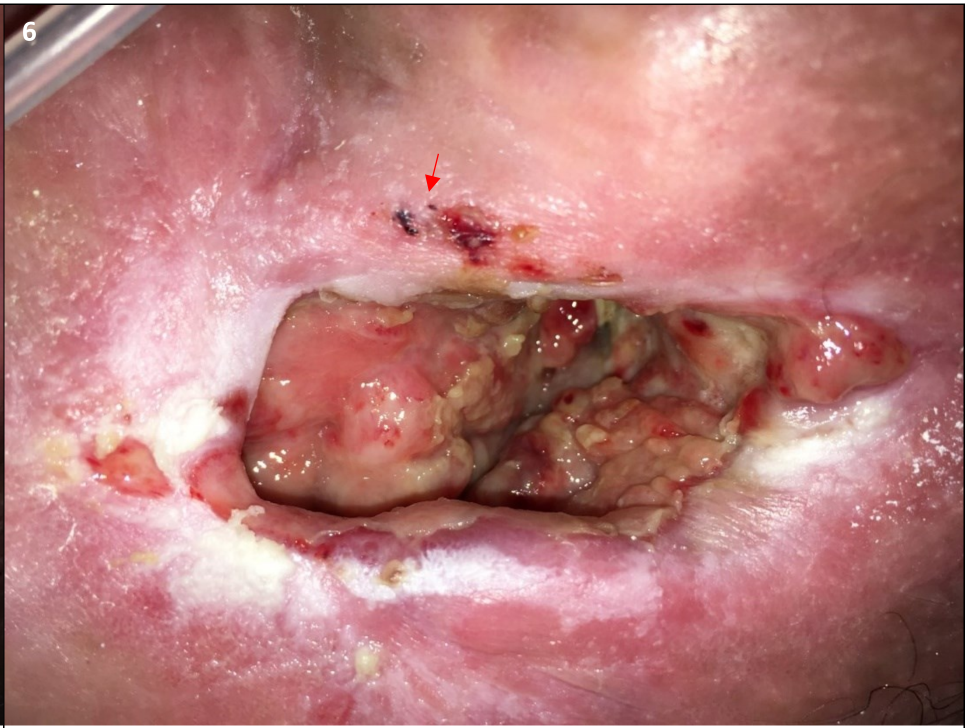

Day 11

The tough slough and the non-viable tissue is removed. The "hole" is now penetrable. Autolytic debridement is still ongoing in the infiltrated soft tissue, at the same time that granulation is evident.

The wound edges are still highly swollen along the lower and right-side rim. The upper and left-side rim has changed and is considerably less swollen.

Red arrow: The dark red spots in the wound edge are red toxins being expelled through the skin – presumably from abscess evacuations in the soft tissue.

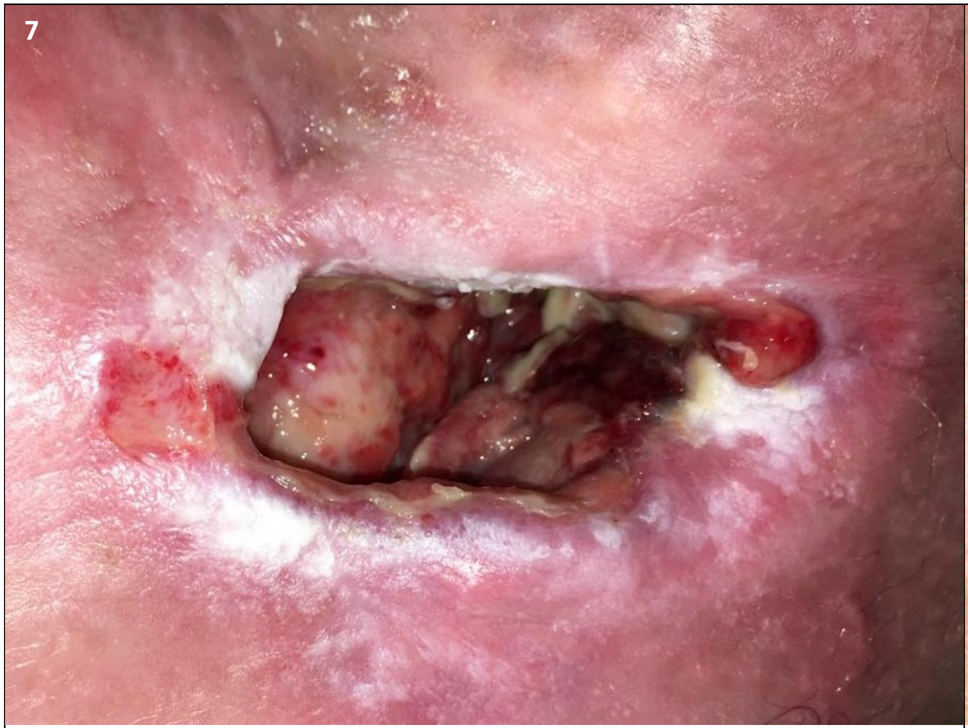

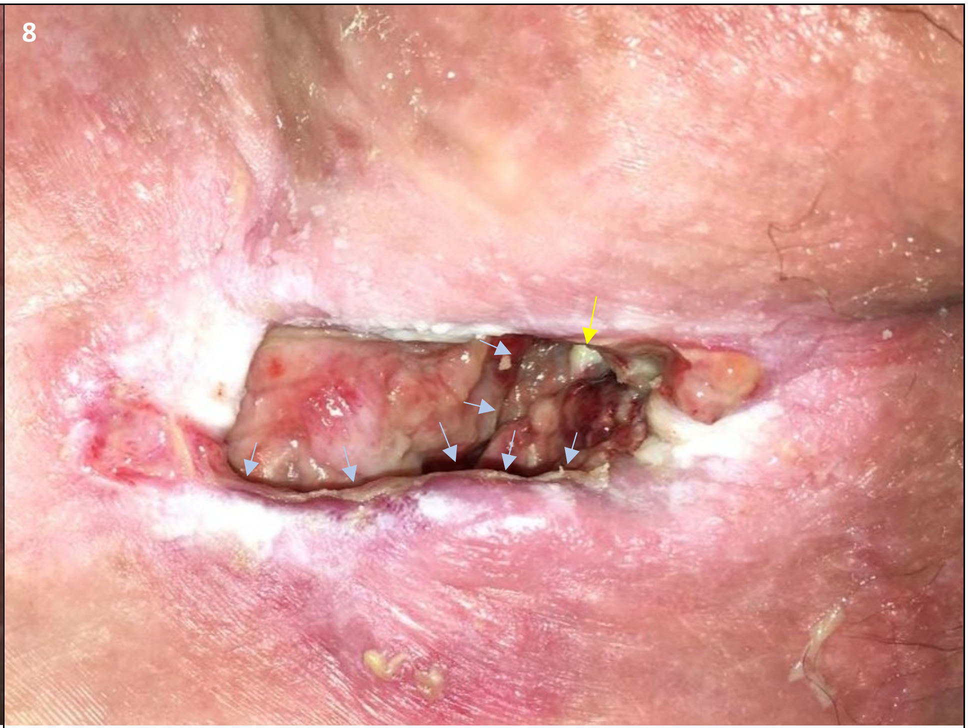

**Day 20 3 weeks**	**Day 24 3½ weeks**
Example of the large volumes of red-pigmented toxins, that have been trapped around the bone, in abscesses in the soft tissue, and disseminated and infiltrating far into the tissues in the vicinity of the primary infectious focus in the bone. It shows the red toxins as they are passing through the interstitial space of the granulation tissue. These toxins are in the process of being expelled through the wound, and will, during the coming hours, be further loosened, ready to be washed off in the next dressing change.<br><br>This phenomenon will continue throughout, as the harmful toxins themselves are associated with the bone infection and, to prevent them from causing or supporting further infection, e.g. in the soft tissue, the body needs to get rid of them. The amount expelled is, however, greater in the beginning of MPPT treatment as old "depots" are being emptied. They can much later in a long-term palliative care setting, increase again, reflecting the unavoidable expansion of the osteomyelitis.	The largest part of the red toxins have been expelled and the granulation is again easily distinguishable by its well defined formation. In the right side, lesser amounts of red toxins are still being expelled through the interstitial space of the new granulation tissue.<br><br>Natural coloured skin is now visible within the more immediate vicinity of the wound opening. The cellulitis has since the beginning been clearing from its periphery and inwards towards the wound opening. The peripheral borders were very far from the wound edges and therefore not within the pictures shown here.<br><br>The wound opening and volume are reducing in size.<br><br>The "hole" has been emptied and the space filled with granulation tissue. The "hole" has now been reduced to a gorge through which the debris from the primary infection in the bone can pass.<br><br>The wound edges are no longer elevated.

The wound edges are highly swollen and all edges are holding large amounts of red toxins (recognisable as purple colour under the skin) to be expelled over the coming days.	*Light blue arrows*: Clean gorge running across the entire bottom overlying the infected bone.<br><br>*Yellow arrow:* the ”hole”, which is gradually changing into a draining canal.

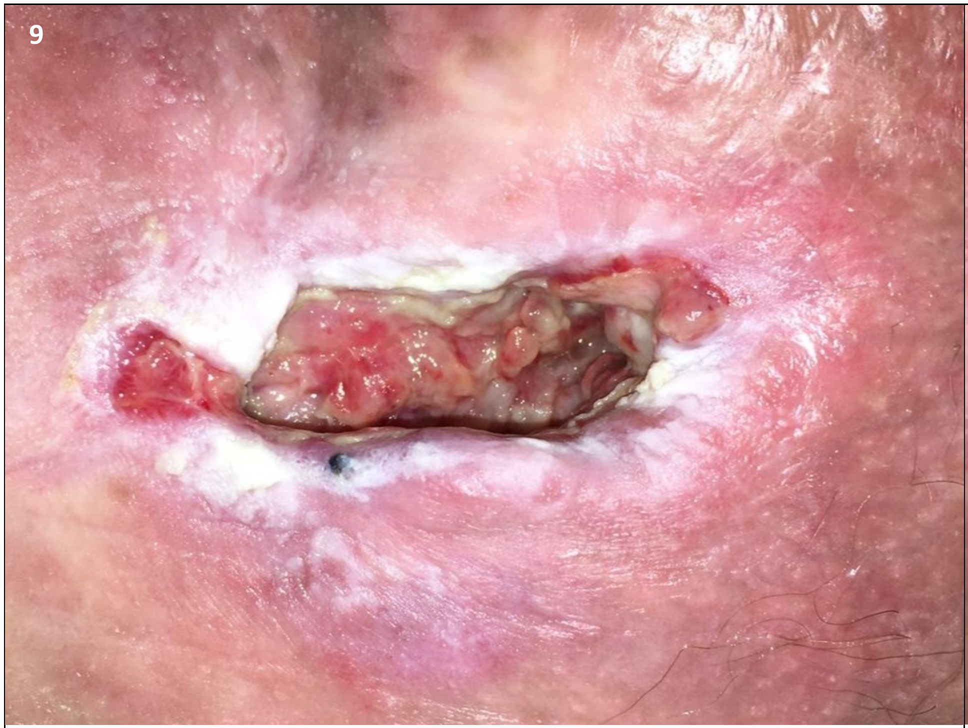

Day 43 1½ month

No antibiotics have been administered in the process, until this point.

4 x 1.5 cm wound opening (nearly half of original size). Volume considerably reduced.

The entire wound bed is granulating. All non-viable tissue has been removed.

The cellulitis is practically gone. The skin has changed from purple-red to skin-like colour, and the stiff card-board-like consistency has changed into the more natural soft and bendable skin structure. There is still some left just below the wound opening and the lower edge is still holding a more limited amount of red toxins to be expelled.

In the right side, red toxins continue to be expelled through the interstitial space of the new granulation tissue.

The froth and air bubbles recognisable in the slough removed daily and in the wound after washing, has been reducing - presumably an indication that the anaerobic microbial activity in the underlying area is reducing.

Systemic antibiotics will today be started to slow progress of the osteomyelitis.

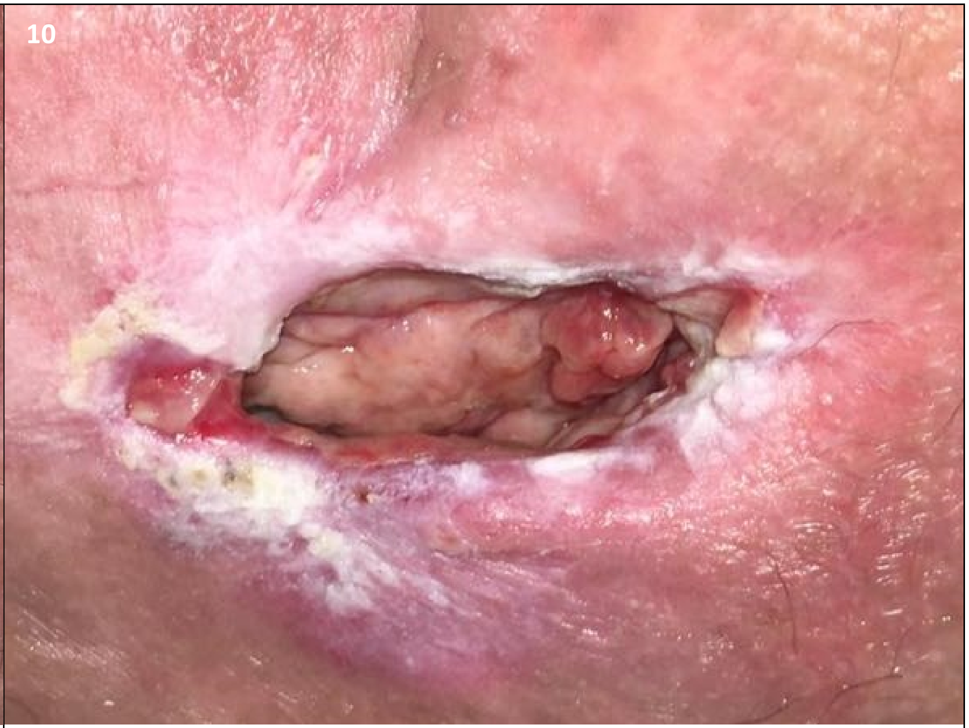

Day 100 3½ months

During different systemic antibiotics

4 cm x 2 cm wound opening.

The wound and cellulitis are holding up well. Both seem more "strained" than before the systemic antibiotics were introduced. The location of the former "hole" is filling up with a large piece of granulation tissue.

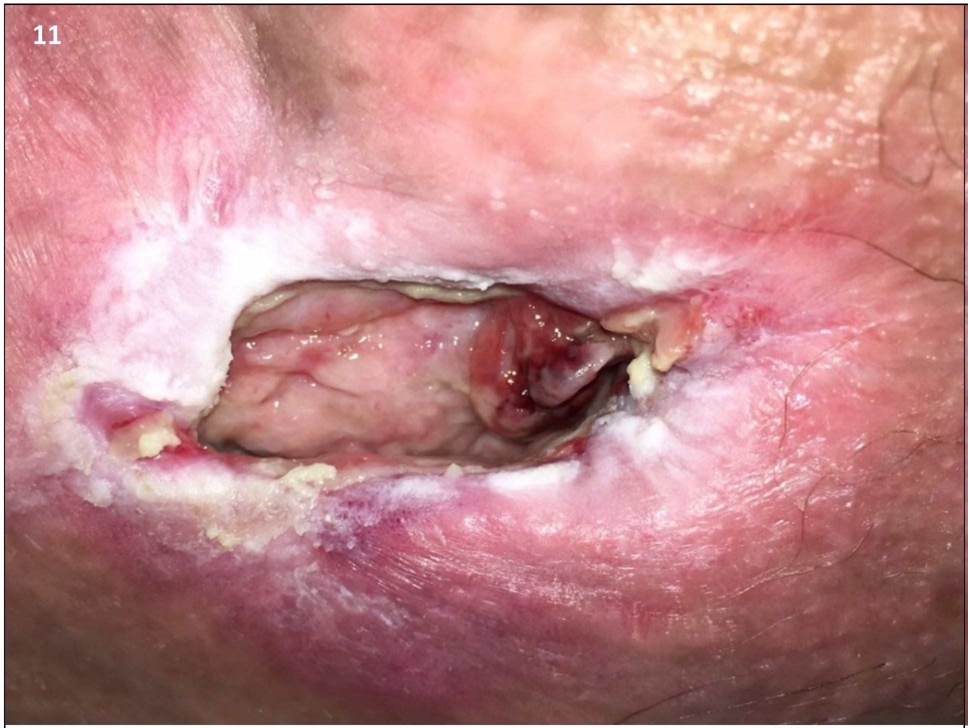

Day 124 4 months

After 2½ months of different systemic antibiotics and an antifungal with 10 changes in antimicrobial medication.

The volume of the wound is slightly increased.

The wound is evidently impacted by the simultaneous infections in the urinary tract and the intestine. The immune system down-prioritises wounds when the body faces several concomitant severe challenges (Crane et al. 2018, 2021).

The large piece of granulation in the right side is now holding large amounts of red toxins waiting to be expelled. The left side granulation has lost its easily distinguishable, well-defined formation and contains off-white pigmented toxins awaiting expulsion.

The lower edge has a strong purple-red collection of toxins to be expelled. Such expulsion serves to prevent the return of cellulitis.

Despite the wound being under strong pressure, it is holding up well.

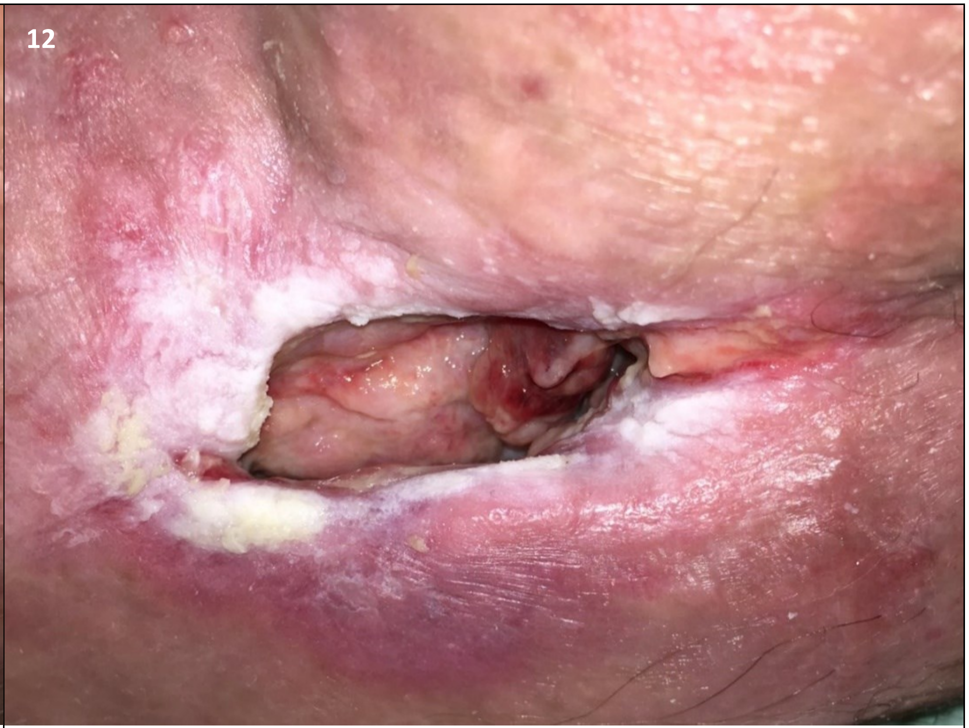

Day 138 4½ months

2 weeks after all antibiotics were stopped.

The wound and the skin are already less strained. Improvement all around.

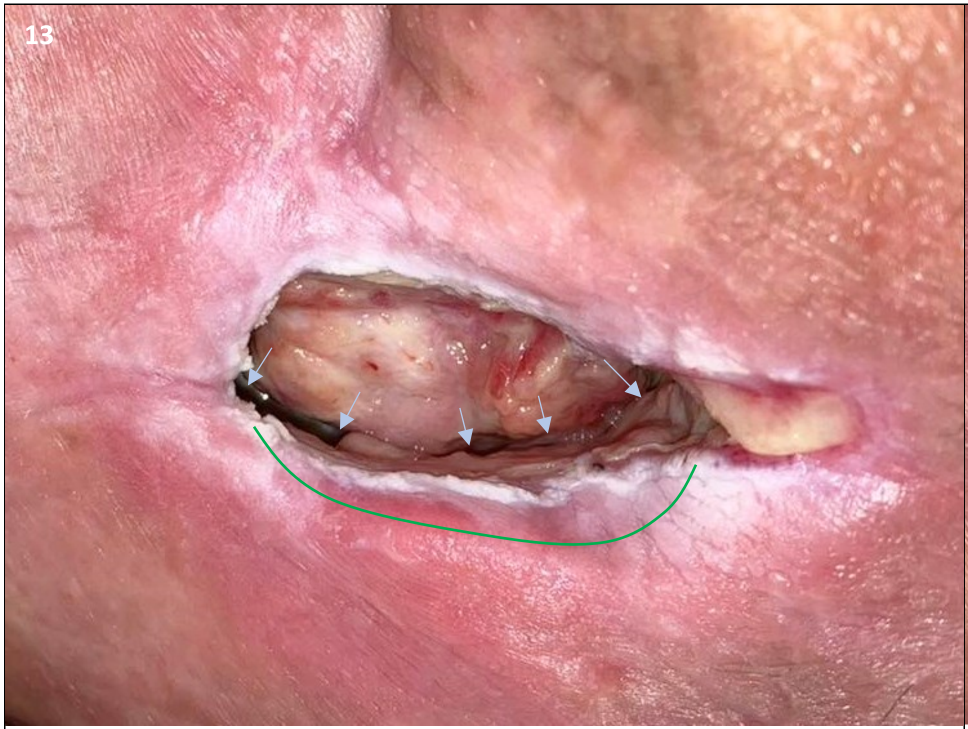

Day 239 7 ¾ months

After 115 days (3 ¾ months) without antibiotics

The gorges are empty, clean and granulating. The gorges have fused and are now one draining canal running through the length of the wound.

Large parts of the wound bed are granulating.

There are no signs of cellulitis. None of the edges or surrounding skin are swollen. The lower edge has fully epithelialized and the epithelium is moving to also line the inside of the draining canal as protection from the harmful debris from the osteomyelitis draining constantly through the gorge.

The wound is now (today) placed on a combination of 2 systemic antibiotics. (The osteomyelitis was not causing trouble and no reason was provided except vigilance for osteomyelitis.)

Light blue arrows: Clean, draining gorge across the wound. The rest of the soft tissue has fused and is granulating.

Green outline: Epithelium lining and protecting the inside of the wound which is serving as a draining fistula.

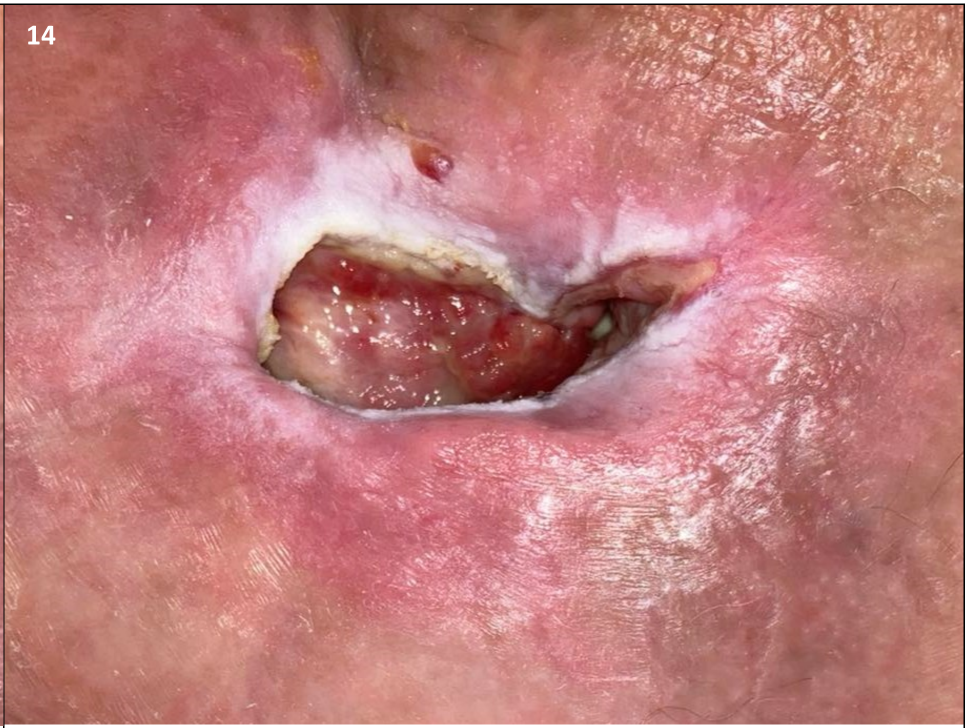

Day 283 9 ¼ months

After 1½ months of 2 systemic antibiotics in parallel – these are stopping now (today).

Wound continues to look well. No cellulitis. Inside lining is retained. Good granulation. A bit more embedded slough than usual. And a bit more generalised inflammation. But none of the adversities are easily detectable or deserving of special attention. The patient is feeling unwell, though.

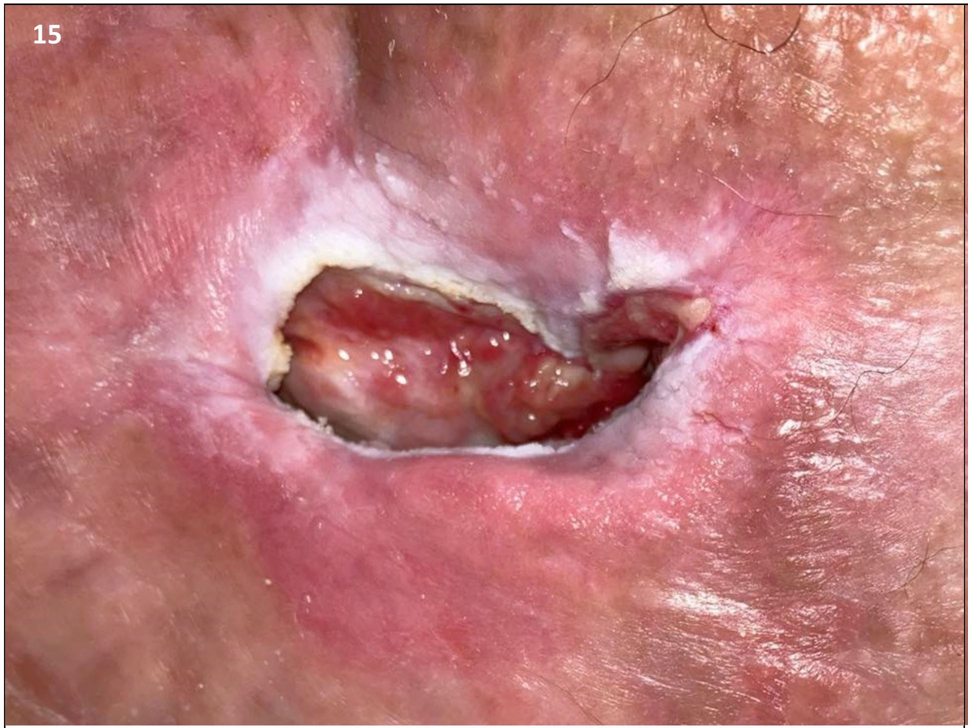	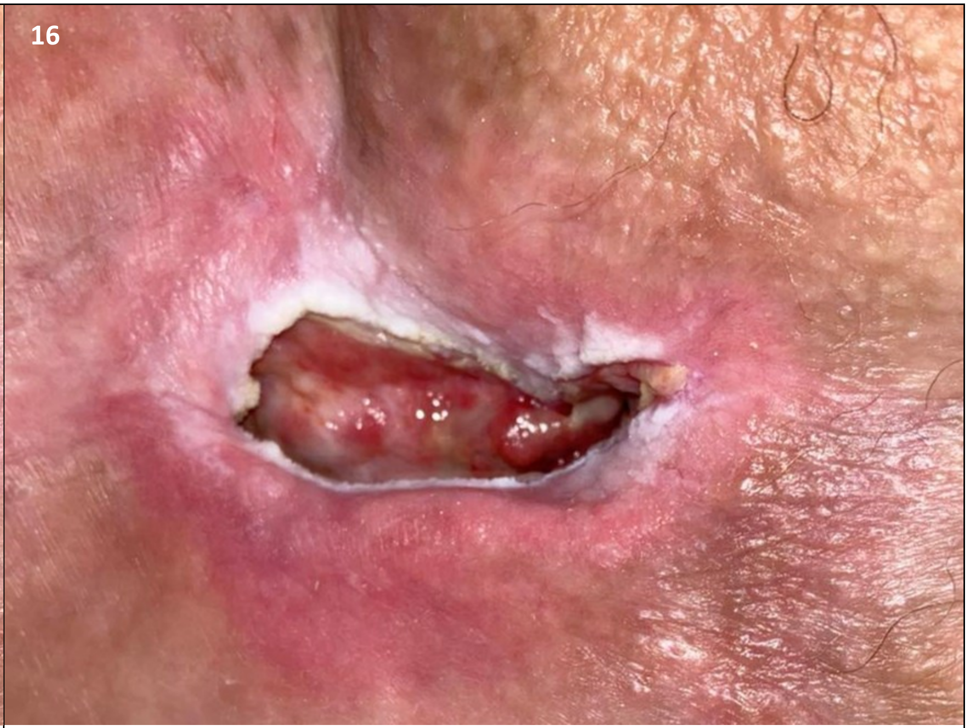
**Day 293 9 ½ months**<br>10 days after the antibiotics were stopped.<br>The slight strain on the wound is easing.<br>The embedded slough is loosening and the generalised slight inflammation in the surrounding skin is reducing. Good granulation in the wound bed.<br>Patient back to feeling well.	**Day 306 10 months**<br>23 days after the antibiotics were stopped.<br>Reduction in size and volume since the discontinuation of the antibiotics.<br>The slough retained withing the granulation is being loosened further and is being removed. The granulation is healthy, strong and without strain.

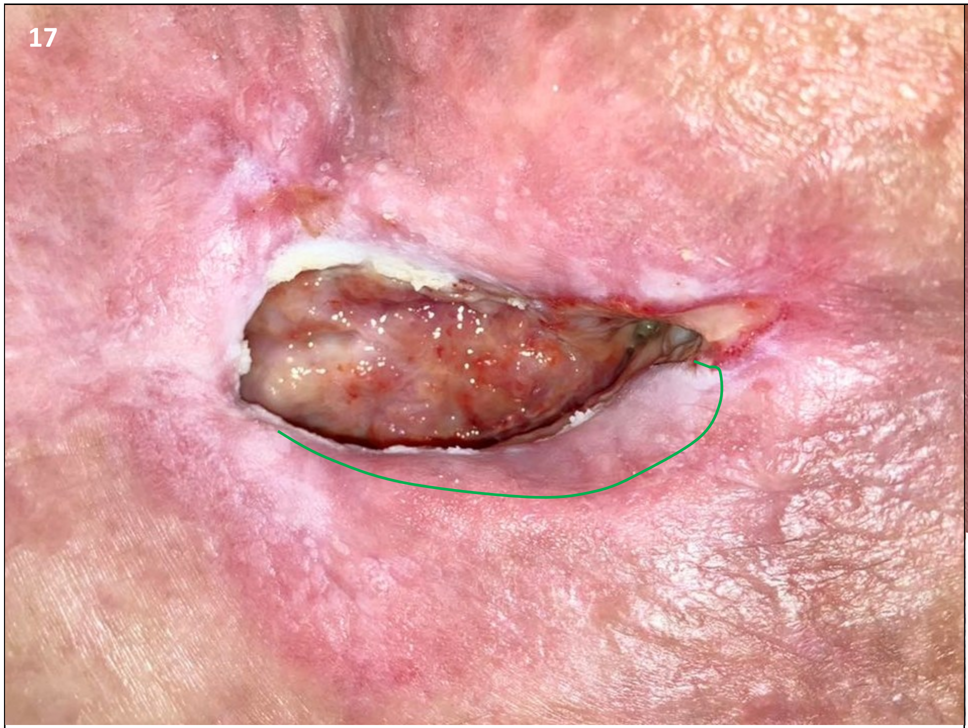

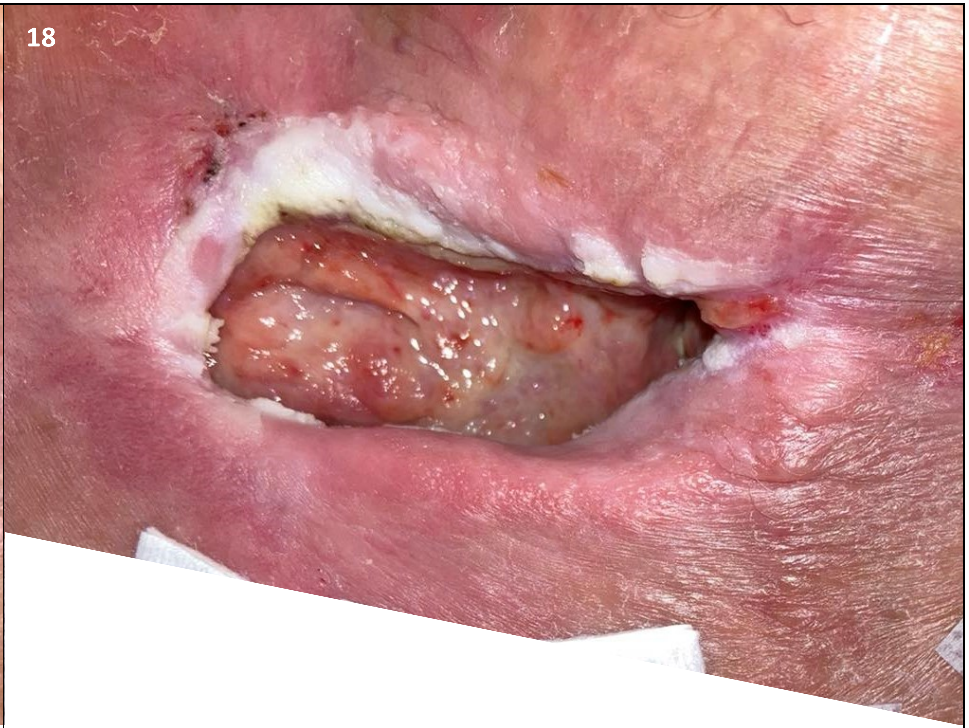

Day 396   1 year 1 month

112 days 3½ months without antibiotics

Wound is healthy and without infection. Good granulation keeps the wound volume small. No signs of cellulitis. The epithelium is moving in further on the lower edge protecting the inside. The same phenomenon is starting to show in the upper edge. All the gorges are clean and granulating and serving as draining canals.

The white matter on the skin surrounding the opening is maturing new epithelium. The corrosive debris continuously affects the skin which therefore continuously is undergoing repair.

Green outline: Epithelium lining and protecting the inside of the wound which is serving as a draining fistula.

Day 510   1 year 5 months

226 days, 7 ½ months, without antibiotics.

4 cm x 1.5 cm wound opening.

MPPT continuing as palliative treatment. No antibiotics.

Gorge clean. Wound bed clean and with granulation. No cellulitis. Patient well.

Wound number 34			Patient	SCI	
**Grade 4**	**8 months old**	**Ischial tuberosity**	**38-year-old male**	**3 years**	**T6 incomplete**

Part 1 – Pictures 1 – 20

Originally the wound presented as an infected abscess. This was surgically drained, leaving an open wound that was packed and occluded daily while the patient remained on full bedrest for a few months, after which plastic surgery was employed to achieve full closure. This procedure involved accessing the original abscess wound via a surgically created subcutaneous tunnel between the original wound and a surgical incision next to this. A haematoma developed. The original abscess wound as well as the secondary surgical incision site dehisced. Both wounds presented very high levels of exudate which were managed with negative pressure wound therapy (VAC). The wounds, however, stagnated and VAC was substituted by daily chlorhexidine washing and carboxymethylcellulose sodium (Aquacel) packing covered with an occlusive foam dressing. (Tielle Lite). The last 6 days before MPPT treatment was started, the washing with chlorhexidine had been substituted with saline (Normasol).

The day MPPT treatment was started, the opening of the abscess wound was 4 cm long, 1 cm wide, 2.5 cm deep. The skin surrounding the opening was scarred and remarkably nodular, which is usually a clear sign of infection lodged deep in the skin. The inside of the void was necrotic and sloughy and through the entire wound bed ran a distinct narrow but seemingly bottomless gorge. (pic 1) The bone was not palpable. A 4 cm long sinus tracked from inside the abscess medially towards the natal cleft and slightly distally towards the anus. The tip of the probe was readily palpable through the skin from the outside in the anal cleft next to the anus. The surgical incision site presented as a 10 x 3 mm, 10 mm deep fully epithelialized pit.

One or more distinct gorge(s) in the wound bed in a chronic wound is often an indication of a primary focus of infection hidden from view and originating in underlying non-soft tissue, such as in the bone or, as was later identified in this case, the intestine. When a trans-anal fistula of any type is present, material from the intestine will constantly pass into the adjacent sterile soft tissue where it will collect and cause and spread infection. The body will push as much of it as it can towards the body surface to get rid of it. This transport of intestinal infectious material mixed with debris from the soft-tissue infection, which together constitute the exudate, passes through extraordinarily narrow channels not readily identifiable by present day imaging techniques, particularly because these channels lack fixed locations. It tends to pass the interstitial space wherever it encounters the least resistance. This waste material will inevitably present as exudate in a wound located above serving as waste disposal point. Depending on the diameter of the trans-anal fistula, the amount of material can be very limited. It may pool in the interstitial space and only need to release a negligible amount of exudate onto the surface of the body every 2 or 3 days. The main debris may be gas and can therefore sometimes go unnoticed. Over time, a trans-anal fistula tends to expand its diameter and the draining channels tend to carve more fixed channels, draining fistulas, for passing the increased amount of material from the intestine to the exterior surface, i.e. the wound.

One week following the initiation of the MPPT treatment, the strong odour from the wound had disappeared and the level of exudate had reduced to a level that was manageable with a single cotton gauze pad. Two weeks after start of treatment, the patient no longer maintained continued bedrest.

As the wound cleaned out the slough and necrotic tissue and initiated granulation and epithelialisation processes, the gorge became clearer, and signs of gas released through the tissue with an emphysematous appearance as well as through the gorge, together with debris and fluid, were revealed (pic 2 & 3 & 4 & 5). The wound gradually regenerated new tissue, filling the void by granulating up from the bottom to skin level (pic 4 & 5 & 6), and the wound edges epithelialized, rapidly reducing the size of the wound opening (pics 2 & 4 & 5 & 6). Also, the infection in the skin was removed as manifested in the clearing of infection-nodules (pic 6). Accordingly, the wound showed all intentions of closing, except that the gorge remained visible in the now very small and still reducing wound bed, and a

canal materialised in the dermis in the furthest medial end (pic 6) - which was the direction towards the former surgical incision and surgically created tunnel. The canal seemed to lead into an extremely narrow tunnel, too slender to even consider probing.

Potentially there now seemed to be two tunnels taking shape, one deep into the muscle via the gorge presumably direction towards the anus as identified on the first day of MPPT but not spotted again, and a more superficial one seemingly in the dermis extending medially towards the incision pit. (pic 7 & 8). This continued to seem the case (pic 9) and was consistent with an MRI showing a remaining slender track towards the incision pit and bending into muscle. The MRI failed to identify the gorge and associated tracks that had consistently been seen leading debris and gas created by anaerobe activity, i.e. the physiological characteristics of an underlying primary focus of infection, into the wound bed, presumably because the tracks were too slender to pick up on imaging. The wound progressed and even closed for a few days (pic 10). However, it reopened in accordance with the physiological necessity of expelling debris and gas onto the surface of the body. It closed again (pic 11) but when the skin was stretched, an opening, through which gas and droplets would be able to escape, appeared. (pic 12).

Reopening only occurs when there is a primary source of infection unrelated to the soft tissue. Amicapsil is therefore used as an additional tool in diagnosing underlying primary infections, which is helpful because imaging in soft tissue still lacks the required resolution. No wound treated with Amicapsil has to date remained closed over a primary infection, thereby causing an abscess. In this case, it was clear that a primary infection was causing the wound, but unclear whether it was osteomyelitis, an anal fistula, or a different issue.

As the original wound as such was now healed and the area under control by the immune system, existing abscesses scattered in the soft tissue including many cm from the wound would be penetrated, presumably by the immune cells. The interior concentrations of red-pigmented toxins would be evacuated and transported by the immune system to the skin surface and disposed of on the body surface via the wound opening or, presumably depending on the distance from the wound, through temporary skin openings. These skin openings would close again within hours of finalised evacuation. The possible origin of such toxins is *Serratia marcescens* which forms part of the natural skin microbiota of SCI persons; it is multi-resistant to antibiotics; and it is associated with abscess formation in SCI (pic 13 & 14) (*S4: SCI, immune dysfunction, osteomyelitis and aim of MPPT)*

The wound remained negligent for many months. However, as a primary source of infection, like any medical condition, over time gradually becomes more severe, it produces slightly more debris that needs to drain onto the surface of the body. These, still extremely limited, amounts of debris continued to drain via the narrow track or fistula which remained well controlled and infection free allowing the patient to live an active life without bedrest (pic 15-20). This was the maintained situation when the patient, approximately a year after the initiation of the treatment with Amicapsil, went into surgery for a planned, not-wound-related issue.

Part 2 – Pictures 21 – 29

The performed surgery required several months of back-to-back systemic antibiotic courses. During this time, the wound was managed conventionally and grew from a small negligible opening in the skin serving as the track's point of exit into a deteriorating wound (pic 21). One week after discontinuation of systemic antibiotics and reinstatement of MPPT treatment, the wound opening had halved (pic. 22) and the inside had granulated up into a negligible non-infected cavity with a gorge (presumably acting as a track-exit) and the exit hole of the other canal, both readily visible upon inspection (pic. 23).

Over the following months, the wound continued to serve as the outlet for the very small but steady volume of infectious material originating in a primary infection in non-soft tissue and transported to the surface through these two tracks (pic 24). The skin regenerated enough to separate the two tracks at skin level displaying a clinical picture similar to a year before (pic 7 & 8 & 9 & 25). The wound was maintained in this steady and controlled state for several months until

the wound, for reasons unknown and not related to the state of the wound, was removed from MPPT treatment for a two-week period and daily washed with the antiseptic, Chlorhexidine, covered with calcium-alginate (Sorbsan) and occluded with a standard foam dressing. The nodulous infection in the skin returned and the exudate level increased considerably. The epithelium covering the surgical incision pit also broke and the pit started steadily exuding extremely limited volumes (pic 26). MPPT treatment was reinstated and sorted the immediate upset (pic 27).

The primary source of infection seemed to have changed permanently as the level of exudate draining through the wound remained higher than before the period of treatment with antiseptics, calcium alginate and occlusion. In addition, the surgical incision pit had also undergone a permanent change and remained active continuing to drain very low levels of exudate, as well (pic. 28).

An MRI at this point revealed the long-awaited primary source of infection. It showed a complex perianal sepsis with a connection to the anal canal, most likely in the form of a transphincteric fistula, with a 5.4 x 1.9 cm abscess in the ischioanal fossa and connection to the skin. It further showed a second set of tracks with a collection, presumably interconnected with the perianal abscess, also connected to the skin.

This was consistent with the two track openings observed throughout the past two years. The one medially in the wound bed, had been presumed to run between the wound and the old incision pit through the former tunnel, that had been made surgically to drain the abscess, and from there bend downwards into the tissue. The other one laterally in the wound bed which had shown itself in the form of a gorge practically all the time, was probed and palpated on the day of initiation of MPPT treatment, as it ran between the wound medially and slightly distally towards the anus with the probe palpable from the outside in the natal cleft not far from the anus.

The surgery was extensive because it had to remove the perianal abscess containing intestinal material; the consequential surrounding inflammatory changes; and the network of interconnected tracks between the perianal abscess and the surface, including the old, but draining, surgical incision pit.

However, flap surgery with extensive removal of soft tissue was not required because MPPT had managed to keep the soft tissue containing the network of interconnected tracks that drained the intestinal matter, free of infection for the past two years. (Pic. 29).

--------------- Part one ---------------

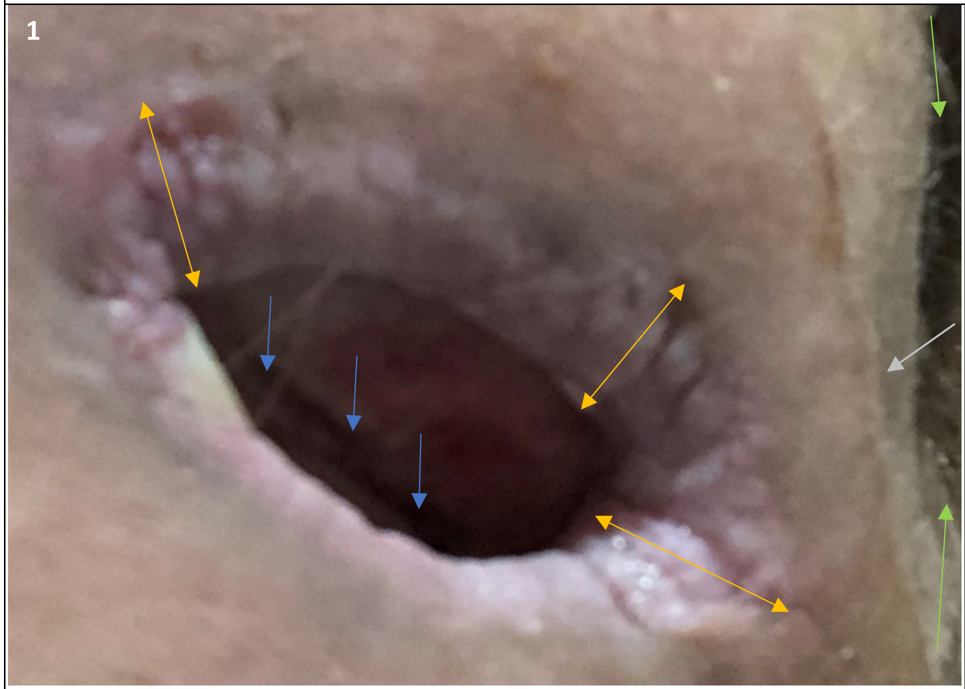

Day 0

Just before first MPPT application

40 x 10 mm opening, 25mm depth; 40 mm sinus tracking medially (right side)

Green arrow: natal cleft – medially

Grey arrow: surgical incision scar – pit 10x3mm; 10mm deep

Blue arrows: gorge

Yellow arrows: examples of “nodulous skin”

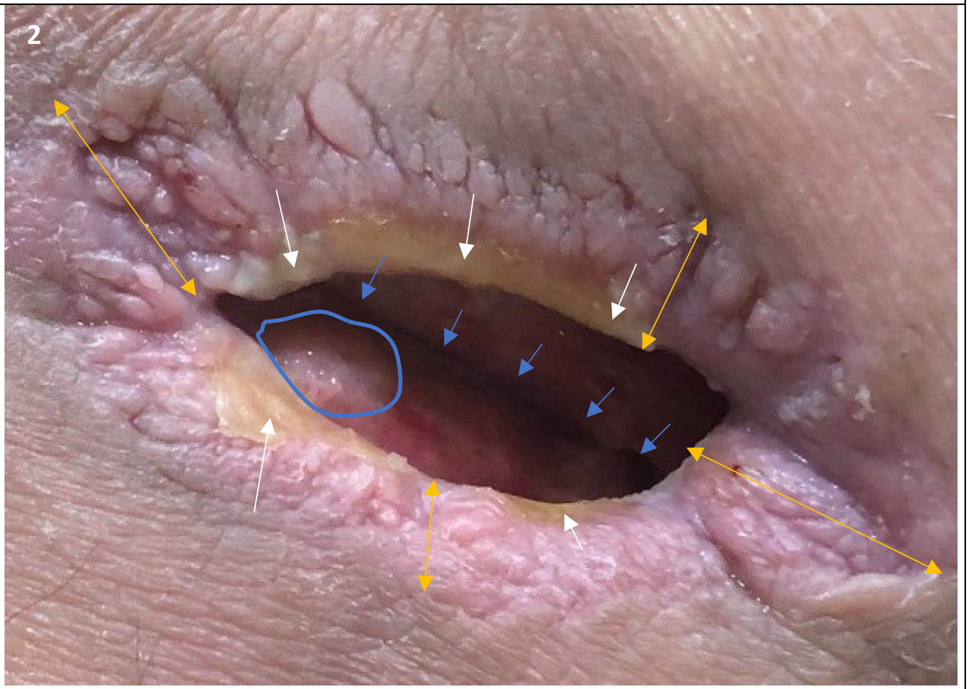

Day 3

Wound bed is granulating.

Blue circle: soft tissue emphysema- presumably releasing gas through the interstitial space. (It may prove easier to pass air through the tissue compared to the gorge which was full of viscous possibly impenetrable slough until 24-48 hours earlier.

Blue arrows: gorge.

White arrows: epithelializing wound edges covered in protective layer.

Yellow arrows: Extensive nodulation of a broad band of the skin surrounding the opening.

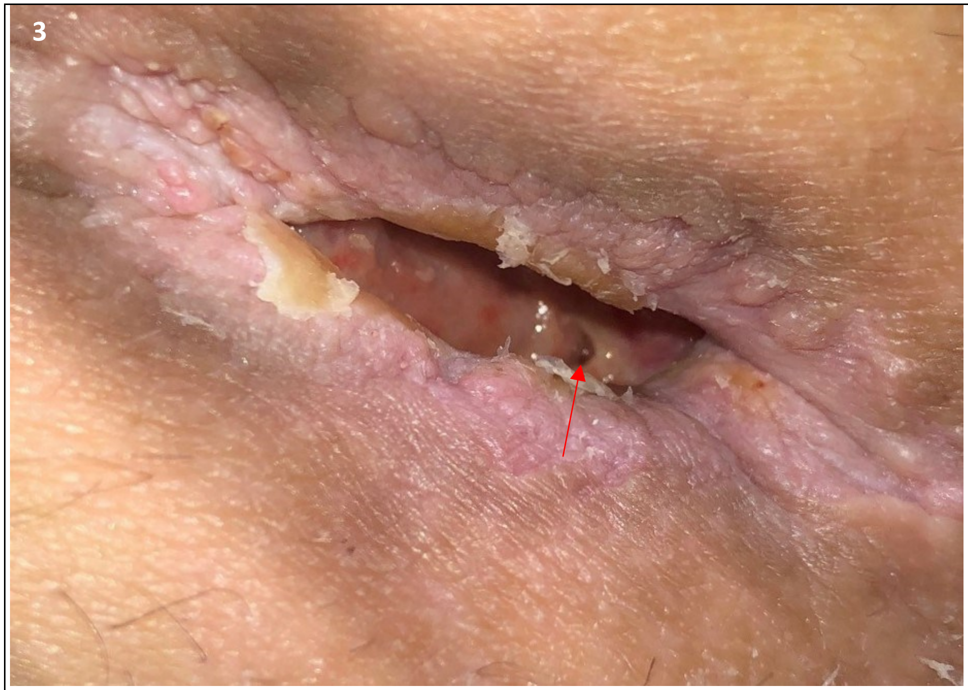

Day 5

Red arrow: The picture is taken shortly *after* showering the wound. Judging from the shape of this structure it was presumably created by one or more air-bubbles that later burst.

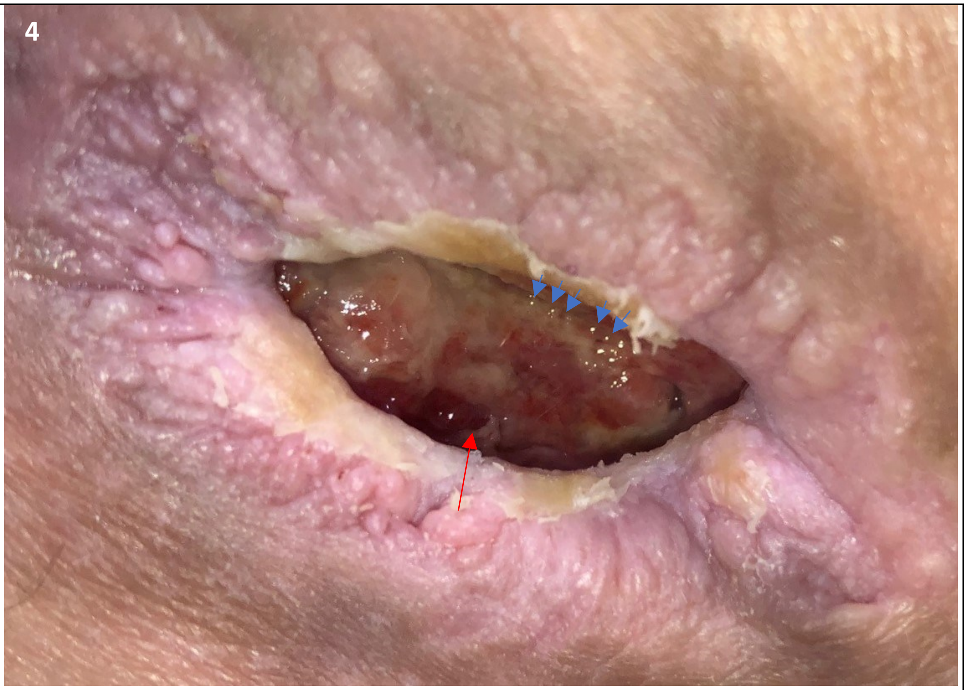

Day 8 1 week

Blue arrows: Pinhole size holes sitting in the gorge, presumably holes through which air is surfacing and escaping.

Red arrow: the air-created structure remains in the tissue, indicating air bubbles are passing regularly.

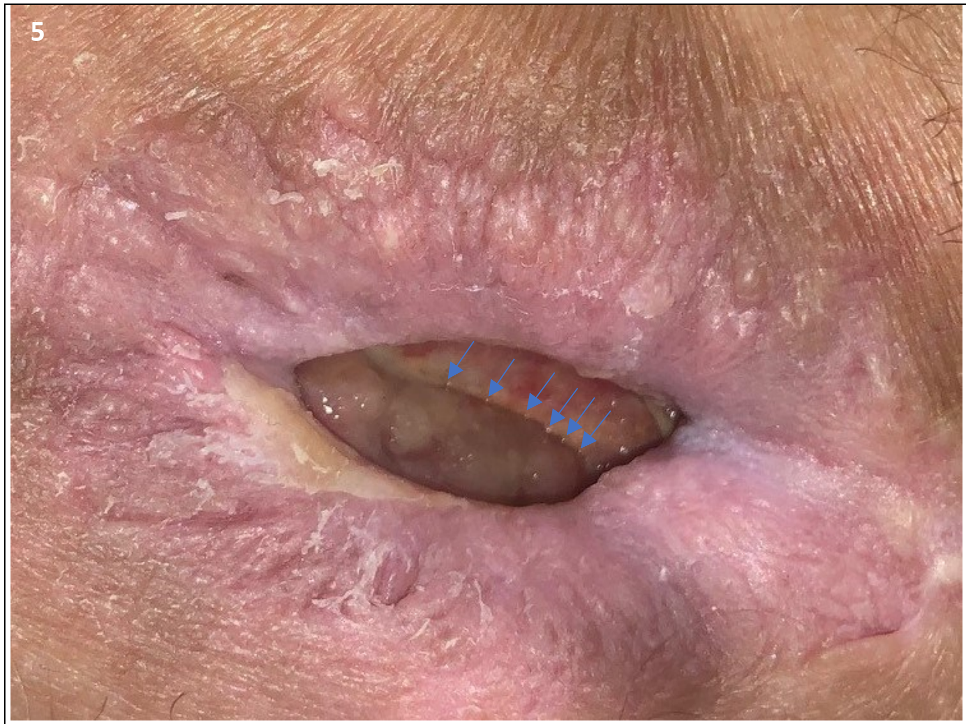

Day 33 1 month

Nodulation reducing in prominence turning into healthier scar tissue, instead.

Blue arrows: Pin-sized holes continue to sit in the persistent gorge of an otherwise well granulating wound bed.

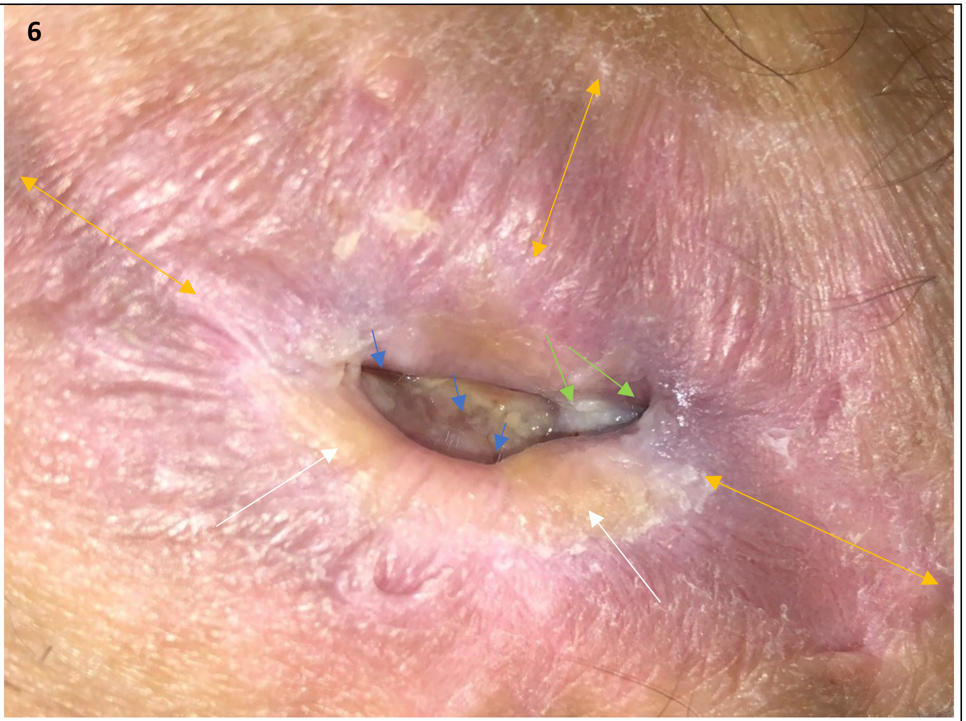

Day 57 2 months

Wound opening has reduced in size to 15 x 4 mm. The wound bed has granulated to the level of the skin. The opaque white-yellowish colour dominating the wound bed is a thick layer of germ cells (any yellow slough has been washed off.)

Nodulation of the skin has now cleared, indicating that the deep infiltration of the skin is gone. Also the new skin along the edges is smooth healthy looking new epithelium.

Blue arrows: The wound bed still holds a gorge.

Green arrows: A superficial canal in the dermis seems to be opening a canal medially.

White arrows: epithelializing wound edges covered in protective layer, similar to a moving scab.

Yellow arrows: Infective nodules practically cleared.

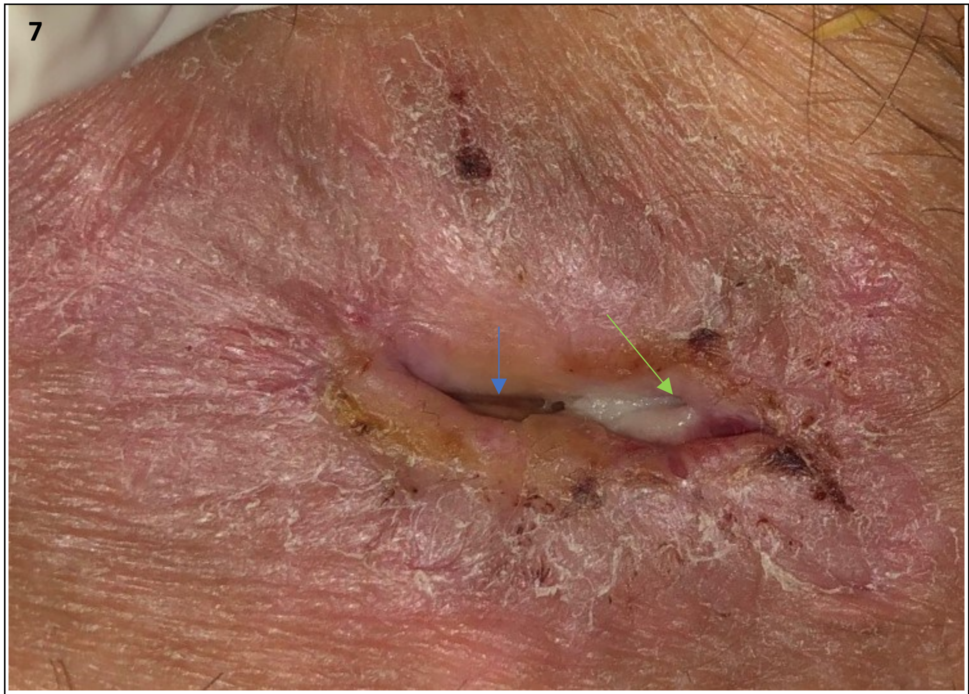<br><br>**Day 75 2.5 months**<br>The wound continues to reduce in size.<br>*Blue arrow*: The original wound bed remaining only as the original groove.<br>*Green arrow:* The superficial canal in the dermis opening medially towards the original surgical incision site.	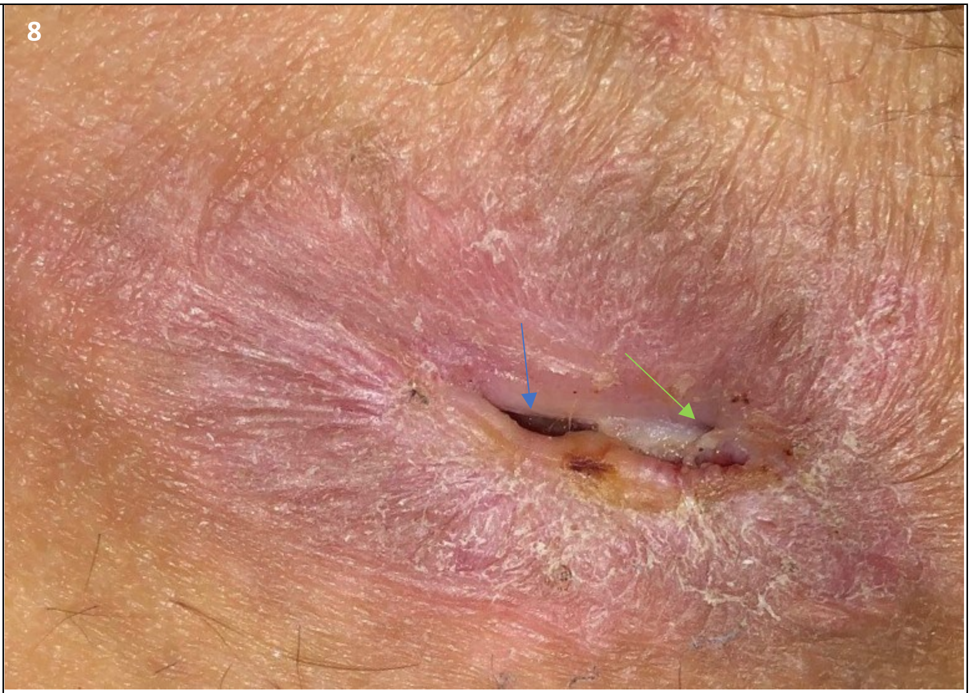<br><br>**Day 83 2.75 months**<br>*Blue arrow*: The original wound bed remaining only as the original groove.<br>*Green arrow:* The superficial canal in the dermis opening medially towards the original surgical incision site.

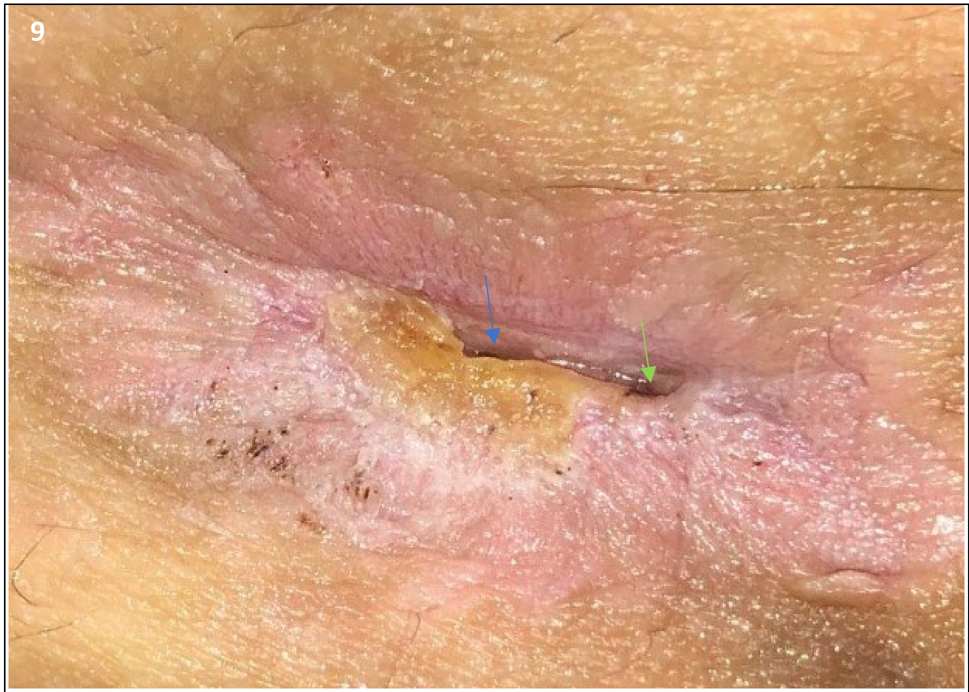

Day 102 3.5 months

Wound opening is now 15 x 1 mm. It practically consists of two different tracks, presumable a deep one acting as a draining fistula from the underlying primary source of infection; and a superficial one in the process of clearing out the infection along the tunnel created during surgery.

Blue arrow: The original wound bed remains only as the original groove.

Green arrow: The superficial canal in the dermis leading medially towards the original surgical incision site.

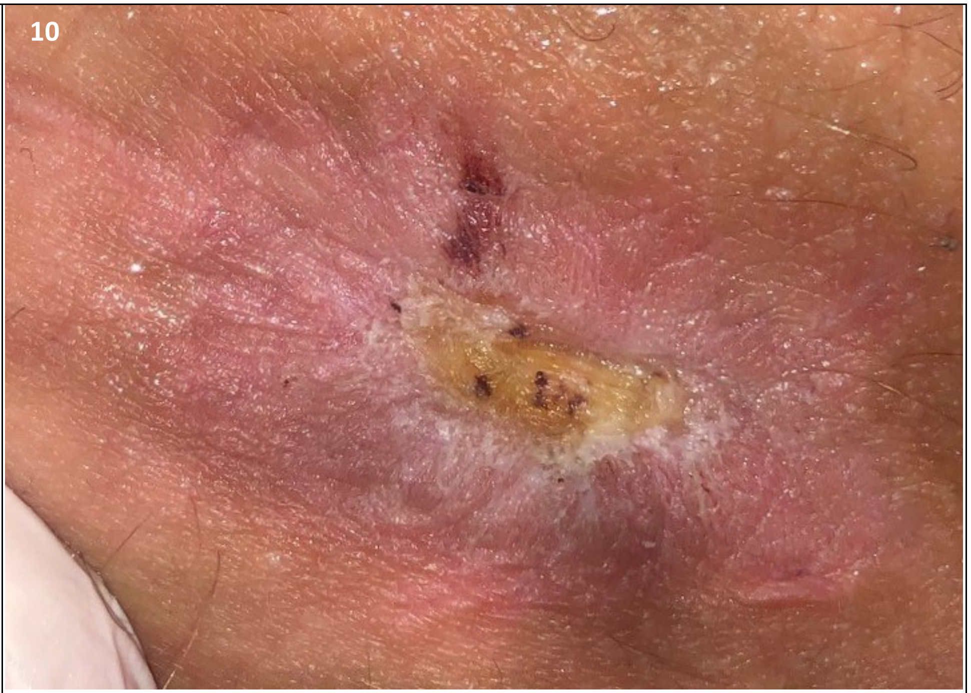

Day 110 3.5 months

Wound seemingly remained closed for two or three days, but reopened when it needed to deposit debris onto the surface of the body.

Reopening only occurs when there is a primary source of infection unrelated to the soft tissue. MPPT is therefore used in diagnosing underlying primary infections. No wound treated with MPPT has to date remained closed over a primary infection, thereby causing an abscess.

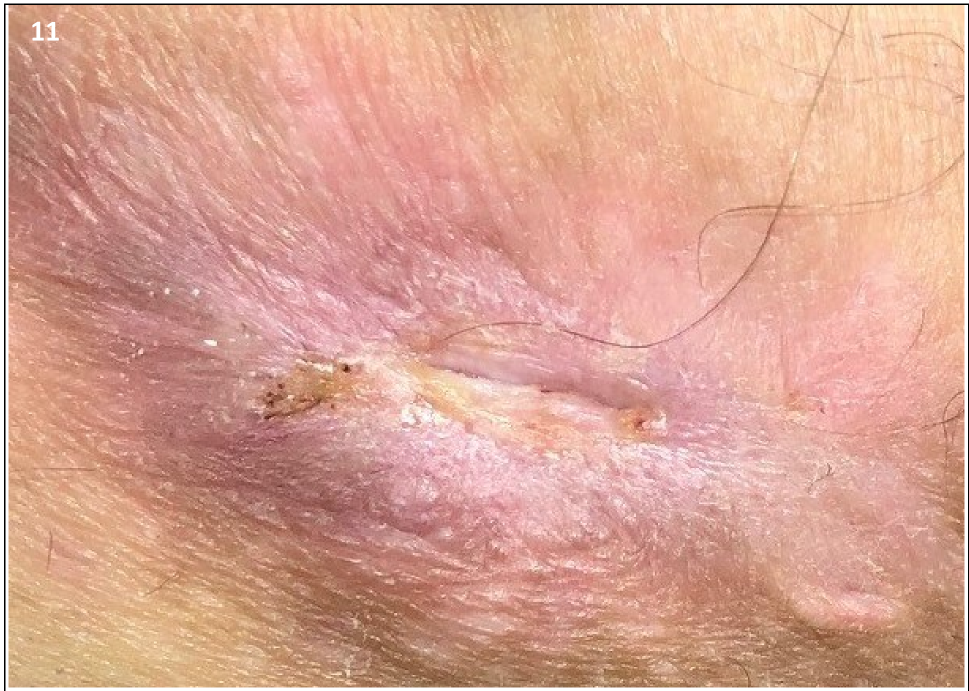

Day 132 4.5 months

Seems fully closed

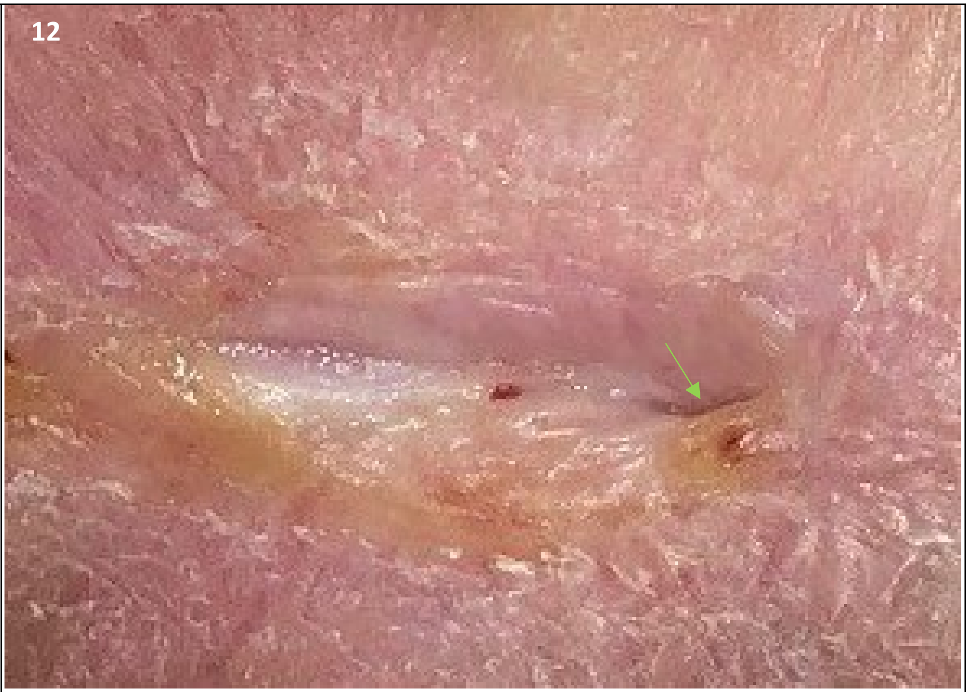

Day 132 4.5 months - Closeup

As the skin in the wound in the previous picture is being stretched and pulled, a 0.1 x 0.05 mm opening appears presumably allowing gas either directly from the anal canal or from anaerobic activity in the extra-anal tissue at the bottom of the track to escape**.**

Green arrow: The superficial canal in the dermis leading medially towards the original surgical incision site.

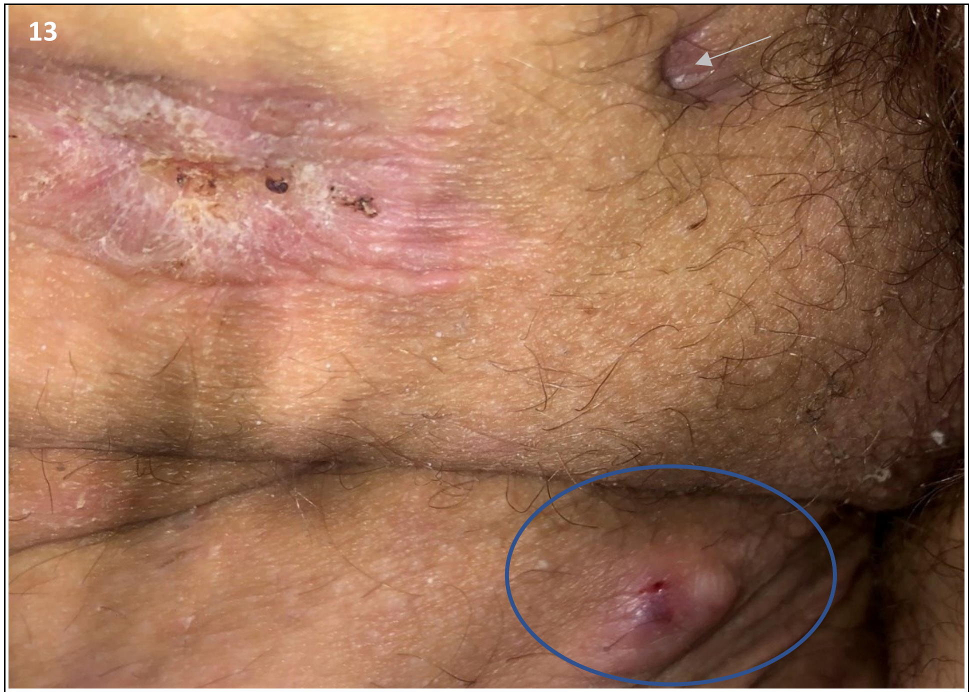

Day 162 5.5 months

As the original wound area is now under control, the immune system moves to further clear the area of disseminated abscesses.

Blue circle shows the location of the closeup in the next picture.

Grey arrow: surgical incision scar pit.

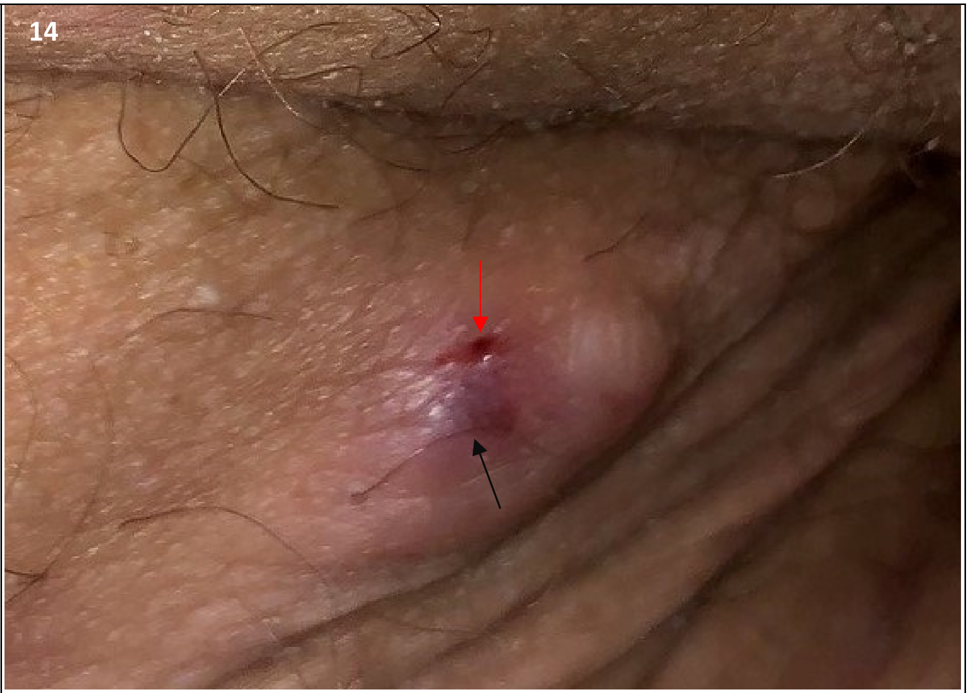

Day 162 5.5 months – Closeup

Excerpt from previous picture

14 cm distance from the original wound.

Example of toxins from an abscess in the process of being expelled.

Black arrow: Concentration of red coloured pigment waiting under the skin to be expelled. The masking by the skin makes it appear purple until exposed unprotected.

Red arrow: opening with bright-red pigment directly visible.

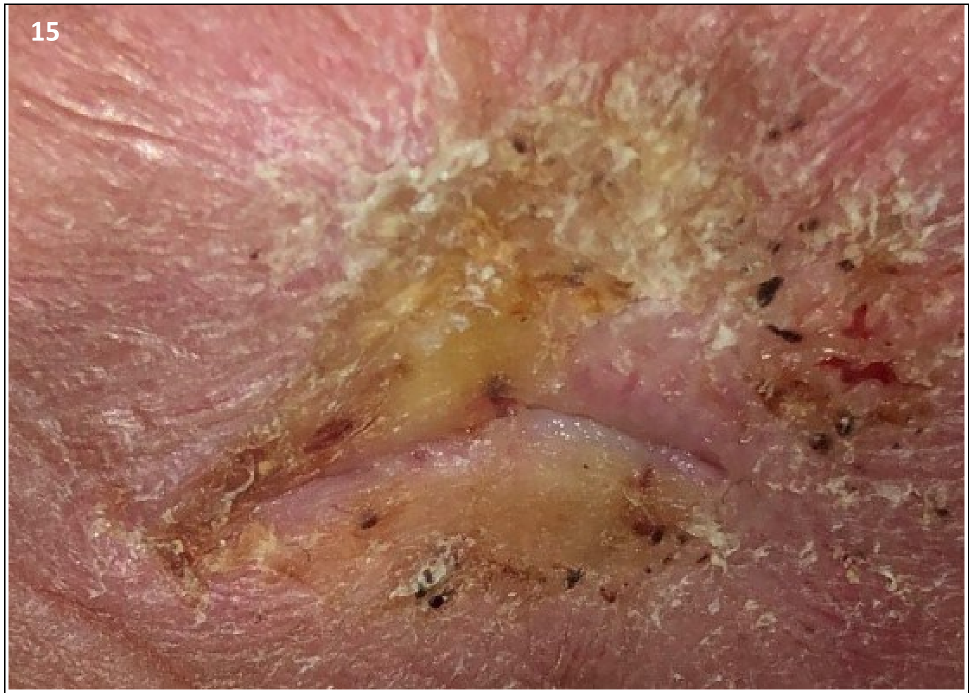

15

Day 212 7 months - Closeup

As the anal fistula becomes wider, more infectious material escapes into the extra-anal tissue needing to drain to the surface of the body through the track. This allows the wound to remain closed for ever shorter periods of time between each expulsion of debris. The wound size will reflect the amount of debris that needs to pass through the soft tissue fistula; the time the expelled debris is left on the skin until it is washed off; and the way the fistula is cared for and maintained.

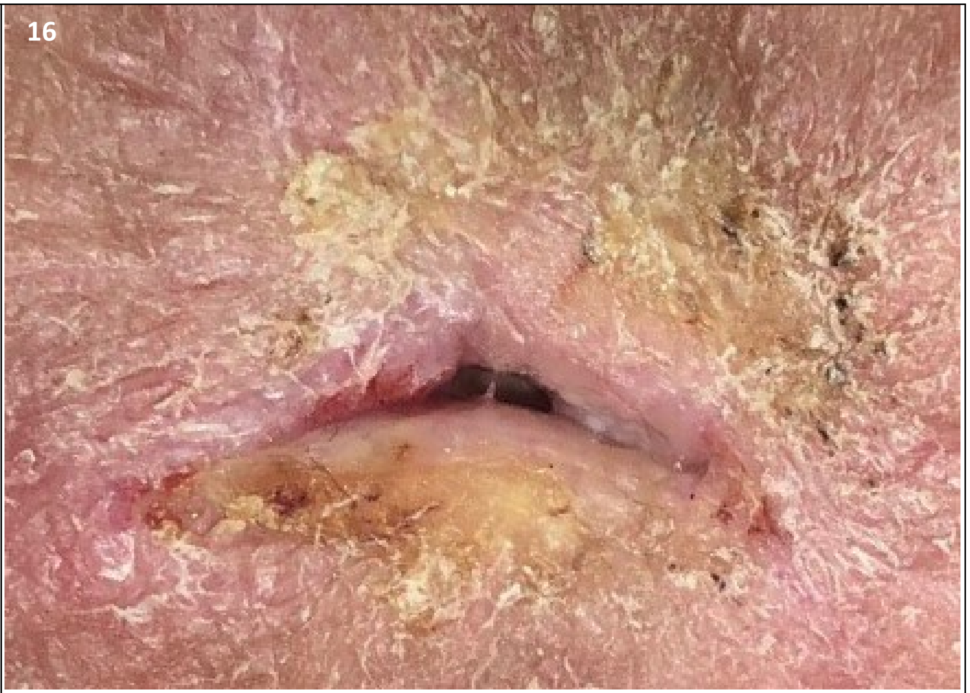

16

Day 229 7.5 months - Closeup

Length: approx. 10mm

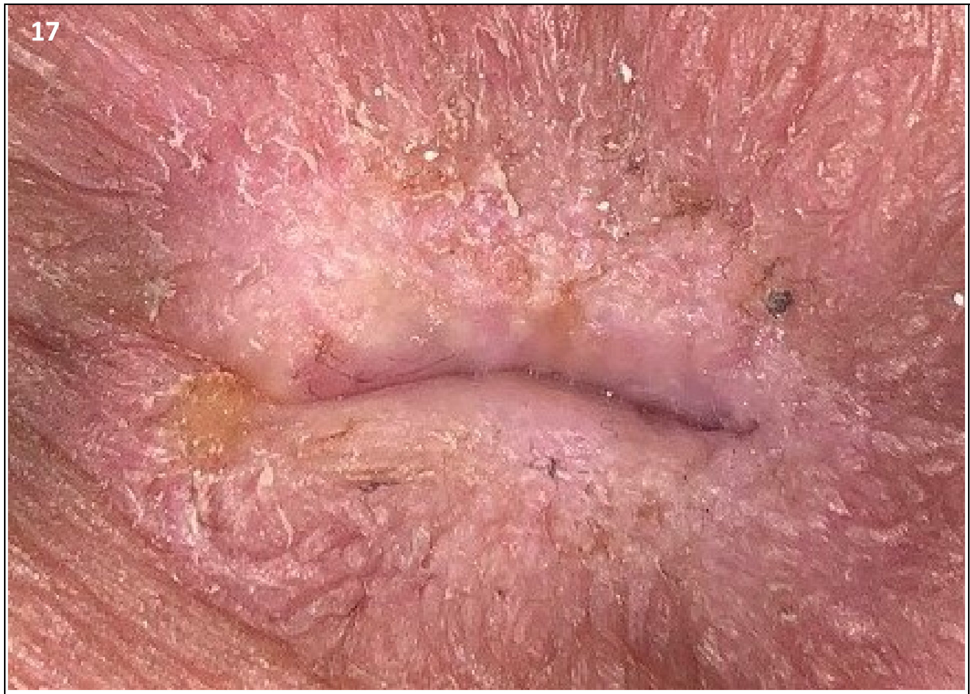

Day 256 8.5 months - Closeup

Length: approx. 10mm

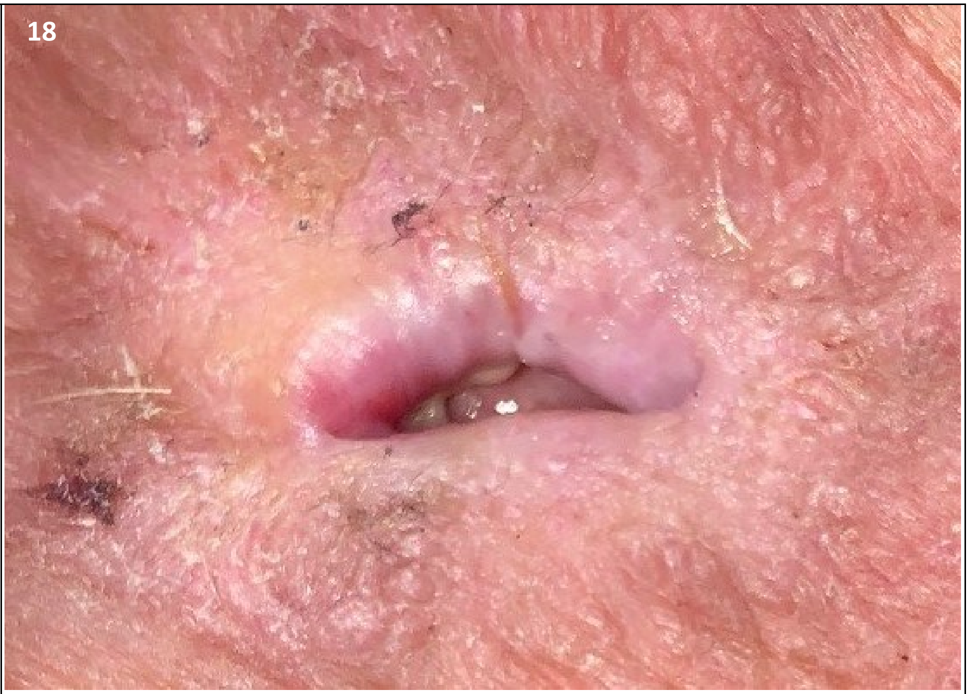

Day 258 8.5 months - Closeup

Length: approx. 5 mm

The fistula is pink and clean indside.

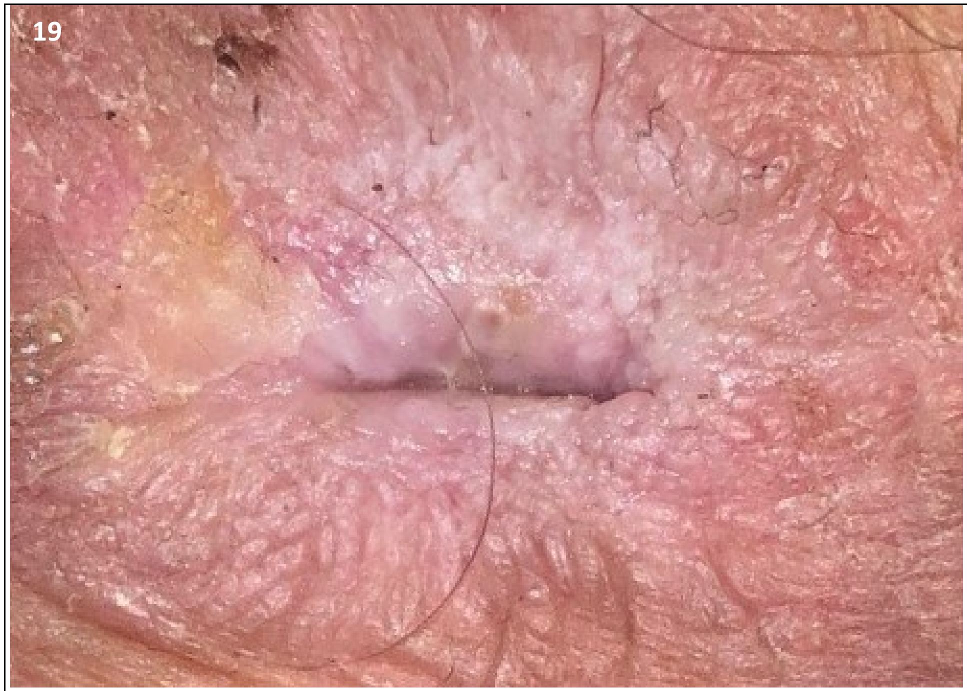

Day 265 8.75 months - Closeup

Length: approx. 5 mm

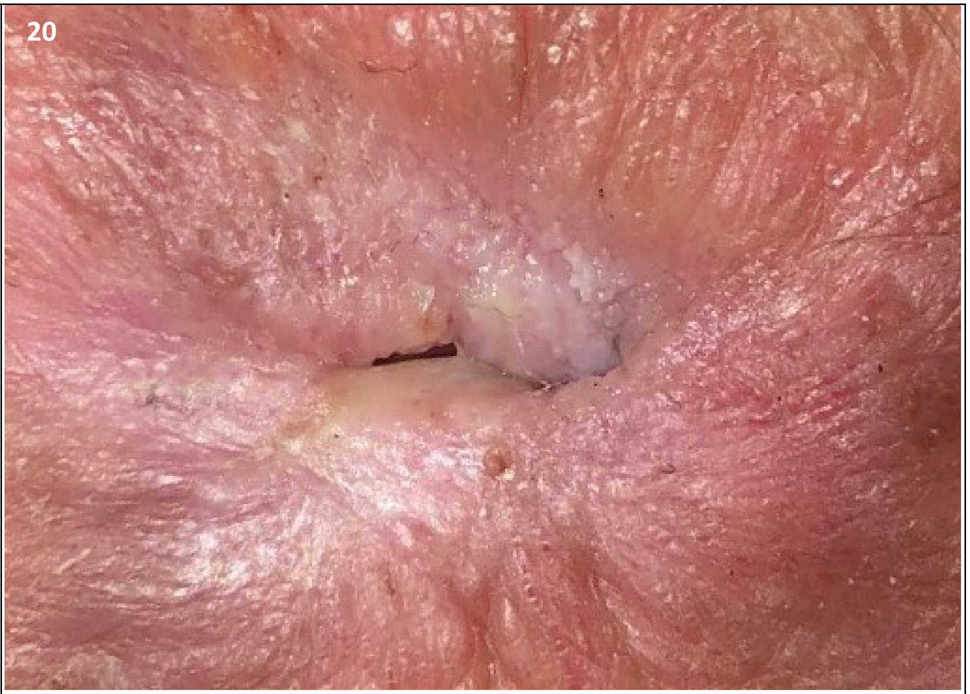

Day 304 10 months - Closeup

Length: approx. 5 mm

No nodulation remained in the skin in the area. The inside was slender and pink. The once wound and knobby surroundings were covered with plain, healthy new skin with a tinge of pink, as is the case for recently healed epithelium.

--------------- Part two ---------------

After approximately 2.5 months on systemic antibiotics for non-wound-related issue

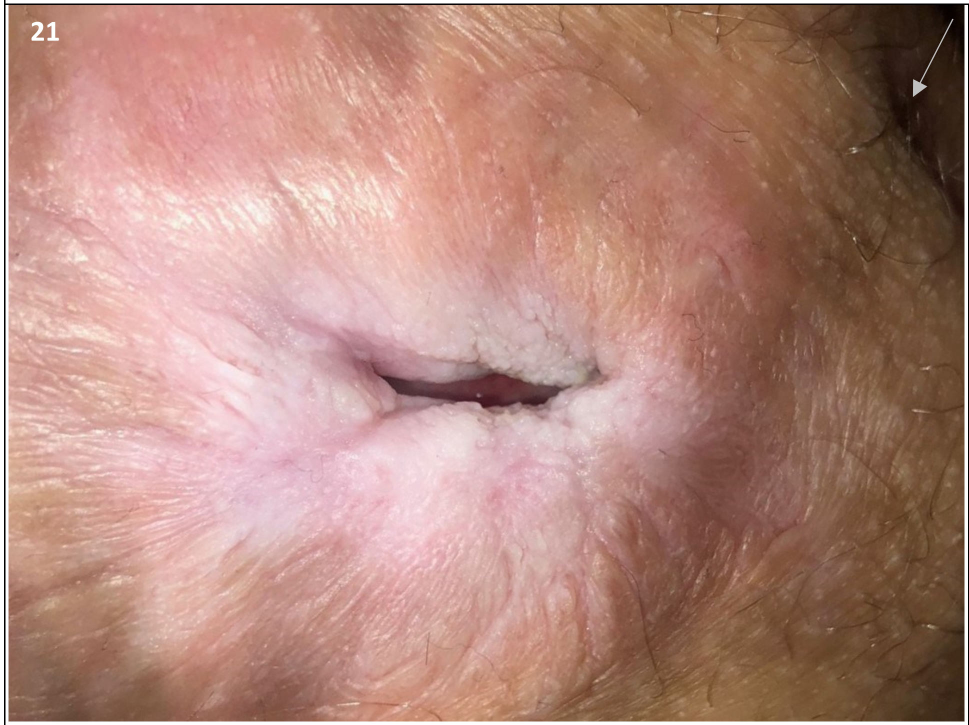

Following 2 months of systemic antibiotics for a non-related issue, without MPPT

Day 420 - 14 months since MPPT was first applied

Day 0 (after stop antibiotics)

Size of opening has more than doubled in size: 13 mm. A cavity has formed behind the skin and at the bottom is a visible gorge.

Skin is weak and affected scar tissue.

The wound is not manually forced open.

Grey arrow: surgical incision scar – pit

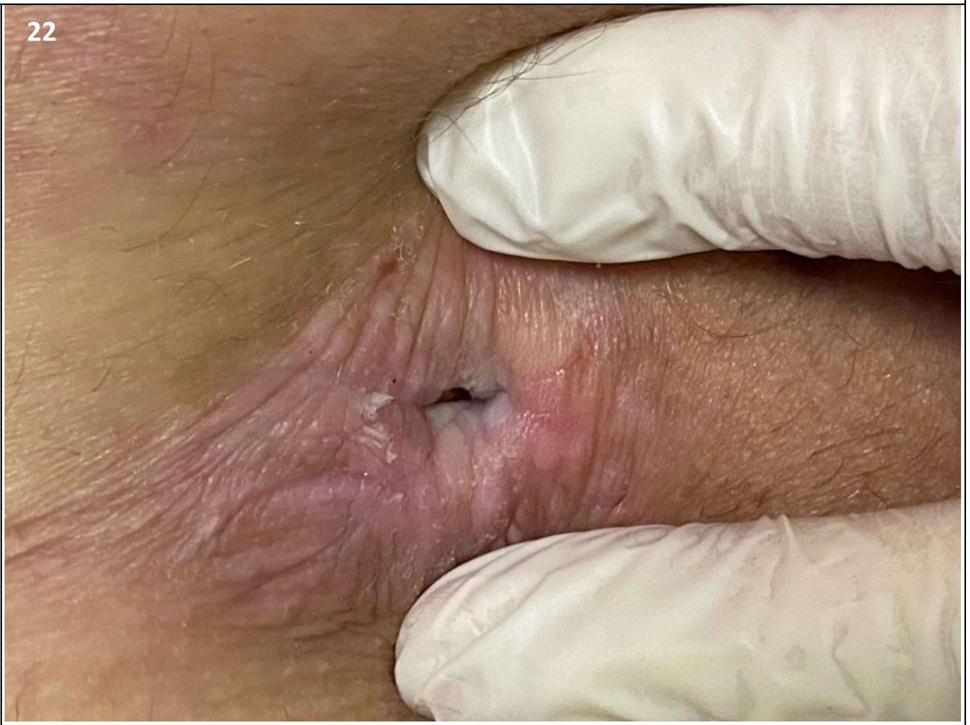

Day 428

Day 8 1 week (after stop antibiotics)

Size of opening: 5mm.

The wound is forced open in order to detect the opening.

Skin is recovering and gaining strength.

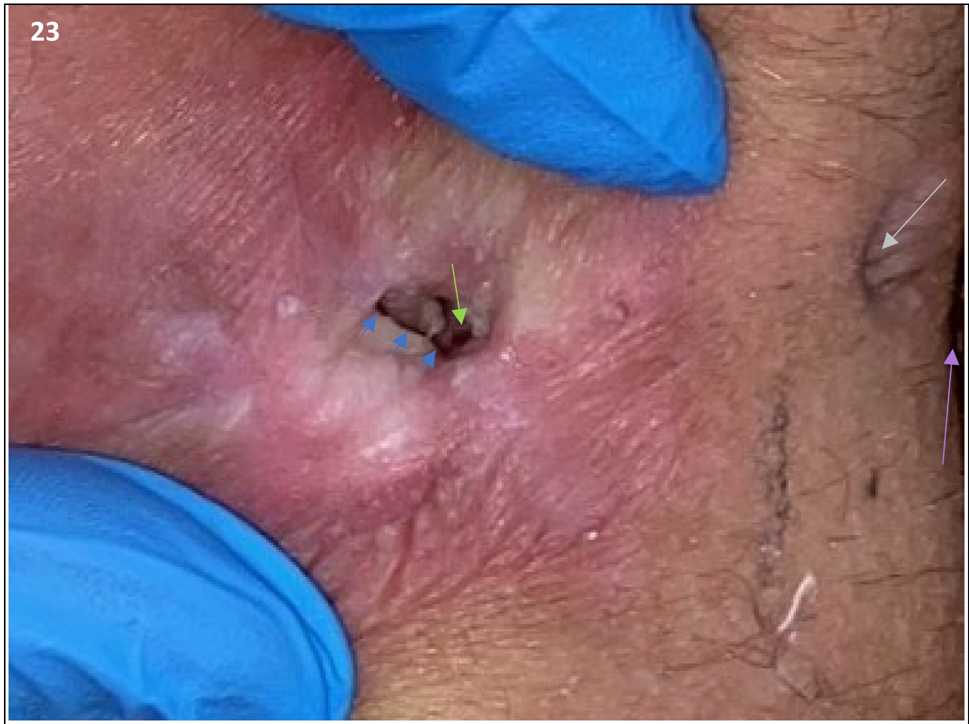

Day 431

Day 11 1.5 week (after stop antibiotics)

View of the inside of the wound where the openings from the two tracks are readily visible. They drain into a minimal void in soft tissue free of infection.

Grey arrow: surgical incision pit

Purple arrow: Natal cleft

Green arrow: right track opening

Blue arrows: The gorge – serving as the left track opening

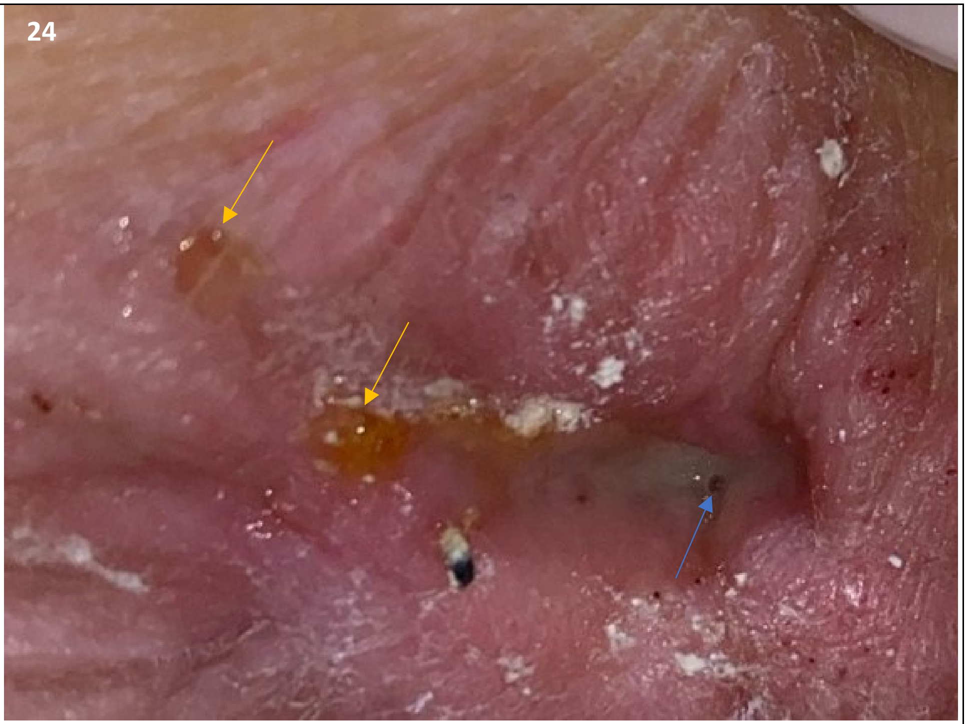

Day 507 - Closeup

Day 87 3 months (after stop antibiotics)

Picture taken before washing

View of the very small volume of intestinal matter as primary source of infection, mixed with immune debris from keeping the tracks free of infection, drained through the tracks during the last 24-hour period.

Blue arrow: tracks opening

Yellow arrows: drained fluid

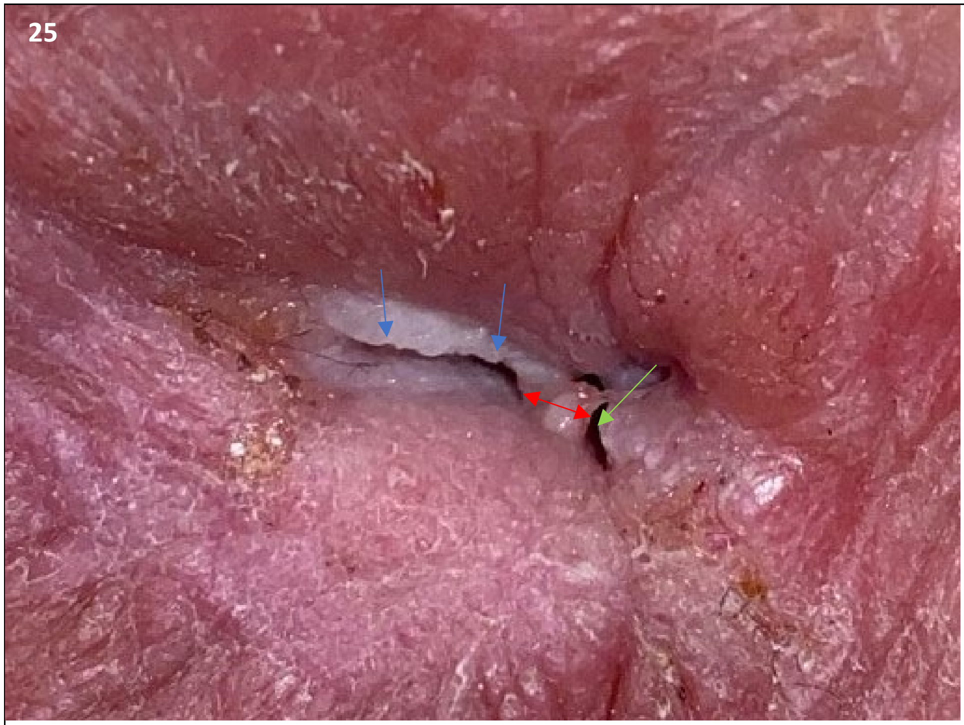

Day 528 - Closeup

Day 108 3.5 months (after stop antibiotics)

The two tracks seen 15 months earlier are again individually recognisable from the outside.

Green arrow: right track opening

Blue arrows: The gorge – serving as the left track opening

Red double-ended arrow: Epithelium separating the two tracks at skin level.

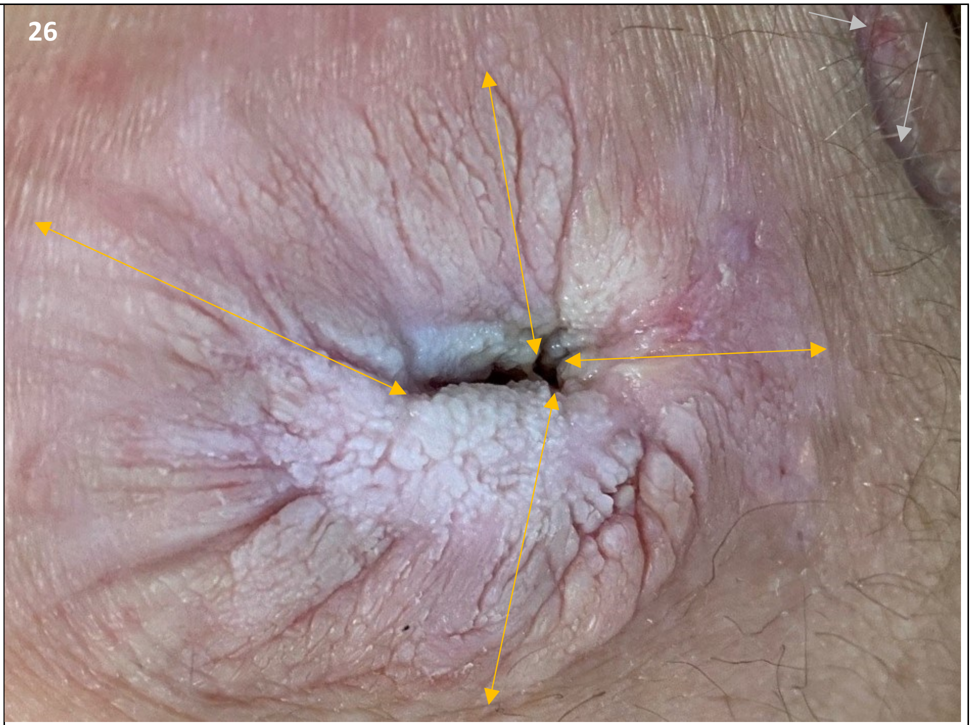

Following 2 weeks antiseptics and moisture occlusion – without MPPT

Day 572

Day 152 5 months (after stop antibiotics)

Day 0 (after stop antiseptics)

After a 2-week-period washing with the antiseptic, Chlorhexidine, treating with calcium alginate, and occluding with standard island foam dressing. Generalised maceration.

The wound opening is enlarged, and the two fistulas now exit and drain into a several mm deep cavity wound that has formed behind the opening in the skin. The incision pit is also macerated, and the skin has broken down in one location.

There has been a considerable increase in exudate and the surgical incision pit is also leaking.

Grey arrows: surgical incision pit. The small one shows the skin breakage

Yellow double-ended arrows: Wide band of nodulous infection in the skin.

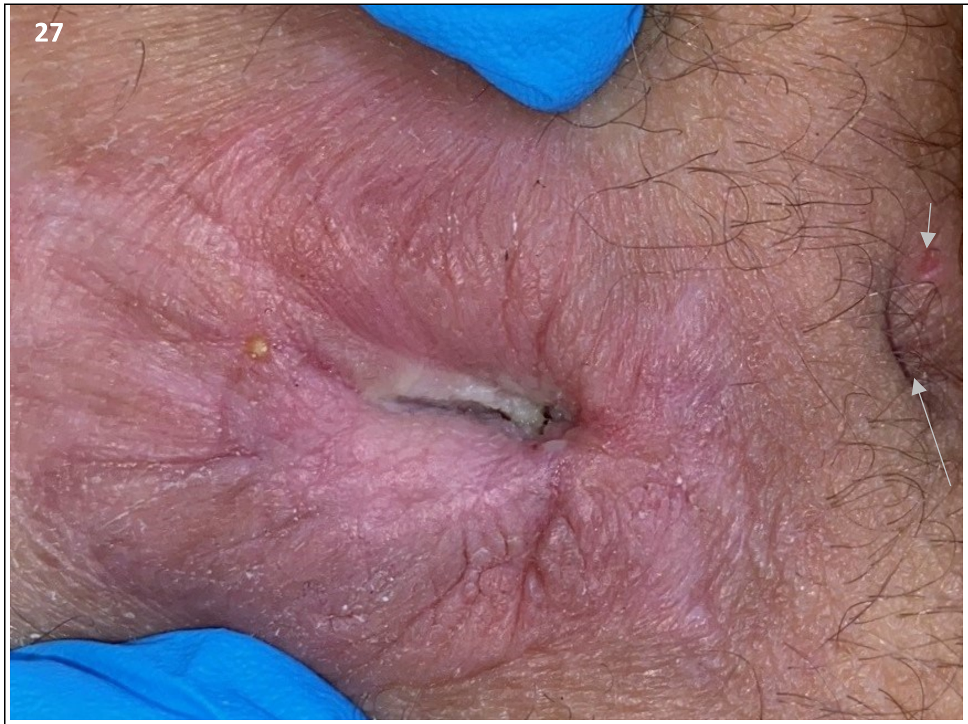

Day 576

Day 165 5 months (after stop antibiotics)

Day 4 (after stop antiseptics)

After 4 days MPPT reinstatement. The generalised maceration is gone. The nodulous skin infection is reducing and the epithelium in the surgical incision pit is recovering. The skin breakage (small arrow) is recovering but not fully epithelialized yet.

Grey arrows: surgical incision pit.

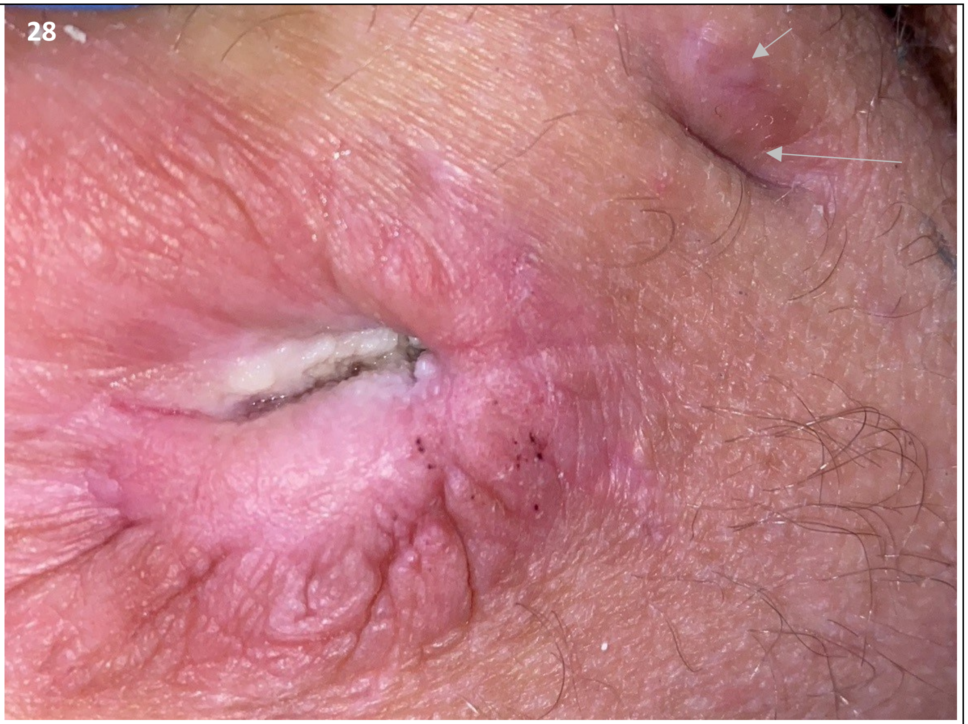

Day 623

Day 203 7 months (after stop antibiotics)

Day 51 1.5 months (after stop antiseptics)

the day MRI was performed which revealed anal fistula

The wound is maintained but now drains considerably higher levels of exudate and the skin is harbouring nodulous infection. The surgical incision pit is inflamed and discharging an extremely low volume. The skin breakage on its side (small arrow) has epithelialized.

Grey arrows: surgical incision pit.

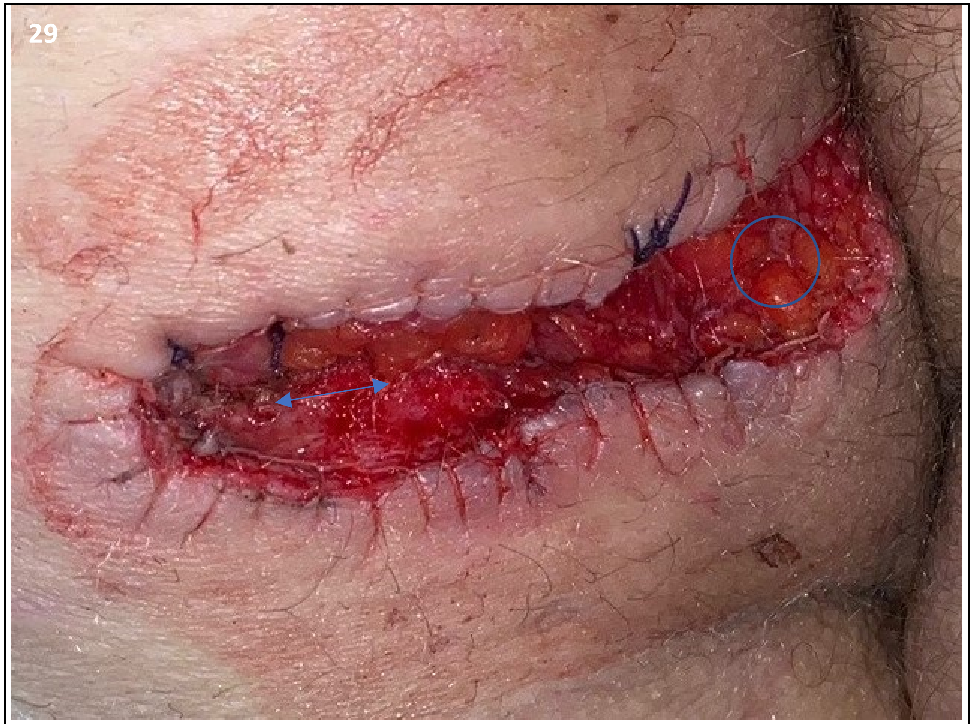

Day 680

Day 266 - 8.5 months (after stop antibiotics)

Day 108 – 3.5 months (after stop antiseptics)

Nearly 2 years after first application of MPPT

12-18 hours after surgery for an anal fistula with two sets of tracks draining to the skin.

Flap surgery to replace missing tissue was not required.

There was enough healthy soft tissue to allow coverage following surgery.

Double ended arrow: former wound area.

Circle: former surgical incision pit.

Wound number 35			Patient	SCI	
**Grade 4**	**3 months**	**Sacrum, ilium, ischium & femur**	**74-year-old female**	**10-15 years**	**L2 incomplete**

A 74-year-old lady had a 12 x 3 x 6 cm ulcer with 20cm and 18 cm deep sinuses along femur and the spine, respectively.

She had, 35 years prior, received very high doses of (megafraction) radiotherapy to treat cervical cancer. This had caused a non-traumatic gradually deteriorating spinal cord injury and the patient had been 100% wheelchair bound for the past 10 to 15 years. It possibly also caused the abscess next to the pelvic bone. This had gone unnoticed until 3 months prior when a sizeable patch of eschar revealed itself on the right-side lower back (pic 1). It was dressed with Manuka honey. Approximately a month and a half later (pic 2), the patient was hospitalised with sepsis; and after 4 days in ICU receiving IV antibiotics , the abscess was surgically debrided. This exposed an infected 10 x 2.5 cm wound opening, 6.5 cm deep albeit failing to identify viable tissue at the bottom as it was deemed too risky to remove anything further. The “floor” of the wound, as it appears in the pictures, was directly on top of the sacrum, ilium ala and the ischium which in their entirety suffered from chronic, severe, advanced osteomyelitis and constituted approximately 25% of the accessible wound. This extensive bone infection needed to drain its infective, corrosive debris and this was causing the uncontrollable, associated wound.

After one month’s daily packing with Manuka honey gel (Medihoney) and alginate gelling fibre (Durafiber) and covering with a foam dressing (Tegaderm) the wound had expanded further to 12 x 3 cm and formed a thick, dry slough on top of the entire surface of the exposed wound cavity, including a 1 cm thick hard leathery cap on top of the osteomyelitic bones under the “floor” of the wound (pic 3). The measurable depth down to the necrotic leathery cap was 6 cm. The medial and cranial edges, i.e. bottom and left in the pictures, respectively, suffered 4 cm deep, 1 cm wide undermining. This cavity between skin and bone/tissue was also full of dry, thick, very viscous slough. The skin surrounding the entire opening was dark red to purple coloured, inflamed, thickened and significantly hardened, resembling the consistency of cardboard - cellulitis was evident. The wound was highly malodorous, and the patient showed renewed signs of toxaemia. An MRI revealed a 20 cm deep, 3 cm diameter tunnel distally in the wound running along the femur along the main vessels and nerves. It was tightly stuffed with viscous “cheese-like” slough. Similar to the entire hip, the femur also suffered from chronic osteomyelitis and needed to drain into this tunnel and further into the wound. This was the situation when MPPT treatment was started (pic 3).

Due to the extent and severity of the osteomyelitis the patient was also referred to the national specialist bone infection unit under Oxford University Hospitals. The consensus of the specialist team was, that any type of surgical intervention (osteomyelitis mitigation or debridement of wound and/or tunnel) was too risky. To reconsider the possibility of surgery for the osteomyelitis, they specified what the treatment of this associated wound needed to achieve: 1) Debride the wound; 2) Maintain the wound without it growing in size or complexity – ideally reduce the size; and 3) Keep the infection and amount of toxins in the wound below the threshold of causing toxaemia. All this, whilst the extensive bone infection continued draining its infectious and toxic waste material into the sinus and into the wound.

The wound progressively cleared the necrotic cap and granulation tissue appeared (pic 4 & 5) and gradually grew stronger (pic 6 and 7 onwards). The malodorous infection of the entire wound started to resolve. By autolytic debridement and concomitant steady granulation, the wound got rid of the necrotic material and built up considerable amounts of new tissue. The broad brim of dark red to purple coloured skin surrounding the wound edges evidencing cellulitis, contracted in width and gained a healthy pale pink (pic 6 onwards). The hardened skin turned softer and bendable and started to fuse with the soft tissue, thereby slowly clearing the undermining. Further, off-white coloured new epithelium generated on the edges themselves, thus decreasing the size of the wound opening (pic 7 onwards). The area on top of the severely infected ilium, which was covered by strong, dry slough, cleared and the covering fascia developed a healthy looking white and structured appearance (pic 7 onwards). On top of this fascia, granulation tissue slowly began to grow across the

exposed osteomyelitic bone area (pic 7 & 11) thereby gradually closing the undermined huge gap between the skin and the body (pic 7). After 5 weeks, the 20 cm deep tunnel was free of all slough and the application regime was changed to once every other day (pic 9). After another 6 weeks, the wound had reduced approximately 50% in volume, and there were no signs of soft tissue infection (pic 11 & 12). The cranial part of the wound was still steadily removing necrotic structures, including non-viable vessels, but the area was contained, non-infected and reducing. The patient was overall performing very well with no signs of toxaemia. At no point during the MPPT treatment period was the patient confined to bedrest.

The treatment goals set by the bone-infection specialist team had been exceeded. For reason unrelated to MPPT, the treatment regime was changed to local standard care. From now on, every other day the wound was applied polyhexanide/PHMB 0.1% and Betaine gel (Prontosan Gel X), packed with carboxymethyl cellulose gelling fibre (KerraCel) and secured with a foam dressing (Tegaderm foam). After 10 days, due to a huge surge in infection and to severe damage to both bone tissue and soft tissue, the treatment regime was changed back to MPPT. (Sams-Dodd and Sams-Dodd, 2020). The extensive damage caused to the bone structure resulted in a substantial increase in the amount of infective material draining from the infected bones. MPPT treatment was now used daily to contain the infection and keep the soft tissue free from infection.

For the following 8 months, while the patient waited for bone surgery, the wound continued to improve and reduced in size by 90% (pics 13 & 14).

The surgery for osteomyelitis was successfully carried out in 3 individual operations followed by flap surgery at the Royal National Orthopaedic Hospital over a 3-month period (pic 15).

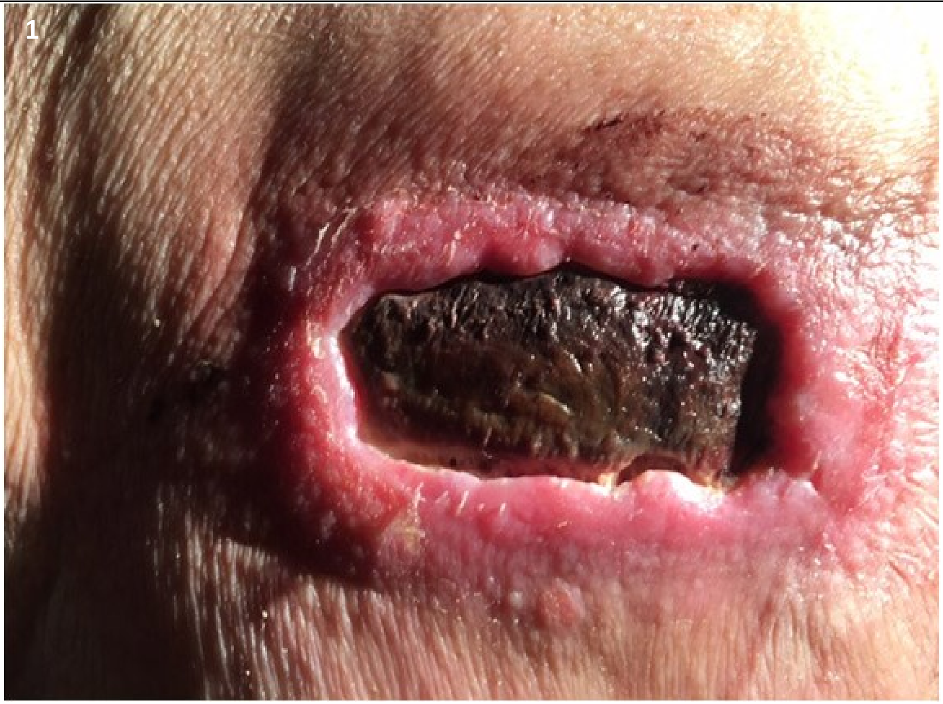

2 ½ - 3 months before MPPT

Upon detection of the wound

Eschar and cellulitis

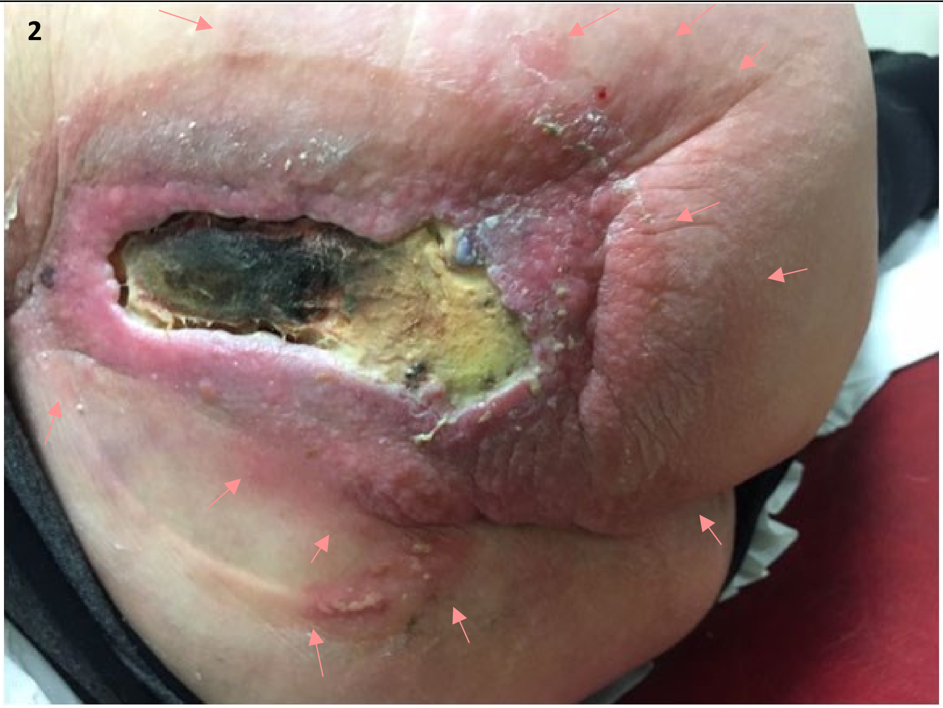

38 days before MPPT

Before surgical debridement after surviving sepsis

Dry eschar covering the cranial half and slightly softer eschar the distal half of wound.

Cellulitis.

LIght red arrows: Ongoing diffuse spread of the infection in and under the skin

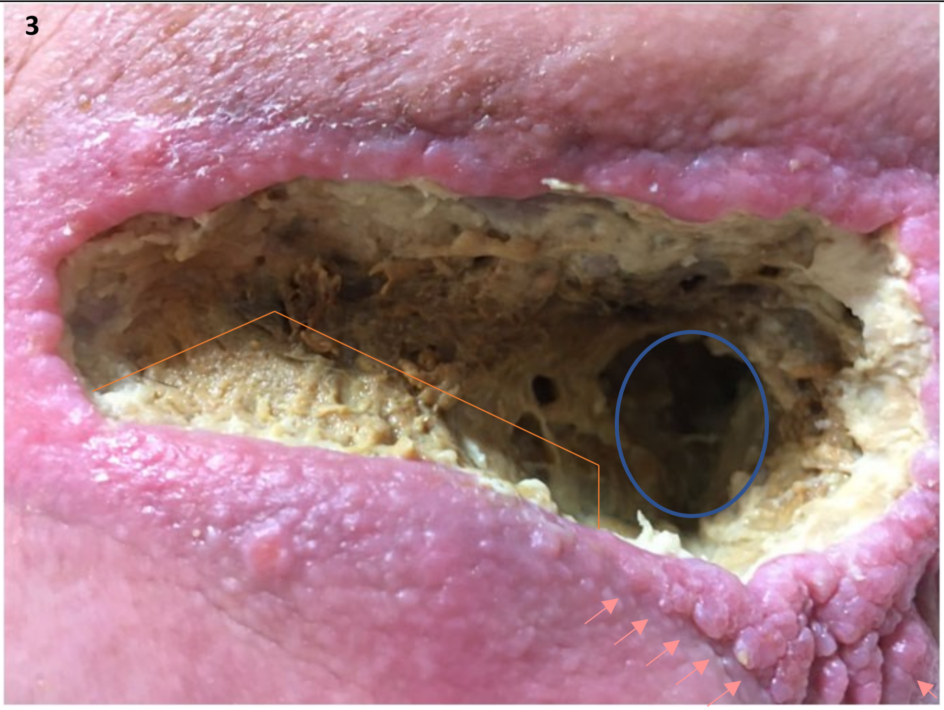

4 days before MPPT

34 days after surgical debridement

Opening 12 x 3 x 6 cm. Surface covered with a 1 cm thick, dry, tough, leathery cap of slough.

Cellulitis.

Blue circle: area of 20cm deep 3 cm wide tunnel along femur, stuffed with viscous slough.

Orange: ”floor area” on top of necrotic bone due to chronic osteomyelitis, approx. 25% of accessible wound area

Light red arrows: Nodulation within the severe cellulitis (example)

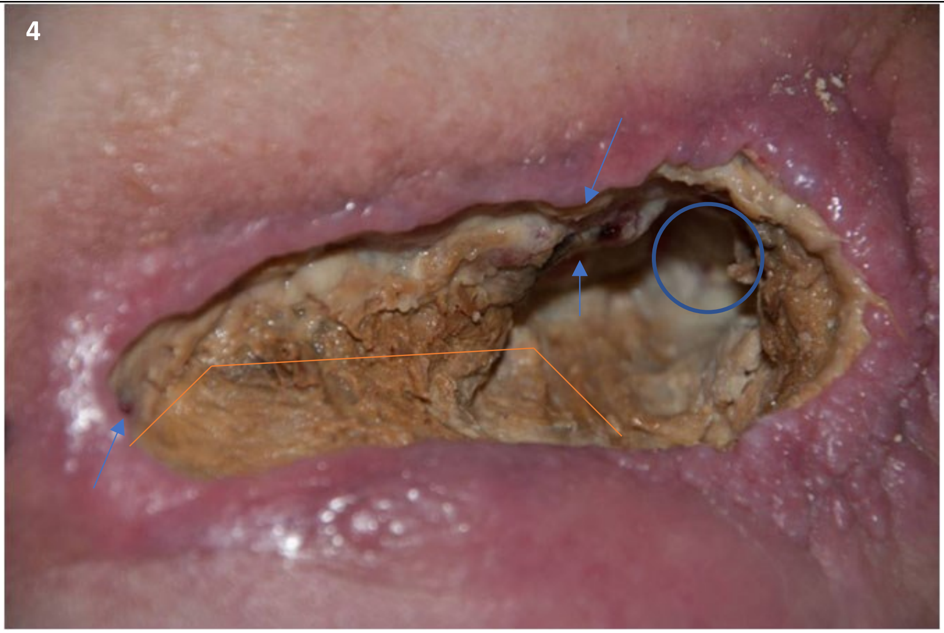

Day 1

After 24 hours MPPT

Blue arrows: single granulation buds

Blue circle: area of 20cm sinus along femur.

Orange: ”floor area” on top of necrotic bone due to chronic osteomyelitic bone

<table>
<tr>
<td>

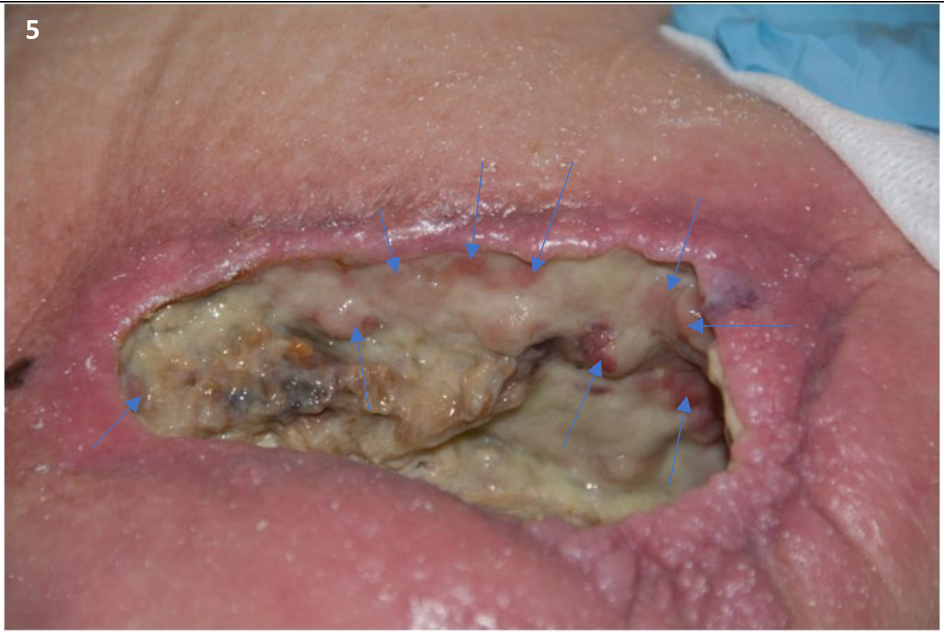

Day 9

Necrotic leathery cap is much thinner.

Cellulitis

Blue arrows: Granulation showing in ceiling area

</td>
<td>

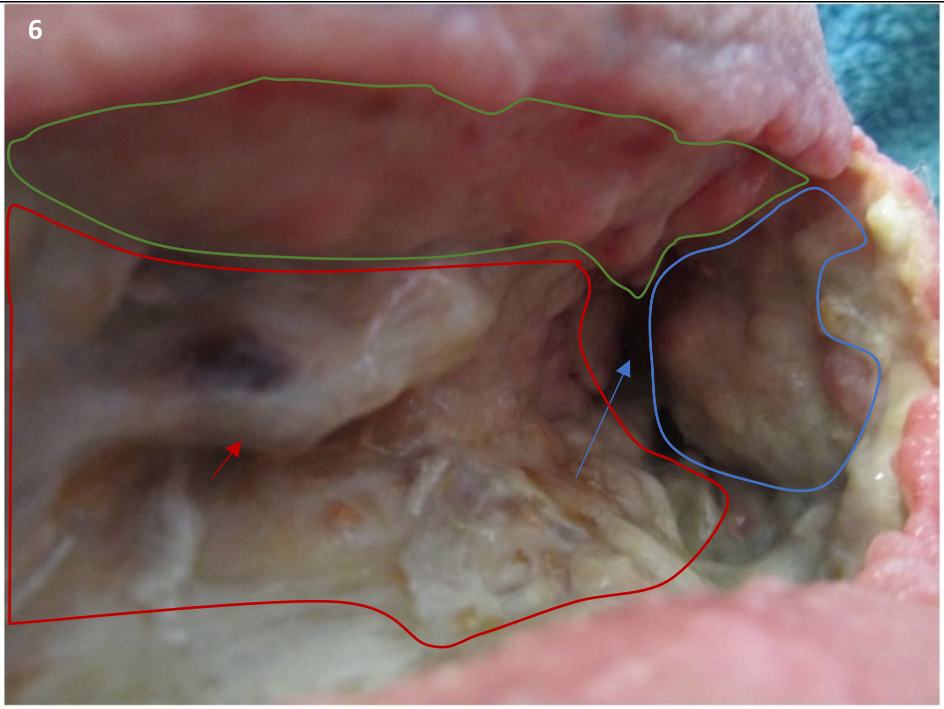

Day 25

Cellulitis has cleared.

Green: Entire wound ceiling granulating

Blue: Distal wall building new tissue via granulation, despite the relatively thin layer of slough on top, which is simultaneously reducing via autolytic debridement.

Blue arrow: Tunnel entrance/exit area.

Red: Autolytic debridement clearing out necrotic tissue, including vessels. The red arrow points to a necrotic vessel.

</td>
</tr>
</table>

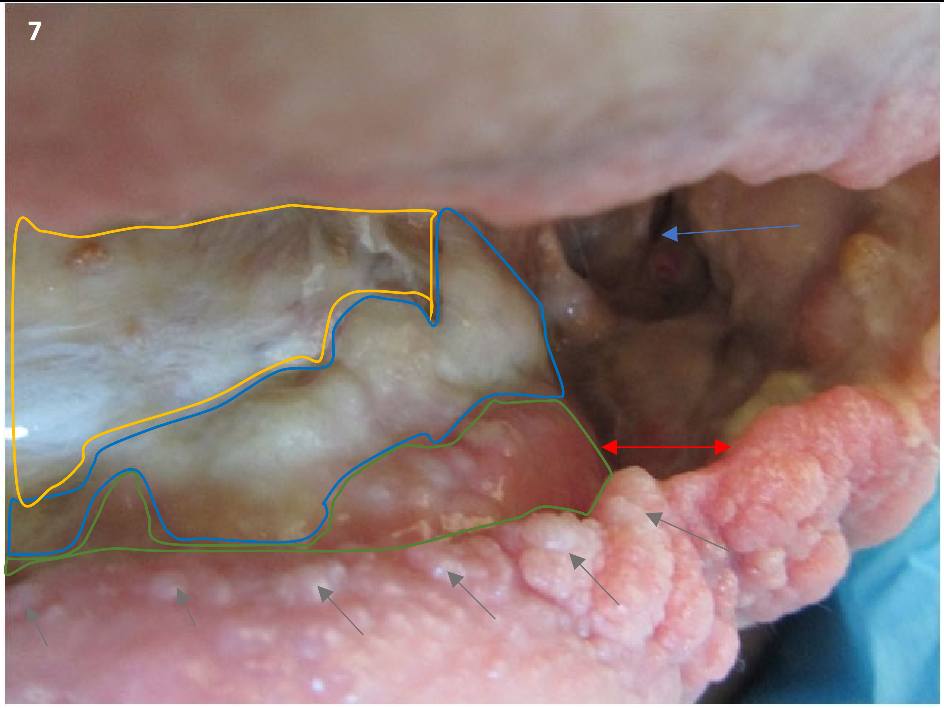

Day 28 1 month

The tunnel is gradually being cleared out. All stages of tissue regeneration are recognisable in the "floor" area. Granulation in the soft tissue walls lining the tunnel. The undermined gap between the body and the skin is cleared of slough and is gradually filling in with new granulation tissue.

Blue arrow: Tunnel entrance/exit area

Red arrow: Undermining, detachment of skin

Green: Granulation tissue maturing, i.e. undergoing antiogenesis and thereby changing from opaque white into pink.

Blue: Granulation germ cells before angiogenesis. This granulation is moving in on top of the fascia (yellow).

Yellow: Fascia covering the osteomyelitic bone is whitish and structured.

Grey arrows: Epithelialising skin edges

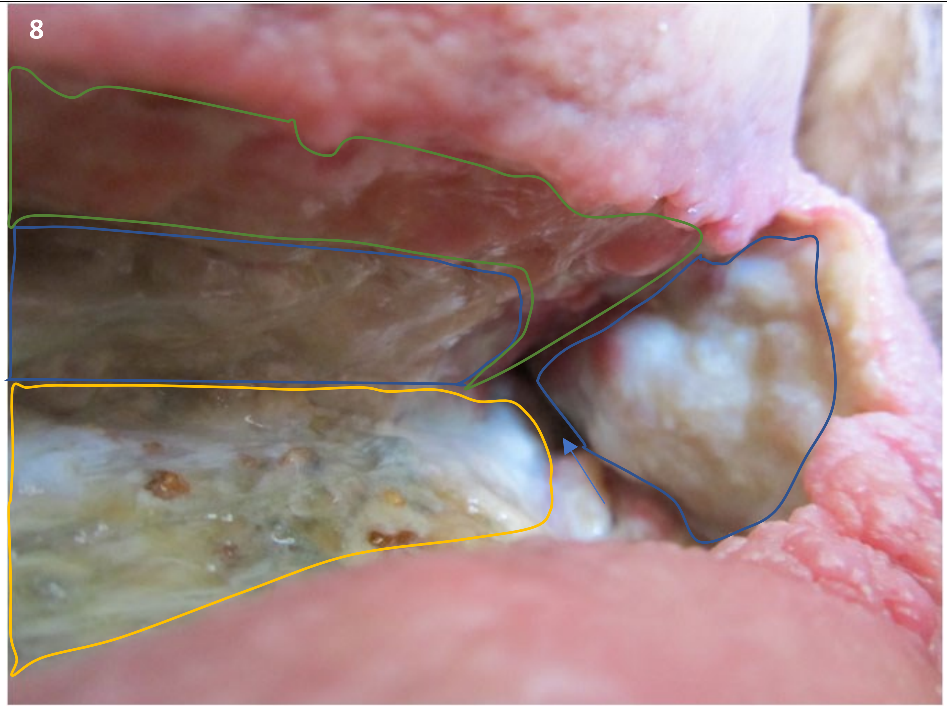

Day 51 1 ¾ month

Tissue regeneration in "ceiling", distal wall (right) and deep floor in front of the sinus entrance. The sinus is now cleared of slough.

Green: Granulation tissue maturing

Blue: Granulation germ cells before angiogenesis

Yellow: Fascia covering the osteomyelitic bone is whitish and structured.

Blue arrow: Tunnel entrance/exit area – granulating and regenerating and narrowing, but not closing over.

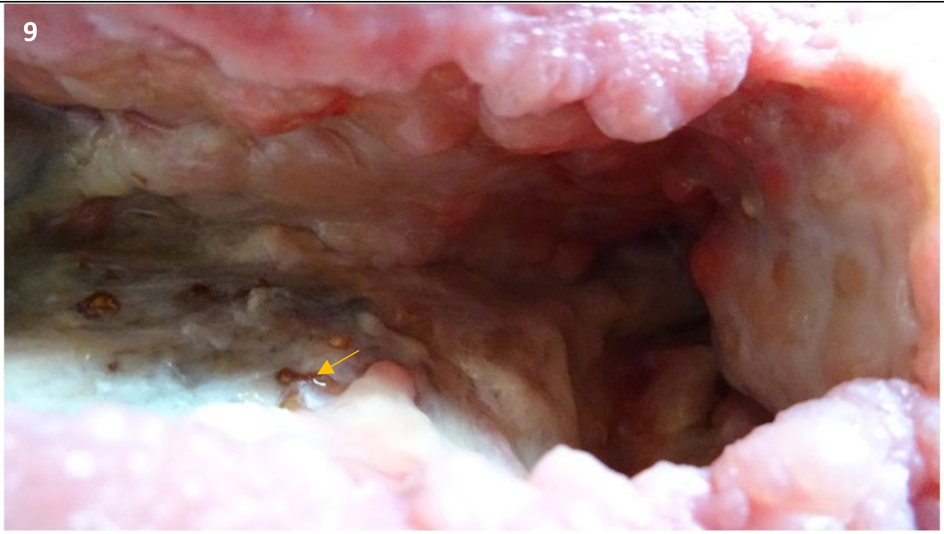

Day 63 2 months

The 20 cm sinus along the femur now free of slough and infection, regenerating and reducing in size but remaining open and able to drain the debris from the osteomyelitic femur into the wound.

Yellow arrows: Deformed miscoloured osteomyelitic bone not covered by healthy fascia but keeping an intact surface

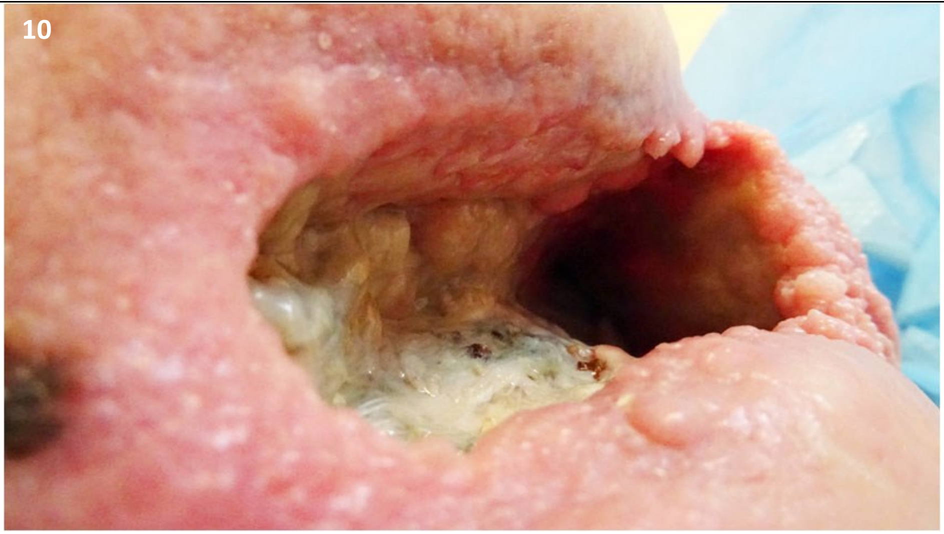

Day 102 3.5 months

Whole wound is granulating strongly except the floor area consisting of necrotic bone covered with a whitish fascia.

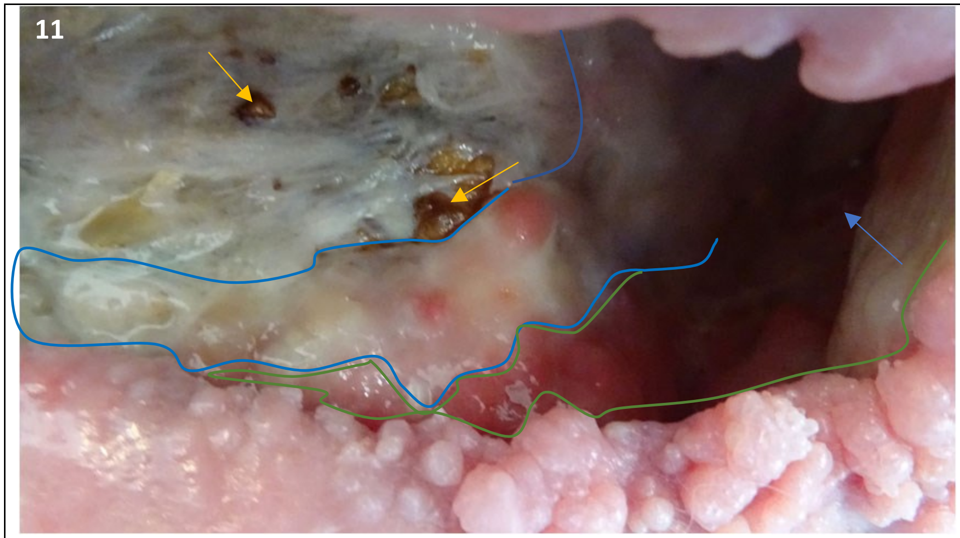

Day 112 - 1 "Floor" area, distal view

3 ¾ months

Granulation building up the 4 cm deep 1 cm wide undermined gab between the skin and body.

Blue: Granulation germ cells before angiogenesis

Green: Granulation tissue maturing

Blue arrow: Pointing into tunnel entrance/exit area

Yellow arrows: Deformed miscoloured osteomyelitic bone not covered by healthy fascia but keeping an intact surface

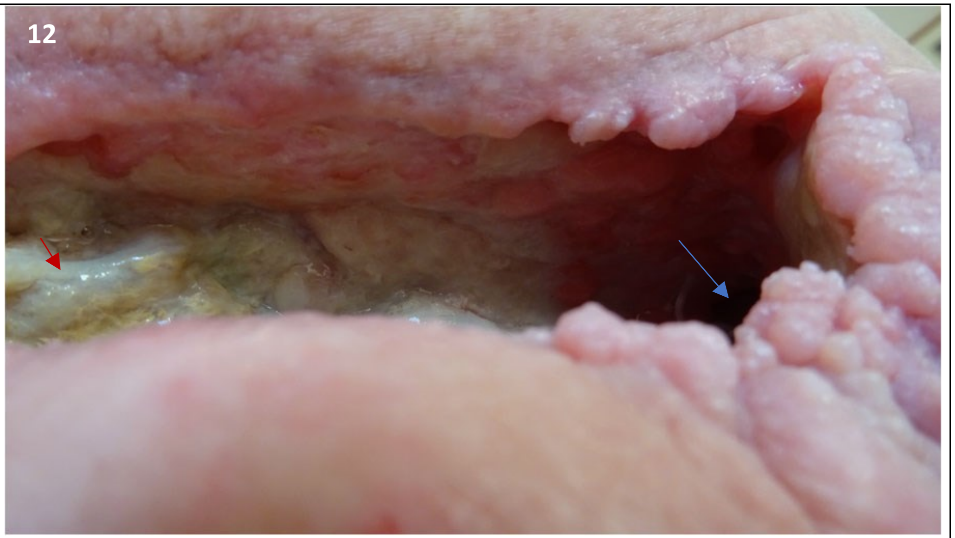

Day 112 - 2 "Ceiling" area, distal view

3 ¾ months

Wound volume has reduced by at least 50% and the femur sinus is clean and reducing in diameter. Treatment goal achieved (Sams-Dodd and Sams-Dodd 2020).

Blue arrow: Tunnel entrance/exit area

Red arrow: Necrotic vessel undergoing removal via autolytic debridement, but with no associated infection in the adjacent soft tissue.

13

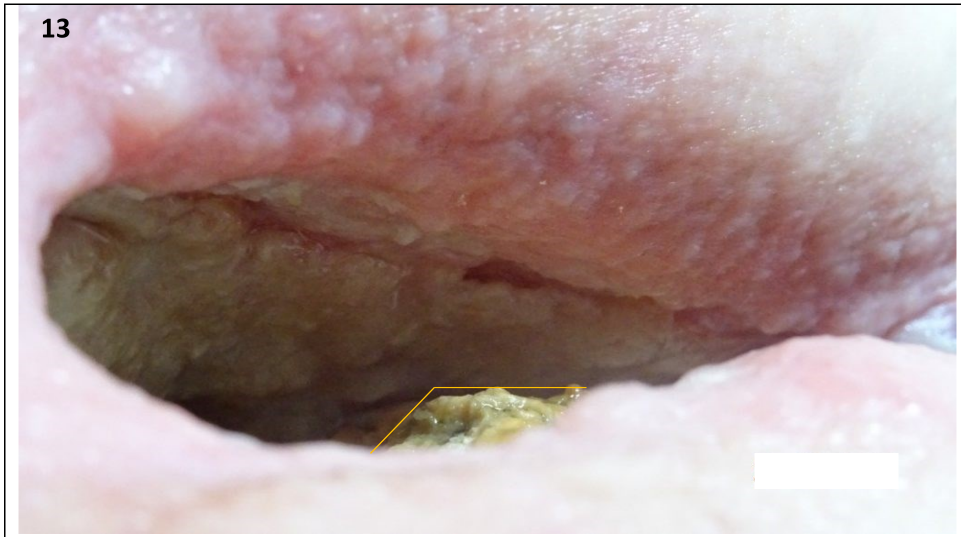

12 months – 1 Cranial view

Wound volume reduced by 90%

Whole wound granulating. The characteristic budding structure displays the characteristic shiny off-white colour of the germ layer, which is yet to vascularise and mature.

Yellow: Necrotic bone in floor area broken up by PHMB-betaine 8 months prior

14

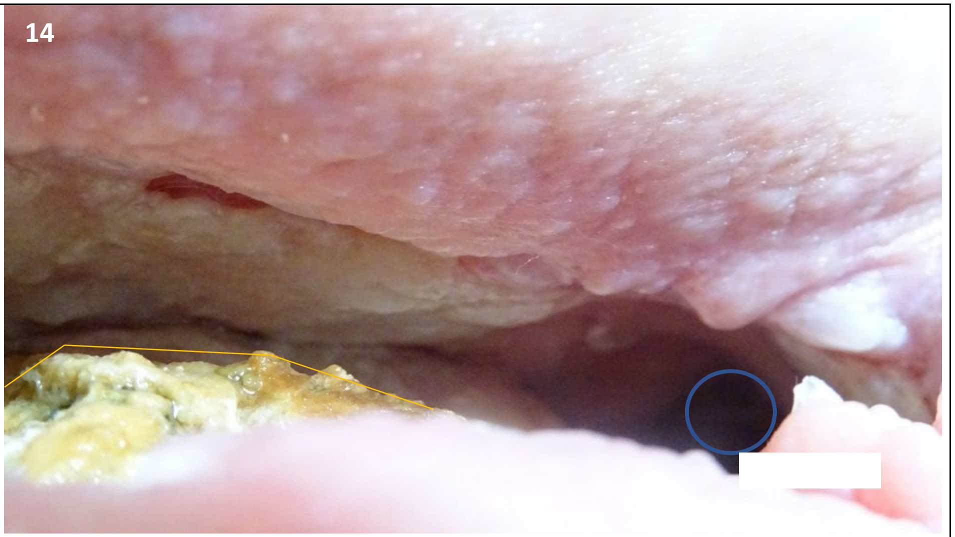

12 months – 2 Distal view

Wound volume reduced by 90%.

Silvery-white tinge is granulation-germ-layer before angiogenesis

Blue circle: Clean and granulating tunnel

Yellow: Necrotic bone in floor broken up by PHMB-betaine 8 months prior.

15

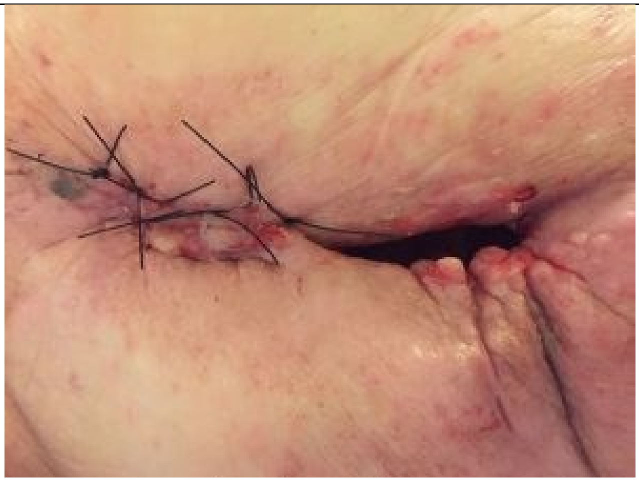

16 months

After completion of stage 1 of 3-stage orthopaedic surgery for osteomyelitis, to be followed by plastic surgery.

Wound number 36			Patient	SCI	
**Grade 4**	**many years old**	**Ischial tuberosity**	**84-year-old female**	**60 years**	**T5 complete**

This pressure ulcer had been causing problems for very many years.

The ulcer opening was a 6 cm x 2 cm, leading into an expanding 10 cm deep cavity that led into 2 additional, wide, 3 cm deep tunnels in both directions along practically exposed bone palpable from all angles. The entire area, skin as well as the soft tissue adjacent to the wound and tunnels, was severely infiltrated, heavily infected, and rapidly deteriorating (see pic 1 & 2).

The underlying osteomyelitis had been treated with IV Piperacillin/tazobactam (Tazocin) for 8 months directly followed by oral antibiotics for the past 2 months but nothing had brought down the CRP infection marker. The oral antibiotics for the osteomyelitis were continued for another 2 months after initiating MPPT. For as long back as could be remembered, the wound had been irrigated with PHMB/ polyhexanide-Betaine, an antiseptic-surfactant combination (Prontosan) then packed with ionic-silver impregnated carboxymethylcellulose packing ribbon (Aquacel Ag) and connected to NPWT (vacuum pump).

MPPT brought the soft tissue infection under control, induced tissue regeneration (pic 3), and after 5 months the wound opening had reduced from 60 x 20 mm to a 12 x 12 mm opening (pic 4). The interior volume of the cavity continued to reduce via tissue regeneration. The tissue ventrally to the bone was restored to the level of the bone. The remaining cavity gradually reduced to a non-infected fistula of a similar diameter to the orifice (pic 5). From a wide area in both directions along the bone, this fistula passed the infectious debris draining from the bone to the surface. The fistula continued to reduce in diameter and at 9 months finally formed into 3 very narrow individual fistulas draining into and through the one 10 x 10 mm opening in the skin (pic 6 & 7).

The wound continuously improved during the 9 months MPPT was used (pic 6 & 7). Then it was, very unfortunately, washed with the antiseptic PHMB and the surfactant Betaine and packed with the antiseptic silver for 12 hours (exactly as before MPPT, detailed above). This caused a severe set-back with loss of tensile strength in the tissue inside the wound as well as in the skin. It also caused a considerable increase in discharged material. This reaction was consistent with previous findings (Sams-Dodd and Sams-Dodd, 2020). The wound was, however, in the process of being brought back on course (pic 8) when, at 10 months, MPPT was discontinued for reasons unrelated to the trial.

During the 10 months MPPT was used, 2 courses of systemic antibiotics were administered for pneumonia and gastrointestinal infection (Clostridium difficile), respectively. No antibiotics for the osteomyelitis were required.

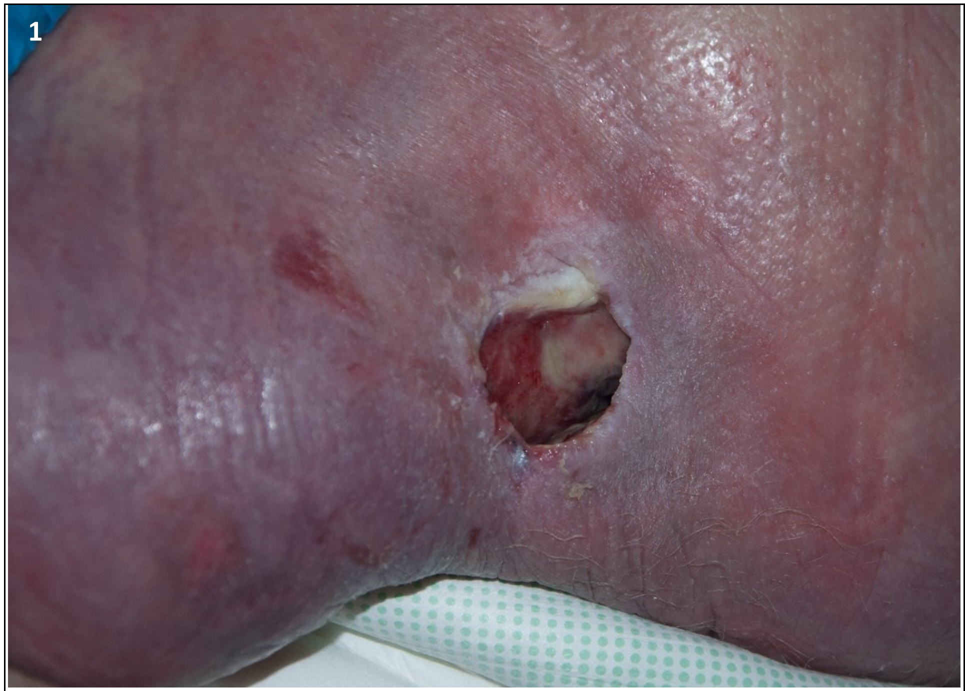	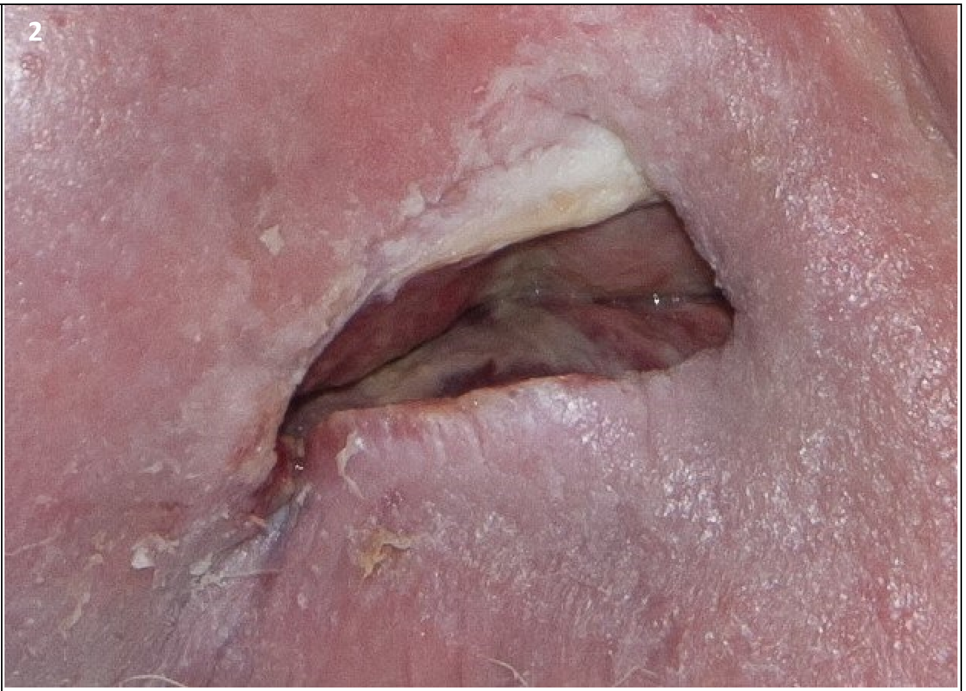
**Day 0**<br>**Just before MPPT**<br>All skin is dark purple and heavily infiltrated.	**Day 0**<br>**Just before MPPT**<br>The wound edges are dull and breaking down; 60 x 20 mm opening.<br>The inside is greyish with deeply embedded tough slough throughout the 100 mm deep, expanding cavity with two 30 mm wings along bone in both directions.<br>The wound volume is calculated to 138 cm^3.

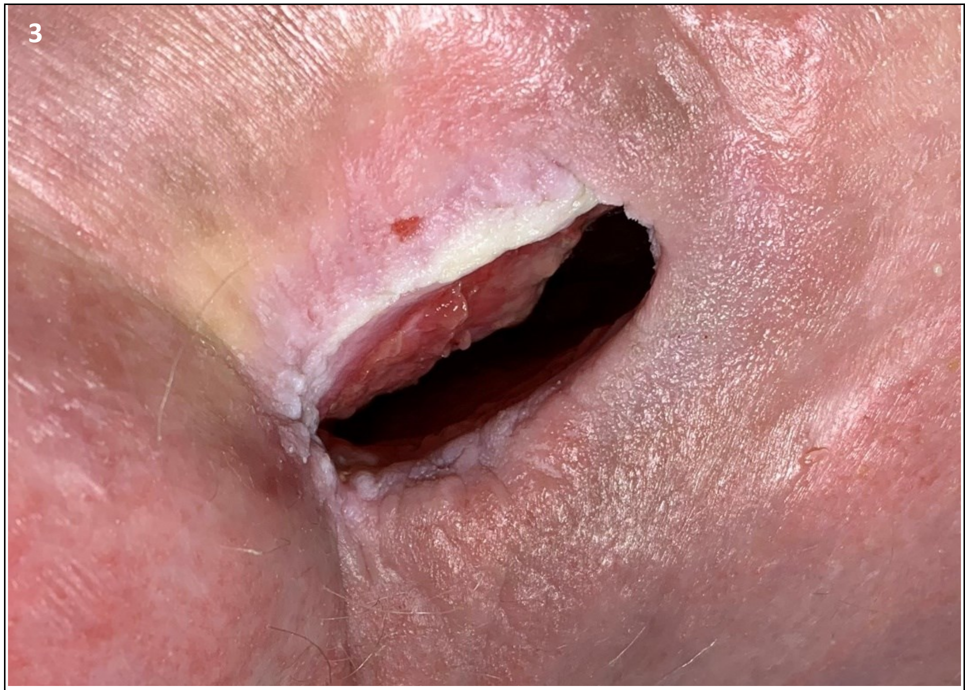

Day 37

The skin borders are epithelializing.

The inside has been cleaned of non-viable tissue and is granulating.

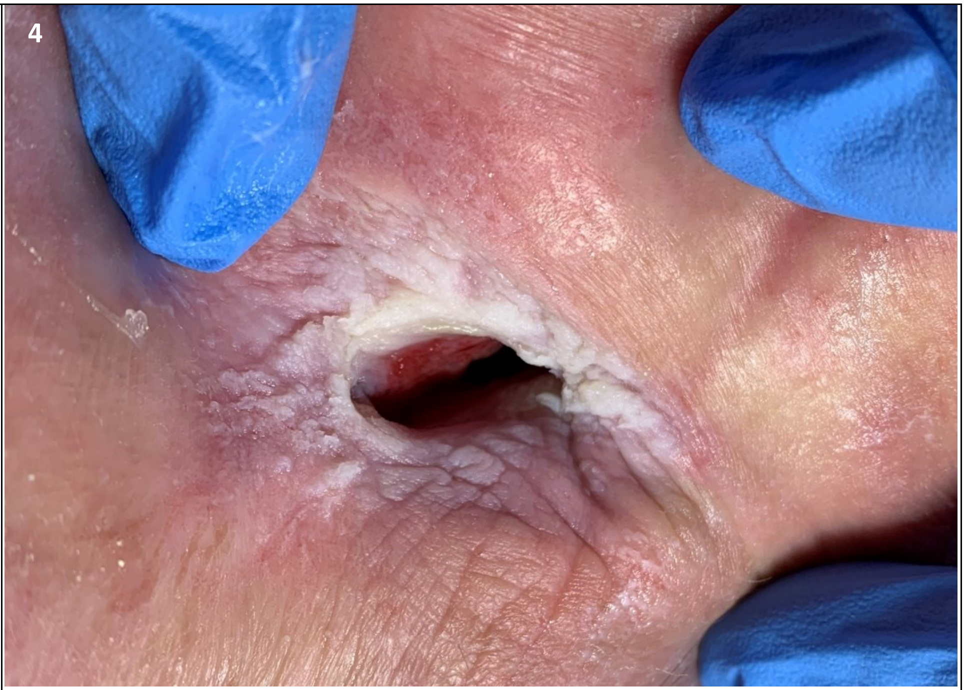

Day 165 5.5 months

The wound contains no slough and is characterised by only granulation tissue.

The volume has reduced by an estimated 86%.

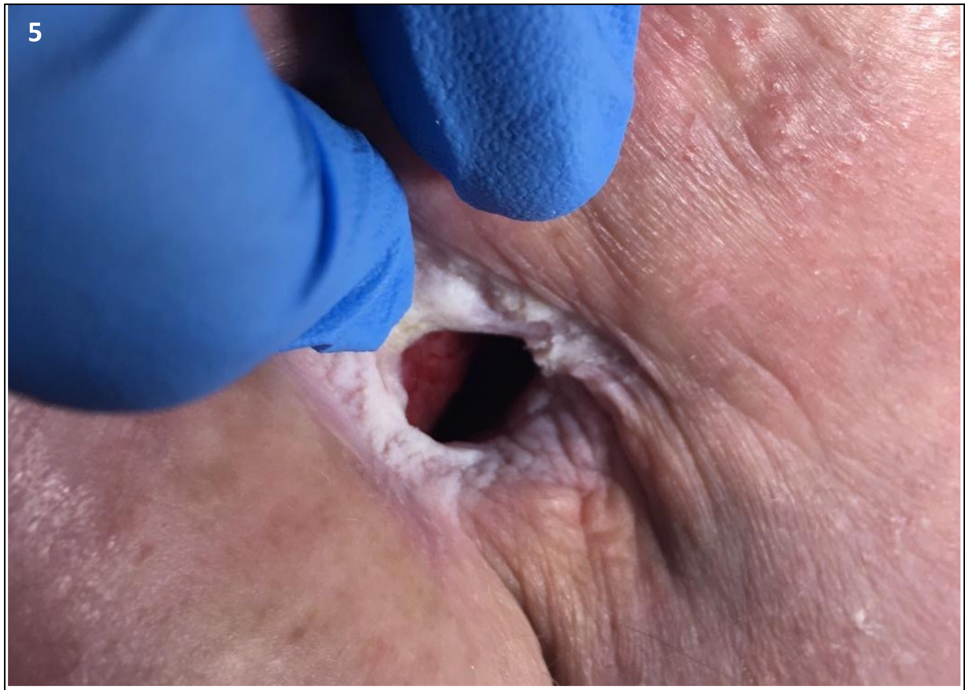<br><br>**6 months Day 190**<br>The volume has reduced by an estimated 92%.	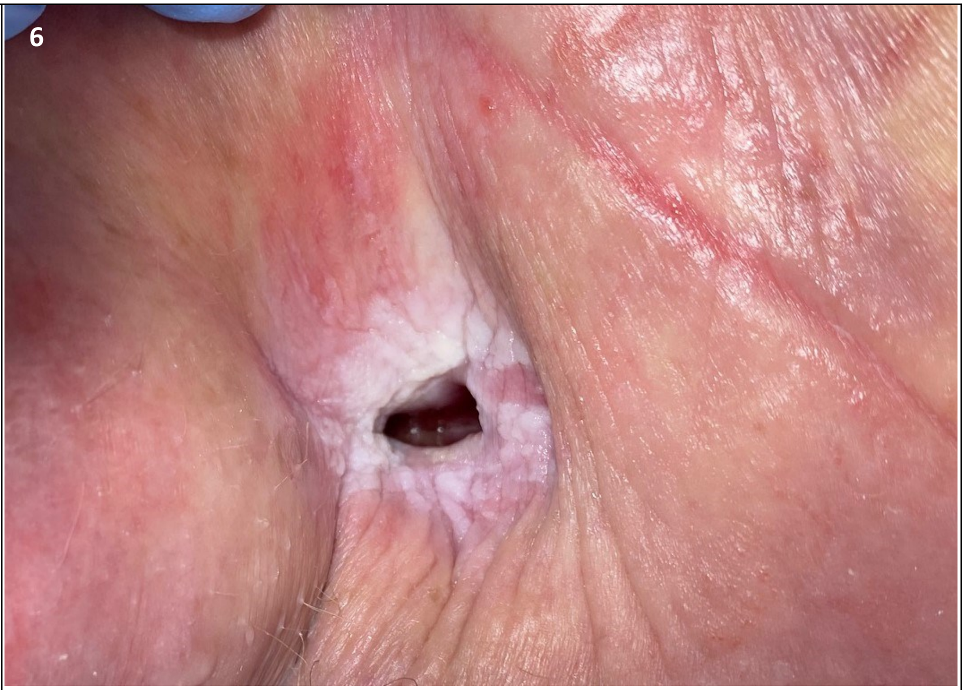<br><br>**9.5 months Day 288**<br>the wound opening is 10 x 10 cm. The cavity has filled up and now displays three very narrow tracts passing the debris from the bone.<br>The skin is healthy looking and displays no signs of purple infiltration.

7

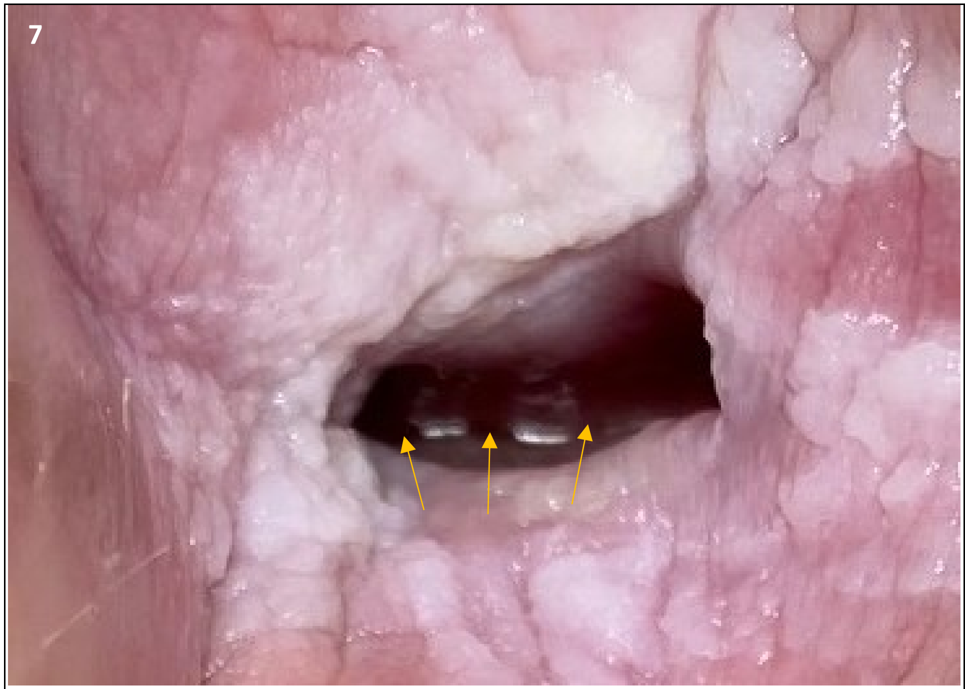

9.5 months Day 288 - Closeup

The inside of the wound has filled up with granulation tissue except for three very narrow fistulas that need to remain open in order to drain the infectious waste material from its origin in the bone to the surface.

The wound is epithelialising around the wound edge and the epithelializing efforts continue lining the inside to protect the soft tissue from the harmful effects of the drained debris. The white matter is still-to-mature epithelium.

Yellow arrows: the exits of the 3 fistulas.

8

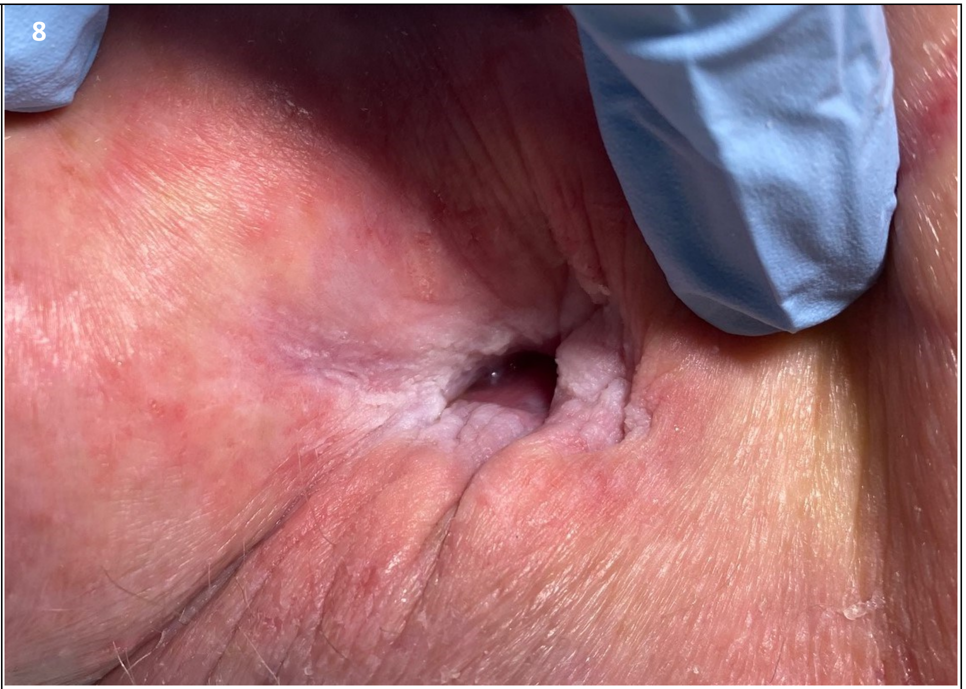

10 days after PHMB-betaine & silver application had caused skin and soft tissue breakdown.
MPPT used since.

10 months (305 days) after first MPPT

The white, constantly epithelialising border of the skin around the opening and lining the inside of the opening has considerably reduced, but is still existing.

The skin was widely affected. It is still a bit inflamed, but recovering.

The texture of the soft tissue is flaccid compared to the day before PHMB-betaine and silver were applied directly to the healthy, granulating soft tissue.

Wound number 37			Patient	SCI	
**Grade 4**	**3¾ months old**	**Ankle**	**51-year-old male**	**30 years**	**C3, C6, C8 incomplete & T1 complete**

Three and a half months earlier, a pinprick sized sore, barely noticeable, had appeared from out of nowhere with no explanation to its appearance. As it started growing bigger it was cleaned with saline; applied a povidone-iodine sheet with a polyethylene glycol base for slow release (Inadine), and dressed with an occlusive polyurethane foam dressing (Biatain Adhesive) on top. When it was 1.5 months old, it was treated with a course of systemic antibiotics (Flucloxacillin). Again 1.5 months later, another course of systemic antibiotics (Ciprofloxacin) was given to remove a *Pseudomonas* infection. At this point in time the dressing regime was also changed to two weeks of topical antibacterial silver sulfadiazine cream (Flamazine). After this, the dressing regime was for a brief period of time changed back to the original regime of saline, Inadine, and occluding foam, before changing to MPPT.

At the start of MPPT treatment, the wound generally contained deeply embedded slough but, most importantly, its central part was holding a "plug" of exceptionally white and tough slough of unknown depth (pic 1 & 2). The surrounding skin was affected, signalling a worsening (pic 1). After a few days, the skin was healed, and the wound bed was cleared of slough and granulating (pic 3). The central plug of viscous, inaccessible slough was also gradually removed via autolytic debridement (pic 4 & 5) and what seemed to be the bottom of the tunnel, that had been holding the slough-plug, was fully cleared and granulating (pic 6). Unfortunately, the bottom of this tunnel was demarcated by a clear oval gorge and had a distinct gorge running across it, as well (pic 5, 6 & 7). This is often a sign, that the tunnel continues its path far deeper than what is visible with the naked eye, or than can be probed, i.e. that it runs in the narrow interstitial space. This, again, is usually a sign that a primary infection, in a structure not pertaining to the soft tissue, e.g. in the bone, is using the tunnel to drain the debris it produces. Osteomyelitis can originate in the bone (haematogenous) and its debris will cause a draining fistula which sooner or later will manifest as a wound, often appearing as barely noticeable and appearing "out of nowhere". Or, osteomyelitis can be caused contiguously by an infected wound – once this happens, the condition changes into a primary infection requiring surgery and the associated wound changes into a secondary condition, as it serves as a draining fistula unable to close until the primary condition has been solved surgically.

Also, the tunnel continued to granulate and reduce its internal diameter; and epithelisation continued to reduce the overall size of the wound. (pic 7 – 10). Despite these two very positive developments, it was a concern that the granulation tissue, which was nicely filling up the former tunnel of the plug, continued to display a slightly darker colour than the rest of the granulation tissue. (pic 9 & 10). This could be a sign of infiltration by, what could look like low amounts of infectious debris from a primary source of infection feeding into the interstitial space of the novel tissue. On Day 94 (3 months into the MPPT treatment) the patient started a course of antibiotics for a urinary tract infection (UTI). It stalled the healing temporarily but otherwise did not impact the wound considerably (pic 11). Once the course was finished, the wound continued closing eventlessly for the following 2 months (pic12 & 13), until the patient contracted another UTI that severely affected many areas of his body before being diagnosed. A 7-day course of antibiotics (Nitrofurantoin) was prescribed (Day 158) (pic 14). The impact on the wound was very clear and in line with the fact that the immune system withdraws its support from wound healing when a different condition requires its attention, i.e. it downgrades wounds, until the more urgent matter has been resolved (Crane et al., 2021). The wound bed changed, practically overnight, from healthily granulating into being covered with characteristic dark-red/purple dots (pic 14).

This time, the UTI and, in particular, the antibiotics had a severe impact on the wound. Whereas it continued to heal and close (pic 15 – 18), the microbial composition seemed changed, now being more virulent, which would be consistent with the systemic antibiotics. The phenomenon seen as dark-red dots in the wound bed are usually reflected in the skin as dark red oval markings or even dark red streaks. If the wound clears these streaks, it will proceed to stable closure.

If, however, they do not disappear, it is usually a sign that debris coming from an underlying infection, such as osteomyelitis, is reinfecting the soft tissue from beneath, and any closure will be unstable.

As long as the infectious debris created in the bone is of an amount small enough for the immune system to be able to repair the soft tissue damage it continuously causes, the wound will remain closed. However, a bone infection spreads continuously, albeit slowly, and will, at some point, have grown large enough to produce more infectious, harmful debris than the immune system is able to deal with whilst, at the same time, repairing the damage at the speed it is caused. When this threshold is reached, the wound will re-open, and the immune system will change its strategy and aim to create a fistula between the bone and the skin surface through which it can transport the infectious, corrosive debris to the surface whilst causing as little damage as possible on its way through. The osteomyelitis will, in itself, pose a risk of sepsis to the patient. Once it is more than 6 weeks old, this can only be removed surgically. It can, however, during periods of bacteraemia or septicaemia be controlled with systemic antibiotics. The infection that the osteomyelitis causes in the soft tissue, i.e. the wound, poses a second risk of sepsis. Antibiotics do not work in wounds. The mitigation of this second risk, therefore, correlates directly with the effectiveness with which the soft tissue is kept free of infection - first while the draining fistula is being established, and subsequently maintained without infecting the surrounding skin and tissue along the fistula until surgery for the osteomyelitis can be performed.

This wound progressed to full closure (pic 18) and remained closed for two and a half months. During this period the dark-red streaks did not disappear (pic 18 – 22). Instead, on day 341 a dark-red bacterial migration appeared in the skin surface (pic 23) and was the sign that the mentioned threshold had been reached. For the following month, the wound fought resolutely against several of these microbial attempts to spread, infiltrating the skin and breaking down the soft tissue in all directions (pic 23 – 28). After that month, the immune system had controlled the attempts to spread, and had established a draining fistula causing as limited damage to the skin and soft tissue lining and surrounding this tunnel between the bone and the skin, as possible. The establishment of this fistula allowed the body to prevent any attempts to cause cellulitis or soft-tissue infiltration (pic 29 - 32). An x-ray of the ankle, to try to determine the location of the osteomyelitis, showed mild degenerative changes of the ankle mortise and subtalar joints. Osteopenia is one of the characteristics of osteomyelitis in x-rays (Giurato et al., 2017), and the patient was referred for further investigations for osteomyelitis. Whilst awaiting further action on the primary source of infection, the fistula was cemented further and maintained in a controlled manner without darkness at the bottom of the opening (pic 30 – 32). Also, at no point was the skin dark-red, warm or swollen (pic 23 – 32). Both features - inflamed skin or darkened wound bed/fistula interior - would have signalled an elevated risk of sepsis.

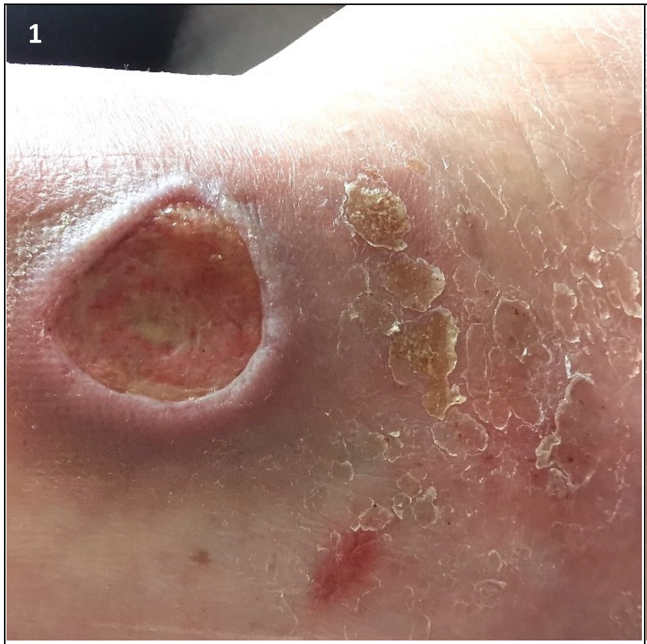	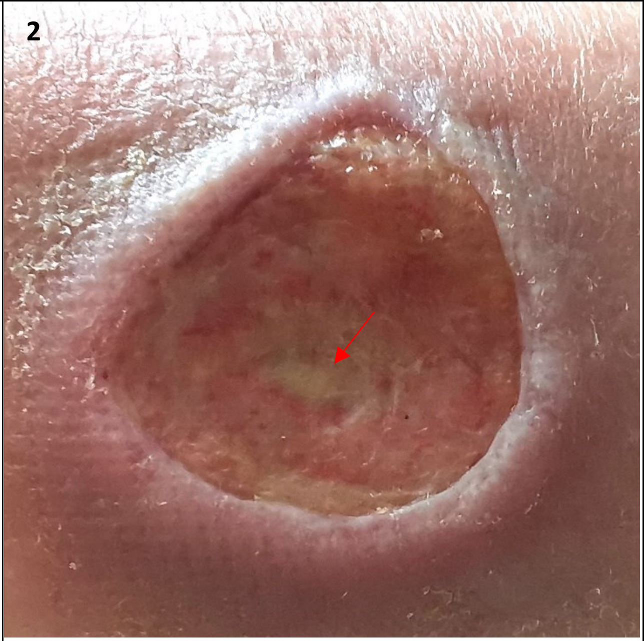	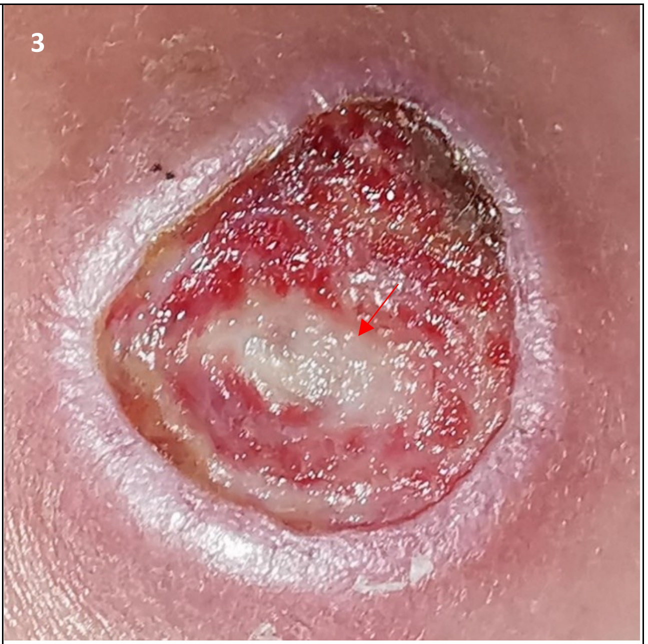
**Day 0**	**Day 0**	**Day 2**
The distally surrounding skin is cracked in a 4 cm wide band. It is covered with scaly scabs in an effort to heal the cracks.<br>A new wound is opening approximately 2 cm from the original wound. (5 o’clock)	23mm in diameter.<br>The wound bed contains lots of dried out, tough, deeply embedded slough. The wound edges are intact but of a dull or lifeless appearance.<br>*Red arrow*: Characteristic plug of viscous slough.	Granulation is budding all over the wound bed whilst the embedded slough is being exposed and removed. The tough, viscous plug of slough of unknown depth in the centre of the wound is also being gradually removed. The wound edges are shiny pink-white and the inner border is epitelialising.<br>*Red arrow*: Plug of viscous slough

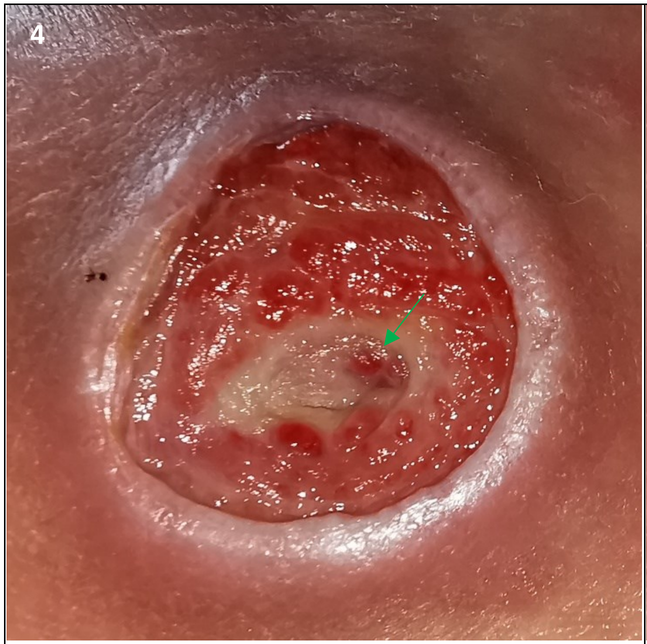

Day 7

The slough in wound be is practically removed and granulation is dominating. The central plug of tough slough is disappearing, and granulation is showing though in one place at the bottom. The wound is epithelializing well around all the wound edges.

Green arrow: Granulation at the bottom of the tunnel that was holding the viscous plug of slough.

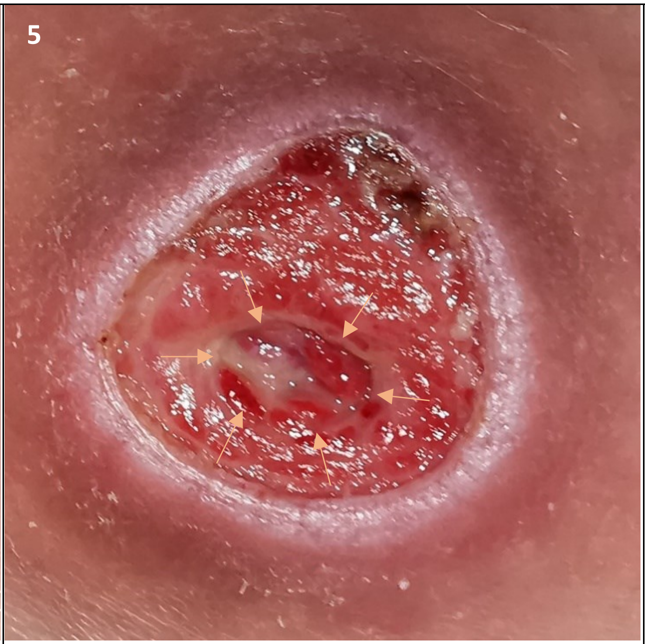

Day 12

20mm. in diameter

Orange arrows: Oval gorge

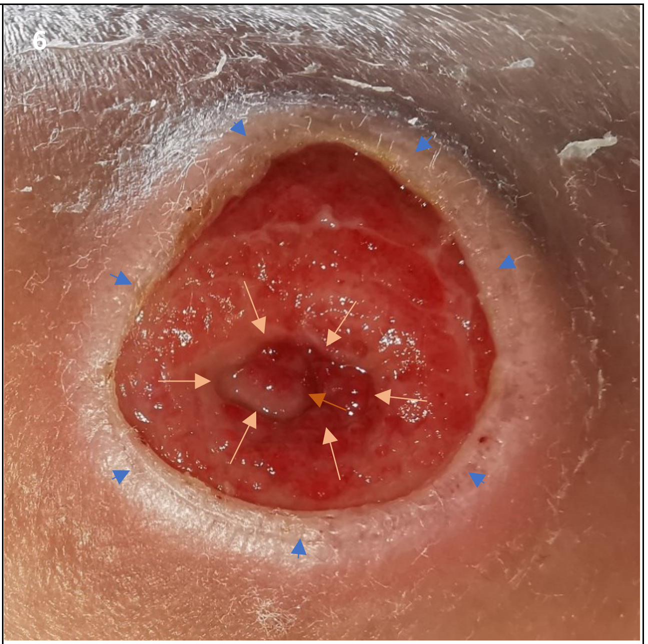

Day 19

The plug has been definitively removed. The tunnel, that it was plugging, is fully granulating but structurally still distinctly visible. A circular gorge is marking the visible bottom of the tunnel and a gorge is cutting through this bottom.

The new epithelium is maturing at the speed it is formed. This phenomenon is visible in the ring formation of the epithelium around all the wound edges. The arrows only point to examples of it.

Orange arrows: Oval gorge with a gorge running across it.

Blue arrows: Newly formed epithelium as it matures in rings according to its age.

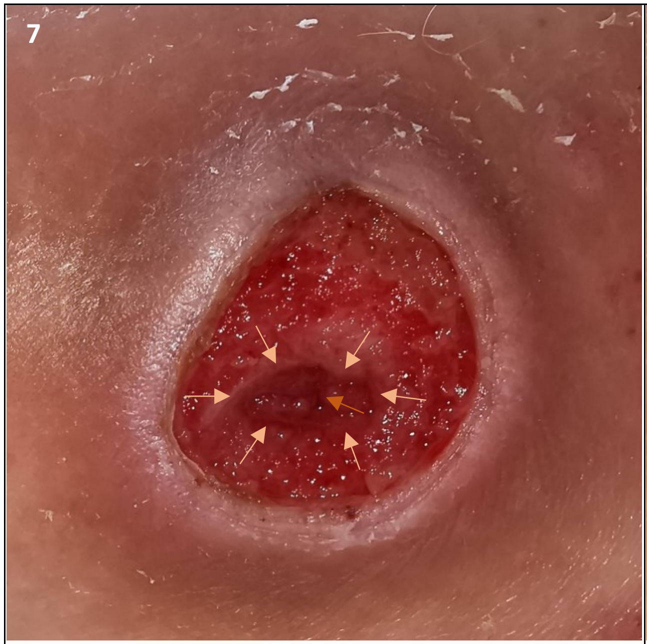	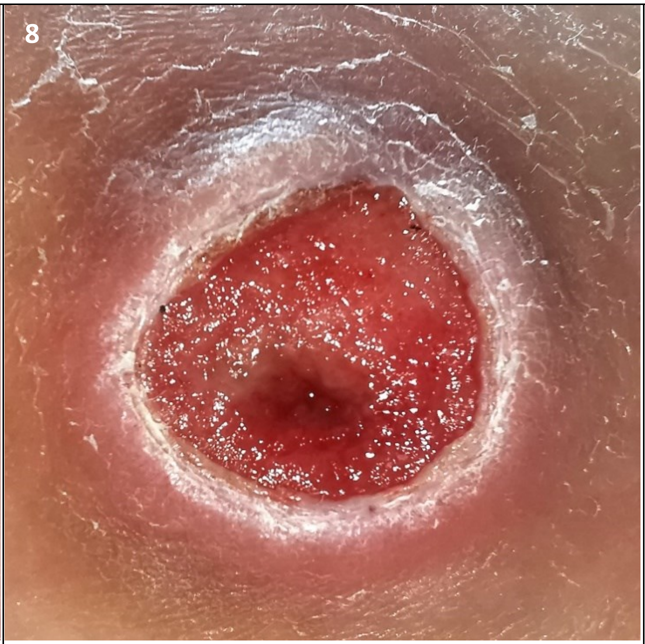	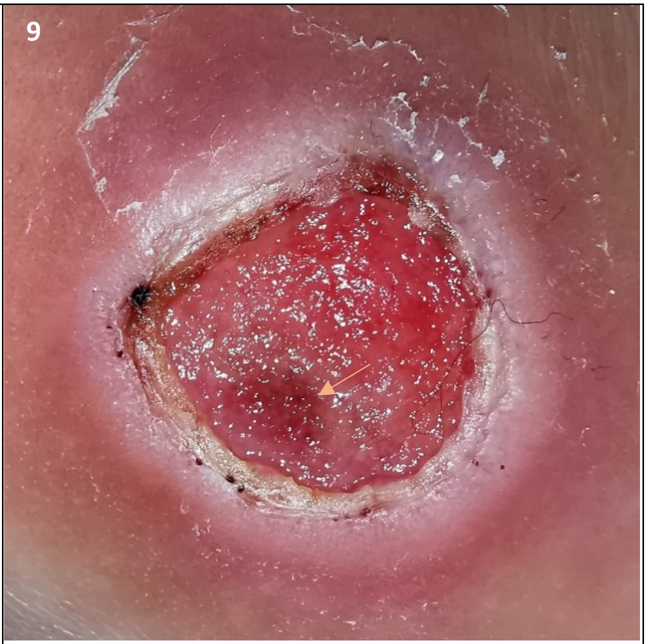
**Day 24**<br>17 x 13 mm.<br>*Orange arrows*: Oval gorge with a gorge running across it.	**Day 37 1¼ months**<br>As the wound area is rapidly reducing, the tunnel is also filling up with new granulation, reducing in diameter.	**Day 68 2¼ months**<br>*Orange arrow:* The granulation tissue is of a slightly darker colour in the area of the former tunnel formation.

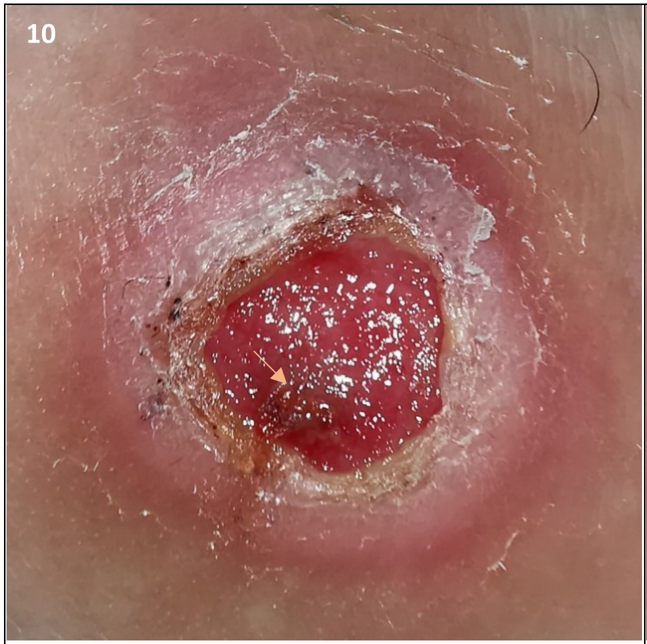

Day 89 3 months

11 mm in diameter.

Orange arrow: The granulation tissue is still slightly darker at the former tunnel formation.

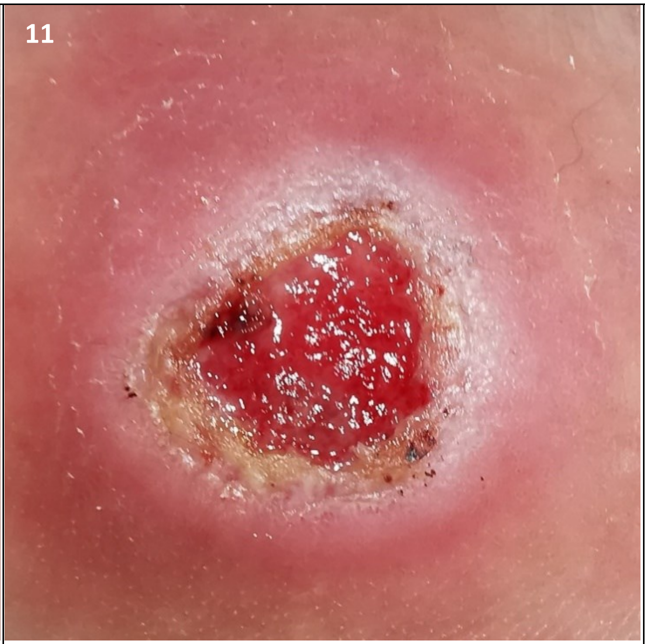

Day 108 3½ months

The granulation and epithelium are holding up well, despite the course of systemic antibiotics finished a week earlier due to an UTI.

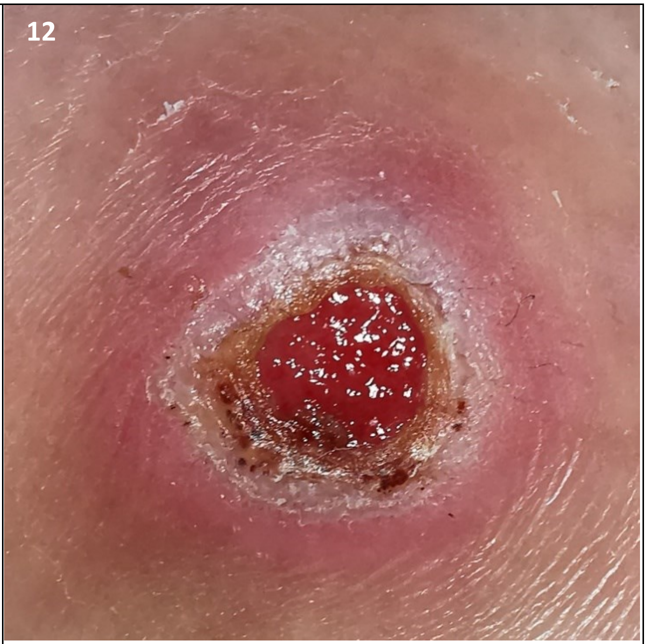

Day 125 4 months

10 mm in diameter.

Granulating, and strong epithelialisation along edges.

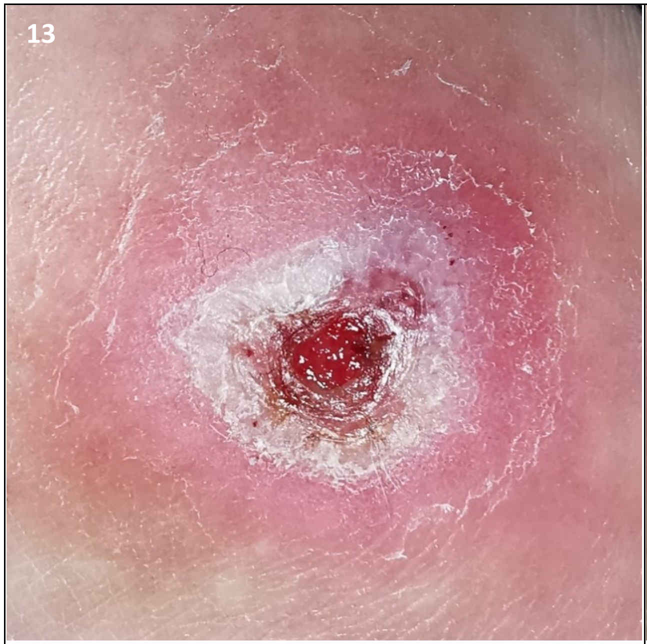

Day 152 5 months

4 mm in diameter.

Granulating, and strong epithelialisation along edges.

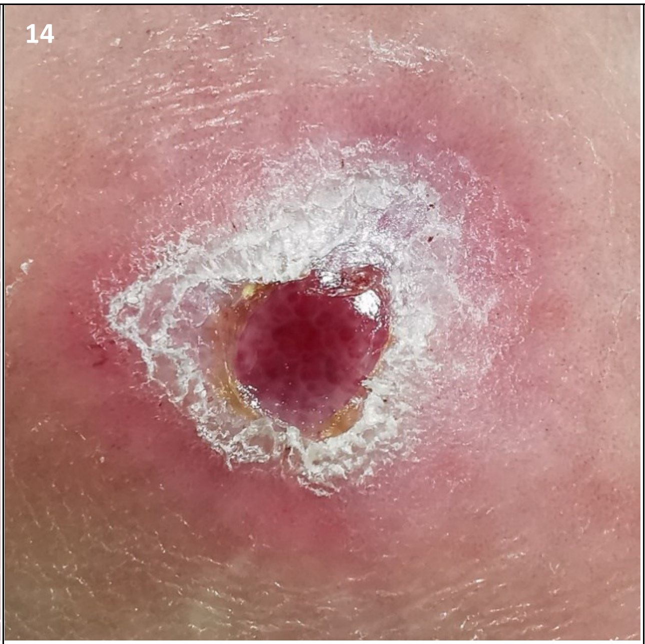

Day 158 5¼ months

A UTI claims the attention of the immune system – away from the wound.

The wound bed is covered with characteristic dark-red/purple dots.

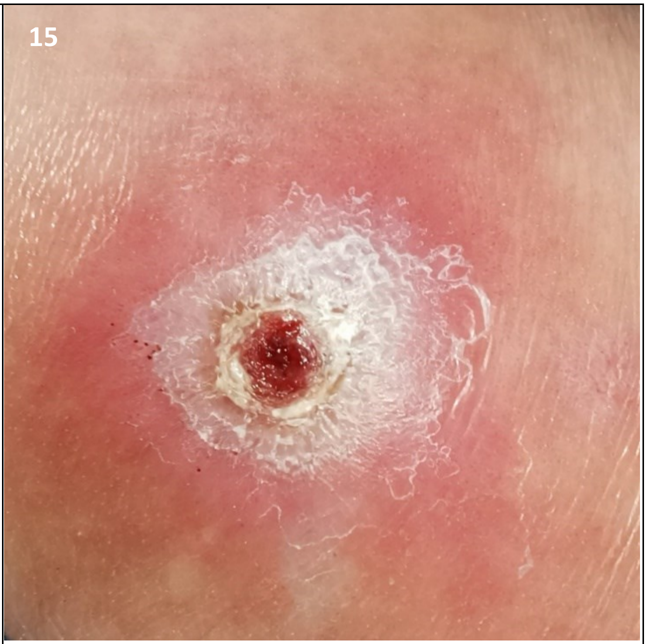

Day 169 5½ months

7 mm in diameter.

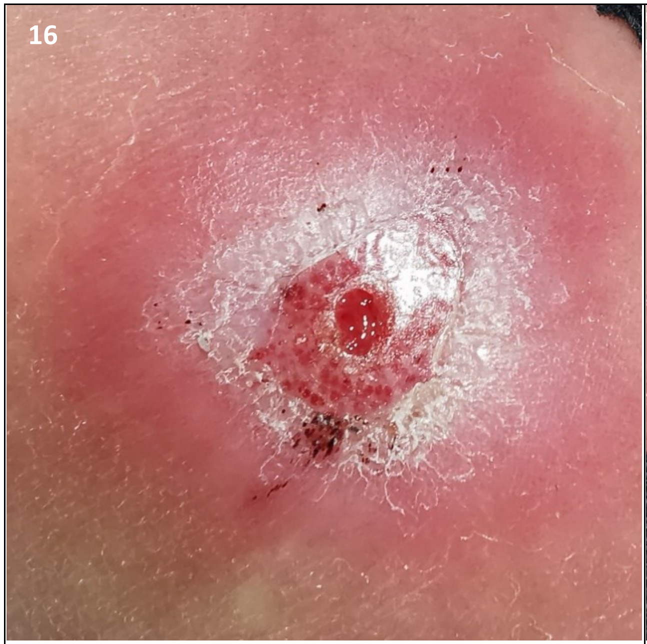

Day 175 5½ months

The wound continues to close, but dark-red/purple dots still act up occasionally. Here, as dark dots in the delicate new epithelium.

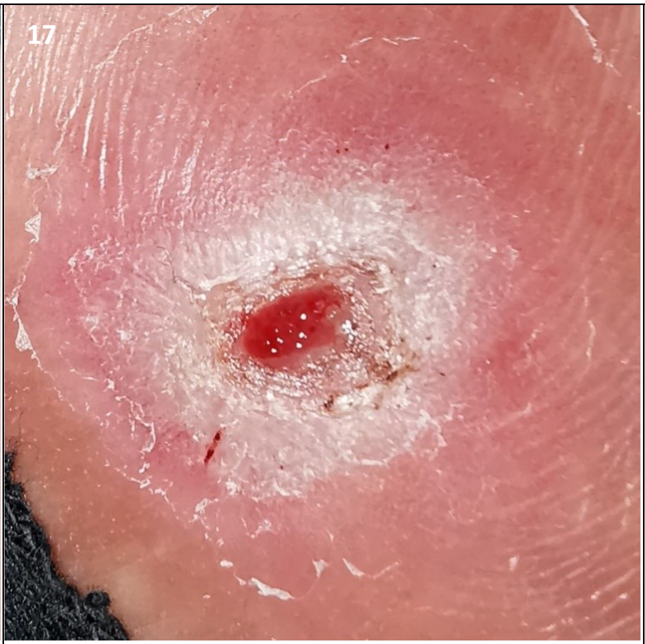

Day 217 7 months

3 x 5 mm.

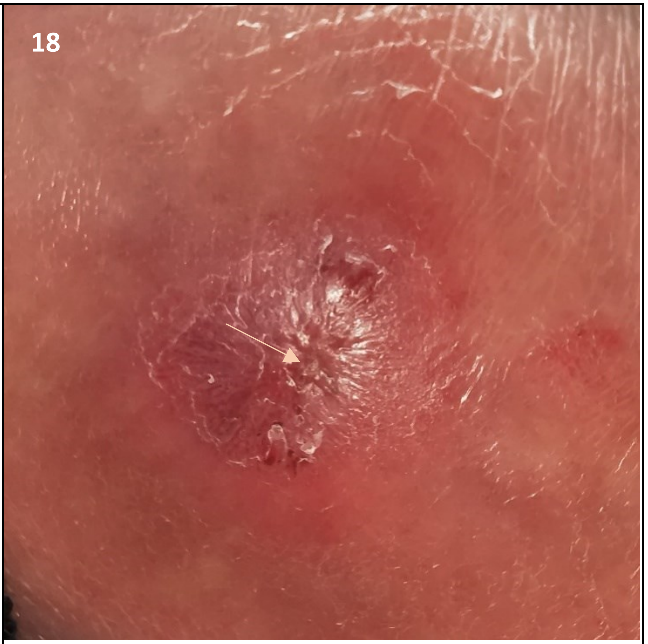

Day 259 8½ months

The wound is closed.

There are dark-red streaks in the new skin. They radiate out from a central point.

No signs of inflammation.

Orange arrow: central point

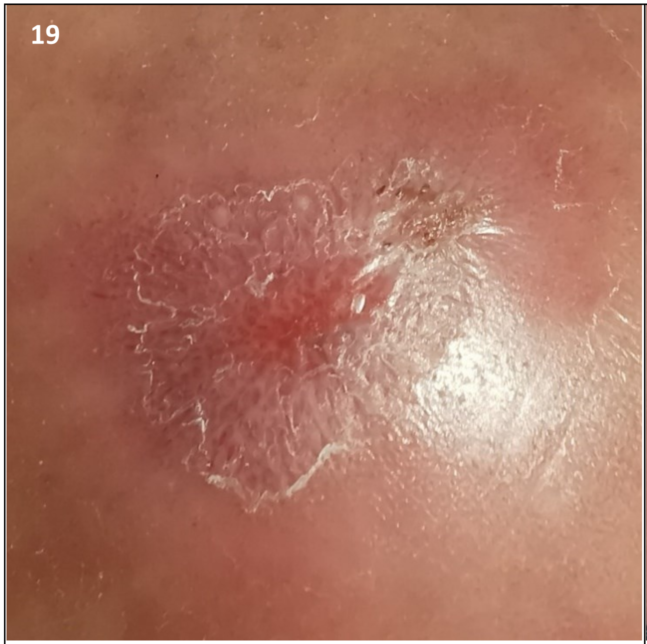

Day 268 8¾ months

The wound remains closed.

The dark-red streaks continue to be present.

No signs of inflammation.

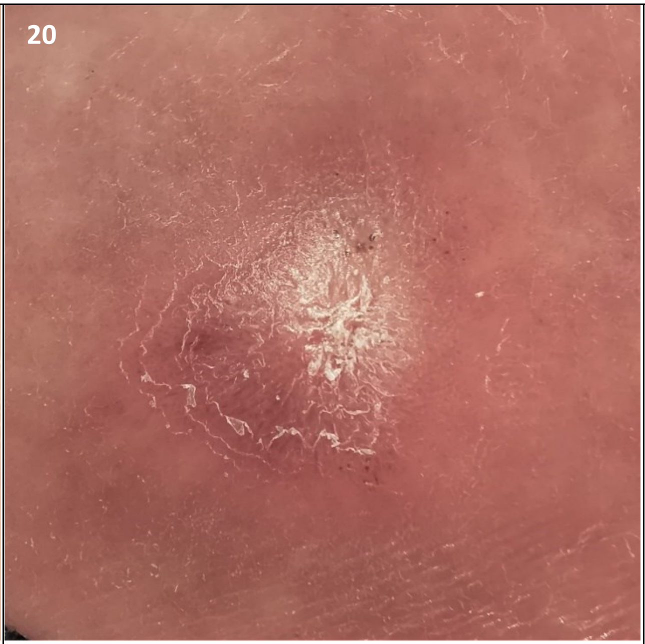

Day 295 9¾ months

The wound remains closed.

The dark-red streaks continue to be present.

No signs of inflammation.

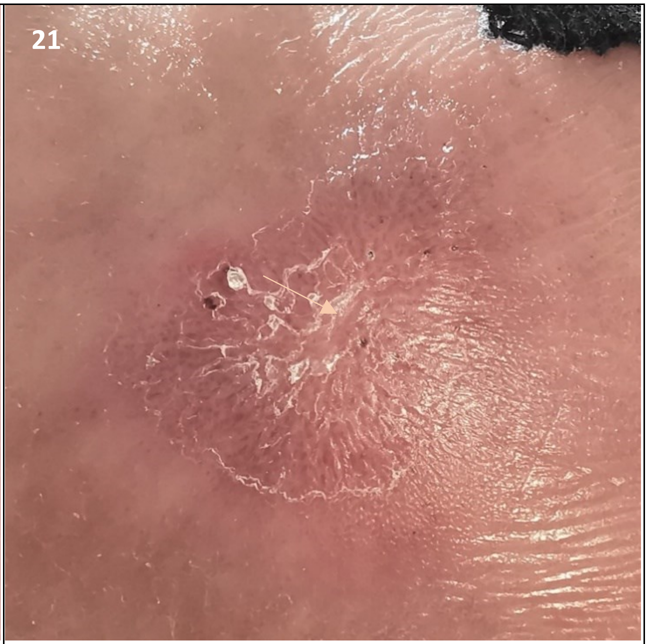

Day 319 10½ months

The wound remains closed.

The dark-red streaks continue to be present.

No signs of inflammation.

Orange arrow: central point

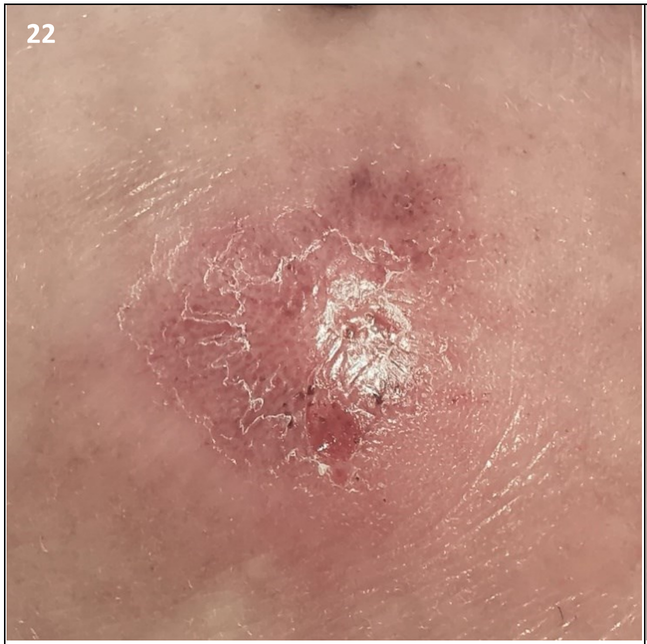

Day 339 11¼ months

The wound remains closed.

The dark-red streaks continue to be present.

No signs of inflammation.

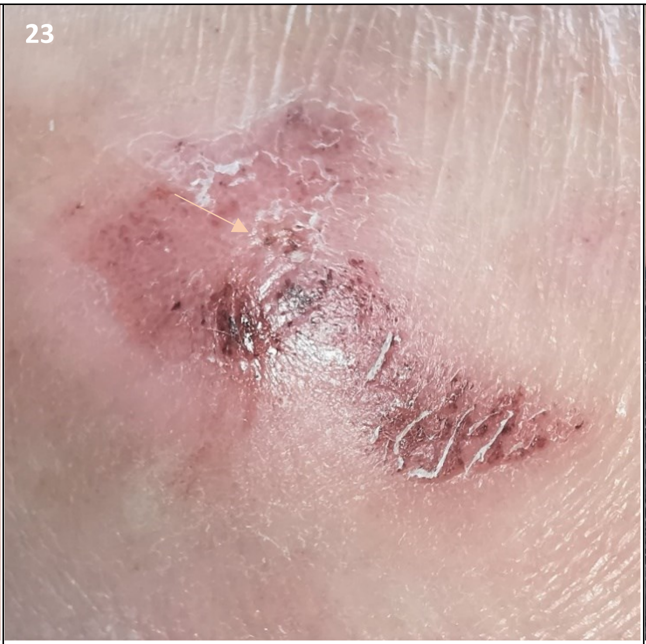

Day 341 11¼ months

Microbial migration in the skin - an intent of microbial spread shooting from the centre.

No signs of inflammation.

Orange arrow: central point

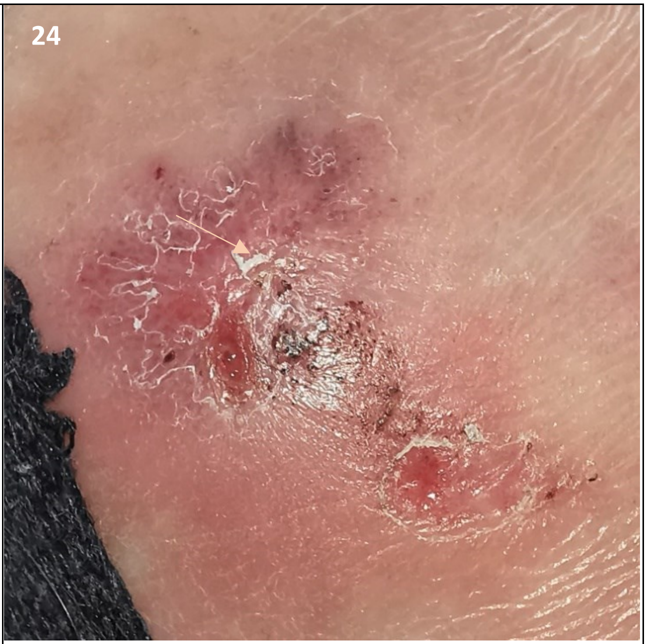

Day 345 11 1/3 months

The spread is contained, and the harm done to the skin is undergoing repair. The two openings are healthily granulating.

Orange arrow: central point

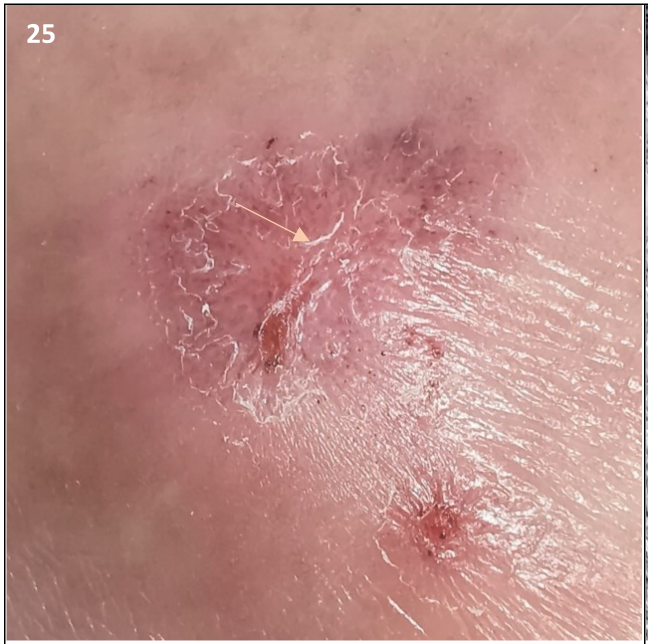

Day 349 11½ months

The infiltrated area and both openings are again fully controlled. The skin is repaired. No inflammation.

Orange arrow: central point

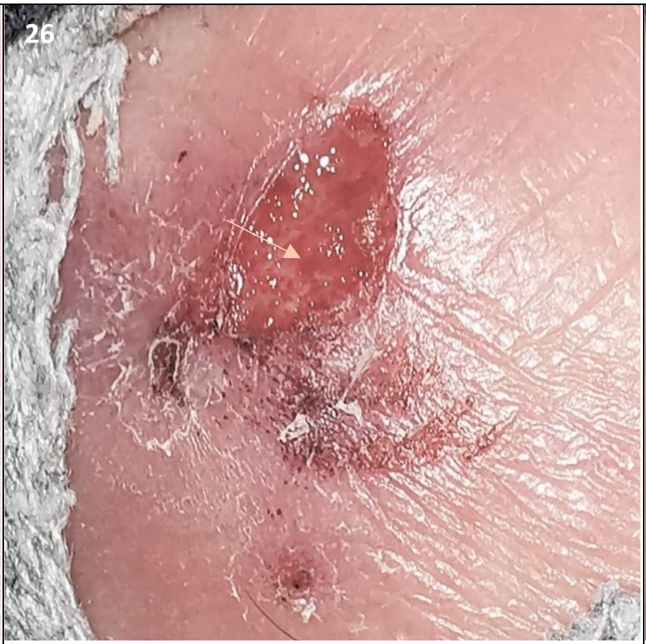

Day 351 11½ months

The microbial load from the underlying bone has definitively passed the threshold. The need to drain constantly is now evident. Also, a new attempt of migration and spread by the microbes has been launched.

Orange arrow: central point

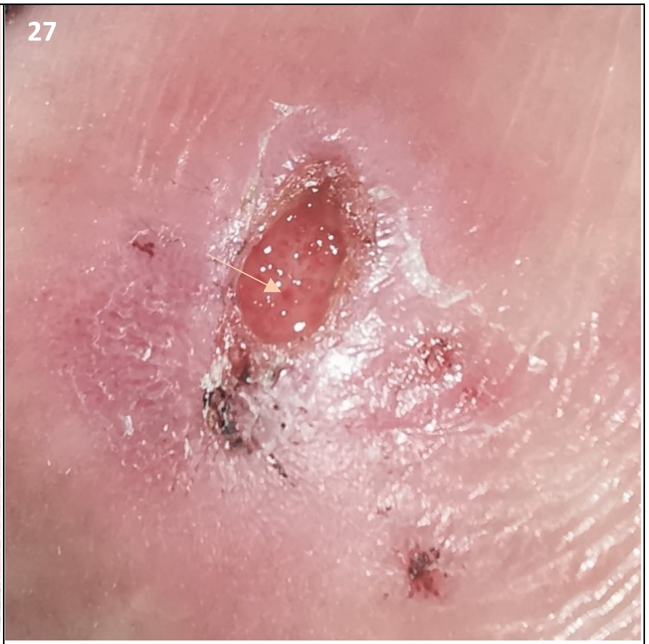

Day 357 11¾ months

The whole area is again under control. The openings are either granulating or practically closed. The bigger opening will form the start of the draining fistula that now needs to be firmly established.

Dark-red streaks are present in the skin along the lower right side (7-9 o'clock).

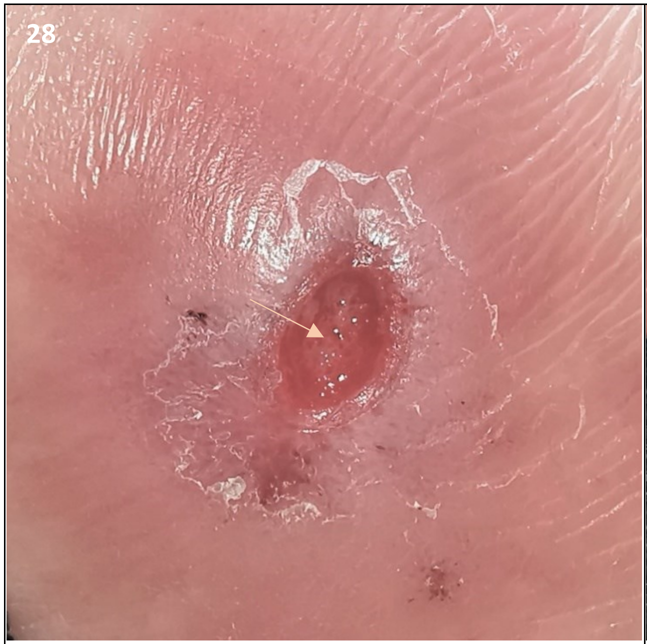

Day 362 12 months

The spread remains contained. The skin is being repaired. No signs of inflammation.

Orange arrow: central point

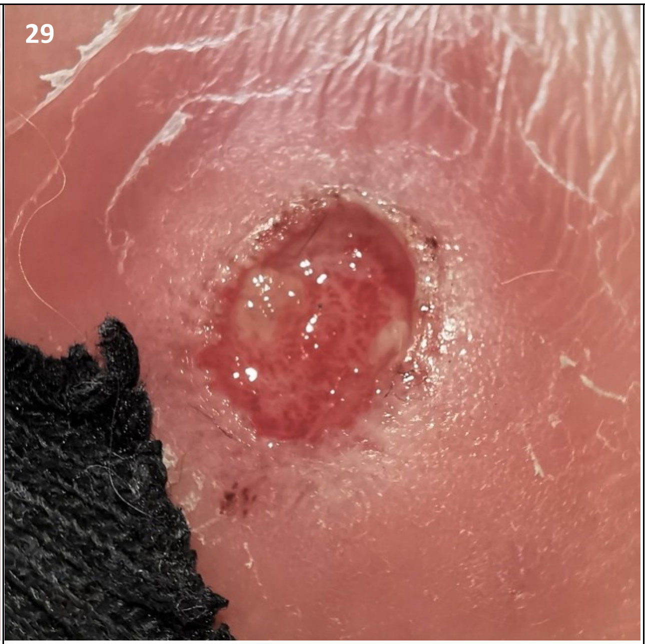

Day 381 1 year ½month

Close-up

10 mm in diameter

The opening of the draining fistula has been firmly established. The surrounding skin is healed, intact and with no signs of inflammation.

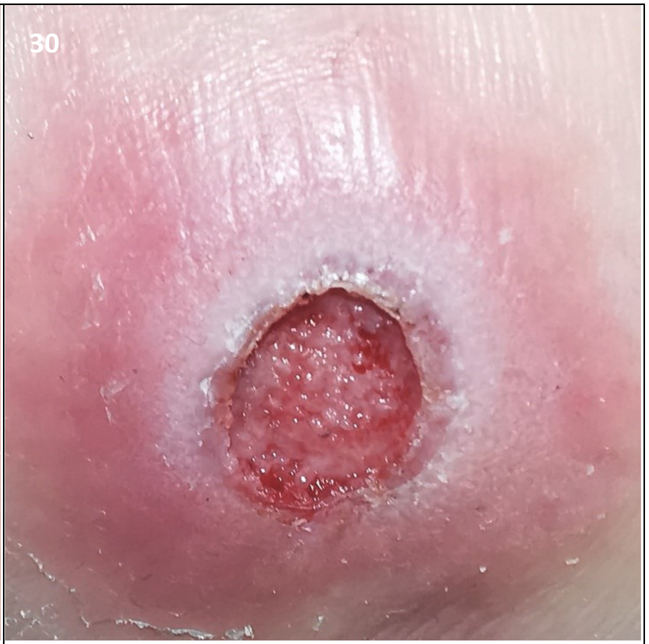

Day 393 1 year 1 month

10 mm in diameter.

The skin is healthy and unaffected.

No signs of inflammation.

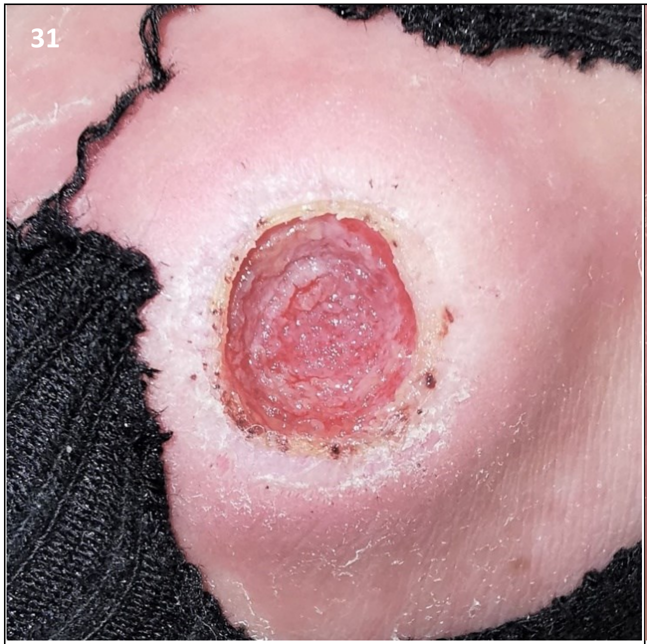

Day 532 1 year 5½ months

10 mm in diameter.

The skin is healthy and unaffected.

No signs of inflammation.

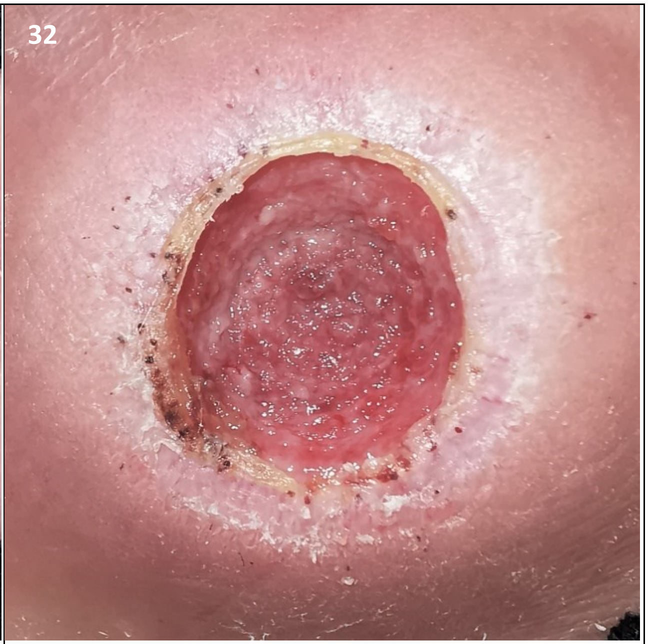

Day 533 1year 5½ months

Close-up

10 mm in diameter.

The wound is acting as a draining fistula for the osteomyelitis and for the past 5 months it has maintained the same narrow diameter, healthy surrounding skin and granulating wound bed / fistula interior. Also, it has displayed no signs of inflammation.

Wound number 38			Patient	SCI	
**Grade 4**	**5 years old**	**Gluteal fold**	**57-year-old male**	**37 years**	**C5/6 complete**

This sore was detected 5 years earlier as an abscess of unknown origin. The patient has practically been on bedrest for 5 years during which period the wound has been managed with Manuka honey (Medihoney) and Tegaderm foam adhesive dressing as well as treated with Sudocrem (antibacterial, antifungal and anaesthetic), and with Cavilon barrier cream (acrylate terpolymer film). The wound repeatedly seemed underway to healing but never succeeded. MPPT treatment was initiated because it was becoming increasingly difficult to keep the wound from deteriorating.

After MPPT treatment was started, the erythema resolved in a few days and the wound granulated to surface level very quickly and gradually closed. However, it would soon reopen to expel a concentrated mass of bright red toxins, whereafter it would gradually reclose. The wound would enter a pattern where it would readily open for expulsion of toxins and remain open for as long as needed. Between expulsions, it would close whenever possible, presumably as a measure of auto-protection. With every repetition of this pattern, the radius and intensity of the red-purple discolouration under the skin would reduce in size, indicating that the infection in the skin and soft tissue was gradually being drawn out. Possibly, every re-opening would correspond to the clearance of a small abscess containing red-pigmented toxins; this would be in agreement with the reduction in the extension of the discolouration of the tissue adjacent to the wound following every occurrence.
Furthermore, the patient could break the 5-year bedrest and progress the wound while leading an active life.

MPPT was used as a diagnostic tool. The wound area and adjacent skin were free of infection and healthy looking. Nevertheless, the wound kept repeating a cyclic pattern of reopening to expel infectious debris with lots of red-pigmented toxins onto the surface of the body for some days or weeks whereafter it would close. The time between each closure did not decrease and the period of time it remained closed each time did not increase. Both features, that would be expected if the wound were just taking a long time to reach stable closure as could be the case of a very old wound with no associated primary source of infection in non-soft tissue. Instead, the wound would reopen more frequently and remain closed for increasingly shorter periods of time. This led to the conclusion that it was not a wound as such but the exit area of a tract leading debris from an osteomyelitis in the bone to the surface. As an osteomyelitis expands over time, the amount of debris to be expelled through the tract will increase correspondingly and consequently, the periods the tissue can seem closed will shorten. The patient was consequently operated for osteomyelitis and removal of the tract. The recovery of the surgical site was swift and without complications, presumably because there was no infection in the soft tissue and the immune response in the area had been prepared ahead of the operation. The period of convalescence was accompanied by two in-hospital UTIs, prolonging in-hospital stay, and a third UTI immediately upon discharge.

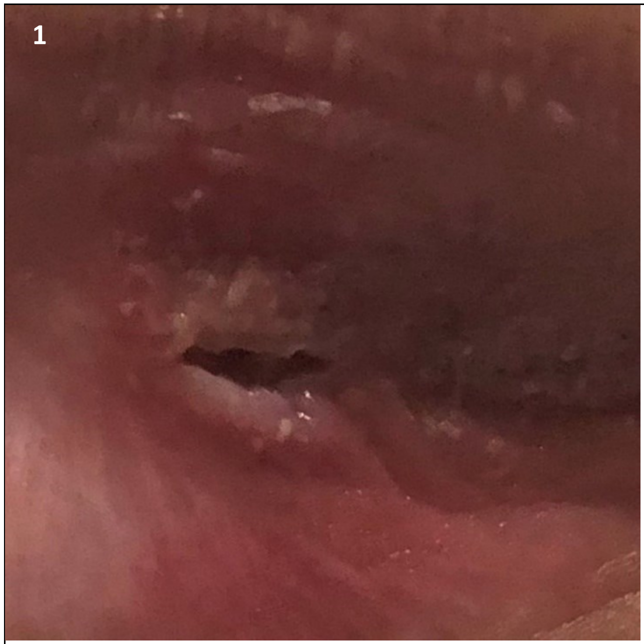	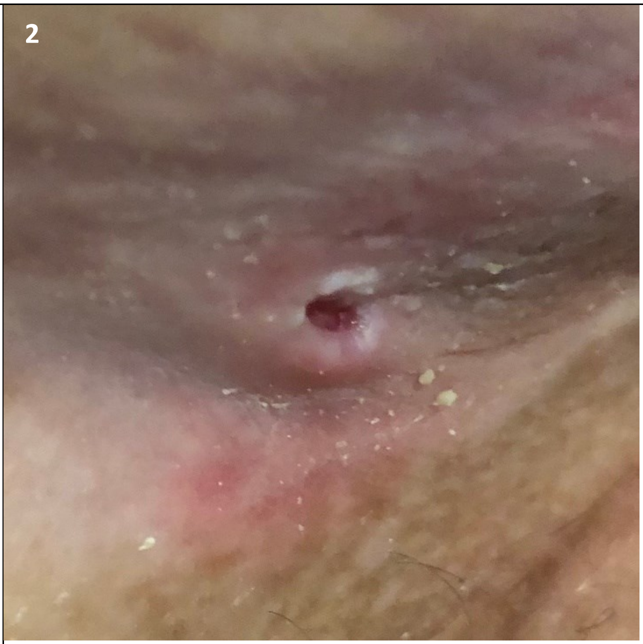	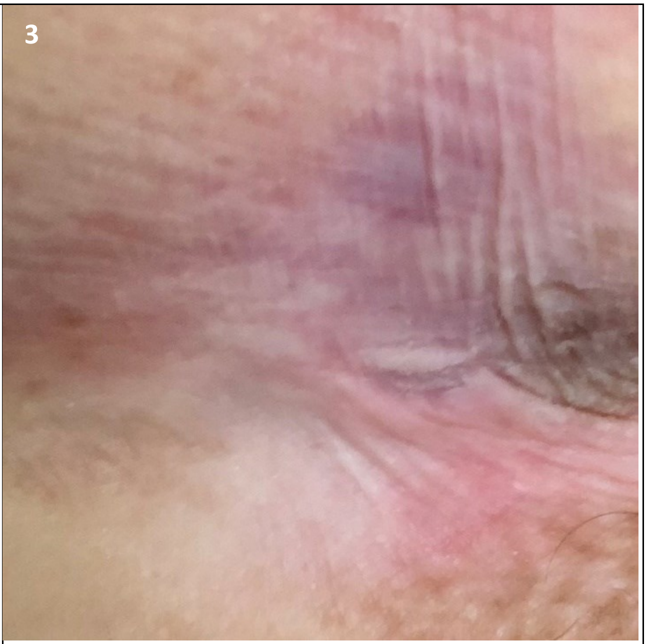
**Day minus-7**<br>**1 week before first MPPT**<br>Opening 10 x 3 mm; depth indeterminable<br>Highly exuding<br>Strong, deep erythema in a radius of between 40 and 60 mm of the opening.	**Day 31 1 month**<br>2 x 2 mm; depth 1 mm<br>Wound bed fully granulated to surface level.<br>Erythema gone.<br>Red-purple discolouring under the skin is gone in cranial-caudal axis but remains in lateral-lateral axis.	**Day 117 3.5 months**<br>Closed<br>The wound is closed but a red-purple discolouration under the skin remains in the lateral axis, i.e. vertically in the photo.

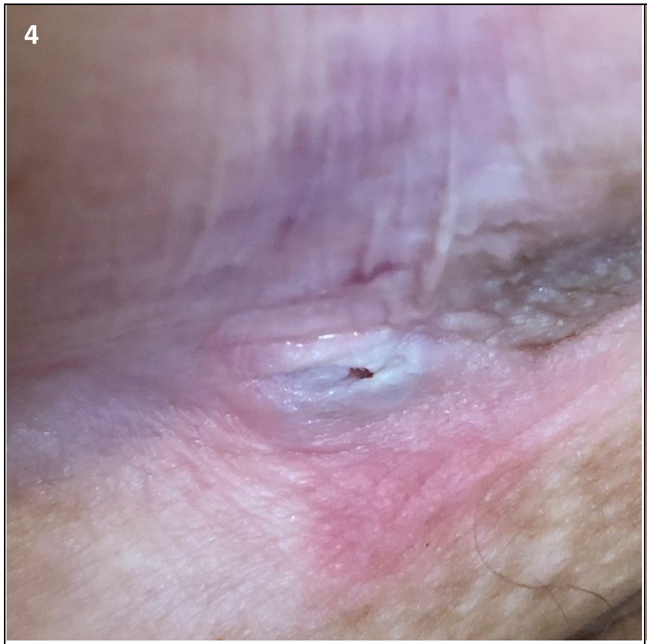	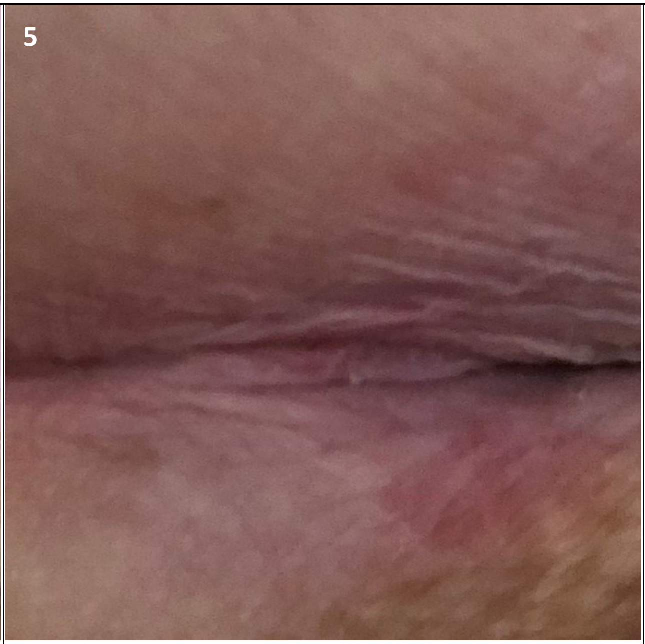	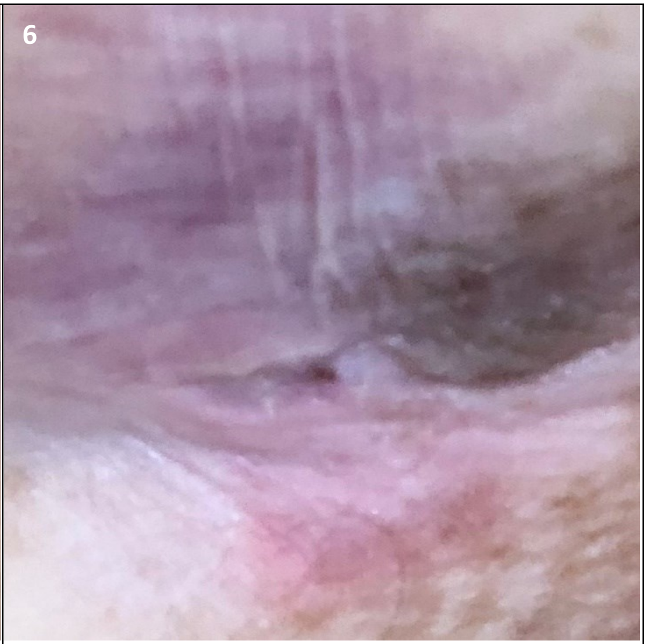
**Day 130 4 months**<br>1 x 1 mm<br>Opens and expels a cargo of red toxins.<br>The red-purple discolouration under the skin and along the edges of the opening shows accumulation of red toxins prior to and during a phase of expulsion.	**Day 147 4.5 months**<br>Closed<br>The wound has closed again and the red-purple area under the skin is reduced in size but still present.	**Day 157 5 months**<br>0.1 x 0.1 mm

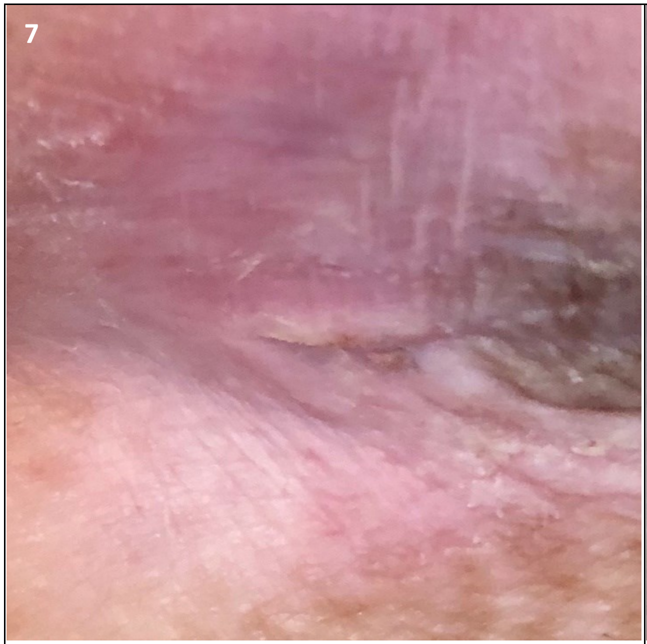

Day 158 **5 months**

Closed

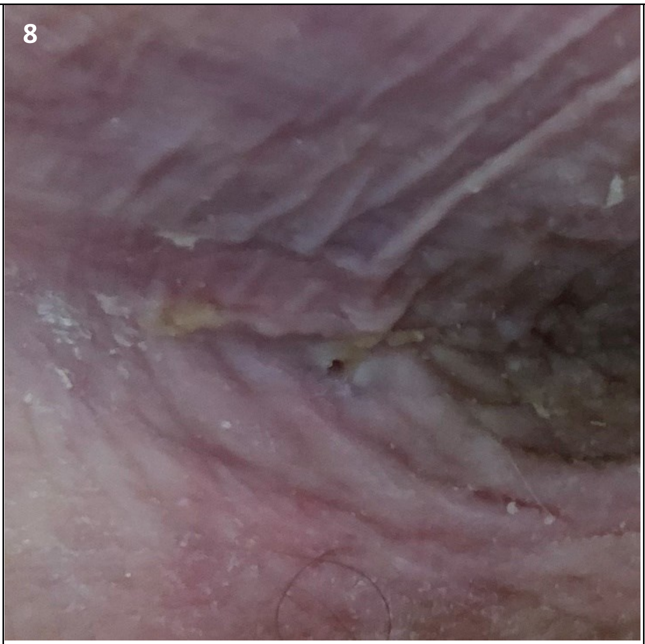

Day 166 **5.5 months**

0.5 x 0.5 mm

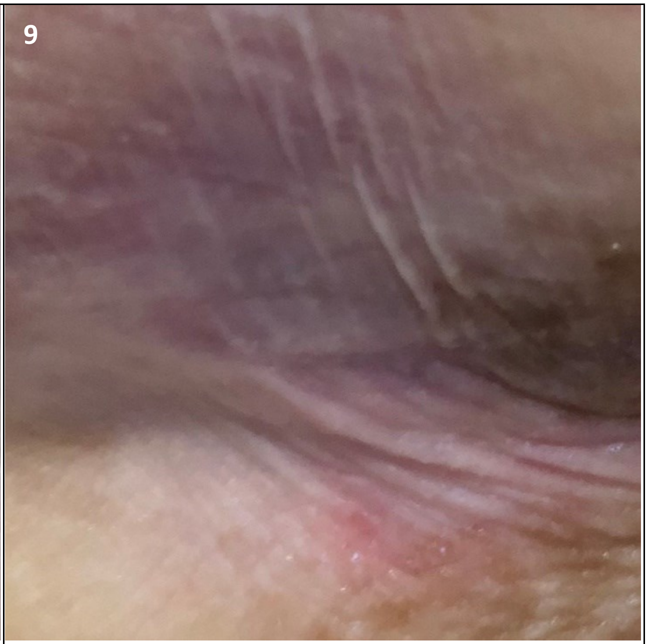

Day 184 **6 months**

Closed

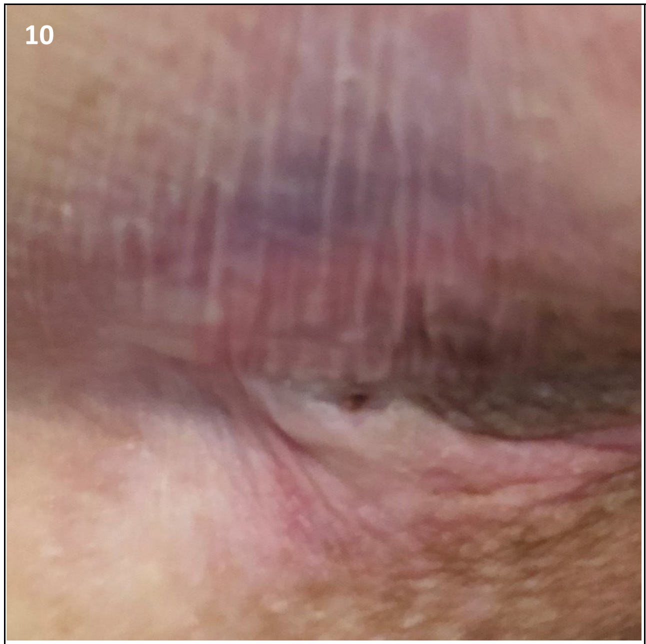

Day 203 6.5 months

0.5 x 0.5 mm

There is a red-purple discolouration under the skin above the opening. This is an ongoing assemblage of red-pigmented toxins that will be moved towards and expelled through the wound opening.

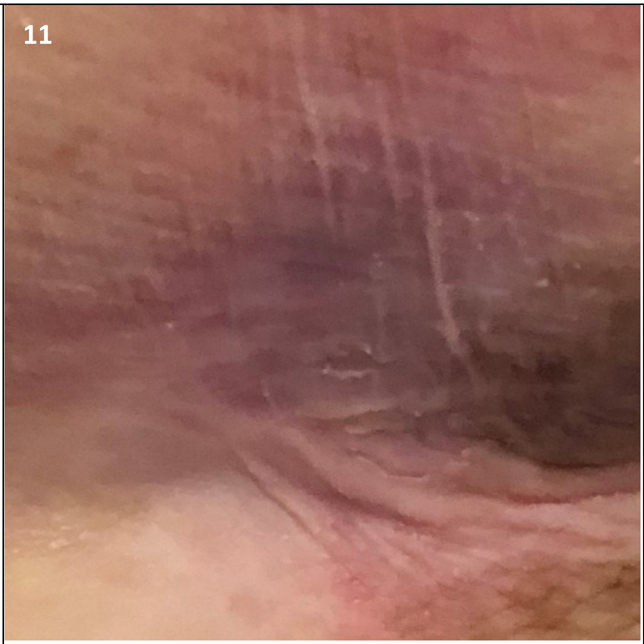

Day 212 7 months

Closed

The skin appears slightly rough and the purple discolouration under the skin above the area of the wound shows that the collection of red-pigmented toxins has advanced but is still ongoing and that they are being prepared for expulsion.

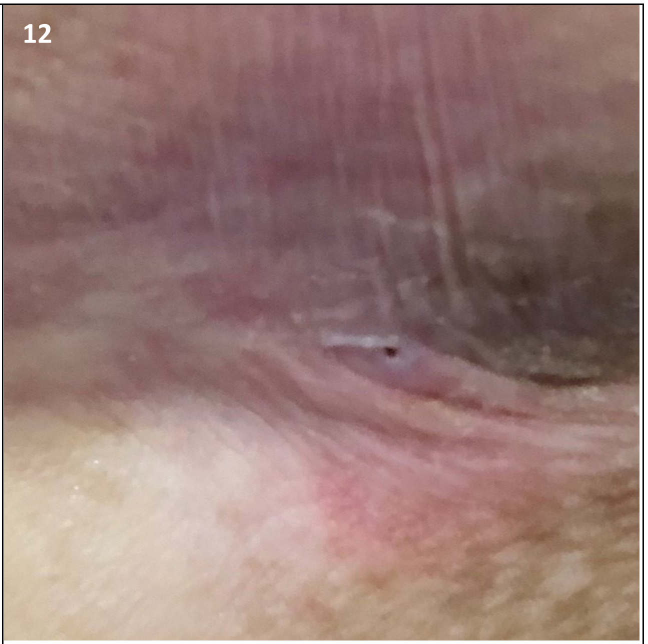

Day 226 7.5 months

0.2 x 0.2 mm

Skin opens to expel red toxins. The red-purple discolouration under the skin continues to reduce in size and intensity.

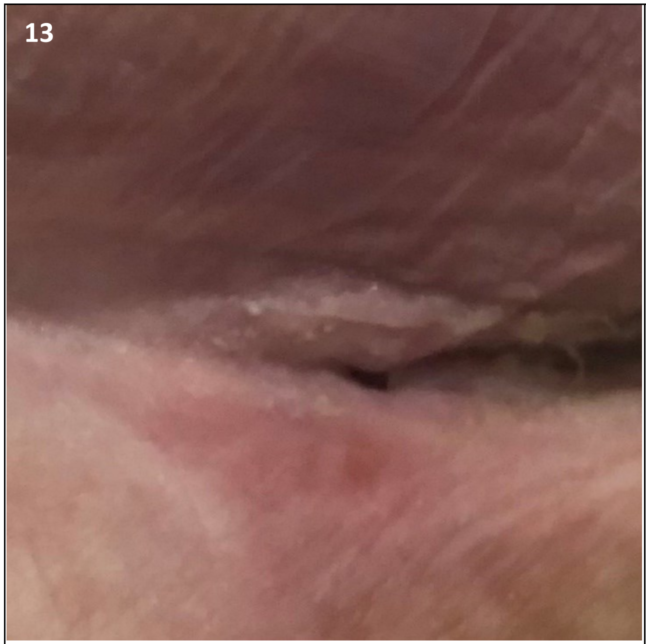

Day 307 10 months

0.5 x 1 mm

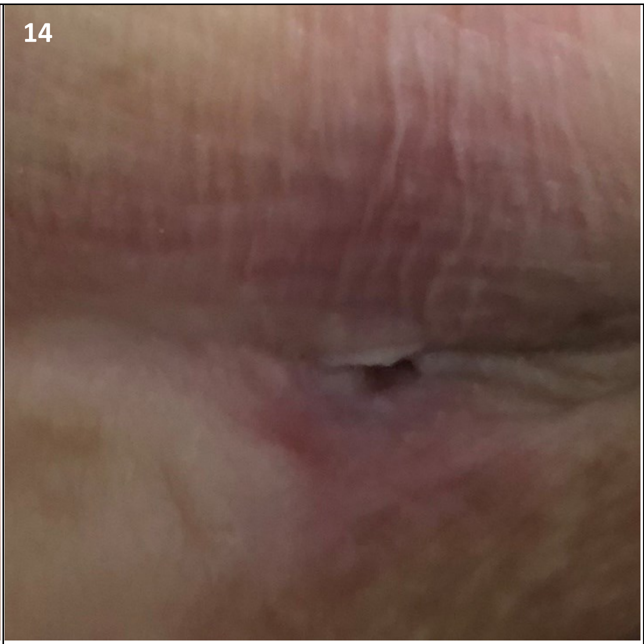

Day 364 12 months

0.5 x 1 mm

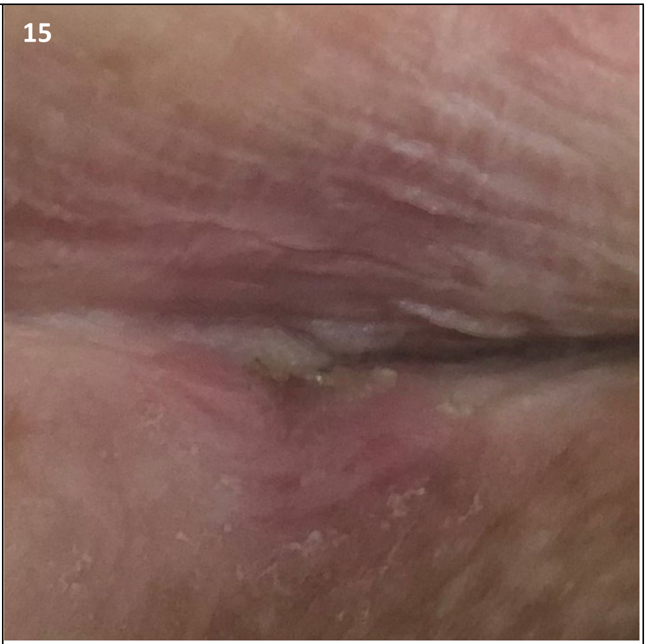

Day 416 13.5 months

Closed

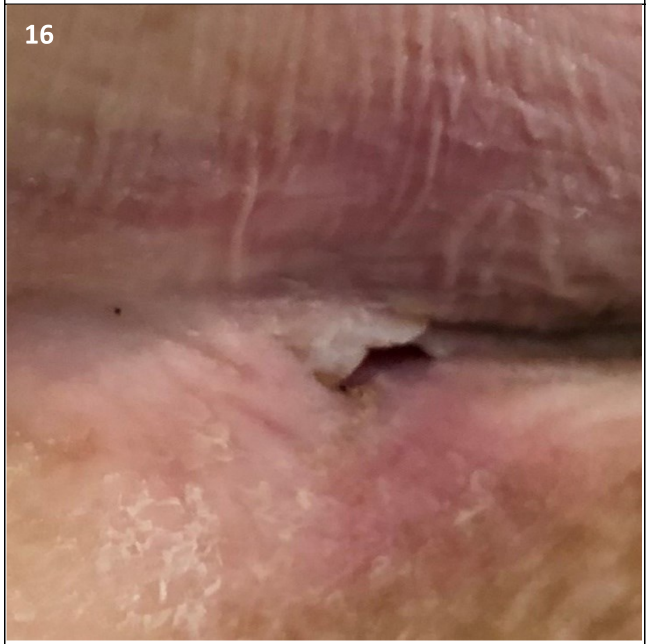

Day 418 13.5 months

1 x 3 mm

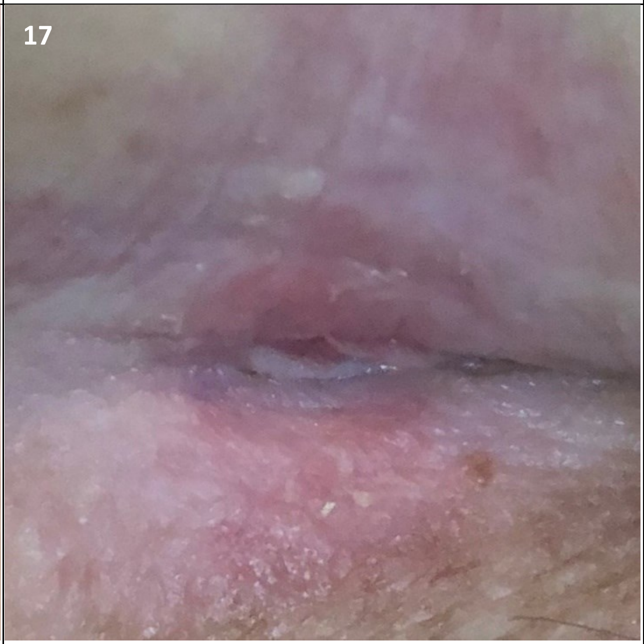

Day 479 15.5 months

Closed

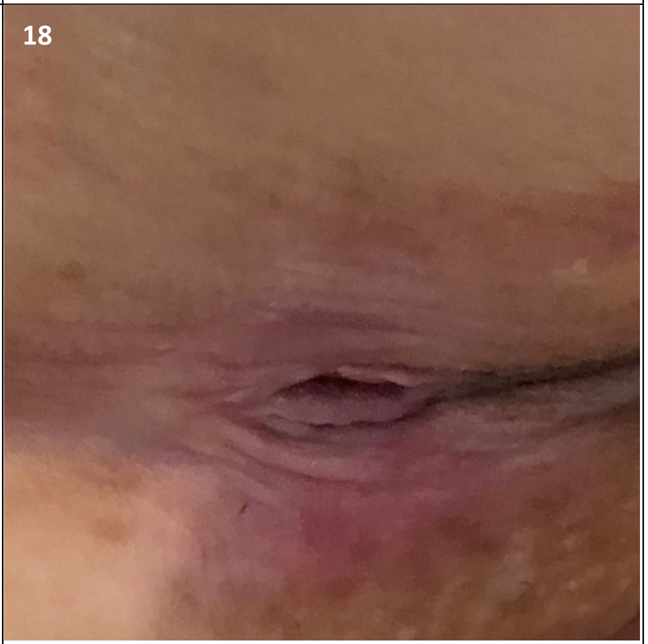

Day 493 16 months

4 x 1 mm

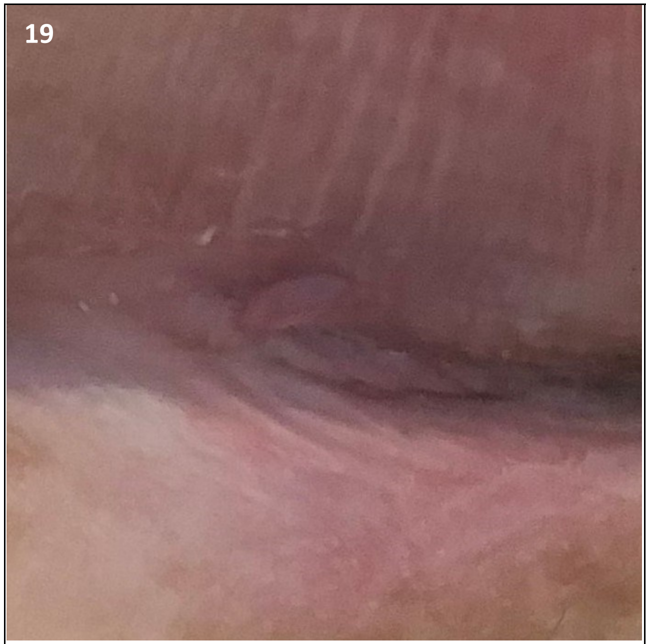

Day 526 17 months

Closed

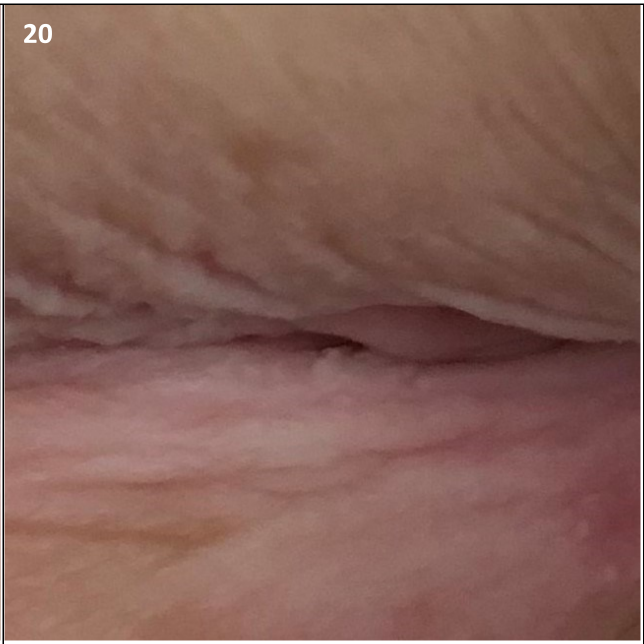

Day 556 18.5 months

10 days before osteomyelitis surgery performed on day 566

0.5 x 3 mm

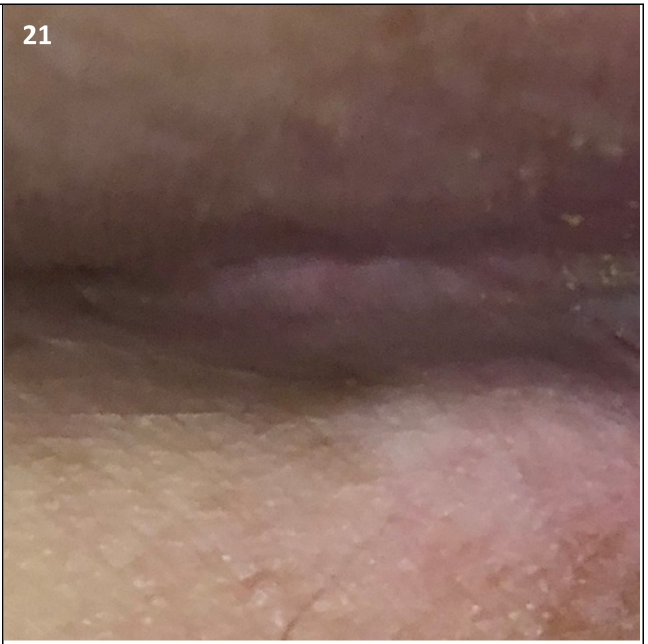

Day 644 21 months

2.5 months (78 days) after osteomyelitis surgery

Wound and surrounding skin well healed and patient mobile.

Wound number 39			Patient	SCI	
**Grade 4**	**9½ weeks old**	**Ischial tuberosity**	**76-year-old male**	**55 years**	**T12 paraplegic wheelchair bound**

10 months earlier, a blocked femoral artery had left one of the patient's kidneys non-functional. This was associated with nausea, vomiting and very high blood pressure. He was treated with i.v. iron and permanent blood pressure medication. During the following months, he suffered several urinary tract infections (UTIs) that were treated with antibiotics. 10 months later, whilst the patient was on a course of antibiotics against a UTI, he was hospitalised with pneumonia and septicaemia. During the admission process, he was sat on a punctured surface that was believed to have tiggered this severe and highly exuding pressure ulcer above his tuber ischii, to penetrate his skin from within less than a week later. Based on the severity of the ulcer, it was suspected to have been developing from the bottom up for some time already – possibly caused by a pre-existing osteomyelitis stemming from haematogenous spread from a different primary source of infection. After 17 days in hospital, the patient was stabilised and discharged into community care where the wound was managed with hydrogels, Manuka Honey (Activon gel) or Flaminal Forte (enzyme based antimicrobial alginate), always packed with 10 cm of Durafibre ribbon (cellulose ethyl sulphonate fibres) and covered with a Tegaderm Foam Adhesive (highly absorbent, occlusive dressing). After 10 days, he was readmitted to hospital for another 10 days, due to renewed septicaemia. 6 Weeks treatment for osteomyelitis was prescribed consisting of double doses of two different antibiotics to be given concurrently. These were non-compatible with the patient's blood pressure medication, which he was ordered to stop. After 10 days, the patient was discharged into community care for wound management identical to the first period a few weeks earlier and to finish the 6-week antibiotics course. Here, the patient's blood pressure increased steadily and his nausea and vomiting returned. The wound continued to deteriorate.

After 32 days in community care, when the wound had been open for nine and a half weeks, MPPT treatment was implemented. The wound opening was 2 x 1.5 cm with 1 cm steep sides leading down into the wound bed. Half of the wound was undermined by 1 cm with two tunnels of 2 cm in diameter, and 3 cm and 2.5 cm in depth, respectively. The skin surrounding the wound opening lacked its natural pliability and presented as a dull and live-less pink which faded into a band, darkened by infiltration. Furthermore, the wound was highly exuding (pic 1 & 2). MPPT was used daily for 3 days (pic 2 - 6) and the wound thereafter left without further treatment and covered only with a single, plain interface dressing (pic 7 & 8). To assess the wound, this dressing was changed every 3 days. Despite the patient suffering increasingly from high blood pressure, nausea and intermittent episodes of fever, the wound continued to improve. By the sixth day, the overall volume of the wound had reduced by 90% (Pic 7). The opening had reduced to 1 x 0,5 cm; the undermining was gone; and the sinuses had both reduced by 1.5 cm to maximum expansion of 0.5 cm in diameter and to 2 cm each in depth. The wound bed was granulating, and the entire edges were epithelializing. There was practically no exudate. Over the following days, the wound continued to improve and reduce in volume (pic 8).

Sadly, renewed septicaemia hospitalised the patient again on the morning of the tenth day, only a few days after finishing the 6 week course of antibiotics. Upon hospitalisation his wound was investigated and confirmed unequivocally free of infection. Due to the history of nephrological trouble, the patient was sent on to nephrology for further investigations to locate the primary focus of the causative infective agent. No nephrological issues were identified, but the patient was instead urgently referred for cardiovascular investigation. However, with his blood pressure medication suspended for the duration of the prior 6 weeks, his aorta ruptured before the cardiovascular team attended.

It was later re-confirmed that his death was neither related to MPPT nor to the wound.

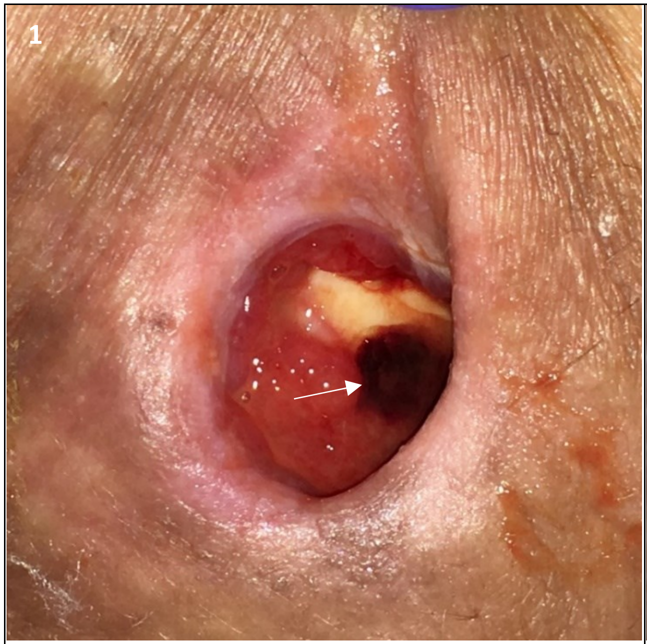	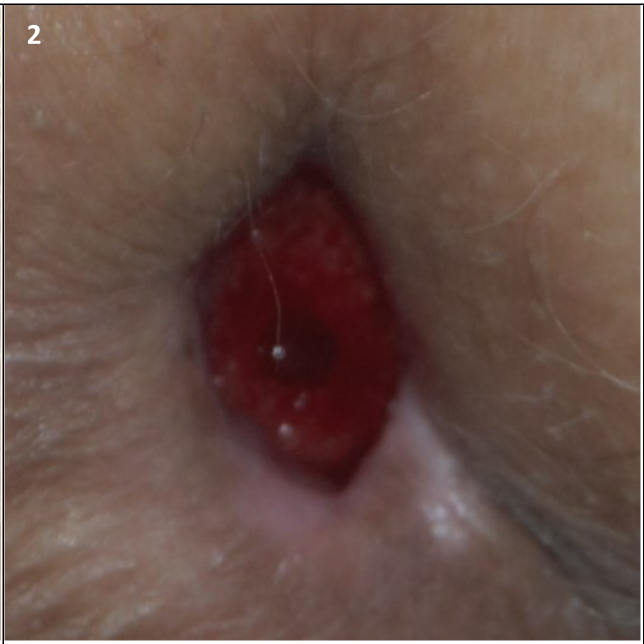	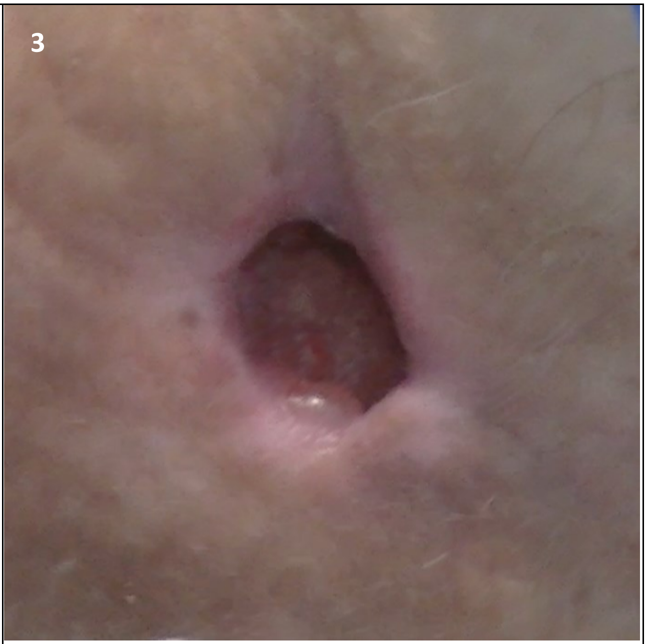
**Day minus-2**<br>**2 days before start MPPT**	**Day 0**<br>**Just before start MPPT**	**Day 1**
Wound bed irritated red. Wound highly exuding.<br>*Arrow:* 2 cm diameter maximum widening tunnel opening. Tunnel 3 cm deep.	Wound opening 2 x 1.5 cm. Sides 1 cm deep. Half of the circumference is undermined by 1 cm.<br>2 tunnels, each of 2 cm maximum widening; 3 cm and 2.5cm deep, respectively.<br>Skin stiffened and darkened.	Considerable reduction in size.<br>Wound bed and sides starting to granulate.<br>Skin no longer stiff.

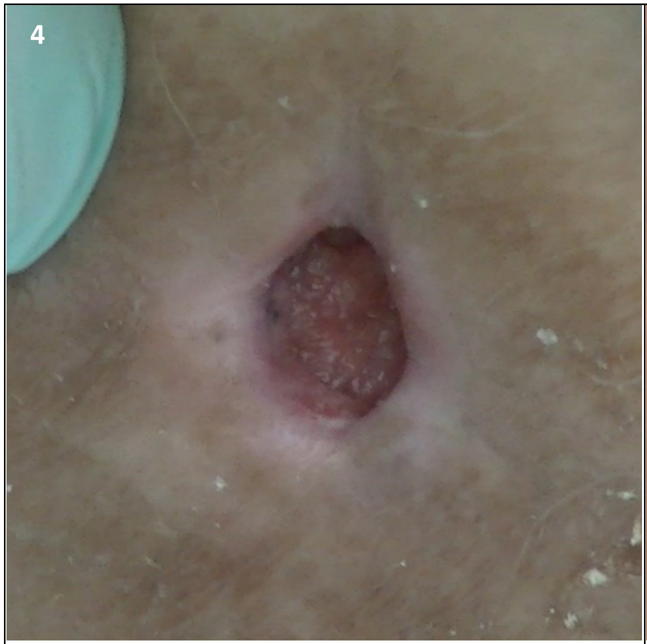

Day 2

Granulating wound bed and sides.

Epithelialising wound edges.

Last MPPT application.

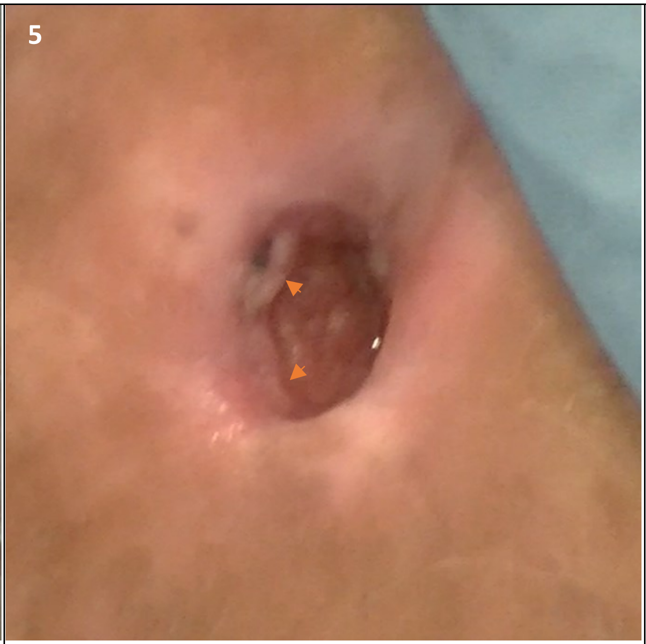

Day 3

The normal plasticity of the skin has been restored, and the darkened band of the skin indicating infiltration has disappeared.

No more MPPT applications.

Arrows: Gorge. (See next picture.)

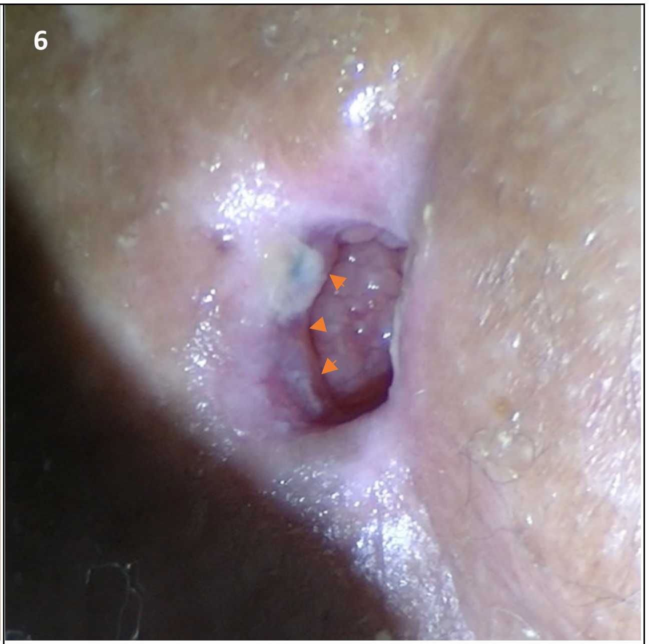

Day 3

This picture has investigative lightning. The entire wound bed is covered with characteristic germ granulation budding, i.e. it is regenerating new tissue swiftly. The wound bed meets the wound sides in a very narrow gorge. Such a gorge is typically the space through which debris is passed from an infection in the underlying bone, through the soft tissue, and lead into the wound to be disposed of. A gorge is therefore often the clinical sign of osteomyelitis.

Arrows: Gorge.

<table>
<tr>
<td>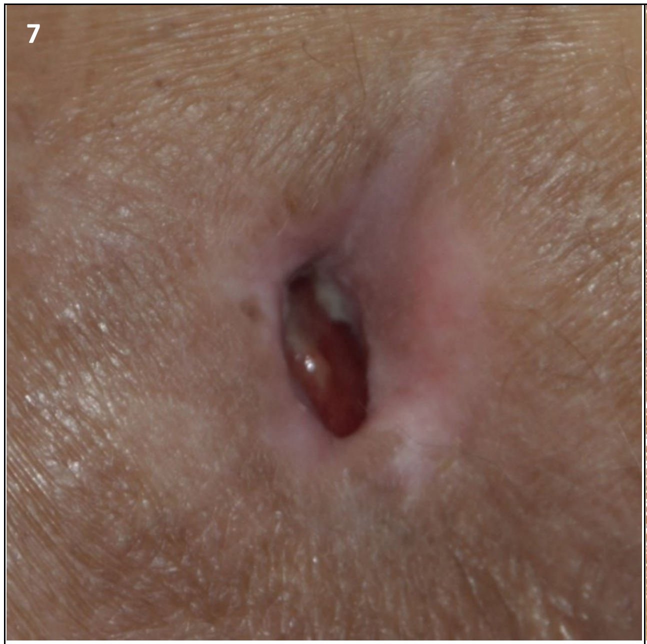
Day 6
Wound opening 1 x 0.5 cm.
No undermining
All wound bed granulating. All edges epithelializing.
2 tunnels each of 0.5 cm diameter maximum widening, each 2 cm deep.
Practically no exudate.</td>
<td>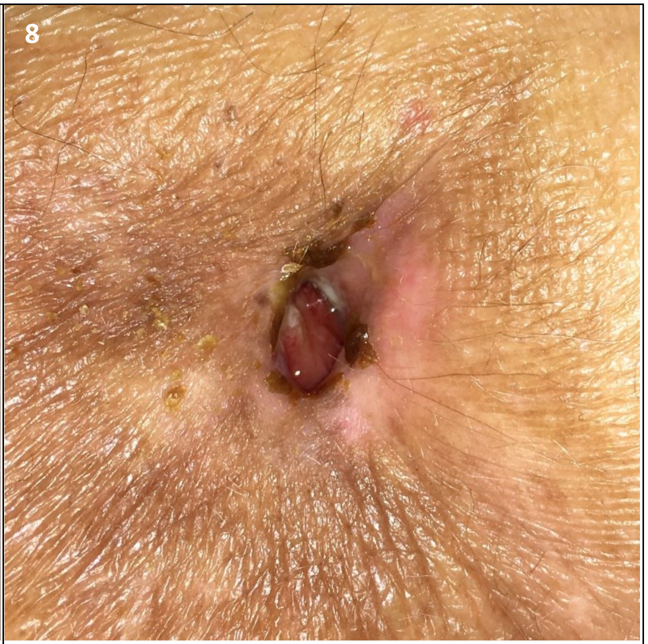
Day 9
Wound opening 0.5 x 0.5 cm. Shallow wound.
100% granulating wound and 100% epithelialising edges.
Picture taken shortly before hospitalisation. The wound has neither been washed nor otherwise cleaned.</td>
</tr>
</table>

Wound number 40			Patient	SCI	
**Grade 4**	**15 months old**	**Ischial tuberosity – Lower back**	**71-year-old male**	**21 years**	**C7/T1**

Six years and 10 months earlier, an ulcer appeared on the ischial tuberosity. It soon started to spread (pic 1) ulcerating a wide area of the adjacent skin in a manner that could possibly indicate **a** dissemination of infectious waste in the interstitial space in and underneath the skin (pic 2). It turned out to be incontrollable despite full bed-rest, antibiotics and antiseptics of all kinds as well as NPWT (pic 3), and approximately 2 and a half years after first detection, to close the wound, the patient underwent extensive flap surgery compromising a large area of the buttock and lower back (pic 4). No bone debridement was performed, and no bone biopsy was taken.

This area broke down again for no immediately evident reason, less than 3 years later, between the old suture line and drain tube exit site. Within 6 weeks, the wound was down to bone and tracking further 9 cm along the bone whilst exuding uncontrollably even with twice daily highly absorbent dressing changes. Despite full bedrest and NPWT for 6 months it continued to deteriorate and break down (pic 6). Extensive surgery to mitigate the recurrence of this wound involved the removal of a large part of the old surgical area (pic 7). No bone debridement was performed, and no bone biopsy was taken. The bone was inspected visually by surgeon and deemed healthy. One of the two drain tubes was left in the wound for a few extra weeks due to unusually high levels of exudate refusing to subside but was eventually removed regardless. After two months, the wound was nearly closed and the patient was discharged.

In the weakest part of the suture line was a small opening (dehiscence) that quickly enlarged (pic 8). In the centre of this wound opening was a minuscule but distinct, unmistakable sinus entrance (pic 9) and the entire suture line and a very wide area surrounding the wound were dark red to purple (pic 10), an indication of dissemination of infectious waste in and underneath the skin, similarly to what the patient had experienced six and a half years earlier (pic 1 & 2). Bedrest and conventional wound management since discharge from hospital had brought about no improvement, and the suspicion that a deep-seated primary source of infection, such as osteomyelitis, could have been overlooked before and during surgery, was reinforced by the fact that the patient, all the time since hospital discharge, had been suffering from, to him, unusually high levels of autonomic dysreflexia caused by none of the “usual triggers”. The wound was now started on MPPT treatment (pic 9 & 10).

The necrotic tissue embedded in the wound bed was rapidly cleared and the wound granulated up to skin level (pic 11) and practically closed within 2 months (pic 12). However, over the subsequent 9 months, an orderly process played out during which the infection, presumably in the bone, became more severe (pic 13 onwards) producing increasing amounts of corrosive waste material containing high levels of both red and green pigmented toxins. This would seep through the most debilitated part of the soft tissue, i.e. the suture line (pic 13, 14 & 15) and the interstitial space in the adjacent soft tissue, to be deposited on the body surface where it would gradually cause the wound to increase in size, superficially only (pic 13, 14, 15 & 16). Eventually, presumably with the osteomyelitis unavoidably becoming more severe over time and generating higher amounts of waste, a tunnel was generated (pic 15, 16, 17 & 18) that allowed the bone waste material to drain directly to the surface without using many different and changing interstitial pathways to reach the surface. This meant, that the wound area could start gradually regenerating (pic 18) and decreasing the surface area of the wound as well as the diameter of the fistula by keeping the travelling infectious waste from harming the tissue lining the fistula and keep it infection free until surgery could be performed. There was no smell from the wound and the bone exudate was manageable with a gauze pad on top of the wound changed daily. Blood infection markers (CRP) were consistently below 20.

For reasons unrelated to the wound, the wound was shortly after taken off MPPT treatment and instead daily packed 2 cm deep with Aquacel and 2 x Mepilex and 1 x Tegaderm on top. However, soon the amount of exudate from the wound was excessive and all dressing attempts would leak or fall off. The wound rapidly deteriorated further, the diameter of the fistula enlarged, the visible depth was 9 cm and the patient started to feel unwell. He was administered a 2-

week course of oral antibiotics (Co-Trimoxazole) and finally admitted via A&E and put under observation for osteomyelitis but with no treatment decision made. The wound was treated in hospital for a week with povidone-iodine (Betadine) leaving both the wound bed and the skin surrounding the wound burnt. The patient was discharged into community care for wound maintenance. Approaches mainly based on silver were used over the following 10 months. The CRP had been rising steadily over several months and had reached 268 (reference range <10 mg/L) when sepsis developed from the wound and the patient spent a week in ICU. An emergency MRI revealed widespread osteomyelitis in the ischium, the acetabulum and the femur. During inspection, pieces of bone were pulled out through the wound. The patient was operated for osteomyelitis as an emergency 2 weeks after hospitalisation. The operation was in infected soft tissue and, typical for advanced osteomyelitis, the haemoglobin was dithering around 68 g/L (reference range 130-180 g/L). The patient received several litres of blood transfusion, indwelling slow-release antibiotics and IV antibiotics.

After 12 weeks in hospital, the patient was discharged with three more weeks of IV antibiotics to be administered at home and an approximately 7 x 7 hydrocele which could not be operated due to the elevated risk of complications. Immediately after finishing the course of antibiotics, the patient again suffered bacteraemia and septicaemia and was treated with additional 3 weeks of antibiotics. During this course, and in a matter of 2 weeks, a 7 cm deep tunnelling wound opened down to bone on the opposite hip. This presumably was an indication that the osteomyelitis had spread contiguously beyond the extensive amount of removed bone and infected the opposite side of the hip. The fact that the surgical site itself was still exuding and refusing to close half a year after the operation (pic 19 & 20) was a further indication that the bone surgery had failed in removing the 9-year-old bone infection causing the septic state. The patient had lived an independent active life as a paraplegic for 22 years, but this ordeal had left him unable to sit up in bed by own force or to be hoisted due to severe stomach cramps and unable to perform his usual daily bowel routine. He was now left fully dependent on care. An attempt to re-operate to remove the remainder of the infected bone would be too high a risk due to his overall deterioration in health but leaving it without operating constituted an equally high risk.

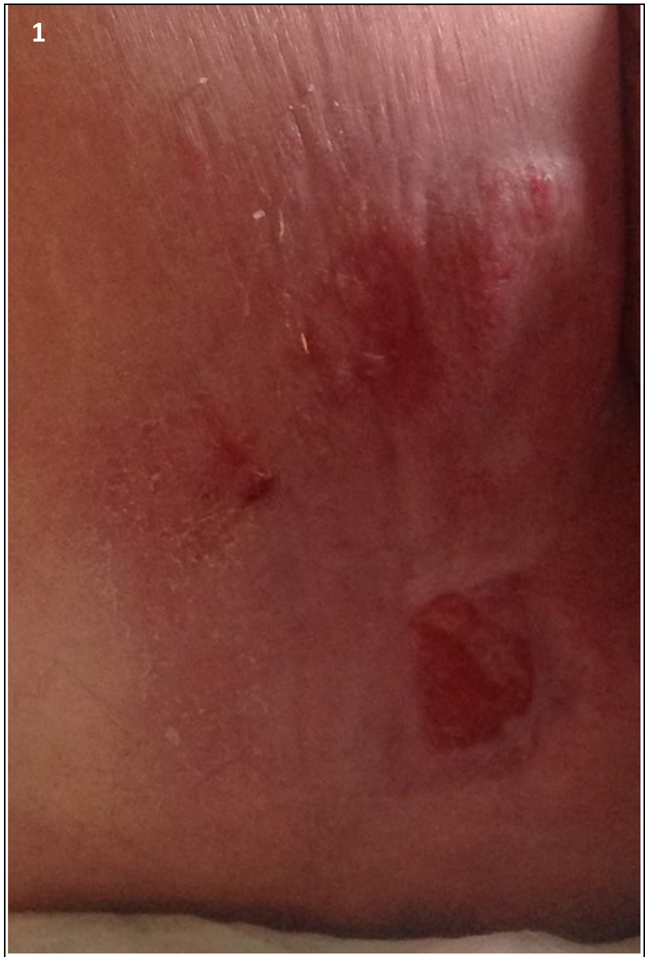

Day minus-2414

2 years 3 months before first surgery

6 years and 7.5 months before MPPT

An at least 2.5 months old skin lesion skin on top of the ischial tuberosity.

Ulceration of a large area of the surrounding skin.

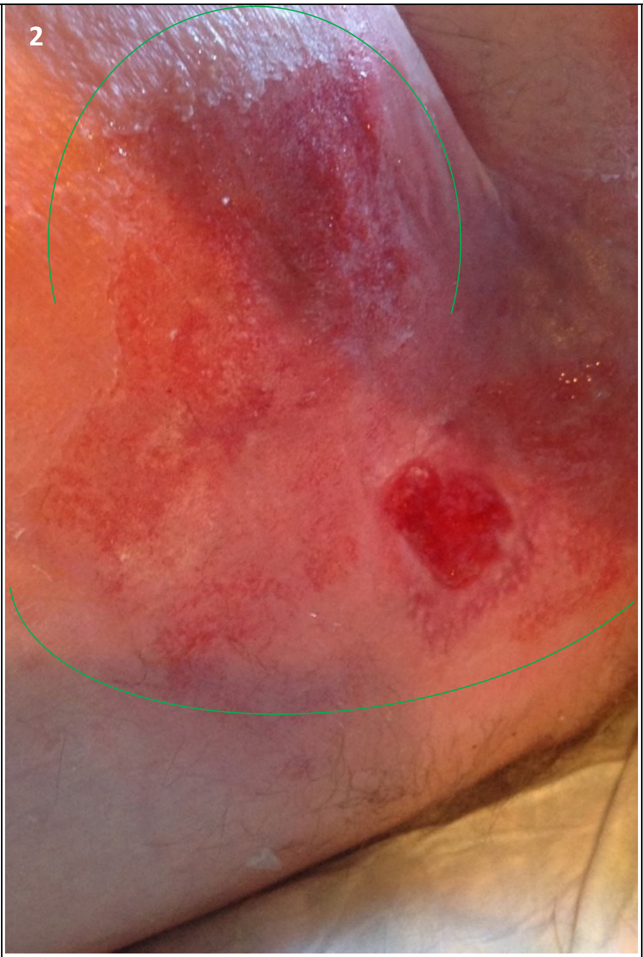

Day minus-2363

2 years 1.5 months before first surgery

6 years and 6 months before MPPT

The wound opening remains of similar size.

The ulceration of the adjacent skin is more widespread and more severe. Possibly an indication of dissemination of infectious waste of a different origin in the interstitial space in and underneath the skin.

Green semi-circles: Area experiencing disseminated uncontrolled spread and ulceration.

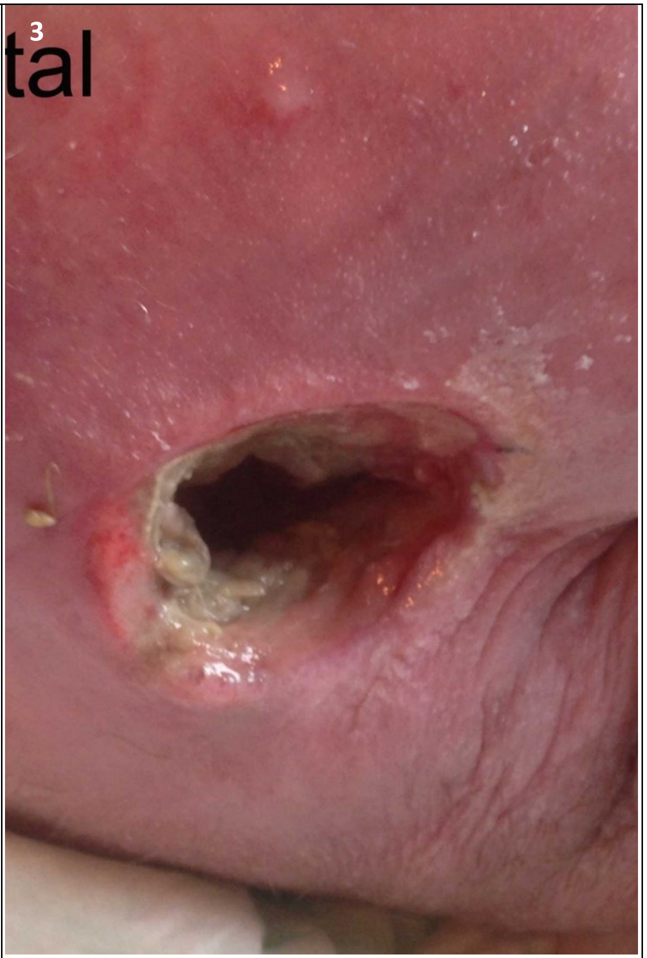

Day minus-1817 Closeup

7.5 months before first surgery

5 years before MPPT

Immediately following some months with NPWT (VAC) followed by 2 weeks in hospital due to the wound.

The infectious waste has concentrated into draining through a defined deep fistula / wound. The bottom is hidden under green-brown slough of an unknown thickness. The sides lining the fistula are covered with 5 – 10 mm of green-brown slough.

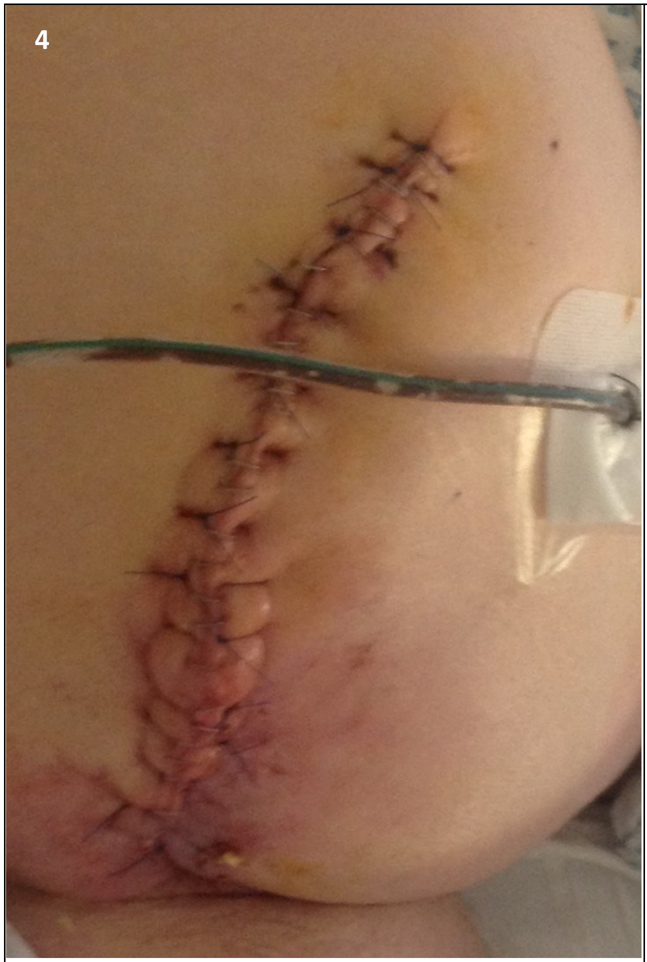

Day minus-1589

The day after first surgery

4 years and 4 months before MPPT

Extensive flap operation involving buttock and lower back in first attempt to close the 2.5 year old wound presumably on top of osteomyelitis.

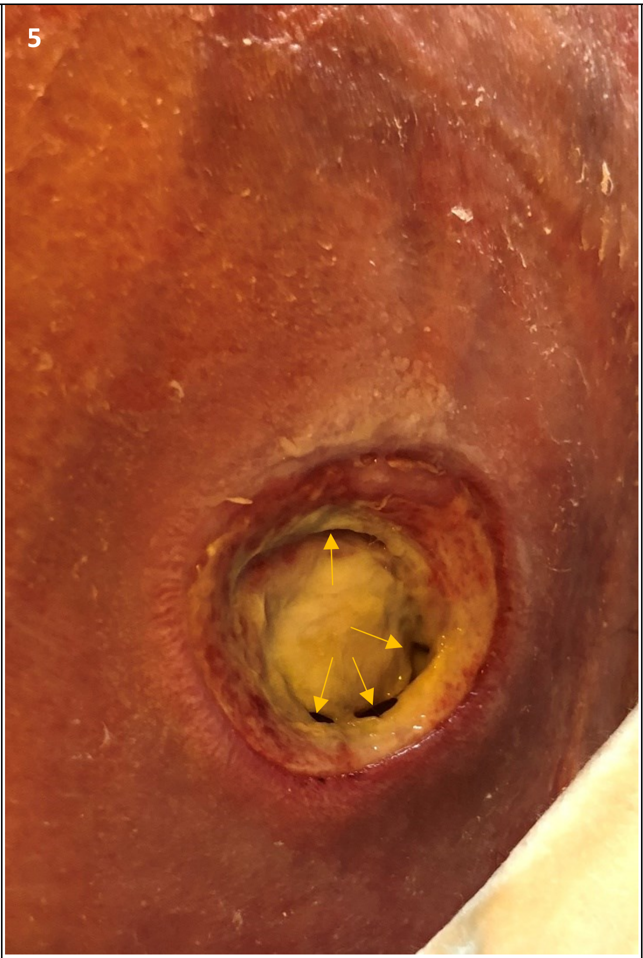

Day minus-234

4.5 months before second surgery

7.5 months before MPPT

The broken down surgical area exhibiting a renewed draining fistula.

The fistula is plugged by viscous slough of unknown depth. Openings along the sides of the plug allow for non-viscous exudate to exit (*yellow arrows*).

The visible part of the wound sides is sloughy and the wound-skin edges are unhealthy and breaking down.

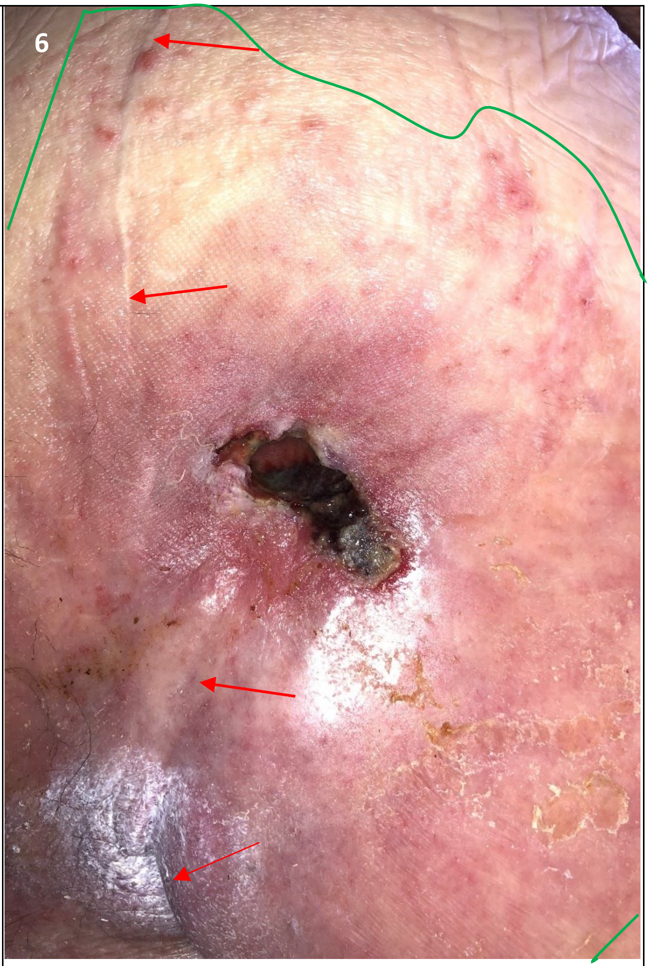

Day minus-132

1 month before second surgery

4.5 months before MPPT

1 year after the ulcer resurfaced. Treated the past 6 months with NPWT and full bed rest. The wound is exuding uncontrollably and continuing to break down. The surrounding skin is dark purple-red indicating deep involvement.

Red arrows: first surgery suture line (scar).

Green lines: Border of skin involvement indicating imminent breakdown.

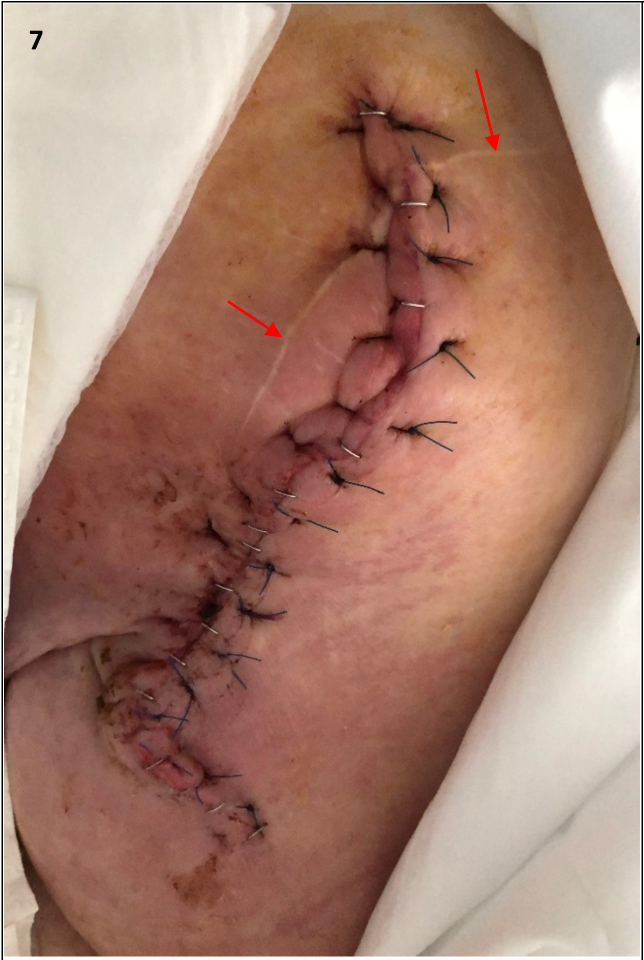

Day minus-95

5 days after second surgery

3 months before MPPT

The 2 drain tubes are covered by the sheets.

Distal part of first suture line has been removed in this second surgery.

Red arrows: first surgery suture line (scar).

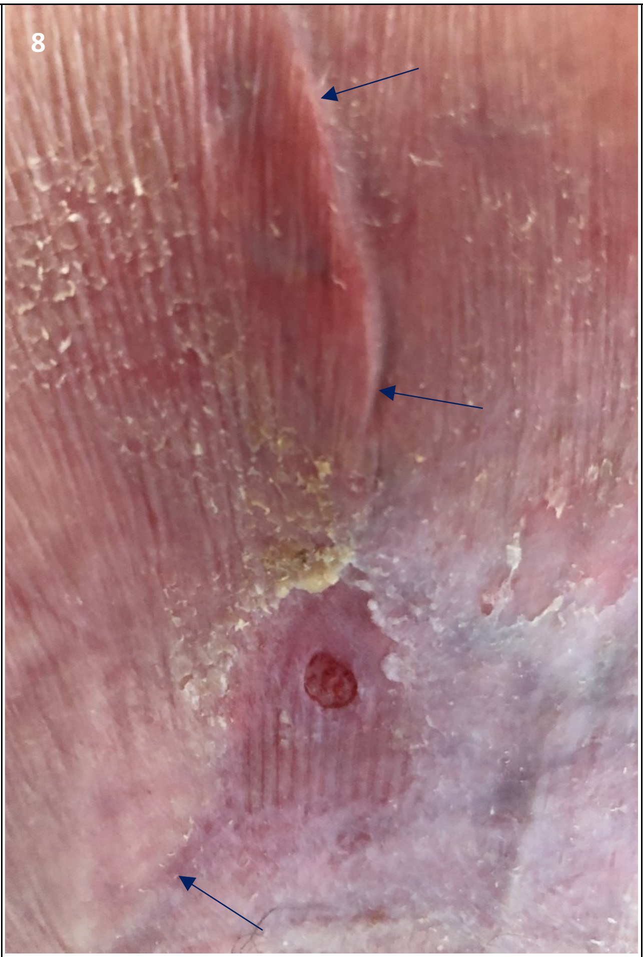

Day minus-4

1 month after discharge from hospital

4 days before MPPT

Dehiscence.

Wound surroundings are dark red to purple

Blue arrows: second surgery suture line (recent scar).

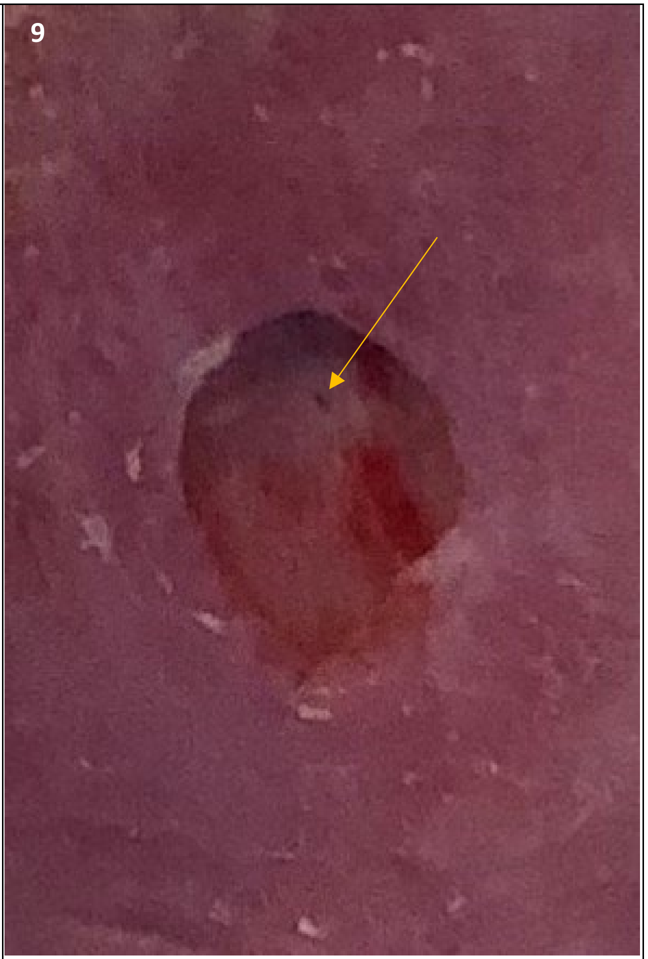

Day 0 Closeup

Just before first MPPT

Close-up showing minuscule, distinct sinus opening centrally in reopened suture line through which infectious waste drains, presumably from the bone.

The wound bed and surrounding skin are purple and looking lifeless.

Yellow arrow: sinus opening.

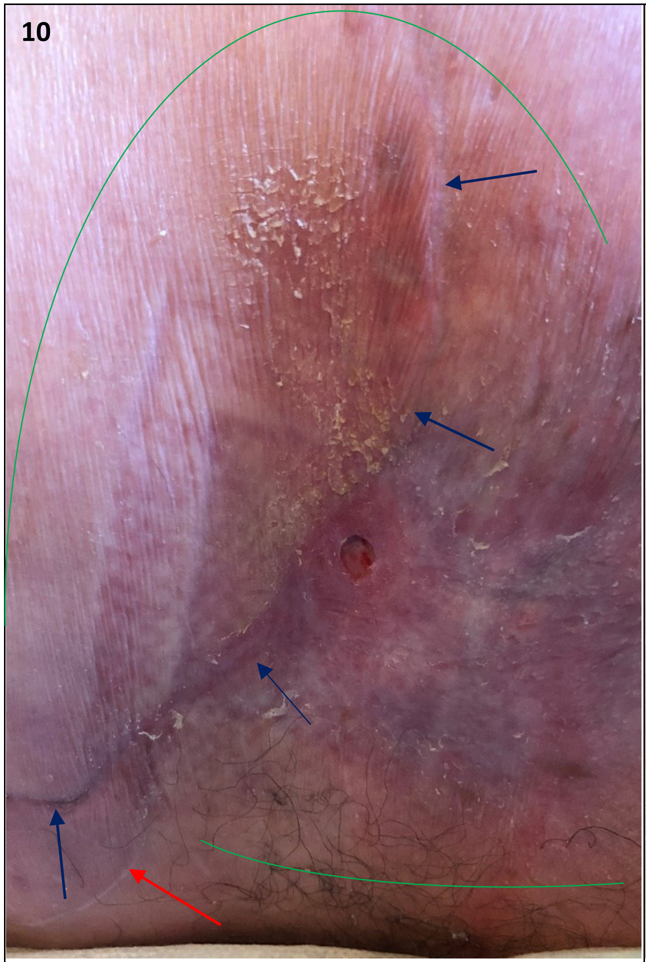

Day 0

Reopening suture line 2 weeks after discharge. The sinus opening is in the centre of the dehiscence. The recent suture line and a wide area of the surrounding skin look purple and strained - an indication of dissemination of infectious waste under the skin.

Red arrows: first surgery suture line (scar).

Blue arrows: second surgery suture line (recent scar).

Green semi-circles: extent of purple, strained skin.

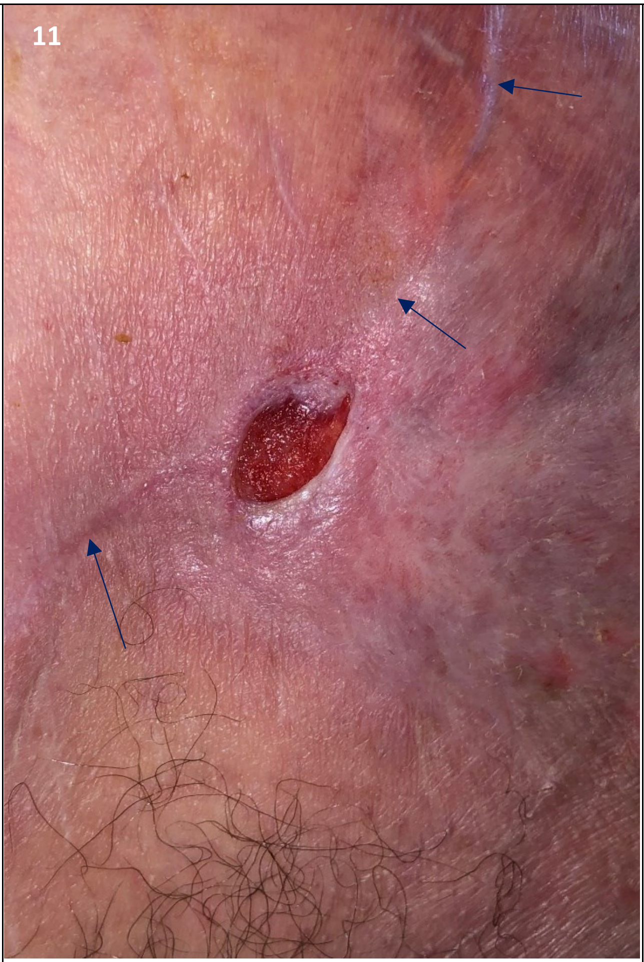

Day 37

The wound bed has granulated up to skin level and new skin is moving in. Whole area is less purple with the skin looking healthier. The suture line is still purple.

Blue arrows: second surgery suture line (recent scar).

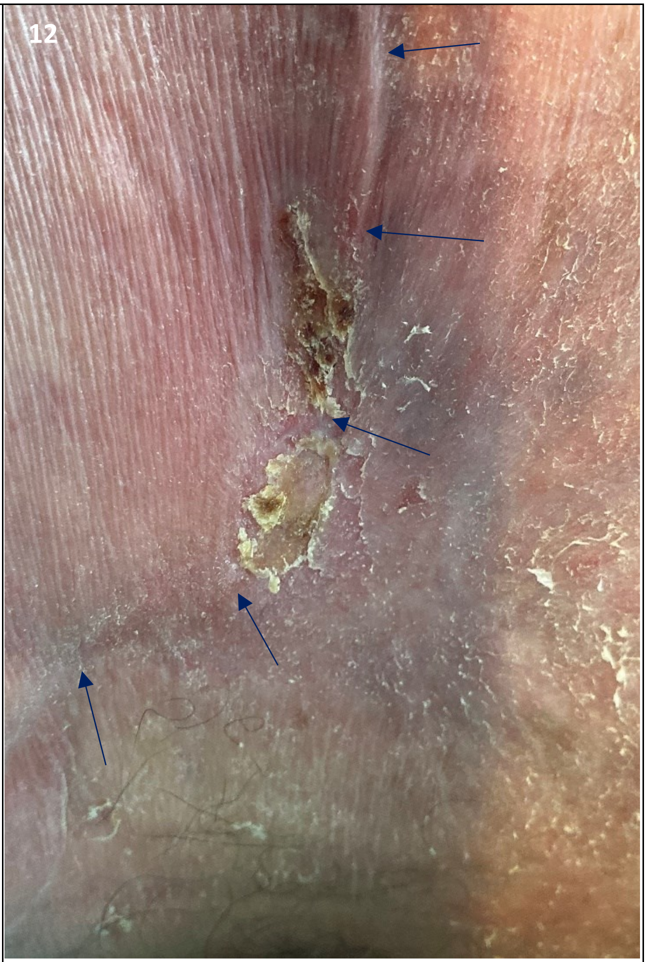

Day 62

The wound is practically closed.

The surrounding skin as well as the suture line are no longer purple.

However, the last bits of thin scab refuse to fall off and the wound therefore cannot be considered fully closed.

Blue arrows: second surgery suture line (recent scar).

13

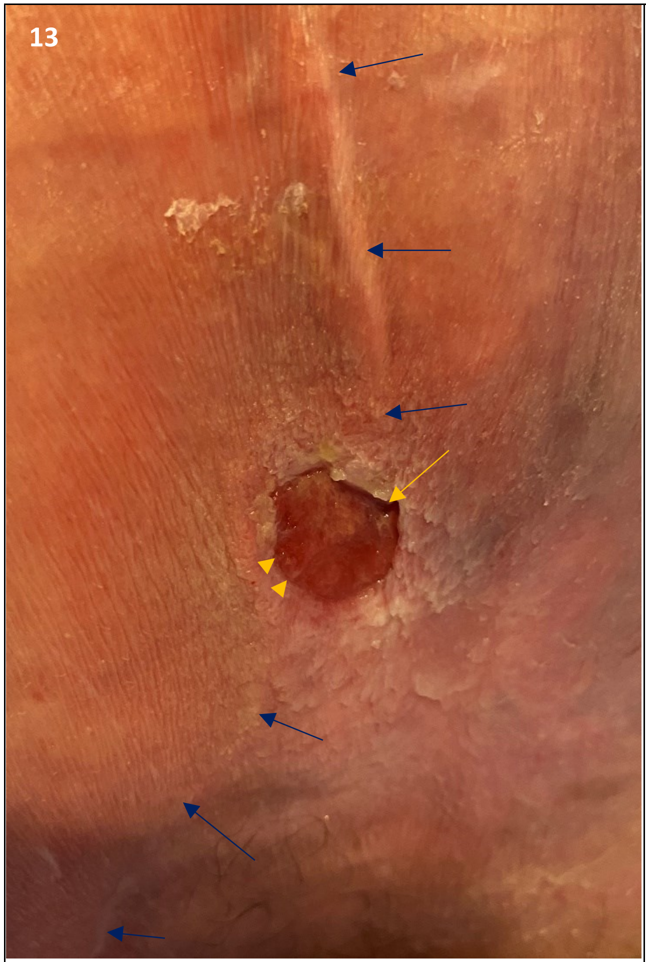

Day 144

As the osteomyelitis expands, the amount of corrosive waste draining to the body surface through the suture line increases, again carving out a superficial wound. The recent suture line remains clear and not red-purple. A distinct sinus opening is showing right in the suture line and a few more pinhole sized ones are scattered along the edge of the wound opening.

Yellow arrows: examples of sinus openings disseminated over the wound bed.

Blue arrows: second surgery suture line (recent scar).

14

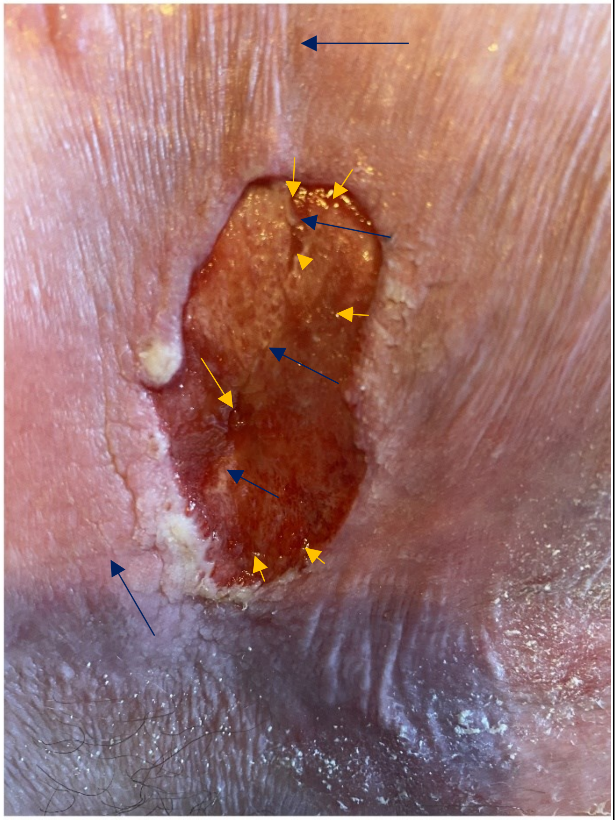

Day 274 Closeup

An increased volume of osteomyelitic waste is seeping through a longer part of the suture line. The wound is kept superficial not involving deep soft tissue.

The known distinct sinus opening is a bit wider and more pinhole sized sinus openings are scattered along the wound edge and in the suture line inside the wound bed. These are exit holes of gas and liquid waste passing through the interstitial space of the soft tissue and, as the territory they pass through

15

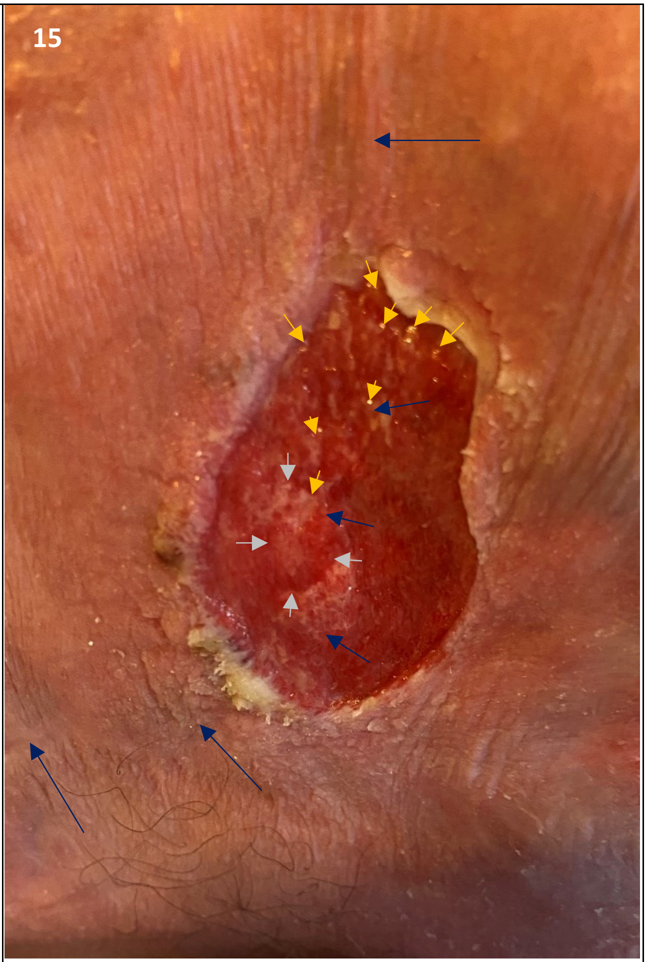

Day 300 Closeup

The wound is still superficial without soft tissue infection. (The picture is taken before washing the wound.)

Even more pinhole sized sinus openings are scattered along the wound edge and in the suture line inside the wound bed.

A rounded shape is forming in the wound bed right in the suture line and where the known most distinct and persistent sinus has been visible throughout.

Blue arrows: second surgery suture line (recent scar).

	expands, their waste causes corrosion of the skin around them, thereby enlarging the wound opening.<br><br>*Yellow arrows*: sinus openings disseminated in the wound bed.<br><br>*Blue arrows*: second surgery suture line (recent scar).	*Yellow arrows*: sinus openings disseminated over the wound bed.<br><br>*Grey arrows*: sinus opening gradually shaping into a draining fistula.

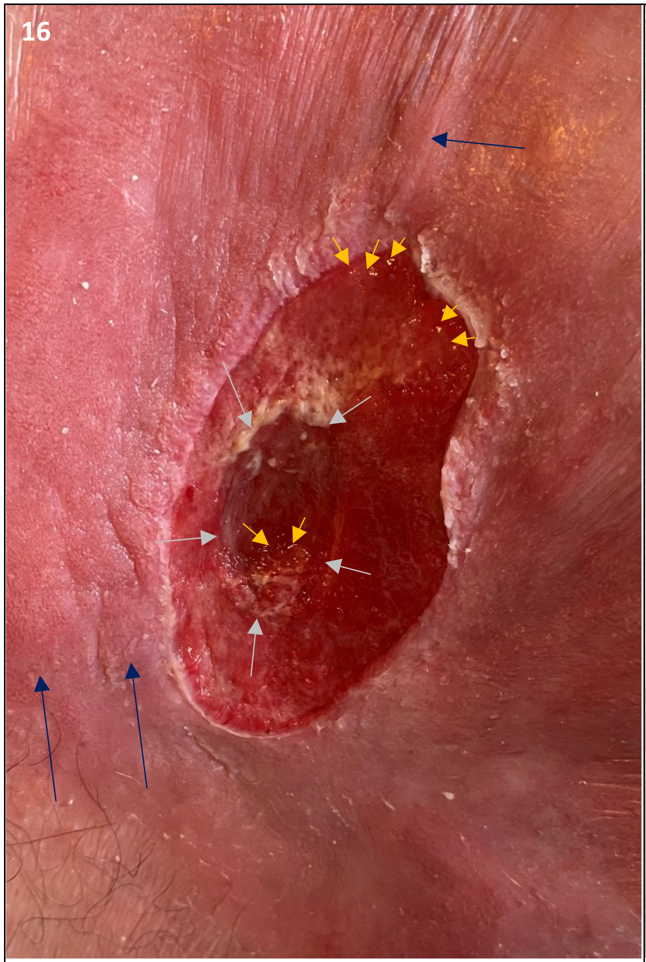

Day 309 Closeup

The centre part of the wound is showing signs of hollowing but no signs of breaking down. The soft tissue remains free of infection. Increased osteomyelitic waste forces the body to open a formal fistula for draining the waste rather than it seeping through large areas of soft tissue and exiting through the tiny sinus openings, at the risk of breaking down a large area. This fistula is in the process of being carved.

Wound edges and surrounding skin are healthy.

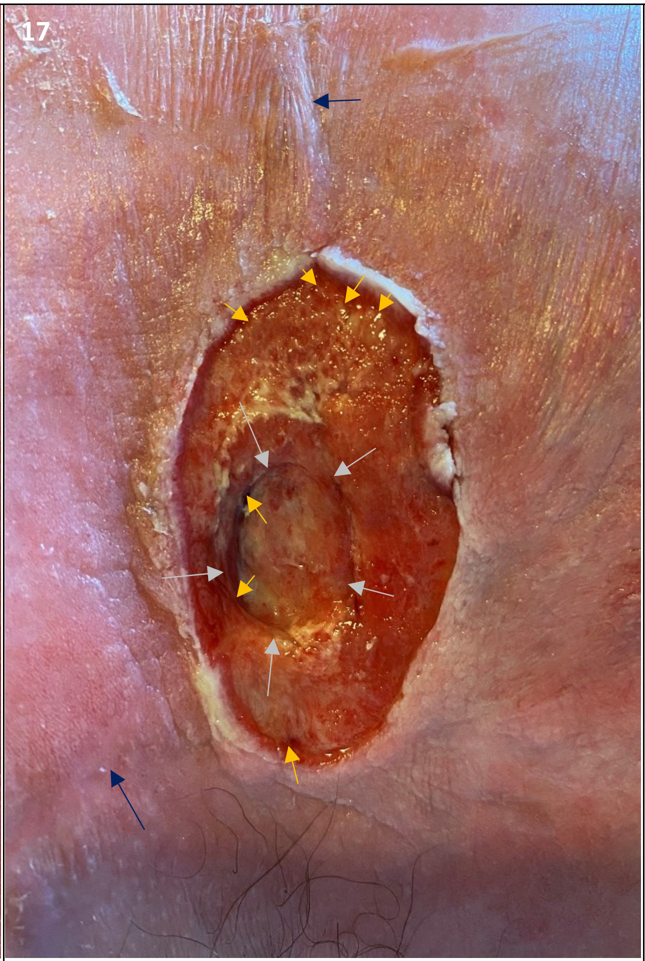

Day 314 Closeup

The fistula is more defined, narrowing and deepening. The gorge pattern, typical of underlying osteomyelitis as primary source of infection, is obvious.

The wound is showing signs of epithelialisation and reducing in circumference. Surrounding skin is healthy.

Yellow arrows: Gorge formation at what is often perceived as the bottom of the wound but is actually the demarcation of the soft tissue gorges through

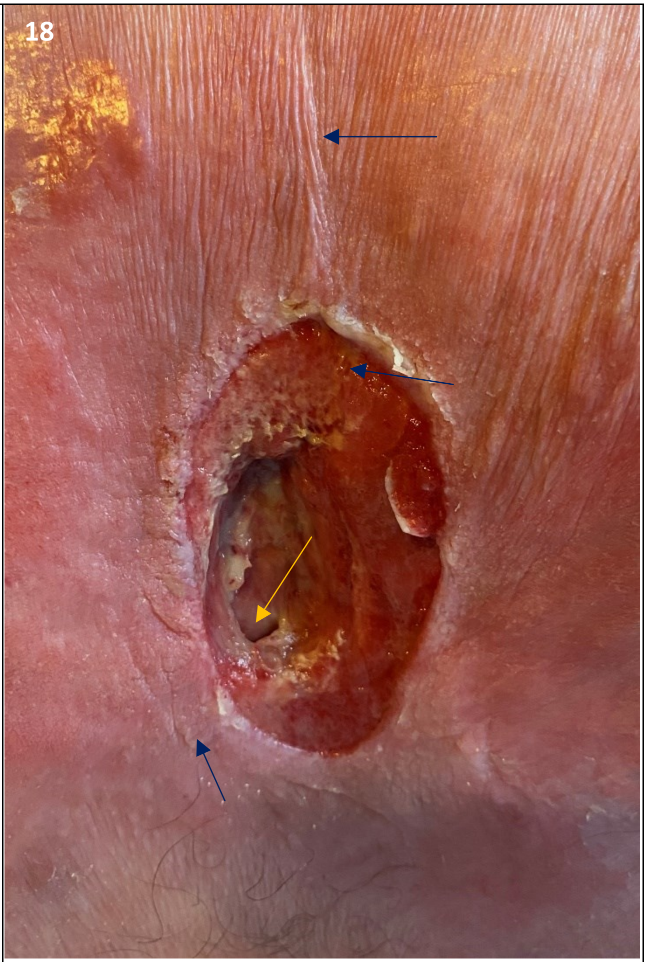

Day 319 Closeup

A couple of days before discontinuation of MPPT.

A non-infected draining fistula has been established in a well-controlled manner. The fistula secures an easy, direct pathway for the infectious debris to pass collectively to the surface without infecting the adjacent tissue and causing it to break down. The infectious debris needs no longer pass through very narrow, randomly distributed paths through the soft tissue interstitium and exit through the widely disseminated minuscule sinus openings. Their disappearance is allowing the wound to rapidly

Yellow arrows: Hollowing of the soft tissue in the suture line in precisely the same location as the minuscule distinct sinus opening was originally observed on Day 0, see pic 9 and 10.<br><br>*Grey arrows*: sinus opening gradually shaping into a draining fistula.<br><br>*Blue arrows*: second surgery suture line (recent scar).	which the bone debris passes/squeezes when expelled from the body.<br><br>*Yellow arrows*: sinus openings disseminated over the wound bed.<br><br>*Grey arrows* draining fistula gradually taking shape and displaying the gorge pattern.	reduce in surface area. This is consistent with the epithelializing wound edges and the surrounding skin being healthy.<br><br>The scar line from the latest surgery is healthy and displaying no signs of directly underlying trouble.<br><br>The wound is not exuding excessively and is kept covered with a single cotton gauze pad. No smell.<br><br>*Yellow arrow*: draining fistula.<br><br>*Blue arrows*: second surgery suture line (recent scar).

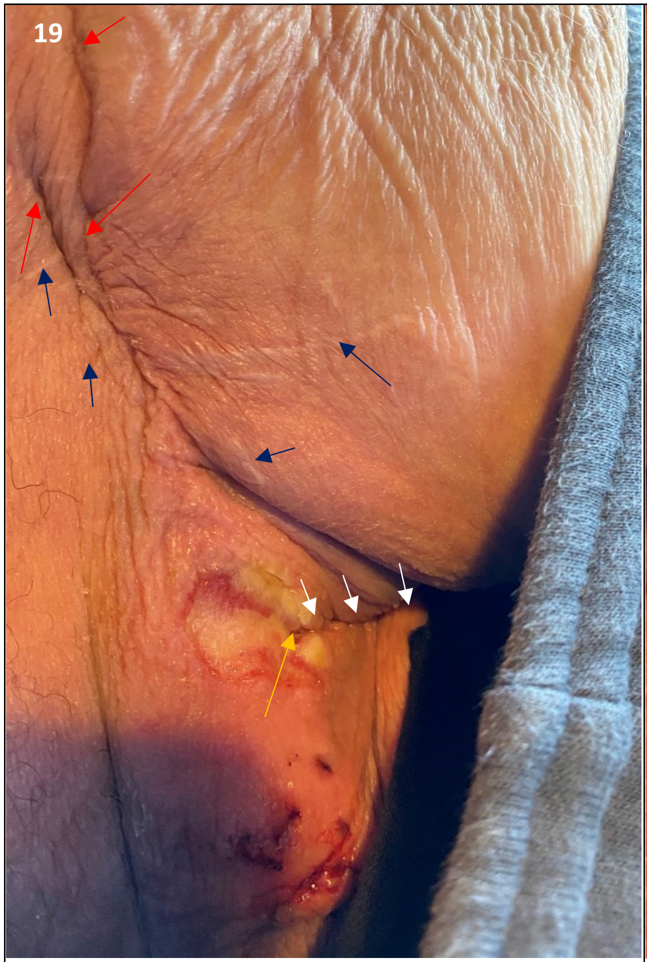

Day 168 after third surgery

5.5 months after operation for osteomyelitis

Treated with conventional dressings – no MPPT used following surgery

At least 9 years 2 months after first detection of wound

Day 860 after first MPPT

The most recent suture line / scar (white arrows) is a nearly ½ year old scar. The sides are not joining accordingly and the suture line is displaying a

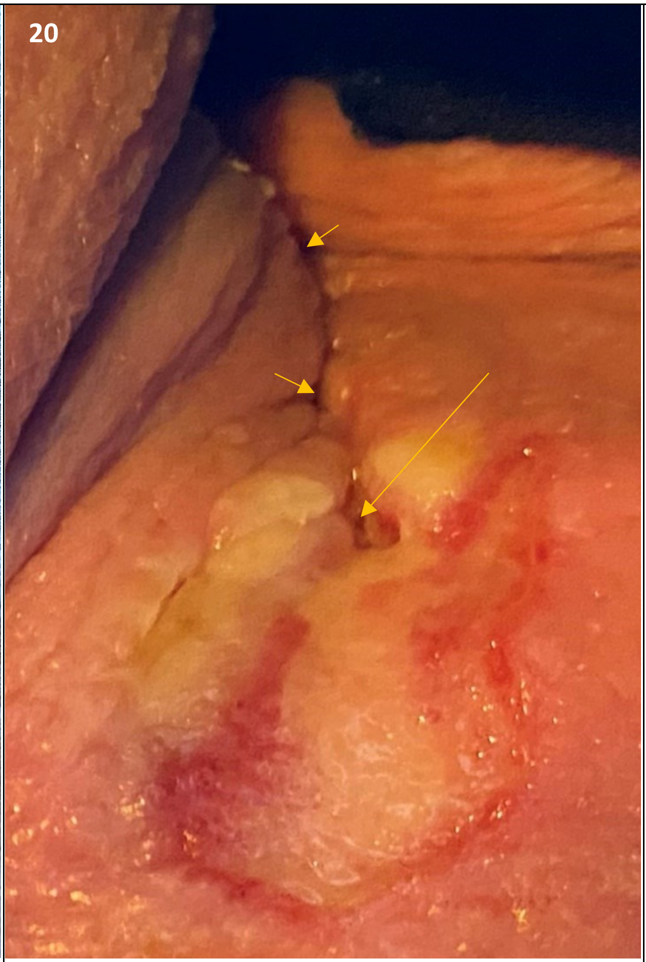

Day 168 after third surgery Closeup

5.5 months after operation for osteomyelitis

Treated with conventional dressings – no MPPT used following surgery

At least 9 years 2 months after first detection of wound

Day 860 after first MPPT

The corrosive exudate from the osteomyelitis keeps draining out in poorly joined areas of the nearly ½ year old suture line. The infectious waste exuding

number of dehiscences, presumably where osteomyelitic debris is again oozing out. The end closest to the camera (dorsal view) is displaying a distinct draining opening with an adjacent wound, seemingly caused by the corrosive waste material.<br><br>The lower part of the picture shows the result of attempts to deal with the hydrocele.<br><br>*Red arrows: first surgery suture line (scar).*<br><br>*Blue arrows*: second surgery suture line (scar).<br><br>*White arrows*: third and most recent osteomyelitis operation suture line (scar).<br><br>*Yellow arrow*: the opening of a new draining canal.	from the most distinct sinus opening (long arrow) is causing ulceration of the skin in a way similar to a river delta.<br><br>*Yellow long arrow*: the opening of a new draining canal.<br><br>*Yellow short arrows*: minor dehiscences allowing the exodus of disseminated travel of osteomyelitic debris through the weakest area of the soft tissue, i.e. the most recent suture line.

Wound number 41			Patient	SCI	
**Grade 4**	**2 years old**	**Coccyx – Gluteal fold**	**42-year-old male**	**24 years**	**T5 complete & C6 Syrinx**

The wound originated as a superficial scratch in the sacral/intergluteal area during a transfer (pic 1). 6 months later, the wound had turned into a cavity and was from then onwards placed on a daily regime of irrigation (later changed to 5 minutes soaking) with polyhexanide-betaine, an antiseptic-surfactant combination (Prontosan); application of alginate-antimicrobial-enzyme protease modulator gel (Flaminal Forte) into the void; followed by packing with ionic-silver impregnated carboxymethylcellulose ribbon (Aquacel Ag), all covered with a superabsorbent cellulose fluff dressing (Zetuvit Plus). The patient suffered several incidents of septicaemia mitigated with systemic antibiotics, and 14 months after first detection, he was hospitalised with sepsis which he survived by means of the antibiotic, Flucloxacillin. By that time, the wound had developed into a grade 4 ulcer with palpable bone expanding into a considerable void underneath the relatively small opening in the skin (1.5 x 1 cm) and with scattered tunnels and gorges. There was a suspicion of osteomyelitis. Upon discharge, the wound management regime was continued as before, except interrupted 4 months, and again 6 months, later when NPWT (the vacuum pump) was tried, but to no avail.

2 months after the last NPWT attempt, i.e. 22 months after the wound was first detected, MPPT treatment was initiated. The patient would go to work in the mornings for a half day's work and for the remainder of the day maintain bed rest as far as is possible for a person living alone and completely independently.

At the start, the wound was associated with a very strong odour. The wound edges and surrounding skin were macerated with signs of diffuse extended infiltration. The wound dressing would leak regularly as the level of exudate could not be contained with the daily highly absorbent packing (60 cm AquacelAg ribbon) and dressing materials (Zetuvit Plus). The skin opening was 1.5x1 cm and opened into an approximately 60 cm^3 void. It was exposing 4 cm of the sacrum-coccyx spine in the cranial end of the void. This was prominently palpable dorsally and along both sides. The wound was widely undermined and comprised very wide gorges extending 2cm cranially, 0.5cm caudally, 3.5cm left and 3cm right. Furthermore 2 narrower tunnel entrances leading medially towards bone were visible in the directly observable part of the wound bed (pic 3). The wound bed was infiltrated with embedded dispersed ropes of fibrinous slough (pic 3 & 4). Air-bubbles were visible, and within 15 minutes or less of washing thoroughly with water, the wound bed would again expose dispersed clusters of froth formation (pic 3 & 4) - presumably due to gas-producing infective agent(s), and often an indication of osteomyelitis.

The odour, exudate level and the width and length of the gorges reduced quickly over the coming days. After 3 weeks the smell had disappeared completely, the exudate level was down to the patient only needing a simple cotton gauze swab covering the wound to absorb it. The soft tissue showed no signs of infection (pic 5). The volume of the void had reduced by an estimated 70%. Bone structure was no longer visible, although its presence underneath a layer of soft tissue was still palpable. The entire interior of the void was regenerating with new pink granulation, marbled in between with white connective tissue (pic 5). The undermining and tunnelling distally in the void, i.e. furthest afar from the originally palpable coccyx, had resolved and the void granulated up entirely and epithelialized accordingly (pic 5), including reducing the opening by 5mm distally.

The wound was evidently healing very efficiently and would, under normal circumstances, have been expected to progress to full closure. However, at dressing changes, froth would continue to appear on the wound bed within 15 minutes after showering the wound. Furthermore, the cranial part of the wound did not follow the example of the distal part. Instead, it entered a cyclic pattern, in which new tissue would build up efficiently, very narrow tunnels or rather gorges would appear in this new tissue in the direction towards the coccyx, followed by foam appearing from these tunnels, soon to be succeeded by expulsion of very

distinct green and red (not bloody) colouring on the covering gauze pad indicating the expulsion of distinctly green- and red-pigmented bacterial toxins. Then, the tunnel and its surrounding tissue would break down - only to be followed by a new identical cycle of tissue build-up, etc.

The froth was interpreted as gas generated by anaerobic fermentation at the bottom of the tunnels and gorges, i.e. in the bone; and the green and red evacuations, as expulsions of pigmented bacterial toxins. Furthermore, as the wound, prior to MPPT, had been treated for 16 months with surfactants, antiseptics (betaine, PHMB and silver) which are cytotoxic, including to human fibroblasts and osteoblasts (Yabes et al. 2016), osteomyelitis was considered the most likely reason for non-closure of the wound.

MPPT kept the wound infection free for 3 months until a triple bone-biopsy confirmed the presence of osteomyelitis caused by mixed anaerobes with no specific known organism identified. As the patient suffered from regular toxaemia caused by the infection in the bone, he was treated with a six-week course of Metronidazole. This was combined with MPPT in the wound to prevent the increased amount of infective debris, entering the wound from the triple-damaged infected bone, from creating renewed soft tissue infection. The prospect of operating in non-infected soft tissue would also lower the risk associated with the subsequent surgical bone debridement and increase the overall chances of success. MPPT was used on an irregular basis during the seven and a half months' wait for surgery. The patient was discharged from hospital a week sooner than anticipated (pic 6).

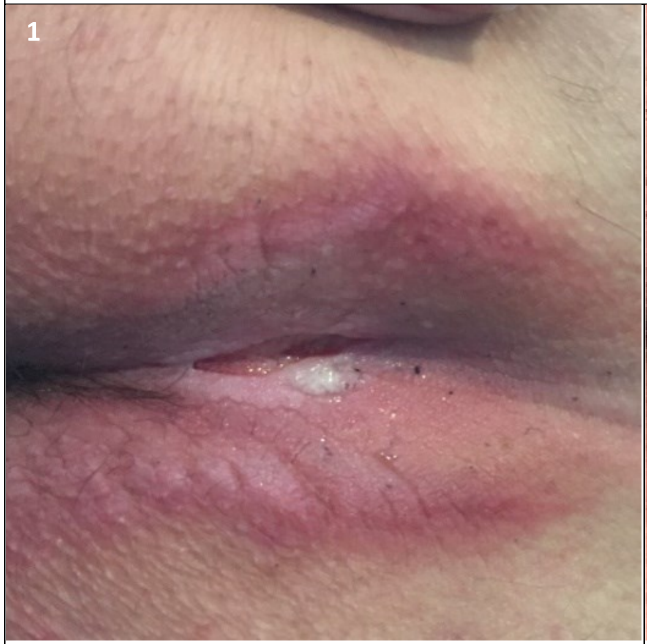

Upon detection of laceration

22 months before MPPT

Superficial laceration with surrounding soft tissue inflammation.

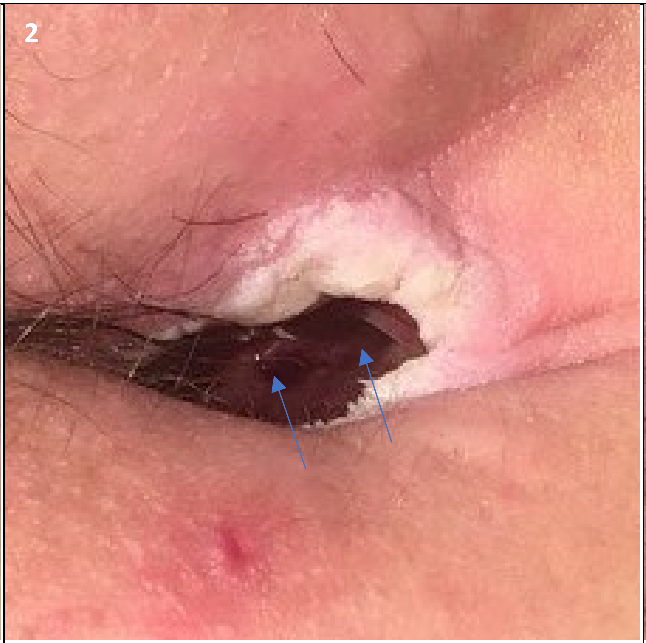

3 weeks before MPPT

Wound bed irritated strong red.

Blue arrows: medial sinus openings.

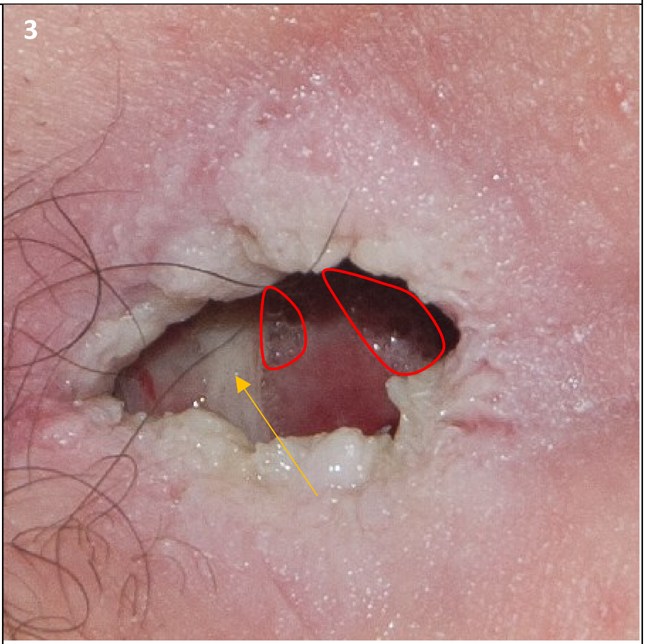

Day 0

Just before first MPPT

Macerated skin edges.

Red outlines: froth formation in tunnel openings.

Yellow arrow: tough embedded, fibrinous slough.

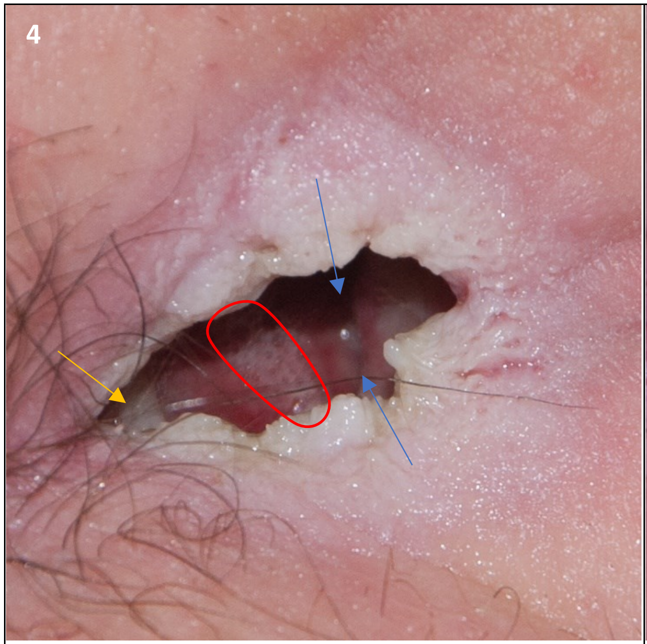

Day 0

from different angle

Blue arrows: gorge.

Red outlines: froth formation in sinus openings.

Yellow arrow: embedded, fibrinous slough.

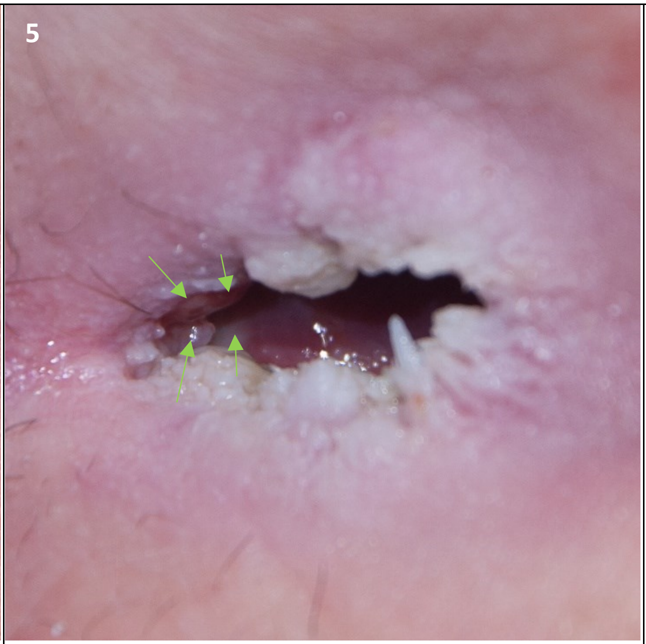

Day 18

The strong granulation is evident in the distal interior wall (left) and in the white-marbled wound bed. The cavity distally under the skin has filled out with new tissue and epithelialized, thereby reducing the opening by 5 mm in the distal end (left side in picture). There is no cavity, tunnelling or gorge formation distally under the skin. The cranial cavity, i.e. on top of the bone is still existent.

Green arrows: examples of strong granulation.

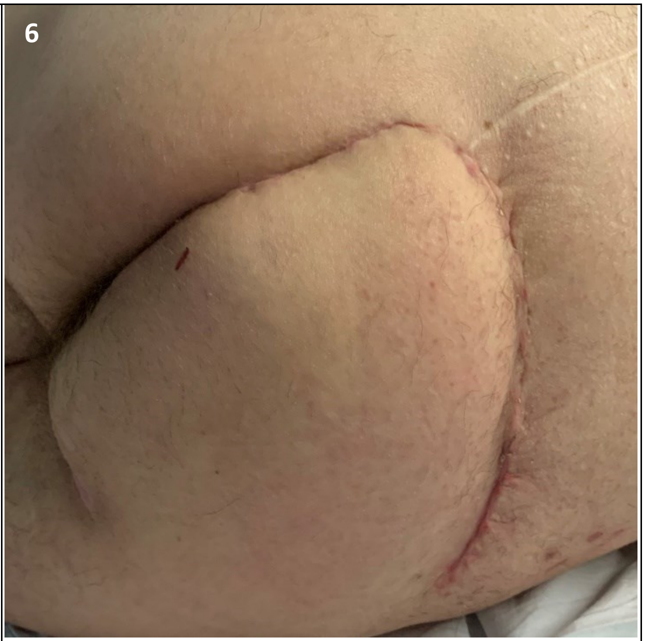

Just before leaving hospital 5 weeks after osteomyelitis surgery.

2 years 7 months after first detection of wound.

Day 270 - approx. 9 months - after first MPPT

Post-operation healing has been unproblematic.

Wound number 42			Patient	SCI
**Grade 4**	**3 years old**	**Sacrum**	**75-year-old male**	**55 years C2/3 incomplete**<br>**4 years T10/11 complete**

The wound was a low sacrum ulcer practically located in the natal cleft. It had been managed with simple dressings for a year and a half when it took a sudden, severe turn for the worse, opening wide and deep with a substantial increase in exudate and smell. After that, all conventional wound management approaches had been tried, including negative pressure wound therapy. All had proven unsuccessful. The wound remained malodorous and continued to exude excessively and the patient was on constant bed rest except at mealtimes.

Approximately 3 years after the wound appeared, MPPT treatment was instigated as last resort. It quickly reduced smell, exudate level, size and depth. Unfortunately, it was clear from the beginning that the wound bed contained gorges. These are characteristic for wounds that serve as exit areas, at the surface of the body, for fistulas draining infectious debris originating in a primary source of infection in non-soft tissue, such as an osteomyelitis - or less often, an anal fistula. Chronic osteomyelitis can only be treated surgically. While awaiting surgery, the skin and soft tissue were maintained without thinning and without infection, including the lining of the narrow gorges used for draining. There was no smell, and the exudate was minimal and consistent with an osteomyelitis. The patient was not on bed rest.

Osteomyelitis spreads via the blood, including to the lungs (Iwanaga et al., 2013)). Unfortunately, a little over a year after starting MPPT, while awaiting operation, the patient developed a pulmonary infection that, presumably combined with the osteomyelitis, caused fatal sepsis.

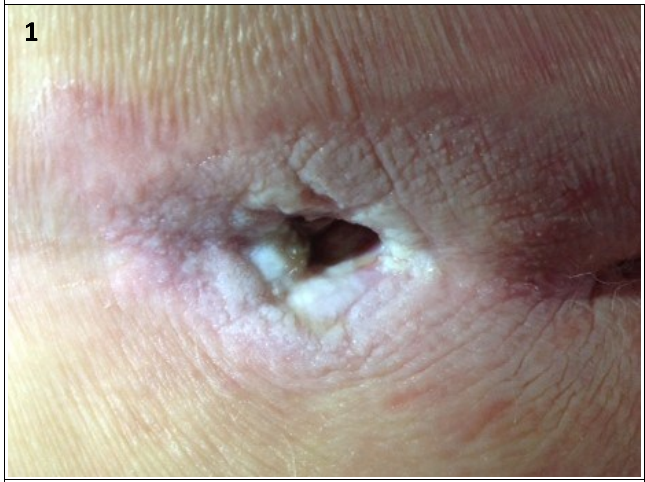

Day 0

Just before first MPPT

Nodulous wound edges and a broad band of damaged skin beyond the opening.

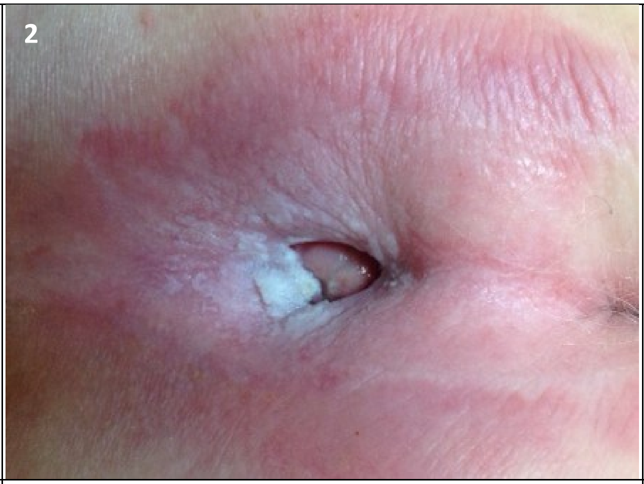

Day 25

Wound bed has granulated up from bottom. Grooves are detectable through the centre of the wound bed and along the edges. Skin is fully restored and with no nodulation.

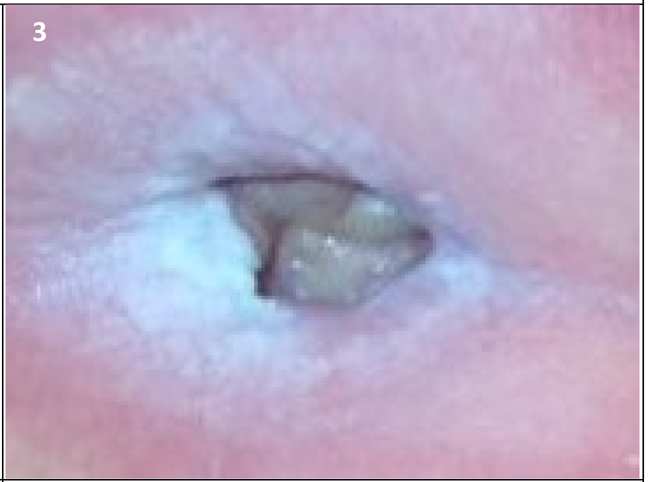

Day 49 Closeup

The wound bed has filled up further, practically to level. The gorges have cleaned up and become more distinct and easily recognisable.

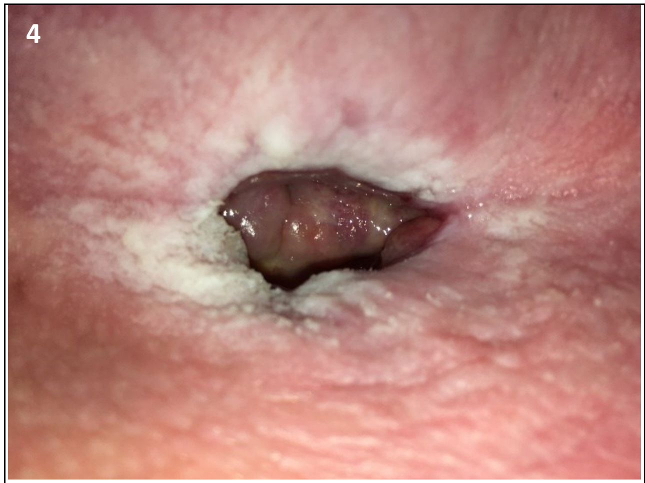

Day 247 8 months Closeup

(different camera & photographer)

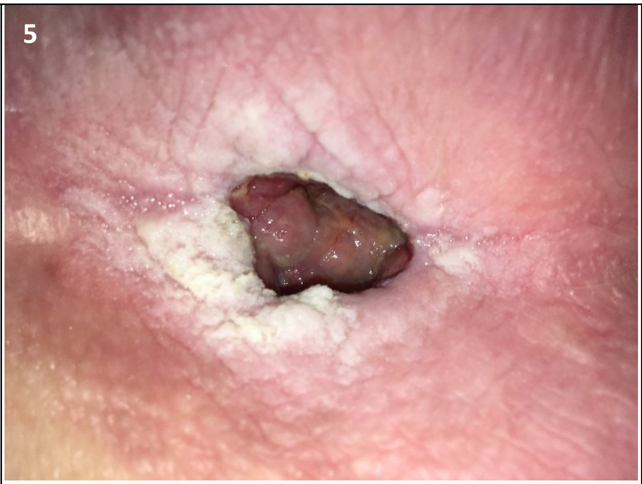

Day 356 12 months Closeup

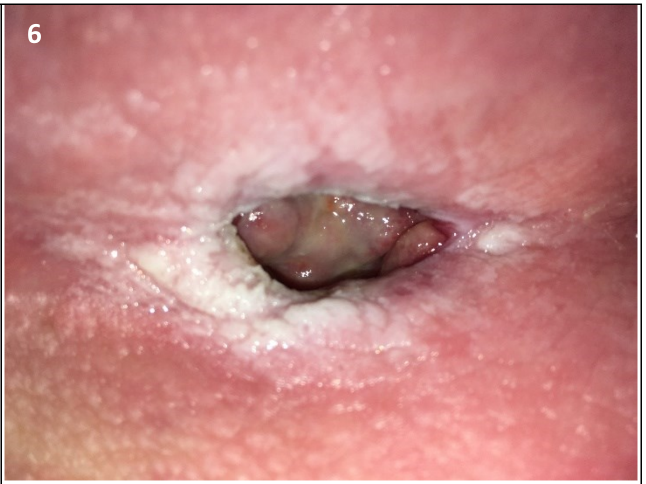

Day 401 13 months Closeup

Approximately a week before the patient passed away.

The skin and soft-tissue, including the lining of the gorges, are maintained free from infection and not declining, despite the deterioration in the osteomyelitis leading to fatal sepsis.

7

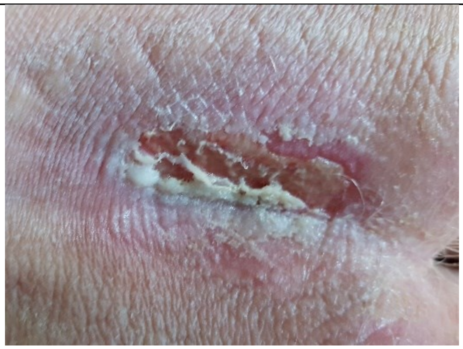

½ year after detection of the wound

2½ years before start MPPT

23.12.2017

How the wound looked approximately half a year after its first detection and 2.5 years before MPPT treatment was initiated. It was still a grade 3 ulcer.

Wound number 43			Patient	SCI	
**Grade 4**	**6 months old**	**Ischial tuberosity**	**60-year-old female**	**12 years**	**C6**

This pressure ulcer had for the past 6 months been washed with a cleanser containing surfactants in 15% glycerol (QV Gentle Wash) with glycerol being antibacterial and antiviral. For the past 3 months, it had been dressed with a petroleum jelly contact layer dressing (Urgotul) (petroleum jelly is an oestrogen-mimic) and for the past month additionally with liquid honey (Surgihoney). It was always covered with a polyurethane foam dressing (Biatain).

The wound presented with a very thick "cap" of tough slough mixed with necrotic tissue hiding a very extensive and severe infection underneath (pic 1). Within 24 hours the uppermost layer had been lifted; two hidden abscesses had been penetrated and evacuated; and the wound was in the process of expulsing red-pigmented bacterial toxins (pic 2). Over the following weeks, very tough, dry, deeply embedded slough would be loosened and disposed of, and further abscesses would be evacuated whilst the wound bed would granulate and regenerate soft tissue concomitantly. The early stages of these processes are exemplified in pic 3. Pic 4 shows the very peak of the yellow, old slough and red toxin expulsion. The speed of change is exemplified in the picture taken 24 hours later (pic 5.) One week later, (pic 6) the immune system was clearly controlling the slough and infection removal as evidenced by the reduction in wound size and overall healthier colour and structure of the wound bed. Unfortunately, the patient became non-compliant and a few weeks later withdrew from the trial for personal reasons.

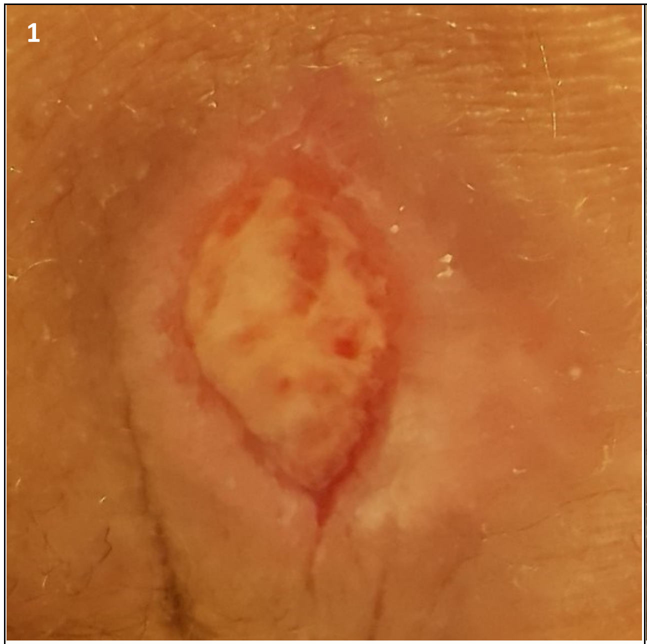

Day 0

Just before first MPPT

Thick slough acting as a lid. Wound edges and surrounding skin are dull and lifeless.

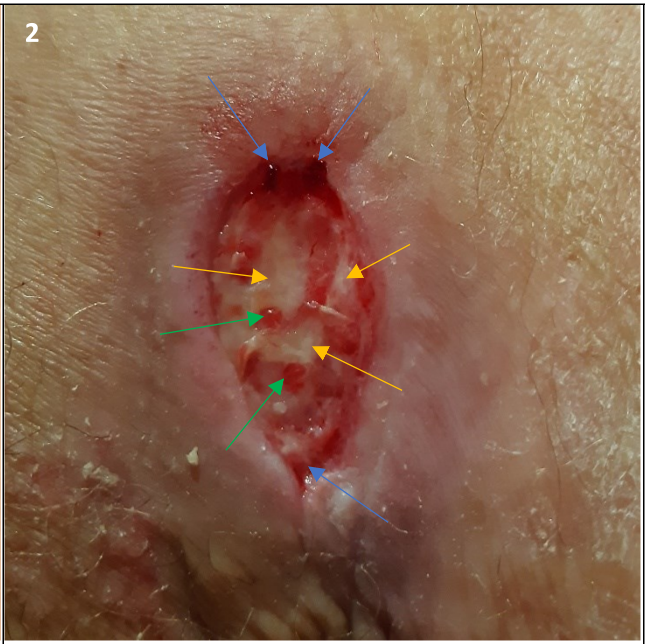

Day 1

The dried slough “lid” has disappeared.

Blue arrows: distinct red- pigmented-toxin expelling openings.

Yellow arrows: tough embedded slough

Green arrows: granulation

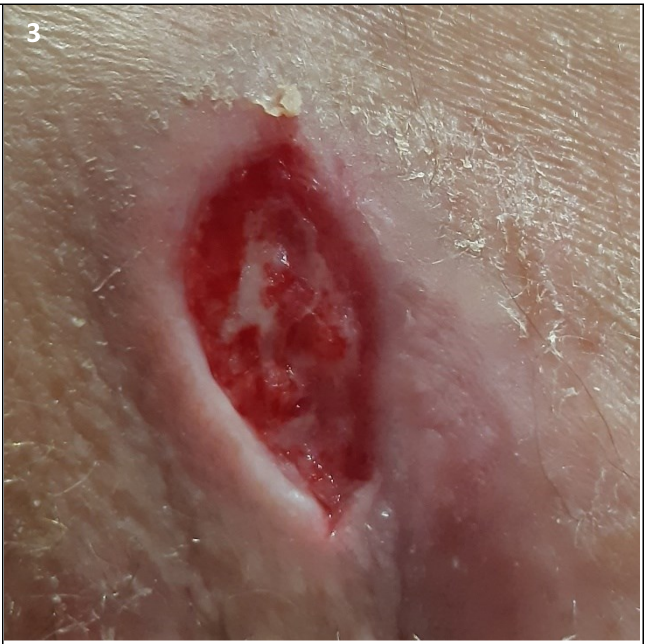

Day 10

Wound bed is less sloughy. The tissue is partly granulation but the brightness of the red colour shows that bacterial pigments are plentiful, as well. Their presence is down to them being transported to the wound surface

The surrounding skin is still lifeless.

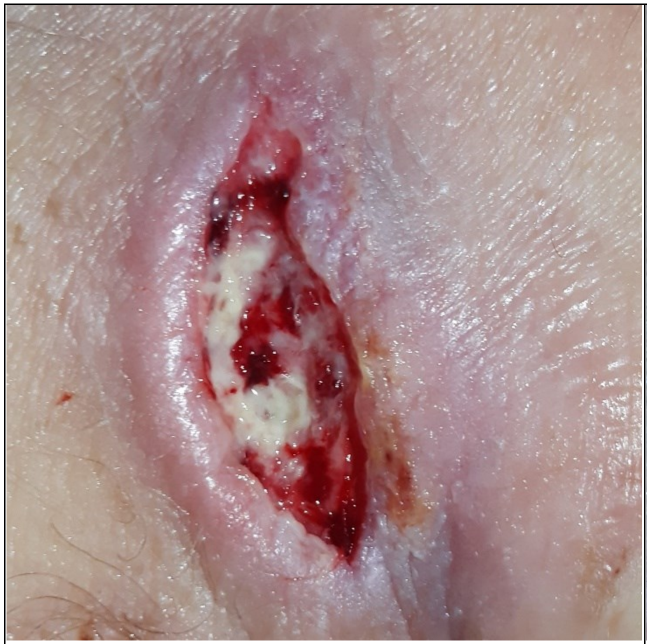	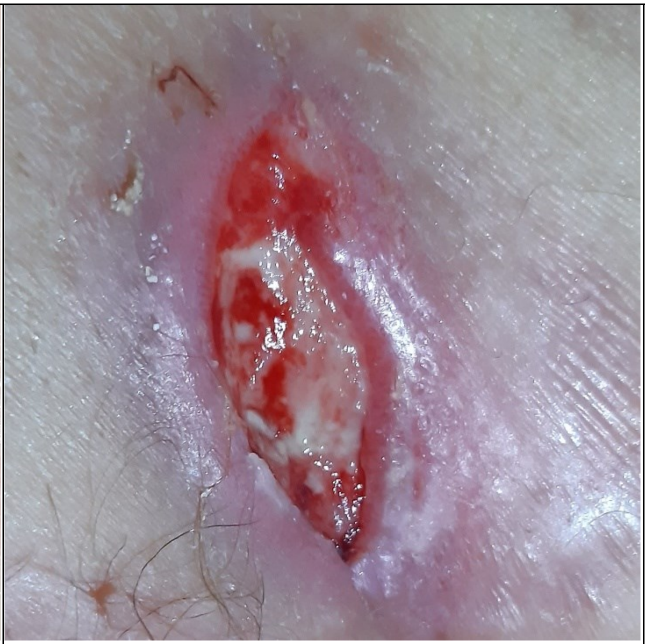	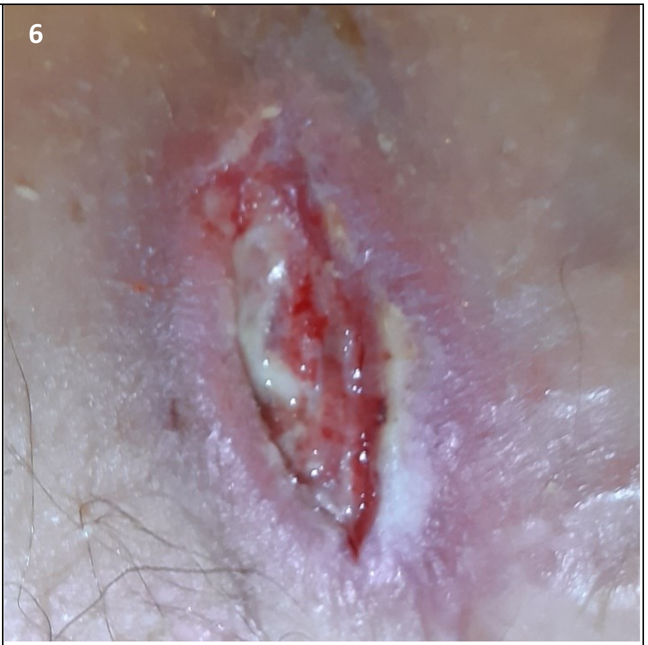
**Day 36**	**Day 37**	**Day 46**
Picture caught just when evacuation of at least 7 abscesses containing red-pigmented-toxins and 2 focal points dominated by yellow-toxin producing microbes are in the process of evacuating and depositing the toxic debris on the wound surface.<br><br>The surrounding skin is reacting and active.	24 hours later. Shows the speed of the healing after the harmful substances are removed.	The wound is considerably smaller.<br><br>The slough is soft and easily removable – no longer embedded.<br><br>The red colour of the wound bed is made up of a larger proportion of granulation and less of red bacterial pigment – compared to day 10 and 37.<br><br>The edges are epithelializing in most places and the surrounding skin is active and contracting.

Wound number 44			Patient	SCI	
**Grade 4**	**18 months old**	**Sacrum – Gluteal fold**	**44-year-old female**	**21 years**	**Tetraplegic**

A pressure ulcer tracking 9.5 cm in the intergluteal cleft area had been identified 18 months prior upon emergency hospitalisation due to sepsis. After 12 months of complete bedrest and washing the wound daily with Betaine-surfactants and 0.1 % PHMB (Prontosan) and packing with sodium carboxymethylcellulose hydrofiber ribbon (Aquacel) interrupted by two unsuccessful attempts of NPWT, the ulcer presented as a 9.5 cm deep sinus, 2 cm in diameter, expanding throughout and widening along the bone (pic 1). Flap surgery was performed (pic 2). No bone debridement was performed, and no bone biopsy was taken. The surgery healed relatively well except in the area of the original ulcer. The patient remained on full bedrest in hospital for 3 months. For the duration of the 3-month-period, the patient was administered Doxycycline on suspicion of osteomyelitis and the troublesome area was re-sutured 4 times during this period. However, the area refused to heal and the patient was discharged into community care.

Here, the patient remained on full bedrest, and the wound was dressed with antimicrobial nanocrystalline silver barrier dressing (Acticoat) for 6 weeks. The cranial end of the wound clearly exposed tunnel formation (pic 3). The dressing regime was changed to cadexomer-iodine polyethylene (Iodoflex) for 5 weeks. The tunnel entrances widened and deepened making them more evident, and the soft tissue was generally burnt (pic 4).

At this point, 6 months following surgery, the treatment regime was changed to MPPT. The sinuses were photographed before first MPPT application (pic 5). These tunels were a clear indication of a deep infiltrating infection draining its debris material into the wound thereby preventing closure. During the month MPPT was used daily, it accessed numerous abscesses and evacuated green, bright red, yellow and brown pigmented toxins, respectively, as well as sloughy debris, embedded in the adjacent soft tissue. This process seemed to widen the skin area, into which the fistulas drained, ever so slightly for a few weeks of intense clean-out efforts. It proceeded to leave the area of the exiting fistulas free of infection and granulating, i.e. building up new tissue from the bottom, and the individual fistulas only recognisable with the naked eye as the gorge structure in the soft tissue inevitably present when draining osteomyelitic debris (pic 6). The remainder of the wound, i.e. the long groove without apparent sinus exits, epithelialized (pic 7). This treatment period enabled the immune response to continue the healing process over the following months, when the wound was only washed with tap-water and covered with a cotton gauze, and MPPT used sporadically, i.e. approximately once weekly (pic 7 & 8). For personal reasons, the patient decided to discontinue the use of MPPT while awaiting surgery for osteomyelitis.

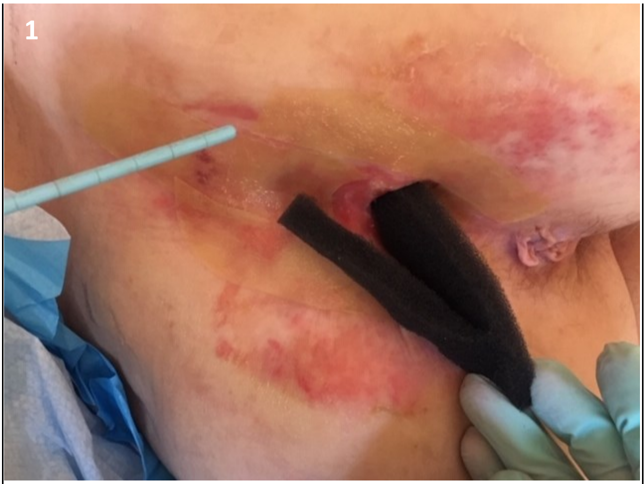

6.5 months after first detection of the ulcer in hospital due to sepsis.

5.5 months before surgery

11 months before MPPT

NPWT foam being fitted into the 9.5 cm deep sinus, 20 mm in diameter and expanding at the bottom along bone.

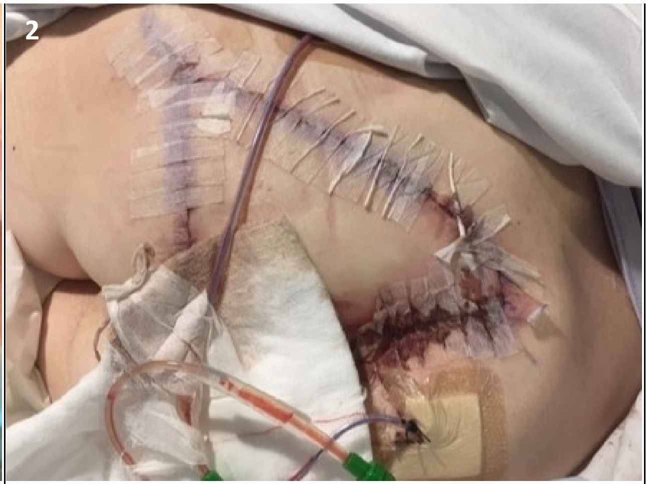

Just after flap surgery

6 months before MPPT

Flap surgery. No bone debridement. To be followed by 3 months in-hospital antibiotics.

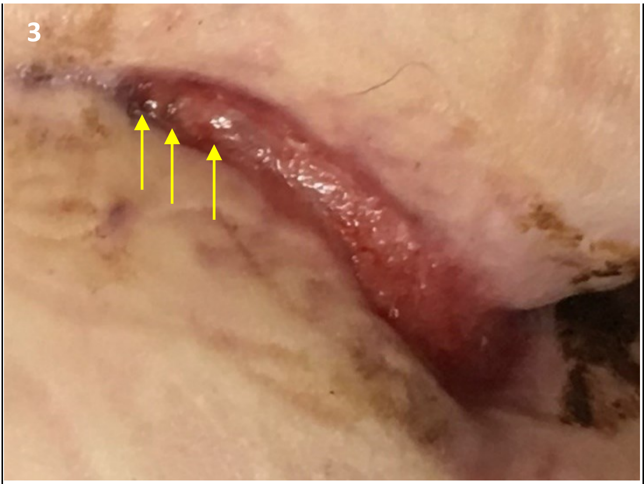

Day minus-40

4.5 months after surgery

1.5 month before start of MPPT

A 2 mm deep, 70 mm long and 20 mm broad groove wound seemingly carved by the corrosive infectious exudate originating in the osteomyelitic bone and reaching the surface through the 3 distinct pinprick sized tunnel openings at the top end.

Following 6 weeks of silver dressings.

Yellow arrows: the 3 tunnels, draining the debris.

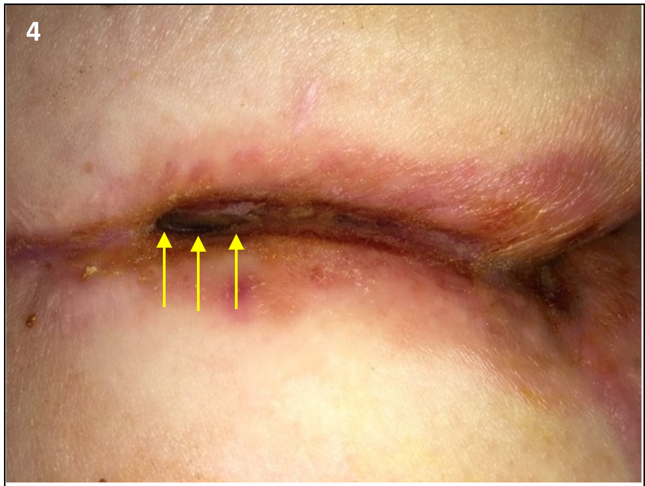

Day minus-6

1 week before start of MPPT

5.5 months after surgery

Following 5 weeks of iodine dressings. The area with the 3 tunnels is deepening, now 8 mm deep. Due to burning of the groove and the surrounding skin, the wound is taken off the current dressing regime for the following week, until MPPT is started.

Yellow arrows: the 3 fistulas draining the waste.

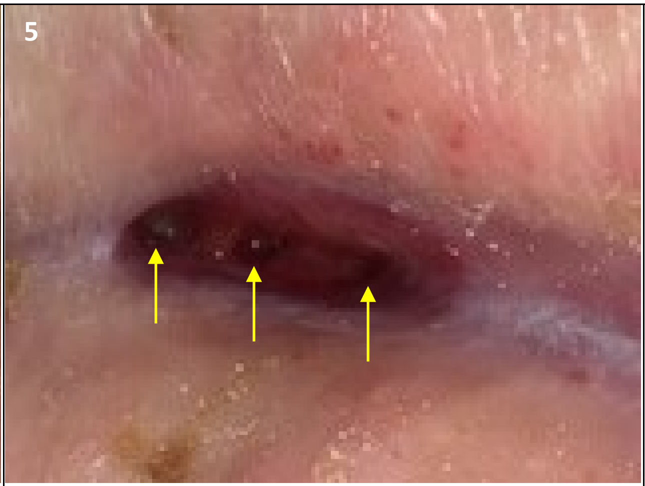

Day 0 Closeup of sinus-area

First day with MPPT

6 months after surgery

3 very distinct openings at the bottom of the wound bed.

The wound bed and draining fistulas are slightly brownish and the surrounding skin looks dull and affected.

Yellow arrows: the 3 fistulas draining the waste.

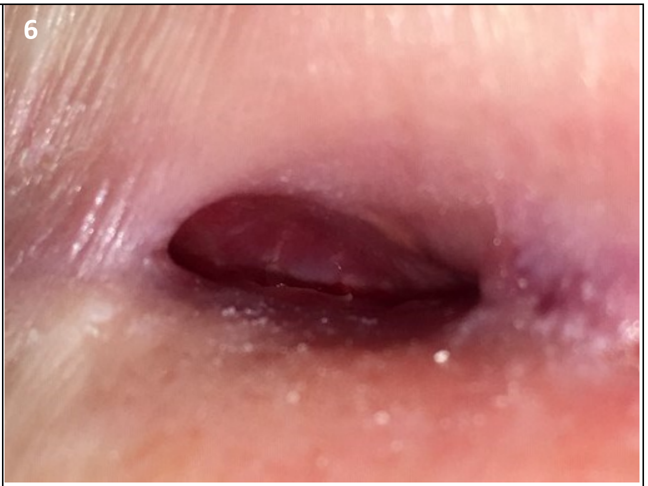

Day 27 Closeup of sinus-area

Non-viable tissue and soft tissue infection has been removed; the wound bed is regenerating despite the necessary draining of infectious osteomyelitis debris into the area. The draining fistulas have been cleaned out and reduced in diameter so that their existence is only recognisable as a gorge.

The skin is no longer affected.

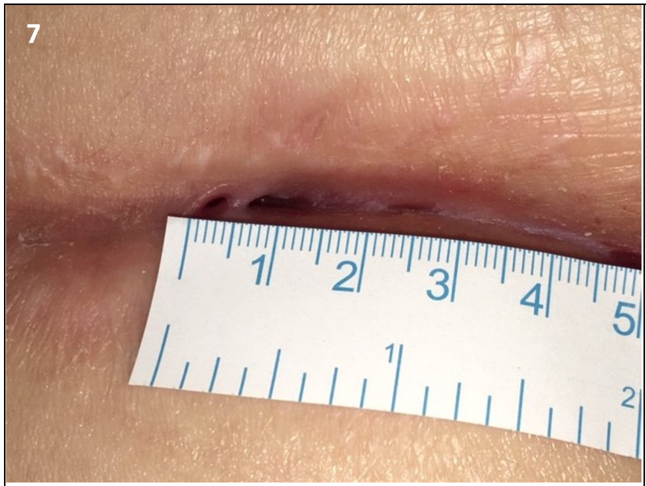

Day 43

1 month with MPPT followed by 1 week washing only with tap water and allowing air access

Regeneration of the soft tissue continues, despite the discontinuation of the MPPT. The groove is epithelializing.

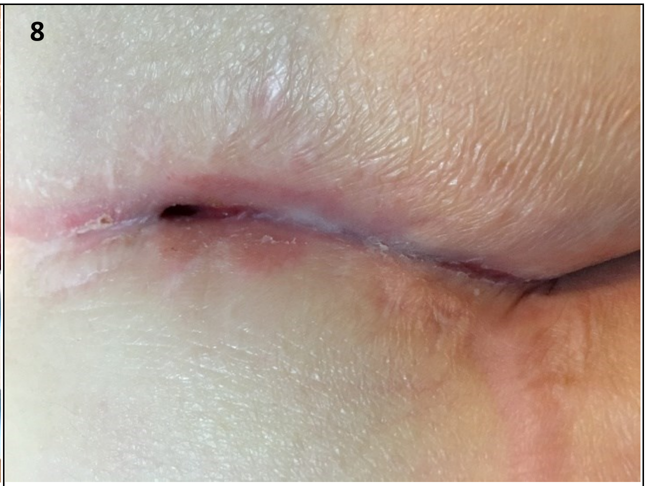

Day 91

3 months after first MPPT

MPPT only once weekly.

The last 2 months, MPPT was only used intermittently, i.e. approximately once a week. The groove has epithelialized and fused 55 mm of the groove, and reduced the associated wound to a 15mm long, 2 mm broad opening in the skin. This fistula-area shows a granulating shallow interior containing the gorge which drains the debris from the bone infection. All soft tissue is without infection, and the surrounding skin is non-infiltrated and healthy.
